# Synthesis of *gem*-Difluorocyclobutanes: Organolanthanum Enabled Synthesis and Divergent Catalytic Functionalization of *gem*-Difluorocyclobutanols

**DOI:** 10.1021/acs.joc.5c01175

**Published:** 2025-07-10

**Authors:** Hikaru Ishikura, Juan J. Rojas, Callum S. Begg, Chulho Choi, James A. Bull

**Affiliations:** † Molecular Sciences Research Hub, Department of Chemistry, 4615 Imperial College London, White City Campus, Wood Lane, London W12 0BZ, U.K.; ‡ Medicine Design, Pfizer Inc., Eastern Point Rd., Groton, Connecticut 06340, United States

## Abstract

Fluorinated cycloalkyl motifs continue to receive intense interest in medicinal chemistry due to their potential to modulate physicochemical properties. In recent years, this interest has extended to *gem*-difluorocyclobutanes as small, polar, yet lipophilic moieties. However, strategies to access *gem*-difluorocyclobutanes remain limited, presenting opportunities for the development of new synthetic methods. Here, we report the synthesis and divergent functionalization of *gem*-difluorocyclobutanols to generate a diverse range of 1,1-disubstituted-3,3-difluorocyclobutanes. The use of organolanthanum reagents is crucial to achieve the addition of carbon nucleophiles to commercially available difluorocyclobutanone to avoid the undesired elimination of HF by controlling nucleophile basicity. The generated difluorocyclobutanols enable functionalization through carbocation and radical intermediates, providing diverse 1,1-disubstituted difluorocyclobutanes and expediting access to new design options for medicinal or materials chemistry.

## Graphical abstract

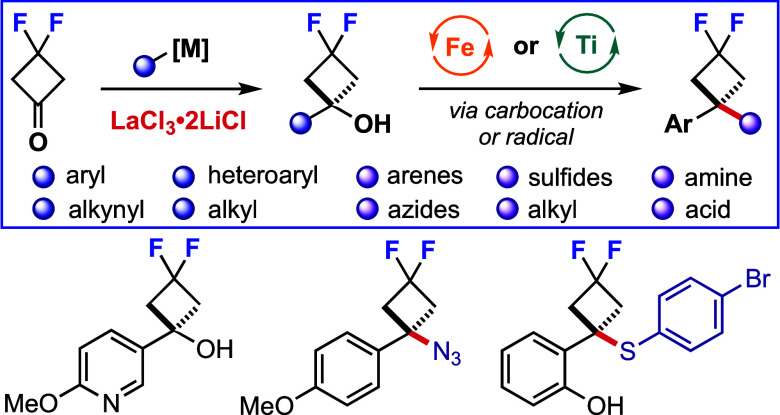

## Introduction

Fluorinated motifs are prevalent in materials, agrochemical, and medicinal chemistry industries.^1^ In drug discovery, the introduction of a fluorine atom into a scaffold can modulate crucial physicochemical properties such as p*K*_a_, lipophilicity, or solubility, as well as often improve the metabolic profile of a compound in development.^2^ Fluorine atoms have also been shown to potentially improve potency through weak noncovalent interactions with biological targets.^3^ At the same time, cyclobutanes are important structural motifs in bioactive small molecules and natural products.^4^ The strained yet stable structure coupled with advances in synthetic tractability has led to broader application by medicinal chemists and successful inclusion in 10 FDA-approved drugs.^5^ Among these, fluciclovine and ivosidenib contain fluorinated cyclobutanes,^6,7^ combining the beneficial properties of fluorine and cyclobutane derivatives.^8,9^ In the development of Ivosidenib (Figure 1A), the *gem*-difluorocyclobutane motif was found to be crucial in increasing metabolic stability while maintaining potency.^7^ Similar trends were observed in the development of glutaminase-1 (GLS-1) inhibitor IPN60090 and melanin concentrating hormone receptor 1 inhibitor BMS-814580 (Figure 1A).^10,11^ In the latter case, 1,1-disubstitution of the *gem*-difluorocyclobutane proved crucial to block a metabolic weak spot while maintaining efficacy.^11^ Spirocyclic-1,1-disubstituted *gem*-difluorocyclobutanes have also been employed to stabilize bioactive conformations and lower the p*K*_aH_ of neighboring basic amidine sites, mitigating hERG inhibition and P-gp efflux of amidine-based β-secretase inhibitors, further illustrating their applicability in drug discovery programs.^12^ Despite the interest in 1,1-disubstituted *gem*-difluorocyclobutanes, recent synthetic strategies have predominantly focused on monosubstituted *gem*-difluorocyclobutanes.^13^ Deoxyfluorination of the preformed 3,3-disubstituted cyclobutanone is currently the only general strategy to access 1,1-disubstituted *gem*-difluorocyclobutanes (Figure 1B).^8,14^ In some cases, 1,1-disubstituted *gem*-difluorocyclobutanes are seen as single examples in a broader reaction scope.^15^ For example, He and Hartwig showcased an example of the Pd-catalyzed α-arylation of the difluorocyclobutane ester, while Grygorenko and co-workers demonstrated a similar transformation promoted by NaHMDS (Figure 1B).^16,17^ Thus, 1,1-disubstitued *gem*-difluorocyclobutanes present an underexplored and valuable motif for medicinal chemistry, where opportunities remain for synthetic innovation.

**Figure 1:**
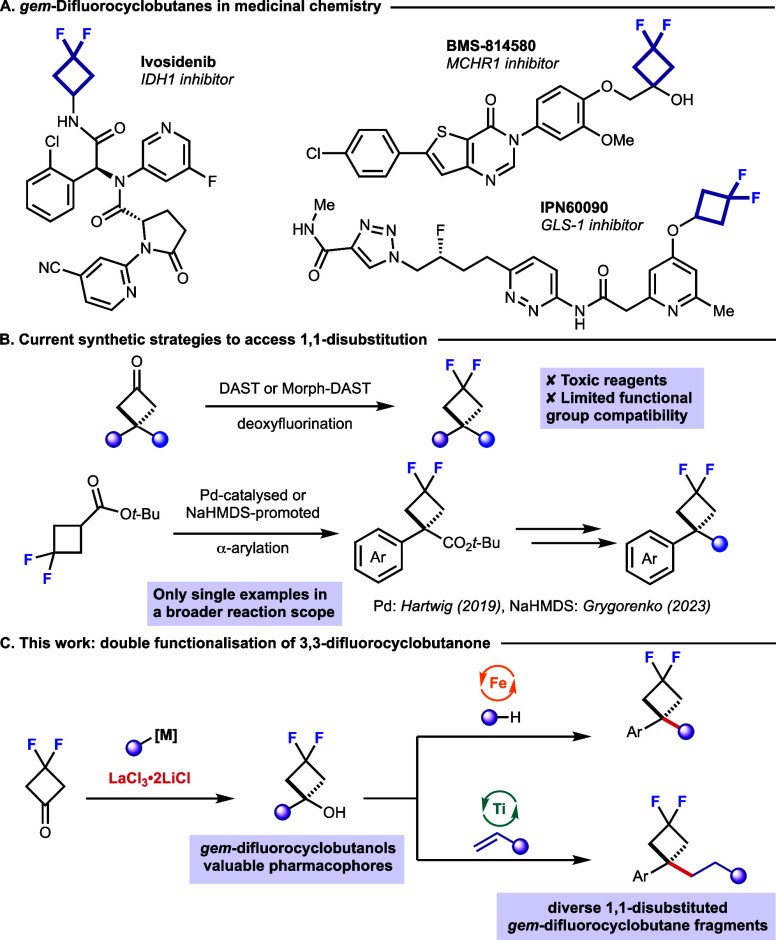
(A) *gem*-Difluorocyclobutane-containing bioactive molecules. (B) Current synthetic strategies to access 1,1-disubstituted *gem*-difluorocyclobutanes. (C) This work: double functionalization of 3,3-difluorocyclobutanone to access a diverse range of 1,1-disubstituted *gem*-difluorocyclobutanes.

Here, we report general approaches to the synthesis of *gem*-difluorocyclobutanes from difluorocyclobutanone that considerably expand access to this motif. 1-Substituted-difluorocyclobutan-1-ols are prepared through the use of organolanthanum reagents, overcoming the high sensitivity of difluorocyclobutanone to elimination. 1-Aryl-difluorocyclobutanols enable iron-catalyzed reactions with arene and thiol nucleophiles, invoking the formation of a difluorocyclobutane carbocation. We also demonstrate the direct formation of the difluorocyclobutane radical using low-valent titanium. These strategies allow for the rapid generation of a diverse set of 1,1-disubstituted difluorocyclobutanes, which display high stability and potential for further diversification, thus expanding the potential design options for medicinal chemists.

## Results and Discussion

We envisaged that 1,1-disubstituted difluorocyclobutanes could be efficiently prepared in a divergent manner through the application of commercially available 3,3-difluorocyclobutanone as a double electrophile, involving nucleophilic addition of organometallic reagents, followed by functionalization of the resulting tertiary alcohol through polar carbocation or radical intermediates. Therefore, we began our studies by investigating the addition of organometallic reagents to 3,3-difluorocyclobutanone, expecting nucleophilic addition with either organolithium or Grignard reagents would afford the desired difluorocyclobutanols, as has been described with other four-membered ring derivatives.^18–25^ However, scanning the literature revealed very few examples of organometallic additions to difluorocyclobutanone, with examples limited to patents reporting very low yields.^26–28^ Indeed, in our hands, treatment of the ketone with 4-methoxyphenyllithium gave only trace amounts of the desired difluorocyclobutanol **1** (6% yield, Table 1, entry 1; by contrast, the yield with cyclobutanone is typically ∼90%). Similar results were observed when the corresponding Grignard reagent was used, providing difluorocyclobutanol **1** in 14% yield, along with **2** and **3** as significant side-products in the reaction (Table 1, entry 2 and lower scheme).

**Table 1:**
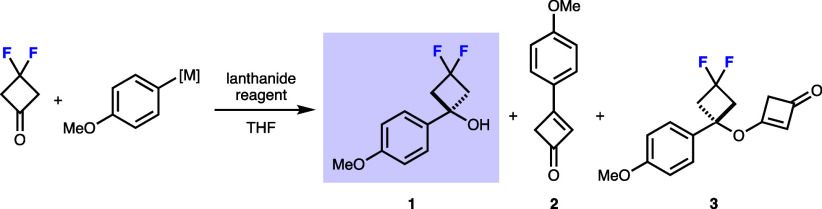

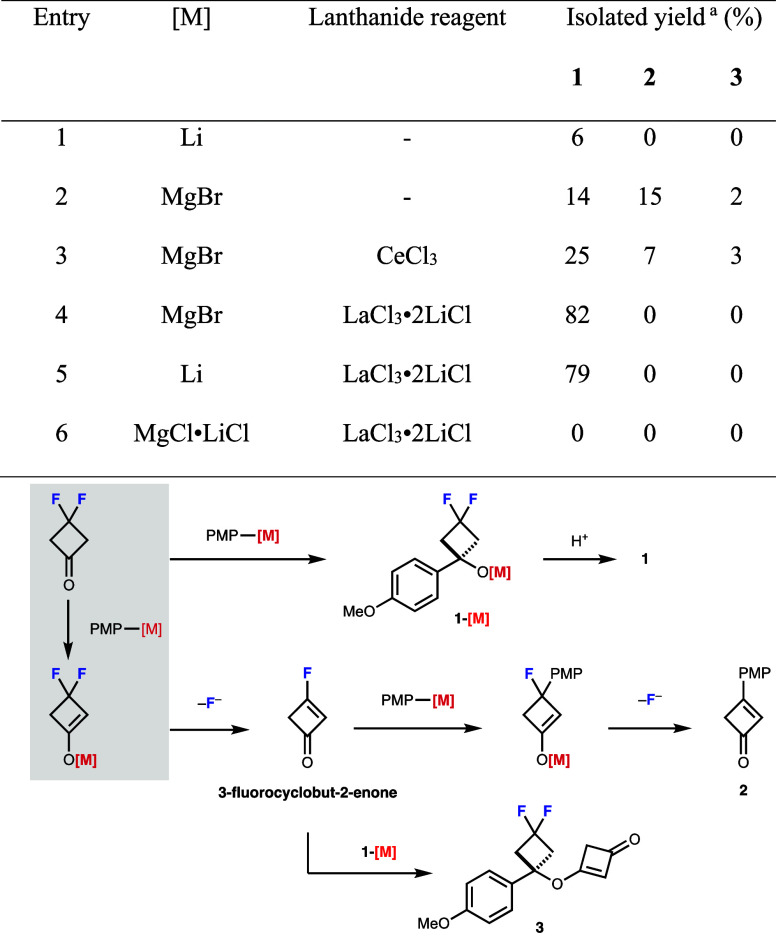
Selected Optimization for the Organometallic Addition to Difluorocyclobutanone

a On a 0.47 mmol scale. Full table in Supporting Information, Table S3.

The observation of small quantities of side products **2** and **3** indicated the cause of the different and unsuccessful outcome to be the marked increase in acidity of the α-protons in comparison to the nonfluorinated cyclobutanone (p*K*_a_ 20 in H_2_O).^9,29^ The formation of the side products was rationalized through, first, an E1_cb_ elimination to afford 3-fluorocyclobut-2-enone. An addition–elimination reaction involving either the generated excess organometallic reagent or difluorocyclobutanol anion affords **2** and **3,** respectively.

Given our envisaged central role of the difluorocyclobutanols to the strategy for further diversification, as well as being valuable pharmacophores themselves,^30^ we surveyed alternative reagents to promote 1,2-addition over competing α-deprotonation by controlling the nucleophile basicity. A variety of oxophilic and nonbasic organometallic reagents were investigated, including organolanthanide,^31^ organocerium,^32^ and organozincate reagents^33^ generated by transmetalation from organolithium or Grignard reagents, and reacted initially with cyclobutanone to ensure appropriate nucleophilic reactivity (see Table S1).^34^ CeCl_3_ and LaCl_3_·2LiCl were chosen as the best performing reagents to form organolanthanum and organocerium nucleophiles, respectively, and then applied with difluorocyclobutanone. The use of CeCl_3_ gave an improvement with the desired difluorocyclobutanol **1** formed in 25% yield, with trace amounts of **2** and **3** observed (Table 1, entry 3). Pleasingly, the use of LaCl_3_·2LiCl with 4-methoxyphenylmagnesium bromide exclusively gave the desired difluorocyclobutanol **1** in 82% isolated yield, with no formation of elimination products **2** or **3** (Table 1, entry 4). A similar reactivity was observed with the lanthanum reagent derived from the corresponding organolithium, affording the product in a 79% isolated yield (Table 1, entry 5). The importance of full transmetalation of the Grignard or organolithium reagent to form the organolanthanum reagent is noted, as simply prestirring the LaCl_3_·2LiCl with the ketone severely impacted the yield (Table S1, entry 3). Using the same conditions with a Turbo Grignard reagent resulted in no productive reaction and recovery of the protonated Grignard reagent (Table 1, entry 6).

With these conditions, we examined the scope of the organolanthanum additions to difluorocyclobutanone using more readily generated organolithium reagents and transmetalation with LaCl_3_·2LiCl (see Scheme 1). Electron-rich (**1**, **4**–**7**), electron-neutral (**8**), and electron-poor (**11**–**12**) aryl halides underwent lithium-halogen exchange, transmetalation, and addition in good yields. This could be extended to complex, medicinally relevant aryl halides such as the Celecoxib fragment (**13**). *p*-Chlorophenyl substrate **11** proved difficult, with elimination products observed under all tested conditions (Table S4), which we ascribe to interactions between the aryl chloride and LaCl_3_·2LiCl reducing the efficiency. Deprotection of TIPS-difluorocyclobutanols **4** and **6** using TBAF proceeded in excellent yields, revealing a phenolic handle for further functionalization. Electron-poor heteroaryl halides (**14**–**17**) required a slight adjustment in reaction conditions to accommodate for the instability of the generated pyridyllithium species, with the transmetalation commenced at −78 °C before warming to 0 °C.

**Scheme 1:**
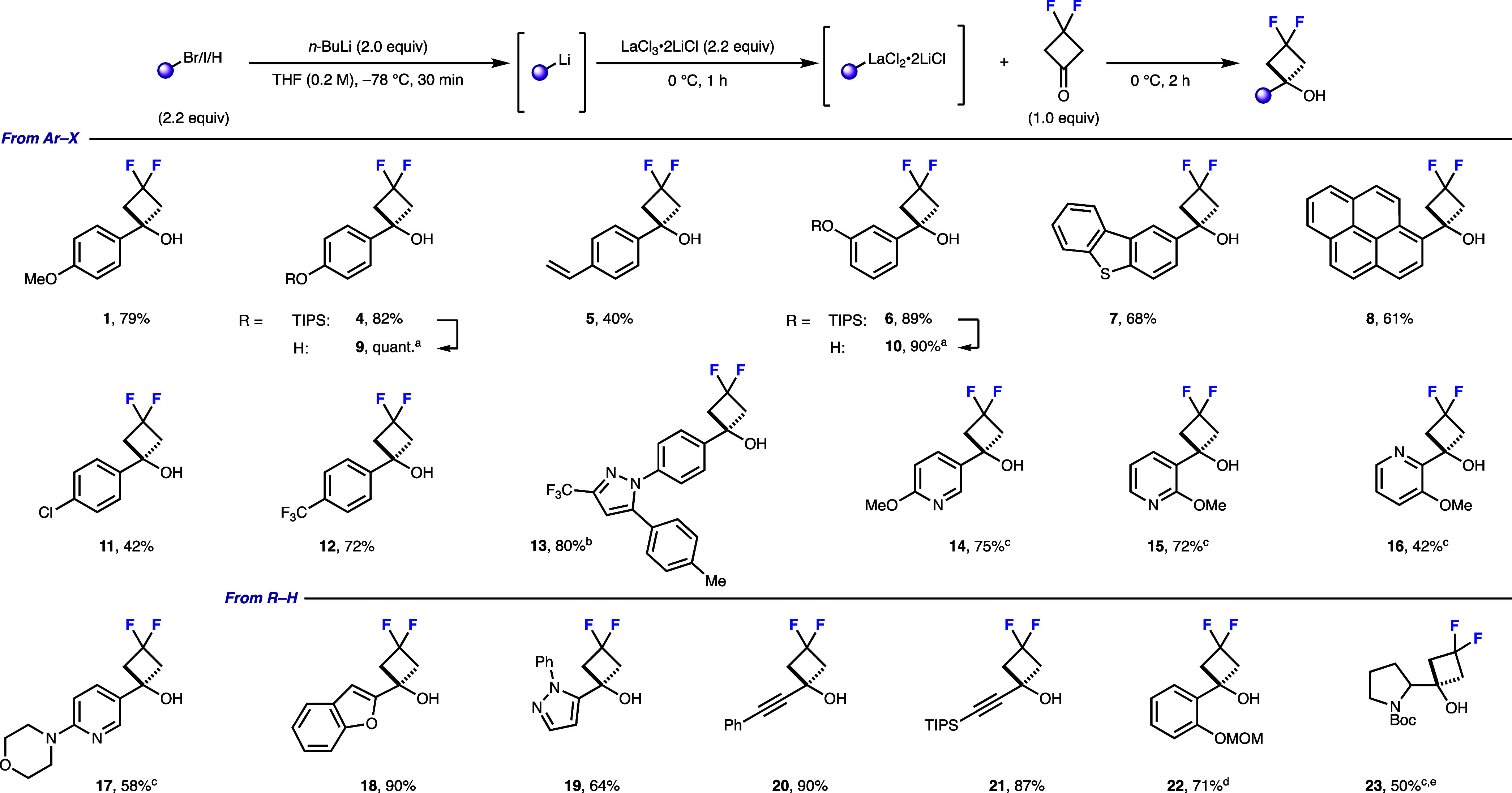
Preparation of Difluorocyclobutanols from Transmetalation of Organolithium Reagents to Organolanthanum Reagents^a^

a On a 0.5 mmol scale. ^a^Deprotection carried out with TBAF (1.2 equiv) in THF (0.2 M), rt, 1.5 h. ^b^Using *t*-BuLi (4.4 equiv). ^c^Transmetalation carried out at −78 °C for 1.5 h, followed by 0.5 h at 0 °C. ^d^Lithiation carried out at 0 °C for 1 h. ^e^Using *s*-BuLi (2.0 equiv)

Direct deprotonation with subsequent transmetalation of electron-rich heterocycles, such as benzofuran (**18**) and pyrazole (**19**), and alkynes (**20**–**21**) was also successful. Directed *ortho*-lithiation of MOM-protected phenol gave the desired difluorocyclobutanol **22** in good yield. Similarly, deprotonation of *N*-Boc pyrrolidine in the C2 position gave alkyl difluorocyclobutanol **23** as an analogue of proline. Overall, this approach provides significantly enhanced access to a broad range of difluorocyclobutanols by controlling the nucleophile basicity, paving the way for broader applications in synthetic and medicinal chemistry.

With a diverse set of valuable difluorocyclobutanols in hand, we turned our attention to their onward reactions to form 1,1-disubstituted difluorocyclobutanes. The generation of carbocation intermediates on four-membered rings and reaction with nucleophiles has been reported from cyclobutanols,^18,19d^ oxetanols,^21^ azetidinols,^22^ and thietanol dioxides.^25,35^ Here, we investigated conditions for the generation and trapping of a difluorocyclobutane carbocation with arene nucleophiles. To our delight, treatment of difluorocyclobutanol **1** with *o*-cresol and the Ca^2+^, Li^+^, or Fe^3+^ catalysts gave full conversion and near quantitative yields of the desired diaryl-difluorocyclobutane **24** (Table 2, entries 1–3), without promoting elimination. The H^+^ catalyst also gave full conversion and similarly excellent yields (Table 2, entry 4). No formation of side-products was observed in the ^1^H NMR spectrum of the crude reaction mixture. Using the inexpensive and abundant FeCl_3_ catalyst, the solvent was changed from dichloromethane to toluene, as a more environmentally and industrially acceptable alternative (Table 2, entry 5). The loading of the nucleophile could also be reduced from 5.0 to 3.0 equiv (Table 2, entry 6), presenting an attractive protocol for access to a diverse range of 1,1-disubstituted difluorocyclobutanes. To combat issues of solubility and reactivity across a broader substrate scope, we examined higher temperature conditions, which gave equally high yields with *o*-cresol and broadly beneficial effects with other nucleophiles (110 °C; entry 7).^36^

**Table 2:**
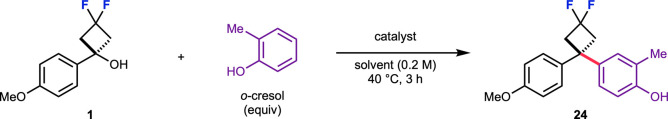
Selected Optimization for the Friedel–Crafts Reaction between Difluorocyclobutanol **1** and *o*-Cresol

entry^a^	catalyst (mol %)	solvent	equiv	yield 24 (%)^b^
1	Ca(NTf_2_)_2_ (5) + NBu_4_PF_6_ (5)	CH_2_Cl_2_	5.0	97
2	LiNTf_2_ (11) + NBu_4_PF_6_ (5.5)	CH_2_Cl_2_	5.0	97
3	FeCl_3_ (10)	CH_2_Cl_2_	5.0	100 (96)
4	HNTf_2_ (10)	CH_2_Cl_2_	5.0	95
5	FeCl_3_ (10)	PhMe	5.0	96
6	FeCl_3_ (10)	PhMe	3.0	95 (91)
7^c^	FeCl_3_ (10)	PhMe	3.0	96 (92)

a On a 47 μmol scale.

b Yield determined by ^1^H NMR spectroscopy using 1,3,5-trimethoxybenzene as an internal standard. Isolated yields in parentheses.

c At 110 °C.

Under the more general higher temperature conditions, the scope of the Friedel–Crafts reaction was explored by varying the difluorocyclobutanol substrate and arene nucleophiles (Scheme 2). Varying the *para*-substitution from a methoxy group to a hydroxy group (**25**) gave a slight increase in yield, while including an OTIPS group in the same position gave the desired product **26** with no observed deprotection. A change to the electron-withdrawing *p*-chlorophenyl group also well-tolerated **27**, although the presence of stronger electron-withdrawing substituents such as *p*-trifluoromethyl only gave trace amounts of the product. Substrates with substitution at the *meta*-position (**28–29**), as well as benzofuran (**30**), pyrene (**31**), and dibenzothiophene (**32**) derivatives, were all successfully reacted. Only *m*-OTIPS difluorocyclobutanol **29** gave a 4:1 mixture of C4:C2 regioisomers; all other reactions proceeded with complete C4 selectivity. More complex aromatic substituents, such as **33,** were also successful, providing a route to medicinally relevant 1,1-diaryl difluorocyclobutanes.

**Scheme 2:**
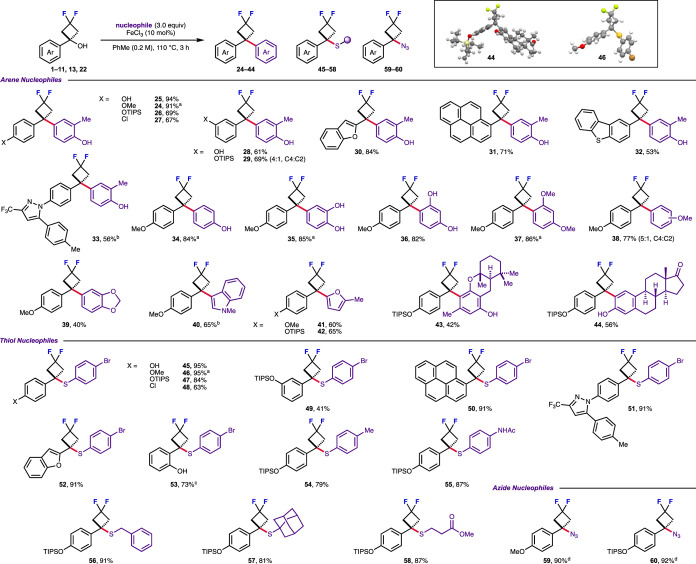
Reaction Scope of Difluorocyclobutanols with Arene, Thiol, and Azide Nucleophiles Using Iron Catalysis to Generate Carbocation Intermediates^a^

a Reactions performed on a 0.1 mmol scale. ^a^at 40 °C. ^b^ Reaction time of 24 h. ^c^ From difluorocyclobutanol **22**. ^d^FeCl_3_ (10 mol %), TMSN_3_ (1.2 equiv), MeCN (0.2 M), 40 °C, 24 h.

Phenol, catechol, and resorcinol were all successful nucleophiles, affording diaryl difluorocyclobutanes **34**–**36** in good yields with complete C4 regioselectivity. *o*-Cresol gave diaryl difluorocyclobutane **24** in 90% yield on a larger 0.3 mmol scale. Nonphenolic nucleophiles, such as 1,3-dimethoxybenzene, were also successful in good yields. Anisole and benzodioxole were reactive (**38**–**39**), although anisole gave a 5:1 mixture of C4:C2 regioisomers. Heterocycle nucleophile *N*-methylindole **40** gave the desired product in 65% yield under 110 °C conditions. 2-Methylfuran was also successful (**41**). Complex polysubstituted phenols, a meroterpenoid analogue, and estrone gave the corresponding difluorocyclobutane appended products (**43, 44**).

The same catalytic system was successful with thiol nucleophiles, affording 3-sulfanyl difluorocyclobutanes. A variety of aryl-difluorocyclobutanols were compatible (**45**–**52**), with variation in the electronics of the arene as well as pyrazole and benzofuran containing substrates. Interestingly, the use of MOM-protected derivative **22** resulted in complete MOM-deprotection to form sulfanyl difluorocyclobutane **53** as the free phenol. Thiophenols, benzylic, and aliphatic thiols were successful nucleophiles (**54**–**58**). Aniline nucleophiles were unsuccessful, resulting only in the recovery of starting material. By switching the solvent to MeCN, TMSN_3_ could also be employed as the nucleophile to form the 3-azido difluorocyclobutanes **59** and **60**. Through these processes, a diverse array of difluorocyclobutane derivatives and building blocks were prepared, expanding the range of potential difluorocyclobutane-containing scaffolds that can be readily prepared.

Diaryl difluorocyclobutane **44** and sulfanyl difluorocyclobutane **46** were further characterized by single-crystal X-ray diffraction. The difluorocyclobutane ring is puckered in both cases, with a puckering angle of 23.6° and 19.9°, respectively. This is more planar in comparison to the nonfluorinated cyclobutanes, which are commonly puckered (∼30°), to minimize eclipsing interactions,^37^ but significantly more puckered than heterocyclic four-membered rings.^21,25,38^ The aryl groups all lie out of plane, almost perpendicular to the difluorocyclobutane (Ω = 89.9° and 92.5° for **44** and 89° for **46**), presumably to avoid steric clashing with the methylene groups on the difluorocyclobutane, in line with previous observations on four-membered rings. Interestingly, the sulfide **46** is held in a *gauche* conformation (dihedral angle: 64.6°) in contrast to a similar thietane dioxide sulfide, which adopts an anti-periplanar conformation (dihedral angle: 179.7°).^25^

The chemical stability of the synthesized 1,1-disubstituted difluorocyclobutanes was studied across a range of conditions using diaryl difluorocyclobutane **24** and difluorocyclobutane sulfide **46** as model substrates (Table S6). Quantitative recovery of the difluorocyclobutanes was observed across acidic and basic conditions, as well as in the presence of strong nucleophiles (NaI in acetone at 50 °C, H-Cys-OMe in DMF/H_2_O) and in buffer solution (phosphate buffered saline), indicating the high chemical stability of these motifs (see Table S6), appropriate for applications in medicinal chemistry programs involving diversification.

Functionalization of the generated 1,1-disubstituted difluorocyclobutane derivatives further demonstrated the stability of these motifs (Scheme 3). The phenol handle of difluorocyclobutanol **9** was alkylated with propargyl bromide in quantitative yields. Difluorocyclobutane carboxylic acid **62** was also prepared from 2-methylfuran difluorocyclobutane **41** by ruthenium-catalyzed oxidative cleavage.^39^ The same carboxylic acid was previously synthesized in four steps;^40^ however, it can now be accessed in two steps from difluorocyclobutanol **1**.

**Scheme 3:**
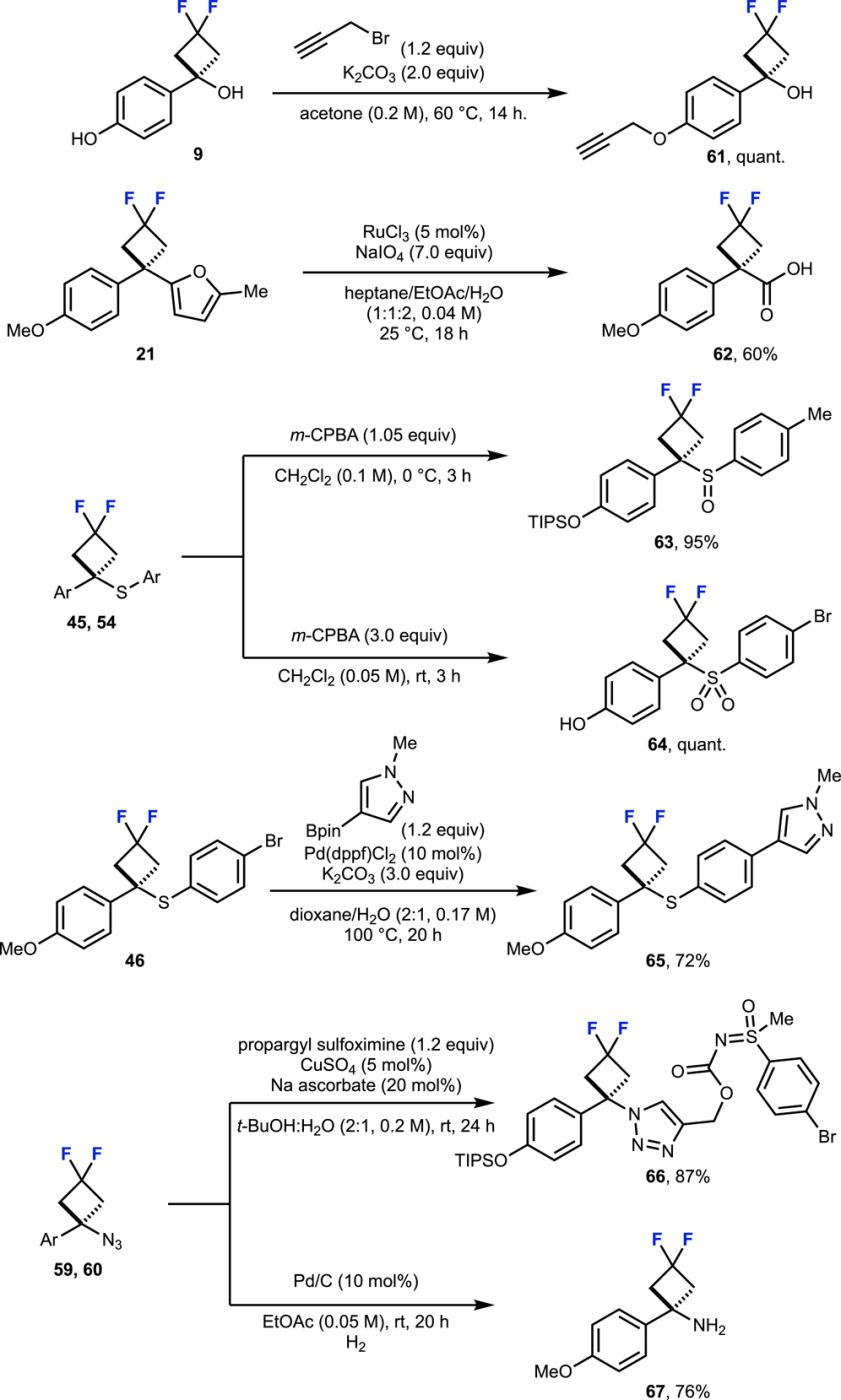
Further Functionalization of 1,1,-Disubstituted *gem*-Difluorocyclobutanes

Oxidation of the sulfide with *m*-CPBA selectively afforded the difluorocyclobutane sulfoxide and sulfone derivatives as novel difluorocyclobutane scaffolds. The aryl bromide handle on difluorocyclobutane sulfide **46** allowed for Suzuki–Miyaura cross-coupling, further demonstrating the stability of the difluorocyclobutane motif under varying reaction conditions. The difluorocyclobutane azide was amenable to click chemistry, allowing for the combination of pharmacophores such as sulfoximines to access new chemical space.^41^ Finally, reduction of the azide gave access to difluorocyclobutane amine **67** as a small, polar difluorocyclobutane building block. The success of these diversification strategies, widely employed in drug discovery campaigns, enhances the potential of the difluorocyclobutane motif to be applied across a broad range of molecular scaffolds.

We envisaged that difluorocyclobutanol derivatives could also act as radical precursors. Inspired by the work of Suga and Ukaji,^42^ we treated difluorocyclobutanol **1** with TiCl_4_·2THF in the presence of Mn and collidine (Scheme 4). Pleasingly, the difluorocyclobutanol underwent homolytic C–O bond cleavage to the difluorocyclobutane radical and subsequent Giese addition to acrylonitrile to afford alkyl difluorocyclobutane **68**. The same reaction with *p*-OTIPS-difluorocyclobutanol **4** gave similar yields. As demonstrated by Xie et al., tuning the electronic and steric features and reduction potential of the Ti center allows for the same transformation under a catalytic regime.^43^ The use of Cp*TiCl_3_ as the catalyst and Zn as a reductant gave the reaction of difluorocyclobutanol **1** with acrylonitrile (Scheme 4). These processes demonstrate the viability of radical reactions on the difluorocyclobutane ring for further diversification and access to new scaffolds.

**Scheme 4:**
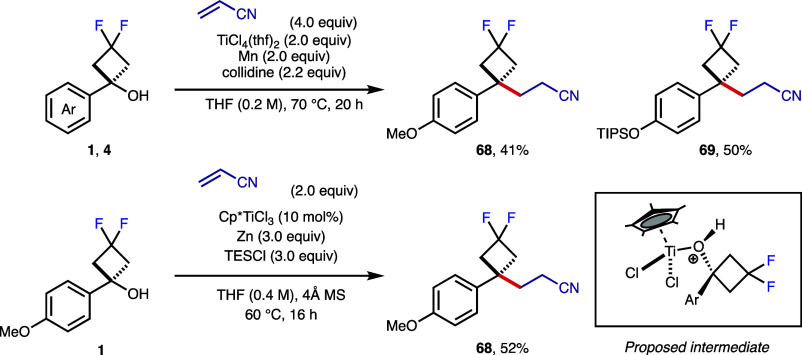
Low-Valent Titanium Catalyzed Giese Addition of Difluorocyclobutanols

## Conclusions

Difluorocyclobutanes present a valuable emerging motif for medicinal chemistry as small, polar, yet lipophilic moieties that are yet to be routinely investigated. We have developed methodologies to access sets of 1,1-disubstituted *gem*-difluorocyclobutanes using 3,3-difluorocyclobutanone. Unlike other four-membered ring ketones, 3,3-difluorocyclobutanone does not undergo productive reaction with organolithium or Grignard reagents. The use of organolanthanum reagents proved crucial in unlocking a route to *gem*-difluorocyclobutanol derivatives with aryl, alkynyl, and C­(sp^3^)-nucleophiles. 3-Aryl-difluorocyclobutanols provided building blocks to prepare 1,1-disubstituted difluorocyclobutanes through the reaction of the hydroxyl group. Iron chloride catalysis enabled the generation of carbocation intermediates that smoothly reacted with arene, thiol, and azide nucleophiles. Reactions through difluorocyclobutyl radicals were also demonstrated. This approach has provided rapid and divergent access to a range of 1,1-disubstituted difluorocyclobutanes. The synthesized difluorocyclobutane derivatives are stable under a range of chemical and reaction conditions, allowing for further functionalization and generation of valuable building blocks. We anticipate that these methods will provide opportunities for medicinal chemists to incorporate difluorocyclobutanes into the design of biologically active compounds in medicinal chemistry programs.

## Supplementary Material



## Data Availability

The data underlying this study are available in the published article, in its Supporting Information, and openly available in Imperial College London Research Data Repository at 10.14469/hpc/15093.

## Notes

Detailed optimization tables; experimental procedures, characterization data, X-ray crystallographic data, and copies of ^1^H, ^13^C, and ^19^F spectra (PDF)

## References

[1] Berger R., Resnati G., Metrangolo P., Weber E., Hulliger J. (2011). Organic Fluorine Compounds: A Great
Opportunity for Enhanced Materials Properties. Chem. Soc. Rev..

[2] Gillis E. P., Eastman K. J., Hill M. D., Donnelly D. J., Meanwell N. A. (2015). Applications of Fluorine in Medicinal Chemistry. J. Med. Chem..

[3] Dalvit C., Invernizzi C., Vulpetti A. (2014). Fluorine as a Hydrogen-Bond
Acceptor: Experimental Evidence and Computational Calculations. Chem.—Eur. J..

[4] Hui C., Liu Y., Jiang M., Wu P. (2022). Cyclobutane-Containing
Scaffolds in Bioactive Small Molecules. Trends
Chem..

[5] Bauer M. R., Di Fruscia P., Lucas S. C. C., Michaelides I. N., Nelson J. E., Storer R. I., Whitehurst B. C. (2021). Put a Ring
on It: Application of Small Aliphatic Rings in Medicinal Chemistry. RSC Med. Chem..

[6] Shoup T. M., Olson J., Hoffman J. M., Votaw J., Eshima D., Eshima L., Camp V. M., Stabin M., Votaw D., Goodman M. M. (1999). Synthesis and Evaluation of [18F]­1-Amino-3-Fluorocyclobutane-1-Carboxylic
Acid to Image Brain Tumors. J. Nucl. Med..

[7] Popovici-Muller J., Lemieux R. M., Artin E., Saunders J. O., Salituro F. G., Travins J., Cianchetta G., Cai Z., Zhou D., Cui D., Chen P., Straley K., Tobin E., Wang F., David M. D., Penard-Lacronique V., Quivoron C., Saada V., De Botton S., Gross S., Dang L., Yang H., Utley L., Chen Y., Kim H., Jin S., Gu Z., Yao G., Luo Z., Lv X., Fang C., Yan L., Olaharski A., Silverman L., Biller S., Su S.-S. M., Yen K. (2018). Discovery of AG-120 (Ivosidenib): A First-in-Class
Mutant IDH1 Inhibitor for the Treatment of IDH1Mutant Cancers. ACS Med. Chem. Lett..

[8] Grygorenko O. O., Volochnyuk D. M., Vashchenko B. V. (2021). Emerging Building Blocks for Medicinal Chemistry: Recent
Synthetic Advances. Eur. J. Org Chem..

[9] Holovach S., Melnykov K. P., Skreminskiy A., Herasymchuk M., Tavlui O., Aloshyn D., Borysko P., Rozhenko A. B., Ryabukhin S. V., Volochnyuk D. M., Grygorenko O. O. (2022). Effect
of *gem*-Difluorination on the Key Physicochemical
Properties Relevant to Medicinal Chemistry: The Case of Functionalized
Cycloalkanes. Chem.—Eur. J..

[10] Soth M. J., Le K., Di Francesco M. E., Hamilton M. M., Liu G., Burke J. P., Carroll C. L., Kovacs J. J., Bardenhagen J. P., Bristow C. A., Cardozo M., Czako B., De Stanchina E., Feng N., Garvey J. R., Gay J. P., Do M. K. G., Greer J., Han M., Harris A., Herrera Z., Huang S., Giuliani V., Jiang Y., Johnson S. B., Johnson T. A., Kang Z., Leonard P. G., Liu Z., McAfoos T., Miller M., Morlacchi P., Mullinax R. A., Palmer W. S., Pang J., Rogers N., Rudin C. M., Shepard H. E., Spencer N. D., Theroff J., Wu Q., Xu A., Yau J. A., Draetta G., Toniatti C., Heffernan T. P., Jones P. (2020). Discovery of IPN60090, a Clinical
Stage Selective Glutaminase-1 (GLS-1) Inhibitor with Excellent Pharmacokinetic
and Physicochemical Properties. J. Med. Chem..

[11] Devasthale P., Wang W., Mignone J., Renduchintala K., Radhakrishnan S., Dhanapal J., Selvaraj J., Kuppusamy R., Pelleymounter M. A., Longhi D., Huang N., Flynn N., Azzara A. V., Rohrbach K., Devenny J., Rooney S., Thomas M., Glick S., Godonis H., Harvey S., Cullen M. J., Zhang H., Caporuscio C., Stetsko P., Grubb M., Huang C., Zhang L., Freeden C., Murphy B. J., Robl J. A., Washburn W. N. (2015). Non-Basic
Azolotriazinone MCHR1 Antagonists for the Treatment of Obesity: An
Empirical Brain-Exposures-Driven Candidate Selection for in Vivo Efficacy
Studies. Bioorg. Med. Chem. Lett..

[12] Fujimoto K., Yoshida S., Tadano G., Asada N., Fuchino K., Suzuki S., Matsuoka E., Yamamoto T., Yamamoto S., Ando S., Kanegawa N., Tonomura Y., Ito H., Moechars D., Rombouts F. J. R., Gijsen H. J. M., Kusakabe K. (2021). Structure-Based
Approaches to Improving Selectivity through Utilizing Explicit Water
Molecules: Discovery of Selective β-Secretase (BACE1) Inhibitors
over BACE2. J. Med. Chem..

[13] Melnykov K., Granat D., Volochnyuk D., Ryabukhin S., Grygorenko O. (2018). Multigram Synthesis of C4/C5 3,3-Difluorocyclobutyl-Substituted
Building Blocks. Synthesis.

[14] Chernykh A. V., Feskov I. O., Chernykh A. V., Daniliuc C. G., Tolmachova N. A., Volochnyuk D. M., Radchenko D. S. (2016). Synthesis of Fluorinated Building Blocks Based on Spiro[3.3]­Heptane
Scaffold. Tetrahedron.

[15] Chu L., Armstrong H. M., Chang L. L., Cheng A. F., Colwell L., Cui J., Evans J., Galka A., Goulet M. T., Hayes N., Lo J., Menke J., Ok H. O., Ondeyka D. L., Patel M., Quaker G. M., Sings H., Witkin S. L., Zhao A., Ujjainwalla F. (2012). Evaluation of Endo- and Exo-Aryl-Substitutions and
Central Scaffold Modifications on Diphenyl Substituted Alkanes as
5-Lipoxygenase Activating Protein Inhibitors. Bioorg. Med. Chem. Lett..

[16] He Z.-T., Hartwig J. F. (2019). Palladium-Catalyzed
α-Arylation for the Addition
of Small Rings to Aromatic Compounds. Nat. Commun..

[17] Sierov D. I., Dzhulai I. V., Siryk K. I., Shvydenko K. V., Shvydenko T. I., Nazarenko K., Kostyuk A., Liashuk O. S., Grygorenko O. O. (2023). Multigram
Synthesis of α- and γ-((Hetera)­Cyclo)­Alkylpyridines
through α-Arylation of (Hetero)­Aliphatic Nitriles. Eur. J. Org Chem..

[18] Croft R. A., Dubois M. A. J., Boddy A. J., Denis C., Lazaridou A., Voisin-Chiret A. S., Bureau R., Choi C., Mousseau J. J., Bull J. A. (2019). Catalytic Friedel-Crafts Reactions on Saturated Heterocycles
and Small Rings for sp^3^-sp^2^ Coupling of Medicinally
Relevant Fragments. Eur. J. Org Chem..

[19] Trost B. M., Xie J. (2006). Palladium-Catalyzed
Asymmetric Ring Expansion of Allenylcyclobutanols: An Asymmetric Wagner–Meerwein
Shift. J. Am. Chem. Soc..

[20] Wuitschik G., Rogers-Evans M., Müller K., Fischer H., Wagner B., Schuler F., Polonchuk L., Carreira E. M. (2006). Oxetanes as Promising
Modules in Drug Discovery. Angew. Chem., Int.
Ed..

[21] Croft R. A., Mousseau J. J., Choi C., Bull J. A. (2016). Structurally Divergent Lithium Catalyzed Friedel–Crafts
Reactions on Oxetan-3-ols: Synthesis of 3,3-Diaryloxetanes and 2,3-Dihydrobenzofurans. Chem.—Eur. J..

[22] Denis C., Dubois M. A. J., Voisin-Chiret A. S., Bureau R., Choi C., Mousseau J. J., Bull J. A. (2019). Synthesis
of 3,3-Diarylazetidines by Calcium­(II)-Catalyzed Friedel–Crafts
Reaction of Azetidinols with Unexpected Cbz Enhanced Reactivity. Org. Lett..

[23] Baumann A. N., Eisold M., Music A., Haas G., Kiw Y. M., Didier D. (2017). Methods for the Synthesis of Substituted Azetines. Org. Lett..

[24] Eisold M., Müller-Deku A., Reiners F., Didier D. (2018). Parallel Approaches
for the Functionalization of Thietes: α-Metalation versus C–H
Activation. Org. Lett..

[25] Saejong P., Zhong J., Rojas J. J., White A. J. P., Choi C., Bull J. A. (2024). Synthesis of 3,3-Disubstituted
Thietane Dioxides. J. Org. Chem..

[26] Alexander, R. P. ; Brace, G. N. ; Brown, J. A. ; Calmiano, M. D. ; Chovatia, P. T. ; Deligny, M. ; Gallimore, E. O. ; Heer, J. P. ; Jackson, V. E. ; Kroeplien, B. ; Coss, M. M. ; Quincey, J. R. ; Sabnis, Y. A. ; Swinnen, D. L. L. ; Zhu, Z. Fused tricyclic imidazole derivatives as modulators of tnf activity, WO 2015086526 A1, 2015.

[27] Fyfe, M. C. T. ; Teobald, B. J. Novel compounds, WO 2022090724 A1, 2022.

[28] Greenman, K. L. ; Pickrell, A. J. ; Li, K. ; Amegadzie, A. K. ; Wang, H.-L. ; Weires, N. A. ; Allen, J. G. ; Bourbeau, M. P. 5,6-fused and 6,6-fused bicyclic alcohols and ethers and compositions for use as 15-prostaglandin dehydrogenase modulators, WO 2024233550 A1, 2024.

[29] Cope S. M., Tailor D., Nagorski R. W. (2011). Determination of
the p*K*
_a_ of Cyclobutanone: Brønsted
Correlation of the General
Base-Catalyzed Enolization in Aqueous Solution and the Effect of Ring
Strain. J. Org. Chem..

[30] Chiodi D., Ishihara Y. (2025). Tertiary Alcohol: Reaping the Benefits but Minimizing
the Drawbacks of Hydroxy Groups in Drug Discovery. J. Med. Chem..

[31] Krasovskiy A., Kopp F., Knochel P. (2006). Soluble Lanthanide
Salts (LnCl_3_·2 LiCl) for the Improved Addition of
Organomagnesium Reagents to Carbonyl Compounds. Angew. Chem., Int. Ed..

[32] Imamoto T., Takiyama N., Nakamura K. (1985). Cerium Chloride-Promoted
Nucleophilic Addition of Grignard Reagents to Ketones an Efficient
Method for the Synthesis of Tertiary Alcohols. Tetrahedron Lett..

[33] Hatano M., Suzuki S., Ishihara K. (2010). Highly Chemoselective
Stoichiometric Alkylation of Ketones with Grignard Reagent Derived
Zinc­(II) Ate Complexes. Synlett.

[34] Initially, organometallic reagents were investigated with nonfluorinated cyclobutanone due to the high cost of difluorocyclobutanone at the time of investigation (£1000/g), which has now considerably reduced (£150/g). See Supporting Information for further details.

[35] Dryzhakov M., Richmond E., Moran J. (2016). Recent Advances in
Direct Catalytic Dehydrative Substitution of Alcohols. Synthesis.

[36] A preliminary reaction scope was examined at 40 °C through variation of the arene nucleophile. At 40 °C phenol, catechol, and 1,3-dimethoxybenzene gave the desired products (**34**, **35**, and **37**) in good yield with complete C4 regioselectivity. However, dialkylation of resorcinol was observed due to insolubility of the nucleophile in toluene. N-Methylindole was unsuccessful, giving only (85% recovery of starting material). Difluorocyclobutane derivatives **37** and **41** were fully stable under elevated temperatures, although no increase in yield was observed.

[37] Gwinn W. D. (1955). Information
Pertaining to Molecular
Structure, as Obtained from the Microwave Spectra of Molecules of
the Asymmetric Rotor Type. Discuss. Faraday
Soc..

[38] Rojas J. J., Croft R. A., Sterling A. J., Briggs E. L., Antermite D., Schmitt D. C., Blagojevic L., Haycock P., White A. J. P., Duarte F., Choi C., Mousseau J. J., Bull J. A. (2022). Amino-Oxetanes as Amide Isosteres
by an Alternative Defluorosulfonylative Coupling of Sulfonyl Fluorides. Nat. Chem..

[39] Dubois M. A. J., Smith M. A., White A. J. P., Lee Wei Jie A., Mousseau J. J., Choi C., Bull J. A. (2020). Short Synthesis
of Oxetane and Azetidine 3-Aryl-3-Carboxylic Acid Derivatives by Selective
Furan Oxidative Cleavage. Org. Lett..

[40] Kanada R., Kagoshima Y., Suzuki T., Nakamura A., Funami H., Watanabe J., Asano M., Takahashi M., Ubukata O., Suzuki K., Aikawa T., Sato K., Goto M., Setsu G., Ito K., Kihara K., Kuroha M., Kohno T., Ogiwara H., Isoyama T., Tominaga Y., Higuchi S., Naito H. (2023). Discovery of DS-9300:
A Highly Potent, Selective, and Once-Daily Oral EP300/CBP Histone
Acetyltransferase Inhibitor. J. Med. Chem..

[41] Zhong Z., Chesti J., Armstrong A., Bull J. A. (2022). Synthesis of Sulfoximine
Propargyl Carbamates under Improved Conditions for Rhodium Catalyzed
Carbamate Transfer to Sulfoxides. J. Org. Chem..

[42] Suga T., Shimazu S., Ukaji Y. (2018). Low-Valent
Titanium-Mediated Radical Conjugate Addition Using Benzyl Alcohols
as Benzyl Radical Sources. Org. Lett..

[43] Xie H., Guo J., Wang Y.-Q., Wang K., Guo P., Su P.-F., Wang X., Shu X.-Z. (2020). Radical Dehydroxylative Alkylation
of Tertiary Alcohols by Ti Catalysis. J. Am.
Chem. Soc..

[44] Ishikura H., Rojas J. J., Begg C. S., Choi C., Bull J. A. (2025). Synthesis
of *gem*-difluorocyclobutanes: Organolanthanum enabled
synthesis and divergent catalytic functionalization of *gem*-difluorocyclobutanols. ChemRxiv.

