# Supplementary material for: Synthesis of gem-Difluorocyclobutanes: Organolanthanum Enabled Synthesis and Divergent Catalytic Functionalization of gem-Difluorocyclobutanols
Source: J Org Chem. 2025 Jul 10;90(29):10425–33. doi: 10.1021/acs.joc.5c01175 (PMC12305666; doi:10.1021/acs.joc.5c01175)

SUPPORTING INFORMATION

Synthesis of *gem*-difluorocyclobutanes: Organolanthanum enabled synthesis and divergent catalytic functionalization of *gem*-difluorocyclobutanols

Hikaru Ishikura[a], Juan J. Rojas[a], Callum S. Begg[a], Chulho Choi[b] and James A. Bull[a,*]

[a] Department of Chemistry, Imperial College London, Molecular Sciences Research Hub, 82 Wood Lane W12 0BZ, UK
[b] Drug Design, Pfizer Research and Development, Groton, CT 06450, USA.

*E-mail: j.bull@imperial.ac.uk

General Experimental Considerations S2
Additional Structures in SI S3
Optimization of the Addition of Organometallics into Ketones S4
Optimization of Carbocation Generation and Trapping S8
Synthesis of Difluorocyclobutanols S9
Limitations in the Synthesis of Difluorocyclobutanols S16
Trapping of Difluorocyclobutane Carbocation S17
Limitations in the Generation and Trapping of Difluorocyclobutane Carbocation S30
Trapping of Difluorocyclobutane Radical S31
Further Functionalisation of Difluorocyclobutane Derivatives S32
Chemical Stability Tests S35
X-Ray Crystallography Details S37
References S40
^{1}H, ^{13}C{^{1}H} and ^{19}F NMR Spectra of Novel Compounds S41–S182

General Experimental Considerations

All reactions were run under an inert atmosphere (Ar) with flame-dried glassware, using standard techniques unless otherwise specified. Anhydrous solvents were purchased from Thermo Scientific Chemicals and used as supplied. Where stated, solvents were degassed by sparging with Ar for 30 min. Water for aqueous solutions and reaction quenches was deionized by electrodeionisation using an Arium® Advance EDI water purification system. Reactions in sealed tubes were run using Biotage® microwave vials (0.5–2 mL, 2–5 mL, 10–20 mL) and aluminum caps with molded butyl/PTFE septa (1,4-dioxane, PhMe, THF) or simple butyl septa (acetone).

Liquid commercial amines were distilled over KOH pellets before use. Anhydrous K_2CO_3 (≥98%, powder, 325 mesh) was purchased from Sigma-Aldrich and flame-dried before use. All other inorganic bases were oven-dried before use. The exact concentration of *n*-BuLi (1.6 M in hexanes, purchased from Sigma-Aldrich), *s*-BuLi (1.4 M in cyclohexane, purchased from Sigma-Aldrich), *t*-BuLi (1.7 M in pentane, purchased from Sigma-Aldrich), *p*-methoxyphenylmagnesium bromide (0.5 M in THF, purchased from Sigma-Aldrich), and *p*-chlorophenylmagnesium bromide (1.0 M in Et_2O or 2-MeTHF, purchased from Sigma-Aldrich) was determined by titration with salicylaldehyde phenylhydrazone as an indicator before each reaction.[1] The average of three titrations was taken. $LaCl_3$•2LiCl (0.6 M in THF, purchased from Sigma-Aldrich, 703559-25ML) was stored in a desiccator and consumed within one month of opening without titration. Liquid alkenes were distilled in the dark under vacuum and stored in an amber vial, under argon, and in the dark at –20 °C. All other commercial reagents were used as supplied or purified by standard techniques where necessary. Room temperature indicates the reaction vessel was not placed in a water or oil bath but instead left to react at ambient temperature (~20–22 °C). All other indications of temperature were measured using a PT 1000.60 temperature sensor (≥25 °C) or liquid-in-glass partial immersion thermometer (≤0 °C).

Manual flash column chromatography was performed using 230–400 mesh silica, with the indicated solvent system according to standard techniques. Analytical thin-layer chromatography (TLC) was performed on precoated glass-backed silica gel plates with F254 fluorescent indicator. Visualization of the developed chromatogram was performed by UV absorbance (254 nm) and staining with aqueous potassium permanganate solution, aqueous cerium molybdate solution, or *p*-anisaldehyde in ethanol. Infrared spectra (ν_{max}, FTIR-ATR) were recorded in reciprocal centimeters (cm^{-1}) using an Agilent Cary 630 FTIR spectrometer. Nuclear magnetic resonance spectra were recorded on either 400 or 500 MHz Bruker AvIII HD spectrometers with a SampleXpress automatic sample changer. Chemical shifts for ^{1}H NMR spectra were recorded in parts per million (ppm) from tetramethylsilane with the residual protonated solvent resonance as the internal standard ($CDCl_3$: δ 7.27 ppm, acetone-*d6*: δ 2.05 ppm, DMSO-*d6*: δ 2.50 ppm). Data was reported as follows: chemical shift (multiplicity [s = singlet, d = doublet, t = triplet, q = quartet, m = multiplet, app. = apparent, and br = broad], coupling constant (in Hz), integration, and assignment). All multiplet signals were quoted over a chemical shift range. ^{13}C NMR spectra were recorded with complete proton decoupling. Chemical shifts were reported in parts per million from tetramethylsilane with the solvent resonance as the internal standard ($^{13}CDCl_3$: δ 77.0 ppm, acetone-*d6*: δ 29.8, 206.3 ppm, DMSO-*d6*: δ 39.5 ppm). The assignments of the ^{1}H and ^{13}C spectra for compound **24** were unambiguously assigned using HSQC and experiments. The assignments of all other ^{1}H and ^{13}C spectra were based upon the analogous analysis of δ and *J* values, as well as HSQC and HMBC experiments where necessary. ^{19}F NMR spectra were recorded with complete proton decoupling. Chemical shifts are reported in parts per million. ^{19}F NMR spectra are indirectly referenced to $CFCl_3$ automatically by direct measurement of the absolute frequency of the deuterium lock signal by the spectrometer hardware. Melting points were obtained using a Stuart SMP10 digital melting point apparatus and are uncorrected.

High-resolution mass spectra (HRMS) were obtained through the Imperial College London mass spectrometry service. HRMS analyses were performed using an electrospray ion source (ESI) or atmospheric pressure chemical ionization (APCI) using an atmospheric solids analysis probe (ASAP). ESI was performed using a Waters LCT Premier (ES-TOF) equipped with an ESI source operated in positive ion mode or a Thermo Scientific Q-Extractive/Dionex Ultimate 3000. APCI was performed using a Thermo Scientific Q-Extractive/Dionex Ultimate 3000 using an ASAP to insert samples into the APCI source operated in positive or negative mode. The sample was introduced at ambient temperature and the temperature increased until the sample vaporized.

Additional Structures in SI

Compounds **S1** and **S2**,[2] **S3**,[3] **S4**,[4] **S5**,[5] **S6**,[6] **S12**,[7] and **S13**[8] were synthesized according to literature procedures.

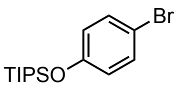

S1

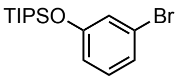

S2

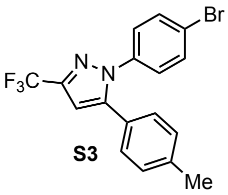

S3

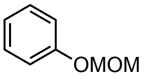

S4

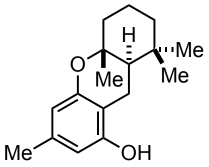

S5

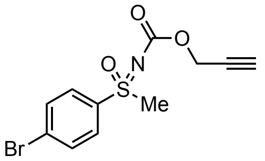

S6

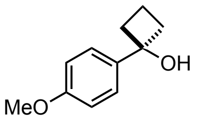

S7

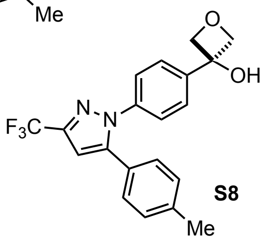

S8

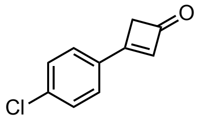

S9

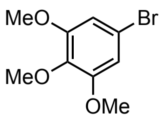

S10

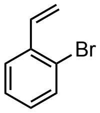

S11

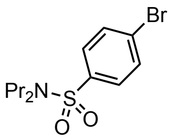

S12

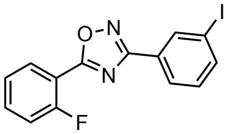

S13

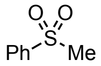

S14

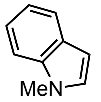

S15

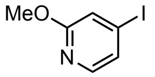

S16

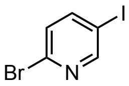

S17

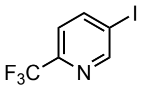

S18

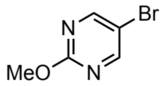

S19

Figure S1: Structures of additional compounds in SI.

Optimization of the Addition of Organometallics into Ketones

We began the optimization of conditions for the addition of organometallic reagents into difluorocyclobutanone by first screening a series of conditions against cyclobutanone (Table S1). Transmetalation of Grignard reagents (MgBr, entry 4) and organolithium reagents (*n*-BuLi, entry 9) with a commercial solution of $LaCl_3$•2LiCl before dropwise addition of cyclobutanone gave the best results.

Table S1: Optimization of the addition of organometallic reagents to cyclobutanone.

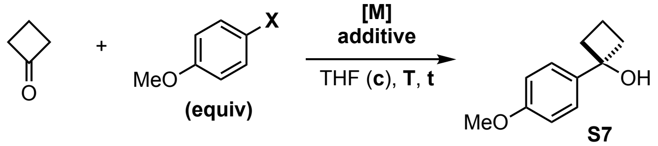

Entry[a]	X	[M]	equiv	additive (equiv)	T / °C	t /h	c / M	Yield (%)[b]
1	-	MgBr[c]	2.0	-	0	2	0.1	75 (73)
2	Br	*n*-BuLi	1.2	-	–78 to rt	3	0.2	88
3	I	*i*PrMgCl•LiCl	2.2	-	0 to rt to 0 to rt	27	0.5	48
3[d]	-	MgBr[c]	2.0	$LaCl_3$•2LiCl (2.0)	0	2	0.1	39
**4[e]**	**-**	**MgBr[c]**	**2.0**	**$LaCl_3$•2LiCl (2.0)**	**0 to rt to 0**	**6**	**0.1**	**89**
5	-	MgBr[c]	1.5	$CeCl_3$ (1.5)	rt to 0	4	0.2	56
6	-	MgBr[c]	3.3	$ZnCl_2$ + LiCl (1.1)	rt to 0	3	0.5	13
7	-	MgBr[c]	1.5	$ZnCl_2$ + LiCl (1.5) TMSCH_2MgCl (3.0)	rt to –78 to 0	5	0.05	0
8	Br	*n*-BuLi	1.3	$LaCl_3$•2LiCl (1.3)	–78 to 0	3.5	0.2	17
**9**	**Br**	***n*-BuLi**	**2.2**	**$LaCl_3$•2LiCl (2.2)**	**–78 to 0**	**3.5**	**0.2**	**89**
10	I	*i*PrMgCl•LiCl	1.3	$LaCl_3$•2LiCl (1.3)	0 to rt to 0 to rt to 0	9	0.5	0
11	I	*i*PrMgCl•LiCl	2.2	$LaCl_3$•2LiCl (2.2)	0 to rt to 0 to rt to 0	27	0.5	0

[a]On a 0.47 mmol scale. [b]Yield determined by ^{1}H NMR spectroscopy using 1,3,5-trimethoxybenzene as an internal standard. Isolated yield in parentheses. [c]Using a commercial solution of *p*-methoxyphenylmagnesium bromide (0.48 M in THF). [d]*p*-Methoxyphenylmagnesium bromide was added to a solution of cyclobutanone and $LaCl_3$•2LiCl. [e]$LaCl_3$•2LiCl was added to a solution of *p*-methoxyphenylmagnesium bromide and stirred for 4 h at rt, before addition of cyclobutanone.

Although the improvements in yield were minimal for already high-yielding substrates, such as *p*-methoxyphenyl (PMP), significant improvements in yield were observed for more difficult substrates, such as aryl bromide **S3** (Table S2). The use of *t*-BuLi also resulted in improved yields, likely due to improved selectivity in the site of addition in comparison to *n*-BuLi.

Table S2: Optimization of the addition of aryl bromide **S3** to oxetanone.

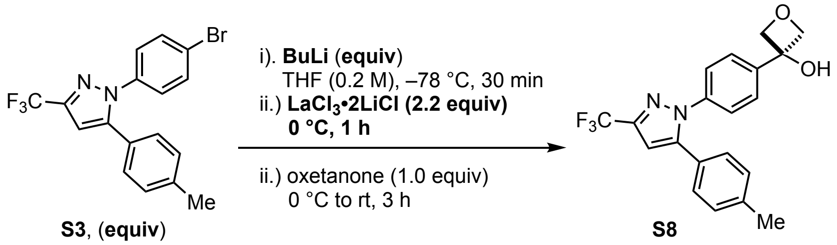

Entry[a]	equiv S3	BuLi	equiv BuLi	additive	Yield (%)[b]
1	1.2	*n*-BuLi	1.1	-	60
2	1.2	*t*-BuLi	2.4	-	70
3	2.2	*t*-BuLi	4.4	-	76
4	2.2	*n*-BuLi	2.0	$LaCl_3$•2LiCl (2.2)	72 (74)
**5**	**2.2**	***t*-BuLi**	**4.4**	**$LaCl_3$•2LiCl (2.2)**	**89 (88)**

[a]On a 0.5 mmol scale. [b]Yield determined by ^{1}H NMR spectroscopy using 1,3,5-trimethoxybenzene as an internal standard. Isolated yield in parentheses.

The ideal conditions from Table S1 were tested with difluorocyclobutanone (Table S3). The best results were obtained by transmetalation of Grignard reagents (MgBr, entry 1) with $LaCl_3$•2LiCl, followed closely by transmetalation of organolithium reagents (*n*-BuLi, entry 6) with $LaCl_3$•2LiCl before addition of difluorocyclobutanone as a solution in THF.

Table S3: Optimization of the addition of organometallic reagents to difluorocyclobutanone.

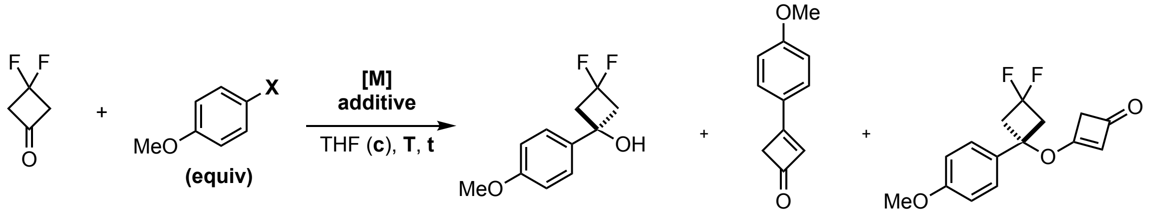

1 2 3

Entry[a]	X	[M]	equiv	additive (equiv)	T / °C	t / h	c / M	Yield (%)[b] 1	2	3
1	-	MgBr[c]	1.1	-	–78 to rt	0.5	0.5	(12)	(16)	(2)
2	-	MgBr[c]	1.1	-	–78	0.25	0.5	(14)	(15)	(2)
3	Br	*n*-BuLi	1.1	-	–78 to rt	1	0.22	(6)	0	0
4	-	MgBr[c]	2.0	$LaCl_3$•2LiCl (2.0)	0 to rt to 0	6	0.1	(82)	0	0
5	-	MgBr[c]	2.0	$LaCl_3$•2LiCl (2.0) + LiCl (2.0)	0 to rt to 0	6	0.1	80 (80)	0	0
6	-	MgBr[c]	1.5	$CeCl_3$ (1.5)	rt to 0	4	0.2	25	7	3
7	Br	*n*-BuLi	2.2	$LaCl_3$•2LiCl (2.2)	–78 to 0	3.5	0.2	(79)	0	0
8	I	*i*PrMgCl•LiCl	2.2	$LaCl_3$•2LiCl (2.2)	0 to rt to 0 to rt to 0	9	0.5	0	0	0

[a]On a 0.47 mmol scale. [b]Yield determined by ^1H NMR spectroscopy using 1,3,5-trimethoxybenzene as an internal standard. Isolated yield in parentheses. [c]Using a commercial solution of *p*-methoxyphenylmagnesium bromide (0.48 M in THF).

Particular difficulties were encountered in the transmetalation of substrates with halides, such as *p*-Cl difluorocyclobutanol **11**. A series of conditions were screened, however, the initial conditions proved most suitable (Table S4). Significant formation of elimination-related side product **S9** was observed in all cases; however, proved separable by flash column chromatography.

Table S4: Optimization of the addition of *p*-chlorophenyl organometallics to difluorocyclobutanone.

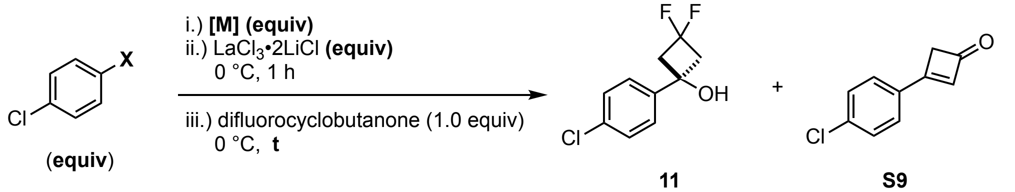

Entry[a]	X	equiv	[M]	equiv	equiv of LaCl3•2LiCl	t / h	Yield (%)[b] 11	Yield (%)[b] S9
1	Br	2.2	Li	2.2	2.2	2	42	35
2	Br	2.2	Li	2.2	2.2	4	38	30
3	I	2.2	Li	2.2	2.2	2	<5	10
4	-	-	MgBr[c]	2.0	2.0	2	<5	28
8	-	-	MgBr[d]	2.0	2.0	2	25	0

[a]On a 0.5 mmol scale. [b]Yield determined by ^{1}H NMR spectroscopy using 1,3,5-trimethoxybenzene as an internal standard. Isolated yield in parentheses. [c]Using a commercial solution of *p*-chlorophenylmagnesium bromide (0.99 M in Et_2O). [d]Using a commercial solution of *p*-chlorophenylmagnesium bromide (0.98 M in 2-MeTHF).

Optimization of Carbocation Generation and Trapping

The Friedel-Crafts reaction proceeded near quantitatively under all tested conditions. $FeCl_3$ (10 mol%) in PhMe (entry 6) were chosen for the final conditions due the favorable reaction profile and to minimize cost and environmental impact. Temperatures could also be increased to 110 °C for difficult substrates or when solubility issues are encountered (entry 7).

Table S5: Optimization of the Friedel-Crafts reaction of PMP difluorocyclobutanol **1** with *o*-cresol.

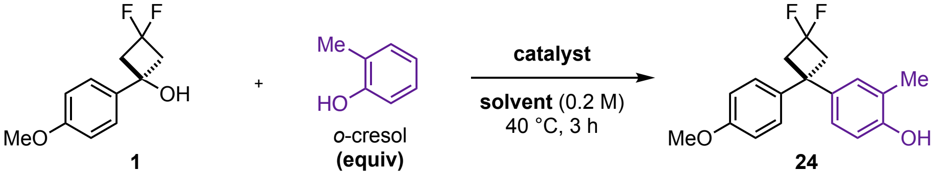

Entry[a]	catalyst	solvent	equiv	Yield (%)[b]
1	$Ca(NTf_2)_2$ (5 mol%) / NBu_4PF_6 (5 mol%)	CH_2Cl_2	5.0	97
2	Li(NTf2) (11 mol%) / NBu_4PF_6 (5.5 mol%)	CH_2Cl_2	5.0	97
3	$FeCl_3$ (10 mol%)	CH_2Cl_2	5.0	100 (96)
4	$HNTf_2$ (10 mol%)	CH_2Cl_2	5.0	95
5	$FeCl_3$ (10 mol%)	PhMe	5.0	96
**6**	**$FeCl_3$ (10 mol%)**	**PhMe**	**3.0**	**95 (91)**
**7[c]**	**$FeCl_3$ (10 mol%)**	**PhMe**	**3.0**	**96 (92)**

[a]On a 47 µmol scale. [b]Yield determined by ^1H NMR spectroscopy using 1,3,5-trimethoxybenzene as an internal standard. Isolated yield in parentheses. [c]at 110 °C.

Synthesis of Difluorocyclobutanols

General Procedure A

n-BuLi (1.59 M in hexanes, 0.65 mL, 1.0 mmol, 2.0 equiv) was added dropwise to a solution of aryl bromide/iodide (1.1 mmol, 2.2 equiv) in anhydrous THF (2.5 mL, 0.2 M) at –78 °C. After stirring for 30 min, the reaction mixture was warmed to 0 °C and $LaCl_3$•2LiCl (0.6 M in THF, 1.83 mL, 1.1 mmol, 2.2 equiv) was added dropwise to the reaction mixture. After a further 1 h of stirring, a solution of 3,3-difluorocyclobutanone (53 mg, 0.5 mmol, 1.0 equiv) in anhydrous THF (0.65 mL) was added dropwise. The 3,3-difluorocyclobutanone vial was rinsed with anhydrous THF (0.65 mL) which was also added dropwise to the reaction mixture to ensure complete transfer. After stirring for 2 h, the reaction mixture was quenched with sat. aq. NH_4Cl (15 mL) and diluted with Et_2O (30 mL). The layers were separated, and the aqueous layer was extracted with Et_2O/pentane (2:1, 30 mL). The combined organic layers were washed with brine, dried over anhydrous Na_2SO_4, filtered, and concentrated *in vacuo*. Purification by flash column chromatography under the stated conditions afforded the difluorocyclobutanol.

General Procedure B

n-BuLi (1.59 M in hexanes, 0.65 mL, 1.0 mmol, 2.0 equiv) was added dropwise to a solution of heteroaryl bromide/iodide (1.1 mmol, 2.2 equiv) in anhydrous THF (2.5 mL, 0.2 M) at –78 °C. After stirring for 30 min, $LaCl_3$•2LiCl (0.6 M in THF, 1.83 mL, 1.1 mmol, 2.2 equiv) was added dropwise to the reaction mixture. After a further 1.5 h of stirring at –78 °C, the reaction mixture was warmed to 0 °C. After stirring for 30 min, a solution of 3,3-difluorocyclobutanone (53 mg, 0.5 mmol, 1.0 equiv) in THF (0.65 mL) was added dropwise. The 3,3-difluorocyclobutanone vial was rinsed with anhydrous THF (0.65 mL) which was also added dropwise to the reaction mixture to ensure complete transfer. After stirring for 2 h, the reaction mixture was quenched with sat. aq. NH_4Cl (15 mL) and diluted with Et_2O (30 mL). The layers were separated, and the aqueous layer was extracted with Et_2O/pentane (2:1, 30 mL). The combined organic layers were washed with brine, dried over anhydrous Na_2SO_4, filtered, and concentrated *in vacuo*. Purification by flash column chromatography under the stated conditions afforded the difluorocyclobutanol.

3,3-Difluoro-1-(4-methoxyphenyl)cyclobutan-1-ol (1)

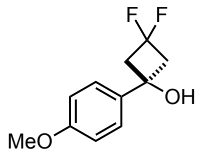

Prepared according to General Procedure A using 4-bromoanisole (0.14 mL, 1.1 mmol, 2.2 equiv). Purification by flash column chromatography (20–30% Et_2O/pentane) afforded difluorocyclobutanol **1** as a pale-yellow oil (84.8 mg, 79%). R_f = 0.35 (40% Et_2O/pentane); IR (film)/cm^{-1} 3413 (OH, br), 2959, 1611, 1514, 1295, 1250, 1177, 1031, 837; ^{1}H NMR (400 MHz, $CDCl_3$) δ 7.40 (d, *J* = 8.7 Hz, 2H, 2 × Ar-CH), 6.95 (d, *J* = 8.7 Hz, 2H, 2 × Ar-CH), 3.85 (s, 3H, OCH_3), 3.25–3.10 (m, 2H, 2 × CH*H*), 3.09–2.94 (m, 2H, 2 × C*H*H), 2.12 (s, 1H, OH); ^{13}C{^{1}H} NMR (101 MHz, $CDCl_3$) δ 159.3 (Ar-*C*$_q$OMe), 136.3 (dd, *J* = 5.7, 2.6 Hz, Ar-*C*$_q$C$_q$), 126.4 (2 × Ar-CH), 118.2 (dd, *J* = 276.0, 276.0 Hz, CF_2), 114.1 (2 × Ar-CH), 67.5 (dd, *J* = 14.8, 12.4 Hz, C_q), 55.4 (OCH_3), 49.9 (dd, *J* = 22.6, 22.6 Hz, *C*H_2CF_2*C*H_2); ^{19}F NMR (377 MHz, $CDCl_3$) δ –90.42 (d, *J* = 199.0 Hz), –92.77 (d, *J* = 199.0 Hz); HRMS (FTMS-APCI$^+$) *m/z* calcd for $C_{11}H_{11}F_2O^+$ $[M–OH]^+$: 197.0772; found 197.0773.

*N.B. PMP difluorocyclobutanol (**1**) was also synthesized on a 2.0 mmol scale, affording difluorocyclobutanol **1** as a pale-yellow oil (331 mg, 77% yield).*

3,3-Difluoro-1-(4-methoxyphenyl)cyclobutan-1-ol (1), 3-(4-methoxyphenyl)cyclobut-2-en-1-one (2), and 3-(3,3-difluoro-1-(4-methoxyphenyl)cyclobutoxy)cyclobut-2-en-1-one (3)

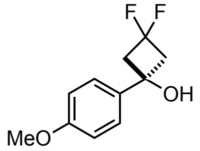

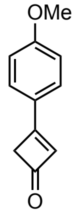

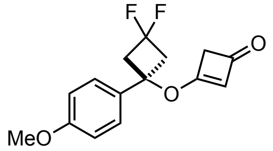

p-Methoxyphenylmagnesium bromide (0.47 M in THF, 2.20 mL, 1.04 mmol, 1.1 equiv) was added dropwise to a solution of 3,3-difluorocyclobutanone (100 mg, 0.94 mmol, 1.0 equiv) in anhydrous THF (2.0 mL, 0.5 M) at –78 °C. After stirring for 20 min at –78 °C, the reaction mixture was warmed to rt and quenched with sat. aq. NH_4Cl (10 mL) and diluted with Et_2O (10 mL). The layers were separated, and the aqueous layer was extracted with Et_2O (3 × 10 mL). The combined

organic layers were washed with brine, dried over anhydrous Na_2SO_4, filtered, and concentrated *in vacuo*. Purification by flash column chromatography (20–50% Et_2O/pentane) afforded difluorocyclobutanol **1** as a pale-yellow oil (24.5 mg, 12%), followed by cyclobutenone **2** as a brown gum (25.5 mg, 16%), and difluorocyclobutane ether **3** as a pale-yellow gum (6.4 mg, 2%).

Cyclobutenone **2:** R_f = 0.24 (40% Et_2O/pentane); IR (film)/cm^{-1} 22947, 1739 (C=O), 1600, 1498, 1258, 1174, 1019, 825; ^{1}H NMR (400 MHz, $CDCl_3$) δ 7.59–7.56 (m, 2H, 2 × Ar-CH), 7.02–6.98 (m, 2H, 2 × Ar-CH), 6.23 (s, 1H, C=CH), 3.89 (s, 3H, OCH_3), 3.49 (s, 2H, CH_2); $^{13}C\{^1H\}$ NMR (101 MHz, $CDCl_3$) δ 187.3 (C=O), 170.5 (C_q=CH), 162.6 (Ar-C_qOMe), 131.0 (2 × Ar-CH), 127.1 (C_q=*C*H), 124.4 (Ar-C_qC_q) 114.4 (2 × Ar-CH), 55.5 (OCH_3), 48.4 (CH_2). The observed characterization data was consistent with that previously reported.[9]

Difluorocyclobutane ether **3**: R_f = 0.16 (40% Et_2O/pentane); IR (film)/cm^{-1} 2933, 2861, 1754 (C=O), 1566, 1515, 1303, 1252, 1176, 1108, 994, 836; ^{1}H NMR (400 MHz, $CDCl_3$) δ 7.33–7.29 (m, 2H, 2 × Ar-CH), 6.96–6.92 (m, 2H, 2 × Ar-CH), 4.45 (s, 1H, C=CH), 3.83 (s, 3H, OCH_3), 3.39–3.23 (m, 4H, $CH_2C_qCH_2$), 3.19 (s, 2H, CH_2); $^{13}C\{^1H\}$ NMR (101 MHz, $CDCl_3$) δ 183.6 (C=O), 177.2 (C_q=CH), 160.1 (Ar-C_qOMe), 129.8 (Ar-C_qC_q), 127.1 (2 × Ar-CH), 118.2 (app. d, *J* = 274.3 Hz, CF_2), 114.4 (2 × Ar-CH), 110.8 (C_q=*C*H), 78.3 (app. d, *J* = 14.2, Hz, C_q), 55.4 (OCH_3), 49.3 (*C*H_2C_q=O), 47.5 (dd, *J* = 24.2, 24.2 Hz, *C*H_2CF_2*C*H_2); ^{19}F NMR (377 MHz, $CDCl_3$) δ –89.9 (d, *J* = 201.3 Hz), –92.7 (d, *J* = 201.3 Hz); HRMS (FTMS-APCI$^-$) *m/z* calcd for $C_{15}H_{13}O_3F_2^-$ [M–H]$^-$: 279.0833; found 279.0832.

3,3-Difluoro-1-(4-((triisopropylsilyl)oxy)phenyl)cyclobutan-1-ol (4)

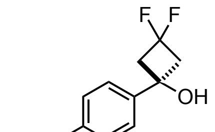

Prepared according to General Procedure A using (4-bromophenoxy)triisopropylsilane **S1** (362 mg, 1.1 mmol, 2.2 equiv). Purification by flash column chromatography (10–20% Et_2O/pentane) afforded difluorocyclobutanol **4** as a yellow oil (145 mg, 82%). R_f = 0.18 (20% Et_2O/pentane); IR (film)/cm^{-1} 3381 (OH, br), 2944, 2866, 1606, 1511, 1463, 1293, 1267, 1172, 1103, 1012, 995, 911, 881, 745, 682; ^{1}H NMR (400 MHz, $CDCl_3$) δ 7.31 (d, *J* = 8.7 Hz, 2H, 2 × Ar-CH), 6.91 (d, *J* = 8.7 Hz, 2H, 2 × Ar-CH), 3.21–3.08 (m, 2H, 2 × CH*H*), 3.07–2.92 (m, 2H, 2 × *C*HH), 2.19 (s, 1H, OH), 1.35–1.20 (m, 3H, 3 × CH), 1.13 (d, *J* = 7.3 Hz, 18H, 6 × CH_3); $^{13}C\{^1H\}$ NMR (101 MHz, $CDCl_3$) δ 155.9 (Ar-C_qOTIPS), 136.6 (dd, *J* = 5.2, 3.1 Hz, Ar-C_qC_q), 126.2 (2 × Ar-CH), 120.0 (2 × Ar-CH), 118.2 (dd, *J* = 276.6, 276.6 Hz, CF_2), 67.5 (dd, *J* = 15.2, 11.9 Hz, C_q), 49.9 (dd, *J* = 22.6, 22.6 Hz, *C*H_2CF_2*C*H_2), 17.9 (6 × CH_3), 12.7 (3 × CH); ^{19}F NMR (377 MHz, $CDCl_3$) δ –90.61 (d, *J* = 197.1 Hz),
–92.45 (d, *J* = 197.1 Hz); HRMS (TOF-MS-ES$^+$) *m/z* calcd for $C_{19}H_{29}OF_2Si^-$ [M–OH]$^-$: 339.1950; found 339.1948.

*N.B. p-OTIPS difluorocyclobutanol (**4**) was also synthesized on a 2.0 mmol scale in 82% yield.*

4-(3,3-Difluoro-1-hydroxycyclobutyl)phenol (9)

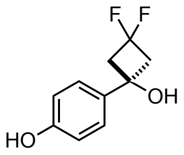

TBAF (1.0 M in THF, 0.24 mL, 0.24 mmol, 1.2 equiv) was added dropwise to a solution of *p*-OTIPS difluorocyclobutanol **4** (71.3 mg, 0.2 mmol, 1.0 equiv) in THF (1.0 mL, 0.2 M) at 0 °C. The cold bath was removed and after stirring for 1.5 h, the reaction mixture was quenched with sat. aq. NH_4Cl (10 mL). The aqueous layer was extracted with EtOAc (3 × 10 mL). The combined organic layers were dried over anhydrous Na_2SO_4, filtered, and concentrated *in vacuo*. Purification by flash column chromatography (50% Et_2O/pentane) afforded difluorocyclobutanol **9** as a white solid (40.4 mg, quant.). R_f = 0.15 (50% Et_2O/pentane); mp = 127–129 °C; IR (film)/cm^{-1} 3304 (OH, br), 1612, 1515, 1440, 1401, 1362, 1295, 11228, 1170, 1126, 1105, 1015, 989, 834, 616, 501; ^{1}H NMR (400 MHz, acetone-*d$_6$*) δ 8.35 (s, 1H, OH), 7.41–7.30 (m, 2H, 2 × Ar-CH), 6.90–6.79 (m, 2H, 2 × Ar-CH), 4.87 (s, 1H, OH), 3.18–3.00 (m, 2H, 2 × CH*H*), 3.00–2.85 (m, 2H, 2 × *C*HH); $^{13}C\{^1H\}$ NMR (101 MHz, acetone-*d$_6$*) δ 156.6 (Ar-C_qOH), 136.6 (dd, *J* = 6.3, 3.0 Hz, Ar-C_qC_q); 126.5 (2 × Ar-CH), 119.0 (dd, *J* = 273.5, 273.5 Hz, CF_2), 114.9 (2 × Ar-CH), 66.0 (dd, *J* = 12.8, 12.2 Hz, C_q), 50.0 (dd, *J* = 22.1, 22.1 Hz, *C*H_2CF_2*C*H_2); ^{19}F NMR (377 MHz, acetone-*d$_6$*) δ –89.88 (d, *J* = 196.9 Hz), –92.46 (d, *J* = 196.9 Hz); HRMS (FTMS-ESI$^-$) *m/z* calcd for $C_{10}H_9F_2O_2^-$ [M–H]$^-$: 199.0576; found 199.0571.

3,3-Difluoro-1-(4-vinylphenyl)cyclobutan-1-ol (5)

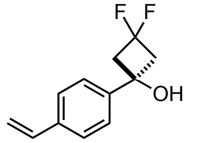

Prepared according to General Procedure A using 4-bromostyrene (0.14 mL, 1.1 mmol, 2.2 equiv). Purification by flash column chromatography (25% Et_2O/pentane) afforded difluorocyclobutanol **5** as a yellow oil (41.0 mg, 40%). R_f = 0.32 (30% Et_2O/pentane); IR (film)/cm^{-1} 3399 (OH, br), 3088, 3009, 2957, 1630 (C=C), 1513, 1391, 1295, 1231, 1187, 1109, 989, 911, 846; ^{1}H NMR (400 MHz, $CDCl_3$) δ 7.54–7.36 (m, 4H, 4 × Ar-CH), 6.75 (dd, *J* = 17.6, 10.9 Hz, 1H, C*H*ArC$_q$), 5.81 (d, *J* = 17.6 Hz, 1H, C*H*H), 5.32 (d, *J* = 10.9 Hz, 1H, CH*H*), 3.25–3.09 (m, 2H, 2 × CH*H*), 3.06–2.92 (m, 2H, 2 × C*H*H), 2.44 (s, 1H, OH); ^{13}C{^{1}H} NMR (101 MHz, $CDCl_3$) δ 143.5 (dd, *J* = 5.7, 2.8 Hz, Ar-*C$_q$*C$_q$), 137.4 (Ar-*C$_q$*CH), 136.1 (Ar-C$_q$*C*H), 126.6 (2 × Ar-CH), 125.2 (2 × Ar-CH), 118.1 (dd, *J* = 276.4 Hz, CF_2), 114.6 (*C*H$_2$CH), 67.6 (dd, *J* = 15.2, 11.9 Hz, C$_q$), 50.1 (dd, *J* = 22.8, 22.8 Hz, *C*H$_2$CF$_2$*C*H$_2$); ^{19}F NMR (377 MHz, $CDCl_3$) δ –90.23 (d, *J* = 199.1 Hz), –92.43 (d, *J* = 199.1 Hz); HRMS (FTMS-ESI$^+$) *m/z* calcd for $C_{12}H_{11}F_2^+$ [M–OH]$^+$: 193.0823; found 193.0824.

3,3-Difluoro-1-(3-((triisopropylsilyl)oxy)phenyl)cyclobutan-1-ol (6)

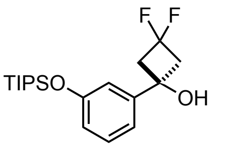

Prepared according to General Procedure A using (3-bromophenoxy)triisopropylsilane **S2** (362 mg, 1.1 mmol, 2.2 equiv). Purification by flash column chromatography (10–20% Et_2O/pentane) afforded difluorocyclobutanol **6** as a yellow oil (159 mg, 89%). R_f = 0.31 (20% Et_2O/pentane); IR (film)/cm^{-1} 3393 (OH, br), 2944, 2868, 1602, 1582, 1431, 1292, 1189, 1170, 1015, 952, 882, 785, 752, 682, 661; ^{1}H NMR (400 MHz, $CDCl_3$) δ 7.27 (t, *J* = 7.7 Hz, 1H, Ar-CH), 7.09–6.96 (m, 2H, 2 × Ar-CH), 6.86 (ddd, *J* = 8.1, 2.4, 1.0 Hz, 1H, Ar-CH), 3.21–3.07 (m, 2H, 2 × CH*H*), 3.07–2.93 (m, 2H, 2 × C*H*H), 2.30–2.22 (m, 1H, OH), 1.30 (ddd, *J* = 14.7, 8.4, 6.7 Hz, 3H, 3 × CH), 1.14 (d, *J* = 7.4 Hz, 18H, 6 × CH_3); ^{13}C{^{1}H} NMR (101 MHz, $CDCl_3$) δ 156.5 (Ar-*C$_q$*OTIPS), 145.8 (dd, *J* = 5.9, 2.5 Hz, Ar-*C$_q$*C$_q$), 129.7 (Ar-CH), 119.3 (Ar-CH), 118.1 (dd, *J* = 273.7, 273.7 Hz, CF_2), 117.3 (Ar-CH), 116.7 (Ar-CH), 67.5 (dd, *J* = 15.0, 12.0 Hz, C$_q$), 50.1 (dd, *J* = 22.8, 22.8 Hz, *C*H$_2$CF$_2$*C*H$_2$), 17.9 (6 × CH_3), 12.7 (3 × CH); ^{19}F NMR (377 MHz, $CDCl_3$) δ –90.31 (d, *J* = 198.9 Hz), –92.66 (d, *J* = 198.9 Hz); HRMS (FTMS-ESI$^-$) *m/z* calcd for $C_{19}H_{29}F_2O_2Si^-$ [M–H]$^-$: 355.1910; found 355.1910.

3-(3,3-Difluoro-1-hydroxycyclobutyl)phenol (10)

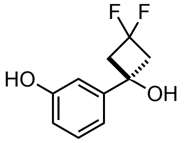

TBAF (1.0 M in THF, 0.24 mL, 0.24 mmol, 1.2 equiv) was added dropwise to a solution of *m*-OTIPS difluorocyclobutanol **6** (71.3 mg, 0.2 mmol, 1.0 equiv) in THF (1.0 mL, 0.2 M) at 0 °C. The cold bath was removed and after stirring for 1.5 h, the reaction mixture was cooled to 0 °C and further TBAF (1.0 M in THF, 0.16 mL, 0.16 mmol, 0.8 equiv) was added dropwise. The cold bath was removed and after stirring for 1.5 h, the reaction mixture was quenched with sat. aq. NH_4Cl (10 mL). The aqueous layer was extracted with EtOAc (3 × 10 mL). The combined organic layers were dried over anhydrous Na_2SO_4, filtered, and concentrated *in vacuo*. Purification by flash column chromatography (50% Et_2O/pentane) afforded difluorocyclobutanol **10** as a white solid (36.1 mg, 90%). R_f = 0.16 (50% Et_2O/pentane); mp = 93–94 °C; IR (film)/cm^{-1} 3360 (OH, br), 1589, 1451, 1293, 1185, 1120, 1019, 900, 868, 784, 698; ^{1}H NMR (400 MHz, acetone-*d$_6$*) δ 8.36 (s, 1H, OH), 7.21 (t, *J* = 7.8 Hz, 1H, Ar-CH), 7.06–6.96 (m, 2H, 2 × Ar-CH), 6.77 (dd, *J* = 8.1, 2.4 Hz, 1H, Ar-CH), 4.98 (s, 1H, OH), 3.18–3.01 (m, 2H, 2 × CH*H*), 3.01–2.85 (m, 2H, 2 × C*H*H); ^{13}C{^{1}H} NMR (101 MHz, acetone-*d$_6$*) δ 157.4 (Ar-*C$_q$*OH), 147.5 (dd, *J* = 6.3, 3.1 Hz, Ar-*C$_q$*C$_q$), 129.3 (Ar-CH), 119.0 (dd, *J* = 277.1, 273.5 Hz, CF_2), 116.1 (Ar-CH), 114.1 (Ar-CH), 112.2 (Ar-CH), 66.0 (dd, *J* = 15.2, 12.3 Hz, C$_q$), 50.2 (dd, *J*= 22.3, 22.3 Hz, *C*H$_2$CF$_2$*C*H$_2$); ^{19}F NMR (377 MHz, acetone-*d$_6$*) δ –89.99 (d, *J* = 197.1 Hz), –92.27 (d, *J* = 197.1 Hz); HRMS (FTMS-ESI$^-$) *m/z* calcd for $C_{10}H_9F_2O_2^-$ [M–H]$^-$: 199.0576; found 199.0575.

1-(Dibenzo[*b*,*d*]thiophen-2-yl)-3,3-difluorocyclobutan-1-ol (7)

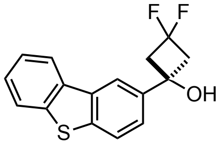

Prepared according to General Procedure A using 2-bromodibenzothiophene (289 mg, 1.1 mmol, 2.2 equiv). Purification by flash column chromatography (20% Et_2O/pentane, followed by 0–5% Et_2O/PhMe) afforded difluorocyclobutanol **7** as a white solid (98.7 mg, 68%). R_f = 0.19 (PhMe); mp = 93–95 °C; IR (film)/cm^{-1} 3386 (OH, br), 3058, 3013, 2953, 1433, 1414, 1291, 1187, 1172, 1112, 1023, 855, 762, 732, 624; ^{1}H NMR (400 MHz, acetone-*d$_6$*) δ 8.51 (d, *J* = 1.9 Hz, 1H, Ar-CH), 8.44–8.37 (m, 1H, Ar-CH), 8.04–7.96 (m, 2H, 2 × Ar-CH), 7.71 (dd, *J* = 8.4, 1.9 Hz, 1H, Ar-CH), 7.58–7.50 (m, 2H, 2 × Ar-CH), 5.25 (s, 1H, OH), 3.40–3.27 (m, 2H, 2 × CH*H*), 3.15–3.03 (m, 2H, 2 × C*H*H); ^{13}C{^{1}H} NMR (101 MHz, acetone-*d$_6$*) δ 142.6 (dd, *J* = 2.94, 2.75 Hz, Ar-*C$_q$*C$_q$), 139.7 (Ar-*C$_q$*S), 138.1 (Ar-*C$_q$*S), 135.5 (Ar-*C$_q$*C$_q$), 135.4 (Ar-*C$_q$*C$_q$), 127.0 (Ar-

CH), 124.7 (Ar-CH), 124.6 (Ar-CH), 122.9 (Ar-CH), 122.8 (Ar-CH), 121.9 (Ar-CH), 119.0 (dd, *J* = 273.6, 273.6 Hz, CF_2), 118.4 (Ar-CH), 66.5 (dd, *J* = 14.8, 12.7 Hz, C_q), 50.2 (dd, *J* = 22.3, 22.3 Hz, $CH_2CF_2CH_2$); ^{19}F NMR (377 MHz, acetone-d_6) δ –90.08 (d, *J* = 196.8 Hz), –92.11 (d, *J* = 196.8 Hz); HRMS (FTMS-ESI$^-$) *m/z* calcd for $C_{16}H_{11}F_2OS^-$ [M–H]$^-$: 289.050; found 289.0503.

3,3-Difluoro-1-(pyren-1-yl)cyclobutan-1-ol (8)

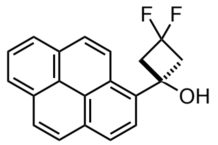

Prepared according to General Procedure A using 1-bromopyrene (309 mg, 1.1 mmol, 2.2 equiv). Purification by flash column chromatography (20% Et_2O/pentane) afforded difluorocyclobutanol **8** as a beige solid (93.8 mg, 61%). R_f = 0.14 (20% Et_2O/pentane); mp = 118–120 °C; IR (film)/cm^{-1} 3547 (OH, br), 3399, 3042, 1297, 1176, 1144, 1131, 1097, 1062, 993, 967, 926, 907, 844, 760, 728, 635, 514; ^{1}H NMR (400 MHz, $CDCl_3$) δ 8.33 (d, *J* = 9.3 Hz, 1H, Ar-CH), 8.22 (d, *J* = 7.6 Hz, 2H, 2 × Ar-CH), 8.15–8.00 (m, 5H, 5 × Ar-CH), 7.92 (d, *J* = 8.0 Hz, 1H, Ar-CH), 3.65–3.50 3.57 (m, 2H, 2 × CH*H*), 3.49–3.31 (m, 2H, 2 × C*H*H), 2.68 (s, 1H, OH); ^{13}C{^{1}H} NMR (101 MHz, $CDCl_3$) δ 135.5 (dd, *J* = 5.6, 4.3 Hz, Ar-C_qC_q), 131.7 (Ar-C_q), 131.2 (Ar-C_q), 130.4 (Ar-C_q), 128.5 (Ar-C_q), 128.1 (Ar-CH), 128.0 (Ar-CH), 127.2 (Ar-CH), 126.3 (Ar-CH), 125.7(Ar-CH), 125.7 (Ar-CH), 125.7 (Ar-C_q), 124.7 (Ar-C_q), 124.2 (Ar-CH), 124.1 (Ar-CH), 123.3 (Ar-CH), 118.7 (dd, *J* = 275.1, 275.1 Hz, CF_2), 69.2 (dd, *J* = 15.7, 11.3 Hz, C_q), 50.0 (dd, *J* = 22.5, 22.5 Hz, $CH_2CF_2CH_2$); ^{19}F NMR (377 MHz, $CDCl_3$) δ –90.74 (d, *J* = 198.8 Hz), –91.62 (d, *J* = 198.8 Hz); HRMS (TOF-ESI$^+$) *m/z* calcd for $C_{20}H_{14}OF_2^{\bullet}$ [M]$^{\bullet}$: 308.1013; found 308.1012.

1-(4-Chlorophenyl)-3,3-difluorocyclobutan-1-ol (11)

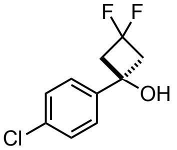

Prepared according to General Procedure A using 2-bromo-4-chlorobenzene (211 mg, 1.1 mmol, 2.2 equiv). Purification by flash column chromatography (20–30% Et_2O/pentane) afforded difluorocyclobutanol **11** as a clear, colorless oil (45.4 mg, 42%). R_f = 0.43 (40% Et_2O/pentane); IR (film)/cm^{-1} 3378 (OH, br), 2957, 1492, 1401, 1297, 1181, 1127, 1092, 1012, 636; ^{1}H NMR (400 MHz, $CDCl_3$) δ 7.47–7.37 (m, 4H, 4 × Ar-CH), 3.26–2.90 (m, 4H, $CH_2CF_2CH_2$), 2.20 (s, 1H, OH); ^{13}C{^{1}H} NMR (101 MHz, $CDCl_3$) δ 142.63 (dd, *J* = 5.8, 2.7 Hz, Ar-C_qC_q), 133.9 (Ar-C_qCl), 128.9 (2 × Ar-CH), 126.5 (2 × Ar-CH), 117.85 (dd, *J* = 275.8, 275.8 Hz, CF_2); 67.4 (dd, *J* = 14.8, 12.1 Hz, C_q); 50.3 (dd, *J* = 22.9, 22.9 Hz, $CH_2CF_2CH_2$); ^{19}F NMR (377 MHz, $CDCl_3$) δ –90.05 (d, *J* = 199.0 Hz), –92.93 (d, *J* = 199.0 Hz); HRMS (FTMS-ESI$^-$) *m/z* calcd for $C_{10}H_6{}^{35}ClF_2O^-$ [M–H_2–H]$^-$: 215.0092; found 215.0077.

3,3-Difluoro-1-(4-(trifluoromethyl)phenyl)cyclobutan-1-ol (12)

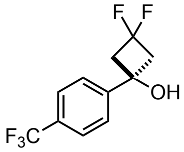

Prepared according to General Procedure A using 4-iodobenzotrifluoride (0.16 mL, 1.1 mmol, 2.2 equiv). Purification by flash column chromatography (20% Et_2O/pentane) afforded difluorocyclobutanol **12** as a yellow solid (90.0 mg, 72%). R_f = 0.23 (20% Et_2O/pentane); mp = 47–49 °C; IR (film)/cm^{-1} 3595 (OH, br), 3392, 1619, 1408, 1326, 1297, 1105, 1067, 1015, 846, 717, 609; ^{1}H NMR (400 MHz, $CDCl_3$) δ 7.69 (d, *J* = 8.2 Hz, 2H, 2 × Ar-CH), 7.63 (d, *J* = 8.2 Hz, 2H, 2 × Ar-CH), 3.30–3.12 (m, 2H, 2 × CH*H*), 3.10–2.96 (m, 2H, 2 × C*H*H), 2.34 (s, 1H, OH); ^{13}C{^{1}H} NMR (101 MHz, $CDCl_3$) δ 148.0 (*app*. d, *J* = 5.9 Hz, Ar-C_qC_q), 130.3 (q, *J* = 32.7 Hz, Ar-C_qCF_3), 125.8 (q, *J* = 3.8 Hz, 2 × Ar-CH), 125.4 (2 × Ar-CH), 121.5 (q, *J* = 273.9 Hz, CF_3), 117.7 (dd, *J* = 276.2, 276.2 Hz, CF_2), 67.4 (dd, *J* = 15.0, 12.3 Hz, C_q), 50.5 (dd, *J* = 23.1, 23.1 Hz, $CH_2CF_2CH_2$); ^{19}F NMR (377 MHz, $CDCl_3$) δ –62.63 (CF_3), –90.12 (d, *J* = 199.3 Hz, C*F*F), –92.60 (d, *J* = 199.3 Hz, CF*F*); HRMS (FTMS-ESI$^-$) *m/z* calcd for $C_{11}H_8F_5O^-$ [M–H]$^-$: 251.0501; found 251.0500.

3,3-Difluoro-1-(4-(5-(*p*-tolyl)-3-(trifluoromethyl)-1*H*-pyrazol-1-yl)phenyl)cyclobutan-1-ol (13)

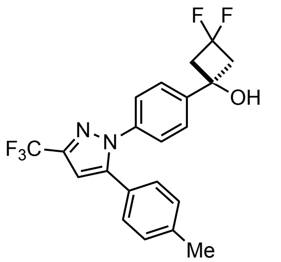

t-BuLi (1.69 M in pentane, 1.29 mL, 2.2 mmol, 4.4 equiv) was added dropwise to a solution of aryl bromide **S3** (419 mg, 1.1 mmol, 2.2 equiv) in anhydrous THF (2.5 mL, 0.2 M) at –78 °C. After stirring for 30 min, the reaction mixture was warmed to 0 °C and $LaCl_3$•2LiCl (0.6 M in THF, 1.83 mL, 1.1 mmol, 2.2 equiv) was added dropwise to the reaction mixture. After a further 1 h min of stirring, a solution of 3,3-difluorocyclobutanone (53 mg, 0.5 mmol, 1.0 equiv) in THF (0.65 mL × 2) was added dropwise. After stirring for 2 h at 0 °C, the reaction mixture was quenched with sat. aq. NH_4Cl (15 mL) and diluted with Et_2O (30 mL). The layers were separated, and the aqueous layer was extracted with Et_2O/pentane (2:1, 30 mL). The combined organic layers were washed with brine, dried over anhydrous Na_2SO_4, filtered, and concentrated *in vacuo*. Purification by flash column chromatography (30% Et_2O/pentane) afforded difluorocyclobutanol **13** as an off-white solid (162 mg, 80%). R_f = 0.26 (30% Et_2O/pentane); mp = 158–160 °C; IR (film)/cm^{-1} 3338 (OH, br), 2998, 1507, 1474, 1448, 1408, 1375, 1295, 1273, 1228, 1203, 1125, 1097, 1012, 985, 844, 823, 801, 754, 628, 613, 451; ^{1}H NMR (400 MHz, acetone-d_6) δ 7.64 (d, *J* = 8.2 Hz, 2H, 2 × Ar-CH), 7.40 (d, *J* = 8.2 Hz, 2H, 2 × Ar-CH), 7.27–7.18 (m, 4H, 4 × Ar-CH), 6.96 (s, 1H, Ar-CH), 5.27 (s, 1H, OH), 3.23–3.09 (m, 2H, 2 × CH*H*), 3.08–2.96 (m, 2H, 2 × *C*HH), 2.36 (s, 3H, CH_3); ^{13}C{^{1}H} NMR (101 MHz, acetone-d_6) δ 146.3 (dd, *J* = 6.0, 3.0 Hz, Ar-$C_q$$C_q$), 145.1(Ar-$C_q$Ar-$C_q$), 142.5 (q, *J* = 37.9 Hz, Ar-$C_q$$CF_3$), 139.1 (Ar-$C_q$N), 138.5 (Ar-$C_q$$CH_3$), 129.3 (2 × Ar-CH), 128.8 (2 × Ar-CH), 126.3 (Ar-C_qAr-C_q), 126.0 (2 × Ar-CH), 125.4 (2 × Ar-CH), 121.8 (q, *J* = 276.8, 276.8 Hz, CF_3), 118.8 (dd, *J* =273.6, 273.6, CF_2), 105.3 (Ar-CH), 66.0 (dd, *J* = 15.3, 12.1 Hz, C_q), 50.3 (dd, *J* = 22.5, 22.5 Hz, *C*H_2CF_2*C*H_2), 20.3 (CH_3); ^{19}F NMR (377 MHz, acetone-d_6) δ –62.61 (CF_3), –90.12 (d, *J* = 198.7 Hz, CF*F*), –92.21 (d, *J* = 198.7 Hz, C*F*F); HRMS (TOF-ESI$^+$) *m/z* calcd for $C_{21}H_{18}N_2OF_5^+$ [M+H]$^+$: 409.1339; found 409.1351.

3,3-Difluoro-1-(6-methoxypyridin-3-yl)cyclobutan-1-ol (14)

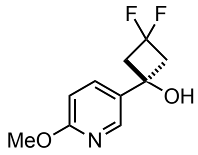

Prepared according to General Procedure B using 5-iodo-2-methoxypyridine (0.14 mL, 1.1 mmol, 2.2 equiv). Purification by flash column chromatography (30–50% Et_2O/pentane) afforded difluorocyclobutanol **14** as a clear colorless oil (80.5 mg, 75%). R_f = 0.22 (50% Et_2O/pentane); IR (film)/cm^{-1} 3363 (OH, br), 3015, 2952, 1605, 1571, 1494, 1373, 1289, 1192, 1174, 1133, 1023, 836; ^{1}H NMR (400 MHz, $CDCl_3$) δ 8.14 (d, *J* = 2.6 Hz, 1H, Ar-CH), 7.67 (dd, *J* = 8.7, 2.6 Hz, 1H, Ar-CH), 6.76 (d, *J* = 8.7 Hz, 1H, Ar-CH), 3.91 (s, 3H, OCH_3), 3.43 (s, 1H, OH), 3.14–2.91 (m, 4H, 2 × CH_2); ^{13}C{^{1}H} NMR (101 MHz, $CDCl_3$) δ 163.8 (Ar-C_qOMe), 143.6 (Ar-CH), 136.5 (Ar-CH), 132.6 (dd, *J* = 5.8, 2.8 Hz, Ar-$C_q$$C_q$), 117.8 (dd, *J* = 277.7, 274.3 Hz, CF_2), 110.9 (Ar-CH), 65.9 (dd, *J* = 15.8, 13.5 Hz, C_q), 53.7 (OCH_3), 49.9 (dd, *J* = 24.9, 22.8 Hz, *C*H_2CF_2*C*H_2); ^{19}F NMR (377 MHz, $CDCl_3$) δ –89.65 (d, J = 199.1 Hz), –92.88 (d, J = 199.1 Hz); HRMS (TOF-ESI$^+$) *m/z* calcd for $C_{10}H_{12}NF_2O_2^+$ [M+H]$^+$: 216.0836; found 216.0840.

3,3-Difluoro-1-(2-methoxypyridin-3-yl)cyclobutan-1-ol (15)

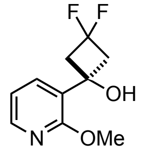

Prepared according to General Procedure B using 2-methoxy-3-iodopyridine (259 mg, 1.1 mmol, 2.2 equiv). Purification by flash column chromatography (50% Et_2O/pentane) afforded difluorocyclobutanol **15** as a yellow oil (77.8 mg, 72%). R_f = 0.21 (50% Et_2O/pentane); IR (film)/cm^{-1} 3394 (OH), 1582, 1464, 1405, 1291, 1246, 1172, 1131, 1090, 1013, 855, 775; ^{1}H NMR (400 MHz, $CDCl_3$) δ 8.14 (dd, *J* = 5.0, 1.8 Hz, 1H, Ar-CH), 7.56 (dd, *J* = 7.4, 1.8 Hz, 1H, Ar-CH), 6.94 (dd, *J* = 7.4, 5.0 Hz, 1H), 4.04 (s, 3H, OCH_3), 3.60 (s, 1H, OH), 3.23–3.06 (m, 2H, 2 × CH*H*), 2.99 (dddd, *J* = 14.5, 13.2, 9.9, 4.4 Hz, 2H, 2 × *C*HH); ^{13}C{^{1}H} NMR (101 MHz, $CDCl_3$) δ 161.0 (Ar-C_qOMe), 146.4 (2 × Ar-CH), 134.1, (2 × Ar-CH), 125.2 (dd, *J* = 6.0, 3.0 Hz, Ar-$C_q$$C_q$), 118.3 (dd, *J* = 275.9, 274.4 Hz, CF_2), 116.9 (Ar-$C_q$$C_q$), 65.7 (dd, *J* = 13.9, 13.5 Hz, C_q), 53.6 (OCH_3), 47.9 (dd, *J* = 27.3, 22.7 Hz, *C*H_2CF_2*C*H_2); ^{19}F NMR (377 MHz, $CDCl_3$) δ –89.75 (d, *J* = 197.3 Hz), –92.47 (d, *J* = 197.3 Hz); HRMS (TOF-ESI$^+$) *m/z* calcd for $C_{10}H_{12}NF_2O_2^+$ [M+H]$^+$: 216.0836; found 216.0828.

3,3-Difluoro-1-(6-methoxypyridin-3-yl)cyclobutan-1-ol (16)

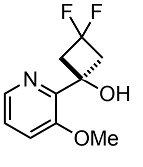

Prepared according to General Procedure B using 2-iodo-3-methoxypyridine (259 mg, 1.1 mmol, 2.2 equiv). Purification by flash column chromatography (50–60% Et_2O/pentane) afforded difluorocyclobutanol **16** as a clear colorless oil (44.1 mg, 42%). R_f = 0.20 (50% Et_2O/pentane); IR (film)/cm^{-1} 3554 (OH), 2963, 1585, 1463, 1431, 1397, 1278, 1194, 1108, 1017, 799; ^{1}H NMR (400 MHz, $CDCl_3$) δ 8.17 (dd, *J* = 4.0, 2.2 Hz, 1H, Ar-CH), 7.31–7.28 (m, 2H, 2 × Ar-CH), 5.51 (s, 1H, OH), 3.96 (s, 3H, OCH_3), 3.60–3.45 (m, 2H, 2 ×

CH*H*), 2.93–2.78 (m, 2H, 2 × C*H*H); $^{13}C\{^1H\}$ NMR (101 MHz, $CDCl_3$) δ 153.0 (Ar-C_qOMe), 149.2 (app. d, *J* = 5.9 Hz), 139.1 (Ar-CH), 124.1 (Ar-CH), 119.3 (dd, *J* = 278.2, 273.5 Hz, CF_2), 118.4 (Ar-CH), 66.1 (dd, *J* = 19.1, 10.1 Hz, C_q), 55.3 (OCH_3), 48.4, (dd, *J* = 24.5, 23.4 Hz, $CH_2CF_2CH_2$); ^{19}F NMR (377 MHz, $CDCl_3$) δ –89.61 (d, *J* = 195.8 Hz), –91.15 (d, *J* = 195.8 Hz); HRMS (TOF-ESI$^+$) *m/z* calcd for $C_{10}H_{12}NF_2O_2^+$ $[M+H]^+$: 216.0836; found 216.0833.

3,3-Difluoro-1-(6-morpholinopyridin-3-yl)cyclobutan-1-ol (17)

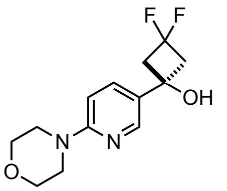

Prepared according to General Procedure B using 5-bromo-2-morpholinopyridine (267 mg, 1.1 mmol, 2.2 equiv). Purification by flash column chromatography (80% Et_2O/pentane) afforded difluorocyclobutanol **17** as a white solid (78.1 mg, 58%). R_f = 0.14 (80% Et_2O/pentane); mp = 104–105 °C; IR (film)/cm^{-1} 3392 (OH, br), 2961, 2894, 2853, 1604, 1558,1496, 1395, 1291, 1241, 1114, 1026, 943, 857, 814; ^1H NMR (400 MHz, $CDCl_3$) δ 8.14 (d, *J* = 2.5 Hz, 1H, Ar-CH), 7.59 (dd, *J* = 8.9, 2.5 Hz, 1H, Ar-CH), 6.65 (d, *J* = 8.9 Hz, 1H, Ar-CH), 3.81 (t, *J* = 4.9 Hz, 4H, 2 × OCH_2), 3.49 (t, *J* = 4.9 Hz, 4H, 2 × NCH_2), 3.44–3.29 (m, 1H, OH), 3.17–2.88 (m, 4H, 2 × CH_2); $^{13}C\{^1H\}$ NMR (101 MHz, $CDCl_3$) δ 159.0 (Ar-C_qN), 144.9 (Ar-CH), 135.4 (Ar-CH), 129.2 (dd, *J* = 5.7, 2.8 Hz, Ar-C_qC$_q$), 118.0 (dd, *J* = 277.8, 274.3 Hz, CF_2), 106.8 (Ar-CH), 66.6 (2 × OCH_2), 65.8 (dd, *J* = 14.4, 13.6 Hz, C_q), 49.6 (dd, *J* = 23.7, 22.6 Hz, $CH_2CF_2CH_2$), 45.6 (2 × NCH_2); ^{19}F NMR (377 MHz, $CDCl_3$) δ –89.57 (d, *J* = 198.3 Hz), –92.83 (d, *J* = 198.3 Hz); HRMS (TOF-ESI$^+$) *m/z* calcd for $C_{13}H_{17}N_2F_2O_2^+$ $[M+H]^+$: 271.1258; found 271.1258.

1-(Benzofuran-2-yl)-3,3-difluorocyclobutan-1-ol (18)

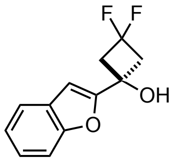

Prepared according to General Procedure A using 2,3-benzofuran (0.12 mL, 1.1 mmol, 2.2 equiv). Purification by flash column chromatography (25% Et_2O/pentane) afforded difluorocyclobutanol **18** as a yellow solid (99.8 mg, 90%). R_f = 0.25 (30% Et_2O/pentane); IR (film)/cm^{-1} 3365 (OH, br), 1453, 1295, 1243, 1164, 1032, 1012, 823, 808, 751, 704; ^1H NMR (400 MHz, $CDCl_3$) δ 7.59 (d, *J* = 8.1 Hz, 1H, Ar-CH), 7.51 (d, *J* = 8.2 Hz, 1H, Ar-CH), 7.39–7.24 (m, 2H, 2 × Ar-CH), 6.73 (s, 1H, Ar-CH), 3.38–3.22 (m, 2H, 2 × CH*H*), 3.14–2.93 (m, 2H, 2 × C*H*H), 2.71 (s, 1H, OH); $^{13}C\{^1H\}$ NMR (101 MHz, $CDCl_3$) δ 158.03 (dd, *J* = 6.4, 2.9 Hz, Ar-C_qC$_q$), 155.1 (Ar-C_qO), 127.9 (Ar-C_qAr-CH), 124.8 (Ar-CH), 123.2 (Ar-CH), 121.4 (Ar-CH), 117.44 (dd, *J* = 277.0, 274.5 Hz, CF_2), 111.4 (Ar-CH), 102.8 (Ar-CH), 63.7 (dd, *J* = 14.2, 14.2 Hz, C_q), 49.0 (dd, *J* = 23.6, 23.6 Hz, $CH_2CF_2CH_2$); ^{19}F NMR (377 MHz, $CDCl_3$) δ –88.64 (d, *J* = 199.0 Hz), –93.74 (d, *J* = 199.0 Hz); HRMS (TOF-ESI$^-$) *m/z* calcd for $C_{12}H_9F_2O_2^-$ $[M-H]^-$: 223.0576; found 223.0576.

3,3-Difluoro-1-(1-phenyl-1*H*-pyrazol-5-yl)cyclobutan-1-ol (19)

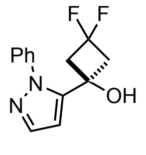

Prepared according to General Procedure A using 1-phenylpyrazole (0.15 mL, 1.1 mmol, 2.2 equiv). Purification by flash column chromatography (30–50% Et_2O/pentane) afforded difluorocyclobutanol **19** as a yellow solid (80.1 mg, 64%). R_f = 0.23 (50% Et_2O/pentane); IR (film)/cm^{-1} 3179 (OH), 1498, 1388, 1295, 1230, 1194, 1181, 1116, 1043, 982, 784, 767, 713, 693; ^1H NMR (400 MHz, acetone-d_6) δ 7.76 (d, *J* = 9.5 Hz, 2H, 2 × Ar-CH), 7.58 (d, *J* = 1.9 Hz, 1H, Ar-CH), 7.52 (dd, *J* = 8.4, 6.7 Hz, 2H, 2 × Ar-CH), 7.45 (t, *J* = 7.3 Hz, 1H, Ar-CH), 6.53 (d, *J* = 1.8 Hz, 1H, Ar-CH), 5.59 (s, 1H, OH), 3.08–2.93 (m, 2H, 2 × CH*H*), 2.93–2.76 (m, 2H, 2 × C*H*H); $^{13}C\{^1H\}$ NMR (101 MHz, acetone-d_6) δ 145.6 (dd, *J* = 6.8, 2.9 Hz, Ar-C_qC$_q$), 141.0 (Ar-C_qN), 139.0 (Ar-CH), 128.7 (2 × Ar-CH), 127.9 (Ar-CH), 125.4 (2 × Ar-CH), 118.3 (dd, *J* = 276.9, 272.3 Hz, CF_2), 106.5 (Ar-CH), 60.6 (dd, *J* =15.9, 14.4 Hz, C_q), 49.4 (dd, *J* = 24.7, 22.3 Hz, $CH_2CF_2CH_2$); ^{19}F NMR (377 MHz, $CDCl_3$) δ –89.26 (d, *J* = 197.6 Hz), –92.76 (d, *J* = 197.6 Hz); HRMS (TOF-ESI$^+$) *m/z* calcd for $C_{13}H_{13}N_2F_2O^+$ $[M+H]^+$: 251.0996; found 251.1003.

3,3-Difluoro-1-(phenylethynyl)cyclobutan-1-ol (20)

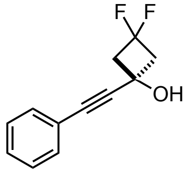

Prepared according to General Procedure A using phenylacetylene (0.12 mL, 1.1 mmol, 2.2 equiv). Purification by flash column chromatography (20% Et_2O/pentane) afforded difluorocyclobutanol **20** as an orange oil (94.2 mg, 90%). R_f = 0.23 (20% Et_2O/pentane); IR (film)/cm^{-1} 3343 (OH, br), 2957, 2230 (C≡C), 1490, 1399, 1295, 1168, 1045, 918, 852, 754, 689, 547, 523, 501, 475, 460; ^1H NMR (400 MHz, $CDCl_3$) δ 7.47 (dd, J = 7.6, 2.1 Hz, 2H, 2 × Ar-CH), 7.44–7.32 (m, 3H, 3 × Ar-CH), 3.33–3.10 (m, 2H, 2 × CH*H*), 3.11–2.93 (m, 2H, 2 × C*H*H), 2.75 – 2.52 (m, 1H, OH); $^{13}C\{^1H\}$ NMR (101 MHz, $CDCl_3$) δ 131.7 (2 × Ar-CH), 128.9 (Ar-CH), 128.4 (2 × Ar-CH), 121.8 (Ar-C_qC$_q$), 117.3 (dd, *J* = 272.6, 272.6 Hz, CF_2), 89.9 (dd, *J* = 4.1, 3.1 Hz, C_qC$_q$), 84.9 (Ar-C$_q$$C_q$), 58.5 (dd, *J* = 17.8, 15.5 Hz, C_q), 51.8 (dd, *J* = 24.1, 24.1 Hz, $CH_2CF_2CH_2$); ^{19}F NMR (377 MHz, $CDCl_3$) δ

–88.21 (d, *J* = 201.0 Hz), –94.46 (d, *J* = 201.0 Hz); HRMS (FTMS-ESI$^+$) *m/z* calcd for $C_{12}H_{11}F_2O^+$ [M+H]$^+$: 209.0772; found 209.0773.

3,3-Difluoro-1-((triisopropylsilyl)ethynyl)cyclobutan-1-ol (21)

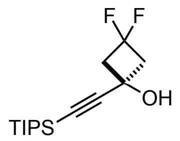

Prepared according to General Procedure A using TIPS acetylene (0.25 mL, 1.1 mmol, 2.2 equiv). Purification by flash column chromatography (10% Et_2O/pentane) afforded difluorocyclobutanol **21** as a clear colorless oil (125 mg, 87%). R_f = 0.27 (10% Et_2O/pentane); IR (film)/cm^{-1} 3363 (OH, br), 2942, 2892, 2866, 1463, 1299, 1209, 1049, 997, 881, 859, 766, 674, 659; ^{1}H NMR (400 MHz, $CDCl_3$) δ 3.16–3.01 (m, 2H, 2 × CH*H*), 3.01–2.86 (m, 2H, 2 × C*H*H), 2.49–2.42 (m, 1H, OH), 1.09 (s, 21H, 3 × CH, 6 × CH_3); $^{13}C\{^1H\}$ NMR (101 MHz, $CDCl_3$) δ 117.0 (dd, *J* = 277.7, 271.9 Hz, CF_2), 108.5 (dd, *J* = 7.3, 2.6 Hz, *C_q*C_q), 86.3 (C_qSi), 58.3 (dd, *J* = 18.2, 12.5 Hz, C_qOH), 52.0 (dd, *J* = 23.5, 23.5 Hz, *C*H_2CF_2*C*H_2), 18.5 (6 × CH_3), 11.0 (3 × CH); ^{19}F NMR (377 MHz, $CDCl_3$) δ –87.67 (d, *J* = 198.8 Hz), –95.51 (d, *J* = 198.8 Hz); HRMS (FTMS-ESI$^-$) *m/z* calcd for $C_{15}H_{25}F_2OSi^-$ [M–H]$^-$: 287.1648; found 287.1649.

3,3-Difluoro-1-(2-(methoxymethoxy)phenyl)cyclobutan-1-ol (22)

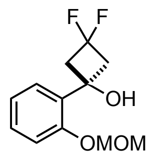

n-BuLi (1.59 M in hexanes, 0.63 mL, 1.0 mmol, 2.0 equiv) was added dropwise to a solution of MOM-protected phenol **S4** (152 mg, 1.1 mmol, 2.2 equiv) in anhydrous THF (2 mL, 0.25 M) at 0 °C. After stirring for 1 h at 0 °C, $LaCl_3$•2LiCl (0.6 M in THF, 1.83 mL, 1.1 mmol, 2.2 equiv) was added dropwise to the reaction mixture. After a further 1 h of stirring, a solution of 3,3-difluorocyclobutanone (53 mg, 0.5 mmol, 1.0 equiv) in THF (0.65 mL × 2) was added dropwise. After stirring for 2 h, the reaction mixture was quenched with sat. aq. NH_4Cl (15 mL) and diluted with Et_2O (30 mL). The layers were separated, and the aqueous layer was extracted with Et_2O/pentane (2:1, 30 mL). The combined organic layers were washed with brine, dried over anhydrous Na_2SO_4, filtered, and concentrated *in vacuo*. Purification by flash column chromatography (30–40% Et_2O/pentane) afforded difluorocyclobutanol **22** as a clear colorless oil (86.1 mg, 71%). R_f = 0.28 (40% Et_2O/pentane); IR (film)/cm^{-1} 3546 (OH, br), 3434, 2959, 1600, 1489, 1295, 1181, 1155, 985, 922, 755; ^{1}H NMR (400 MHz, $CDCl_3$) δ 7.37–7.25 (m, 2H, 2 × Ar-CH), 7.17 (d, *J* = 8.2 Hz, 1H, Ar-CH), 7.06 (tt, *J* = 7.5, 1.0 Hz, 1H, 2 × Ar-CH), 5.30 (s, 2H, OCH_2), 3.50 (s, 4H, OCH_3 + OH), 3.24–3.12 (m, 2H, 2 × CH*H*), 3.10–2.95 (m, 2H, 2 × C*H*H); $^{13}C\{^1H\}$ NMR (101 MHz, $CDCl_3$) δ 154.8 (Ar-*C_q*OMe), 131.8 (dd, *J* = 5.5, 4.6 Hz, Ar-*C_q*C_q), 129.6 (Ar-CH), 126.0 (Ar-CH), 118.9 (dd, *J* = 276.4, 276.0 Hz, CF_2) 114.6 (Ar-CH), 94.6 (OCH_2), 66.8 (dd, *J* = 15.3, 12.4 Hz, C_q), 56.6 (OCH_3), 48.4 (dd, *J* = 24.9, 22.4 Hz, *C*H_2CF_2*C*H_2); ^{19}F NMR (377 MHz, $CDCl_3$) δ –90.65 (d, *J* = 196.7 Hz), –91.29 (d, *J* = 196.7 Hz); HRMS (FTMS-APCI$^-$) *m/z* calcd for $C_{12}H_{13}F_2O_3^-$ [M–H]$^-$: 243.0838; found 243.0837.

***tert*-Butyl 2-(3,3-difluoro-1-hydroxycyclobutyl)pyrrolidine-1-carboxylate (23)**

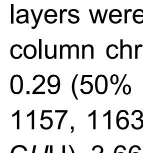

s-BuLi (1.33 M in cyclohexane, 0.75 mL, 1.0 mmol, 2.0 equiv) was added dropwise to a solution of 1-Boc-pyrrolidine (0.19 mL, 1.1 mmol, 2.2 equiv) in anhydrous THF (3.5 mL, 0.14 M) at –78 °C. After stirring for 1 h, $LaCl_3$•2LiCl (0.6 M in THF, 1.83 mL, 1.1 mmol, 2.2 equiv) was added dropwise to the reaction mixture. After a further 1.5 h of stirring at –78 °C, the reaction mixture was warmed to 0 °C. After a further 30 min of stirring, a solution of 3,3-difluorocyclobutanone (53 mg, 0.5 mmol, 1.0 equiv) in THF (0.65 mL × 2) was added dropwise. After stirring for 2 h at 0 °C, the reaction mixture was quenched with sat. aq. NH_4Cl (15 mL) and diluted with Et_2O (30 mL). The layers were separated, and the aqueous layer was extracted with Et_2O/pentane (2:1, 30 mL). The combined organic layers were washed with brine, dried over anhydrous Na_2SO_4, filtered, and concentrated *in vacuo*. Purification by flash column chromatography (50% Et_2O/pentane) afforded difluorocyclobutanol **23** as a yellow solid (68.3 mg, 50%). R_f = 0.29 (50% Et_2O/pentane); mp = 129–130 °C; IR (film)/cm^{-1} 3384 (OH, br), 2976, 288, 1662 (C=O), 1392, 1366, 1291, 1157, 1163, 918, 937, 866, 773; ^{1}H NMR (400 MHz, $CDCl_3$) δ 6.17–5.55 (br, 1H, OH), 4.03 (dd, *J* = 7.8, 6.5 Hz, 1H, C*H*H), 3.66 (ddd, *J* = 11.5, 7.2, 4.6 Hz, 1H, CH*H*), 3.32–3.24 (m, 1H, CH), 3.00–2.85 (m, 1H, CH*H*), 2.60 (tddt, *J* = 28.4, 14.4, 9.9, 4.3 Hz, 3H, C*H*H + CH_2), 2.09 (dt, *J* = 13.0, 6.9 Hz, 1H, C*H*H), 2.01–1.87 (m, 1H, CH*H*), 1.77 (ddt, *J* = 15.6, 11.6, 6.0 Hz, 2H, CH_2), 1.47 (s, 9H, 3 × CH_3); $^{13}C\{^1H\}$ NMR (126 MHz, $CDCl_3$) δ 157.2 (C_q=O), 118.5 (dd, *J* = 276.4, 276.4 Hz, CF_2), 80.8 (*C_q*$(CH_3)_3$), 68.6 (dd, *J* = 18.0, 13.2 Hz, C_q), 64.6 (CH), 48.6 (CH_2), 47.4 (dd, *J* = 25.4, 22.7 Hz, CF_2CH_2), 45.5 (dd, *J* = 24.9, 22.5 Hz, CF_2*C*H_2), 28.3 (3 × CH_3), 24.1 (CH_2); ^{19}F NMR (377 MHz, $CDCl_3$) δ –88.64 (d, *J* = 197.8 Hz), –93.25 (d, *J* = 197.8 Hz); HRMS (TOF-ESI$^+$) *m/z* calcd for $C_{13}H_{22}NO_3F_2^+$ [M+H]$^+$: 278.1568; found 278.1556.

Limitations in the Synthesis of Difluorocyclobutanols

Below are the unsuccessful substrates in synthesis of difluorocyclobutanols. Lithiation, transmetalation, and addition of trimethoxybenzene **S10** into difluorocyclobutanone led to dimerisation of the aryl halide alongside the desired reaction, which was inseparable from the product by flash column chromatography. Substrates with strong coordinating groups, such as **S11** and **S12** led to a complex mixture of products, likely due to failed transmetalation. Ataluren-derived aryl iodide **S13** and sulfone **S14** were also unsuccessful. Deprotonation of *N*-methyl indole **S14** led to the formation of an elimination-derived side product similar to **2** and **S9**. The use of electron-deficient heteroaryl halides such as **S16**–**S18** resulted in a complex mixture of products, likely due to the instability of the corresponding anions.

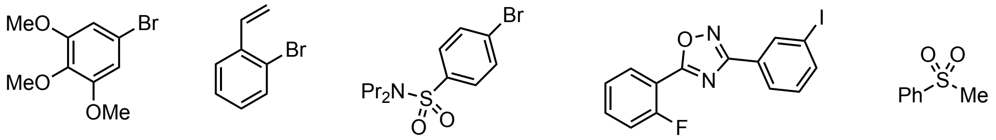

S10 **S11** **S12** **S13** **S14**

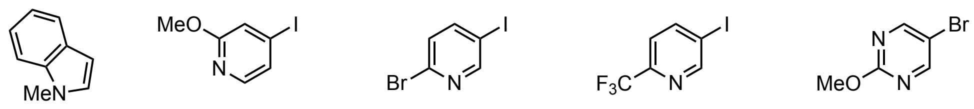

S15 **S16** **S17** **S18** **S18**

Figure S2: Unsuccessful substrates in the lithiation, transmetalation, and addition reaction with difluorocyclobutanone.

Trapping of Difluorocyclobutane Carbocation

General Procedure C

$FeCl_3$ (1.6 mg, 0.01 mmol, 10 mol%) was added to a solution of difluorocyclobutanol (0.1 mmol, 1.0 equiv) and nucleophile (0.3 mmol, 3.0 equiv) in PhMe (0.5 mL, 0.2 M). The vial was sealed and stirred for 3 h at 40 °C or 110 °C. After cooling to rt, the reaction mixture was quenched with sat. aq. $NaHCO_3$ (10 mL) and diluted with CH_2Cl_2 (15 mL). The layers were separated, and the aqueous layer was extracted with CH_2Cl_2 (3 × 15 mL). The combined organic layers were dried over anhydrous Na_2SO_4, filtered, and concentrated *in vacuo*. Purification by flash column chromatography under the stated conditions afforded the 1,1-disubstituted difluorocyclobutane.

N.B. Extraction could also be carried out with EtOAc instead of CH_2Cl_2.

Diaryl Difluorocyclobutanes 24–44

4-(3,3-Difluoro-1-(4-hydroxyphenyl)cyclobutyl)-2-methylphenol (25)

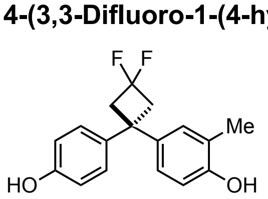

Prepared according to General Procedure C at 110 °C for 3 h using *p*-OH difluorocyclobutanol **9** (20.0 mg, 0.1 mmol, 1.0 equiv) and *o*-cresol (32.4 mg, 0.3 mmol, 3.0 equiv). Purification by flash column chromatography (50% Et_2O/pentane) afforded diaryl difluorocyclobutane **25** as a white foam (27.4 mg, 94%). R_f = 0.29 (50% Et_2O/pentane); IR (film)/cm^{-1} 3399 (OH, br), 1511, 1302, 1269, 1235, 1179, 1125, 1049, 1023, 1002, 821, 760; ^{1}H NMR (400 MHz, DMSO-d_6) δ 9.27 (s, 1H, OH), 9.16 (s, 1H, OH), 7.16–7.08 (m, 2H, 2 × Ar-CH), 7.01 (d, *J* = 2.5 Hz, 1H, 2 × Ar-CH), 6.95 (dd, *J* = 8.3, 2.5 Hz, 1H, Ar-CH), 6.70–6.63 (m, 3H, 3 × Ar-CH), 3.28–3.16 (m, 4H, 2 × CH_2), 2.07 (s, 3H, CH_3); ^{13}C{^{1}H} NMR (101 MHz, DMSO-d_6) δ 155.7 (Ar-C_qOH), 153.8 (Ar-C_qOH), 139.1 (Ar-C_qC$_q$), 138.8 (Ar-C_qC$_q$), 128.8 (Ar-CH), 127.5 (2 × Ar-CH), 124.6 (Ar-CH), 124.1 (Ar-C_qMe), 120.2 (dd, *J* = 276.7, 274.5 Hz, CF_2), 115.4 (2 × Ar-CH), 114.7 (Ar-CH), 48.1 (dd, *J* = 20.9, 19.4 Hz, $CH_2CF_2CH_2$), 38.8 (assigned by HMBC), 16.6 (CH_3); ^{19}F NMR (377 MHz, DMSO-d_6) δ –87.14 (d, *J* = 190.2 Hz), –86.60 (d, *J* = 190.2 Hz); HRMS (FTMS-ESI$^-$) *m/z* calcd for $C_{17}H_{15}F_2O_2^-$ [M–H]$^-$: 289.1046; found 289.1046.

4-(3,3-Difluoro-1-(4-methoxyphenyl)cyclobutyl)-2-methylphenol (24)

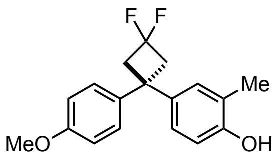

Prepared according to General Procedure C at 40 °C for 3 h using PMP difluorocyclobutanol **1** (21.4 mg, 0.1 mmol, 1.0 equiv) and *o*-cresol (32.4 mg, 0.3 mmol, 3.0 equiv). Purification by flash column chromatography (20% Et_2O/pentane) afforded diaryl difluorocyclobutane **24** as a clear colorless gum (27.6 mg, 91%). R_f = 0.25 (40% Et_2O/pentane); IR (film)/cm^{-1} 3417 (OH, br), 3008, 2954, 2841, 1608, 1506, 1301, 1241, 1115, 1031, 816, 737, 668, 626, 550; ^{1}H NMR (400 MHz, $CDCl_3$) δ 7.20 (d, *J* = 7.0 Hz, 2H, 2 × Ar-CH), 7.05–6.93 (m, 2H, 2 × Ar-CH), 6.91–6.83 (m, 2H, 2 × Ar-CH), 6.72 (d, *J* = 8.2 Hz, 1H, Ar-CH), 4.78 (s, 1H, OH), 3.81 (s, 3H, CH_3), 3.33 (dd, *J* = 13.4, 12.4 Hz, 4H, 2 × CH_2), 2.24 (s, 3H, CH_3); ^{13}C{^{1}H} NMR (101 MHz, $CDCl_3$) δ 157.8 (Ar-C_qOMe), 152.1 (Ar-C_qOH), 139.9 (2 × Ar-C_qC$_q$), 129.0 (Ar-CH), 127.4 (2 × Ar-CH), 124.9 (Ar-CH), 123.9 (Ar-C_qMe), 119.01 (dd, *J* = 278.3, 270.4 Hz, CF_2), 114.8 (Ar-CH), 113.9 (2 × Ar-CH), 55.3 (OCH_3), 48.5 (dd, *J* = 22.1, 21.8 Hz, $CH_2CF_2CH_2$), 39.2 (dd, *J* = 10.9, 10.7 Hz, C$_q$), 16.0 (CH_3); ^{19}F NMR (377 MHz, $CDCl_3$) δ –88.4 (d, *J* = 194.0 Hz), –89.0 (d, *J* = 194.0 Hz); HRMS (FTMS-ESI$^-$) *m/z* calcd for $C_{18}H_{17}F_2O_2^-$ [M–H]$^-$: 303.1202; found 303.1194.

*N.B. Carrying out this reaction on a 0.3 mmol scale afforded diaryl difluorocyclobutane **24** in 90% yield.*

4-(3,3-Difluoro-1-(4-methoxyphenyl)cyclobutyl)-2-methylphenol (26)

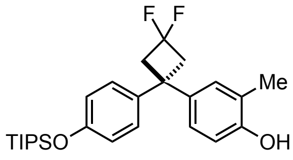

Prepared according to General Procedure C at 110 °C for 3 h using *p*-OTIPS difluorocyclobutanol **4** (35.7 mg, 0.1 mmol, 1.0 equiv) and *o*-cresol (32.4 mg, 0.3 mmol, 3.0 equiv). Purification by flash column chromatography (15% Et_2O/pentane) afforded diaryl difluorocyclobutane **26** as a clear colorless gum (30.9 mg, 69%). R_f = 0.18 (20% Et_2O/pentane); IR (film)/cm^{-1} 3598 (OH, br), 2944, 2892, 2866, 1606, 1507, 1463, 1300, 1361, 1177, 1144, 1114, 913, 881, 833, 684; ^{1}H NMR (400 MHz, $CDCl_3$) δ 7.11–7.06 (m, 2H, 2 × Ar-CH), 7.02–6.95 (m, 2H, 2 × Ar-CH), 6.86–6.79 (m, 2H, 2 × Ar-CH), 6.75–6.70 (m, 1H, Ar-CH), 4.62 (s, 1H, OH), 3.31 (dd, J = 12.4, 12.4 Hz, 4H, 2 × CH_2), 2.23 (s, 3H, CH_3), 1.26 (ddd, J = 14.6, 8.4, 6.5 Hz, 3H, CH), 1.11 (d, J = 7.3 Hz, 18H, 6 × CH_3); ^{13}C{^{1}H} NMR (101 MHz, $CDCl_3$) δ 154.3 (Ar-C_qOTIPS), 152.0 (Ar-C_qOH), 140.1 (Ar-C_qC$_q$), 139.9 (Ar-C_qC$_q$), 129.2 (Ar-CH), 127.4 (2 ×

Ar-CH), 125.1 (Ar-CH), 123.6 (Ar-*Cq*Me), 119.7 (2 × Ar-CH), 119.1 (dd, *J* = 276.8, 275.4 Hz, CF_2), 114.8 (Ar-CH), 48.7 (dd, *J* = 23.2, 21.9 Hz, $CH_2CF_2CH_2$), 39.1 (dd, *J* = 12.5, 10.0 Hz, C_q), 17.9 (6 × CH_3), 16.0 (CH_3), 12.6 (3 × CH); ^{19}F NMR (377 MHz, $CDCl_3$) δ –88.25 (d, *J*= 194.4 Hz), –89.01 (d, *J* = 194.4 Hz); HRMS (TOF-ESI$^+$) *m/z* calcd for $C_{26}H_{36}F_2O_2Si^{\bullet}$ [M]$^{\bullet}$: 446.2453; found 446.2450.

4-(1-(4-Chlorophenyl)-3,3-difluorocyclobutyl)-2-methylphenol (27)

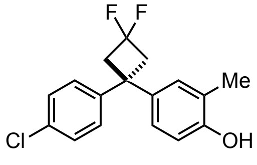

Prepared according to General Procedure C at 110 °C for 3 h using *p*-Cl difluorocyclobutanol **11** (21.8 mg, 0.1 mmol, 1.0 equiv) and *o*-cresol (32.4 mg, 0.3 mmol, 3.0 equiv). Purification by flash column chromatography (20% Et_2O/pentane) afforded diaryl difluorocyclobutane **27** as a clear colorless gum (20.5 mg, 67%). R_f = 0.42 (40% Et_2O/pentane); IR (film)/cm^{-1} 3585 (OH, br), 2957, 2926, 2855, 1508, 1492, 1410, 1302, 1261, 1237, 1116, 1095, 1012, 922, 868, 818, 758, 734, 594, 527, 452; ^{1}H NMR (400 MHz, $CDCl_3$) δ 7.33–7.26 (m, 2H, 2 × Ar-CH), 7.21 (d, *J* = 8.6 Hz, 2H, 2 × Ar-CH), 7.01–6.94 (m, 2H, 2 × Ar-CH), 6.73 (d, *J* = 8.2 Hz, 1H, Ar-CH), 4.68 (s, 1H, OH), 3.42–3.24 (m, 4H, 2 × CH_2), 2.24 (s, 3H, CH_3); $^{13}C\{^1H\}$ NMR (101 MHz, $CDCl_3$) δ 152.3 (Ar-*Cq*OH), 146.2 (Ar-*Cq*C_q), 139.0 (Ar-*Cq*C_q), 132.1 (Ar-*Cq*Cl), 129.0 (Ar-CH), 128.7 (2 × Ar-CH), 127.7 (2 × Ar-CH), 124.9 (Ar-CH), 124.1 (Ar-*Cq*Me), 118.6 (dd, *J* = 276.8, 273.1 Hz, CF_2), 115.0 (Ar-CH), 48.3 (dd, *J* = 22.1, 19.8 Hz, $CH_2CF_2CH_2$), 39.6 (dd, *J* = 11.7, 10.6 Hz, C_q), 16.0 (CH_3); ^{19}F NMR (377 MHz, $CDCl_3$) δ –88.55 (d, *J* = 195.2 Hz), –89.18 (d, *J* = 195.2 Hz); HRMS (FTMS-ESI$^-$) *m/z* calcd for $C_{17}H_{14}{}^{35}ClF_2O^-$ [M–H]$^-$: 307.0707; found 307.0695.

4-(3,3-Difluoro-1-(3-hydroxyphenyl)cyclobutyl)-2-methylphenol (28)

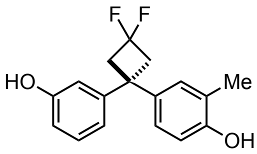

Prepared according to General Procedure C at 110 °C for 3 h using *m*-OH difluorocyclobutanol **10** (20.0 mg, 0.1 mmol, 1.0 equiv) and *o*-cresol (32.4 mg, 0.3 mmol, 3.0 equiv). Purification by flash column chromatography (50% Et_2O/pentane) afforded diaryl difluorocyclobutane **28** as a clear colorless gum (17.6 mg, 61%). R_f = 0.33 (50% Et_2O/pentane); IR (film)/cm^{-1} 3352 (OH, br), 2955, 1587, 1507, 1446, 1410, 1303, 1246, 1181, 1142, 1120, 928, 878, 818, 730, 702; ^{1}H NMR (400 MHz, acetone-d_6) δ 8.54 (br, 1H, OH), 8.35 (br, 1H, OH), 7.15–7.08 (m, 2H, 2 × Ar-CH), 7.04 (dd, *J* = 8.3, 2.5 Hz, 1H, Ar-CH), 6.86 (ddd, *J* = 7.9, 2.0, 1.0 Hz, 1H, Ar-CH), 6.82 (t, *J* = 2.0 Hz, 1H, Ar-CH), 6.75 (d, *J* = 8.3 Hz, 1H, Ar-CH), 6.63 (ddd, *J* = 7.9, 2.5, 1.0 Hz, 1H, Ar-CH), 3.44–3.26 (m, 4H, 2 × CH_2), 2.16 (s, 3H, CH_3); $^{13}C\{^1H\}$ NMR (101 MHz, acetone-d_6) δ 157.4 (Ar-*Cq*OH), 153.7 (Ar-*Cq*OH), 150.2 (Ar-*Cq*C_q), 138.4 (Ar-*Cq*C_q), 129.3 (Ar-CH), 128.8 (Ar-CH), 124.6 (Ar-CH), 124.1 (Ar-*Cq*Me), 119.4 (dd, *J* = 277.9, 275.7 Hz, CF_2), 117.1 (Ar-CH), 114.4 (Ar-CH), 113.3 (Ar-CH), 112.8 (Ar-CH), 47.7 (dd, *J* = 27.0, 21.7 Hz, $CH_2CF_2CH_2$), 39.8 (dd, *J* = 11.5, 11.1 Hz, C_q), 15.5 (CH_3); ^{19}F NMR (377 MHz, acetone-d_6) δ –88.64 (d, *J* = 193.2 Hz), –89.58 (d, *J* = 193.2 Hz); HRMS (FTMS-APCI$^-$) *m/z* calcd for $C_{17}H_{15}F_2O_2^-$ [M–H]$^-$: 289.1046; found 289.1041.

4-(3,3-Difluoro-1-(3-((triisopropylsilyl)oxy)phenyl)cyclobutyl)-2-methylphenol (29) and 2-(3,3-difluoro-1-(3-((triisopropylsilyl)oxy)phenyl)cyclobutyl)-6-methylphenol (29')

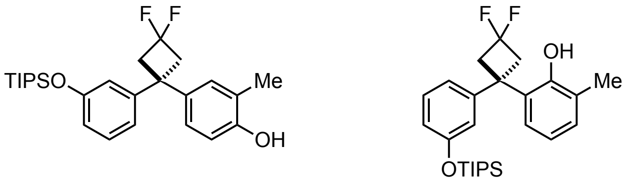

Prepared according to General Procedure C at 110 °C for 3 h using *m*-OTIPS difluorocyclobutanol **6** (35.7 mg, 0.1 mmol, 1.0 equiv) and *o*-cresol (32.4 mg, 0.3 mmol, 3.0 equiv). Purification by flash column chromatography (10% Et_2O/pentane) afforded diaryl difluorocyclobutane **29'** as a yellow oil (6.2 mg, 14%) followed by diaryl difluorocyclobutane **29** as a clear colorless oil (24.5 mg, 55%).

N.B. The observed isolated ratio of 4:1 ***29*:*29'*** *is consistent with that observed in the ^{19}F NMR of the crude reaction mixture.*

Diaryl difluorocyclobutane **29:** R_f = 0.12 (10% Et_2O/pentane); IR (film)/cm^{-1} 3598 (OH, br), 2944, 2866, 1599, 1582, 1507, 1483, 1463, 1429, 1300, 1276, 1246, 1198, 1179, 1116, 1000, 944, 881, 790, 684; ^{1}H NMR (400 MHz, $CDCl_3$) δ 7.17 (t, *J* = 7.8 Hz, 1H, Ar-CH), 6.98 (d, *J* = 6.8 Hz, 2H, 2 × Ar-CH), 6.88–6.84 (m, 1H, Ar-CH), 6.76–6.70 (m, 3H, 3 × Ar-CH),

4.65 (s, 1H, OH), 3.36–3.19 (m, 4H, 2 × CH_2), 2.23 (s, 3H, CH_3), 1.22 (ddt, *J* = 13.4, 10.6, 6.3 Hz, 3H, 3 × CH), 1.09 (d, *J* = 7.2 Hz, 18H, 6 × CH_3); $^{13}C\{^1H\}$ NMR (101 MHz, $CDCl_3$) δ 156.1 (Ar-C_qOTIPS), 152.1 (Ar-C_qOH), 149.3 (Ar-$C_q$$C_q$), 139.4 (Ar-$C_q$$C_q$), 129.4 (Ar-CH), 129.2 (Ar-CH), 125.0 (Ar-CH), 123.7 (Ar-C_qMe), 118.9 (dd, *J* = 276.8, 275.7 Hz, CF_2), 118.8 (Ar-CH), 118.3 (Ar-CH), 117.8 (Ar-CH), 114.8 (Ar-CH), 48.4 (dd, *J* = 24.9, 22.0 Hz, *C*H_2CF_2*C*H_2), 39.7 (dd, *J* = 11.7, 10.8 Hz, C_q), 17.9 (6 × CH_3), 15.9 (CH_3), 12.7 (3 × CH); ^{19}F NMR (377 MHz, $CDCl_3$) δ –88.51 (d, *J* = 194.6 Hz), –89.11 (d, *J* = 194.6 Hz); HRMS (FTMS-ESI$^-$) *m/z* calcd for $C_{26}H_{35}F_2O_2Si^-$ [M–H]$^-$: 445.2380; found 445.2378.

Diaryl difluorocyclobutane **29'**: R_f = 0.31 (10% Et_2O/pentane); IR (film)/cm^{-1} 3602 (OH, br), 2944, 2866, 1599, 1582, 1483, 1463, 1431, 1300, 1272, 946, 883, 784, 745, 684; ^1H NMR (400 MHz, $CDCl_3$) δ 7.21–7.15 (m, 2H, 2 × Ar-CH), 7.07 (d, *J* = 7.4 Hz, 1H, Ar-CH), 7.01 (dd, *J* = 7.9, 1.9 Hz, 1H, Ar-CH), 6.92 (t, *J* = 7.6 Hz, 1H, Ar-CH), 6.82 (t, *J* = 2.2 Hz, 1H, Ar-CH), 6.74 (dd, *J* = 8.0, 2.4 Hz, 1H, Ar-CH), 4.41 (s, 1H, OH), 3.34 (dd, *J* = 15.3, 11.9 Hz, 4H, 2 × CH_2), 2.19 (s, 3H, CH_3), 1.24–1.14 (m, 3H, 3 × CH), 1.05 (d, *J* = 7.2 Hz, 18H, 6 × CH_3); $^{13}C\{^1H\}$ NMR (101 MHz, $CDCl_3$) δ 156.2 (Ar-C_qOTIPS), 151.5 (Ar-C_qOH), 146.8 (*app.* d, *J* = 3.2 Hz, Ar-$C_q$$C_q$), 132.5 (*app.* d, *J* = 5.4 Hz, Ar-$C_q$$C_q$), 129.7 (Ar-CH), 129.5 (Ar-CH), 125.1 (Ar-CH), 124.4 (Ar-C_qMe), 120.4 (Ar-CH), 119.2 (dd, *J* = 278.3, 275.2 Hz, CF_2), 118.7 (Ar-CH), 118.4 (Ar-CH), 118.3 (Ar-CH), 47.5 (dd, *J* = 25.1, 22.2 Hz, *C*H_2CF_2*C*H_2), 38.2 (dd, *J* = 14.4, 8.7 Hz, C_q), 17.8 (6 × CH_3), 15.6 (CH_3), 12.6 (3 × CH); ^{19}F NMR (377 MHz, $CDCl_3$) δ –85.98 (d, *J* = 193.5 Hz), –92.04 (d, *J* = 193.5 Hz); HRMS (FTMS-ESI$^-$) *m/z* calcd for $C_{26}H_{35}F_2O_2Si^-$ [M–H]$^-$: 445.2380; found 445.2381.

4-(1-(Benzofuran-2-yl)-3,3-difluorocyclobutyl)-2-methylphenol (30)

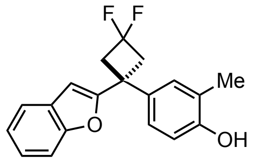

Prepared according to General Procedure C at 110 °C for 3 h using benzofuran difluorocyclobutanol **18** (20.8 mg, 0.1 mmol, 1.0 equiv) and *o*-cresol (32.4 mg, 0.3 mmol, 3.0 equiv). Purification by flash column chromatography (30% Et_2O/pentane) afforded diaryl difluorocyclobutane **30** as a clear colorless gum (26.4 mg, 84%). R_f = 0.25 (30% Et_2O/pentane); IR (film)/cm^{-1} 3588 (OH, br), 3017, 2959, 2924, 2855, 1507, 1453, 1300, 1243, 1164, 1135, 1114, 1079, 908, 877, 807, 751; ^1H NMR (400 MHz, $CDCl_3$) δ 7.53–7.43 (m, 2H, 2 × Ar-CH), 7.32–7.18 (m, 2H, 2 × Ar-CH), 7.16–7.07 (m, 2H, 2 × Ar-CH), 6.80 (d, *J* = 8.2 Hz, 1H, Ar-CH), 6.29 (d, *J* = 0.9 Hz, 1H, Ar-CH), 4.81 (s, 1H, OH), 3.59–3.44 (m, 2H, CH_2), 3.37–3.24 (m, 2H, CH_2), 2.29 (s, 3H, CH_3); $^{13}C\{^1H\}$ NMR (101 MHz, $CDCl_3$) δ 161.8 (app. d, *J* = 3.5 Hz, Ar-$C_q$$C_q$) 155.1 (Ar-$C_q$OH), 152.8 (Ar-$C_q$OAr-$C_q$), 135.4 (app. d, *J* = 3.5 Hz, Ar-$C_q$$C_q$), 129.6 (Ar-CH), 128.4 (Ar-C_qAr-CH), 125.6 (Ar-CH), 124.0 (Ar-CH + Ar-C_qMe), 122.8 (Ar-CH), 120.8 (Ar-CH), 118.6 (dd, *J* = 276.6, 274.7 Hz, CF_2), 114.9 (Ar-CH), 111.2 (Ar-CH), 103.2 (Ar-CH), 47.0 (dd, *J* = 23.0, 22.9 Hz, *C*H_2CF_2*C*H_2), 35.8 (dd, *J* = 11.0, 8.4 Hz, C_q), 16.0 (CH_3); ^{19}F NMR (377 MHz, $CDCl_3$) δ –87.40 (d, *J* = 194.3 Hz), –89.52 (d, *J* = 194.3 Hz); HRMS (FTMS-ESI$^-$) *m/z* calcd for $C_{19}H_{15}F_2O_2^-$ [M–H]$^-$: 313.1046; found 313.1039.

4-(3,3-Difluoro-1-(pyren-1-yl)cyclobutyl)-2-methylphenol (31)

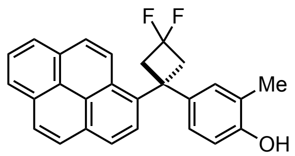

Prepared according to General Procedure C at 110 °C for 3 h using pyrene difluorocyclobutanol **8** (30.8 mg, 0.1 mmol, 1.0 equiv) and *o*-cresol (32.4 mg, 0.3 mmol, 3.0 equiv). Purification by flash column chromatography (20% Et_2O/pentane) afforded diaryl difluorocyclobutane **31** as a white solid (28.2 mg, 71%). R_f = 0.13 (30% Et_2O/pentane); mp = 194–195 °C; IR (film)/cm^{-1} 3356 (OH, br), 2924, 2857, 1610, 1507, 1472, 1412, 1375, 1233, 1161, 1121, 978, 807; ^1H NMR (400 MHz, $CDCl_3$) δ 8.26 (d, *J* = 8.0 Hz, 1H, Ar-CH), 8.18 (d, *J* = 7.7 Hz, 1H, Ar-CH), 8.13 (d, *J* = 7.7 Hz, 2H, 2 × Ar-CH), 8.10–8.03 (m, 2H, 2 × Ar-CH), 8.03–7.93 (m, 2H, 2 × Ar-CH), 7.76 (d, *J* = 9.3 Hz, 1H, Ar-CH), 7.13 (d, *J* = 2.5 Hz, 1H, Ar-CH), 7.06 (dd, *J* = 8.4, 2.5 Hz, 1H, Ar-CH), 6.63 (d, *J* = 8.4 Hz, 1H, Ar-CH), 4.58 (s, 1H, OH), 3.98–2.96 (m, 4H, 2 × CH_2), 2.15 (s, 3H, CH_3); $^{13}C\{^1H\}$ NMR (101 MHz, $CDCl_3$) δ 152.2 (Ar-C_qOH), 140.3 (*app.* d, *J* = 3.8 Hz, Ar-$C_q$$C_q$), 139.3 (app.d, *J* = 3.1 Hz, Ar-$C_q$$C_q$), 131.3 (Ar-$C_q$Ar-CH), 130.6 (Ar-$C_q$Ar-CH), 130.4 (Ar-$C_q$Ar-CH), 129.2 (Ar-CH), 128.1 (Ar-C_qAr-C_q), 127.4 (Ar-CH), 127.4 (Ar-CH), 127.3 (Ar-CH), 126.1 (Ar-CH), 125.9 (Ar-C_qAr-C_q), 125.4 (Ar-CH), 125.3 (Ar-CH), 125.1 (Ar-CH), 125.1 (Ar-CH), 124.9 (Ar-C_qMe), 124.6 (Ar-CH), 124.4 (Ar-CH), 123.8 (Ar-C_qAr-C_q), 119.5 (dd, *J* = 273.1, 272.9 Hz, CF_2), 114.7 (Ar-CH), 50.1 (dd, *J* = 22.1, 21.9 Hz, *C*H_2CF_2*C*H_2), 40.2 (dd, *J* = 13.5, 8.7 Hz, C_q), 16.0 (CH_3); ^{19}F NMR (377 MHz, $CDCl_3$) δ –85.09 (d, *J* = 195.7 Hz), –91.89 (d, *J* = 195.7 Hz); HRMS (FTMS-ESI$^-$) *m/z* calcd for $C_{27}H_{19}F_2O^-$ [M–H]$^-$: 397.1406; found 397.1406.

4-(1-(Dibenzo[*b*,*d*]thiophen-2-yl)-3,3-difluorocyclobutyl)-2-methylphenol (32)

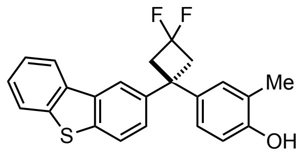

Prepared according to General Procedure C at 110 °C for 3 h using dibenzothiophene difluorocyclobutanol **7** (29.0 mg, 0.1 mmol, 1.0 equiv) and *o*-cresol (32.4 mg, 0.3 mmol, 3.0 equiv). Purification by flash column chromatography (30% Et_2O/pentane) afforded diaryl difluorocyclobutane **32** as a white solid (20.1 mg, 53%). R_f = 0.19 (30% Et_2O/pentane); mp = 213–215 °C; IR (film)/cm^{-1} 3373 (OH, br), 3056, 2008, 2953, 1507, 1468, 1429, 1412, 1299, 1254, 1233, 1120, 922, 883, 812, 732, 631; ^{1}H NMR (400 MHz, acetone-*d*$_6$) δ 8.48 (d, *J* = 2.0 Hz, 1H, Ar-CH), 8.42–8.37 (m, 1H, Ar-CH), 8.12 (br, 1H, OH), 7.99–7.92 (m, 1H, Ar-CH), 7.89 (d, *J* = 8.4 Hz, 1H, Ar-CH), 7.58–7.47 (m, 3H, 3 × Ar-CH), 7.28 (d, *J* = 2.5 Hz, 1H, Ar-CH), 7.17 (dd, *J* = 8.3, 2.5 Hz, 1H, Ar-CH), 6.77 (d, *J* = 8.3 Hz, 1H, Ar-CH), 3.65–3.40 (m, 4H, 2 × CH_2), 2.17 (s, 3H, CH_3); ^{13}C{^{1}H} NMR (126 MHz, acetone-*d*$_6$) δ 154.5 (Ar-*C*$_q$OH), 146.4 (dd, *J* = 3.11, 2.85, Ar-*C*$_q$C$_q$), 140.6 (Ar-*C*$_q$Ar-C$_q$), 139.5 (Ar-*C*$_q$C$_q$), 137.6 (Ar-*C*$_q$Ar-C$_q$), 136.4 (Ar-*C*$_q$S), 136.3 (Ar-*C*$_q$S), 129.6 (Ar-CH), 127.8 (Ar-CH), 126.8 (Ar-CH), 125.5 (Ar-CH), 125.3 (Ar-CH), 125.1 (Ar-*C*$_q$Me), 123.7 (2 × Ar-CH), 122.8 (Ar-CH), 120.2 (dd, *J* = 277.2, 275.5 Hz, CF_2), 120.0 (Ar-CH), 115.4 (Ar-CH), 48.7 (dd, *J* = 23.8, 21.7 Hz, *C*H$_2$CF$_2$*C*H$_2$) 41.1 (dd, *J* = 12.7, 10.8 Hz, C$_q$), 16.3 (CH_3); ^{19}F NMR (377 MHz, acetone-*d*$_6$) δ –87.4 (d, *J* = 192.8 Hz), –89.4 (d, *J* = 192.8 Hz); HRMS (FTMS-APCI$^-$) *m/z* calcd for $C_{23}H_{17}F_2OS^-$ [M–H]$^-$: 379.0974; found 379.0969.

4-(3,3-Difluoro-1-(4-(5-(*p*-tolyl)-3-(trifluoromethyl)-1*H*-pyrazol-1-yl)phenyl)cyclobutyl)-2-methylphenol (33)

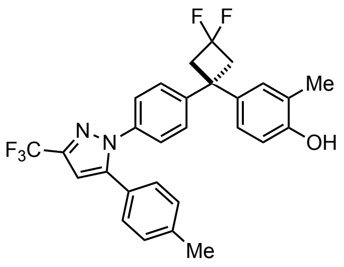

Prepared according to General Procedure C at 110 °C for 24 h using Celecoxib difluorocyclobutanol **13** (40.8 mg, 0.1 mmol, 1.0 equiv) and *o*-cresol (32.4 mg, 0.3 mmol, 3.0 equiv). Purification by flash column chromatography (30% Et_2O/pentane) afforded diaryl difluorocyclobutane **33** as a white solid (27.9 mg, 56%). R_f = 0.22 (30% Et_2O/pentane); mp = 87–89 °C; IR (film)/cm^{-1} 3356 (OH, br), 2924, 2857, 1610, 1507, 1472, 1412, 1375, 1233, 1161, 1121, 978, 807; ^{1}H NMR (400 MHz, acetone-*d*$_6$) δ 8.23 (s, 1H, OH), 7.53–7.43 (m, 2H, 2 × Ar-CH), 7.34–7.28 (m, 2H, 2 × Ar-CH), 7.17 (dd, *J* = 10.1, 2.6 Hz, 5H, 5 × Ar-CH), 7.06 (dd, *J* = 8.3, 2.6 Hz, 1H, Ar-CH), 6.91 (s, 1H, Ar-CH), 6.77 (d, *J* = 8.3 Hz, 1H, Ar-CH), 3.55–3.22 (m, 4H, 2 × CH_2), 2.32 (s, 3H, CH_3), 2.16 (s, 3H, CH_3); ^{13}C{^{1}H} NMR (101 MHz, acetone-*d*$_6$) δ 153.8 (Ar-*C*$_q$OH), 149.0 (Ar-*C*$_q$C$_q$), 145.1 (Ar-*C*$_q$Ar-C$_q$), 142.3 (q, *J* = 37.6 Hz, Ar-*C*$_q$CF$_3$), 139.1 (Ar-*C*$_q$Ar-C$_q$), 137.8 (Ar-*C*$_q$C$_q$), 137.5 (Ar-*C*$_q$N), 129.3 (2 × Ar-CH), 128.9 (Ar-CH), 128.8 (2 × Ar-CH), 127.1 (2 × Ar-CH), 126.3 (Ar-*C*$_q$Me), 125.5 (2 × Ar-CH), 124.6 (Ar-CH), 124.4 (Ar-*C*$_q$Me), 121.8 (q, *J* = 267.6 Hz, CF_3), 119.1 (dd, *J* = 276.3, 276.3 Hz, CF_2), 114.6 (Ar-CH), 105.2 (Ar-CH), 47.8 (dd, *J* = 22.7, 21.9 Hz, *C*H$_2$CF$_2$*C*H$_2$), 39.8 (dd, *J* = 13.0, 11.1 Hz, C$_q$), 20.3 (CH_3), 15.5 (CH_3); ^{19}F NMR (377 MHz, acetone-*d*$_6$) δ –62.64 (CF_3), –88.98 (d, *J* = 192.8 Hz, C*F*F), –89.64 (d, *J* = 192.8 Hz, CF*F*); HRMS (TOF-ESI$^+$) *m/z* calcd for $C_{28}H_{24}N_2F_5O^+$ [M+H]$^+$: 499.1809; found 499.1809.

4-(3,3-Difluoro-1-(4-methoxyphenyl)cyclobutyl)phenol (34)

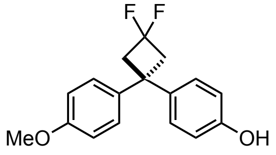

Prepared according to General Procedure C at 40 °C for 3 h using PMP difluorocyclobutanol **1** (21.4 mg, 0.1 mmol, 1.0 equiv) and phenol (28.2 mg, 0.3 mmol, 3.0 equiv). Purification by flash column chromatography (30% Et_2O/pentane) afforded diaryl difluorocyclobutane **34** as a white solid (24.5 mg, 84%). R_f = 0.31 (40% Et_2O/pentane); mp = 91–93 °C; IR (film)/cm^{-1} 3393 (OH, br), 3006, 2956, 2836, 1608, 1509, 1438, 1463, 1412, 1299, 1224, 1179, 1148, 1082, 1031, 885, 825, 605; ^{1}H NMR (400 MHz, $CDCl_3$) δ 7.19 (d, *J* = 8.8 Hz, 2H, 2 × Ar-CH), 7.14 (d, *J* = 8.6 Hz, 2H, 2 × Ar-CH), 6.87 (d, *J* = 8.8 Hz, 2H, 2 × Ar-CH), 6.79 (d, *J* = 8.6 Hz, 2H, 2 × Ar-CH), 5.07 (s, 1H, OH), 3.81 (s, 3H, OCH_3), 3.42–3.26 (m, 4H, 2 × CH_2); ^{13}C{^{1}H} NMR (101 MHz, $CDCl_3$) δ 157.8 (Ar-*C*$_q$OMe), 153.8 (Ar-*C*$_q$OH), 140.0 (Ar-*C*$_q$C$_q$), 139.8 (Ar-*C*$_q$C$_q$), 127.6 (2 × Ar-CH), 127.4 (2 × Ar-CH), 119.0 (dd, *J* = 276.9, 271.8 Hz, CF_2) 115.4 (2 × Ar-CH), 113.9 (2 × Ar-CH), 55.3 (OCH_3), 48.5 (dd, *J* = 23.5, 21.8 Hz, *C*H$_2$CF$_2$*C*H$_2$), 39.3 (dd, *J* = 10.8, 10.3 Hz, CF_2); ^{19}F NMR (377 MHz, $CDCl_3$) δ –88.84 (dd, *J* = 27.5 Hz); HRMS (FTMS-ESI$^-$) *m/z* calcd for $C_{17}H_{16}F_2O_2^-$ [M–H]$^-$: 289.1046; found 289.1046.

N.B. Trace amounts (<3%) of the ortho-alkylated product were observed in the ^{19}F NMR spectrum of the crude reaction mixture.

4-(3,3-Difluoro-1-(4-methoxyphenyl)cyclobutyl)benzene-1,2-diol (35)

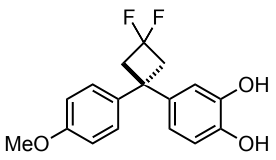

Prepared according to General Procedure C at 40 °C for 3 h using PMP difluorocyclobutanol **1** (21.4 mg, 0.1 mmol, 1.0 equiv) and catechol (33.0 mg, 0.3 mmol, 3.0 equiv). Purification by flash column chromatography (20–30% Et_2O/pentane) afforded diaryl difluorocyclobutane **35** as a clear colorless gum (25.9 mg, 85%). R_f = 0.12 (40% Et_2O/pentane); IR (film)/cm^{-1} 3826 (OH, br), 2957, 2834, 1638, 1511, 1433, 1299, 1247, 1179, 1142, 1116, 1036, 915, 870, 827, 807, 784, 735, 639; ^1H NMR (400 MHz, $CDCl_3$) δ 7.17 (d, *J* = 8.8 Hz, 2H, 2 × Ar-CH), 6.86 (d, *J* = 8.8 Hz, 2H, 2 × Ar-CH), 6.80 (d, *J* = 8.2 Hz, 1H, Ar-CH), 6.76–6.70 (m, 2H, 2 × Ar-CH), 5.18 (br, 2 × OH), 3.80 (s, 3H, OCH_3), 3.40–2.95 (m, 4H, 2 × CH_2); $^{13}C\{^1H\}$ NMR (101 MHz, $CDCl_3$) δ 157.8 (Ar-*C*qOMe), 143.5 (Ar-*C*qOH), 141.7 (Ar-*C*qOH), 141.0 (Ar-*C*qCq), 139.8 (Ar-*C*qCq), 127.4 (2 × Ar-CH), 118.9 (dd, *J* = 277.8, 276.9 Hz, CF_2), 118.8 (Ar-CH), 115.3 (Ar-CH), 113.9 (3 × Ar-CH), 55.3 (OCH_3), 48.4 (dd, *J* = 22.2, 21.6 Hz, *C*H_2CF_2*C*H_2), 39.4 (dd, *J* = 13.8, 9.9 Hz, C_q); ^{19}F NMR (377 MHz, $CDCl_3$) δ –88.44 (d, *J* = 195.5 Hz), –88.99 (d, *J* = 195.5 Hz); HRMS (FTMS-ESI⁻) *m/z* calcd for $C_{17}H_{15}F_2O_3^-$ [M–H]⁻: 305.0995; found 305.0998.

4-(3,3-Difluoro-1-(4-methoxyphenyl)cyclobutyl)benzene-1,3-diol (36)

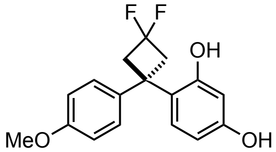

Prepared according to General Procedure C at 110 °C for 3 h using PMP difluorocyclobutanol **1** (21.4 mg, 0.1 mmol, 1.0 equiv) and resorcinol (33.0 mg, 0.3 mmol, 3.0 equiv). Purification by flash column chromatography (30–40% EtOAc/hexane) afforded diaryl difluorocyclobutane **36** as a white gum (25.2 mg, 82%). R_f = 0.30 (50% EtOAc/hexane); IR (film)/cm^{-1} 3412 (OH), 2959, 2838, 1709, 1606, 1511, 1302, 1246, 1181, 984, 909, 883, 829, 732; ^1H NMR (400 MHz, DMSO-*d6*) δ 9.28 (s, 1H, OH), 9.12 (s, 1H, OH), 7.30 (d, *J* = 8.8 Hz, 2H, 2 × Ar-CH), 7.04 (d, *J* = 9.0 Hz, 1H, Ar-CH), 6.81 (d, *J* = 8.8 Hz, 2H, 2 × Ar-CH), 6.26–6.13 (m, 2H, 2 × Ar-CH), 3.69 (s, 3H, OCH_3), 3.24 (dd, *J* = 14.9, 10.8 Hz, 4H, 2 × CH_2); $^{13}C\{^1H\}$ NMR (101 MHz, DMSO-*d6*) δ 157.5 (Ar-*C*qOMe), 157.3 (Ar-*C*qOH), 155.7 (Ar-*C*qOH), 139.6 (app. d, *J* = 2.7 Hz, Ar-*C*qCq), 128.0 (Ar-CH), 127.9 (2 × Ar-CH), 124.5 (Ar-*C*qCq), 121.0 (dd, *J* = 278.8, 276.2 Hz, CF_2), 113.6 (2 × Ar-CH), 106.3 (Ar-CH), 103.3 (Ar-CH), 55.4 (OCH_3), 47.6 (dd, *J* = 23.0, 21.7 Hz, *C*H_2CF_2*C*H_2), 37.8 (dd, *J* = 14.1, 8.1 Hz, C_q); ^{19}F NMR (377 MHz, DMSO-*d6*) δ –83.72 (d, *J* = 197.6 Hz), –90.26 (d, *J* = 197.6 Hz); HRMS (TOF-ESI⁺) *m/z* calcd for $C_{17}H_{17}F_2O_3^+$ [M+H]⁺: 307.1146; found 307.1167.

N.B. Carrying out this reaction at 40 °C resulted in di-alkylation due to the poor solubility of resorcinol in toluene.

1-(3,3-Difluoro-1-(4-methoxyphenyl)cyclobutyl)-2,4-dimethoxybenzene (37)

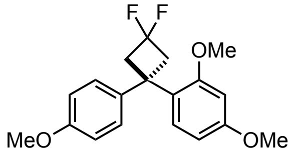

Prepared according to General Procedure C at 40 °C for 3 h using PMP difluorocyclobutanol **1** (21.4 mg, 0.1 mmol, 1.0 equiv) and 1,3-dimethoxybenzene (39 μL, 0.3 mmol, 3.0 equiv). Purification by flash column chromatography (10% Et_2O/pentane) afforded diaryl difluorocyclobutane **37** as a clear colorless gum (28.8 mg, 86%). R_f = 0.12 (10% Et_2O/pentane); IR (film)/cm^{-1} 3002, 2957, 2834, 1608, 1582, 1504, 1463, 1438, 1416, 1299, 1246, 1205, 1179, 1149, 1133, 1030, 976, 915, 881, 825, 803, 739, 691, 637, 605; ^1H NMR (400 MHz, $CDCl_3$) δ 7.31–7.24 (m, 2H, 2 × Ar-CH), 7.21 (d, *J* = 8.4 Hz, 1H, Ar-CH), 6.85–6.78 (m, 2H, 2 × Ar-CH), 6.51 (dd, *J* = 8.4, 2.4 Hz, 1H, Ar-CH), 6.42 (d, *J* = 2.4 Hz, 1H, Ar-CH), 3.81 (s, 3H, OCH_3), 3.78 (s, 3H, OCH_3), 3.73 (s, 3H, OCH_3), 3.37–3.22 (m, 4H, 2 × CH_2); $^{13}C\{^1H\}$ NMR (101 MHz, $CDCl_3$) δ 159.8 (Ar-*C*qOMe), 157.8 (Ar-*C*qOMe), 157.5 (Ar-*C*qOMe), 138.7 (app. d, *J* = 3.1 Hz, Ar-*C*qCq) , 127.9 (Ar-*C*qCq), 127.5 (2 × Ar-CH), 127.3 (Ar-CH), 119.9 (dd, *J* = 278.1, 273.6 Hz, CF_2), 113.4 (2 × Ar-CH), 103.8 (Ar-CH), 99.4 (Ar-CH), 55.4 (OCH_3), 55.2 (OCH_3), 55.1 (OCH_3), 47.7 (dd, *J* = 21.5, 21.0 Hz, *C*H_2CF_2*C*H_2), 37.7 (dd, *J* = 14.3, 8.0 Hz, C_q); ^{19}F NMR (377 MHz, $CDCl_3$) δ –85.49 (d, *J* = 186.6 Hz), –92.58 (d, *J* = 186.6 Hz); HRMS (FTMS-ESI⁺) *m/z* calcd for $C_{19}H_{21}F_2O_3^+$ [M+H]⁺: 335.1453; found 335.1453.

N.B. Carrying out this reaction at 110 °C also afforded diaryl difluorocyclobutane ***37*** *in 86% yield.*

4,4'-(3,3-Difluorocyclobutane-1,1-diyl)bis(methoxybenzene) (38) and 1-(3,3-difluoro-1-(4-methoxyphenyl)cyclobutyl)-2-methoxybenzene (38')

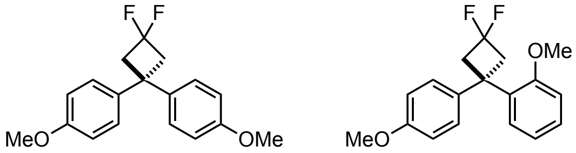

Prepared according to General Procedure C at 110 °C for 3 h using PMP difluorocyclobutanol **1** (21.4 mg, 0.1 mmol, 1.0 equiv) and anisole (33 µL, 0.3 mmol, 3.0 equiv). Purification by flash column chromatography (5–10% Et_2O/pentane) afforded diaryl difluorocyclobutane **38'** as a clear colorless gum (3.1 mg, 12%) followed by diaryl difluorocyclobutane **38** as a clear colorless gum (18.8 mg, 65%).

N.B. The observed isolated ratio of 5:1 ***38***:***38'*** *is consistent with that observed in the* ^{19}*F NMR of the crude reaction mixture.*

Diaryl difluorocyclobutane **38:** R_f = 0.37 (20% Et_2O/pentane); IR (film)/cm^{-1} 2957, 2933, 1608, 1582, 1511, 1490, 1463, 1436, 1272, 1302, 1248, 1220, 1179, 1149, 1121, 1030, 885, 825, 753; ^{1}H NMR (400 MHz, $CDCl_3$) δ 7.20 (d, *J* = 8.8 Hz, 4H, 4 × Ar-CH), 6.87 (d, *J* = 8.8 Hz, 4H, 4 × Ar-CH), 3.80 (s, 6H, 2 × OCH_3), 3.39–3.29 (m, 4H, 2 × CH_2); ^{13}C{^{1}H} NMR (101 MHz, $CDCl_3$) δ 157.9 (2 × Ar-C_qOMe), 139.8 (2 × Ar-C_qC$_q$), 127.4 (4 × Ar-CH), 119.0 (dd, *J* = 277.8, 276.7 Hz, CF_2), 113.9 (4 × Ar-CH), 55.3 (2 × OCH_3), 48.5 (dd, *J* = 24.0, 21.9 Hz, *C*H$_2$CF$_2$*C*H$_2$), 39.2 (dd, *J* = 12.3, 10.9 Hz, C$_q$); ^{19}F NMR (377 MHz, $CDCl_3$) δ –88.86; HRMS (TOF-ESI$^+$) *m/z* calcd for $C_{18}H_{19}F_2O_2^+$ [M+H]$^+$: 305.1353; found 305.1351.

Diaryl difluorocyclobutane **38':** R_f = 0.38 (20% Et_2O/pentane); IR (film)/cm^{-1} 2957, 2933, 1608, 1582, 1511, 1490, 1463, 1436, 1272, 1302, 1248, 1220, 1179, 1149, 1121, 1030, 885, 825, 753; ^{1}H NMR (400 MHz, $CDCl_3$) δ 7.37 7.26 (m, 3H, 3 × Ar-CH), 7.26–7.19 (m, 1H, Ar-CH), 7.03–6.94 (m, 1H, Ar-CH), 6.87–6.77 (m, 3H, 3 × Ar-CH), 3.78 (s, 3H, OCH_3), 3.75 (s, 3H, OCH_3), 3.42–3.22 (m, 4H, 2 × CH_2); ^{13}C{^{1}H} NMR (101 MHz, $CDCl_3$) δ 157.6 (Ar-C_qOMe), 156.8 (Ar-C_qOMe), 138.1 (Ar-C_qC$_q$), 135.3 (Ar-C_qC$_q$), 128.0 (Ar-CH), 127.6 (2 × Ar-CH), 126.8 (Ar-CH), 120.4 (Ar-CH), 119.8 (dd, *J* = 283.6, 272.4 Hz, CF_2), 113.3 (2 × Ar-CH), 111.4 (Ar-CH), 55.2 (OCH_3), 55.0 (OCH_3), 47.7 (dd, *J* = 22.4, 21.7 Hz, *C*H$_2$CF$_2$*C*H$_2$), 37.8 (dd, *J* = 15.9, 8.3 Hz, C$_q$); ^{19}F NMR (377 MHz, $CDCl_3$) δ –85.14 (d, *J* = 193.4 Hz), –93.52 (d, *J* = 193.4 Hz).

N.B: Diaryl difluorocyclobutane ***38'****did not ionize well and no tractable mass ion was found.*

5-(3,3-Difluoro-1-(4-methoxyphenyl)cyclobutyl)benzo[*d*][1,3]dioxole (39)

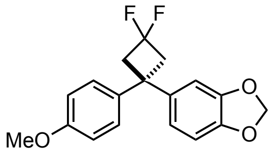

Prepared according to General Procedure C at 110 °C for 3 h using PMP difluorocyclobutanol **1** (21.4 mg, 0.1 mmol, 1.0 equiv) and 1,3-benzodioxole (35 µL, 0.3 mmol, 3.0 equiv). Purification by flash column chromatography (5–10% Et_2O/pentane) afforded diaryl difluorocyclobutane **39** as a white solid (12.7 mg, 40%). R_f = 0.19 (10% Et_2O/pentane); mp = 107–108 °C; IR (film)/cm^{-1} 3004, 2957, 2901, 2836, 1608, 1505, 1485, 1435, 1302, 1243, 1181, 1166, 1036, 933, 805, 736, 631, 551; ^{1}H NMR (400 MHz, $CDCl_3$) δ 7.19 (d, *J* = 8.8 Hz, 2H, 2 × Ar-CH), 6.86 (d, *J* = 8.8 Hz, 2H, 2 × Ar-CH), 6.78–6.70 (m, 3H, 3 × Ar-CH), 5.94 (s, 2H, OCH_2), 3.80 (s, 3H, OCH_3), 3.36–3.25 (m, 4H, 2 × CH_2); ^{13}C{^{1}H} NMR (101 MHz, $CDCl_3$) δ 157.9 (Ar-C_qOMe), 147.9 (Ar-C_qOCH$_2$), 145.9 (Ar-C_qOCH$_2$), 141.7 (Ar-C_qC$_q$), 139.5 (Ar-C_qC$_q$), 127.3 (2 × Ar-CH), 119.1 (Ar-CH), 118.8 (dd, *J* = 277.2, 274.8 Hz, CF_2), 116.2 (2 × Ar-CH), 108.0 (Ar-CH), 107.2 (Ar-CH), 101.1 (OCH_2), 55.3 (OCH_3), 48.4 (dd, *J* = 22.0, 21.8 Hz, *C*H$_2$CF$_2$*C*H$_2$), 39.8 (dd, *J* = 10.5, 9.5 Hz, C$_q$); ^{19}F NMR (377 MHz, $CDCl_3$) δ –88.78 (dd, *J* = 194.4 Hz), –89.21 (dd, *J* = 194.4 Hz).

N.B. Diaryl difluorocyclobutane ***39*** *did not ionize well and no tractable mass ion was found.*

3-(3,3-Difluoro-1-(4-methoxyphenyl)cyclobutyl)-1-methyl-1*H*-indole (40)

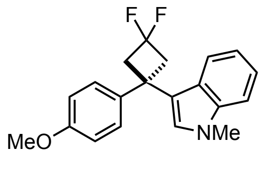

Prepared according to General Procedure C at 110 °C for 24 h using PMP difluorocyclobutanol **1** (21.4 mg, 0.1 mmol, 1.0 equiv) and 1-methylindole (38 µL, 0.3 mmol, 3.0 equiv). Purification by flash column chromatography (10% Et_2O/pentane) afforded diaryl difluorocyclobutane **40** as a white solid (21.4 mg, 65%). R_f = 0.31 (20% Et_2O/pentane); mp = 120–121 °C; IR (film)/cm^{-1} 3049, 3004, 2959, 2834, 1610, 1511, 1466, 1442, 1375, 1328, 1295, 1246, 1228, 1179, 1155,

1094, 1034, 825, 739, 584. 533; ^{1}H NMR (400 MHz, $CDCl_3$) δ 7.42–7.28 (m, 4H, 4 × Ar-CH), 7.23 (ddd, *J* = 8.3, 6.9, 1.1 Hz, 1H, Ar-CH), 7.05 (ddd, *J* = 8.0, 6.9, 1.1 Hz, 1H, Ar-CH), 6.86 (d, *J* = 8.2 Hz, 3H, 3 × Ar-CH), 3.80 (s, 3H, OCH_3), 3.79 (s, 3H, OCH_3), 3.52–3.26 (m, 4H, 2 × CH_2); $^{13}C\{^{1}H\}$ NMR (101 MHz, $CDCl_3$) δ 157.8 (Ar-*C*$_q$OMe), 139.0 (app. d, *J* = 3.7 Hz, Ar-*C*$_q$C$_q$), 137.9 (Ar-*C*$_q$Ar-CH), 127.6 (2 × Ar-CH), 126.5 (Ar-CH), 125.9 (Ar-*C*$_q$Ar-CH), 122.4 (Ar-CH), 121.8 (Ar-CH), 121.0 (app. d, *J* = 3.5 Hz, Ar-*C*$_q$C$_q$), 120.1 (Ar-CH), 119.6 (dd, *J* = 280.5, 277.8 Hz, CF_2) 119.0 (Ar-CH), 113.7 (2 × Ar-CH), 109.6 (Ar-CH), 55.3 (OCH_3), 48.5 (dd, *J* = 23.3, 21.8 Hz, *C*H_2CF_2*C*H_2), 34.3 (dd, *J* = 12.4, 10.6 Hz, C$_q$), 32.8 (NMe); ^{19}F NMR (377 MHz, $CDCl_3$) δ –86.38 (d, *J* = 170.2 Hz), –88.43 (d, *J* = 170.2 Hz); HRMS (TOF-ESI$^+$) *m/z* calcd for $C_{20}H_{20}NF_2O^+$ [M+H]$^+$: 328.1513; found 328.1506.

2-(3,3-Difluoro-1-(4-methoxyphenyl)cyclobutyl)-5-methylfuran (41)

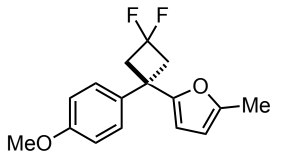

Prepared according to General Procedure C at 40 °C for 3 h using PMP difluorocyclobutanol **1** (21.4 mg, 0.1 mmol, 1.0 equiv) and 2-methylfuran (27 µL, 0.3 mmol, 3.0 equiv). Purification by flash column chromatography (5% Et_2O/pentane) afforded diaryl difluorocyclobutane **41** as a clear colorless oil (16.7 mg, 60%). R$_f$ = 0.59 (5% Et_2O/pentane); IR (film)/cm^{-1} 3006, 2957, 2924, 2838, 1612, 1513, 1464, 1442, 1412, 1300, 1250, 1178, 1157, 1090, 1034, 829, 784; ^{1}H NMR (400 MHz, $CDCl_3$) δ 7.22 (d, *J* = 8.7 Hz, 2H, 2 × Ar-CH), 6.91 (d, *J* = 8.7 Hz, 2H, 2 × Ar-CH), 5.87–5.81 (m, 1H, Ar-CH), 5.72 (d, *J* = 3.1 Hz, 1H, Ar-CH), 3.83 (s, 3H, OCH_3), 3.43–3.29 (m, 2H, CH_2), 3.25–3.07 (m, 2H, CH_2), 2.27 (s, 3H, CH_3); $^{13}C\{^{1}H\}$ NMR (101 MHz, $CDCl_3$) δ 158.3 (Ar-*C*$_q$OMe), 156.9 (Ar-*C*$_q$C$_q$), 151.8 (Ar-*C*$_q$Me), 136.2 (Ar-*C*$_q$C$_q$), 127.9 (2 × Ar-CH), 118.8 (dd, *J* = 274.9, 272.5 Hz, CF_2), 113.8 (2 × Ar-CH), 106.9 (Ar-CH), 106.0 (Ar-CH), 55.3 (OCH_3), 47.1 (dd, *J* = 23.2, 22.5 Hz, *C*H_2CF_2*C*H_2), 35.4 (dd, *J* = 11.6, 9.8 Hz, C$_q$), 13.6 (CH_3); ^{19}F NMR (377 MHz, $CDCl_3$) δ –87.89 (d, *J* = 194.6 Hz), -89.49 (d, *J* = 194.6 Hz); HRMS (TOF-ESI$^+$) *m/z* calcd for $C_{16}H_{17}F_2O_2^+$ [M+H]$^+$: 279.1197; found 279.1184.

*N.B. Carrying out this reaction at 110 °C also afforded diaryl difluorocyclobutane **41** in 60% yield.*

(4-(3,3-Difluoro-1-(5-methylfuran-2-yl)cyclobutyl)phenoxy)triisopropylsilane (42)

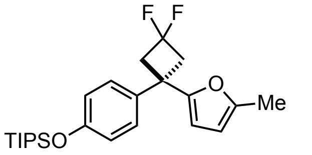

Prepared according to General Procedure C at 110 °C for 3 h using *p*-OTIPS difluorocyclobutanol **4** (35.7 mg, 0.1 mmol, 1.0 equiv) and 2-methylfuran (27 µL, 0.3 mmol, 3.0 equiv). Purification by flash column chromatography (20% CH_2Cl_2/hexane) afforded diaryl difluorocyclobutane **42** as a clear colorless oil (27.3 mg, 65%). R$_f$ = 0.33 (20% CH_2Cl_2/hexane); IR (film)/cm^{-1} 2944, 2866, 1608, 1509, 1299, 1265, 1233, 1155, 1088, 911, 881, 833, 781, 682; ^{1}H NMR (400 MHz, $CDCl_3$) δ 7.14 (d, *J* = 8.7 Hz, 2H, 2 × Ar-CH), 6.88 (d, *J* = 8.7 Hz, 2H, 2 × Ar-CH), 5.87–5.80 (m, 1H, Ar-CH), 5.68 (d, *J* = 3.1 Hz, 1H, Ar-CH), 3.41–3.26 (m, 2H, CH_2), 3.26–3.07 (m, 2H, CH_2), 2.28 (s, 3H, CH_3), 1.34–1.22 (m, 3H, 3 × CH), 1.13 (d, *J* = 7.3 Hz, 18H, 6 × CH_3); $^{13}C\{^{1}H\}$ NMR (101 MHz, $CDCl_3$) δ 157.0 (app. d, *J* = 3.2 Hz, Ar-*C*$_q$C$_q$), 154.8 (Ar-*C*$_q$OTIPS), 151.7 (Ar-*C*$_q$Me), 136.5 (app. d, *J* = 3.7 Hz, Ar-*C*$_q$C$_q$), 127.8 (2 × Ar-CH), 119.7 (2 × Ar-CH), 118.8 (dd, *J* = 276.1, 267.2 Hz, CF_2), 107.0 (Ar-CH), 106.0 (Ar-CH), 47.2 (dd, *J* = 23.0, 22.6 Hz, *C*H_2CF_2*C*H_2), 35.4 (dd, *J* = 12.2, 10.9 Hz, C$_q$), 17.9 (6 × CH_3), 13.6 (CH_3), 12.7 (3 × CH); ^{19}F NMR (377 MHz, $CDCl_3$) δ –87.89 (d, *J* = 194.4 Hz), –89.57 (d, *J* = 194.4 Hz); HRMS (TOF-ESI$^+$) *m/z* calcd for $C_{24}H_{35}F_2O_2Si^+$ [M+H]$^+$: 421.2374; found 421.2354.

(4a*R*,9a*R*)-5-(3,3-Difluoro-1-(4-((triisopropylsilyl)oxy)phenyl)cyclobutyl)-1,1,4a,6-tetramethyl-2,3,4,4a,9,9a-hexahydro-1*H*-xanthen-8-ol (43)

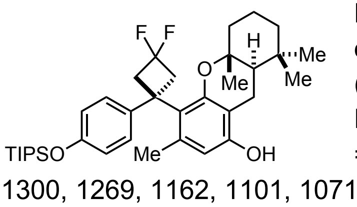

Prepared according to General Procedure C at 110 °C for 3 h using *p*-OTIPS difluorocyclobutanol **4** (35.7 mg, 0.1 mmol, 1.0 equiv) and meroterpenoid analogue **S5** (70.0 mg, 0.27 mmol, 2.7 equiv). Purification by flash column chromatography (10–20% Et_2O/pentane) afforded diaryl difluorocyclobutane **43** as an orange oil (24.8 mg, 42%). R$_f$ = 0.24 (20% Et_2O/pentane); IR (film)/cm^{-1} 3390 (OH, br), 2942, 2866, 1606, 1578, 1507, 1300, 1269, 1162, 1101, 1071, 911, 684; ^{1}H NMR (400 MHz, acetone-*d$_6$*) δ 8.06 (s, 1H, OH), 7.44 (d, *J* = 8.7 Hz, 2H, 2 × Ar-CH), 6.82 (d, *J* = 8.7 Hz, 2H, 2 × Ar-CH), 6.23 (s, 1H, Ar-CH), 3.43–3.18 (m, 4H, 2 × CH_2), 2.75 (dd, *J* = 16.8, 5.0 Hz, 1H, CH*H*), 2.33 (dd, *J* = 16.8, 13.4 Hz, 1H, C*H*H), 2.11 (s, 3H, CH_3), 2.02–1.96 (m, 1H, CH*H*), 1.63 (ddd, *J* = 18.6, 7.6, 4.9 Hz, 4H, C*H*H + CH_2 + CH), 1.55–1.49 (m, 1H, CH*H*), 1.43–1.34 (m, 1H, C*H*H), 1.32–1.23 (m, 3H, 3 × CH), 1.17 (s, 3H, CH_3), 1.10 (d, *J* = 7.3 Hz, 18H, 6 × CH_3), 1.04 (s, 3H, CH_3), 0.96 (s, 3H, CH_3); $^{13}C\{^{1}H\}$ NMR (126 MHz, acetone-*d$_6$*) δ 154.8 (Ar-*C*$_q$OC$_q$), 154.5 (Ar-*C*$_q$OTIPS), 152.1 (Ar-*C*$_q$OH), 140.1 (app. d, *J* = 3.6 Hz, Ar-*C*$_q$C$_q$) 134.9 (Ar-*C*$_q$Me), 128.6

(2 × Ar-CH), 126.2 (Ar-*C*$_q$C$_q$), 121.7 (dd, *J* = 270.8, 268.2 Hz, CF_2) 119.8 (2 × Ar-CH), 109.9 (Ar-CH), 108.8 (Ar-*C*$_q$CH$_2$), 77.8 (OC$_q$), 50.7 (dd, *J* = 24.4, 20.7 Hz, *C*H$_2$CF$_2$CH$_2$), 50.4 (dd, *J* = 24.4, 20.7 Hz, CH$_2$CF$_2$*C*H$_2$), 48.4 (CH), 42.2 (CH_2), 40.8 (CH_2), 39.1 (dd, *J* = 19.3, 3.9 Hz, C$_q$), 33.9 (C$_q$), 32.3 (CH_3), 21.3 (CH_3), 20.9 (CH_3), 20.3 (CH_2), 19.7 (CH_2), 18.9 (CH_3), 18.2 (6 × CH_3), 13.3 (3 × CH); ^{19}F NMR (377 MHz, acetone-*d*$_6$) δ –81.50 (d, *J* = 194.1 Hz), –100.37 (d, *J* = 194.1 Hz); HRMS (TOF-ESI$^+$) *m/z* calcd for $C_{36}H_{53}F_2O_3Si^+$ [M+H]$^+$: 599.3732; found 599.3716.

(8*R*,9*S*,13*S*,14*S*)-2-(3,3-Difluoro-1-(4-((triisopropylsilyl)oxy)phenyl)cyclobutyl)-3-hydroxy-13-methyl-6,7,8,9,11,12,13,14,15,16-decahydro-17*H*-cyclopenta[*a*]phenanthren-17-one (44)

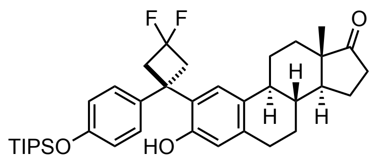

Prepared according to General Procedure C at 110 °C for 3 h using *p*-OTIPS difluorocyclobutanol **4** (35.7 mg, 0.1 mmol, 1.0 equiv) and estrone (81.1 mg, 0.3 mmol, 3.0 equiv). Purification by flash column chromatography (50% Et_2O/pentane) afforded diaryl difluorocyclobutane **44** as a white solid (33.8 mg, 56%). R$_f$ = 0.40 (50% Et_2O/pentane); mp = 171–176 °C; IR (film)/cm^{-1} 3431 (OH, br), 2942, 2865, 1723, 1507, 1418, 1300, 1265, 1197, 915, 883; ^{1}H NMR (400 MHz, $CDCl_3$) δ 7.21 (d, *J* = 8.7 Hz, 3H, 3 × Ar-CH), 6.80 (d, *J* = 8.6 Hz, 2H, 2 × Ar-CH), 6.48 (s, 1H, Ar-CH), 4.50 (s, 1H, OH), 3.43–3.18 (m, 4H, 2 × CH_2), 2.90–2.79 (m, 2H, CH_2), 2.59–2.43 (m, 2H, CH_2), 2.35–2.25 (m, 1H, CH), 2.23–1.95 (m, 4H, 2 × CH_2), 1.73–1.62 (m, 2H, CH_2), 1.58–1.50 (m, 2H, CH_2), 1.48–1.38 (m, 2H, 2 × CH), 1.24 (qd, *J* = 7.0, 2.2 Hz, 3H, 3 × CH), 1.09 (d, *J* = 7.3 Hz, 18H, 6 × CH_3), 0.96 (s, 3H, CH_3); ^{13}C{^{1}H} NMR (126 MHz, $CDCl_3$) δ 221.1 (C=O), 154.5 (Ar-*C*$_q$OTIPS), 151.0 (Ar-*C*$_q$OH), 137.9 (app.d, *J* = 3.8 Hz, Ar-*C*$_q$C$_q$), 136.5 (Ar-*C*$_q$CH), 131.9 (Ar-*C*$_q$CH$_2$), 130.6 (app.d, *J* = 3.8 Hz, Ar-*C*$_q$C$_q$), 127.4 (2 × Ar-CH), 124.3 (Ar-CH), 119.6 (2 × Ar-CH), 119.5 (dd, *J* = 276.8, 273.7 Hz, CF_2), 116.8 (Ar-CH), 50.4 (CH), 48.0 (C$_q$), 47.7 (dd, *J* = 22.0, 21.3 Hz, *C*H$_2$CF$_2$*C*H$_2$), 44.1 (CH), 38.3 (CH), 37.7 (dd, *J* = 13.6, 8.8 Hz, C$_q$), 35.9 (C=O*C*H$_2$), 31.6 (CH_2), 29.0 (CH_2), 26.4 (CH_2), 26.1 (CH_2), 21.6 (CH_2), 17.9 (6 × CH_3), 13.9 (CH_3), 12.6 (3 × CH); ^{19}F NMR (377 MHz, $CDCl_3$) δ –85.94 (d, *J* = 193.6 Hz), –91.50 (d, *J* = 193.6 Hz); HRMS (FTMS-ESI$^-$) *m/z* calcd for $C_{37}H_{49}F_2O_3Si^-$ [M–H]$^-$: 607.3425; found 607.3425.

*N.B. Diaryl difluorocyclobutane **44** was further characterized by single crystal x-ray diffraction.*

Aryl Sulfanyl Difluorocyclobutanes 45–58

4-(1-((4-Bromophenyl)thio)-3,3-difluorocyclobutyl)phenol (45)

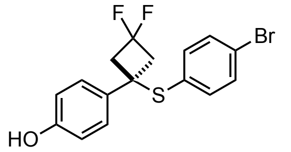

Prepared according to General Procedure C at 110 °C for 3 h using *p*-OH difluorocyclobutanol **4** (20.0 mg, 0.1 mmol, 1.0 equiv) and 4-bromothiophenol (56.7 mg, 0.3 mmol, 3.0 equiv). Purification by flash column chromatography (20–30% Et_2O/pentane) afforded aryl sulfanyl difluorocyclobutane **45** as an off-white solid (35.3 mg, 95%). R$_f$ = 0.20 (20% Et_2O/pentane); mp = 95–96 °C; IR (film)/cm^{-1} 3387 (OH), 2946, 1511, 1370, 1299, 1237, 1172, 1157, 1103, 1008, 818; ^{1}H NMR (400 MHz, acetone-*d*$_6$) δ 8.45 (s, 1H, OH), 7.47 (d, *J* = 8.4 Hz, 2H, 2 × Ar-CH), 7.09 (d, *J* = 8.4 Hz, 2H, 2 × Ar-CH), 6.99 (d, *J* = 8.6 Hz, 2H, 2 × Ar-CH), 6.76 (d, *J* = 8.6 Hz, 2H, 2 × Ar-CH), 3.36–3.10 (m, 4H, 2 × CH_2); ^{13}C{^{1}H} NMR (101 MHz, acetone-*d*$_6$) δ 156.4 (Ar-*C*$_q$OH), 137.4 (2 × Ar-CH), 134.8 (app. d, *J* = 3.2 Hz, Ar-*C*$_q$C$_q$), 131.9 (Ar-*C*$_q$S), 131.8 (2 × Ar-CH), 128.2 (2 × Ar-CH), 123.2 (Ar-*C*$_q$Br), 118.5 (dd, *J* = 278.2, 276.6 Hz, CF_2), 114.8 (2 × Ar-CH), 48.4 (dd, *J* = 25.0, 22.6 Hz, *C*H$_2$CF$_2$*C*H$_2$), 44.3 (dd, *J* = 13.8, 10.8 Hz, C$_q$); ^{19}F NMR (377 MHz, acetone-*d*$_6$) δ –87.44 (d, *J* = 194.7 Hz), –89.48 (d, *J* = 194.7 Hz); HRMS (FTMS-ESI$^-$) *m/z* calcd for $C_{16}H_{12}F_2OS^{79}Br^-$ [M–H]$^-$: 368.9766; found 368.9772.

(4-Bromophenyl)(3,3-difluoro-1-(4-methoxyphenyl)cyclobutyl)sulfane (46)

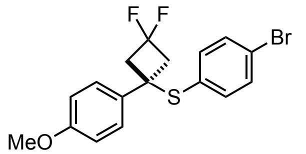

Prepared according to General Procedure C at 40 °C for 3 h using PMP difluorocyclobutanol **1** (21.4 mg, 0.1 mmol, 1.0 equiv) and 4-bromothiophenol (56.7 mg, 0.3 mmol, 3.0 equiv). Purification by flash column chromatography (10–20% CH_2Cl_2/hexane) afforded aryl sulfanyl difluorocyclobutane **46** as a white solid (36.6 mg, 95%). R$_f$ = 0.26 (20% CH_2Cl_2/hexane); mp = 65–67 °C; IR (film)/cm^{-1} 3004, 2953, 2836, 1610, 1513, 1472, 1300, 1250, 1161, 1105, 1090, 1034, 1010, 820; ^{1}H NMR (400 MHz, $CDCl_3$) δ 7.36 (d, *J* = 8.4 Hz, 2H, 2 × Ar-CH), 6.98–6.91 (m, 4H, 4 × Ar-CH), 6.82 (d, *J* = 8.8 Hz, 2H, 2 × Ar-CH), 3.83 (s, 3H, OCH_3), 3.22–3.07 (m, 4H, 2 × CH_2); ^{13}C{^{1}H} NMR (101 MHz, $CDCl_3$) δ 158.5 (Ar-*C*$_q$OMe), 137.5 (2 × Ar-CH), 136.2 (app. d, *J* = 4.9 Hz, Ar-*C*$_q$C$_q$), 131.9 (2 × Ar-CH), 131.3 (Ar-*C*$_q$S), 127.9 (2 × Ar-CH),

124.0 (Ar-*C_q*Br), 118.0 (dd, *J* = 278.1, 276.7 Hz, CF_2), 113.5 (2 × Ar-CH), 55.3 (OCH_3), 48.5 (dd, 23.3, 22.7 Hz, *C*$H_2$$CF_2$*C*$H_2$), 44.4 (dd, *J* =13.6, 9.1 Hz, C_q); ^{19}F NMR (377 MHz, $CDCl_3$) δ –86.9 (d, *J* = 192.1 Hz), 89.7 (d, *J* = 192.1 Hz).

*N.B. Carrying out this reaction on a 0.3 mmol scale with 2.0 equiv of 4-bromothiophenol afforded aryl sulfanyl difluorocyclobutane **46** in 93% yield. Difluorocyclobutane sulfide **46** did not ionize well, and no tractable mass ion was found. Aryl sulfanyl difluorocyclobutane **46** was further characterized by single crystal x-ray diffraction.*

(4-(1-((4-Bromophenyl)thio)-3,3-difluorocyclobutyl)phenoxy)triisopropylsilane (47)

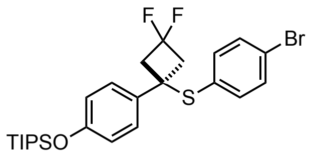

Prepared according to General Procedure C at 110 °C for 3 h using *p*-OTIPS difluorocyclobutanol **4** (35.7 mg, 0.1 mmol, 1.0 equiv) and 4-bromothiophenol (56.7 mg, 0.3 mmol, 3.0 equiv). Purification by flash column chromatography (0–10% CH_2Cl_2/hexane) afforded aryl sulfanyl difluorocyclobutane **47** as a clear colorless oil (44.2 mg, 84%). R_f = 0.24 (10% CH_2Cl_2/hexane); IR (film)/cm^{-1} 2947, 2864, 1604, 1507, 1470, 1299, 1265, 1172, 1161, 1010, 909, 881, 818, 684; ^{1}H NMR (400 MHz, $CDCl_3$) δ 7.32 (d, *J* = 8.4 Hz, 2H, 2 × Ar-CH), 6.91 (d, *J* = 8.4 Hz, 2H, 2 × Ar-CH), 6.83 (d, *J* = 8.7 Hz, 2H, 2 × Ar-CH), 6.78 (d, *J* = 8.7 Hz, 2H, 2 × Ar-CH), 3.24–3.07 (m, 4H, 2 × CH_2), 1.32–1.23 (m, 3H, 3 × CH), 1.13 (d, *J* = 7.3 Hz, 18H, 6 × CH_3); ^{13}C{^{1}H} NMR (101 MHz, $CDCl_3$) δ 155.1 (Ar-*C_q*OTIPS), 137.6 (2 × Ar-CH), 136.5 (app. d, *J* =5.2 Hz, Ar-*C_q*C_q), 131.8 (2 × Ar-CH), 131.2 (Ar-*C_q*S), 127.8 (2 × Ar-CH), 124.0 (Ar-*C_q*Br), 119.5 (2 × Ar-CH), 118.1 (dd, *J* = 277.7, 272.5 Hz, CF_2) 48.5 (dd, *J* = 22.7, 22.3 Hz, *C*$H_2$$CF_2$*C*$H_2$), 44.6 (dd, J = 15.4, 8.7 Hz, C_q*)* 17.9 (6 × CH_3), 12.7 (3 × CH); ^{19}F NMR (377 MHz, $CDCl_3$) δ –85.66 (d, *J* = 195.2 Hz), –90.01 (d, *J* =195.2 Hz); HRMS (FTMS-APCI$^-$) *m/z* calcd for $C_{25}H_{32}F_2OSSi^{79}Br^-$ [M–H]$^-$: 525.1100; found 525.1094.

(4-Bromophenyl)(1-(4-chlorophenyl)-3,3-difluorocyclobutyl)sulfane (48)

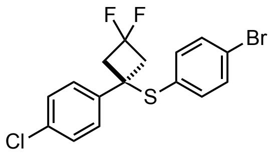

Prepared according to General Procedure C at 110 °C for 3 h using *p*-Cl difluorocyclobutanol **11** (21.8 mg, 0.1 mmol, 1.0 equiv) and 4-bromothiophenol (56.7 mg, 0.3 mmol, 3.0 equiv). Purification by flash column chromatography (5–10% CH_2Cl_2/hexane) afforded aryl sulfanyl difluorocyclobutane **48** as an off-white solid (24.5 mg, 63%). R_f = 0.12 (10% CH_2Cl_2/hexane); mp = 64–65 °C; IR (film)/cm^{-1} 2924, 2854, 1492, 1472, 1403, 1302, 1243, 1161, 1103, 1010, 820, 535; ^{1}H NMR (400 MHz, $CDCl_3$) δ 7.38 (d, *J* = 8.4 Hz, 2H, 2 × Ar-CH), 7.31–7.25 (m, 2H, 2 × Ar-CH), 6.97–6.88 (m, 4H, 4 × Ar-CH), 3.29–2.99 (m, 4H, 2 × CH_2); ^{13}C{^{1}H} NMR (101 MHz, $CDCl_3$) δ 142.8 (Ar-*C_q*C_q), 137.5 (2 × Ar-CH), 133.0 (Ar-*C_q*S), 132.1 (2 × Ar-CH), 130.7 (Ar-*C_q*Cl), 128.4 (2 × Ar-CH), 128.0 (2 × Ar-CH), 124.4 (Ar-*C_q*Br), 117.7 (dd, *J* = 277.9, 277.2 Hz, CF_2), 48.3 (dd, *J* = 23.0, 19.9 Hz, *C*$H_2$$CF_2$*C*$H_2$), 44.2 (dd, *J* = 15.4, 9.4 Hz, C_q); ^{19}F NMR (377 MHz, $CDCl_3$) δ –85.92 (d, *J* = 195.9 Hz), –90.07 (d, *J* = 195.9 Hz); HRMS (FTMS-APCI$^-$) *m/z* calcd for $C_{16}H_{14}FOS^{35}Cl^{79}Br^-$ [M–F+H_2O]$^-$: 386.9627; found 386.9611.

(4-(1-((4-Bromophenyl)thio)-3,3-difluorocyclobutyl)phenoxy)triisopropylsilane (49)

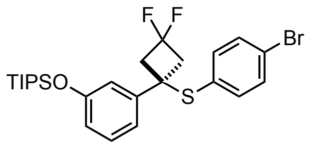

Prepared according to General Procedure C at 110 °C for 3 h using *m*-OTIPS difluorocyclobutanol **4** (35.7 mg, 0.1 mmol, 1.0 equiv) and 4-bromothiophenol (56.7 mg, 0.3 mmol, 3.0 equiv). Purification by flash column chromatography (0–10% CH_2Cl_2/hexane) afforded aryl sulfanyl difluorocyclobutane **49** as a clear colorless oil (21.4 mg, 41%). R_f = 0.29 (10% CH_2Cl_2/hexane); IR (film)/cm^{-1} 2944, 2866, 1599, 1582, 1485, 1472, 1302, 1250, 1215, 1010, 933, 881, 700; ^{1}H NMR (400 MHz, $CDCl_3$) δ 7.34 (d, *J* = 8.4 Hz, 2H, 2 × Ar-CH), 7.12 (t, *J* = 7.8 Hz, 1H, Ar-CH), 6.95 (d, *J* = 8.4 Hz, 2H, 2 × Ar-CH), 6.77–6.73 (m, 1H, Ar-CH), 6.58–6.52 (m, 2H, 2 × Ar-CH), 3.22–3.10 (m, 4H, 2 × CH_2), 1.29–1.16 (m, 3H, 3 × CH), 1.10 (d, *J* = 7.2 Hz, 18H, 6 × CH_3); ^{13}C{^{1}H} NMR (126 MHz, $CDCl_3$) δ 155.9 (Ar-*C_q*OTIPS), 145.6 (dd, *J* = 5.08, 1.71 Hz, Ar-*C_q*C_q), 137.1 (2 × Ar-CH), 131.8 (2 × Ar-CH), 131.1 (Ar-*C_q*S), 129.0 (Ar-CH), 123.9 (Ar-*C_q*Br), 119.2 (Ar-CH), 118.5 (Ar-CH), 118.3 (Ar-CH), 118.0 (dd, *J* = 280.4, 278.0 Hz, CF_2), 48.4 (dd, *J* = 22.8, 21.9 Hz, *C*$H_2$$CF_2$*C*$H_2$), 44.5 (dd, *J* = 15.7, 8.7 Hz, C_q), 17.9 (6 × CH_3), 12.6 (3 × CH); ^{19}F NMR (377 MHz, $CDCl_3$) δ –85.71 (d, *J* = 195.3 Hz), –90.26 (d, *J* = 195.3 Hz); HRMS (FTMS-ESI$^-$) *m/z* calcd for $C_{25}H_{32}F_2OSSi^{79}Br^-$ [M–H]$^-$: 525.1100; found 525.1102.

(4-Bromophenyl)(1-(8,10-dihydropyren-1-yl)-3,3-difluorocyclobutyl)sulfane (50)

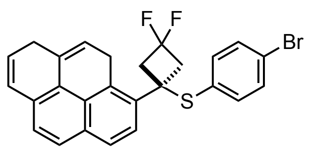

Prepared according to General Procedure C at 110 °C for 3 h using pyrene difluorocyclobutanol **8** (30.8 mg, 0.1 mmol, 1.0 equiv) and 4-bromothiophenol (56.7 mg, 0.3 mmol, 3.0 equiv). Purification by flash column chromatography (0–20% CH_2Cl_2/hexane) afforded aryl sulfanyl difluorocyclobutane **50** as an off-white solid (43.6 mg, 91%). R_f = 0.31 (20% CH_2Cl_2/hexane); mp = 144–146 °C; IR (film)/cm^{-1} 3041, 2946, 2926, 2868, 1302, 1246, 1198, 1177, 1121, 1086, 1008, 907, 844, 818, 730, 684; ^{1}H NMR (400 MHz, $CDCl_3$) δ 8.30–8.17 (m, 5H, 5 × Ar-CH), 8.14–8.02 (m, 3H, 3 × Ar-CH), 7.93 (d, *J* = 8.0 Hz, 1H, Ar-CH), 7.17 (dd, *J* = 16.1, 8.2 Hz, 3H, 3 × Ar-CH), 6.71 (d, *J* = 8.4 Hz, 2H, 2 × Ar-CH), 3.74–3.41 (br, 4H, 2 × CH_2); ^{13}C{^{1}H} NMR (101 MHz, $CDCl_3$) δ 138.2 (2 × Ar-CH), 136.7 (dd, *J* = 5.0, 2.0 Hz, Ar-*C*$_q$C$_q$), 131.8 (2 × Ar-CH), 131.3 (Ar-*C*$_q$S), 131.0 (Ar-*C*$_q$Ar-C$_q$), 130.9 (Ar-*C*$_q$Ar-CH), 130.5 (Ar-*C*$_q$Ar-CH), 127.8 (Ar-CH), 127.5 (Ar-*C*$_q$Ar-CH), 127.5 (Ar-CH), 127.3 (Ar-CH), 126.3 (Ar-CH), 125.7 (Ar-CH), 125.7 (Ar-CH), 125.6 (Ar-*C*$_q$Ar-C$_q$), 125.5 (Ar-CH), 124.9 (Ar-*C*$_q$Ar-CH), 124.4 (Ar-*C*$_q$Br), 124.2 (Ar-CH), 123.6 (Ar-CH), 118.4 (dd, *J* = 278.7, 273.5 Hz, CF_2), 50.7 (br, *C*$H_2CF_2CH_2$), 49.1 (br, CH_2CF_2*C*H_2), 45.8 (dd, *J* = 18.1, 6.1 Hz, C$_q$); ^{19}F NMR (377 MHz, $CDCl_3$) δ –83.33 (d, *J* = 196.4 Hz), –92.34 (d, *J* = 196.4 Hz); HRMS (FTMS-APCI$^+$) *m/z* calcd for $C_{26}H_{18}F_2S^{79}Br^+$ [M]$^+$: 479.0275; found 479.0258.

1-(4-(1-((4-Bromophenyl)thio)-3,3-difluorocyclobutyl)phenyl)-5-(*p*-tolyl)-3-(trifluoromethyl)-1*H*-pyrazole (51)

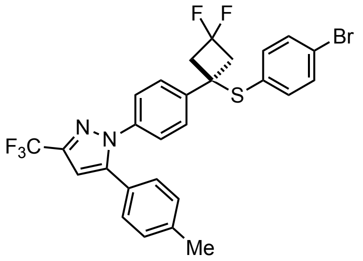

Prepared according to General Procedure C at 110 °C for 3 h using Celecoxib difluorocyclobutanol **13** (40.8 mg, 0.1 mmol, 1.0 equiv) and 4-bromothiophenol (56.7 mg, 0.3 mmol, 3.0 equiv). Purification by flash column chromatography (40% CH_2Cl_2/hexane) afforded aryl sulfanyl difluorocyclobutane **51** as a white foam (51.0 mg, 91%). R_f = 0.21 (40% CH_2Cl_2/hexane); mp = 60–63 °C; IR (film)/cm^{-1} 3021, 2946, 2924, 1507, 1470, 1302, 1233, 1157, 1067, 1008, 971, 805; ^{1}H NMR (400 MHz, acetone-*d*$_6$) δ 7.50–7.44 (m, 2H, 2 × Ar-CH), 7.32–7.26 (m, 6H, 6 × Ar-CH), 7.17 (d, *J* = 8.6 Hz, 2H, 2 × Ar-CH), 7.08 (d, *J* = 8.4 Hz, 2H, 2 × Ar-CH), 6.96 (s, 1H, Ar-CH), 3.41–3.20 (m, 4H, 2 × CH_2), 2.42 (s, 3H, CH_3); ^{13}C{^{1}H} NMR (101 MHz, acetone-*d*$_6$) δ 145.2 (Ar-*C*$_q$Ar-C$_q$), 144.7 (dd, *J* = 4.29, 1.50, Hz, Ar-*C*$_q$C$_q$) 142.5 (q, *J* = 38.1 Hz, Ar-*C*$_q$$CF_3$), 139.2 (Ar-*C*$_q$N), 138.2 (Ar-*C*$_q$Ar-C$_q$), 137.7 (2 × Ar-CH), 132.1 (2 × Ar-CH), 131.1 (Ar-*C*$_q$S), 129.4 (2 × Ar-CH), 128.9 (2 × Ar-CH), 127.7 (2 × Ar-CH), 126.4 (Ar-*C*$_q$Me), 125.2 (2 × Ar-CH), 123.8 (Ar-*C*$_q$Br), 121.7 (q, *J* = 274.2 Hz, CF_3), 118.2 (dd, *J* = 278.7 274.2 Hz, CF_2), 105.4 (app. d, *J* = 2.6 Hz, Ar-CH), 48.0 (dd, *J* = 25.7, 23.0 Hz, *C*H_2CF_2*C*H_2),44.4 (dd, *J* = 15.1, 9.6 Hz, C$_q$), 20.5 (CH_3); ^{19}F NMR (377 MHz, acetone-*d*$_6$) δ –62.58 (CF_3), –86.85 (d, *J* = 195.2 Hz, C*F*F), –90.23 (d, *J* = 195.2 Hz, CF*F*); HRMS (TOF-ESI$^+$) *m/z* calcd for $C_{27}H_{21}F_5N_2S^{79}Br^+$ [M+H]$^+$: 579.0529; found 579.0524.

2-(1-((4-Bromophenyl)thio)-3,3-difluorocyclobutyl)benzofuran (52)

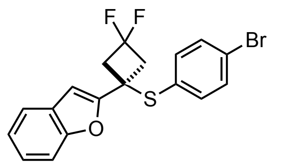

Prepared according to General Procedure C at 110 °C for 3 h using benzofuran difluorocyclobutanol **18** (22.4 mg, 0.1 mmol, 1.0 equiv) and 4-bromothiophenol (56.7 mg, 0.3 mmol, 3.0 equiv). Purification by flash column chromatography (0–20% CH_2Cl_2/hexane) afforded aryl sulfanyl difluorocyclobutane **52** as a white solid (30.1 mg, 91%). R_f = 0.27 (20% CH_2Cl_2/hexane); mp = 120–121 °C; IR (film)/cm^{-1} 3110, 3062, 2952, 2926, 2853, 1472, 1451, 1300, 1246, 1161, 1086, 1010, 818, 807, 751; ^{1}H NMR (400 MHz, $CDCl_3$) δ 7.55–7.48 (m, 2H, 2 × Ar-CH), 7.40–7.33 (m, 3H, 3 × Ar-CH), 7.30–7.23 (m, 1H, Ar-CH), 7.07 (d, *J* = 8.4 Hz, 2H, 2 × Ar-CH), 6.41 (d, *J* = 0.9 Hz, 1H, Ar-CH), 3.40–3.22 (m, 2H, 2 × CH*H*), 3.19–3.06 (m, 2H, 2 × C*H*H); ^{13}C{^{1}H} NMR (101 MHz, $CDCl_3$) δ 157.4 (Ar-*C*$_q$C$_q$), 155.1 (Ar-*C*$_q$O), 136.1 (2 × Ar-CH), 132.1 (2 × Ar-CH), 131.0 (Ar-*C*$_q$S), 127.8 (Ar-*C*$_q$Ar-CH), 124.8 (Ar-CH), 123.7 (Ar-*C*$_q$Br), 123.1 (Ar-CH), 121.1 (Ar-CH), 117.7 (dd, *J* = 279.8, 277.5 Hz, CF_2), 111.3 (Ar-CH), 105.0 (Ar-CH), 47.8 (dd, *J* = 28.0, 23.7 Hz, *C*H_2CF_2*C*H_2), 39.4 (dd, *J* = 13.6, 12.4 Hz, C$_q$); ^{19}F NMR (377 MHz, $CDCl_3$) δ –86.80 (d, *J* = 195.6 Hz), –87.96 (d, *J* = 195.6 Hz); HRMS (FTMS-ESI$^-$) *m/z* calcd for $C_{18}H_{12}F_2OS^{79}Br^-$ [M–H]$^-$: 392.9766; found 392.9764.

4-(1-((4-Bromophenyl)thio)-3,3-difluorocyclobutyl)phenol (53)

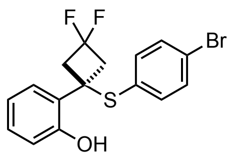

Prepared according to General Procedure C at 110 °C for 3 h using MOM difluorocyclobutanol **22** (24.4 mg, 0.1 mmol, 1.0 equiv) and 4-bromothiophenol (56.7 mg, 0.3 mmol, 3.0 equiv). Purification by flash column chromatography (20% Et_2O/pentane) afforded aryl sulfanyl difluorocyclobutane **53** as an off-white solid (26.9 mg, 73%). R_f = 0.29 (20% Et_2O/pentane); mp = 94–96 °C; IR (film)/cm^{-1} 3401 (OH), 3062, 3034, 2952, 1472, 1451, 1300, 1157, 1114, 1088, 1008, 816, 751; ^{1}H NMR (400 MHz, acetone-*d*$_6$) δ 8.73 (s, 1H, OH), 7.43 (d, *J* = 8.4 Hz, 2H, 2 × Ar-CH), 7.13 (td, *J* = 7.6, 1.8 Hz, 1H, Ar-CH), 7.05 (d, *J* = 8.4 Hz, 2H, 2 × Ar-CH), 6.90 (dd, *J* = 8.0, 1.2 Hz, 1H, Ar-CH), 6.69 (td, *J* = 7.5, 1.2 Hz, 1H, Ar-CH), 6.62 (dd, *J* = 7.7, 1.8 Hz, 1H, Ar-CH), 3.38–3.22 (m, 2H, 2 × CH*H*), 3.21–3.05 (m, 2H, 2 × C*H*H); ^{13}C{^{1}H} NMR (101 MHz, acetone-*d*$_6$) δ 154.7 (Ar-*C*$_q$OH), 138.0 (2 × Ar-CH), 132.2 (Ar-*C*$_q$S), 131.6 (2 × Ar-CH), 129.5 (app. d, *J* = 5.0 Hz, Ar-*C*$_q$C$_q$), 128.8 (Ar-CH), 128.0 (Ar-CH), 123.3 (Ar-*C*$_q$Br), 119.4 (dd, *J* = 279.0, 275.9 Hz, CF_2), 118.7 (Ar-CH), 115.9 (Ar-CH), 47.9 (t, *J* = 22.3, 21.8 Hz, *C*H_2CF_2*C*H_2), 43.3 (dd, *J* = 18.0, 7.5 Hz, C$_q$); ^{19}F NMR (377 MHz, acetone-*d*$_6$) δ –84.70 (d, *J* = 194.2 Hz), –92.08 (d, *J* = 194.2 Hz); HRMS (FTMS-APCI$^-$) *m/z* calcd for $C_{16}H_{12}F_2OS^{79}Br^-$ [M–H]$^-$: 368.9766; found 368.9766.

N.B. Complete in situ deprotection of the ortho-MOM group was observed under the reaction conditions for the thiol alkylation.

(4-(3,3-Difluoro-1-(*p*-tolylthio)cyclobutyl)phenoxy)triisopropylsilane (54)

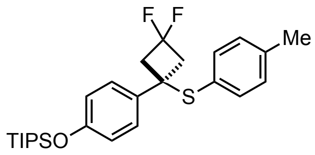

Prepared according to General Procedure C at 110 °C for 3 h using *p*-OTIPS difluorocyclobutanol **4** (35.7 mg, 0.1 mmol, 1.0 equiv) and 4-methylthiophenol (37.3 mg, 0.3 mmol, 3.0 equiv). Purification by flash column chromatography (0–20% CH_2Cl_2/hexane) afforded aryl sulfanyl difluorocyclobutane **54** as a clear colorless oil (36.5 mg, 79%). R_f = 0.24 (20% CH_2Cl_2/hexane); IR (film)/cm^{-1} 2944, 2866, 1507, 1299, 1263, 1241, 1172, 1159, 1103, 909, 881, 810, 684; ^{1}H NMR (400 MHz, $CDCl_3$) δ 7.02 (d, *J* = 8.0 Hz, 2H, 2 × Ar-CH), 6.97 (d, *J* = 8.0 Hz, 2H, 2 × Ar-CH), 6.83 (d, *J* = 8.6 Hz, 2H, 2 × Ar-CH), 6.77 (d, *J* = 8.6 Hz, 2H, 2 × Ar-CH), 3.22–3.00 (m, 4H, 2 × CH_2), 2.34 (s, 3H, CH_3), 1.28 (ddt, *J* = 13.5, 10.1, 6.5 Hz, 3H, 3 × CH), 1.14 (d, *J* = 7.3 Hz, 18H, 6 × CH_3); ^{13}C{^{1}H} NMR (101 MHz, $CDCl_3$) δ 154.9 (Ar-*C*$_q$OTIPS), 139.4 (Ar-*C*$_q$Me), 137.1 (Ar-*C*$_q$C$_q$), 136.4 (2 × Ar-CH), 129.4 (2 × Ar-CH), 128.6 (Ar-*C*$_q$S), 127.8 (2 × Ar-CH), 119.3 (2 × Ar-CH), 118.3 (dd, *J* = 278.1, 272.6 Hz, CF_2), 48.4 (dd, *J* = 23.1, 22.5 Hz, *C*H_2CF_2*C*H_2), 44.3 (dd, *J* = 15.0, 9.1 Hz, C$_q$), 21.3 (CH_3), 17.9 (6 × CH_3), 12.7 (3 × CH); ^{19}F NMR (377 MHz, $CDCl_3$) δ –85.88 (d, *J* = 194.2 Hz), -89.80 (d, *J* = 194.2 Hz); HRMS (FTMS-ESI$^+$) *m/z* calcd for $C_{26}H_{36}F_2OSSiNa^+$ [M+Na]$^+$: 485.2116; found 485.2116.

***N*-(4-((3,3-Difluoro-1-(4-((triisopropylsilyl)oxy)phenyl)cyclobutyl)thio)phenyl)acetamide (55)**

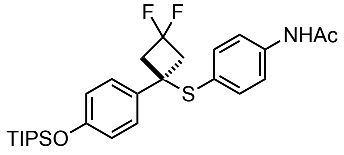

Prepared according to General Procedure C at 110 °C for 3 h using *p*-OTIPS difluorocyclobutanol **4** (35.7 mg, 0.1 mmol, 1.0 equiv) and 4-acetamidothiophenol (50.2 mg, 0.3 mmol, 3.0 equiv). Purification by flash column chromatography (40% EtOAc/hexane) afforded aryl sulfanyl difluorocyclobutane **55** as a clear colorless gum (43.8 mg, 87%). R_f = 0.33 (40% EtOAc/hexane); IR (film)/cm^{-1} 3311 (NH), 2942, 2892, 2866, 1697 (C=O),1669, 1589, 1524, 1507, 1463, 1394, 1299, 1259, 1241, 1172, 1159, 1103, 907, 881, 831, 758, 682, 559, 462; ^{1}H NMR (400 MHz, acetone-*d*$_6$) δ 9.28 (s, 1H, NH), 7.57 (d, *J* = 8.7 Hz, 2H, 2 × Ar-CH), 7.05 (d, *J* = 8.7 Hz, 2H, 2 × Ar-CH), 6.97 (d, *J* = 8.6 Hz, 2H, 2 × Ar-CH), 6.82 (d, *J* = 8.6 Hz, 2H, 2 × Ar-CH), 3.26–3.14 (m, 4H, 2 × CH_2), 2.09 (s, 3H, CH_3), 1.32–1.26 (m, 3H, 3 × CH), 1.14 (d, *J* = 7.3 Hz, 18H, 6 × CH_3); ^{13}C{^{1}H} NMR (101 MHz, acetone-*d*$_6$) δ 168.2 (C=O), 154.8 (Ar-*C*$_q$OTIPS), 140.9 (Ar-*C*$_q$NH), 137.2 (dd, *J* = 4.8, 1.0 Hz, Ar-*C*$_q$C$_q$), 137.0 (2 × Ar-CH), 128.2 (2 × Ar-CH), 125.5 (Ar-*C*$_q$S), 119.2 (2 × Ar-CH), 118.8 (2 × Ar-CH), 118.5 (dd, *J* = 280.8, 277.0 Hz, CF_2), 48.0 (dd, *J* = 23.6, 22.4 Hz, *C*H_2CF_2*C*H_2), 44.2 (dd, *J* = 14.2, 10.4 Hz, C$_q$), 23.5 (CH_3), 17.4 (6 × CH_3), 12.5 (3 × CH); ^{19}F NMR (377 MHz, acetone-*d*$_6$) δ –87.33 (d, *J* = 194.5 Hz), –89.46 (d, *J* = 194.5 Hz); HRMS (TOF-ESI$^+$) *m/z* calcd for $C_{27}H_{38}F_2O_2SNSi^+$ [M+H]$^+$: 506.2361; found 506.2364.

(4-(1-(Benzylthio)-3,3-difluorocyclobutyl)phenoxy)triisopropylsilane (56)

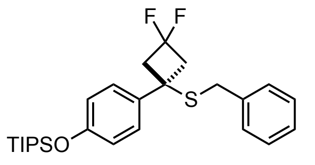

Prepared according to General Procedure C at 110 °C for 3 h using *p*-OTIPS difluorocyclobutanol **4** (35.7 mg, 0.1 mmol, 1.0 equiv) and benzyl mercaptan (35 µL, 0.3 mmol, 3.0 equiv). Purification by flash column chromatography (10–20% CH_2Cl_2/hexane) afforded aryl sulfanyl difluorocyclobutane **56** as a clear colorless oil (41.8 mg, 91%). R_f = 0.14 (20% CH_2Cl_2/hexane); IR (film)/cm^{-1} 2942, 2866, 1604, 1507, 1299, 1263, 1239, 1161, 1101, 907, 881, 684; ^{1}H NMR (400 MHz, $CDCl_3$) δ 7.32–7.22 (m, 3H, 3 × Ar-CH), 7.22–7.13 (m, 4H, 4 × Ar-CH), 6.93 (d, *J* = 8.6 Hz, 2H, 2 × Ar-CH), 3.43 (s, 2H, CH_2), 3.26–3.10 (m, 2H, 2 × CH*H*), 3.02–2.92 (m, 2H, 2 × C*H*H), 1.35–1.26 (m, 3H, 3 × CH), 1.16 (d, *J* = 7.3 Hz, 18H, 6 × CH_3); ^{13}C{^{1}H} NMR (101 MHz, $CDCl_3$) δ 155.0 (Ar-*C$_q$*OTIPS), 137.1 (Ar-*C$_q$*S), 136.3 (dd, *J* = 4.5, 1.2 Hz, Ar-*C$_q$*C$_q$), 129.1 (2 × Ar-CH), 128.5 (2 × Ar-CH), 127.8 (2 × Ar-CH), 127.1 (Ar-CH), 119.7 (2 × Ar-CH), 118.4 (dd, *J* = 277.9, 276.5 Hz), 49.0 (dd, *J* = 23.3, 22.5 Hz, *C*H$_2$CF$_2$*C*H$_2$), 41.5 (dd, *J* = 14.2, 9.4 Hz, C$_q$), 35.5 (CH_2), 17.9 (6 × CH_3), 12.7 (3 × CH); ^{19}F NMR (377 MHz, $CDCl_3$) δ –86.00 (d, *J* = 194.2 Hz), –89.27 (d, *J* = 194.2 Hz); HRMS (FTMS-ESI$^+$) *m/z* calcd for $C_{26}H_{36}F_2OSSiNa^+$ [M+Na]$^+$: 485.2116; found 485.2118.

(4-(1-(((3*s*,5*s*,7*s*)-Adamantan-1-yl)thio)-3,3-difluorocyclobutyl)phenoxy)triisopropylsilane (57)

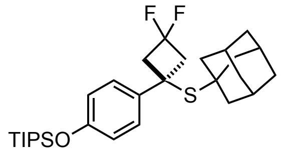

Prepared according to General Procedure C at 110 °C for 3 h using *p*-OTIPS difluorocyclobutanol **4** (35.7 mg, 0.1 mmol, 1.0 equiv) and 1-adamantanethiol (50.5 mg, 0.3 mmol, 3.0 equiv). Purification by flash column chromatography (10–20% CH_2Cl_2/hexane) afforded aryl sulfanyl difluorocyclobutane **57** as an off-white solid (41.0 mg, 81%). R_f = 0.26 (20% CH_2Cl_2/hexane); mp = 96–98 °C; IR (film)/cm^{-1} 2903, 2866, 2849, 1507, 1297, 1265, 1235, 1174, 911, 881, 833, 684; ^{1}H NMR (400 MHz, $CDCl_3$) δ 7.31 (d, *J* = 8.7 Hz, 2H, 2 × Ar-CH), 6.86 (d, *J* = 8.7 Hz, 2H, 2 × Ar-CH), 3.30 (td, *J* = 14.4, 9.5 Hz, 2H, CH_2), 3.12 (dt, *J* = 14.4, 9.5 Hz, 2H, CH_2), 1.85 (br, 3H, 3 × CH), 1.62–1.54 (m, 9H, 4 × CH_2 + C*H*H), 1.54–1.47 (m, 3H, CH_2 + C*H*H), 1.27 (tt, *J* = 9.0, 6.6 Hz, 3H, 3 × CH), 1.12 (d, *J* = 7.3 Hz, 18H, 6 × CH_3); ^{13}C{^{1}H} NMR (101 MHz, $CDCl_3$) δ 154.9 (Ar-*C$_q$*OTIPS), 138.0 (app. d, *J* = 4.1 Hz, Ar-*C$_q$*C$_q$), 128.3 (2 × Ar-CH), 119.6 (2 × Ar-CH), 118.8 (dd, *J* = 280.1, 274.7 Hz, CF_2), 51.4 (dd, *J* = 22.5, 22.1 Hz, *C*H$_2$CF$_2$*C*H$_2$), 49.1 (C$_q$S), 43.4 (3 × CH_2), 40.6 (dd, *J* = 13.1, 11.7 Hz, C$_q$), 36.1 (3 × CH_2), 29.5 (3 × CH), 17.9 (6 × CH_3), 12.7 (3 × CH); ^{19}F NMR (377 MHz, $CDCl_3$) δ –86.65 (d, *J* = 194.4 Hz), –88.12 (d, *J* = 194.4 Hz); HRMS (FTMS-ESI$^+$) *m/z* calcd for $C_{29}H_{44}F_2OSSiNa^+$ [M+Na]$^+$: 529.2742; found 529.2750.

Methyl 3-((3,3-difluoro-1-(4-((triisopropylsilyl)oxy)phenyl)cyclobutyl)thio)propanoate (58)

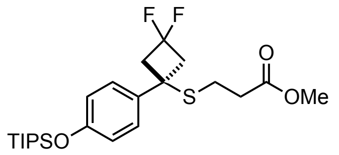

Prepared according to General Procedure C at 110 °C for 3 h using *p*-OTIPS difluorocyclobutanol **4** (35.7 mg, 0.1 mmol, 1.0 equiv) and methyl 3-mercaptopropionate (33 µL, 0.3 mmol, 3.0 equiv). Purification by flash column chromatography (10% Et_2O/pentane) afforded aryl sulfanyl difluorocyclobutane **58** as a clear colorless oil (39.9 mg, 87%). R_f = 0.27 (10% Et_2O/pentane); IR (film)/cm^{-1} 2944, 2866, 1738 (C=O), 1604, 1507, 1300, 1263, 1241, 1161, 1101, 907, 881, 836, 682; ^{1}H NMR (400 MHz, $CDCl_3$) δ 7.14 (d, *J* = 8.7 Hz, 2H, 2 × Ar-CH), 6.87 (d, *J* = 8.7 Hz, 2H, 2 × Ar-CH), 3.65 (s, 3H, OCH_3), 3.33–3.17 (m, 2H, CH_2, 2 × CH*H*), 3.12–2.98 (m, 2H, CH_2, 2 × C*H*H), 2.51 (t, *J* = 7.5 Hz, 2H, CH_2), 2.27 (t, *J* = 7.5 Hz, 2H, CH_2), 1.28 (dtd, *J* = 9.0, 7.4, 7.0, 4.8 Hz, 3H, 3 × CH), 1.12 (d, *J* = 7.3 Hz, 18H, 6 × CH_3); ^{13}C{^{1}H} NMR (101 MHz, $CDCl_3$) δ 172.1 (C=O), 155.1 (Ar-*C$_q$*OTIPS), 136.4 (dd, *J* = 4.4, 1.0 Hz, Ar-*C$_q$*C$_q$), 127.5 (2 × Ar-CH), 119.8 (2 × Ar-CH), 118.2 (dd, *J* = 278.2, 277.9 Hz, CF_2), 51.7 (OCH_3), 49.2 (dd, *J* = 22.6, 22.5 Hz, *C*H$_2$CF$_2$*C*H$_2$), 41.2 (dd, *J* = 14.5, 9.1 Hz, C$_q$), 33.6 (CH_2), 25.7 (CH_2), 17.9 (6 × CH_3), 12.6 (3 × CH); ^{19}F NMR (377 MHz, $CDCl_3$) δ –85.84 (d, *J* = 194.6 Hz), –89.60 (d, *J* = 194.6 Hz); HRMS (TOF-ESI$^+$) *m/z* calcd for $C_{23}H_{40}F_2O_3NSSi^+$ [M+NH$_4$]$^+$: 476.2466; found 476.2449.

Aryl Azide Difluorocyclobutane 59–60

1-(1-Azido-3,3-difluorocyclobutyl)-4-methoxybenzene (59)

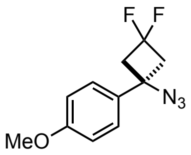

$FeCl_3$ (1.6 mg, 0.01 mmol, 10 mol%) was added to a solution of PMP difluorocyclobutanol **1** (35.7 mg, 0.1 mmol, 1.0 equiv) and $TMSN_3$ (16 µL, 0.12 mmol, 1.2 equiv) in MeCN (0.5 mL, 0.2 M). The vial was sealed and stirred for 24 h at 40 °C. After cooling to rt, the reaction mixture was quenched with aq. NaOH (1M, 10 mL) and diluted with Et_2O (15 mL). The layers were separated, and the aqueous layer was extracted with Et_2O (3 × 15 mL). The combined organic layers were dried over anhydrous Na_2SO_4, filtered, and concentrated *in vacuo*. Purification by flash column chromatography (10% Et_2O/pentane) afforded aryl azide difluorocyclobutane **59** as a clear colorless oil (21.5 mg, 90%). R_f = 0.45 (10% Et_2O/pentane); IR (film)/cm^{-1} 2981, 2840, 2104 (N_3), 1610, 1515, 1304, 1252, 1172, 1034, 833; ^{1}H NMR (400 MHz, $CDCl_3$) δ 7.32–7.27 (m, 2H, 2 × Ar-CH), 7.00–6.95 (m, 2H, 2 × Ar-CH), 3.86 (s, 3H, OCH_3), 3.26–3.05 (m, 4H, 2 × CH_2); ^{13}C{^{1}H} NMR (101 MHz, $CDCl_3$) δ 159.6 (Ar-C_qOMe), 132.0 (dd, J = 4.8, 2.1 Hz, Ar-$C_q$$C_q$), 127.2 (2 × Ar-CH), 117.4 (dd, J = 276.5, 263.2 Hz, CF_2), 114.3 (2 × Ar-CH), 57.4 (dd, J = 15.6, 10.8 Hz, C_q), 55.4 (CH_3), 47.4 (dd, J = 28.0, 23.4 Hz, $CH_2CF_2CH_2$); HRMS (TOF-ESI$^+$) *m/z* calcd for $C_{11}H_{11}F_2ON^+$ [M–N_2+H]$^+$: 212.0881; found 212.0882.

(4-(1-Azido-3,3-difluorocyclobutyl)phenoxy)triisopropylsilane (60)

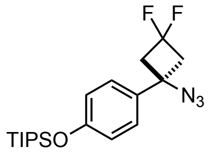

$FeCl_3$ (1.6 mg, 0.01 mmol, 10 mol%) was added to a solution of *p*-OTIPS difluorocyclobutanol **4** (35.7 mg, 0.1 mmol, 1.0 equiv) and $TMSN_3$ (16 µL, 0.12 mmol, 1.2 equiv) in MeCN (0.5 mL, 0.2 M). The vial was sealed and stirred for 24 h at 40 °C. After cooling to rt, the reaction mixture was quenched with aq. NaOH (1M, 10 mL) and diluted with Et_2O (15 mL). The layers were separated, and the aqueous layer was extracted with Et_2O (3 × 15 mL). The combined organic layers were dried over anhydrous Na_2SO_4, filtered, and concentrated *in vacuo*. Purification by flash column chromatography (10% Et_2O/pentane) afforded aryl azide difluorocyclobutane **60** as a clear colorless oil (35.2 mg, 92%). R_f = 0.63 (10% Et_2O/pentane); IR (film)/cm^{-1} 2944, 2868, 2100 (N_3), 1606, 1267, 1302, 1267, 1235, 1172, 907, 881, 838, 684; ^{1}H NMR (400 MHz, $CDCl_3$) δ 7.21 (d, J = 8.6 Hz, 2H, 2 × Ar-CH), 6.95 (d, J = 8.6 Hz, 2H, 2 × Ar-CH), 3.28–3.01 (m, 4H, 2 × CH_2), 1.38–1.22 (m, 3H, 3 × CH), 1.13 (d, J = 7.3 Hz, 18H, 6 × CH_3); ^{13}C{^{1}H} NMR (101 MHz, $CDCl_3$) δ 156.3 (Ar-C_qOTIPS), 132.3 (app. d, J = 4.7 Hz, Ar-$C_q$$C_q$), 127.1 (2 × Ar-CH), 120.2 (2 × Ar-CH), 117.4 (dd, J = 276.4, 272.3 Hz, CF_2), 57.4 (dd, J = 16.4, 10.5 Hz, C_q), 47.5 (dd, J = 27.1, 23.5 Hz, $CH_2CF_2CH_2$), 17.9 (6 × CH_3), 12.6 (3 × CH); ^{19}F NMR (377 MHz, $CDCl_3$) δ –89.27 (d, J = 198.4 Hz), –92.09 (d, J = 198.4 Hz); HRMS (TOF-ESI$^+$) *m/z* calcd for $C_{19}H_{30}F_2ONSi^+$ [M–N_2]$^+$: 354.2065; found 354.2071.

Limitations in the Generation and Trapping of Difluorocyclobutane Carbocations

Below are the unsuccessful substrates in the generation and trapping of difluorocyclobutane carbocations with various nucleophiles (Figure S3). Notably, toluene was not trapped in the absence of an external nucleophile, rather, a complex mixture of products was observed. Anilines also appear to inhibit the Lewis acid catalyst due to their basicity; a distinct color change was observed in these reactions. The reaction between PMP difluorocyclobutanol **1** and resorcinol at 40 °C resulted in di-alkylation of resorcinol due to its poor solubility in toluene. In the case of styrene difluorocyclobutanol **5**, addition into the alkene was observed alongside the desired Friedel-Crafts product. Alkynyl and electron-deficient difluorocyclobutanols were not suitable precursors for carbocation formation, while *p*-substituted phenols were unsuccessful nucleophiles.

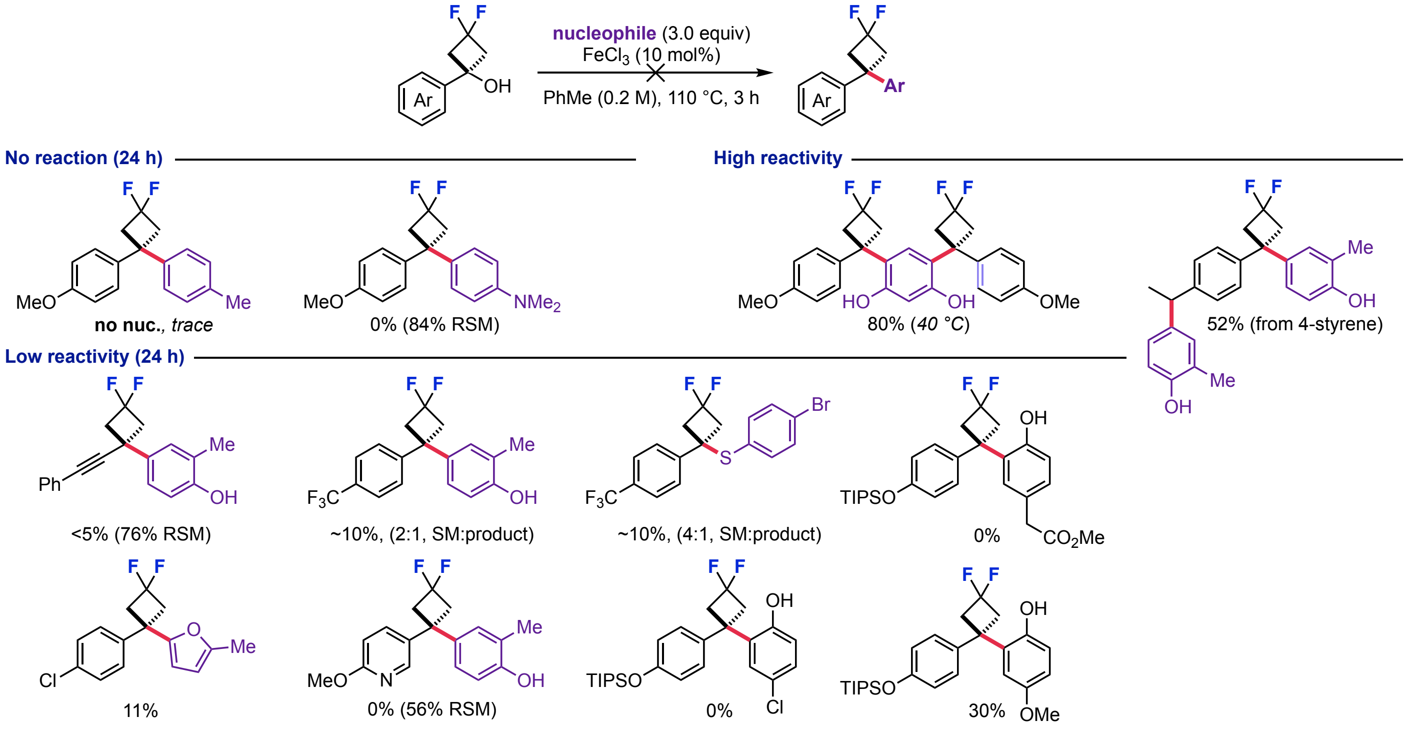

Figure S3: Unsuccessful substrates in the generation and trapping of difluorocyclobutane carbocations.

Trapping of Difluorocyclobutane Radical

3-(3,3-Difluoro-1-(4-methoxyphenyl)cyclobutyl)propanenitrile (68)

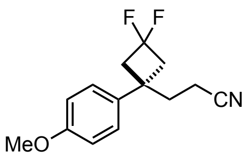

Prepared according to a modified literature procedure.[10] Molecular sieves (15 mg), Zn powder (19.6 mg, 0.3 mmol, 3.0 equiv), and Cp*$TiCl_3$ (2.9 mg, 0.01 mmol, 10 mol%) were added to a reaction vial and sealed. The reaction vial was evacuated and refilled with Ar three times. A solution of PMP difluorocyclobutanol **1** (21.4 mg, 0.1 mmol, 1.0 equiv) in THF (0.25 mL) was added. After a color change from orange to green was observed, acrylonitrile (13 µL, 0.2 mmol, 2.0 equiv) followed by TESCl (50 µL, 0.3 mmol, 3.0 equiv) was added. The reaction mixture was placed in a pre-heated DrySyn® heating block at 60 °C and stirred for 16 h. After cooling to rt, the reaction mixture was filtered through Celite and concentrated *in vacuo*. Purification by flash column chromatography (30% Et_2O/pentane) afforded alkyl difluorocyclobutane **68** as a clear colorless oil (13.0 mg, 52%). R_f = 0.30 (30% Et_2O/pentane); IR (film)/cm^{-1} 3004, 2948, 2838, 2245 (CN), 1610, 1515, 1299, 1246, 1179, 1135, 1030, 918, 833; ^{1}H NMR (400 MHz, $CDCl_3$) δ 7.07 (d, *J* = 8.8 Hz, 2H, 2 × Ar-CH), 6.93 (d, *J* = 8.8 Hz, 2H, 2 × Ar-CH), 3.84 (s, 3H, OCH_3), 3.04–2.90 (m, 2H, CH*H*CF_2CH*H*), 2.90–2.77 (m, 2H, C*H*HCF_2C*H*H), 2.19 (t, *J* = 7.4, 2H, CH_2), 2.01 (t, *J* = 7.4 Hz, 2H, CH_2); ^{13}C{^{1}H} NMR (101 MHz, $CDCl_3$) δ 158.5 (Ar-C_qOMe), 135.7 (Ar-$C_q$$C_q$), 127.1 (2 × Ar-CH), 119.1 (CN), 118.7 (dd, *J* = 280.5, 272.8 Hz, CF_2), 114.3 (2 × Ar-CH), 55.4 (OCH_3), 46.0 (dd, *J* = 22.8, 21.8 Hz, *C*H_2CF_2*C*H_2), 37.7 (app. d, *J* = 3.9 Hz, C_q*C*H_2), 35.1 (dd, *J* = 14.0, 6.8 Hz, C_q), 13.3 (*C*H_2CN); ^{19}F NMR (377 MHz, $CDCl_3$) δ –84.61 (d, *J* = 196.8 Hz), –90.16 (d, *J* = 196.8 Hz); HRMS (FTMS-ESI$^-$) *m/z* calcd for $C_{14}H_{14}F_2ON^-$ [M–H]$^-$: 250.1049; found 250.1049.

<u>*Alternatively*</u>:

Prepared according to a modified literature procedure.[11] Mn powder (11.0 mg, 0.2 mmol, 2.0 equiv) and $TiCl_4$•2THF (66.8 mg, 0.2 mmol, 2.0 equiv) were added to a reaction vial and sealed. The reaction vial was evacuated and refilled with Ar three times. THF (0.25 mL) followed by collidine (30 µL, 0.22 mmol, 2.2 equiv) were added. After stirring for 5 min, a solution of PMP difluorocyclobutanol **1** (21.4 mg, 0.1 mmol, 1.0 equiv) in THF (0.1 mL) and acrylonitrile (26 µL, 0.4 mmol, 4.0 equiv) were added. The reaction mixture was placed in a pre-heated DrySyn® heating block at 70 °C and stirred for 24 h. After cooling to rt, the reaction mixture was diluted with Et_2O (10 mL) and quenched with aq. HCl (1M, 10 mL). The layers were separated, and the aqueous layer was extracted with Et_2O (3 × 10 mL). The combined organic layers were dried over anhydrous Na_2SO_4, filtered, and concentrated *in vacuo*. Purification by flash column chromatography (30% Et_2O/pentane) afforded alkyl difluorocyclobutane **68** as a clear colorless oil (10.3 mg, 41%).

3-(3,3-Difluoro-1-(4-((triisopropylsilyl)oxy)phenyl)cyclobutyl)propanenitrile (69)

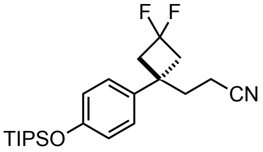

Prepared according to a modified literature procedure.[11] Mn powder (11.0 mg, 0.2 mmol, 2.0 equiv) and $TiCl_4$•2THF (66.8 mg, 0.2 mmol, 2.0 equiv) were added to a reaction vial and sealed. The reaction vial was evacuated and refilled with Ar three times. THF (0.25 mL) followed by collidine (30 µL, 0.22 mmol, 2.2 equiv) were added. After stirring for 5 min, a solution of *p*OTIPS difluorocyclobutanol **4** (35.7 mg, 0.1 mmol, 1.0 equiv) in THF (0.1 mL) and acrylonitrile (26 µL, 0.4 mmol, 4.0 equiv) were added. The reaction mixture was placed in a pre-heated DrySyn® heating block at 70 °C and stirred for 24 h. After cooling to rt, the reaction mixture was diluted with Et_2O (10 mL) and quenched with aq. HCl (1M, 10 mL). The layers were separated, and the aqueous layer was extracted with Et_2O (3 × 10 mL). The combined organic layers were dried over anhydrous Na_2SO_4, filtered, and concentrated *in vacuo*. Purification by flash column chromatography (30% Et_2O/pentane) afforded alkyl difluorocyclobutane **69** as a clear colorless oil (19.5 mg, 50%). R_f = 0.21 (20% Et_2O/pentane); IR (film)/cm^{-1} 2944, 2868, 2249 (CN), 1727, 1606, 1511, 1463, 1300, 1265, 915, 883, 687; ^{1}H NMR (400 MHz, $CDCl_3$) δ 6.98 (d, *J* = 8.6 Hz, 2H, 2 × Ar-CH), 6.89 (d, *J* = 8.6 Hz, 2H, 2 × Ar-CH), 3.04–2.89 (m, 2H, CH*H*CF_2CH*H*), 2.89–2.76 (m, 2H, C*H*HCF_2C*H*H), 2.18 (t, *J* = 7.6 Hz, 2H, CH_2), 2.00 (t, *J* = 7.6 Hz, 2H, CH_2), 1.30–1.24 (m, 3H, 3 × CH), 1.12 (d, *J* = 7.3 Hz, 18H, 6 × CH_3); ^{13}C{^{1}H} NMR (126 MHz, $CDCl_3$) δ 155.1 (Ar-C_qOTIPS), 136.0 (app. d, *J* = 4.0 Hz, Ar-$C_q$$C_q$), 126.9 (2 × Ar-CH), 120.2 (2 × Ar-CH), 119.1 (CN), 118.7 (dd, *J* =279.6, 272.7 Hz, CF_2), 45.9 (dd, *J* = 25.8, 22.1 Hz, *C*H_2CF_2*C*H_2), 37.6 (app. d, *J* = 4.6 Hz, C_q*C*H_2), 35.1 (dd, *J* = 14.2, 6.3 Hz, C_q), 17.9 (6 × CH_3), 13.3 (*C*H_2CN), 12.6 (3 × CH); ^{19}F NMR (377 MHz, $CDCl_3$) δ –84.34 (d, *J* = 196.9 Hz), –90.43 (d, *J* = 196.9 Hz); HRMS (TOF-ESI$^+$) *m/z* calcd for $C_{22}H_{37}F_2ON_2Si^+$ [M+NH$_4$]$^+$: 411.2643; found 411.2654.

Further Functionalisation of Difluorocyclobutane Derivatives

3,3-Difluoro-1-(4-(prop-2-yn-1-yloxy)phenyl)cyclobutan-1-ol (61)

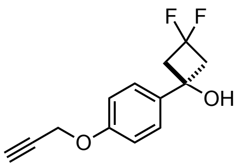

Propargyl bromide (80 w/w% in PhMe, 14 µL, 0.12 mmol, 1.2 equiv) was added to a solution of *p*-OH difluorocyclobutanol **9** (20.0 mg, 0.1 mmol, 1.0 equiv) in acetone (0.5 mL, 0.2 M). The vial was sealed and stirred for 14 h at 60 °C. After cooling to rt, the reaction mixture was diluted with Et_2O (15 mL) and quenched with H_2O (15 mL). The layers were separated, and the aqueous layer was extracted with Et_2O (3 × 15 mL). The combined organic layers were dried over anhydrous Na_2SO_4, filtered, and concentrated *in vacuo* to afford propargyl difluorocyclobutanol **61** as a yellow oil (23.9 mg, quant.). R_f = 0.27 (50% Et_2O/pentane); IR (film)/cm^{-1} 3401 (OH), 3295, 1608, 1511, 1295, 1224, 1176, 1023, 836; ^{1}H NMR (400 MHz, $CDCl_3$) δ 7.41 (d, *J* = 6.9 Hz, 2H, 2 × Ar-CH), 7.02 (d, *J* = 6.9 Hz, 2H, 2 × Ar-CH), 4.73 (s, 2H, CH_2), 3.22–3.07 (m, 2H, CH_2), 3.07–2.93 (m, 2H, CH_2), 2.57–2.50 (m, 1H, OH), 2.25 (s, 1H, CH); ^{13}C{^{1}H} NMR (101 MHz, $CDCl_3$) δ 157.2 (Ar-*C$_q$*OCH$_2$), 137.3 (dd, *J* = 6.2, 2.5 Hz, Ar-*C$_q$*C$_q$), 126.4 (2 × Ar-CH), 118.1 (dd, *J* = 279.1, 276.1 Hz, CF_2), 115.1 (2 × Ar-CH), 78.3 (C$_q$), 75.8 (CH), 67.43 (dd, *J* = 15.1, 12.1 Hz, C$_q$), 55.8 (OCH_2), 50.0 (dd, *J* = 22.7, 22.1 Hz, *C*H$_2$CF$_2$*C*H$_2$); ^{19}F NMR (377 MHz, $CDCl_3$) δ –90.38 (d, *J* = 198.5 Hz), –92.62 (d, *J* = 198.5 Hz); HRMS (FTMS-ESI$^-$) *m/z* calcd for $C_{13}H_{10}FO_2^-$ [M–H$_2$F]$^-$: 217.0670; found 217.0667.

3,3-Difluoro-1-(4-methoxyphenyl)cyclobutane-1-carboxylic acid (62)

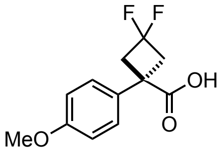

PMP furan difluorocyclobutane **21** (13.9 mg, 0.05 mmol, 1.0 equiv) was added to a solution of $NaIO_4$ (74.9 mg, 0.35 mmol, 7.0 equiv) in EtOAc/heptane/H_2O (1:1:2, 1.24 mL, 0.04 M). After stirring for 5 min at rt, the reaction mixture was cooled to 0 °C and $RuCl_3$ (0.5 mg, 2.5 µmol, 5 mol%) was added. The vial was sealed and stirred for 20 h at 25 °C. The reaction mixture was quenched with H_2O (10 mL) and diluted with sat. aq. $Na_2S_2O_3$ (10 mL) and EtOAc (10 mL). The layers were separated, and the aqueous layer was extracted with EtOAc (3 × 10 mL). The combined organic layers were extracted with aq. NaOH (1M, 3 x 10 mL). The aqueous extracts were combined and acidified with aq. HCl (2M, 30 mL) to pH 1. The resulting aqueous solution was extracted with EtOAc (5 × 20 mL). The combined organic extracts were dried over anhydrous Na_2SO_4, filtered, and concentrated *in vacuo* to afford difluorocyclobutane carboxylic acid **62** as a white gum (7.2 mg, 60%). R_f = 0.53 (30% Et_2O/pentane); IR (film)/cm^{-1} 3006 (OH), 2959, 2933, 2838, 1701 (C=O), 1511, 1299, 1176, 1128, 1034, 833; ^{1}H NMR (500 MHz, acetone-*d$_6$*) δ 7.32 (d, *J* = 8.8 Hz, 2H, 2 × Ar-CH), 6.93 (d, *J* = 8.8 Hz, 2H, 2 × Ar-CH), 3.79 (s, 3H, OCH_3), 3.46–3.35 (m, 2H, 2 × CH*H*), 3.10–3.00 (m, 2H, 2 × *C*HH); ^{13}C{^{1}H} NMR (126 MHz, acetone-*d$_6$*) δ 175.0 (C=O), 159.9 (Ar-*C$_q$*OMe), 133.9 (app. d, *J* = 2.5 Hz, Ar-*C$_q$*C$_q$), 128.7 (2 × Ar-CH), 119.5 (dd, *J* = 277.9, 273.6 Hz, CF_2), 114.7 (2 × Ar-CH), 55.5 (OCH_3), 45.8 (dd, *J* = 25.1, 23.2 Hz, *C*H$_2$CF$_2$*C*H$_2$), 41.8 (dd, *J* = 13.9, 8.9 Hz, C$_q$); ^{19}F NMR (377 MHz, acetone-*d$_6$*) δ –83.46 (d, *J* = 194.9 Hz), –87.59 (d, *J* = 194.9 Hz); HRMS (FTMS-ESI$^-$) *m/z* calcd for $C_{12}H_{11}F_2O_3^-$ [M–H]$^-$: 241.0682; found 241.0684. The observed characterization data (^{1}H) was consistent with that previously reported.[12]

(4-(3,3-Difluoro-1-(*p*-tolylsulfinyl)cyclobutyl)phenoxy)triisopropylsilane (63)

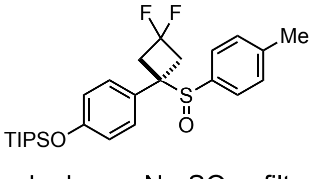

m-CPBA (70 wt.%, 13 mg, 0.052 mmol, 1.05 equiv) was added to a solution of OTIPS difluorocyclobutane sulfide **54** (23.6 mg, 0.05 mmol, 1.0 equiv) in CH_2Cl_2 (0.5 mL, 0.1 M) at 0 °C. After stirring for 3 h at 0 °C, the reaction mixture was quenched with sat. aq. $NaHCO_3$ (10 mL) and diluted with CH_2Cl_2 (10 mL). The layers were separated, and the aqueous layer was extracted with CH_2Cl_2 (5 × 10 mL). The combined organic layers were dried over anhydrous Na_2SO_4, filtered, and concentrated *in vacuo*. Purification by flash column chromatography (25% Et_2O/pentane) afforded difluorocyclobutane sulfoxide **63** as a clear colorless oil (22.7 mg, 95%). R_f = 0.21 (20% Et_2O/pentane); IR (film)/cm^{-1} 2942, 2866, 1604, 1509, 1463, 1300, 1267, 1243, 1166, 1053, 907, 881, 833, 807, 661, 501; ^{1}H NMR (400 MHz, $CDCl_3$) δ 7.06 (d, *J* = 7.9 Hz, 2H, 2 × Ar-CH), 6.86–6.78 (m, 4H, 4 × Ar-CH), 6.73 (d, *J* = 8.7 Hz, 2H, 2 × Ar-CH), 3.65–3.46 (m, 2H, CH_2), 3.05–2.92 (m, 1H, C*H*H), 2.74–2.60 (m, 1H, CH*H*), 2.36 (s, 3H, CH_3), 1.34–1.23 (m, 3H, 3 × CH), 1.14 (d, *J* = 7.3 Hz, 18H, 6 × CH_3); ^{13}C{^{1}H} NMR (126 MHz, $CDCl_3$) δ 156.3 (Ar-*C$_q$*OTIPS), 141.9 (Ar-*C$_q$*S), 135.5 (Ar-*C$_q$*Me), 129.6 (2 × Ar-CH), 128.9 (2 × Ar-CH), 126.8 (app. d, *J* = 3.7 Hz, Ar-*C$_q$*C$_q$), 125.4 (2 × Ar-CH), 119.7 (2 × Ar-CH), 116.9 (dd, *J* = 284.8, 271.4 Hz, CF_2), 57.8 (dd, *J* = 15.8, 6.9 Hz, C$_q$), 44.1 (dd, *J* = 25.9, 23.7 Hz, CH$_2$CF$_2$*C*H$_2$), 36.3 (dd, *J* = 24.3, 23.6 Hz, *C*H$_2$CF$_2$CH$_2$), 21.4 (CH_3), 17.9 (6 × CH_3), 12.6 (3 × CH); ^{19}F NMR (377 MHz,

$CDCl_3$) δ –86.80 (d, *J* = 191.6 Hz), –93.44 (d, *J* = 191.6 Hz); HRMS (TOF-ESI$^+$) *m/z* calcd for $C_{28}H_{39}F_2O_2NSSiNa^+$ [M+MeCN+Na]$^+$: 542.2337; found 542.2339.

4-(1-((4-Bromophenyl)sulfonyl)-3,3-difluorocyclobutyl)phenol (64)

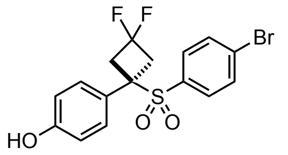

m-CPBA (70 wt.%, 29.6 mg, 0.12 mmol, 3.0 equiv) was added to a solution of OH difluorocyclobutane sulfide **45** (14.9 mg, 0.04 mmol, 1.0 equiv) in CH_2Cl_2 (0.8 mL, 0.05 M). After stirring for 3 h at rt, the reaction mixture was quenched with sat. aq. $NaHCO_3$ (10 mL) and diluted with CH_2Cl_2 (10 mL). The layers were separated, and the aqueous layer was extracted with CH_2Cl_2 (5 × 10 mL). The combined organic layers were dried over anhydrous Na_2SO_4, filtered, and concentrated *in vacuo*. Purification by flash column chromatography (50% Et_2O/pentane) afforded difluorocyclobutane sulfone **64** as a white solid (16.3 mg, quant.). R_f = 0.24 (50% Et_2O/pentane); mp = decomposition observed at 210 °C; IR (film)/cm^{-1} 3371 (OH), 1612, 1572, 1517, 1306, 1274, 1170, 1148, 1077, 1010, 825, 620; ^1H NMR (400 MHz, acetone-d_6) δ 8.71 (s, 1H, OH), 7.71 (d, *J* = 6.7 Hz, 2H, 2 × Ar-CH), 7.33 (d, *J* = 6.7 Hz, 2H, 2 × Ar-CH), 6.99 (d, *J* = 8.6 Hz, 2H, 2 × Ar-CH), 6.76 (d, *J* = 8.6 Hz, 2H, 2 × Ar-CH), 3.97–3.66 (m, 2H, 2 × CH*H*), 3.32–3.19 (m, 2H, 2 × *C*HH); $^{13}C\{^1H\}$ NMR (126 MHz, acetone-d_6) δ 158.2 (Ar-C_qOH), 134.9 (Ar-C_qS), 132.4 (2 × Ar-CH), 131.8 (2 × Ar-CH), 131.2 (2 × Ar-CH), 129.1 (Ar-C_qMe), 125.1 (app. d, *J* = 3.6 Hz, Ar-C_qC_q), 117.2 (dd, *J* = 270.8, 269.5 Hz, CF_2), 115.2 (2 × Ar-CH), 59.3 (dd, *J* = 16.3, 7.4 Hz, C_q), 43.6 (dd, *J* = 24.8, 20.8 Hz, $CH_2CF_2CH_2$); ^{19}F NMR (377 MHz, acetone-d_6) δ –86.95 (d, *J* = 192.9 Hz), –92.56 (d, *J* = 192.9 Hz); HRMS (FTMS-ESI$^-$) *m/z* calcd for $C_{16}H_{13}F_2O_3S^{79}Br^-$ [M–H]$^-$: 400.9664; found 400.9660.

4-(4-((3,3-Difluoro-1-(4-methoxyphenyl)cyclobutyl)thio)phenyl)-1-methyl-1*H*-pyrazole (65)

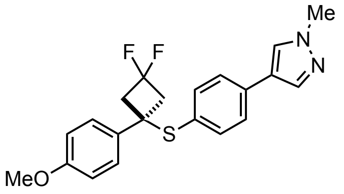

PMP difluorocyclobutane sulfide **46** (38.5 mg, 0.1 mmol, 1.0 equiv), 1-methylpyrazole-4-boronic acid pinacol ester (25.0 mg, 0.12 mmol, 1.2 equiv), K_2CO_3 (41.0 mg, 0.3 mmol, 3.0 equiv), and Pd(dppf)Cl_2 (7.4 mg, 0.01 mmol, 10 mol%) were added to a vial and sealed. The vial was evacuated and re-filled with Ar three times. Degassed 1,4-dioxane (0.4 mL) followed by H_2O (0.2 mL) was added and the reaction mixture was stirred for 20 h at 100 °C. After cooling to rt, quenched with H_2O (10 mL) and diluted with EtOAc (10 mL). The layers were separated, and the aqueous layer was extracted with EtOAc (5 × 10 mL). The combined organic layers were dried over anhydrous Na_2SO_4, filtered, and concentrated *in vacuo*. Purification by flash column chromatography (60% EtOAc/hexane) afforded difluorocyclobutane sulfide **65** as a white solid (27.8 mg, 72%). R_f = 0.21 (60% EtOAc/hexane); mp = 100–102 °C; IR (film)/cm^{-1} 3000, 2939, 2834, 1600, 1511, 1405, 1297, 1239, 1157, 1094, 952, 829, 799, 681, 661, 508; ^1H NMR (400 MHz, $CDCl_3$) δ 7.77 (s, 1H, Ar-CH), 7.62 (s, 1H, Ar-CH), 7.34 (d, *J* = 8.4 Hz, 2H, 2 × Ar-CH), 7.08 (d, *J* = 6.9 Hz, 2H, 2 × Ar-CH), 6.96 (d, *J* = 8.4 Hz, 2H, 2 × Ar-CH), 6.81 (d, *J* = 6.9 Hz, 2H, 2 × Ar-CH), 3.96 (s, 3H, NCH_3), 3.82 (s, 3H, OCH_3), 3.23–3.11 (m, 4H, $CH_2CF_2CH_2$); $^{13}C\{^1H\}$ NMR (101 MHz, $CDCl_3$) δ 158.4 (Ar-C_qOMe), 136.8 (3 × Ar-CH), 136.7 (app. d, *J* = 4.6 Hz, Ar-C_qC_q), 133.6 (Ar-C_qS), 129.6 (Ar-C_qAr-C_q), 127.9 (2 × Ar-CH), 127.2 (Ar-CH), 125.5 (2 × Ar-CH), 122.3 (Ar-C_qAr-C_q), 118.2 (dd, *J* = 278.1, 271.0 Hz, CF_2), 113.4 (2 × Ar-CH), 55.3 (OCH_3), 48.4 (dd, *J* = 25.1, 22.6 Hz, $CH_2CF_2CH_2$), 44.3 (dd, *J* = 14.7, 9.5 Hz, C_q), 39.2 (NCH_3); ^{19}F NMR (377 MHz, $CDCl_3$) δ –86.19 (d, *J* = 194.6 Hz), –89.54 (d, *J* = 194.6 Hz); HRMS (TOF-ESI$^+$) *m/z* calcd for $C_{21}H_{21}F_2ON_2S^+$ [M+H]$^+$: 387.1343; found 387.1346.

(1-(3,3-Difluoro-1-(4-((triisopropylsilyl)oxy)phenyl)cyclobutyl)-1*H*-1,2,3-triazol-4-yl)methyl ((4-bromophenyl)(methyl)(oxo)-λ6-sulfaneylidene)carbamate (66)

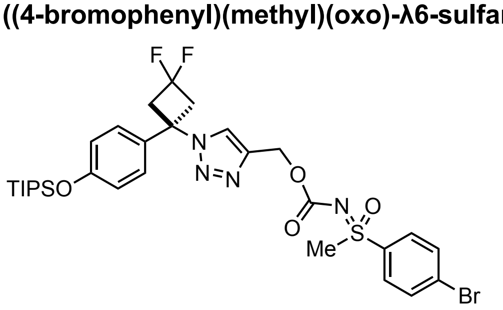

Difluorocyclobutane azide **60** (29.1 mg, 76 μmol, 1.0 equiv), propargyl sulfoximine **S6** (28.8 mg, 91 μmol, 1.2 equiv), sodium ascorbate (3.0 mg, 15 μmol, 20 mol%), and $CuSO_4$ (0.6 mg, 3.8 μmol, 5 mol%) were added to a vial and sealed. The vial was purged with Ar for 5 min. A degassed solution of *t*-BuOH:H_2O (2:1, 0.39 mL, 0.2 M) was added and the reaction mixture was stirred for 24 h at rt. The reaction mixture was quenched with sat. aq. NH_4Cl (10 mL) and diluted with CH_2Cl_2 (10 mL). The layers were separated, and the aqueous layer was extracted with CH_2Cl_2 (5 × 10 mL). The combined organic layers were dried over anhydrous Na_2SO_4, filtered, and concentrated *in vacuo*. Purification by flash column

chromatography (75–90% Et_2O/pentane) afforded difluorocyclobutane triazole **66** as a clear colorless gum (46.1 mg, 87%). R_f = 0.14 (80% Et_2O/pentane); IR (film)/cm^{-1} 2944, 2866, 1669, 1606, 1571, 1511, 1463, 1308, 1231, 1176, 976, 907, 881, 734, 685; ^{1}H NMR (400 MHz, $CDCl_3$) δ 7.82 (d, *J* = 6.7 Hz, 2H, 2 × Ar-CH), 7.73 (d, *J* = 8.6 Hz, 2H, 2 × Ar-CH), 7.33 (s, 1H, Ar-CH), 7.13 (d, *J* = 6.7 Hz, 2H, 2 × Ar-CH), 6.90 (d, *J* = 8.6 Hz, 2H, 2 × Ar-CH), 5.15 (s, 2H, OCH_2), 4.04–3.73 (m, 2H, 2 × CH*H*), 3.60–3.45 (m, 2H, 2 × C*H*H), 3.30 (s, 3H, SCH_3), 1.32–1.23 (m, 3H, 3 × CH), 1.11 (dd, *J* = 7.3, 1.9 Hz, 18H, 6 × CH_3); ^{13}C{^{1}H} NMR (101 MHz, $CDCl_3$) δ 158.3 (C=O), 156.6 (Ar-C_qOTIPS), 143.4 (Ar-$C_q$$CH_2$), 137.1 (Ar-$C_q$S), 133.1 (2 × Ar-CH), 132.1 (app. d, *J* = 3.9 Hz, Ar-$C_q$$C_q$), 129.6 (Ar-$C_q$Br), 129.0 (2 × Ar-CH), 127.5 (2 × Ar-CH), 122.9 (Ar-CH), 120.4 (2 × Ar-CH), 117.0 (dd, *J* = 277.3, 273.0 Hz, CF_2), 59.4 (OCH_2), 57.0 (dd, *J* = 16.8, 12.6 Hz, Cq), 48.7 (t, *J* = 27.2, 25.0 Hz, *C*H_2CF_2*C*H_2), 44.5 (SCH_3), 17.9 (6 × CH_3), 12.6 (3 × CH); ^{19}F NMR (377 MHz, $CDCl_3$) δ –89.43 (d, *J* = 199.4 Hz), –92.11 (d, *J* = 199.4 Hz); HRMS (TOF-ESI$^+$) *m/z* calcd for $C_{30}H_{40}F_2O_4N_4SSi^{79}Br^+$ $[M+H]^+$: 697.1691; found 697.1710.

3,3-Difluoro-1-(4-methoxyphenyl)cyclobutan-1-amine (67)

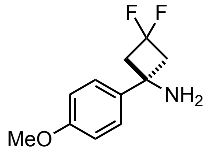

A solution of PMP difluorocyclobutane azide **59** (12.0 mg, 0.05 mmol, 1.0 equiv) in EtOAc (1 mL, 0.05 M), was added to Pd/C (10 wt.%, 10.6 mg, 0.01 mmol, 20 mol%) in a vial and sealed. The vial was evacuated and re-filled with N_2 three times, followed by H_2 three times. The reaction mixture was sparged with H_2 for 15 min and left to stir for 20 h. The reaction mixture was filtered through Celite and concentrated *in vacuo* to afford difluorocyclobutane amine **67** as a clear colorless gum (8.1 mg, 76%). R_f = 0.08 (50% Et_2O/pentane); IR (film)/cm^{-1} 3500 (NH), 3006, 2953, 2912, 1725,1610, 1515, 1295, 1250, 1179, 1030, 634; ^{1}H NMR (400 MHz, $CDCl_3$) δ 7.33 (d, *J* = 8.3 Hz, 2H, 2 × Ar-CH), 6.93 (d, *J* = 8.3 Hz, 2H, 2 × Ar-CH), 3.84 (s, 3H, OCH_3), 3.12 (q, *J* = 13.6 Hz, 2H, 2 × CH*H*), 2.80 (td, *J* = 13.9, 6.9 Hz, 2H, 2 × C*H*H); ^{13}C{^{1}H} NMR (126 MHz, $CDCl_3$) δ 158.6 (Ar-C_qOMe), 138.8 (dd, *J* = 5.9, 3.3 Hz, Ar-$C_q$$C_q$) 126.3 (2 × Ar-CH), 118.9 (dd, *J* = 279.6, 274.2 Hz, CF_2), 114.0 (2 × Ar-CH), 55.3 (OCH_3), 50.1 (dd, *J* = 24.4, 21.8 Hz, *C*H_2CF_2*C*H_2), 48.9 (dd, *J* = 15.7, 7.9 Hz, *C*H_2CF_2*C*H_2); ^{19}F NMR (377 MHz, $CDCl_3$) δ –88.88 (d, *J* = 197.1 Hz), –91.08 (d, *J* = 197.1 Hz); HRMS (TOF-ESI$^+$) *m/z* calcd for $C_{11}H_{13}F_2NO^+$ $[M+H]^+$: 214.1038; found 214.1033.

Chemical and Thermal Stability Tests

Acid-Base Stability

A solution of aq. HCl (1M, 1 mL) was added to a solution of diaryl difluorocyclobutane **24** (15.2 mg, 0.05 mmol) or difluorocyclobutane sulfide **46** (19.3 mg, 0.05 mmol) in MeOH (0.3 mL). After stirring at 37 °C for 24 h, the reaction mixture was neutralized to pH 7 and diluted with brine (10 mL) followed by Et_2O (10 mL). The layers were separated, and the aqueous layer was extracted with Et_2O (3 × 10 mL). The combined organic layers were dried over anhydrous Na_2SO_4, filtered, and concentrated *in vacuo*. The recovery was determined by ^1H NMR spectroscopy with a 30 s delay using 1,3,5-trimethoxybenzene as an internal standard.

A solution of aq. NaOH (1M, 1 mL) was added to a solution of diaryl difluorocyclobutane **24** (15.2 mg, 0.05 mmol) or difluorocyclobutane sulfide **46** (19.3 mg, 0.05 mmol) in MeOH (0.3 mL). After stirring at rt for 24 h, the reaction mixture was neutralized to pH 7 and diluted with brine (10 mL) followed by Et_2O (10 mL). The layers were separated, and the aqueous layer was extracted with Et_2O (3 × 10 mL). The combined organic layers were dried over anhydrous Na_2SO_4, filtered, and concentrated *in vacuo*. The recovery was determined by ^1H NMR spectroscopy with a 30 s delay using 1,3,5-trimethoxybenzene as an internal standard.

Buffer Stability

PBS solution (1 mL) was added to a solution of diaryl difluorocyclobutane **24** (15.2 mg, 0.05 mmol) or difluorocyclobutane sulfide **46** (19.3 mg, 0.05 mmol) in DMSO (0.1 mL). After stirring at 37 °C for 24 h, the reaction mixture was diluted with brine (10 mL) followed by Et_2O (10 mL). The layers were separated, and the aqueous layer was extracted with Et_2O (3 × 10 mL). The combined organic layers were dried over anhydrous Na_2SO_4, filtered, and concentrated *in vacuo*. The recovery was determined by ^1H NMR spectroscopy with a 30 s delay using 1,3,5-trimethoxybenzene as an internal standard.

Chemical Stability

NaI

NaI (75.0 mg, 0.5 mmol, 10.0 equiv) was added to a solution of diaryl difluorocyclobutane **24** (15.2 mg, 0.05 mmol, 1.0 equiv) or difluorocyclobutane sulfide **46** (19.3 mg, 0.05 mmol, 1.0 equiv) in acetone (0.25 mL, 0.2 M). After stirring at 50 °C for 1 h, the reaction mixture was quenched with sat. aq. $Na_2S_2O_3$ (10 mL) and diluted with Et_2O (10 mL). The layers were separated, and the aqueous layer was extracted with Et_2O (3 × 10 mL). The combined organic layers were dried over anhydrous Na_2SO_4, filtered, and concentrated *in vacuo*. The recovery was determined by ^1H NMR spectroscopy with a 30 s delay using 1,3,5-trimethoxybenzene as an internal standard.

H-Cysteine-OMe

H-Cys-OMe (8.6 mg, 0.05 mmol, 1.0 equiv) and K_2CO_3 (6.9 mg, 0.05 mmol, 1.0 equiv) were added to a solution of diaryl difluorocyclobutane **24** (15.2 mg, 0.05 mmol, 1.0 equiv) or difluorocyclobutane sulfide **46** (19.3 mg, 0.05 mmol, 1.0 equiv) in DMF:H_2O (1:1, 0.5 mL, 0.4 M). After stirring at rt for 24 h, the reaction mixture was diluted with water (10 mL) followed by Et_2O (10 mL). The layers were separated, and the aqueous layer was extracted with Et_2O (3 × 10 mL). The combined organic layers were dried over anhydrous Na_2SO_4, filtered, and concentrated *in vacuo*. The recovery was determined by ^1H NMR spectroscopy with a 30 s delay using 1,3,5-trimethoxybenzene as an internal standard.

Table S6: Results of chemical stability tests.

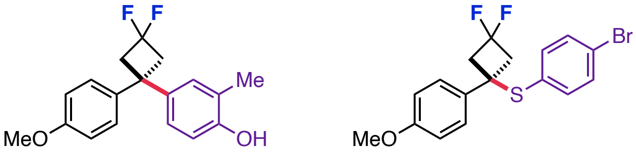

24 **46**

		Recovery (%)[b]	
**Entry**[a]	**Conditions**	**24**	**46**
1	1 M aq. HCl, 37 °C, 24 h	98	98
2	1 M aq. NaOH, rt, 24 h	99	97
3	Phosphate buffered saline, 37 °C, 24 h	99	98
4	10 equiv NaI, acetone, 50 °C, 1 h	99	99
5	1 equiv H-Cys-OMe, DMF/H_2O, rt, 24 h	96	99

[a]On a 50 µmol scale. [b]Recovery determined by ^{1}H NMR spectroscopy using 1,3,5-trimethoxybenzene as an internal standard.

X-Ray Crystallography Details

Crystals suitable for X-ray analysis were grown by slow evaporation from acetone at 25 °C. Data were collected using Agilent Xcalibur PX Ultra A [**44**] and Agilent Xcalibur 3 E [**46**] diffractometers, and the structures were solved by direct methods and refined by full-matrix least squares on F2 for all data using the OLEX2,[13] SHELXTL,[14] and SHELX-2013[15] program systems.[16] Crystallographic data and related CIFs for the structures **44** and **46** have been deposited with the joint Cambridge Crystallographic Data Centre and Fachinformationszentrum Karlsruhe Access Structures service and are available free of charge with the following deposition numbers: CCDC Deposition Numbers: 2433704–2433705.

X-Ray Crystallography Notes

The O–H hydrogen atom in the structure of **44** was located from a Δ*F* map and refined freely subject to an O–H distance constraint of 0.90 Å.

The absolute structure of **44** was determined from the know absolute chirality of the starting material; Flack parameter [x = −0.08(4)].

X-ray Crystal Structure of Diaryl Difluorocyclobutane 44

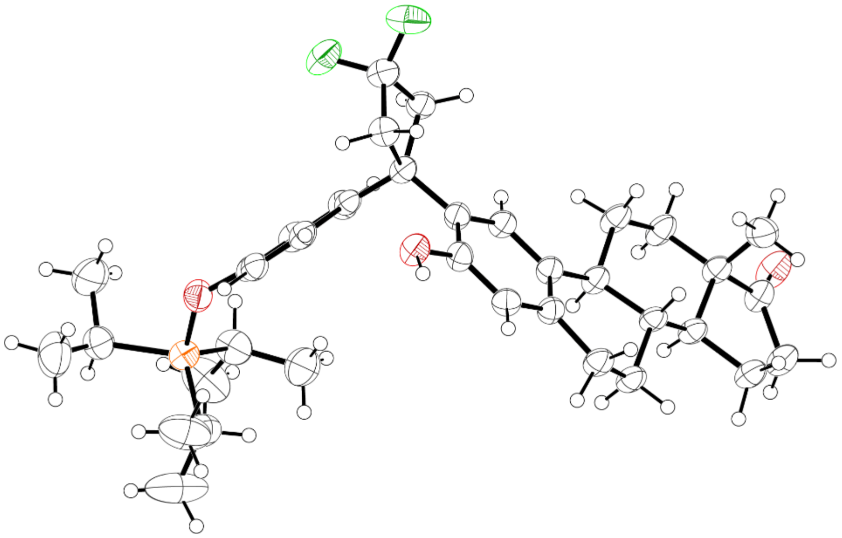

Figure S4: The crystal structure of **44** (50% probability ellipsoids).

CCDC deposition number	2433704
Empirical formula	$C_{37}H_{50}O_3F_2Si$
Formula weight	608.86
Temperature/K	173(4)
Crystal system	monoclinic
Space group	$P2_1$
a/Å	11.0877(5)
b/Å	12.0777(4)
c/Å	13.0123(7)
α/°	90
β/°	103.945(5)
γ/°	90
Volume/Å^3	1691.17(14)
Z	2
ρ_{calc}g/cm^3	1.196
μ/mm^{-1}	0.974
F(000)	656.0
Crystal size/mm^3	0.5 × 0.12 × 0.03
Radiation	Cu Kα (λ = 1.54184)
2Θ range for data collection/°	7 to 147.558
Index ranges	-13 ≤ h ≤ 12, -14 ≤ k ≤ 14, -15 ≤ l ≤ 16
Reflections collected	14500
Independent reflections	6290 [R_{int} = 0.0578, R_{sigma} = 0.0655]
Data/restraints/parameters	6290/2/399
Goodness-of-fit on F^2	1.041
Final R indexes [I>=2σ (I)]	R_1 = 0.0506, wR_2 = 0.1209
Final R indexes [all data]	R_1 = 0.0650, wR_2 = 0.1319
Largest diff. peak/hole / e Å^{-3}	0.30/-0.22
Flack parameter	-0.08(4)

X-ray Crystal Structure of Aryl Sulfanyl Difluorocyclobutane 46

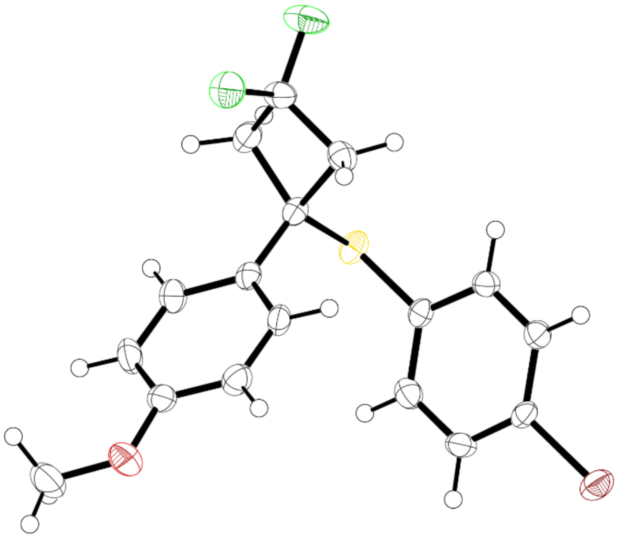

Figure S5: The crystal structure of **46** (50% probability ellipsoids).

CCDC deposition number	2433705
Empirical formula	$C_{17}H_{15}OF_2SBr$
Formula weight	385.26
Temperature/K	173.05(10)
Crystal system	monoclinic
Space group	$P2_1/c$
a/Å	19.8074(10)
b/Å	5.6896(2)
c/Å	15.1336(8)
α/°	90
β/°	112.234(6)
γ/°	90
Volume/Å^3	1578.69(14)
Z	4
ρ_{calc}g/cm^3	1.621
μ/mm^{-1}	2.755
F(000)	776.0
Crystal size/mm^3	0.186 × 0.142 × 0.053
Radiation	Mo Kα (λ = 0.71073)
2Θ range for data collection/°	5.384 to 56.906
Index ranges	-25 ≤ h ≤ 16, -7 ≤ k ≤ 7, -13 ≤ l ≤ 19
Reflections collected	9324
Independent reflections	3319 [R_{int} = 0.0310, R_{sigma} = 0.0441]
Data/restraints/parameters	3319/0/200
Goodness-of-fit on F^2	1.025
Final R indexes [I>=2σ (I)]	R_1 = 0.0324, wR_2 = 0.0596
Final R indexes [all data]	R_1 = 0.0522, wR_2 = 0.0656
Largest diff. peak/hole / e Å^{-3}	0.32/-0.31

References

1. Love, B. E.; Jones, E. G. The Use of Salicylaldehyde Phenylhydrazone as an Indicator for the Titration of Organometallic Reagents. *J. Org. Chem.* **1999**, *64*(10), 3755–3756. DOI: 10.1021/jo982433e.
2. Rojas, J. J.; Croft, R. A.; Sterling, A. J.; Briggs, E. L.; Antermite, D.; Schmitt, D. C.; Blagojevic, L.; Haycock, P.; White, A. J. P.; Duarte, F.; Choi, C.; Mousseau, J. J.; Bull, J. A. Amino-Oxetanes as Amide Isosteres by an Alternative Defluorosulfonylative Coupling of Sulfonyl Fluorides. *Nat. Chem.* **2022**, *14* (2), 160–169. DOI: 10.1038/s41557-021-00856-2.
3. Chen, Y.; Murray, P. R. D.; Davies, A. T.; Willis, M. C. Direct Copper-Catalyzed Three-Component Synthesis of Sulfonamides. *J. Am. Chem. Soc.* **2018**, *140* (28), 8781–8787. DOI: 10.1021/jacs.8b04532.
4. Cai, Z.; Li, S.; Gao, Y.; Li, G. Rhodium(II)-Catalyzed Aryl C−H Carboxylation of 2-Pyridylphenols with CO_2. *Adv Synth Catal* **2018**, *360* (20), 4005–4011. DOI: 10.1002/adsc.201800611.
5. Ma, T.-K.; Elliott, D. C.; Reid, S.; White, A. J. P.; Parsons, P. J.; Barrett, A. G. M. Meroterpenoid Synthesis via Sequential Polyketide Aromatization and Cationic Polyene Cyclization: Total Syntheses of (+)-Hongoquercin A and B and Related Meroterpenoids. *J. Org. Chem.* **2018**, *83* (21), 13276–13286. DOI: 10.1021/acs.joc.8b02095.
6. Zhong, Z.; Chesti, J.; Armstrong, A.; Bull, J. A. Synthesis of Sulfoximine Propargyl Carbamates under Improved Conditions for Rhodium Catalyzed Carbamate Transfer to Sulfoxides. *J. Org. Chem.* **2022**, *87* (23), 16115–16126. DOI: 10.1021/acs.joc.2c02083.
7. Shim, J.; Eid, C.; Lee, J.; Liu, E.; Chaudhary, D.; Boschelli, D. H. Synthesis and PKCθ Inhibitory Activity of a Series of 5-Vinyl Phenyl Sulfonamide-3-Pyridinecarbonitriles. *Bioorg. Med. Chem. Lett.* **2009**, *19* (23), 6575–6577. DOI: 10.1016/j.bmcl.2009.10.031.
8. Lee, Y. H.; Morandi, B. Metathesis-Active Ligands Enable a Catalytic Functional Group Metathesis between Aroyl Chlorides and Aryl Iodides. *Nat. Chem.* **2018**, *10* (10), 1016–1022. DOI: 10.1038/s41557-018-0078-8.
9. Liebeskind, L. S.; Stone, G. B.; Zhang, S. 3-(Tri-*n*-butylstannyl)-2-cyclobuten-1-one: Synthesis and Stille Cross-Coupling as a Route to 3-Substituted Cyclobutenones. *J. Org. Chem.* **1994**, *59*, 7917–7920.
10. Xie, H.; Guo, J.; Wang, Y.-Q.; Wang, K.; Guo, P.; Su, P.-F.; Wang, X.; Shu, X.-Z. Radical Dehydroxylative Alkylation of Tertiary Alcohols by Ti Catalysis. *J. Am. Chem. Soc.* **2020**, *142* (39), 16787–16794. DOI: 10.1021/jacs.0c07492.
11. Suga, T.; Shimazu, S.; Ukaji, Y. Low-Valent Titanium-Mediated Radical Conjugate Addition Using Benzyl Alcohols as Benzyl Radical Sources. *Org. Lett.* **2018**, *20* (17), 5389–5392. DOI: 10.1021/acs.orglett.8b02305.
12. Kanada, R.; Kagoshima, Y.; Suzuki, T.; Nakamura, A.; Funami, H.; Watanabe, J.; Asano, M.; Takahashi, M.; Ubukata, O.; Suzuki, K.; Aikawa, T.; Sato, K.; Goto, M.; Setsu, G.; Ito, K.; Kihara, K.; Kuroha, M.; Kohno, T.; Ogiwara, H.; Isoyama, T.; Tominaga, Y.; Higuchi, S.; Naito, H. Discovery of DS-9300: A Highly Potent, Selective, and Once-Daily Oral EP300/CBP Histone Acetyltransferase Inhibitor. *J. Med. Chem.* **2023**, *66* (1), 695–715. DOI: 10.1021/acs.jmedchem.2c01641
13. Dolomanov, O. V.; Bourhis, L. J.; Gildea, R. J.; Howard, J. A. K.; Puschmann, H. *OLEX2* : A Complete Structure Solution, Refinement and Analysis Program. *J. Appl. Crystallogr*. **2009**, *42* (2), 339–341. DOI: 10.1107/S0021889808042726.
14. SHELXTL v5.1, Bruker AXS, Madison, WI, 1998.
15. SHELX-2013, Sheldrick, G. M. Crystal Structure Refinement with *SHELXL*. *Acta Crystallogr. C Struct. Chem*. **2015**, *71* (1), 3–8. DOI: 10.1107/S2053229614024218
16. A.L. Spek (2003, 2009) PLATON, A Multipurpose Crystallographic Tool, Utrecht University, Utrecht, The Netherlands. See also Spek, A. L. *PLATON* SQUEEZE: A Tool for the Calculation of the Disordered Solvent Contribution to the Calculated Structure Factors. *Acta Crystallogr. C Struct. Chem.* **2015**, *71* (1), 9–18. DOI: 10.1107/S2053229614024929.

^{1}H, $^{13}C\{^{1}H\}$, and ^{19}F NMR Spectra of Novel Compounds

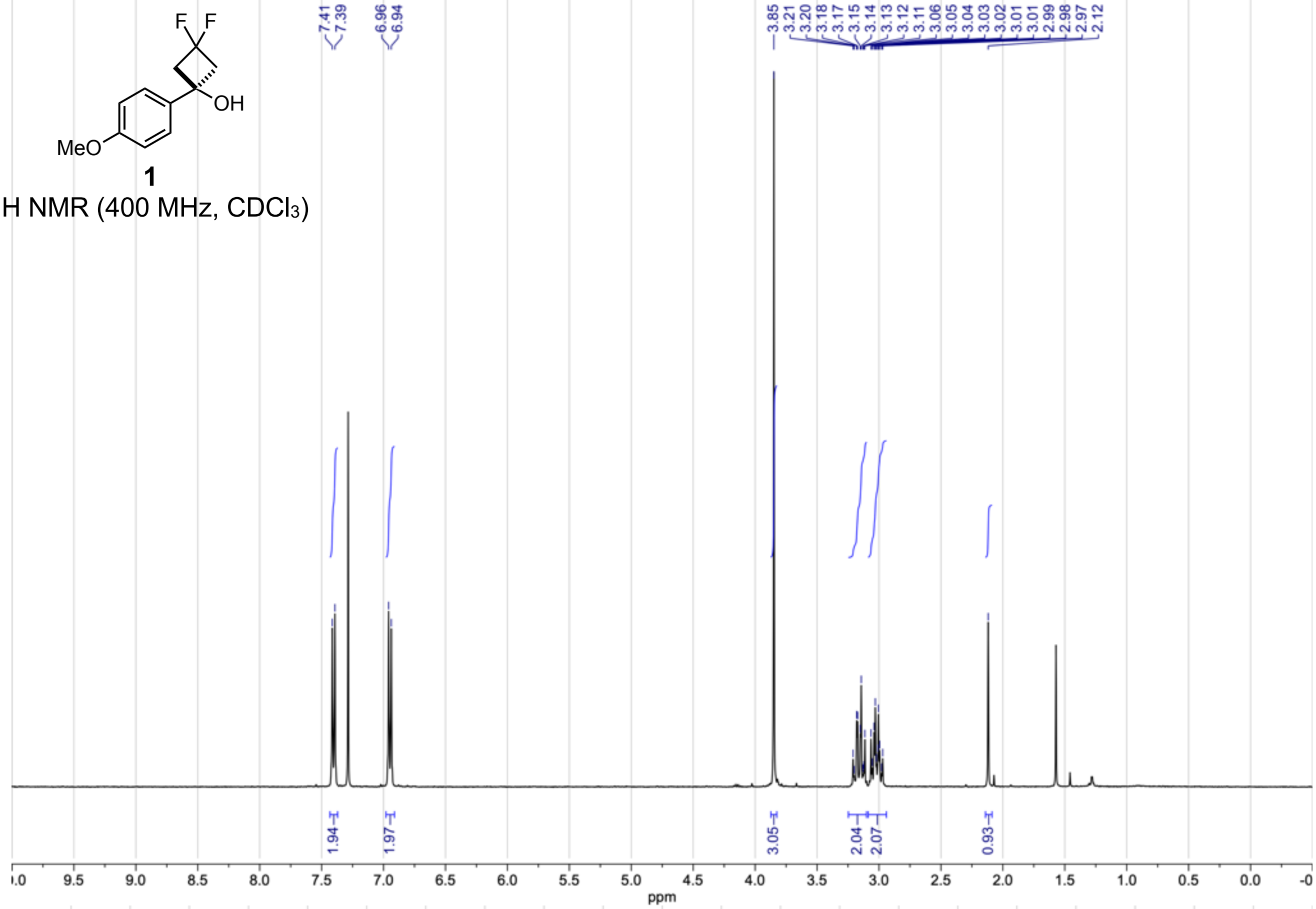

1

^{1}H NMR (400 MHz, $CDCl_3$)

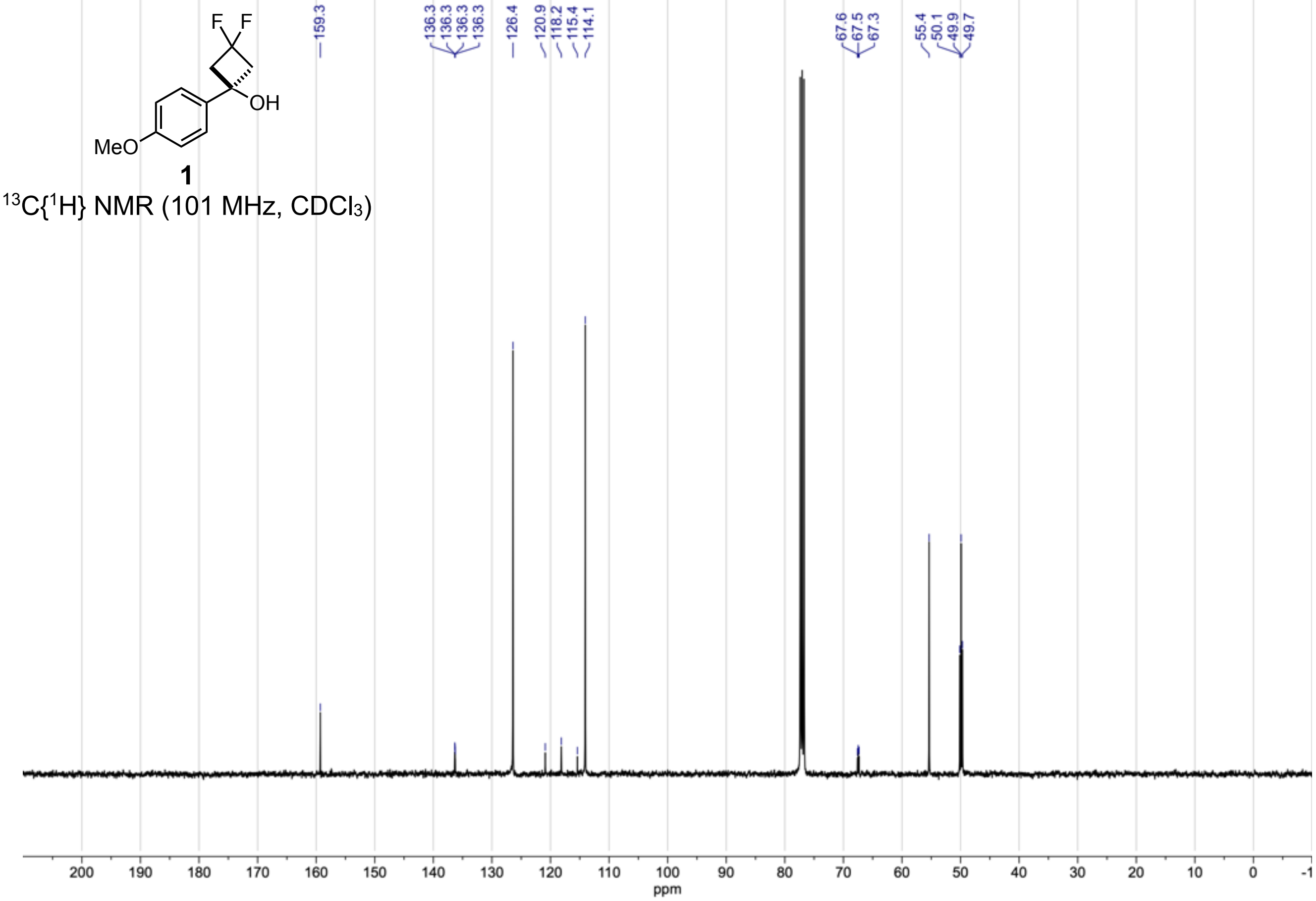

1

$^{13}C\{^{1}H\}$ NMR (101 MHz, $CDCl_3$)

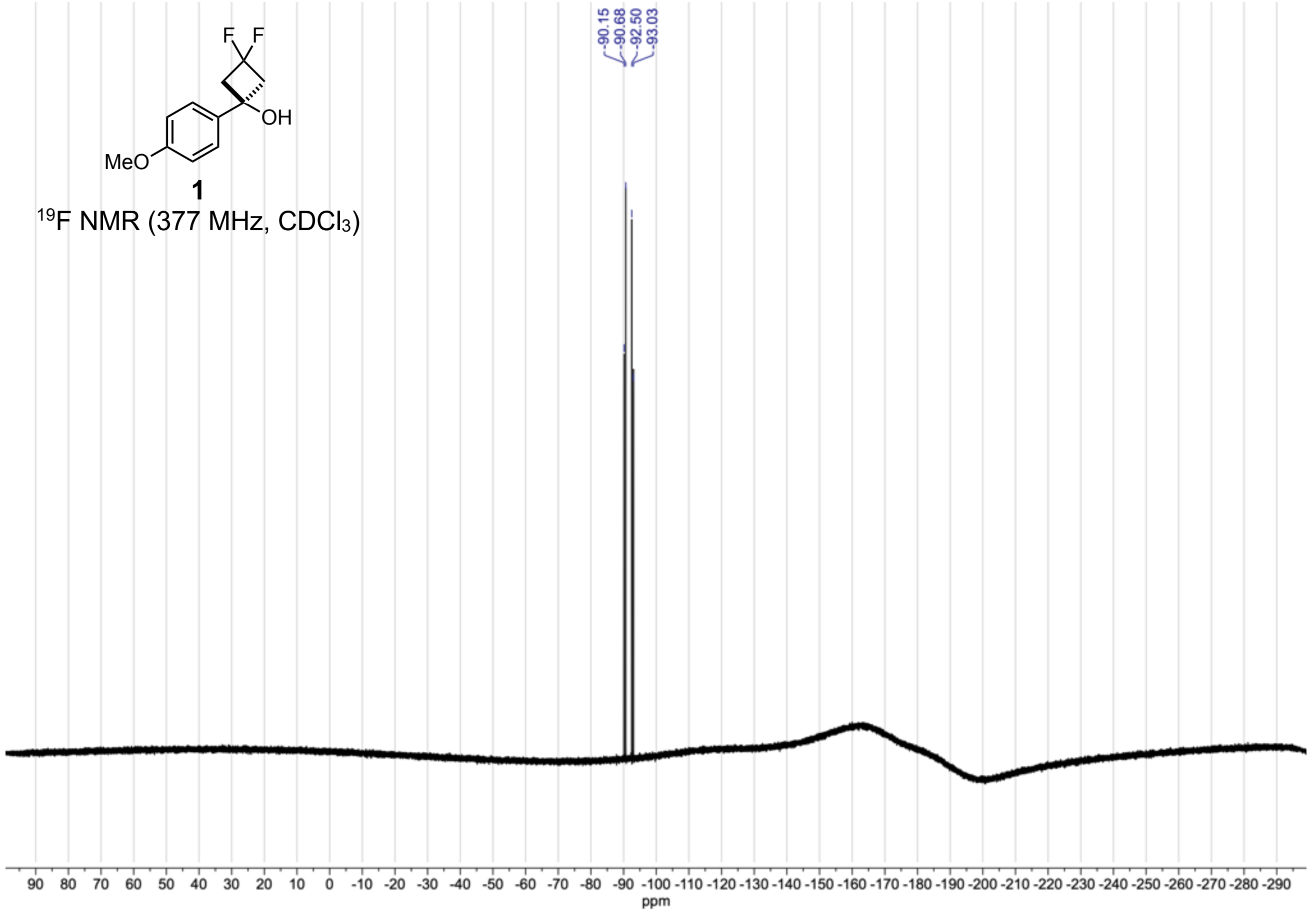
F F
OH
MeO
1
19F NMR (377 MHz, CDCl3)
-90.15
-90.68
-92.50
-93.03
90 80 70 60 50 40 30 20 10 0 -10 -20 -30 -40 -50 -60 -70 -80 -90 -100 -110 -120 -130 -140 -150 -160 -170 -180 -190 -200 -210 -220 -230 -240 -250 -260 -270 -280 -290
ppm

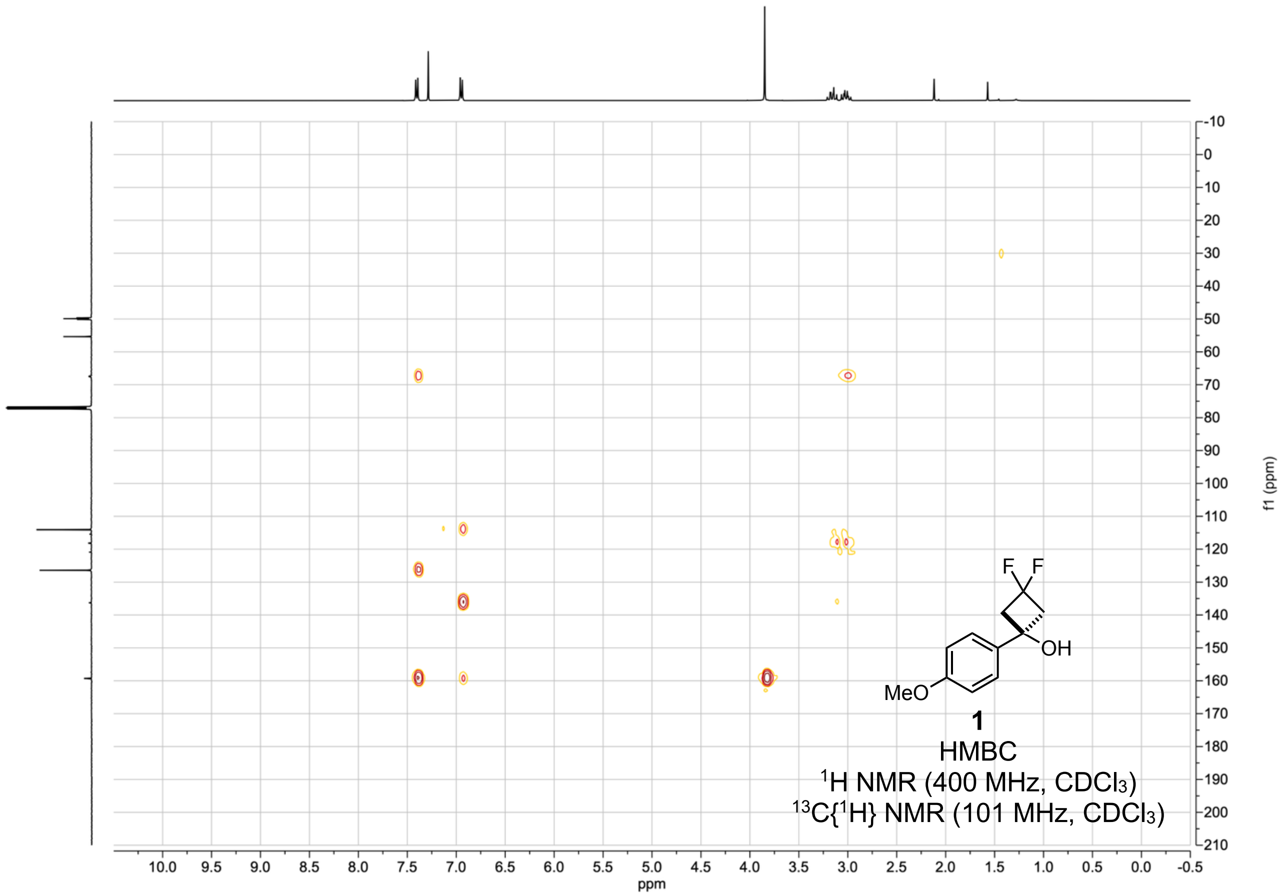
F F
OH
MeO
1
HMBC
1H NMR (400 MHz, CDCl3)
13C{1H} NMR (101 MHz, CDCl3)
f1 (ppm)
ppm

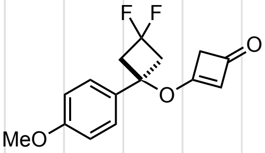

3

^{1}H NMR (400 MHz, $CDCl_3$)

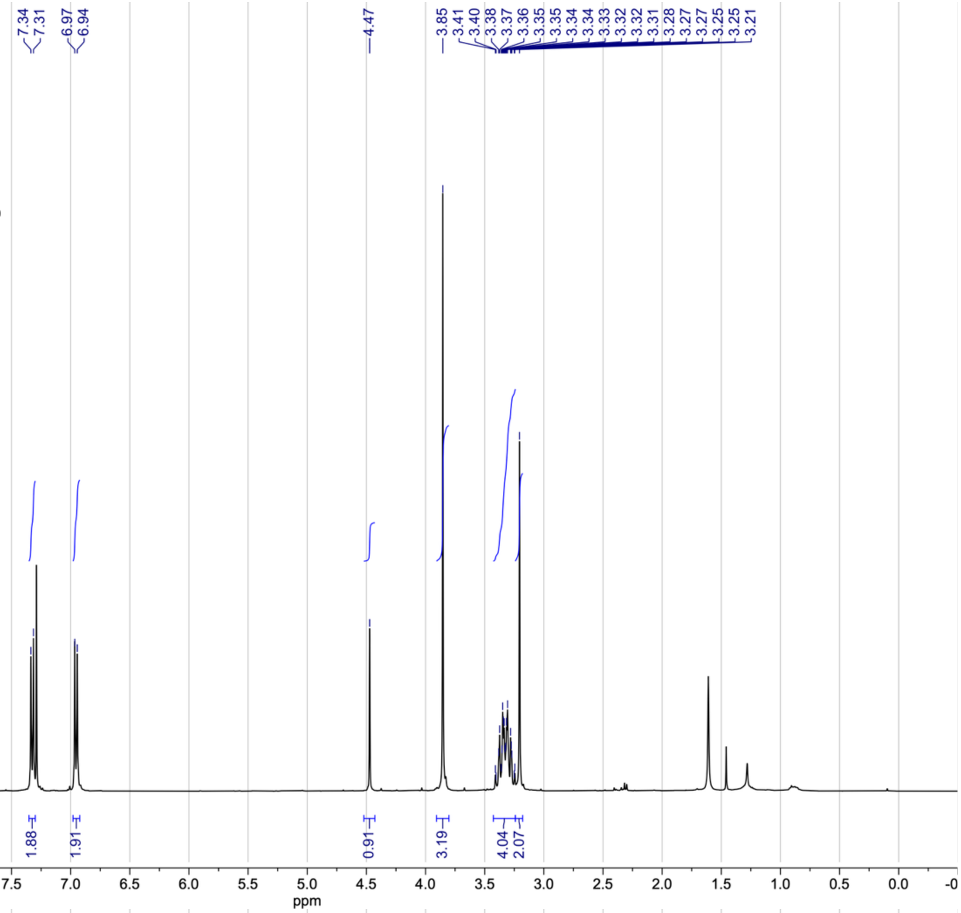

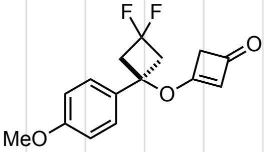

3

$^{13}C\{^{1}H\}$ NMR (101 MHz, $CDCl_3$)

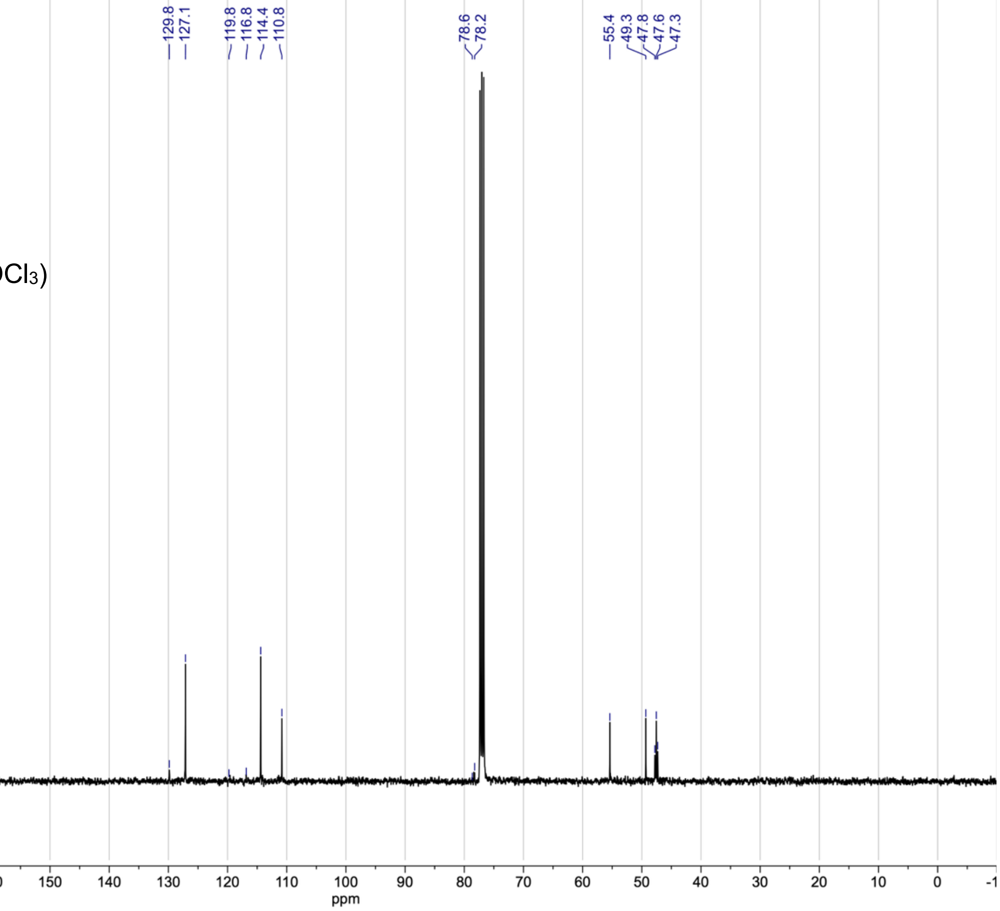

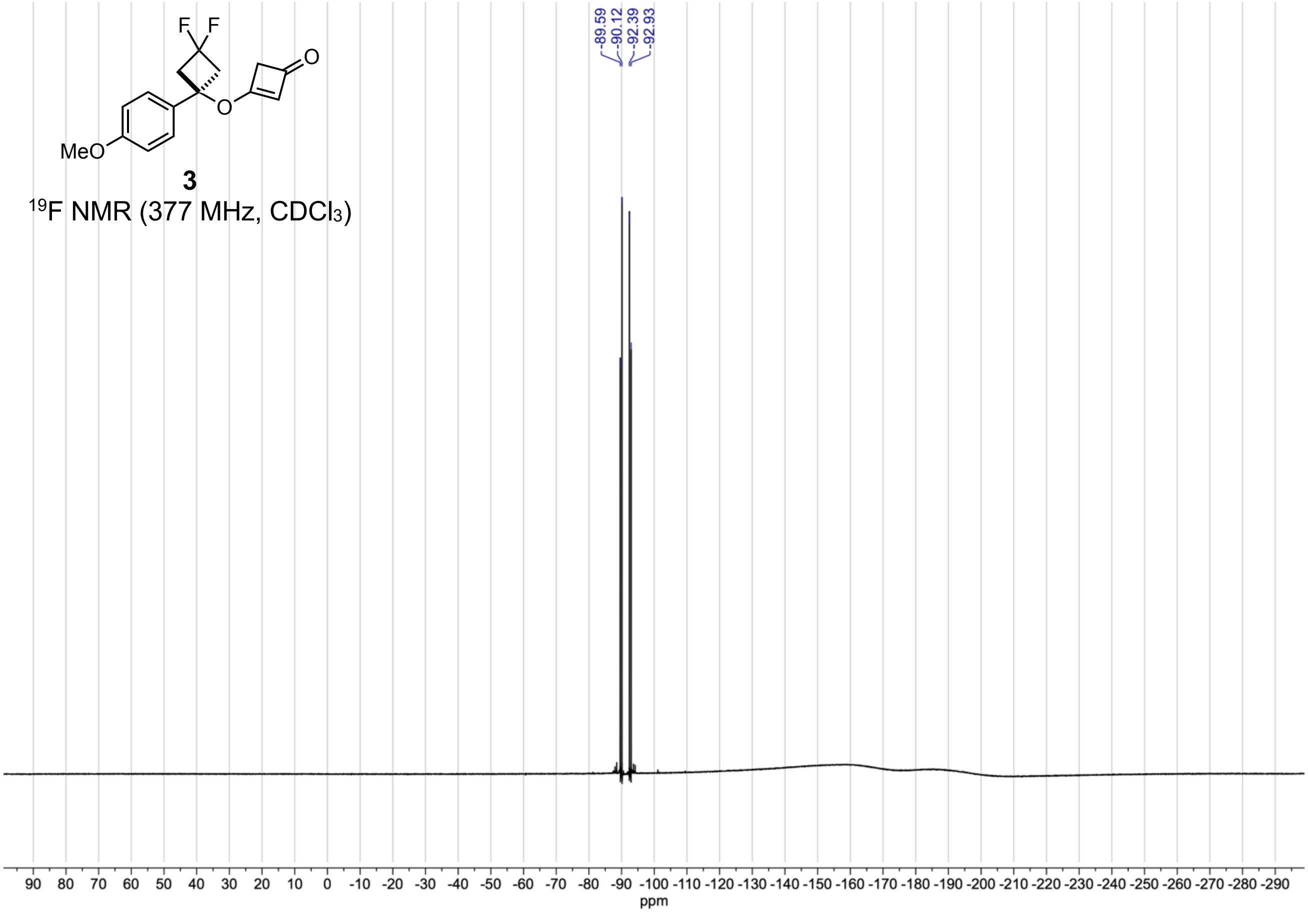

3

^{19}F NMR (377 MHz, $CDCl_3$)

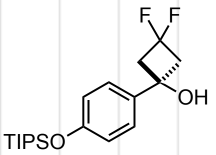

4

^{1}H NMR (400 MHz, $CDCl_3$)

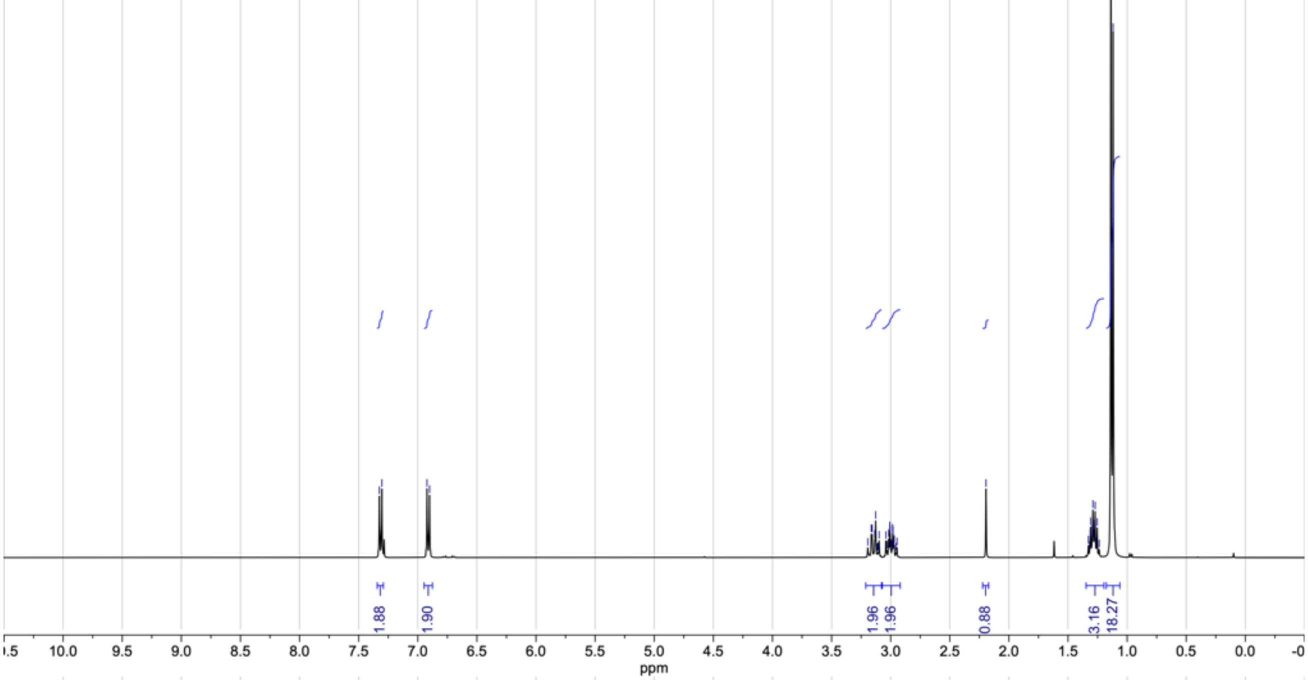

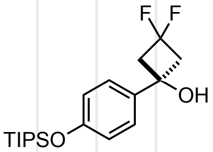

4

^{13}C{^{1}H} NMR (101 MHz, $CDCl_3$)

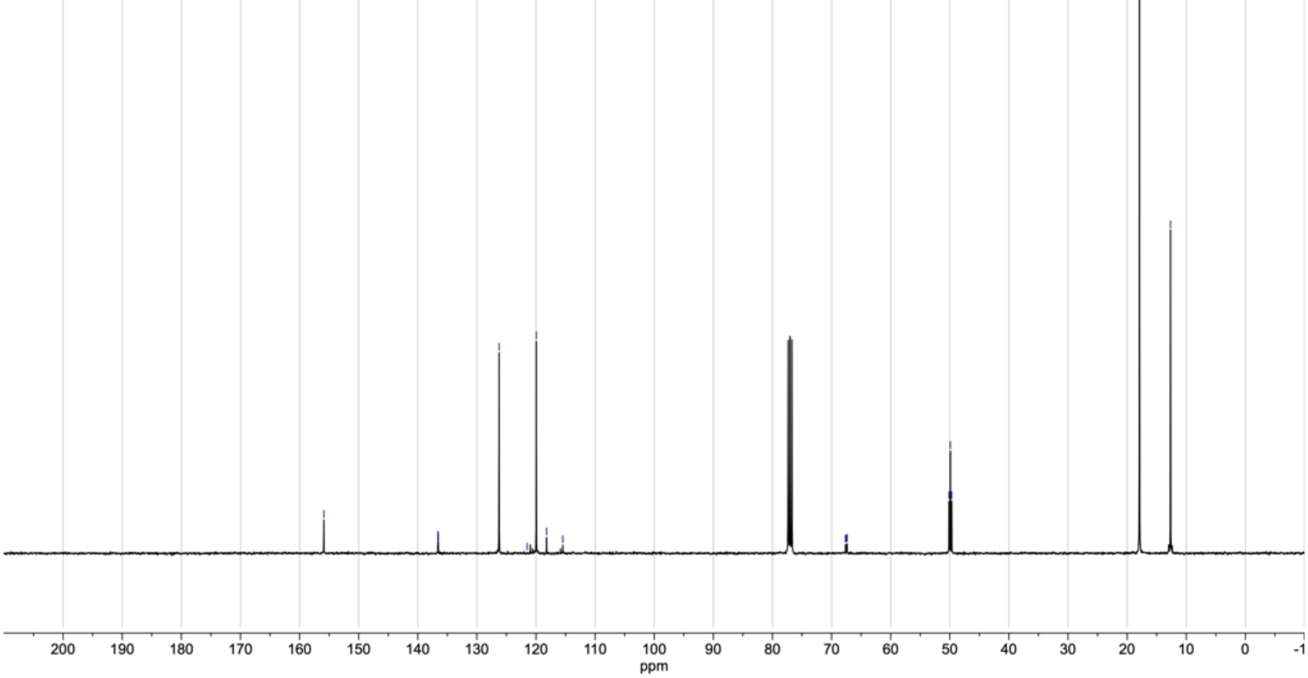

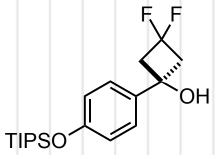

4

^{19}F NMR (377 MHz, $CDCl_3$)

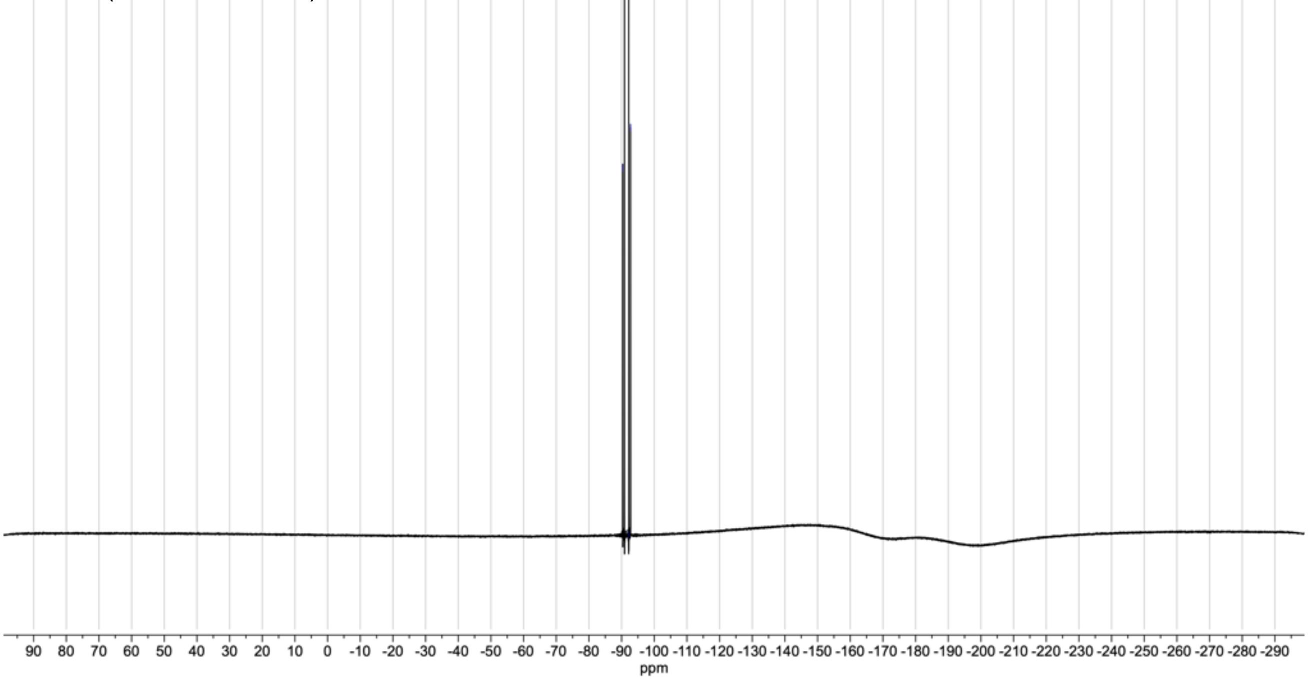

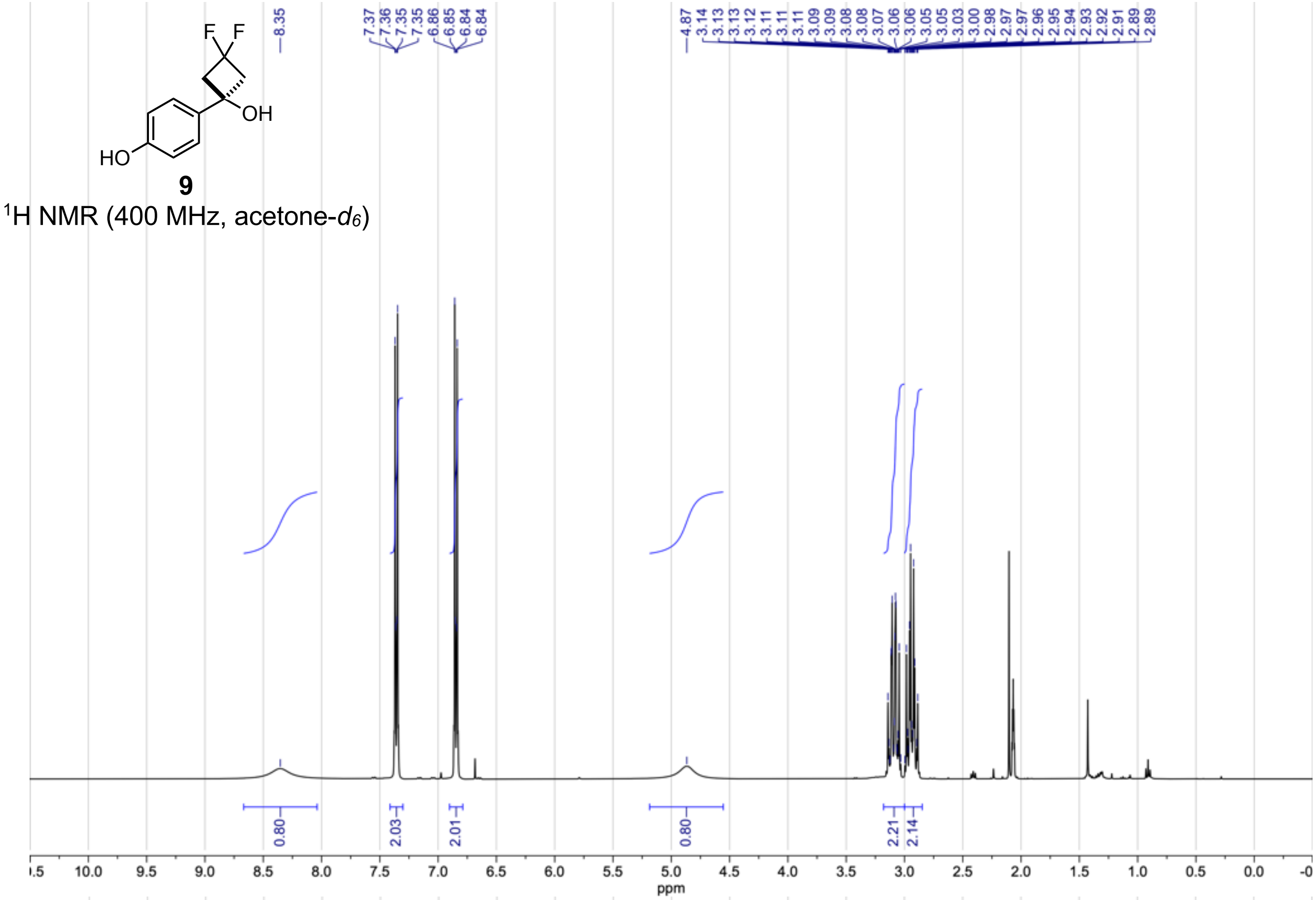

9
^{1}H NMR (400 MHz, acetone-d_6)
8.35
7.37 7.36 7.35 7.35 6.86 6.85 6.84 6.84
4.87 3.14 3.13 3.13 3.12 3.11 3.11 3.11 3.09 3.09 3.08 3.08 3.07 3.06 3.06 3.05 3.05 3.03 3.00 2.98 2.97 2.97 2.96 2.95 2.94 2.93 2.92 2.91 2.89 2.89
0.80 2.03 2.01 0.80 2.21 2.14
ppm

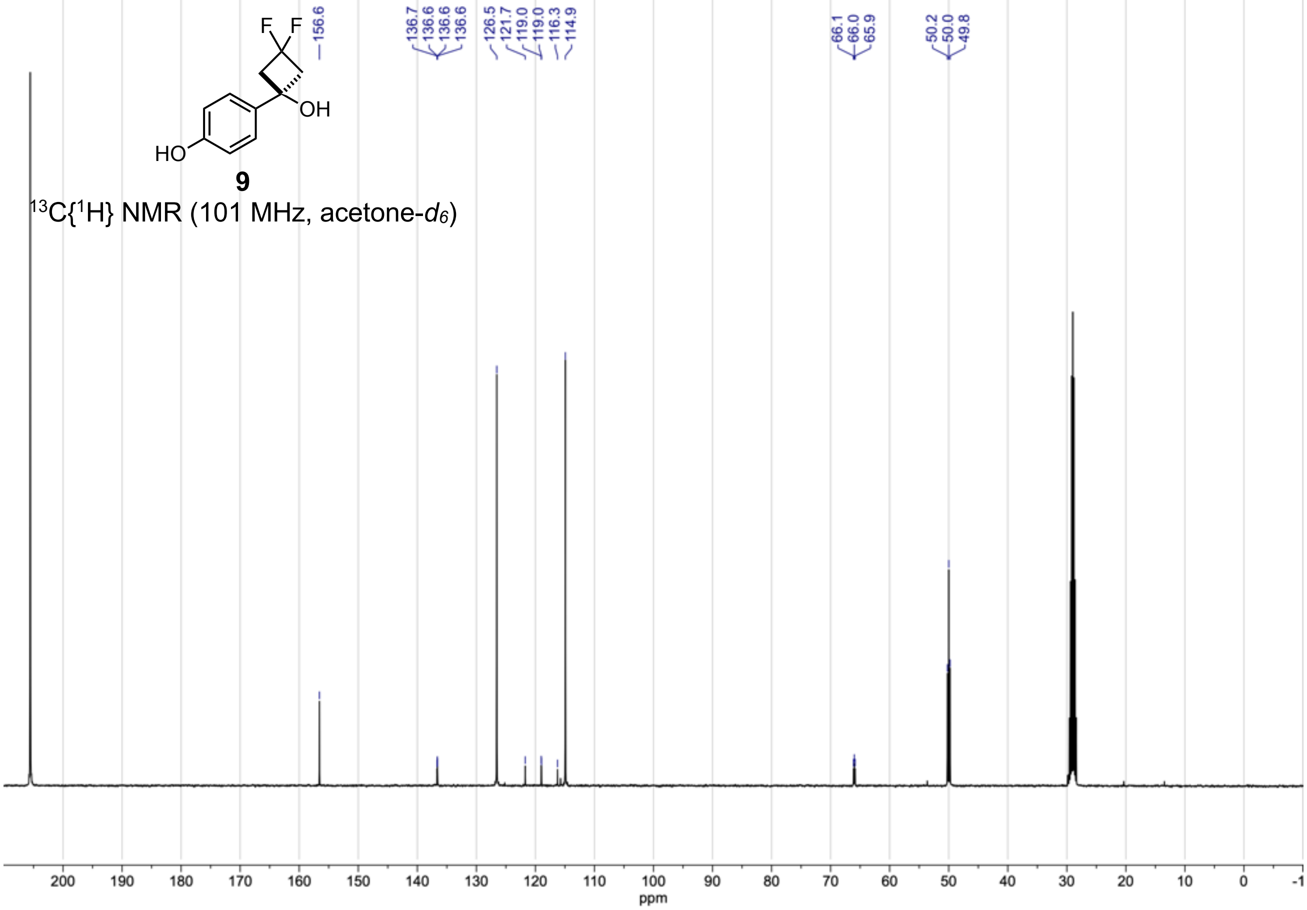

9
^{13}C{^{1}H} NMR (101 MHz, acetone-d_6)
156.6
136.7 136.6 136.6 136.6
126.5 121.7 119.0 119.0 116.3 114.9
66.1 66.0 65.9
50.2 50.0 49.8
ppm

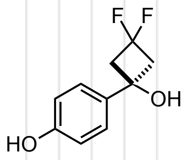

9

^{19}F NMR (377 MHz, acetone-d_6)

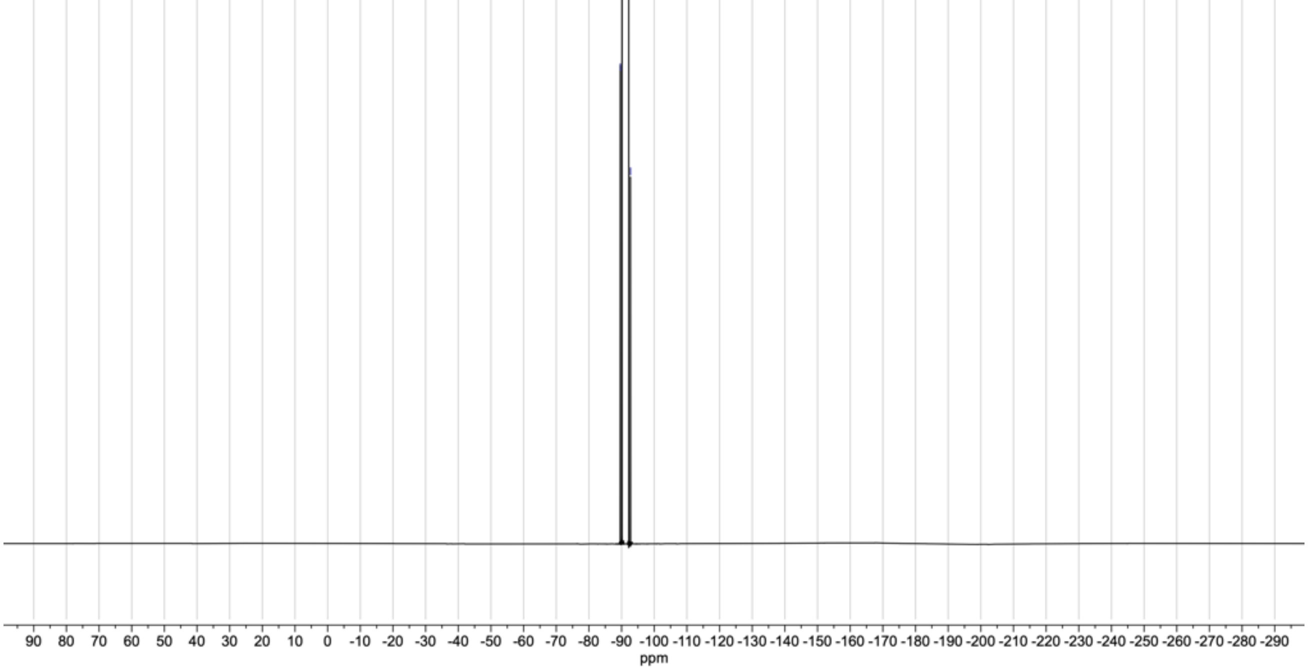

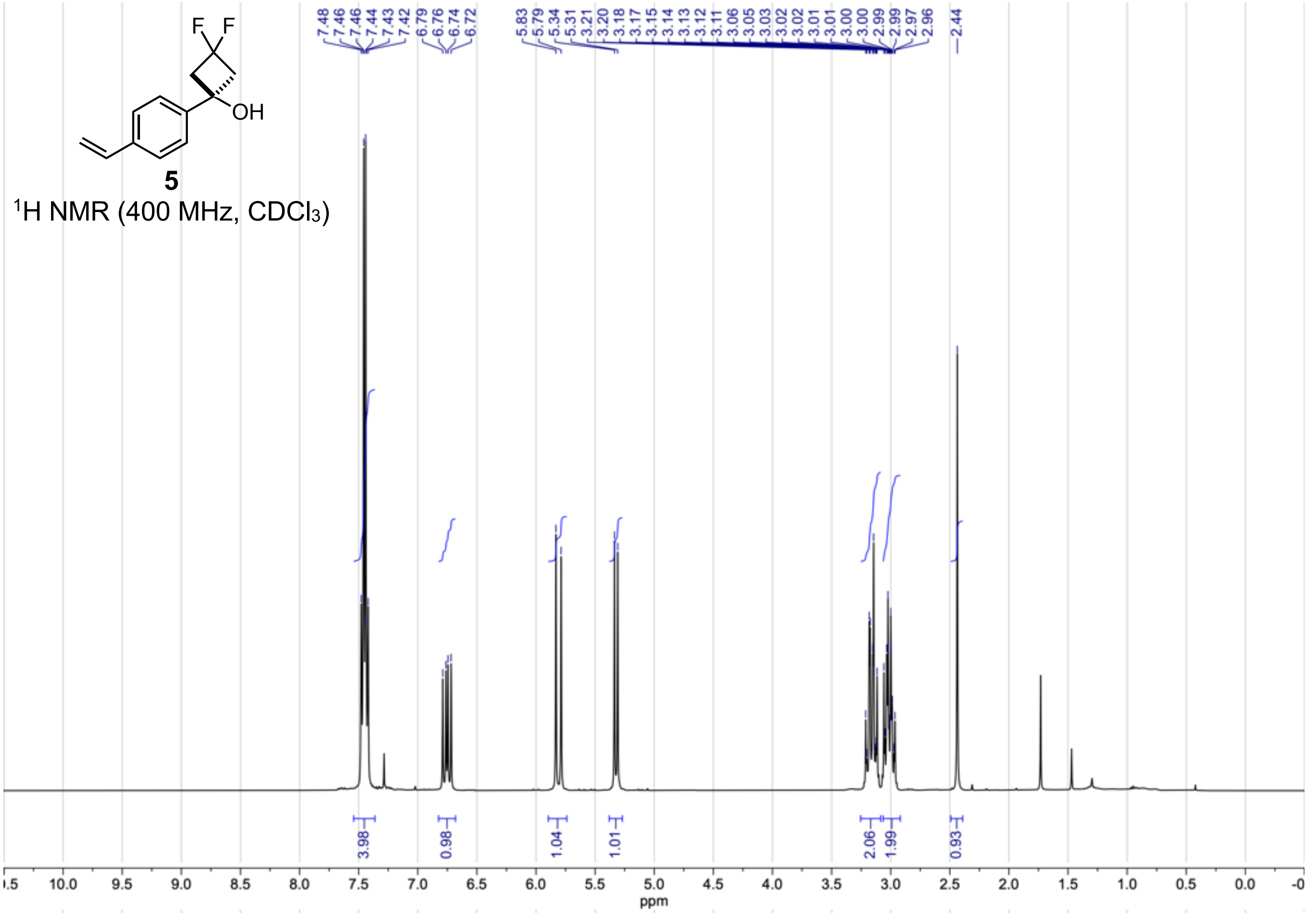
F F
OH
5
1H NMR (400 MHz, CDCl3)
7.48 7.46 7.46 7.44 7.43 7.42 6.79 6.76 6.74 6.72
5.83 5.79 5.34 5.31 3.21 3.20 3.18 3.17 3.15 3.14 3.13 3.12 3.11 3.06 3.05 3.03 3.02 3.02 3.01 3.01 3.00 3.00 2.99 2.99 2.97 2.96
2.44
3.98 0.98 1.04 1.01 2.06 1.99 0.93
ppm

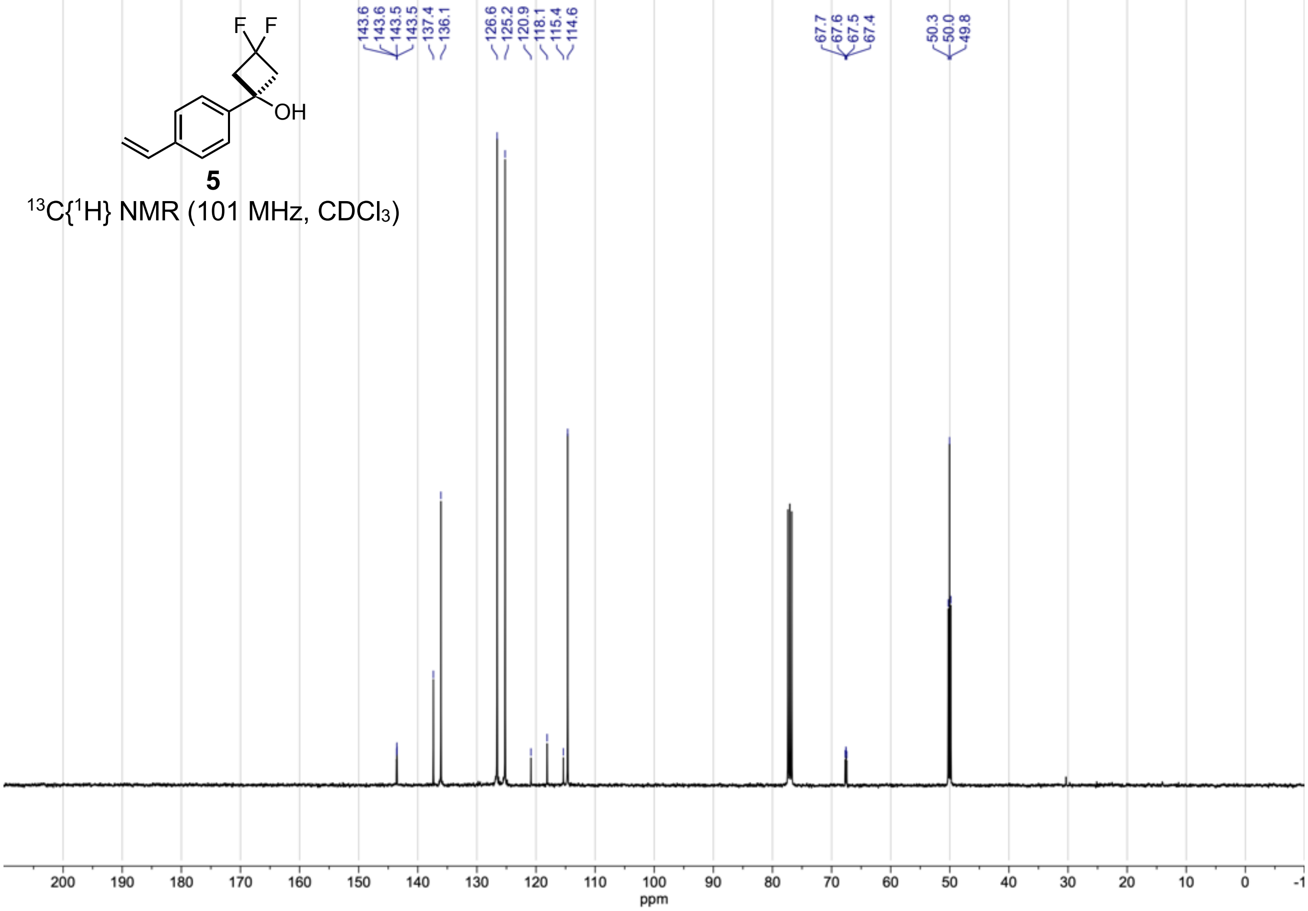
F F
OH
5
13C{1H} NMR (101 MHz, CDCl3)
143.6 143.6 143.5 143.5 137.4 136.1
126.6 125.2 120.9 118.1 115.4 114.6
67.7 67.6 67.5 67.4
50.3 50.0 49.8
ppm

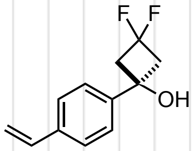

5

^{19}F NMR (377 MHz, $CDCl_3$)

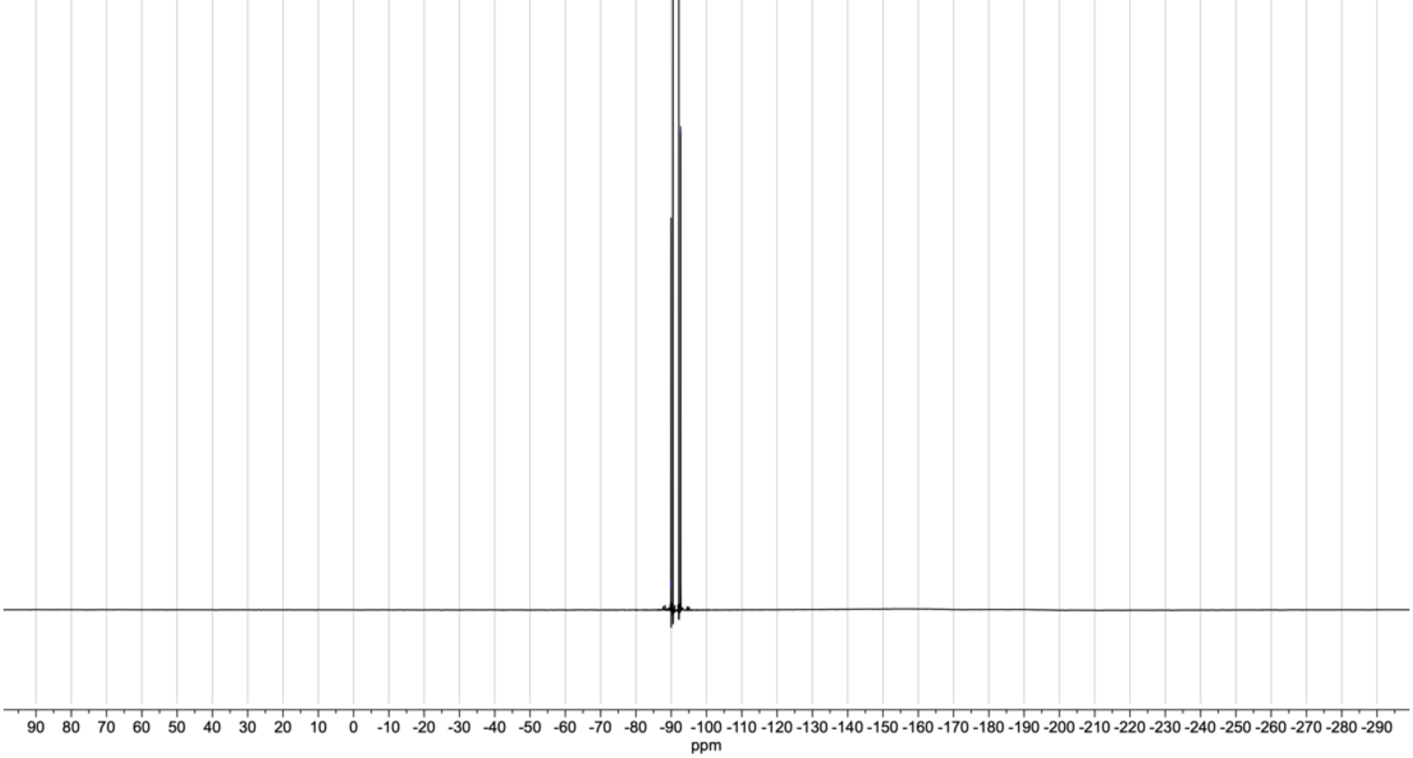

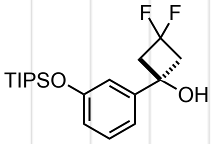

6

^{1}H NMR (400 MHz, $CDCl_3$)

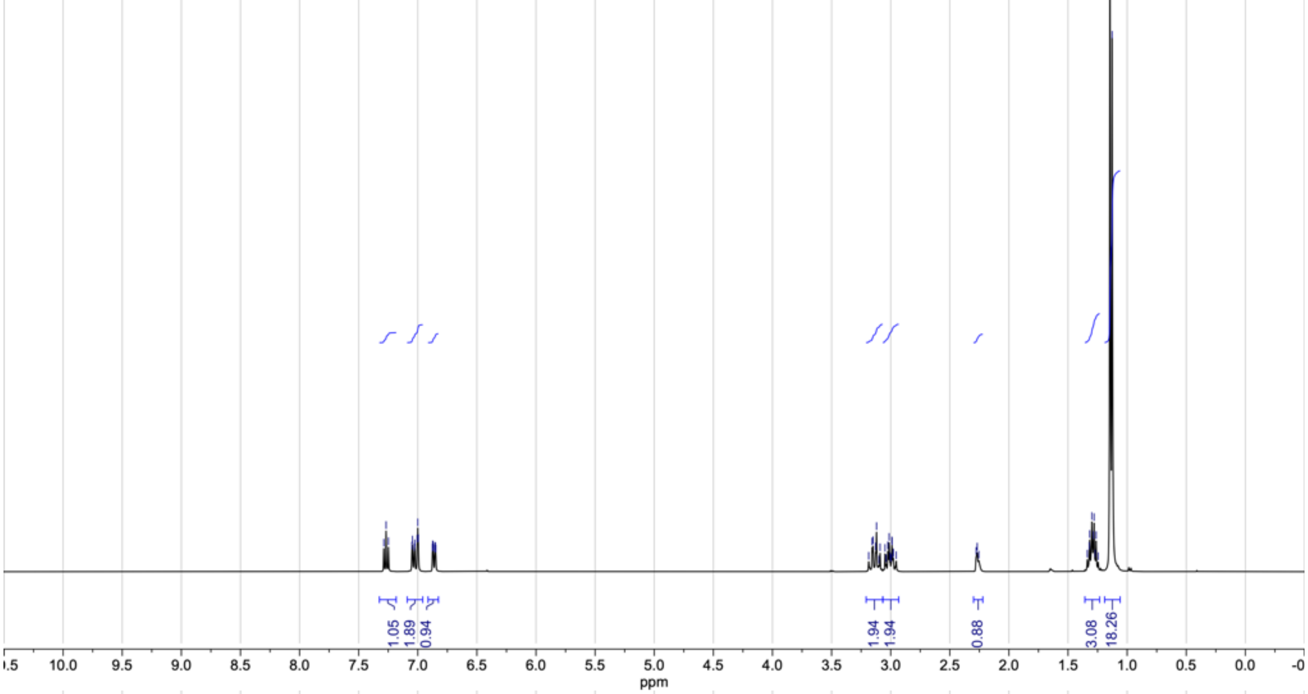

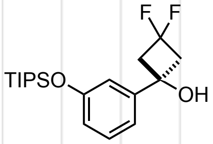

6

$^{13}C\{^1H\}$ NMR (101 MHz, $CDCl_3$)

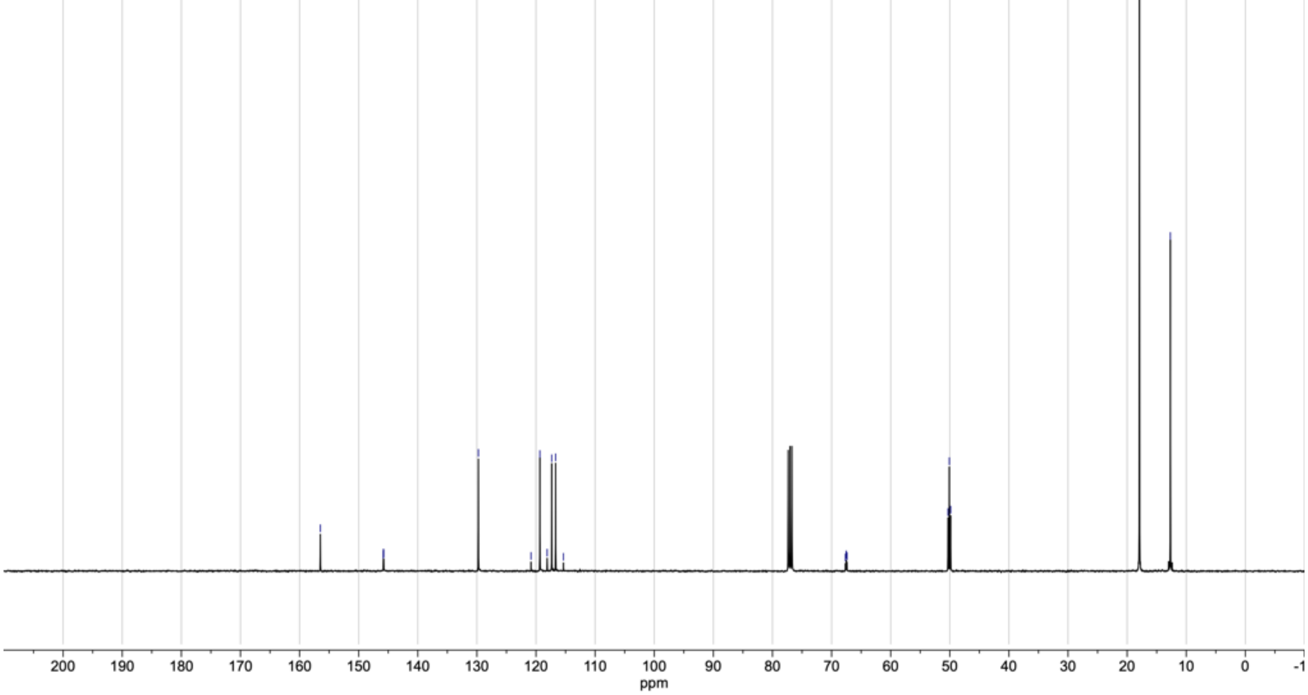

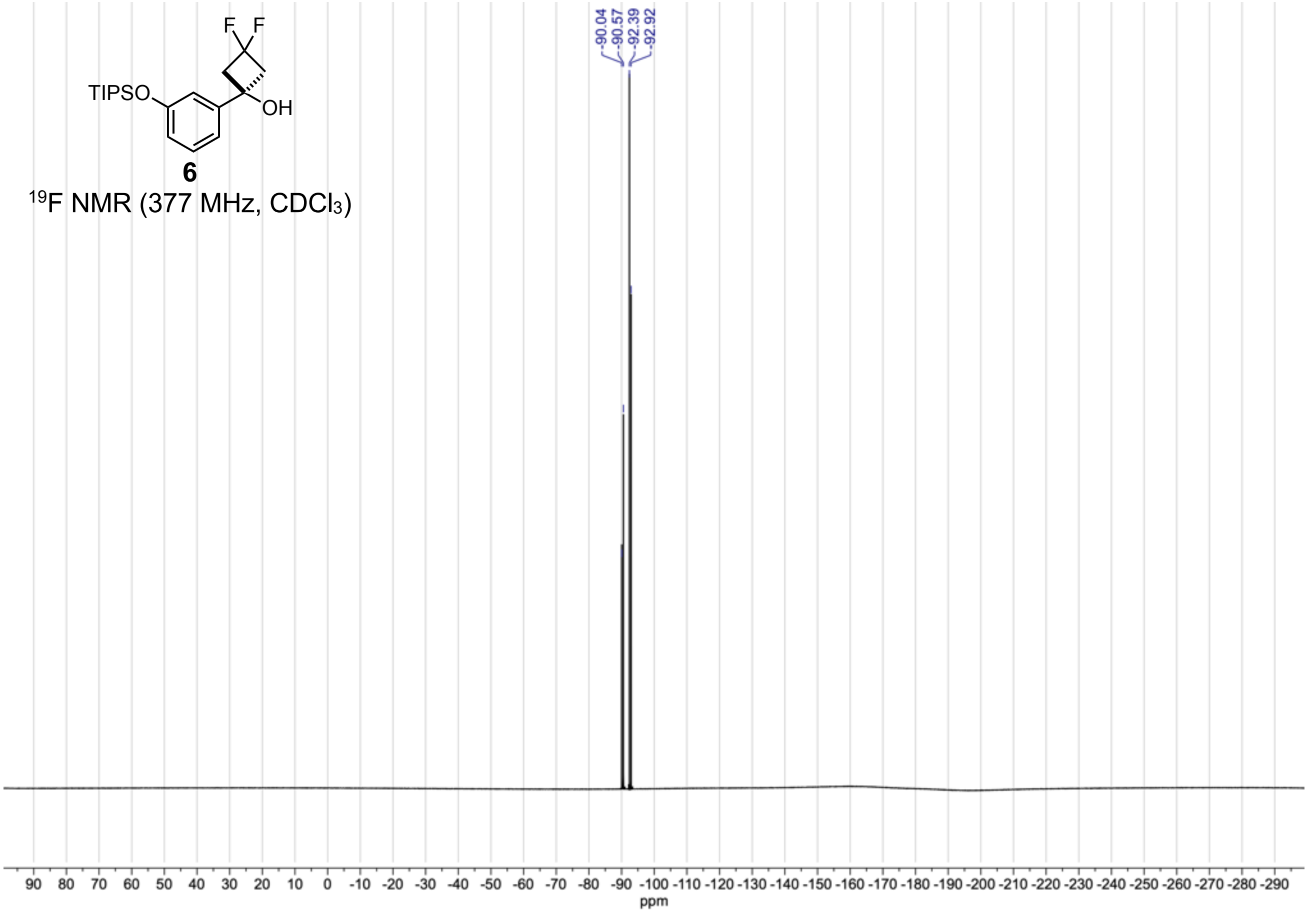
TIPSO
F
F
OH
6
19F NMR (377 MHz, CDCl3)
-90.04
-90.57
-92.39
-92.92
ppm

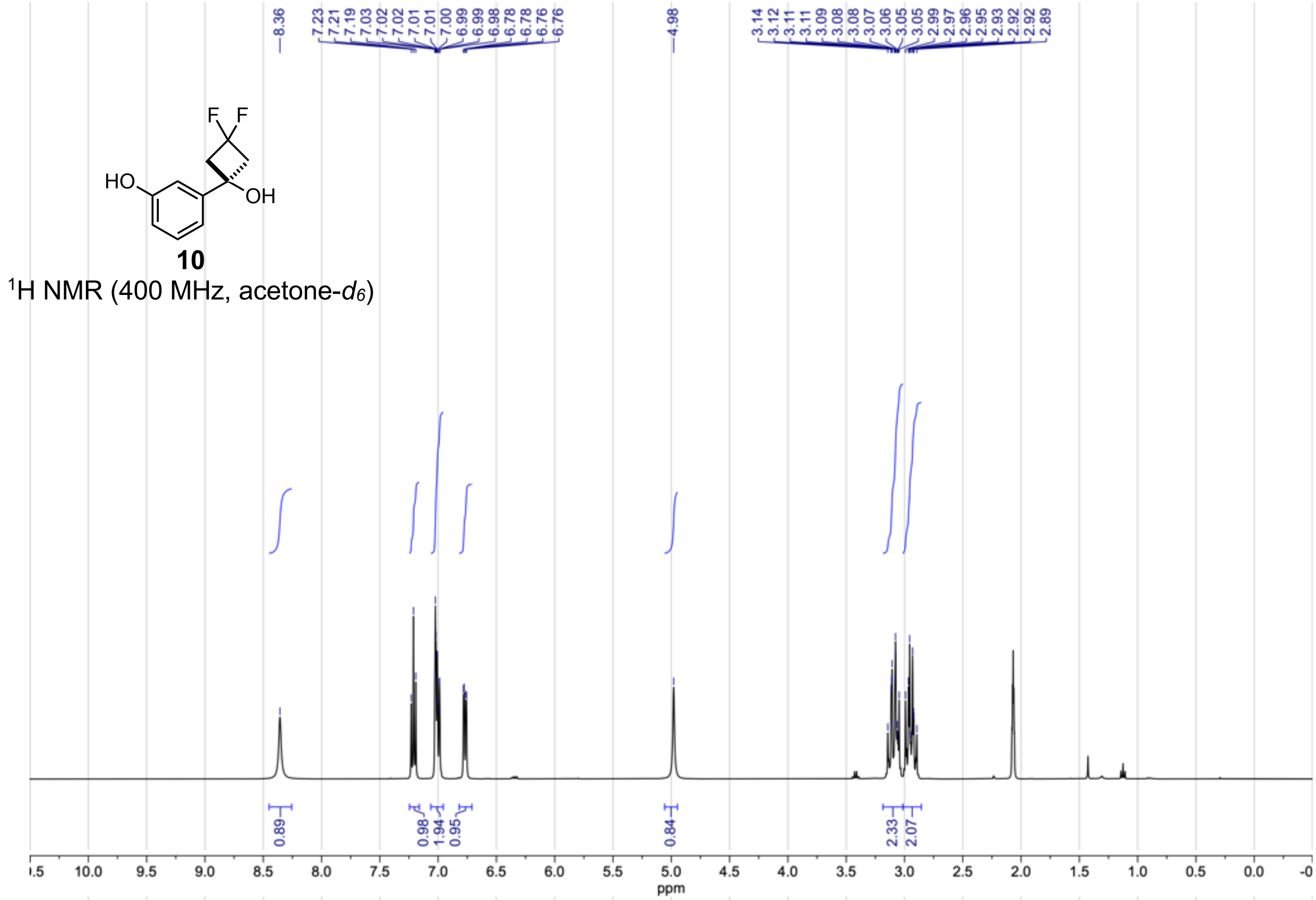
8.36
7.23
7.21
7.19
7.03
7.02
7.02
7.01
7.00
6.99
6.99
6.98
6.78
6.78
6.76
6.76
4.98
3.14
3.12
3.11
3.11
3.09
3.08
3.08
3.07
3.06
3.05
3.05
2.99
2.97
2.96
2.95
2.93
2.92
2.89
HO
F
F
OH
10
^{1}H NMR (400 MHz, acetone-d_6)
0.89
0.98
1.94
0.95
0.84
2.33
2.07
ppm

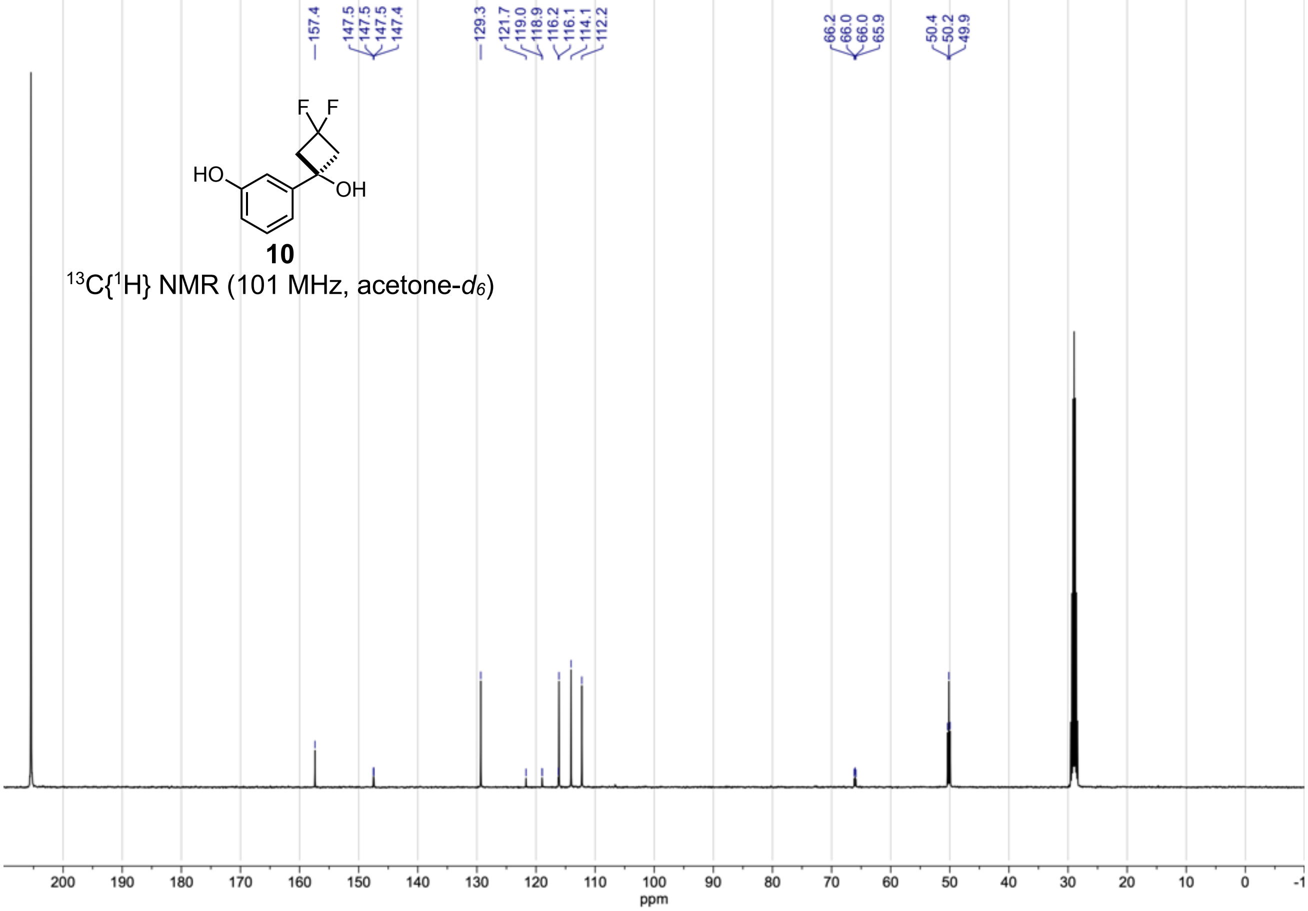
157.4
147.5
147.5
147.5
147.4
129.3
121.7
119.0
118.9
116.2
116.1
114.1
112.2
66.2
66.0
66.0
65.9
50.4
50.2
49.9
HO
F
F
OH
10
^{13}C{^{1}H} NMR (101 MHz, acetone-d_6)
ppm

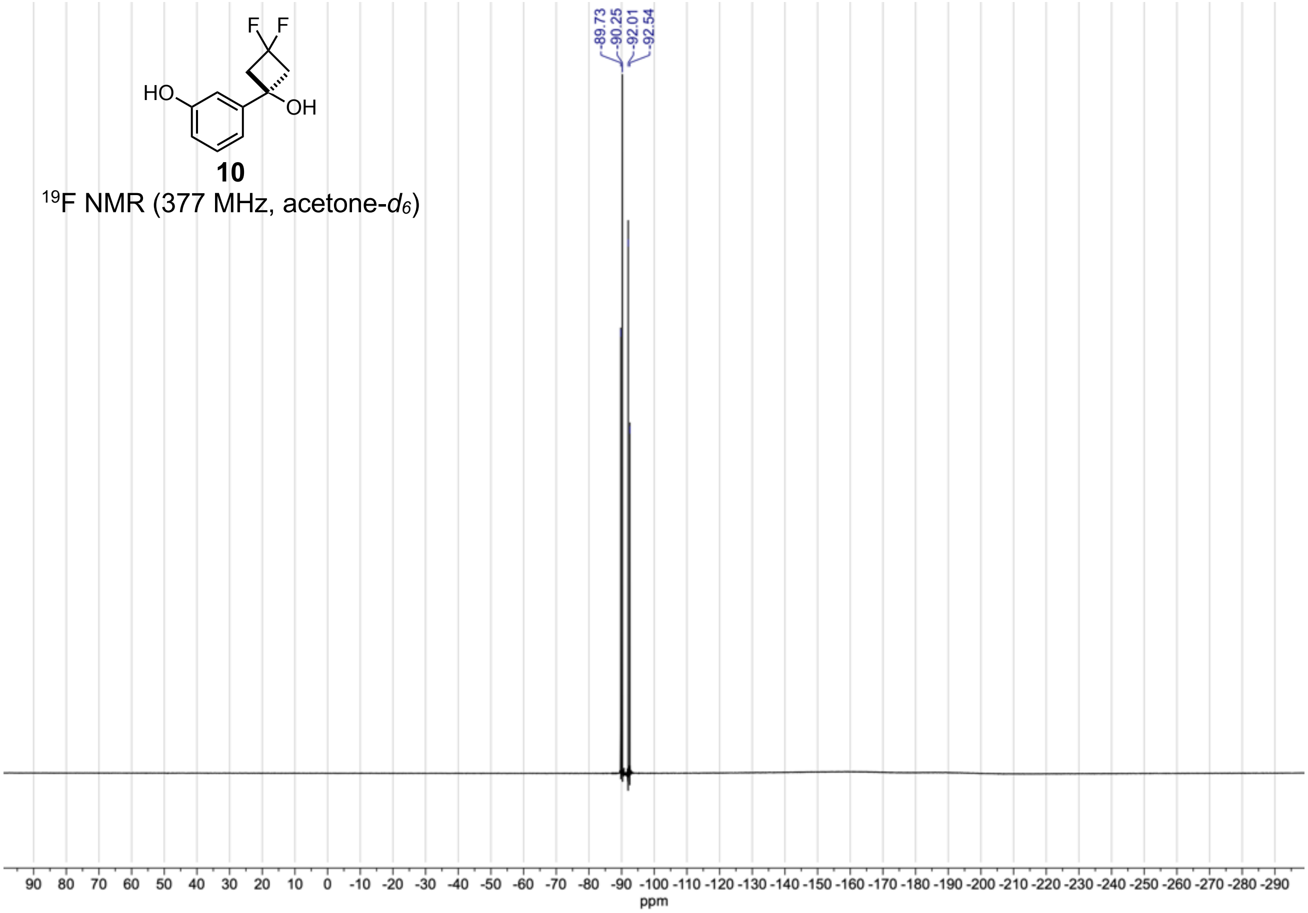

10

^{19}F NMR (377 MHz, acetone-d_6)

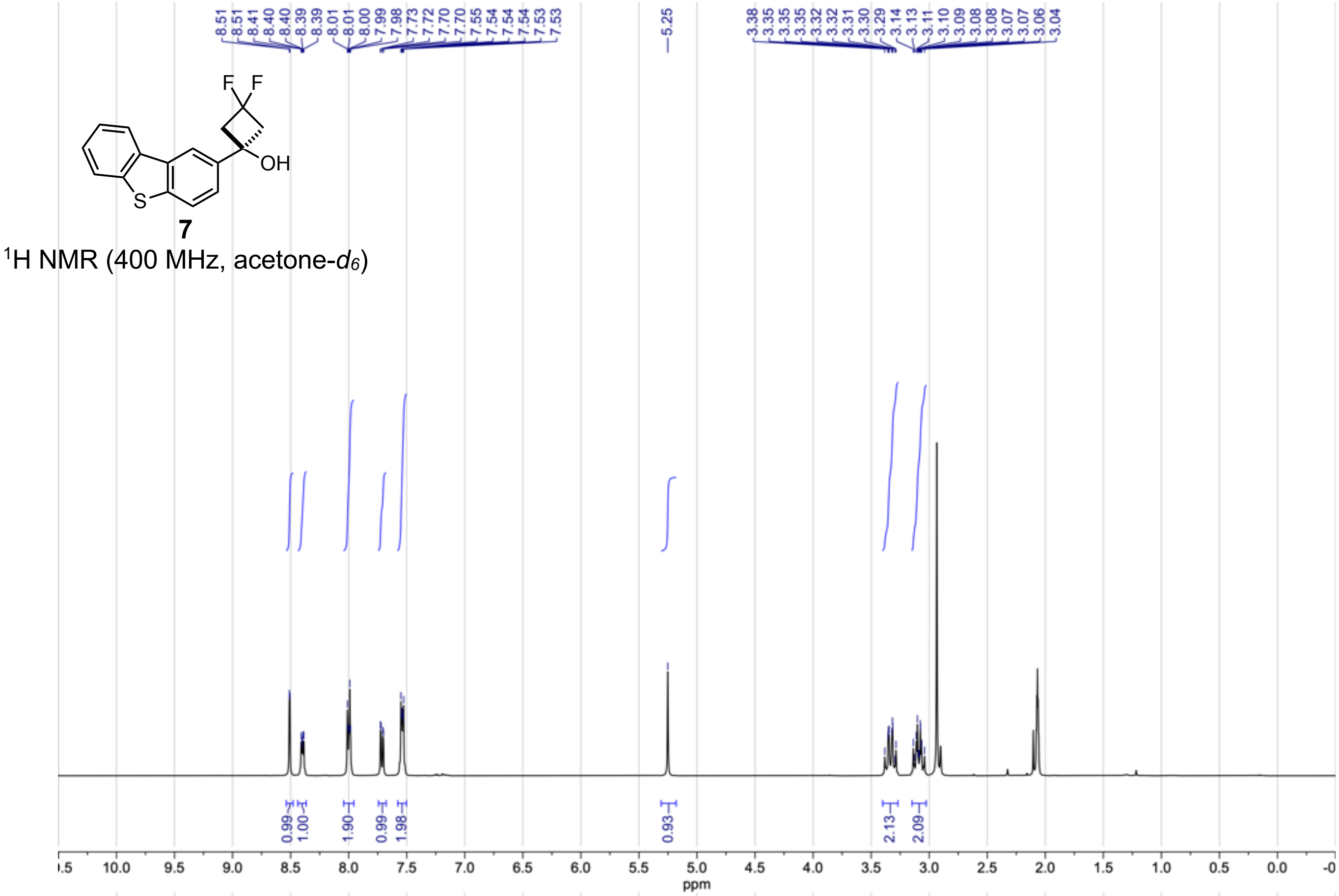

7
^{1}H NMR (400 MHz, acetone-d_6)
ppm

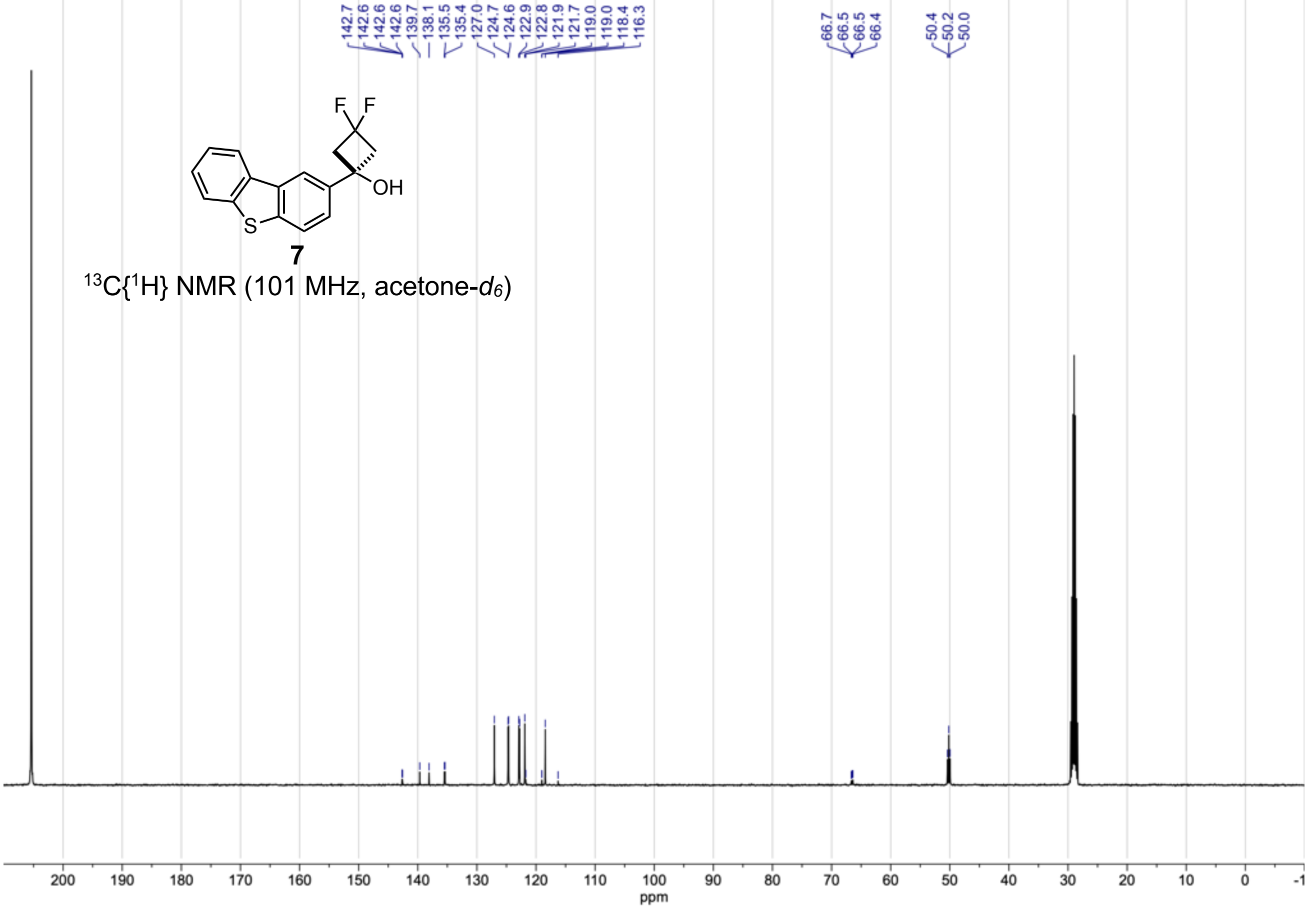

7
^{13}C{^{1}H} NMR (101 MHz, acetone-d_6)
ppm

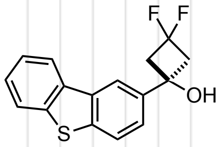

7

^{19}F NMR (377 MHz, acetone-d_6)

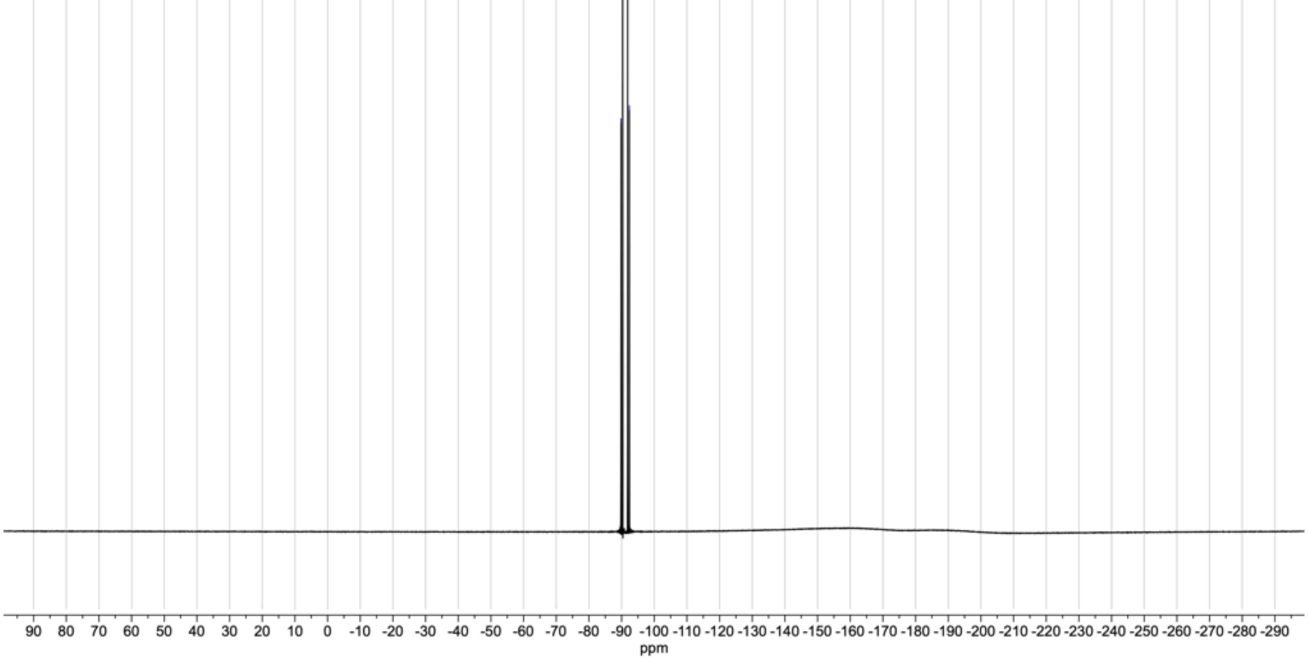

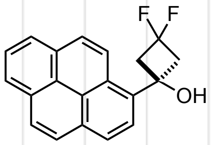

8

^{1}H NMR (400 MHz, $CDCl_3$)

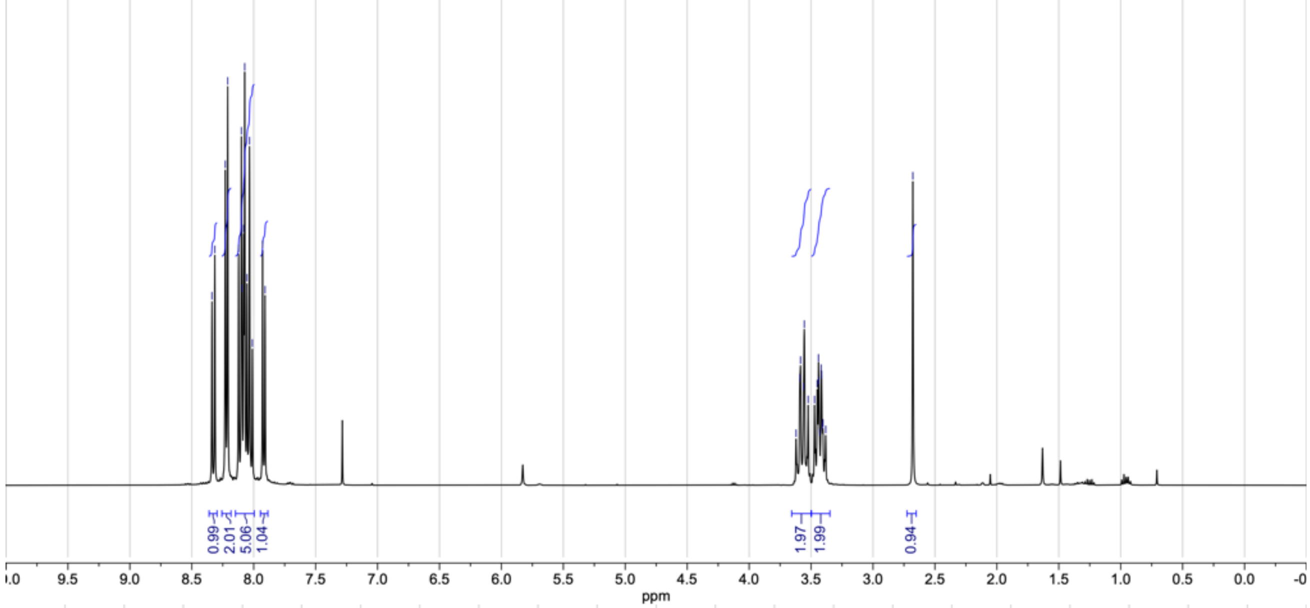

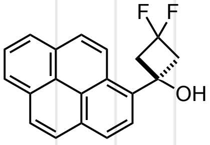

8

$^{13}C\{^{1}H\}$ NMR (101 MHz, $CDCl_3$)

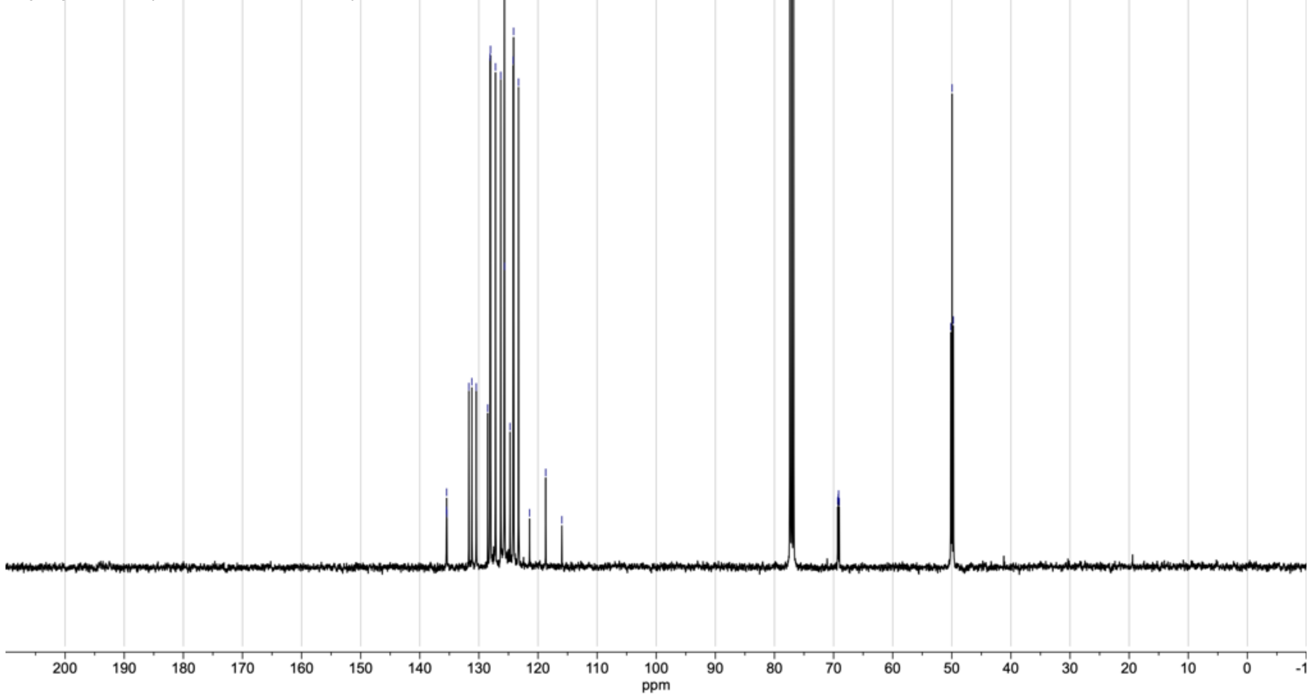

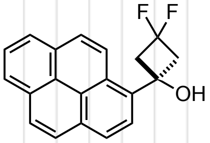

8

^{19}F NMR (377 MHz, $CDCl_3$)

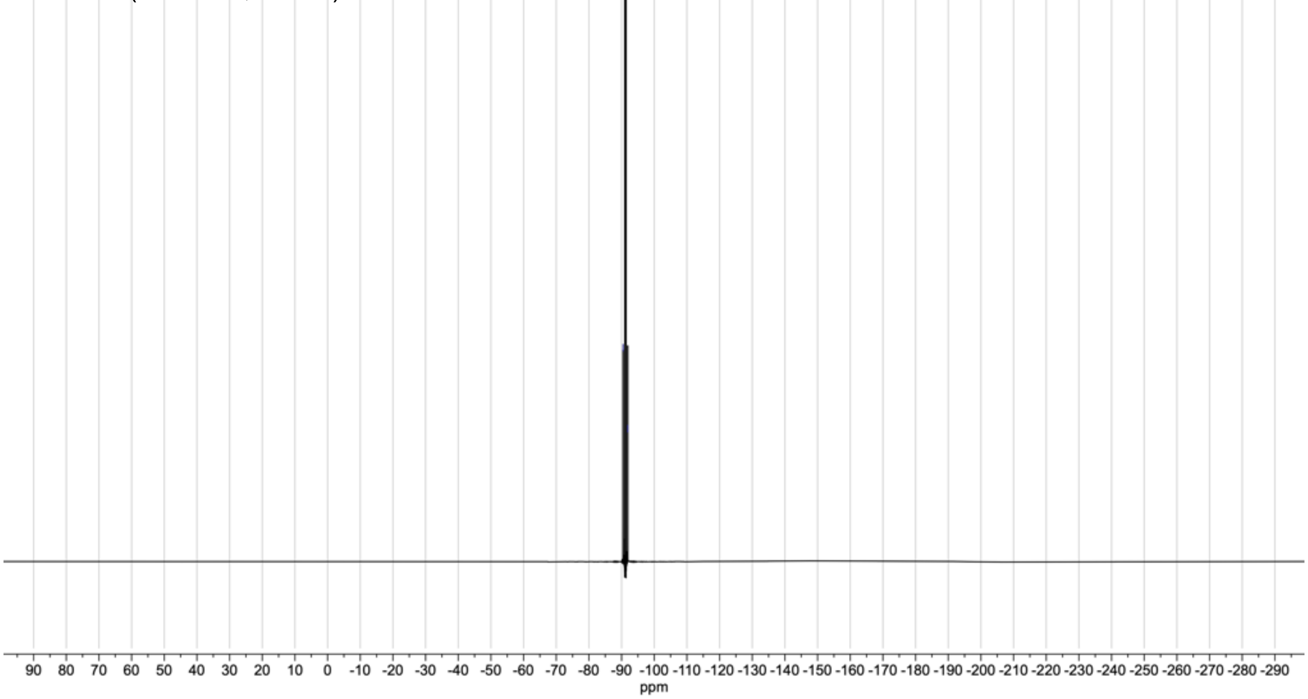

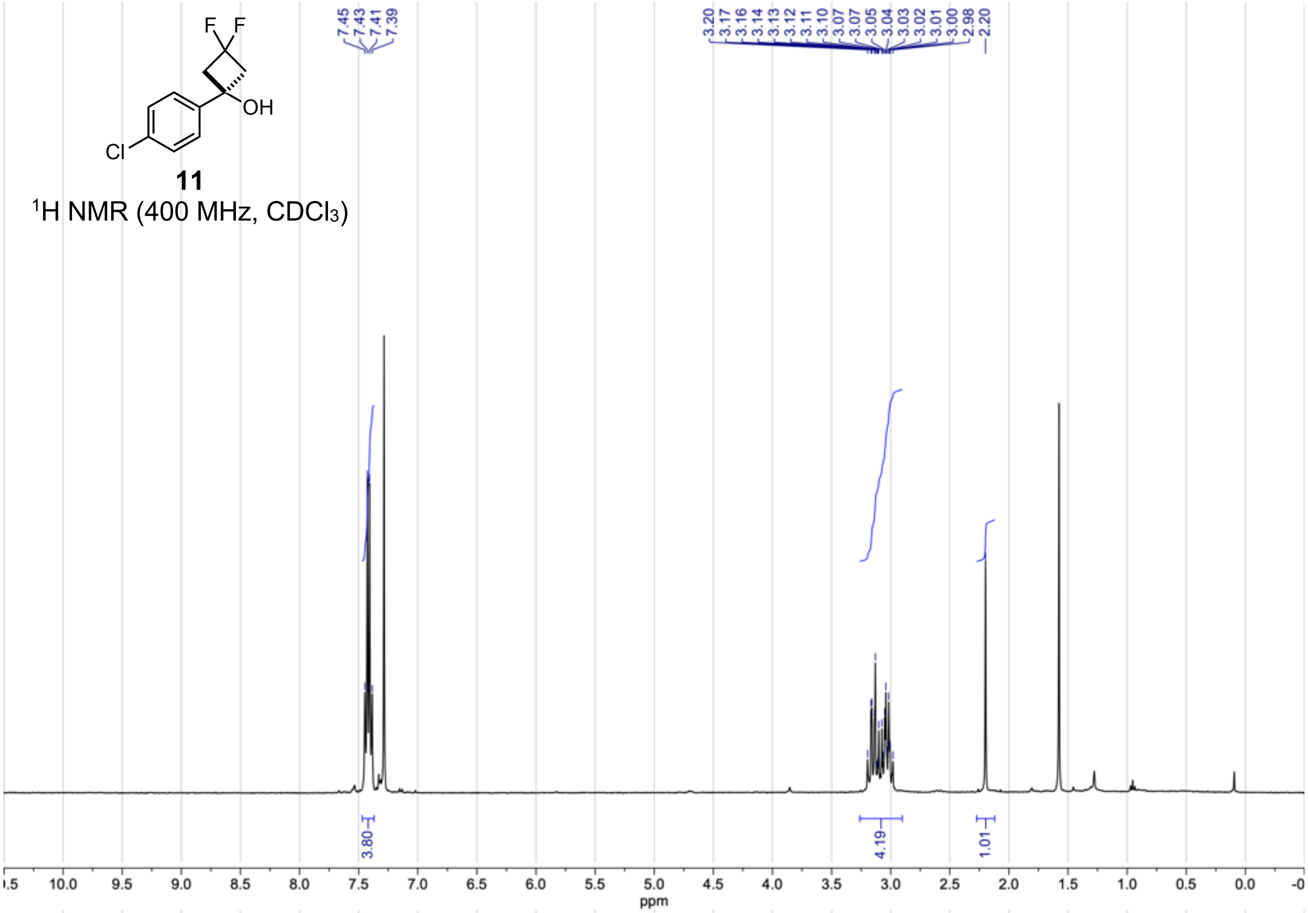

11

^{1}H NMR (400 MHz, $CDCl_3$)

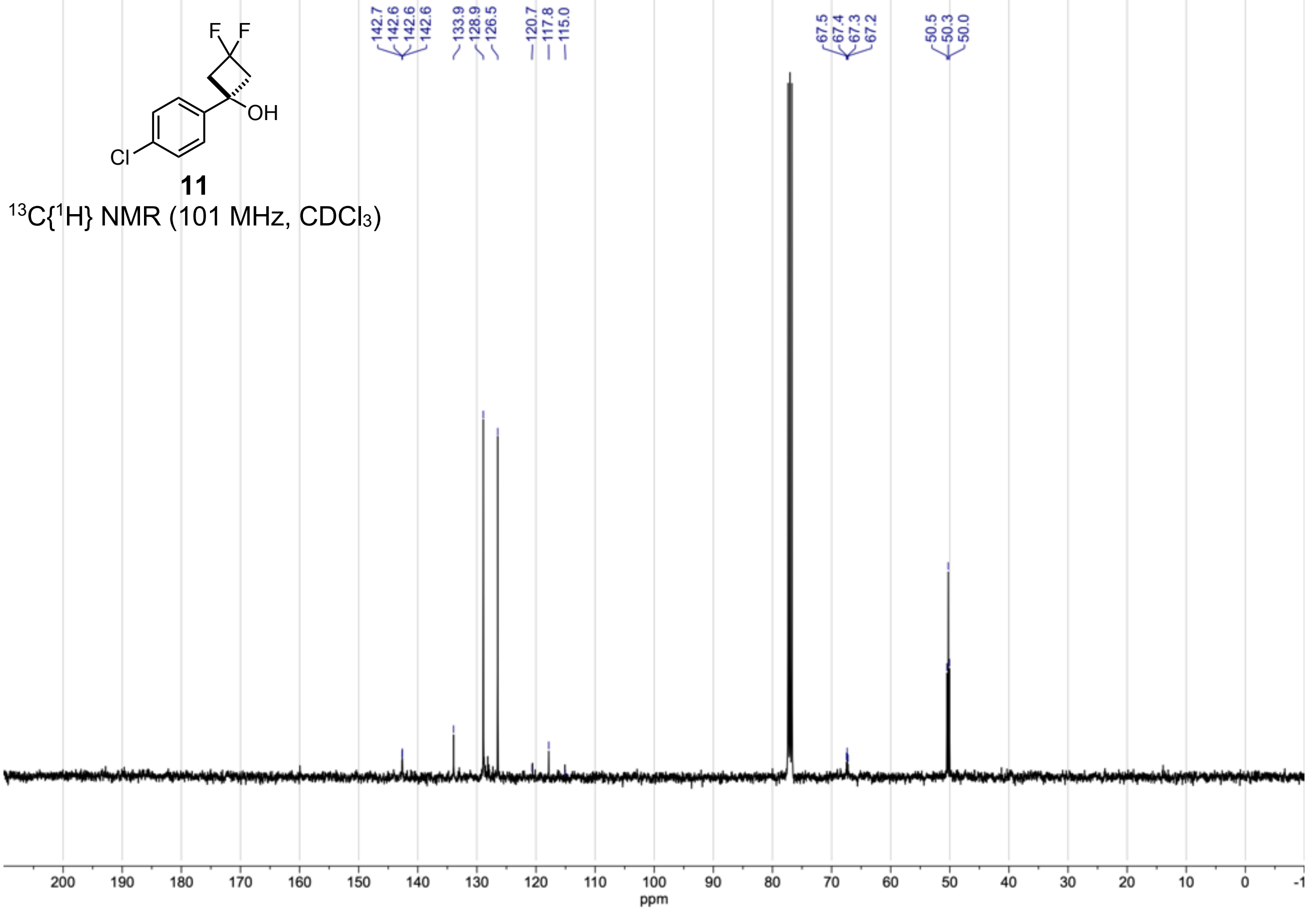

11

$^{13}C\{^1H\}$ NMR (101 MHz, $CDCl_3$)

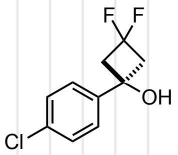

11

^{19}F NMR (377 MHz, $CDCl_3$)

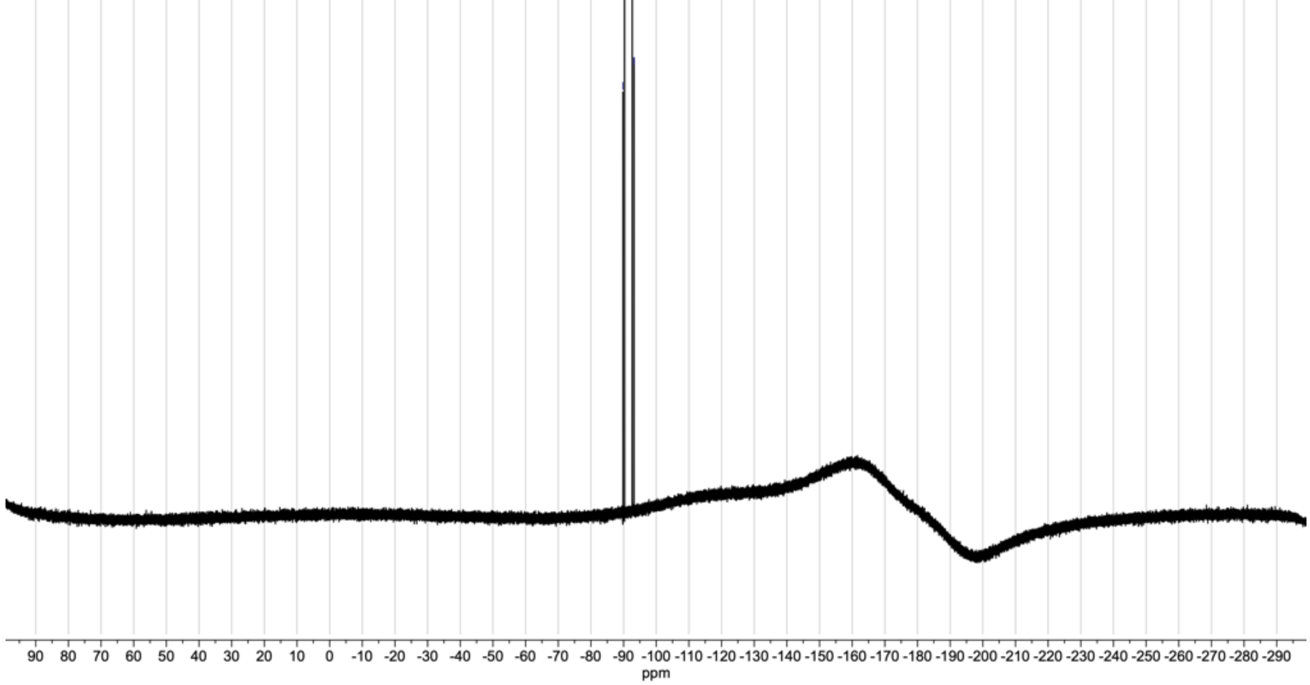

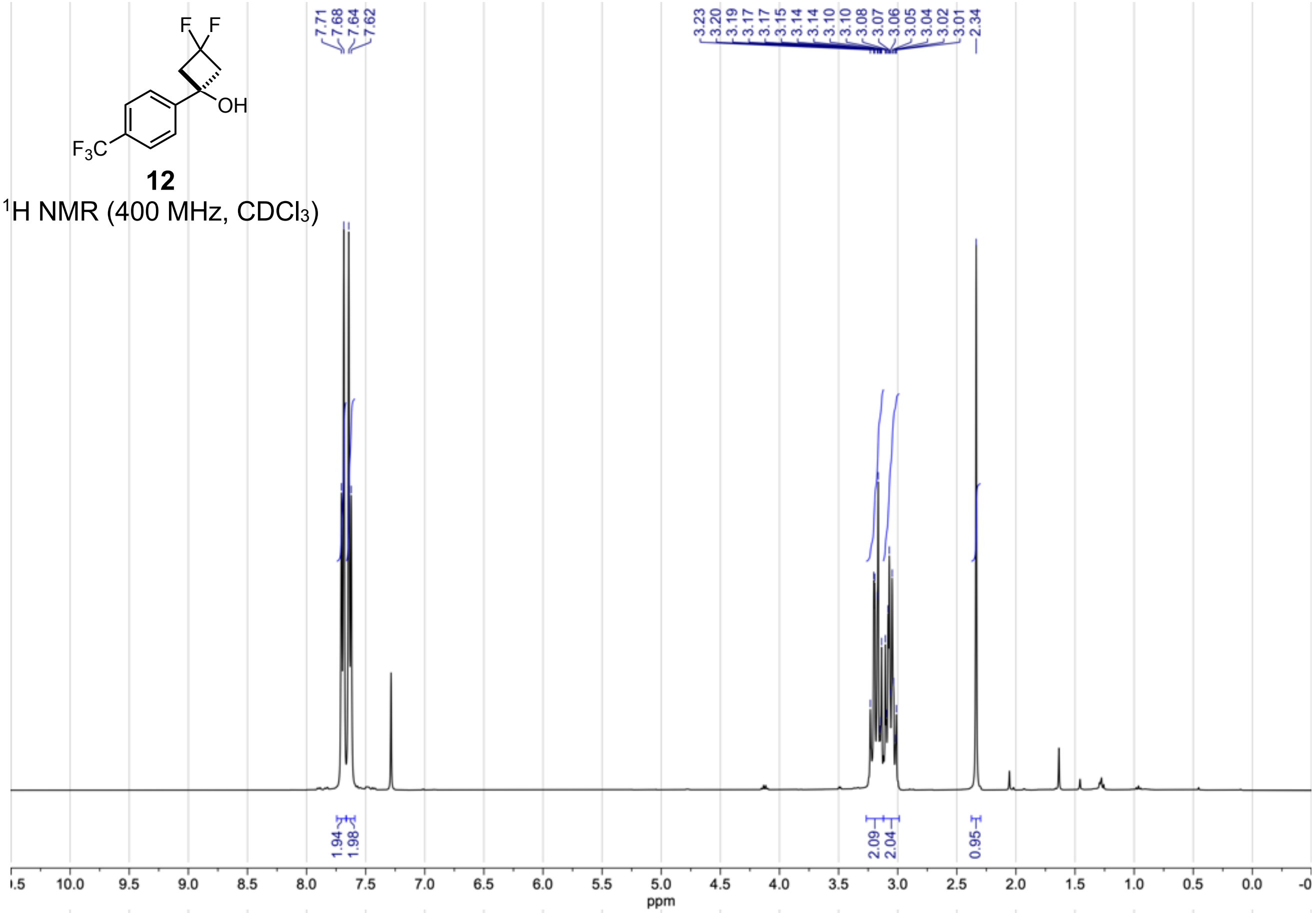
F F
OH
F_3C
12
^{1}H NMR (400 MHz, CDCl$_3$)
7.71 7.68 7.64 7.62
3.23 3.20 3.19 3.17 3.17 3.15 3.14 3.10 3.10 3.08 3.07 3.06 3.05 3.04 3.02 3.01
2.34
1.94 1.98
2.09 2.04
0.95
ppm

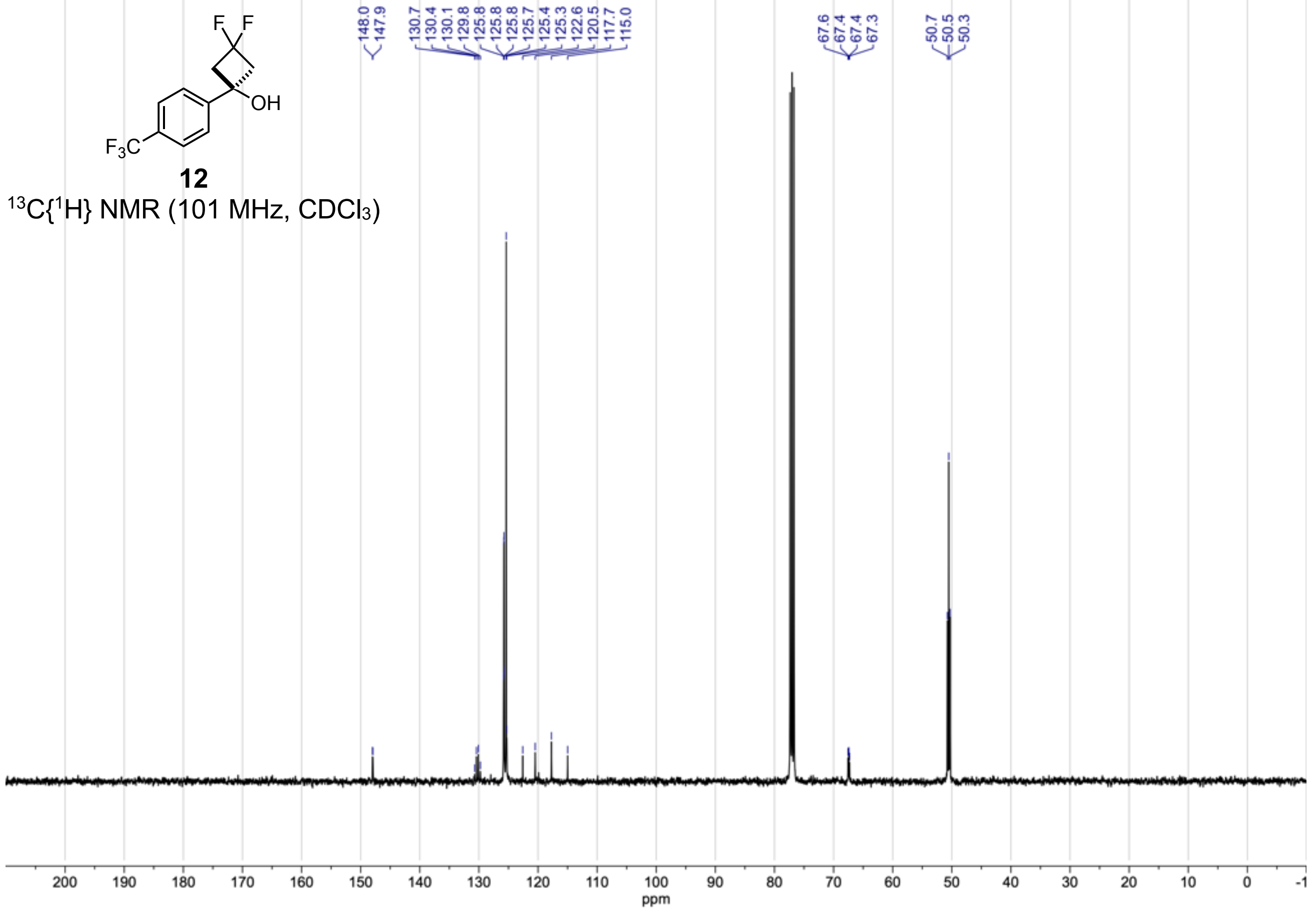
F F
OH
F_3C
12
^{13}C{^{1}H} NMR (101 MHz, CDCl$_3$)
148.0 147.9
130.7 130.4 130.1 129.8 125.8 125.8 125.7 125.4 125.3 122.6 120.5 117.7 115.0
67.6 67.4 67.4 67.3
50.7 50.5 50.3
ppm

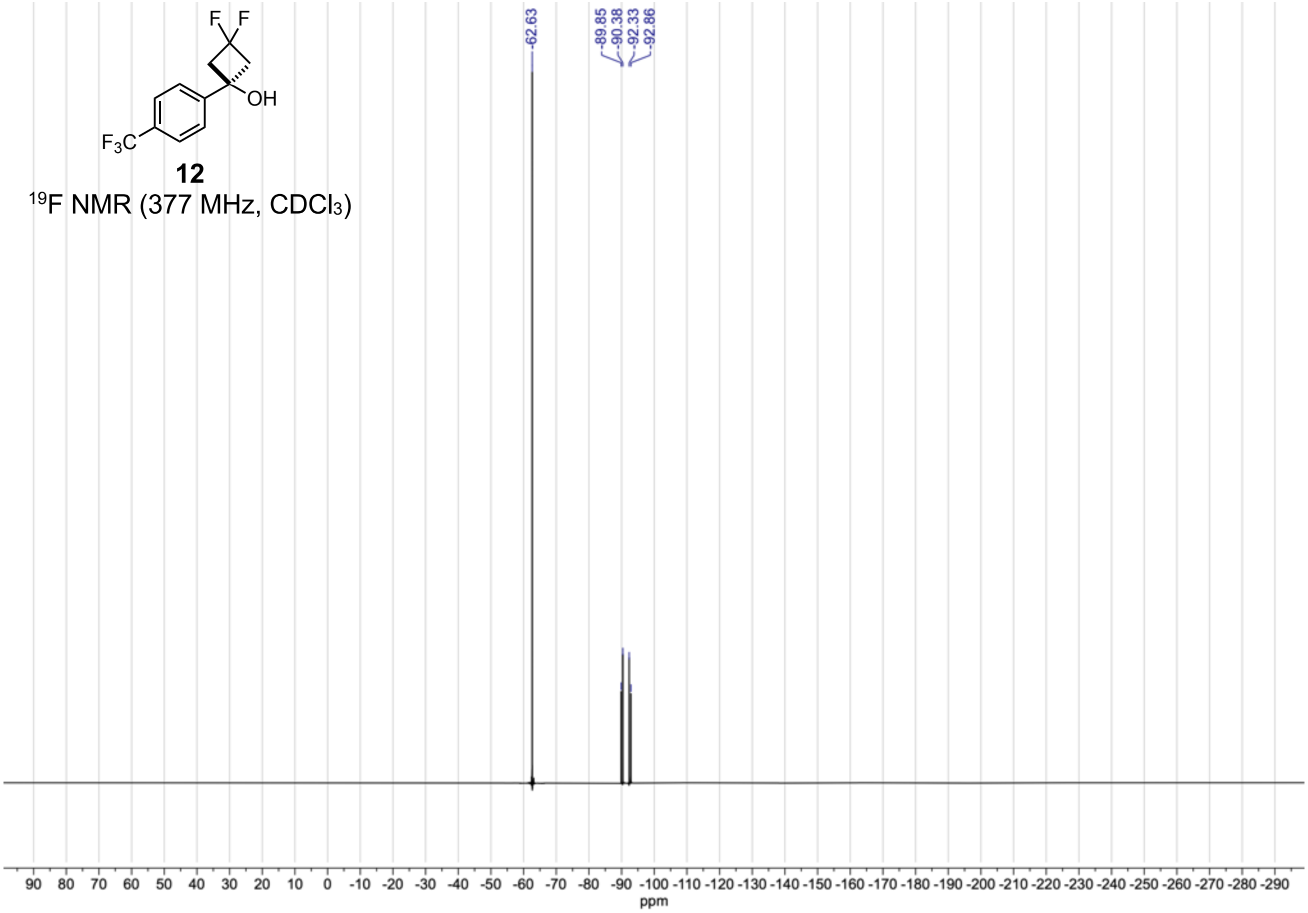

12

^{19}F NMR (377 MHz, $CDCl_3$)

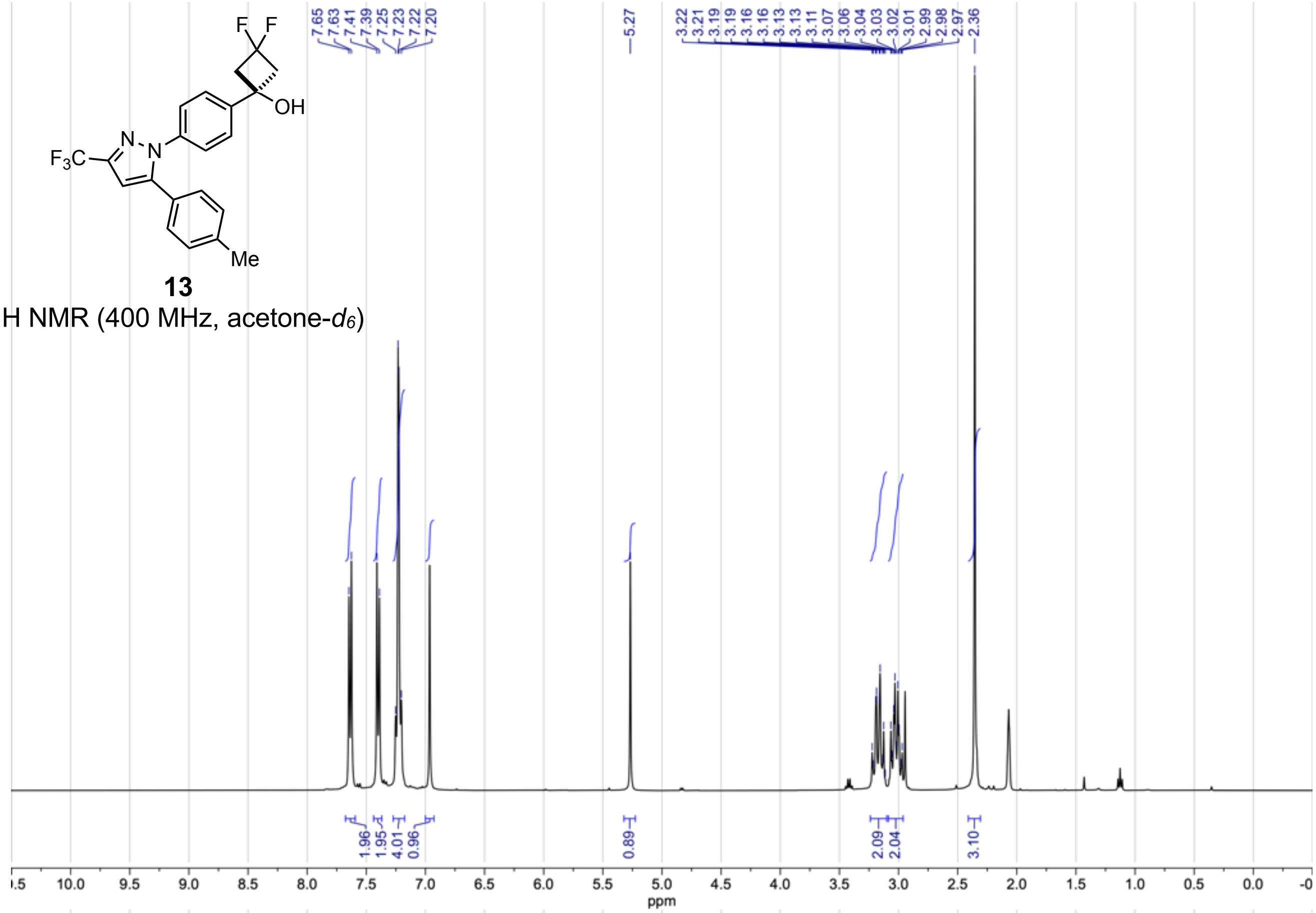

13

^{1}H NMR (400 MHz, acetone-d_6)

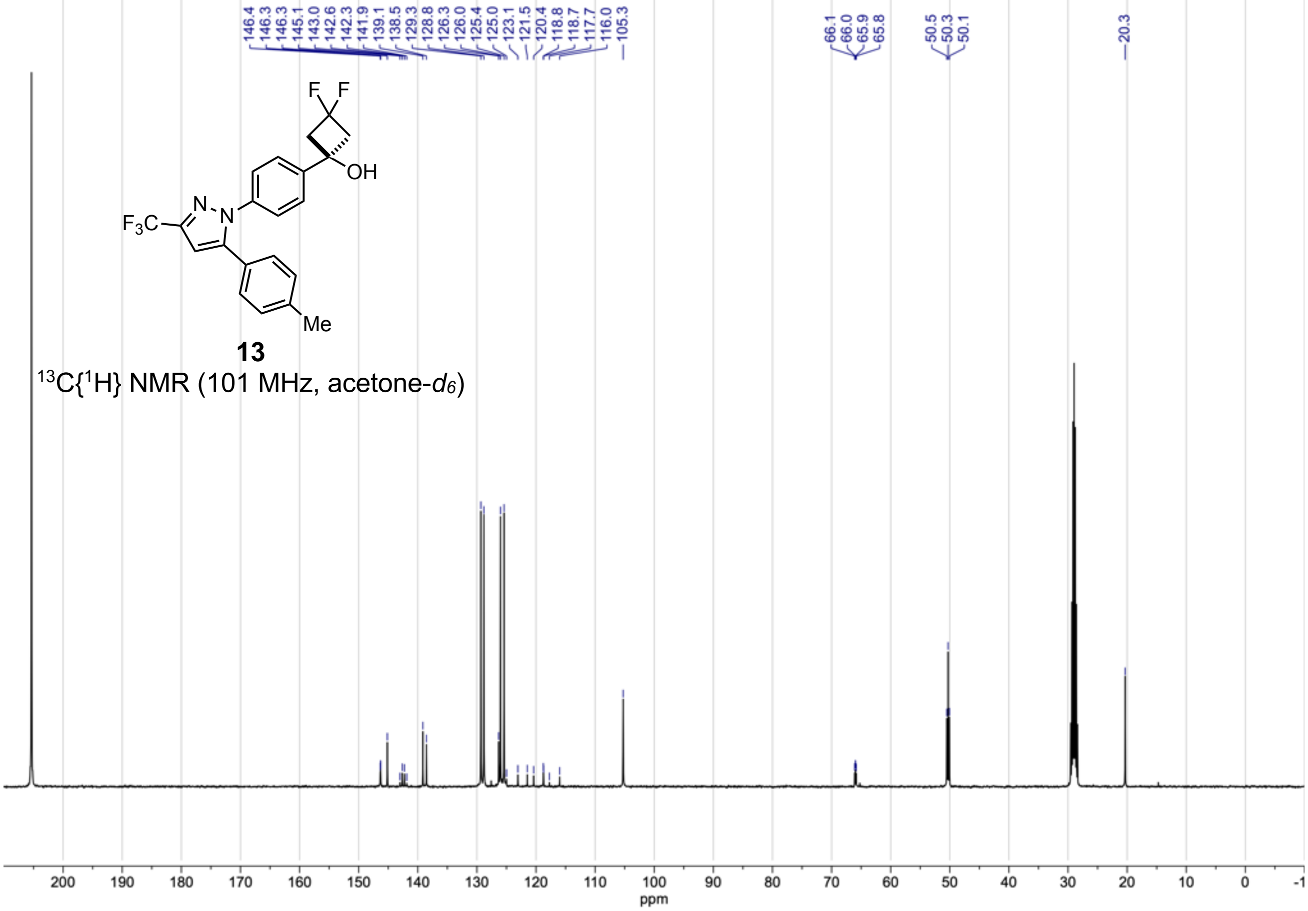

13

^{13}C{^{1}H} NMR (101 MHz, acetone-d_6)

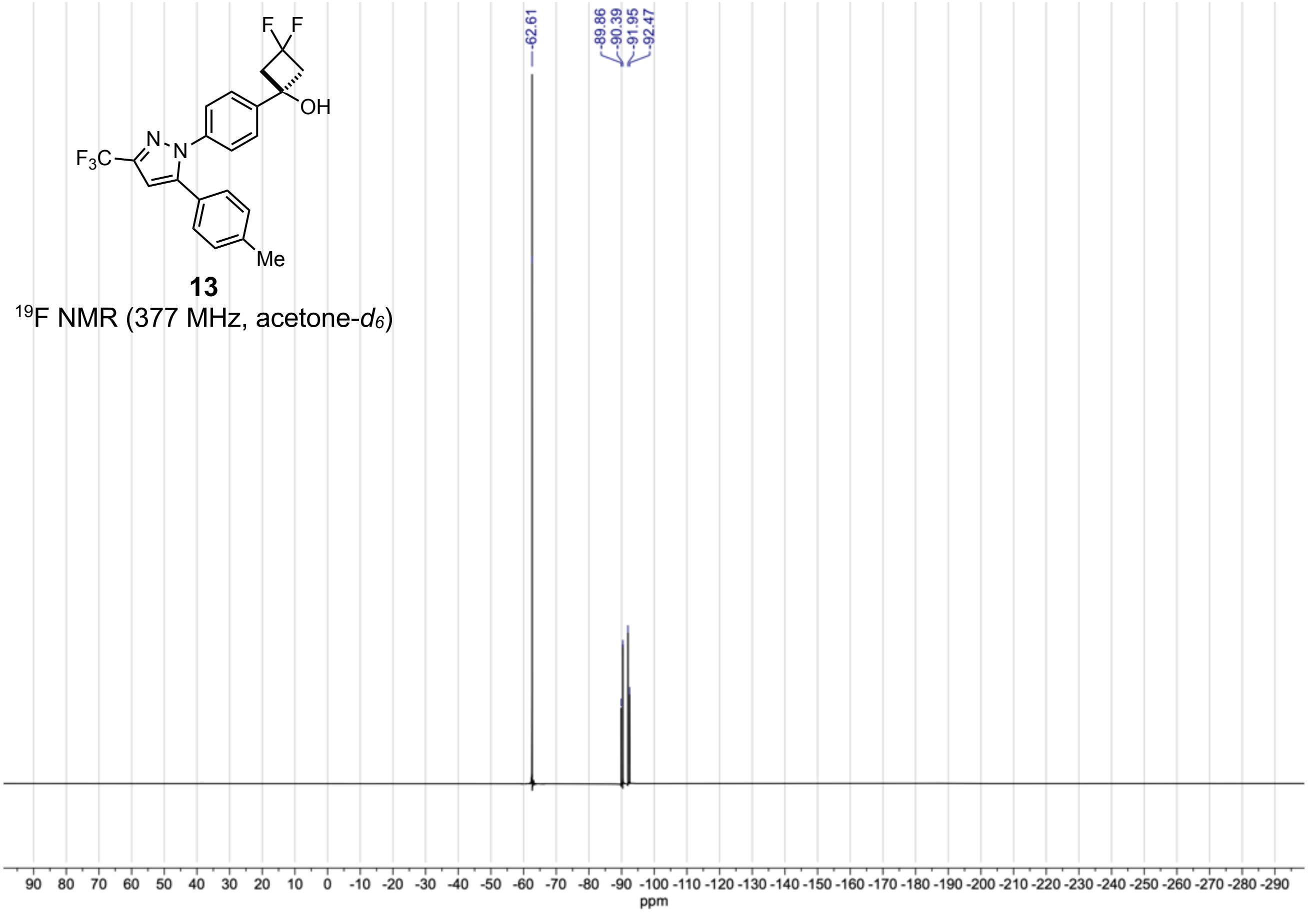

13

^{19}F NMR (377 MHz, acetone-d_6)

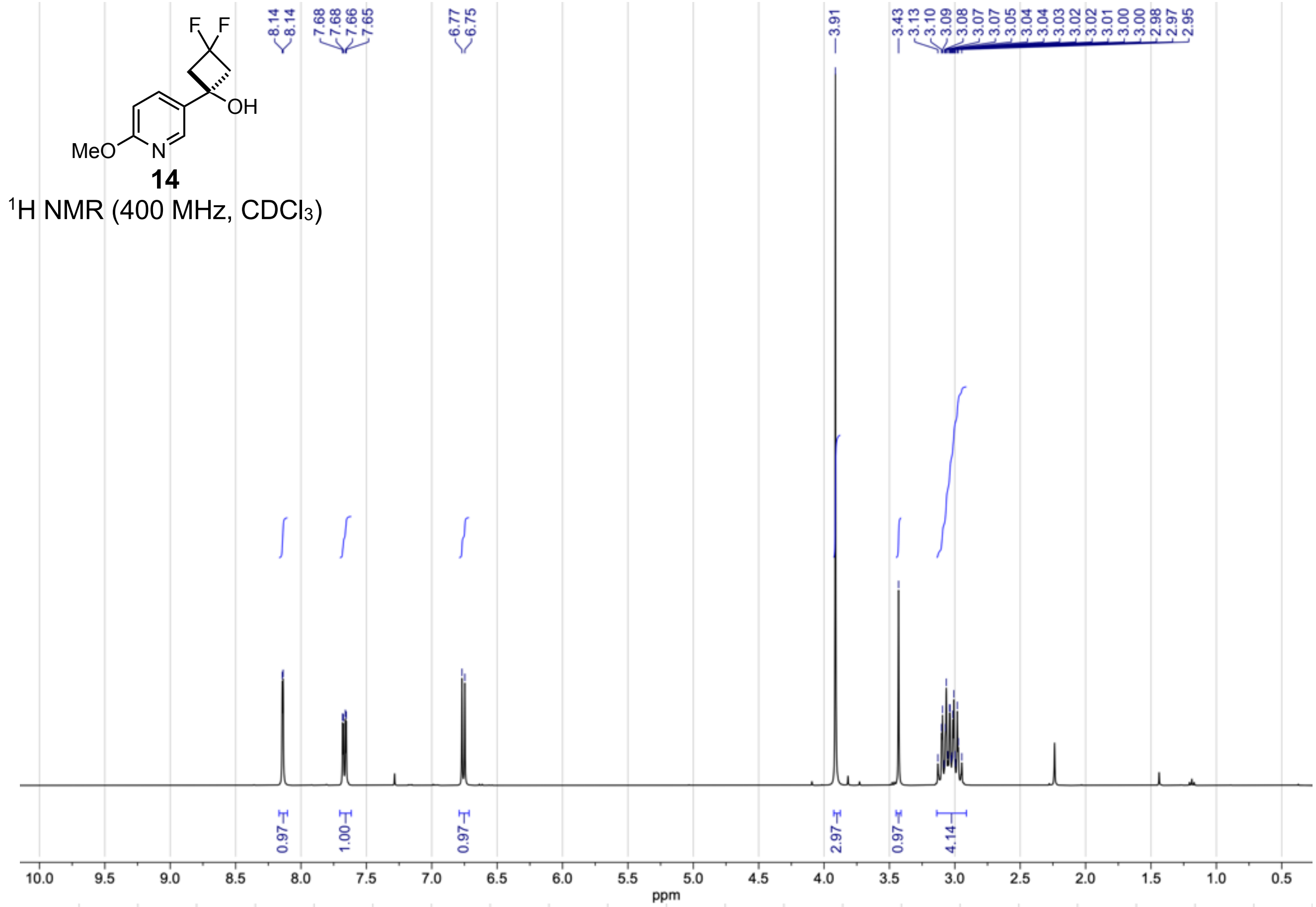
F F
OH
MeO
N
14
1H NMR (400 MHz, CDCl3)
8.14
8.14
7.68
7.68
7.66
7.65
6.77
6.75
3.91
3.43
3.13
3.10
3.09
3.08
3.07
3.07
3.05
3.04
3.04
3.03
3.02
3.02
3.01
3.00
3.00
2.98
2.97
2.95
0.97
1.00
0.97
2.97
0.97
4.14
ppm

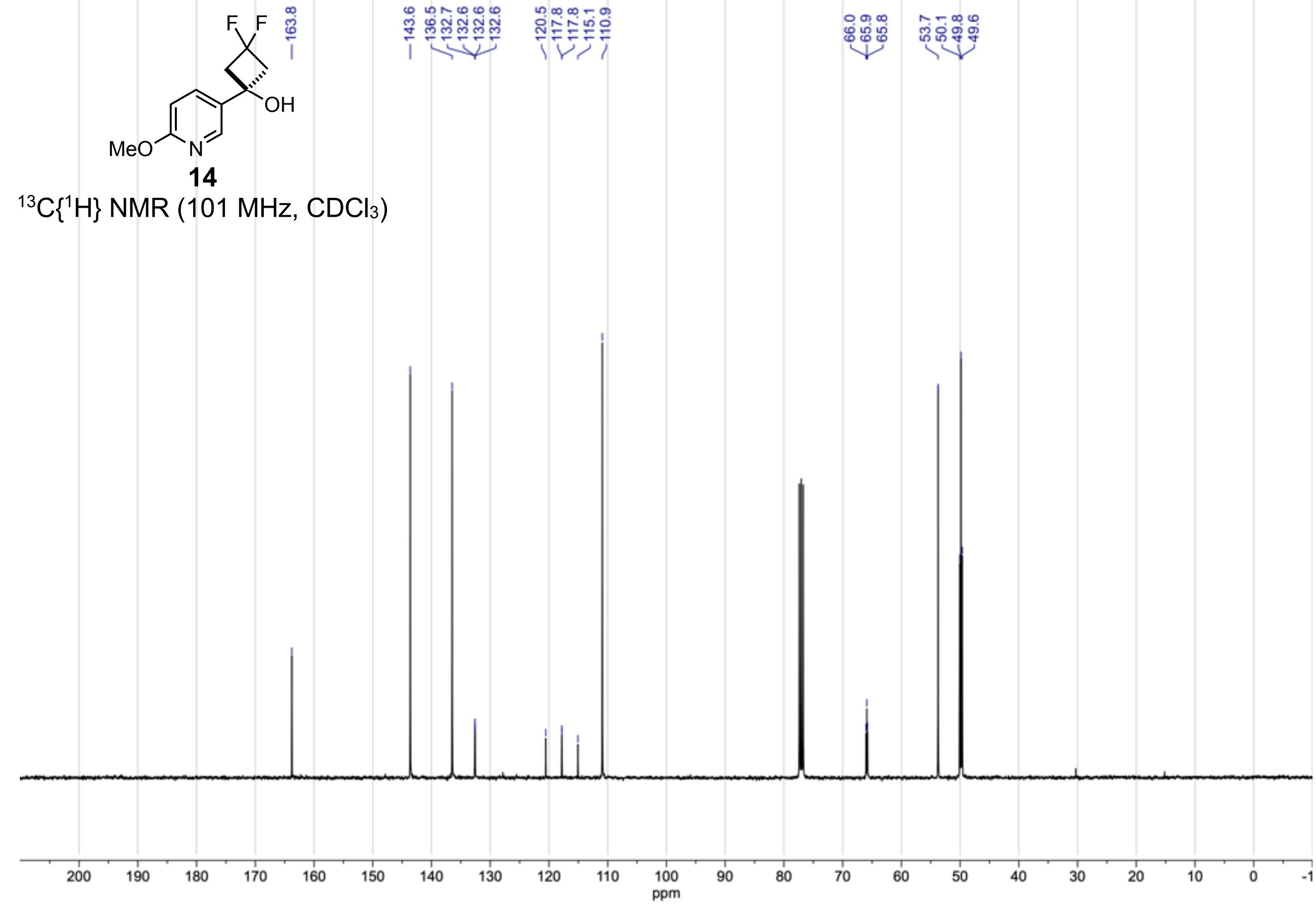
F F
OH
MeO
N
14
13C{1H} NMR (101 MHz, CDCl3)
163.8
143.6
136.5
132.7
132.6
132.6
132.6
120.5
117.8
117.8
115.1
110.9
66.0
65.9
65.8
53.7
50.1
49.8
49.6
ppm

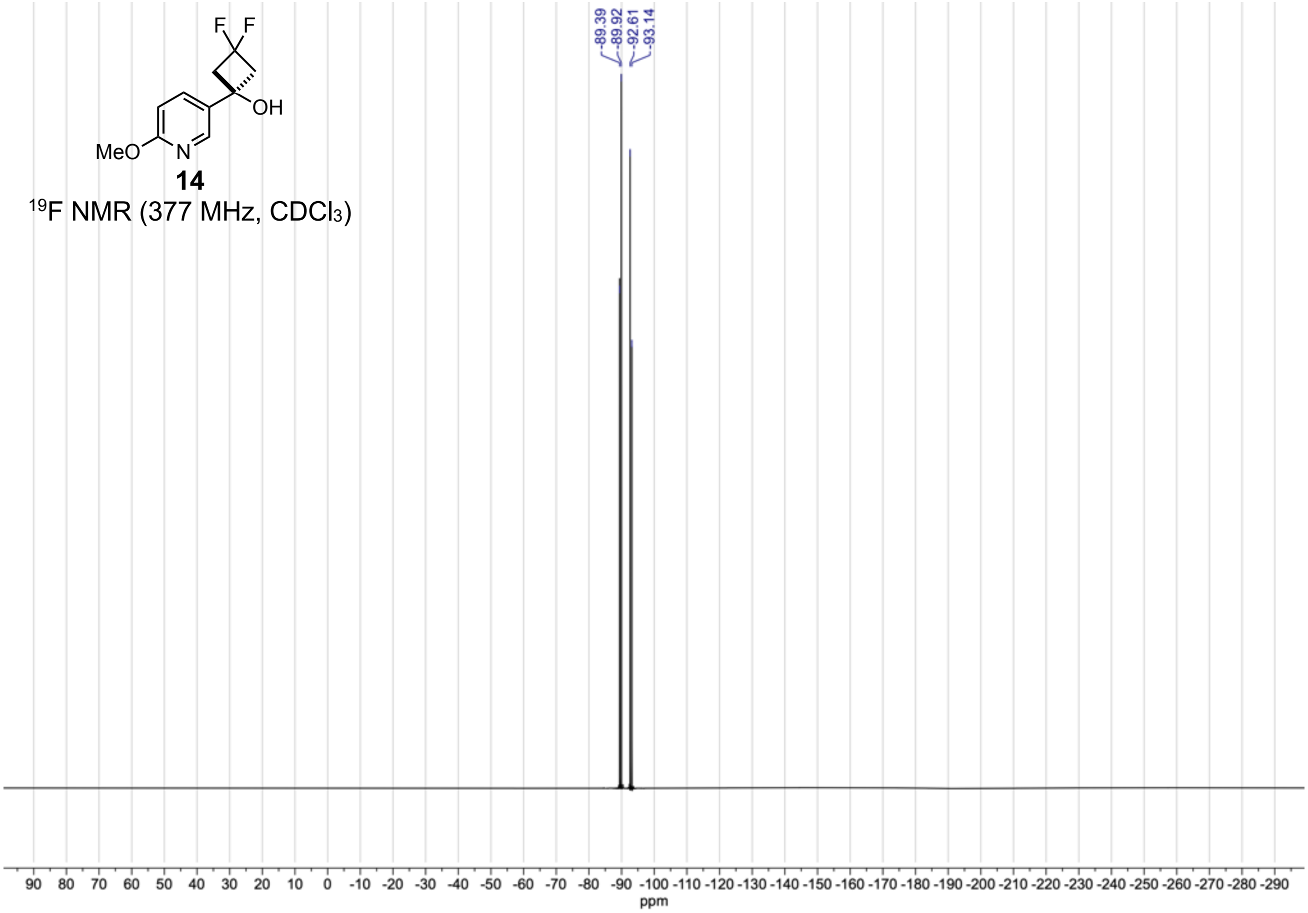
F F
OH
MeO
N
14
^{19}F NMR (377 MHz, $CDCl_3$)
-89.39
-89.92
-92.61
-93.14
90 80 70 60 50 40 30 20 10 0 -10 -20 -30 -40 -50 -60 -70 -80 -90 -100 -110 -120 -130 -140 -150 -160 -170 -180 -190 -200 -210 -220 -230 -240 -250 -260 -270 -280 -290
ppm

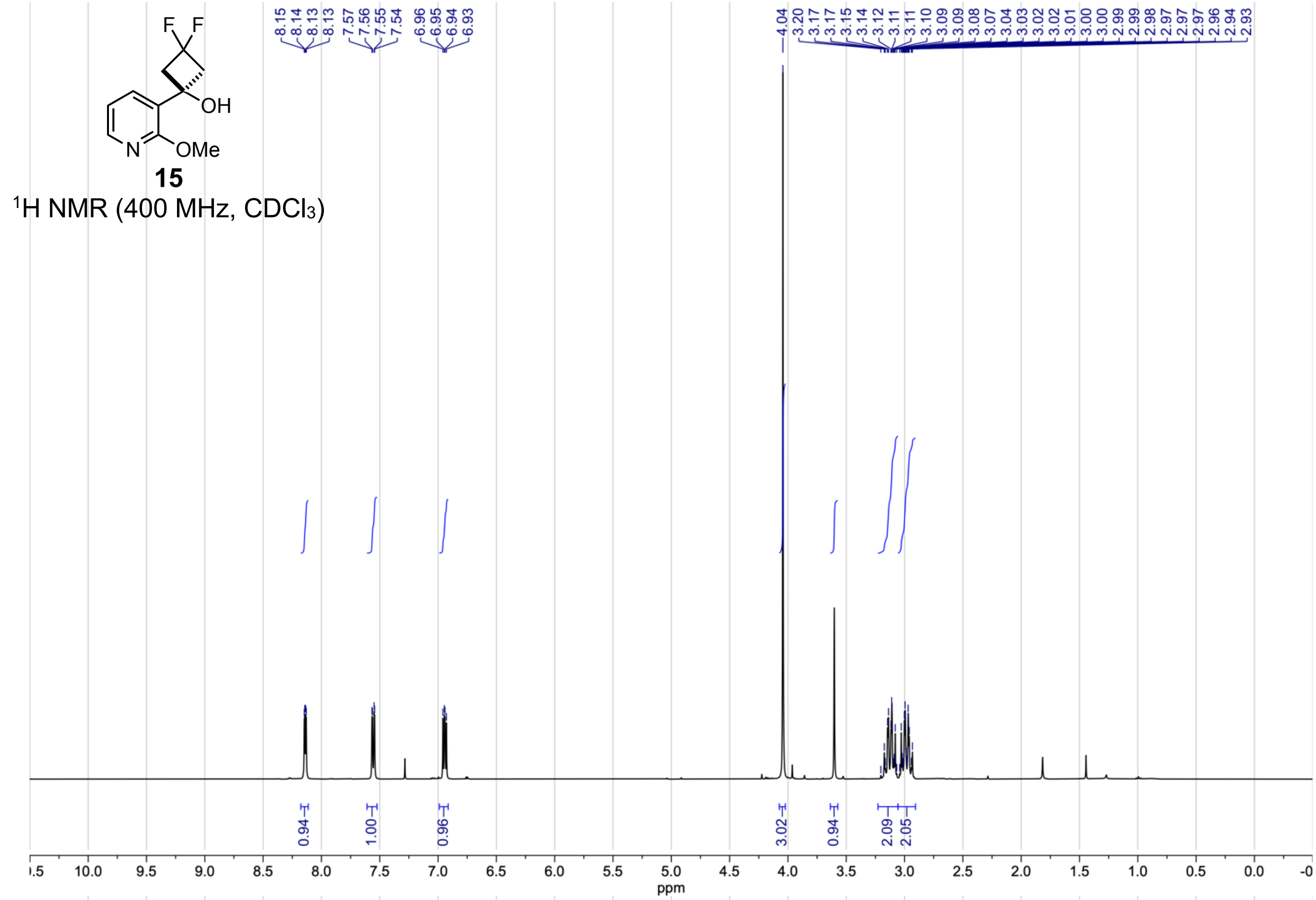
F
F
OH
N
OMe
15
1H NMR (400 MHz, CDCl3)
8.15
8.14
8.13
8.13
7.57
7.56
7.55
7.54
6.96
6.95
6.94
6.93
4.04
3.20
3.17
3.17
3.15
3.14
3.12
3.11
3.11
3.10
3.09
3.08
3.07
3.04
3.03
3.02
3.01
3.00
3.00
2.99
2.99
2.98
2.97
2.97
2.97
2.96
2.94
2.93
0.94
1.00
0.96
3.02
0.94
2.09
2.05
ppm

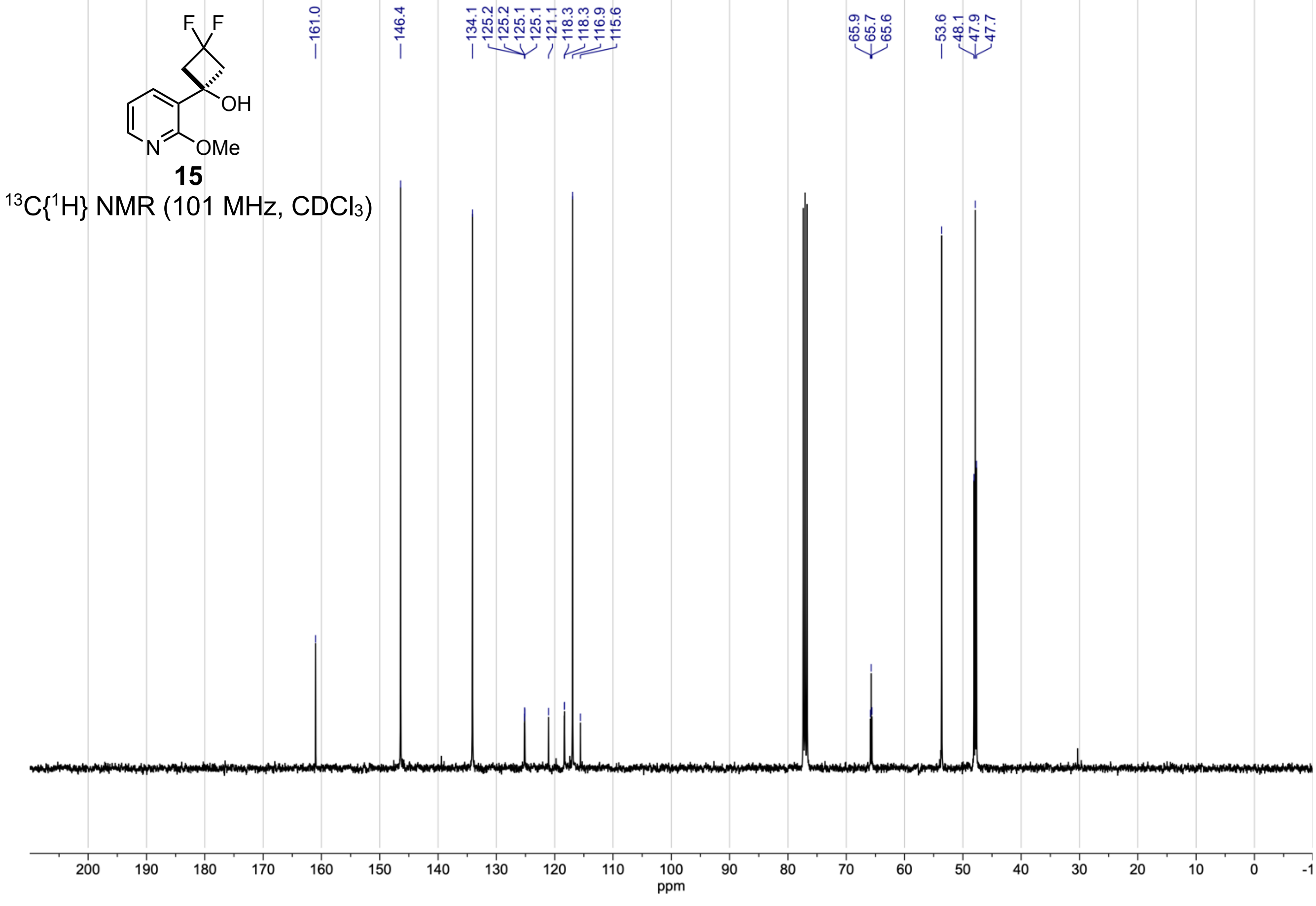
F
F
OH
N
OMe
15
13C{1H} NMR (101 MHz, CDCl3)
161.0
146.4
134.1
125.2
125.2
125.1
125.1
121.1
118.3
118.3
116.9
115.6
65.9
65.7
65.6
53.6
48.1
47.9
47.7
ppm

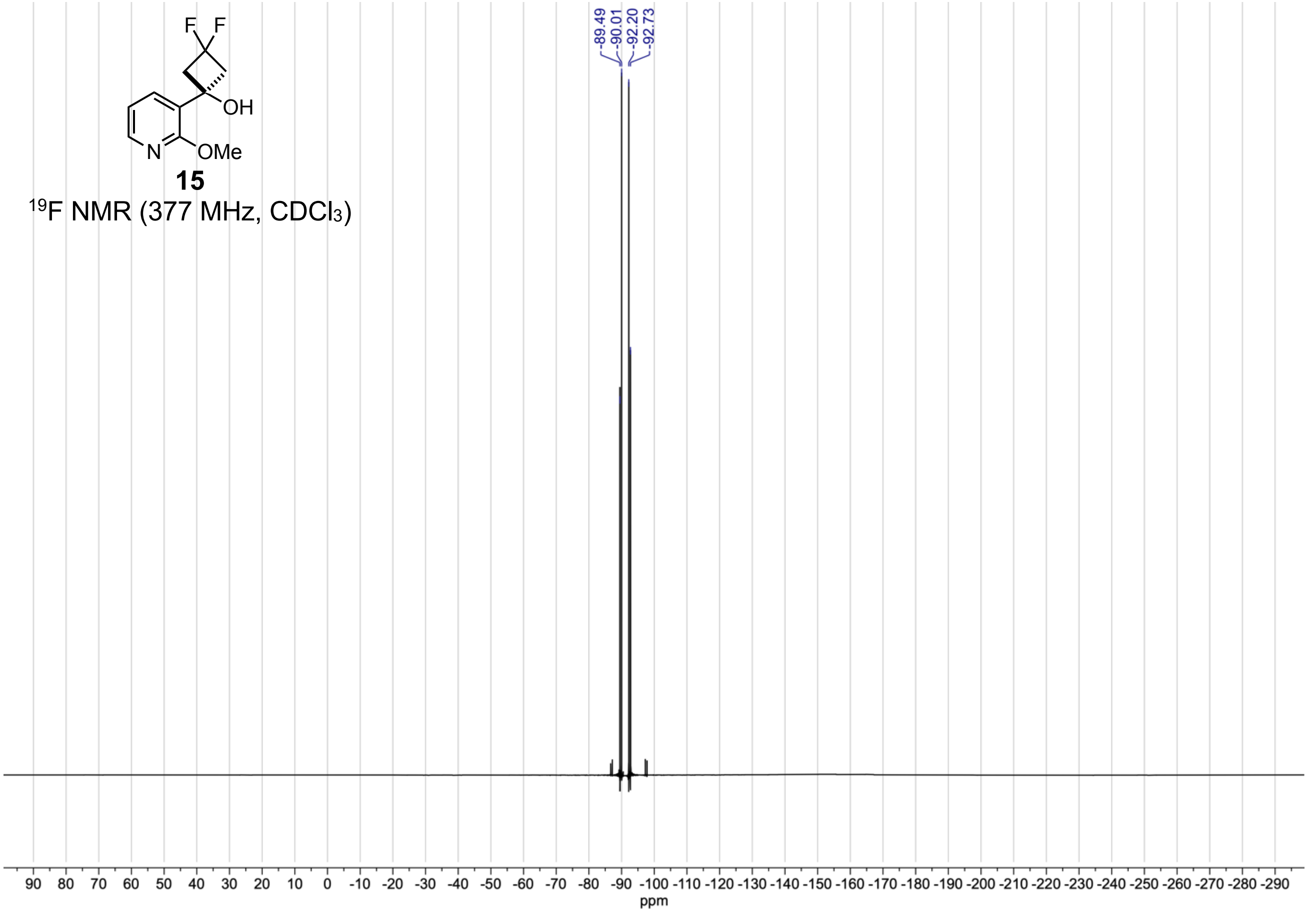

^{19}F NMR (377 MHz, $CDCl_3$)

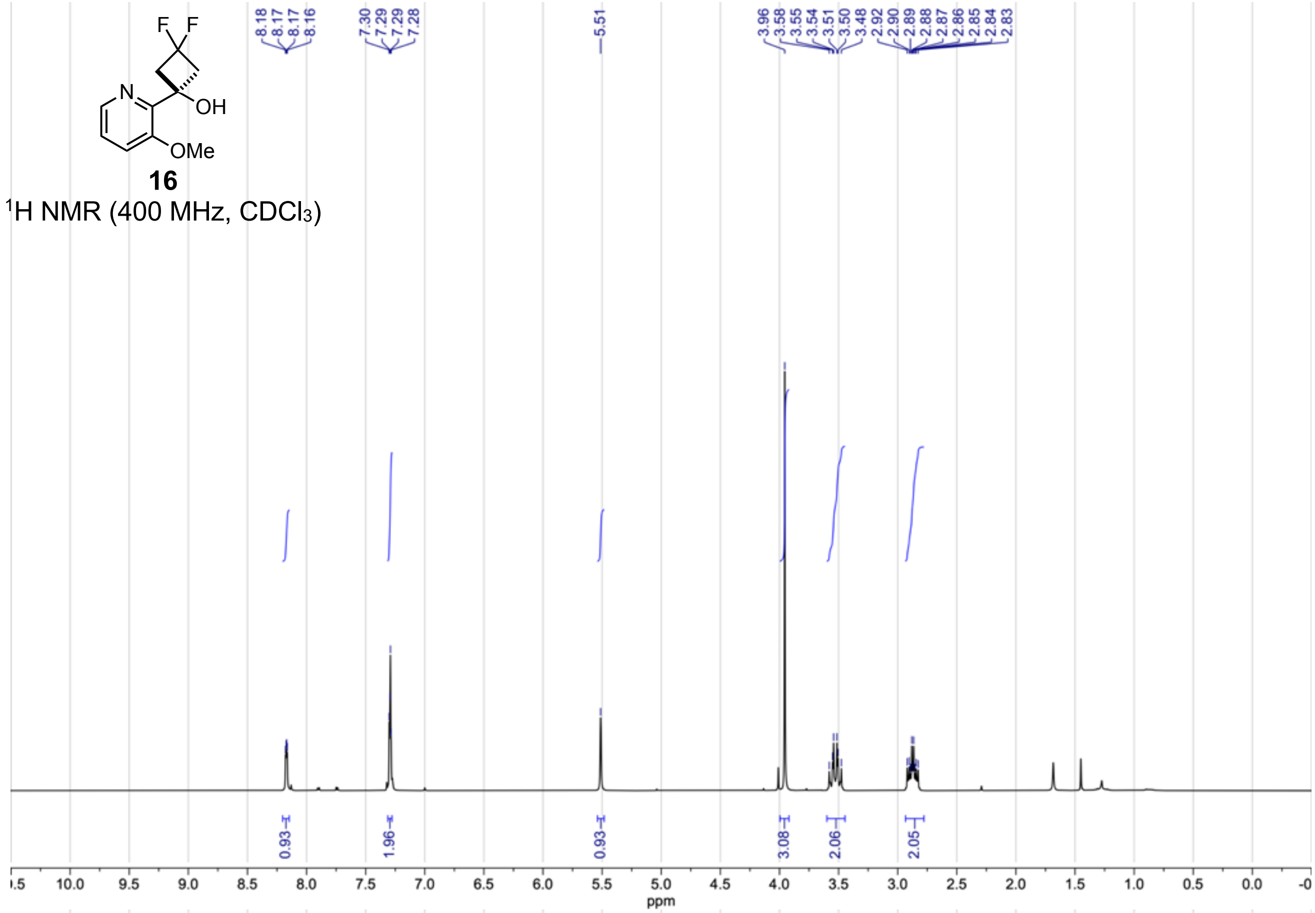
F F
N
OH
OMe
16
1H NMR (400 MHz, CDCl3)
8.18 8.17 8.17 8.16
7.30 7.29 7.29 7.28
5.51
3.96 3.58 3.55 3.54 3.51 3.50 3.48 2.92 2.90 2.89 2.88 2.87 2.86 2.85 2.84 2.83
0.93
1.96
0.93
3.08
2.06
2.05
ppm

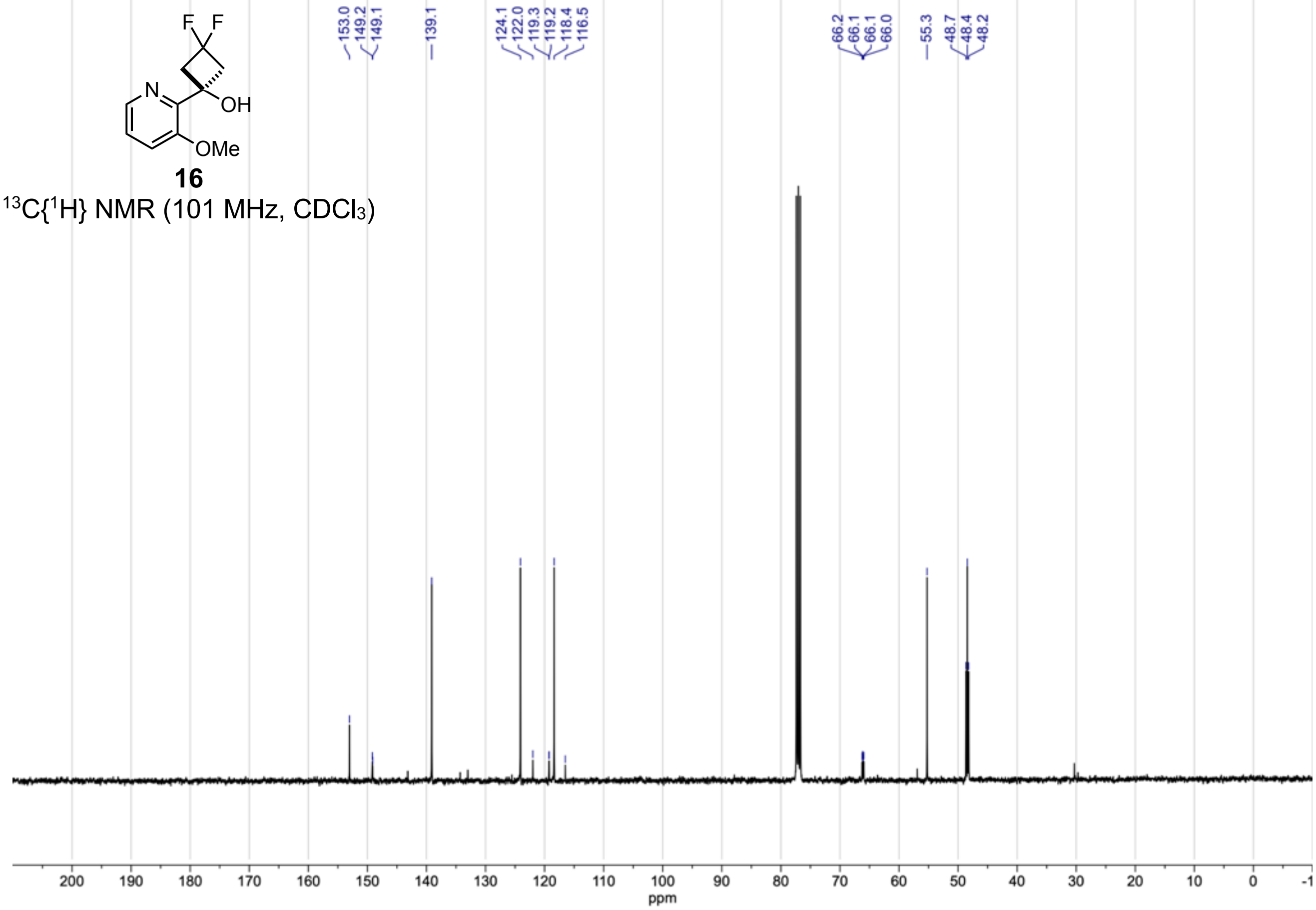
F F
N
OH
OMe
16
13C{1H} NMR (101 MHz, CDCl3)
153.0 149.2 149.1
139.1
124.1 122.0 119.3 119.2 118.4 116.5
66.2 66.1 66.1 66.0
55.3
48.7 48.4 48.2
ppm

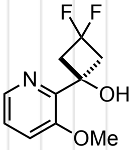

16

^{19}F NMR (377 MHz, $CDCl_3$)

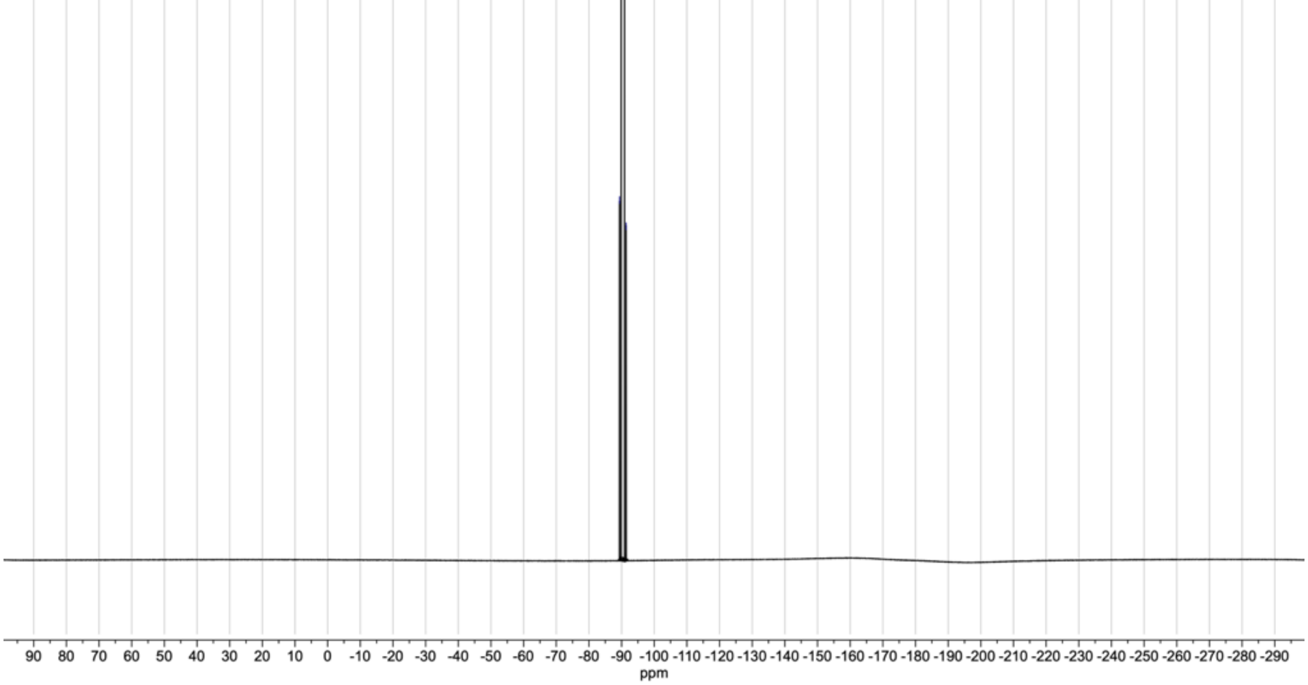

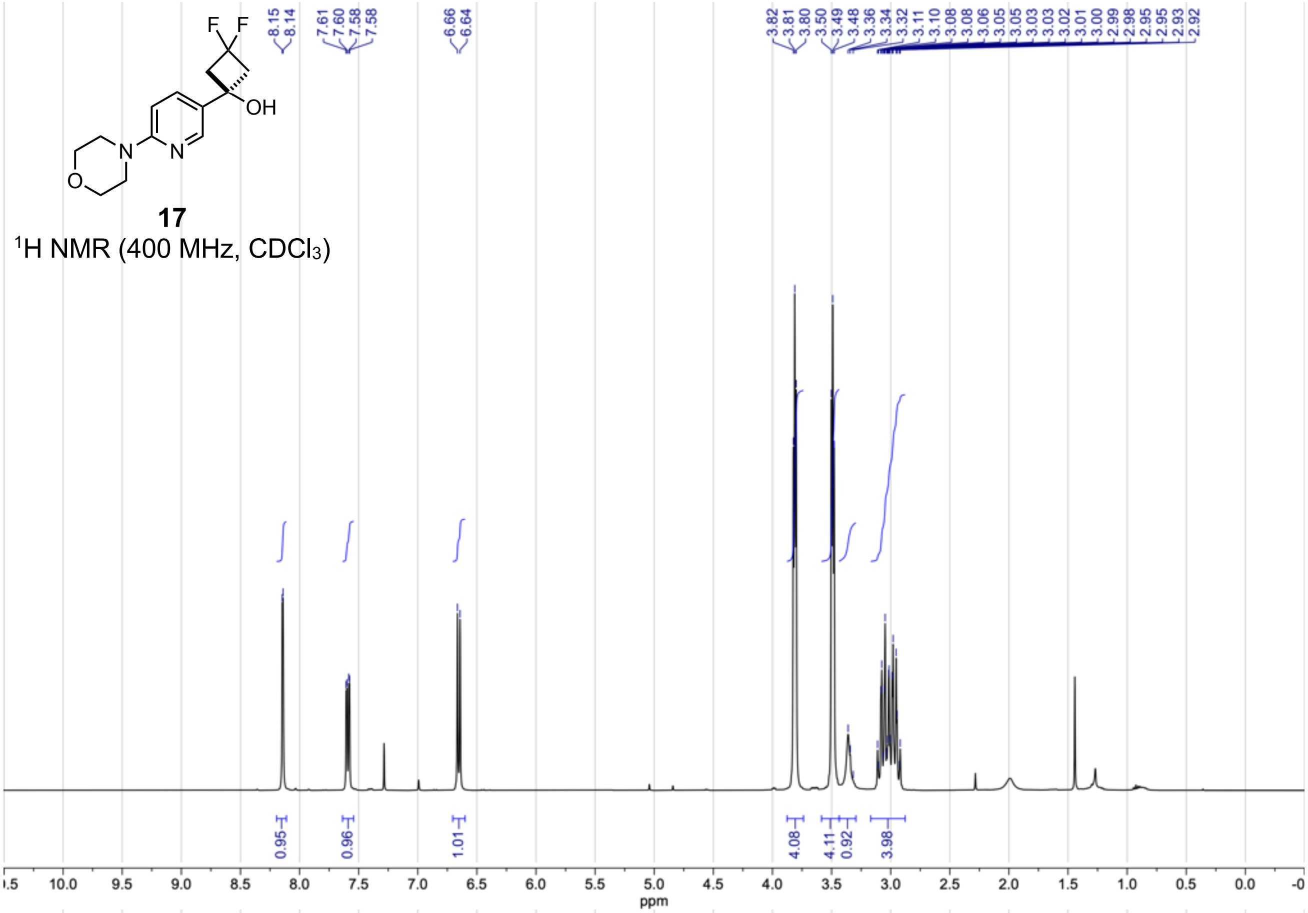
17
^{1}H NMR (400 MHz, CDCl$_3$)
ppm

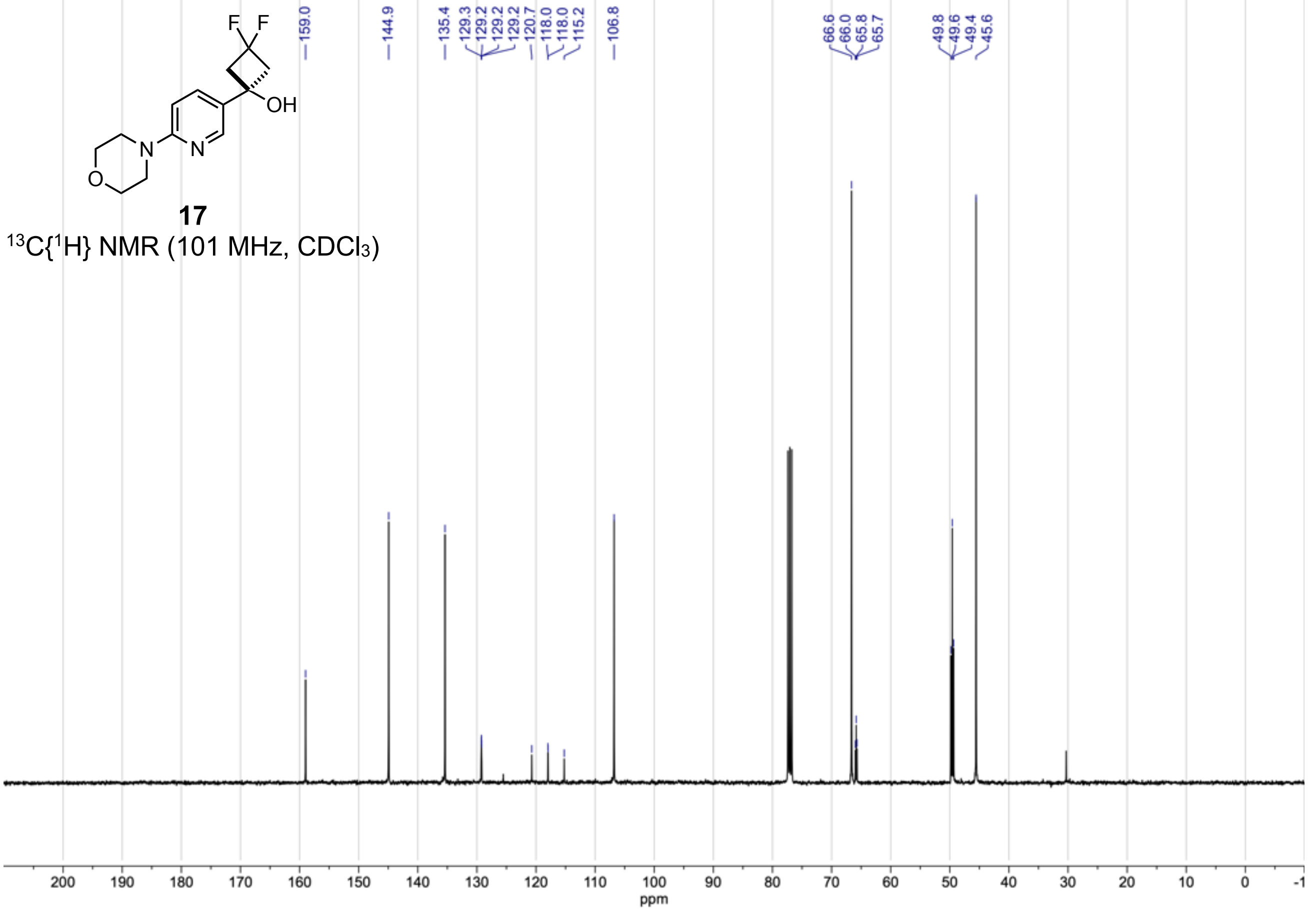
17
^{13}C{^{1}H} NMR (101 MHz, CDCl$_3$)
ppm

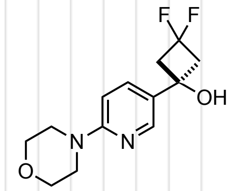

17

^{19}F NMR (377 MHz, $CDCl_3$)

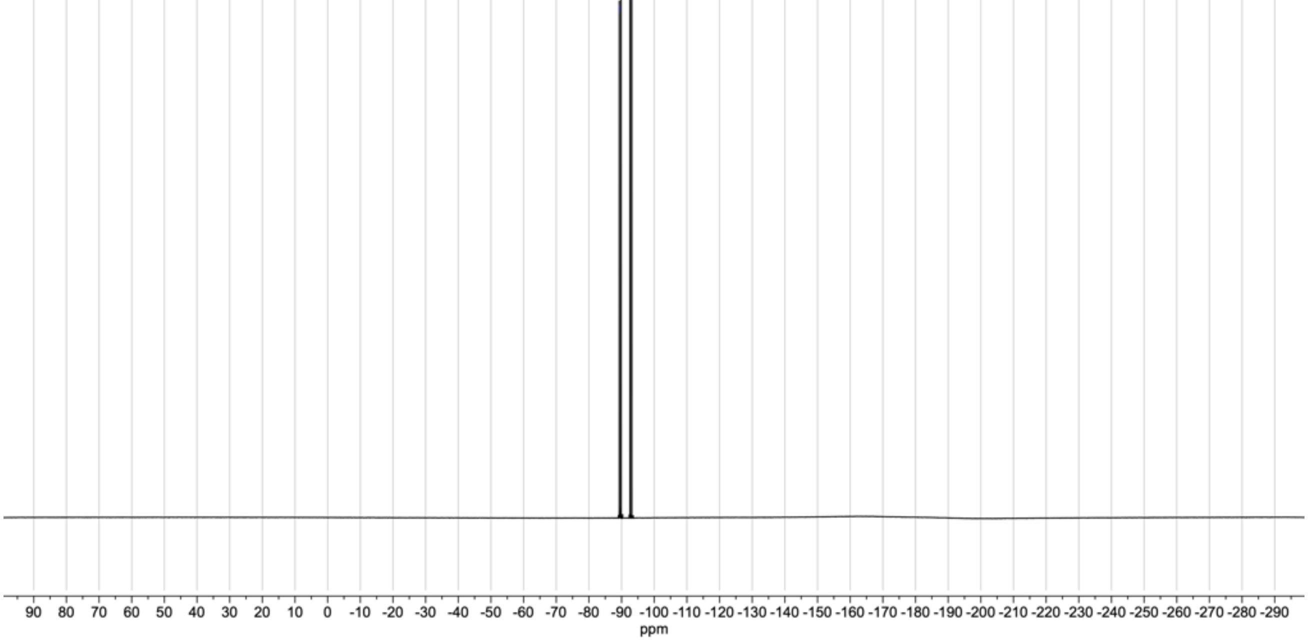

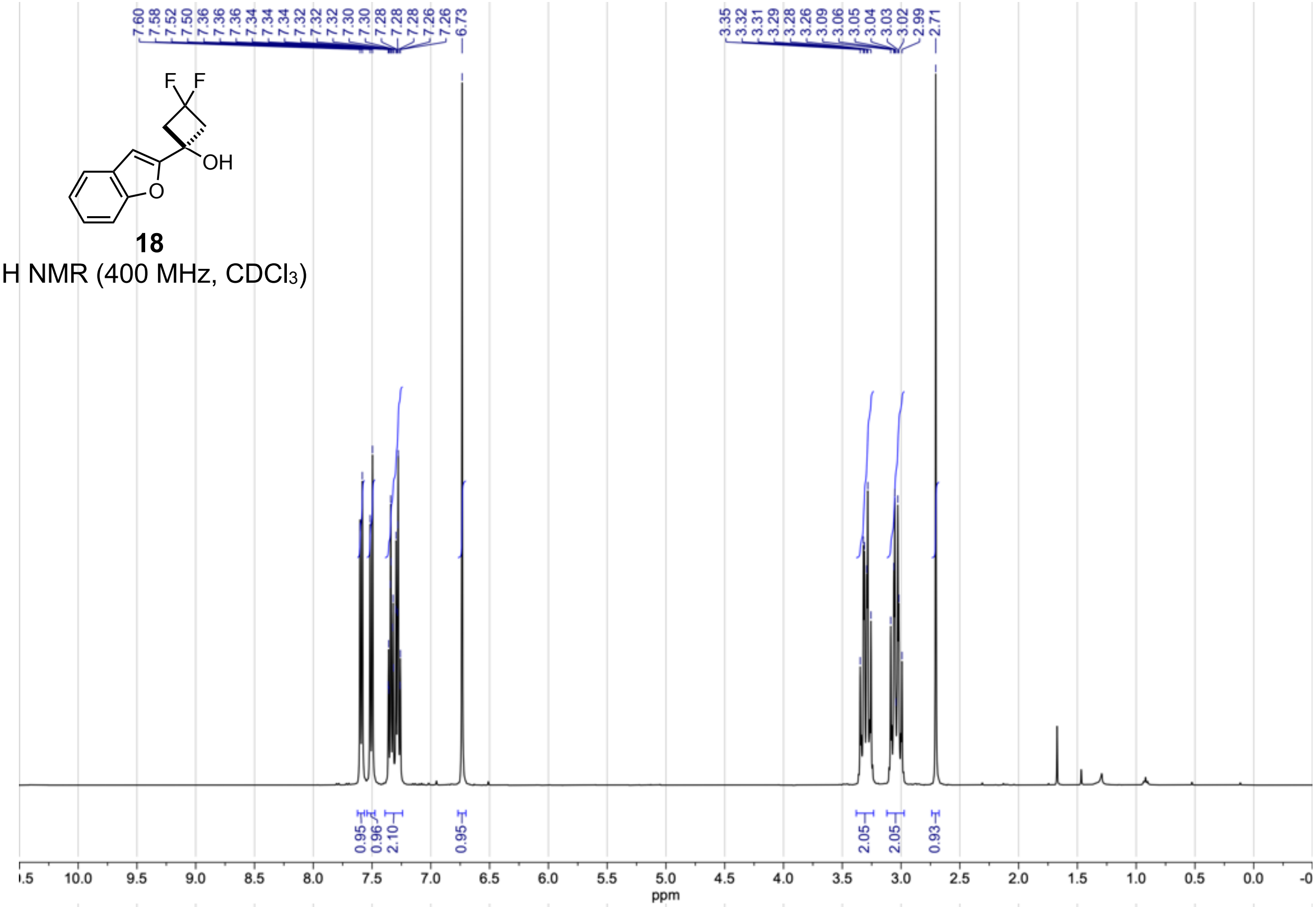

18

^{1}H NMR (400 MHz, $CDCl_3$)

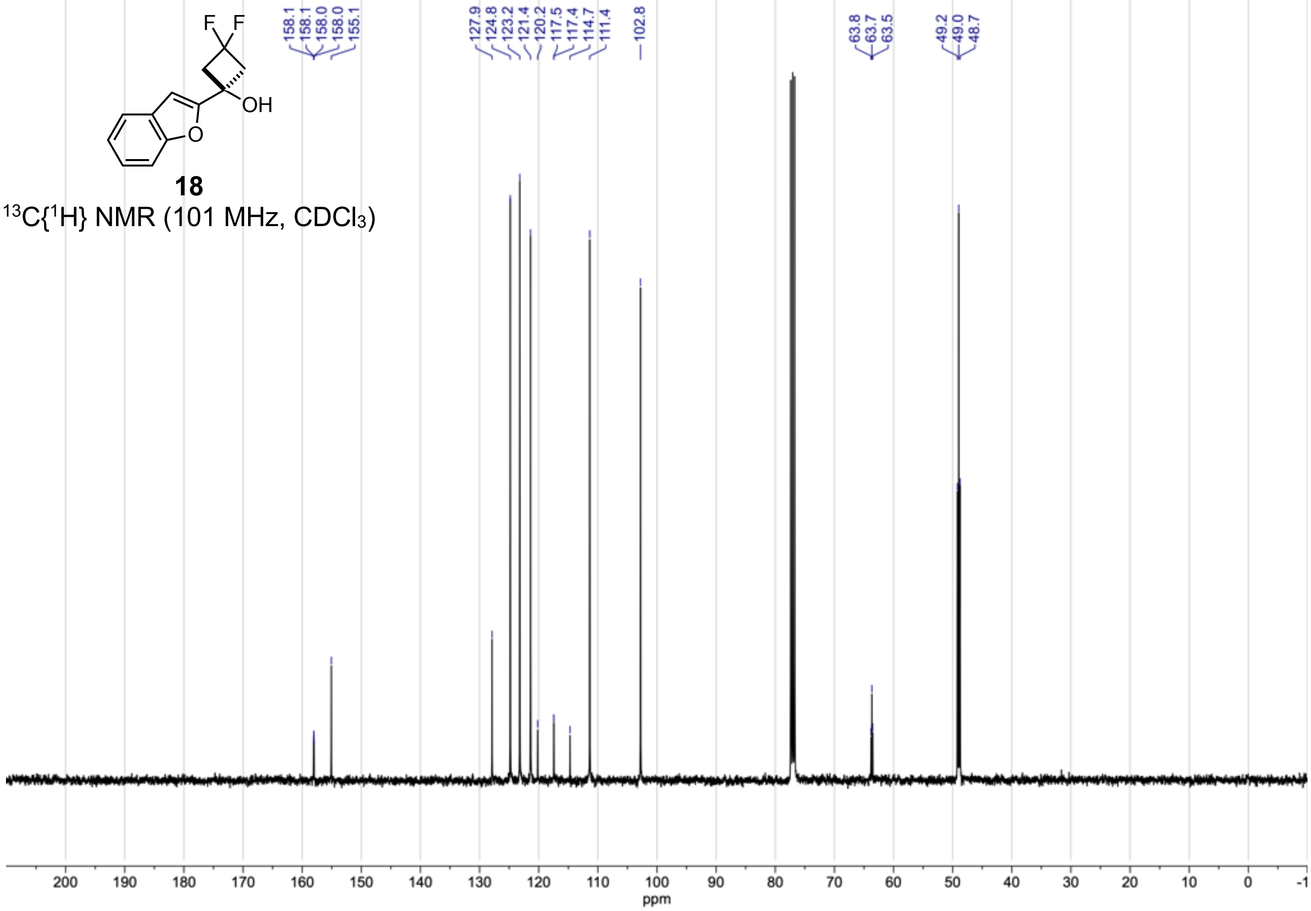

18

$^{13}C\{^1H\}$ NMR (101 MHz, $CDCl_3$)

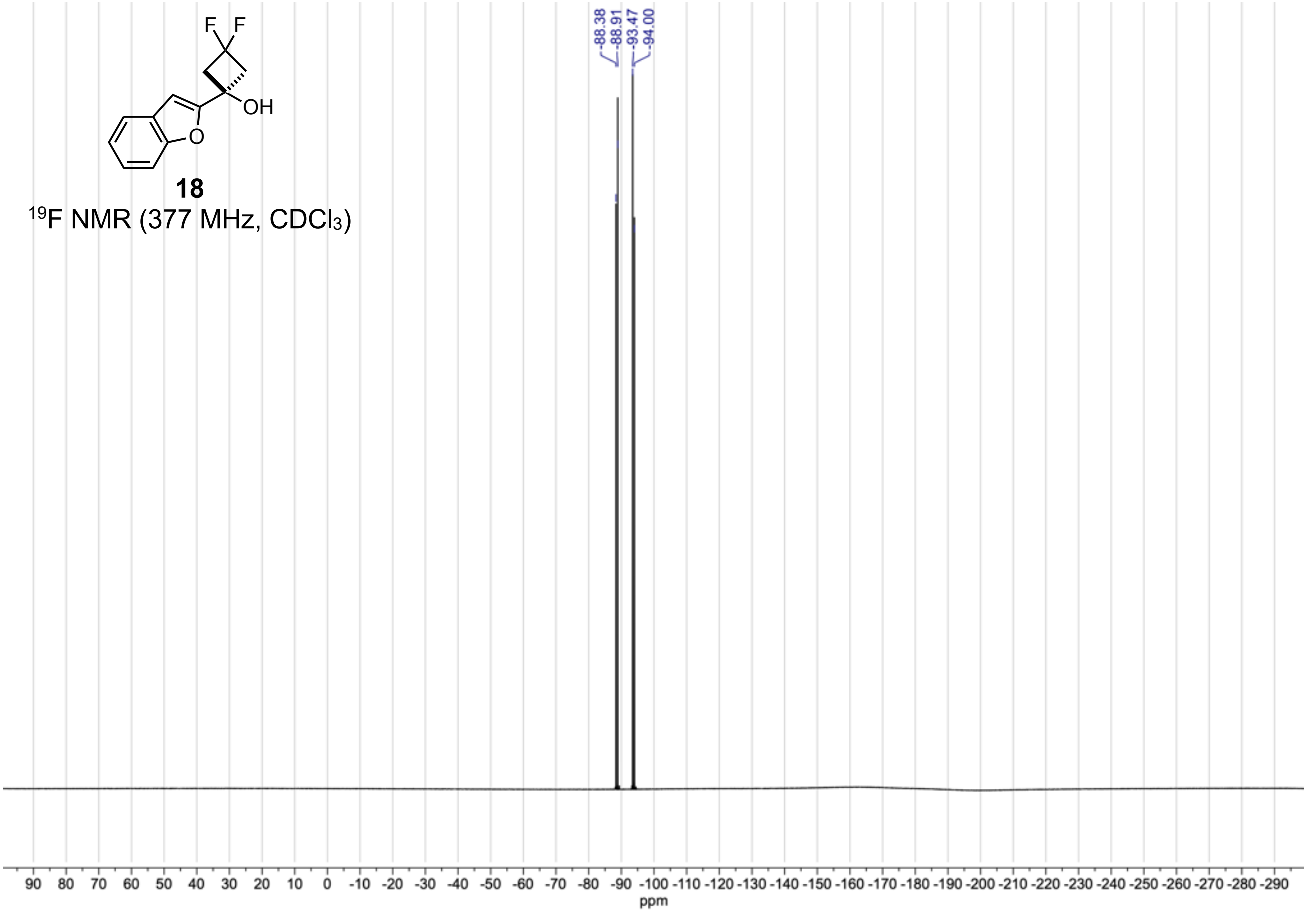

18

^{19}F NMR (377 MHz, $CDCl_3$)

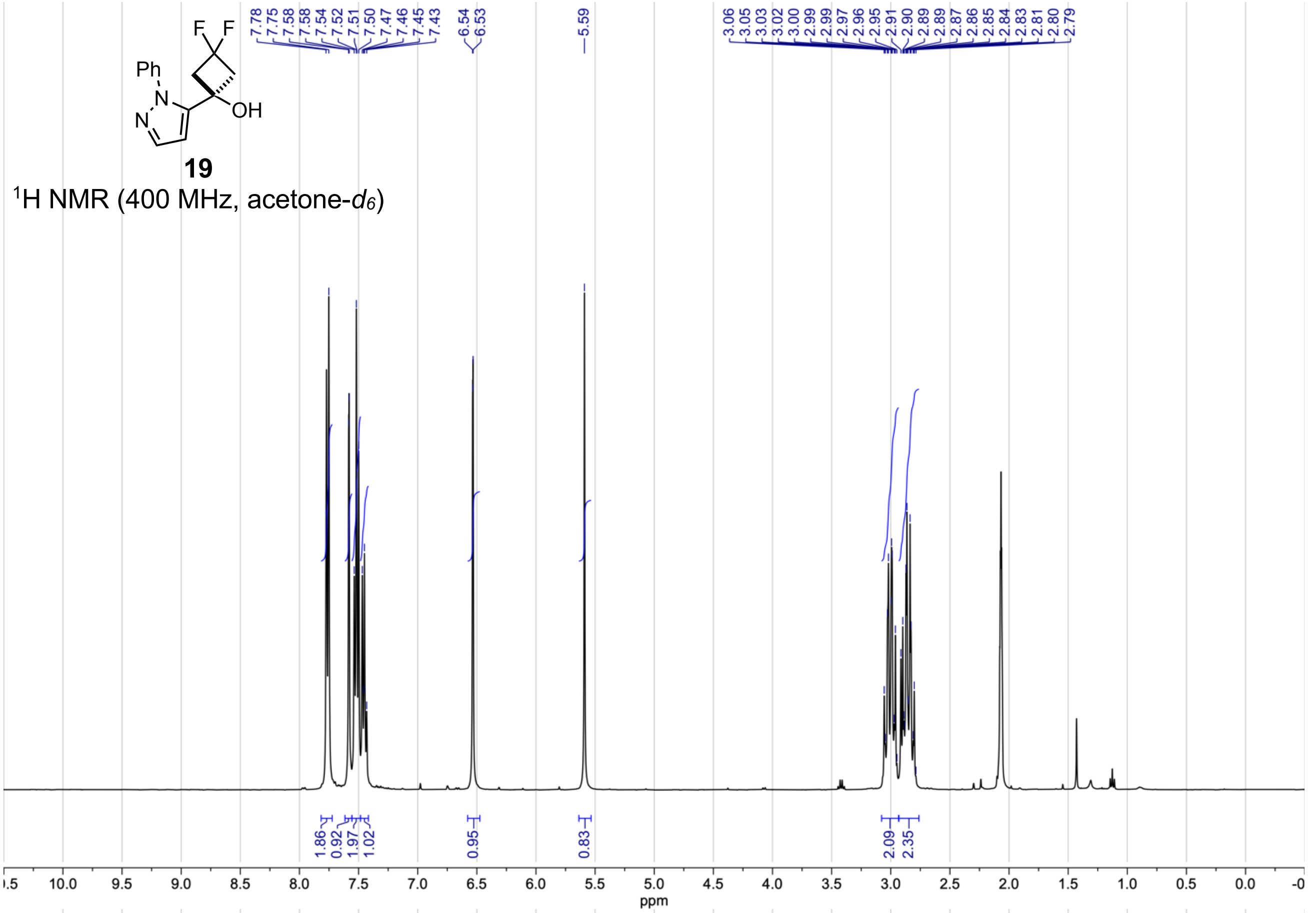
19
^{1}H NMR (400 MHz, acetone-d_6)

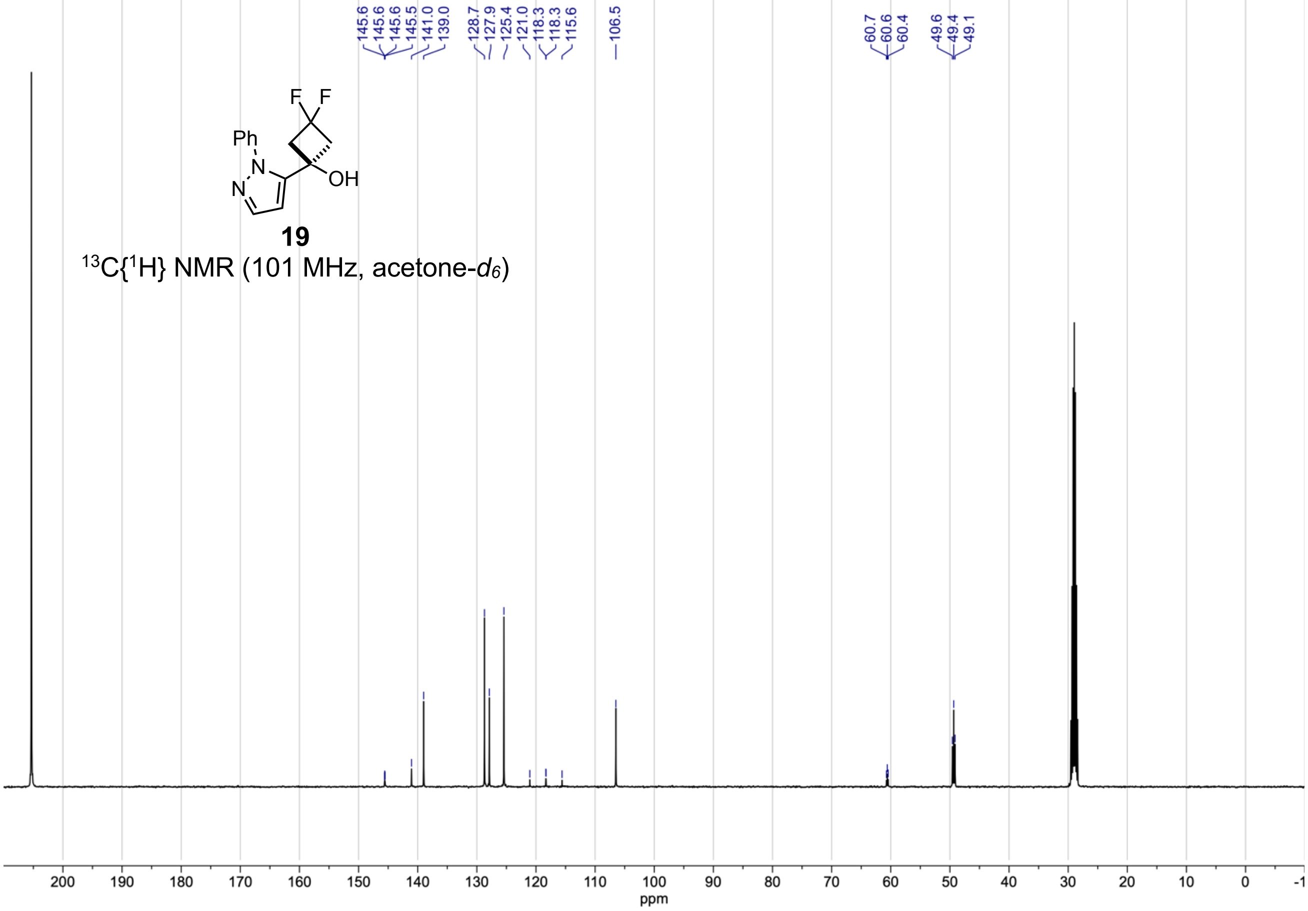
19
^{13}C{^{1}H} NMR (101 MHz, acetone-d_6)

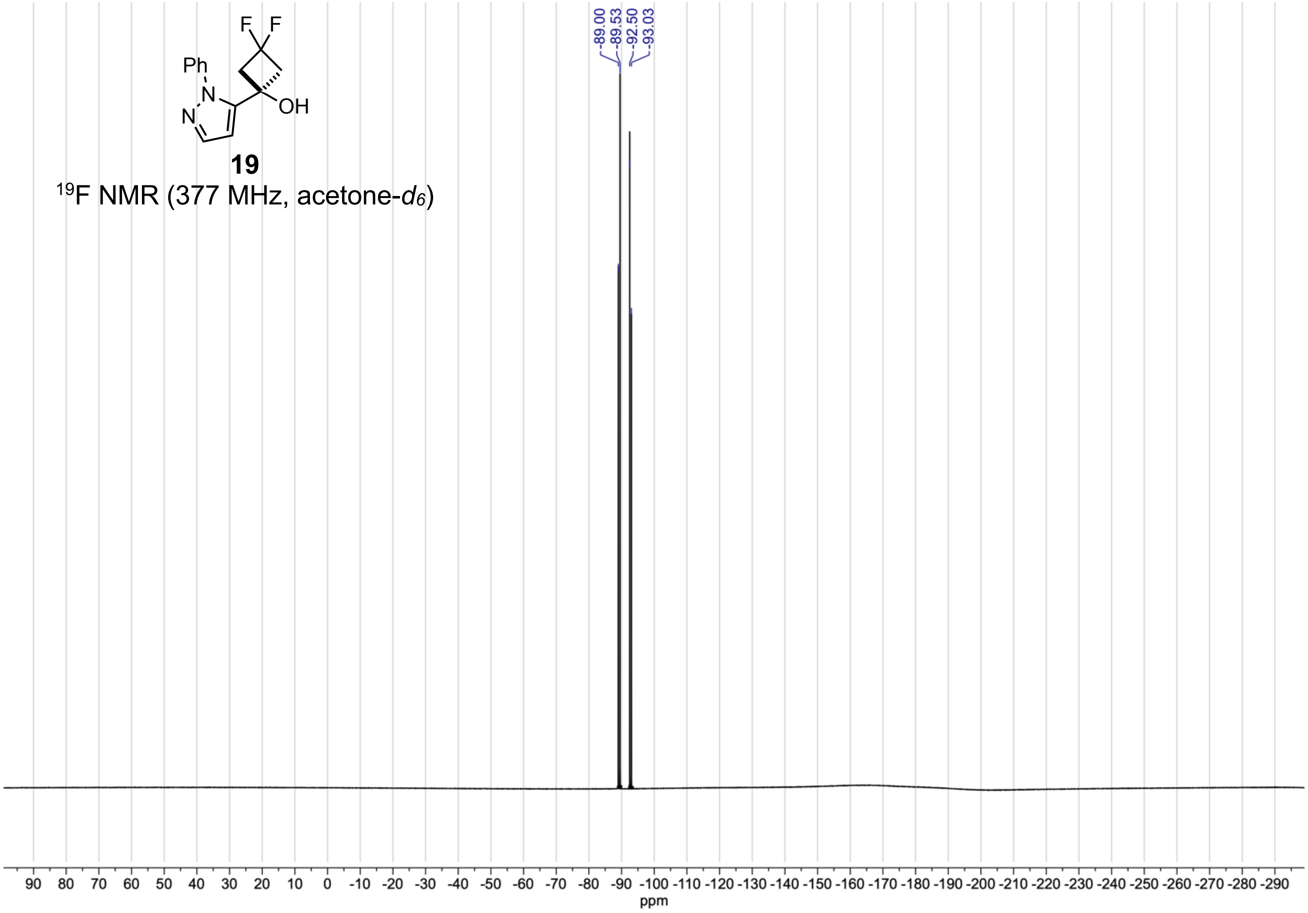

19

^{19}F NMR (377 MHz, acetone-d_6)

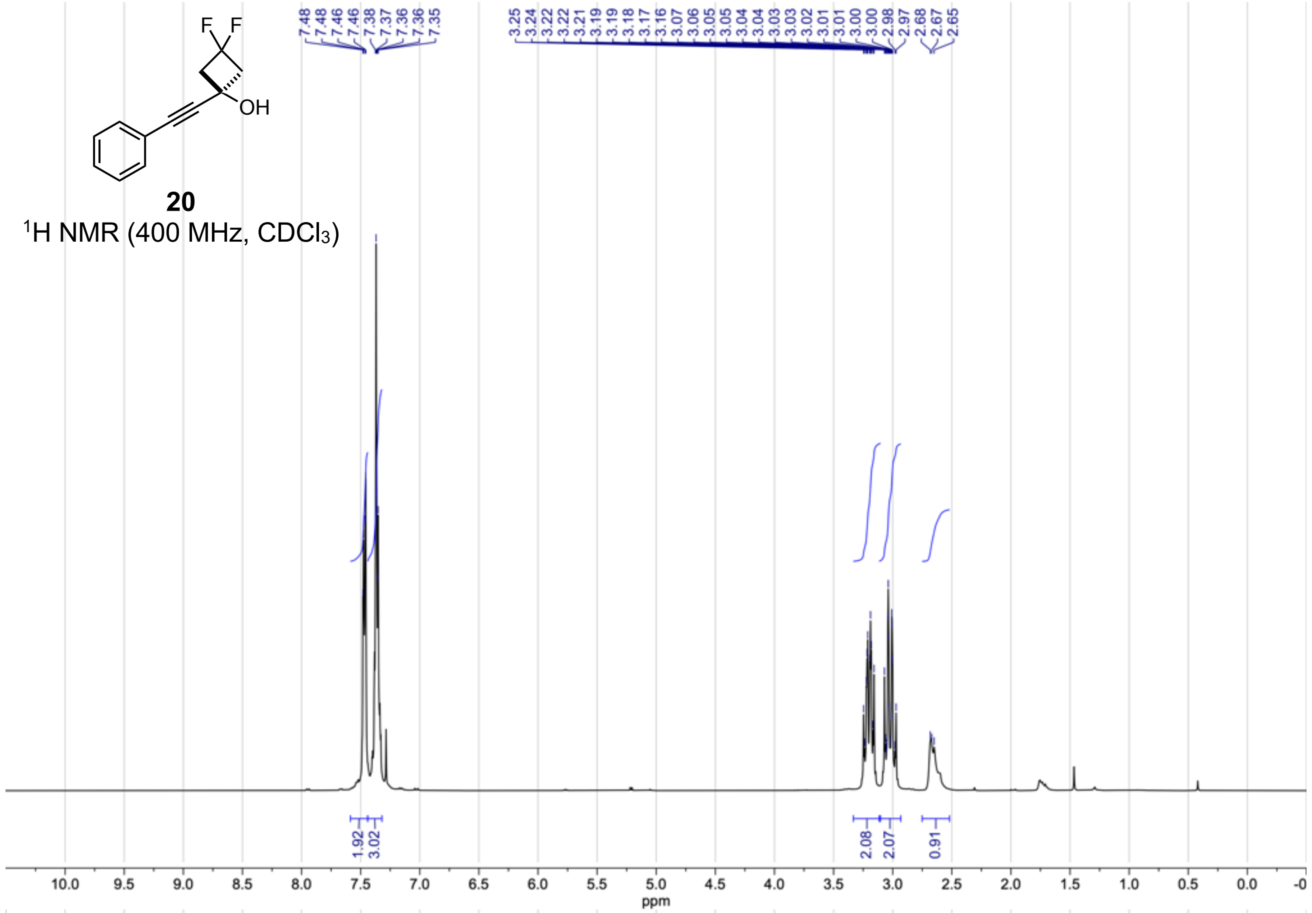
F F
OH
20
1H NMR (400 MHz, CDCl3)
7.48 7.48 7.46 7.46 7.38 7.37 7.36 7.36 7.35
3.25 3.24 3.22 3.22 3.21 3.19 3.19 3.18 3.17 3.16 3.07 3.06 3.05 3.05 3.04 3.04 3.03 3.03 3.02 3.01 3.01 3.00 3.00 2.98 2.97 2.68 2.67 2.65
1.92 3.02 2.08 2.07 0.91
ppm

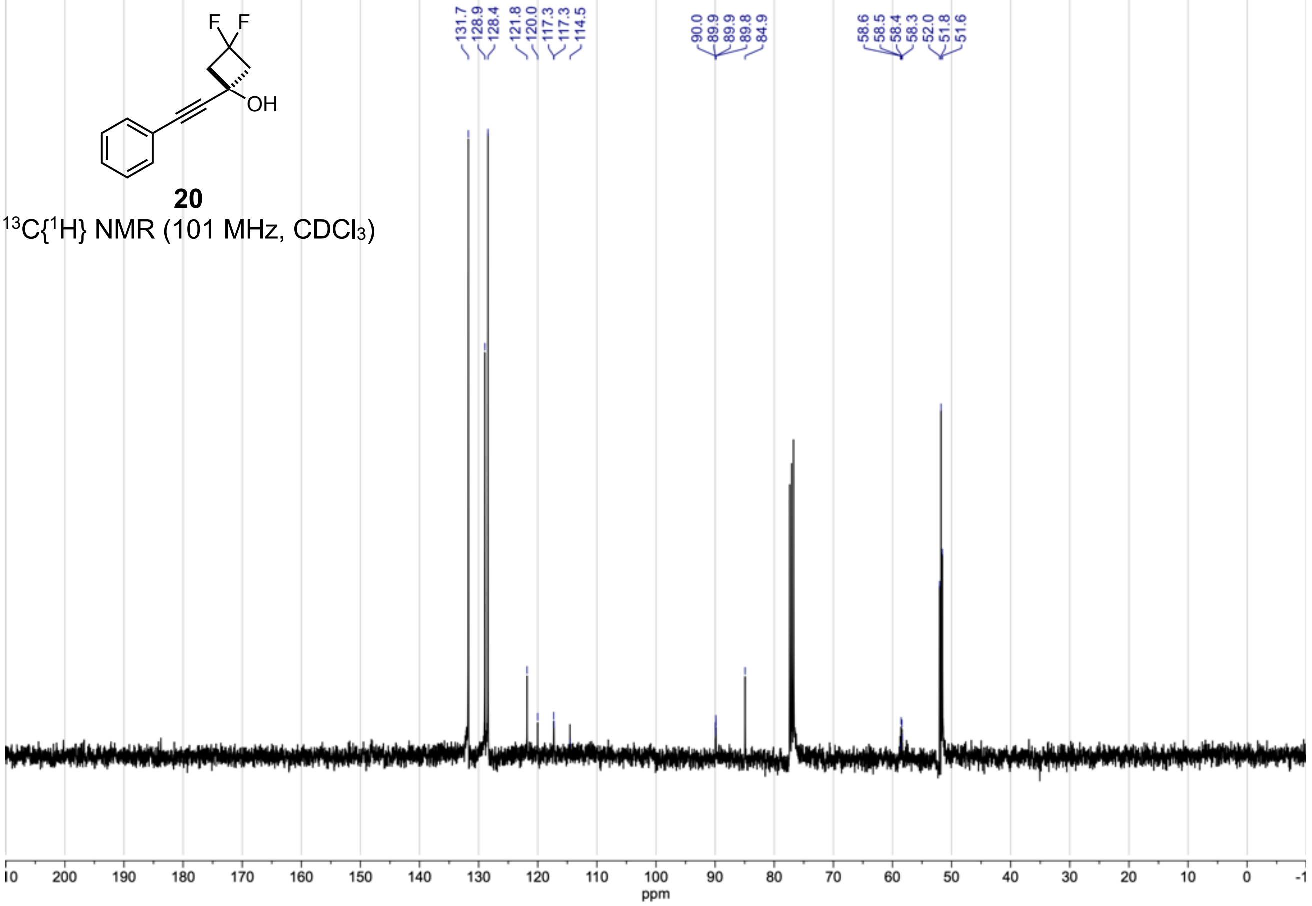
F F
OH
20
13C{1H} NMR (101 MHz, CDCl3)
131.7 128.9 128.4 121.8 120.0 117.3 117.3 114.5
90.0 89.9 89.9 89.8 84.9
58.6 58.5 58.4 58.3 52.0 51.8 51.6
ppm

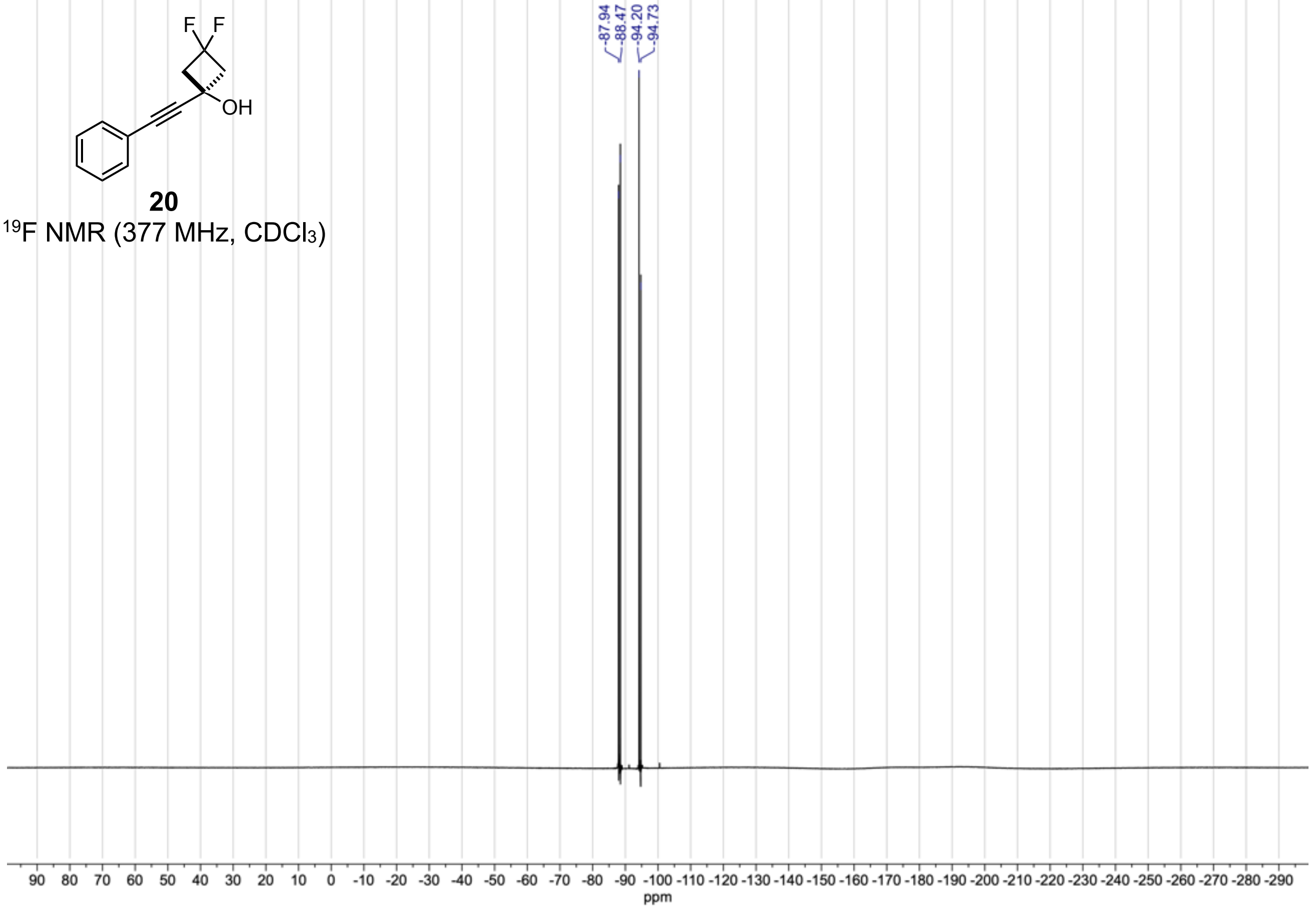

20

^{19}F NMR (377 MHz, $CDCl_3$)

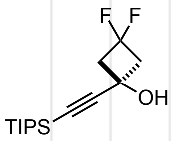

21

^{1}H NMR (400 MHz, $CDCl_3$)

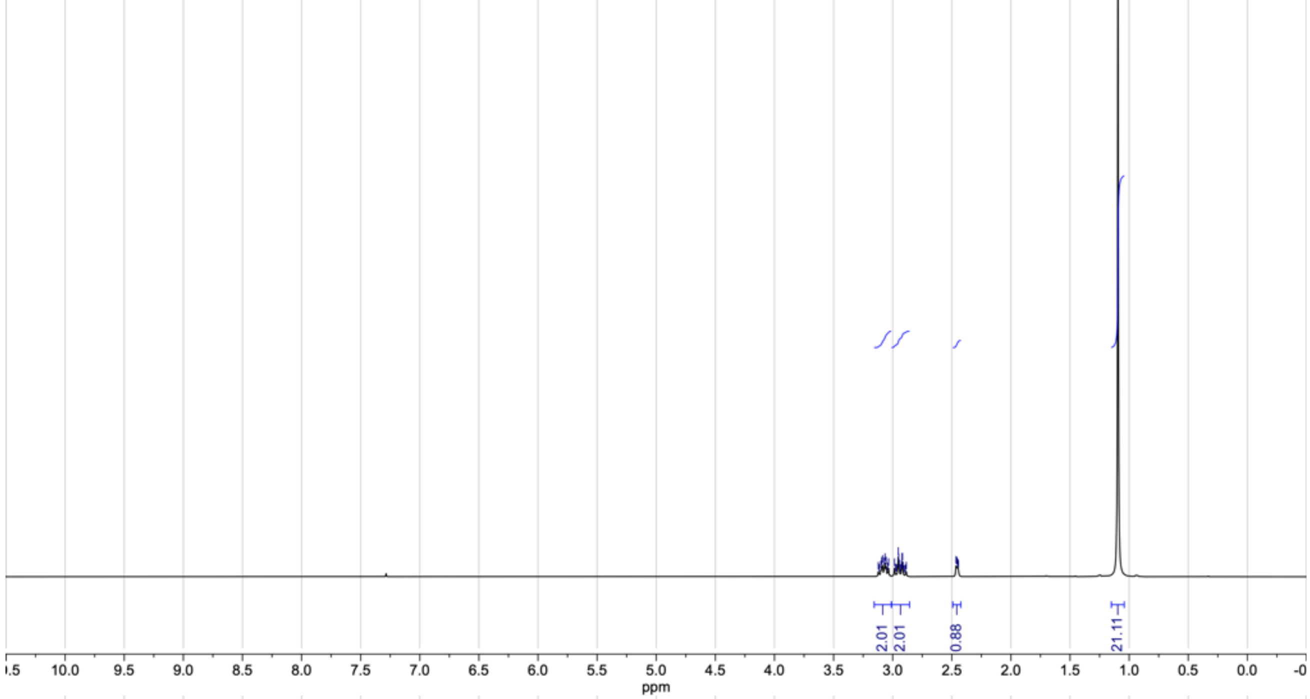

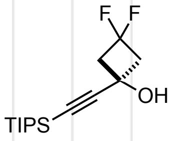

21

^{13}C{^{1}H} NMR (101 MHz, $CDCl_3$)

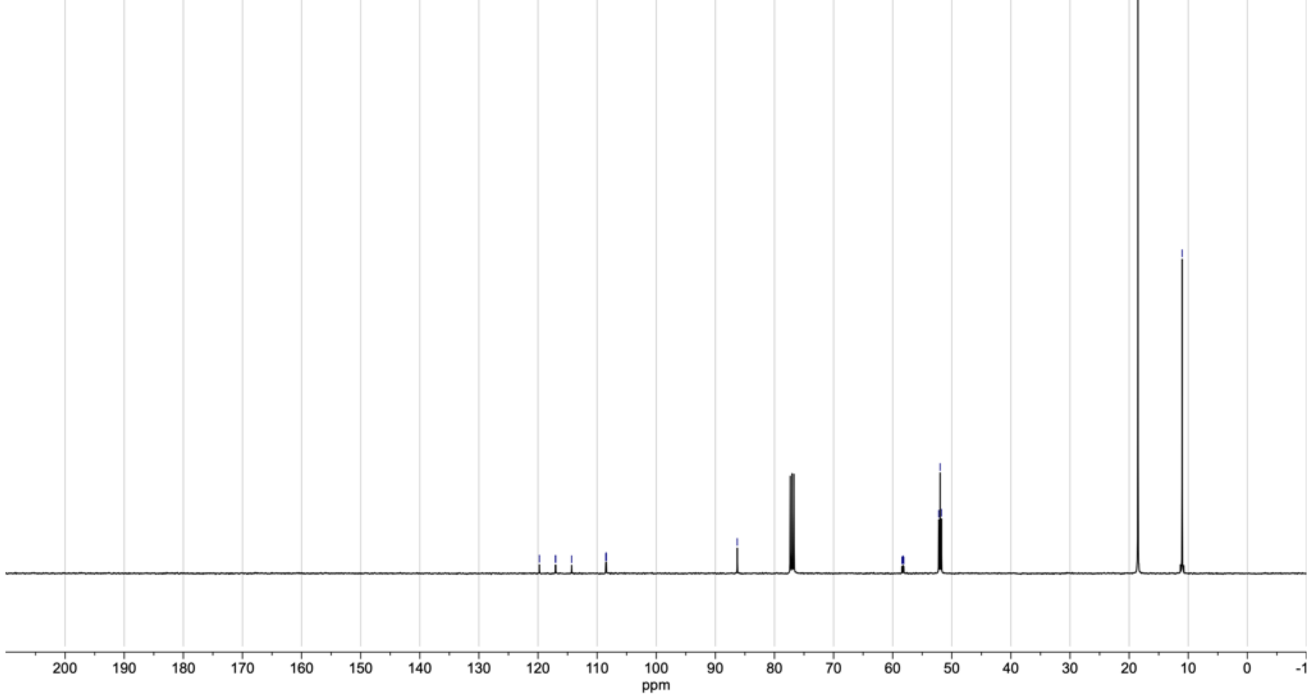

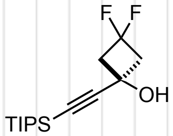

21

^{19}F NMR (377 MHz, $CDCl_3$)

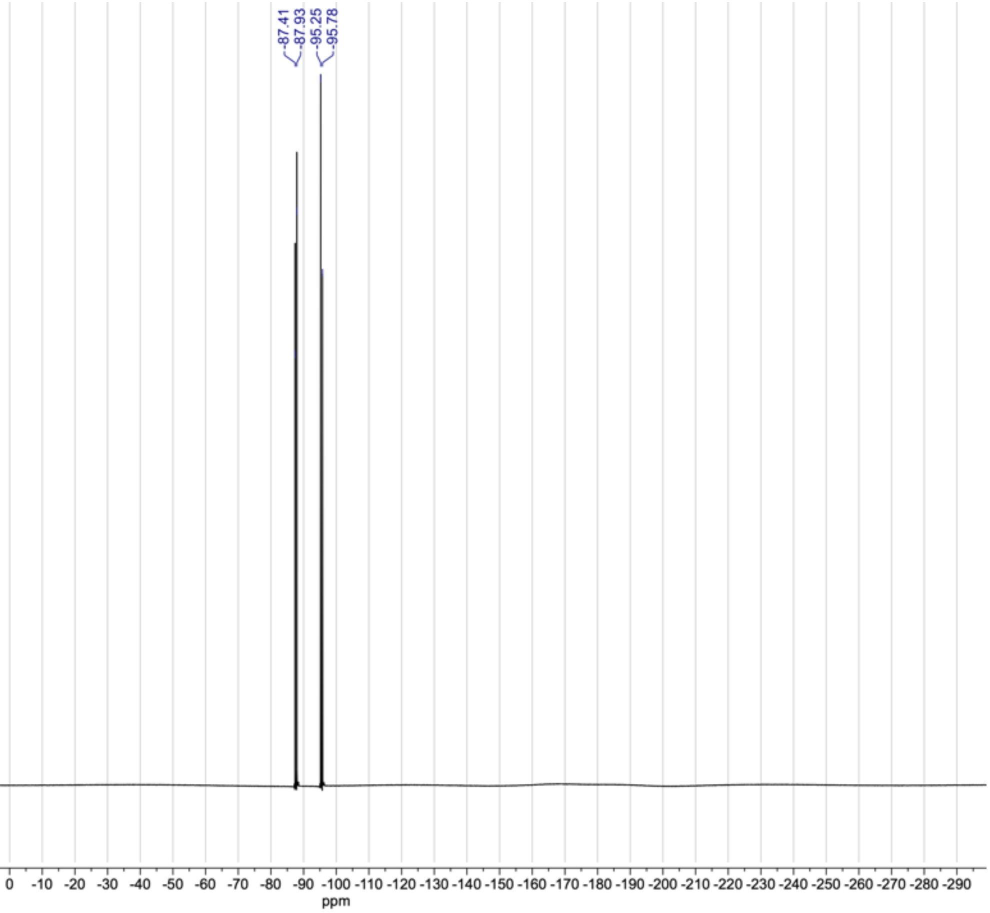

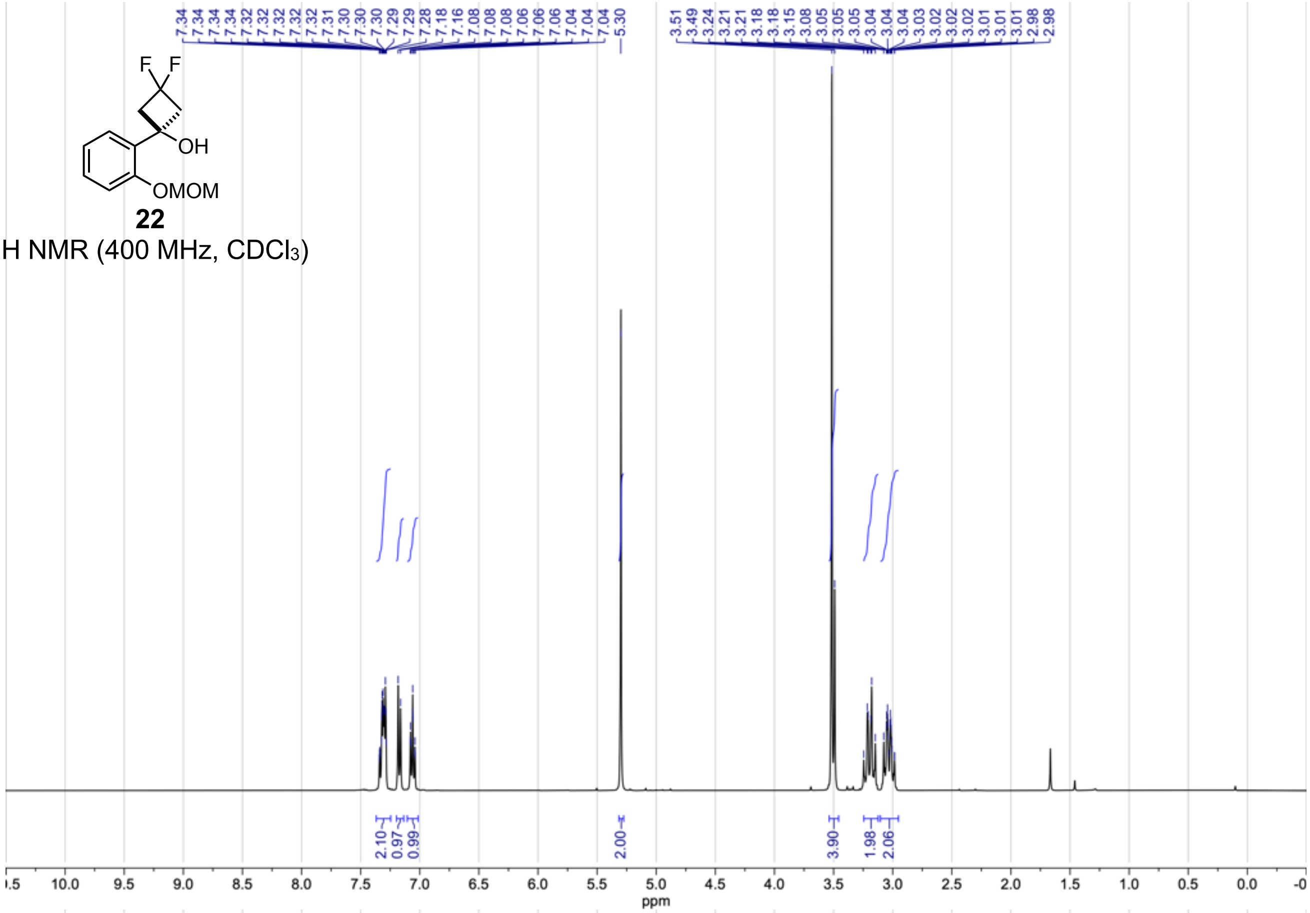

7.34 7.34 7.34 7.34 7.32 7.32 7.32 7.32 7.31 7.30 7.30 7.29 7.29 7.28 7.18 7.16 7.08 7.08 7.08 7.06 7.06 7.06 7.04 7.04 7.04
5.30
3.51 3.49 3.24 3.21 3.21 3.18 3.18 3.15 3.08 3.05 3.05 3.05 3.04 3.04 3.04 3.03 3.02 3.02 3.02 3.01 3.01 3.01 2.98 2.98
F
F
OH
OMOM
22
^{1}H NMR (400 MHz, $CDCl_3$)
2.10
0.97
0.99
2.00
3.90
1.98
2.06
ppm

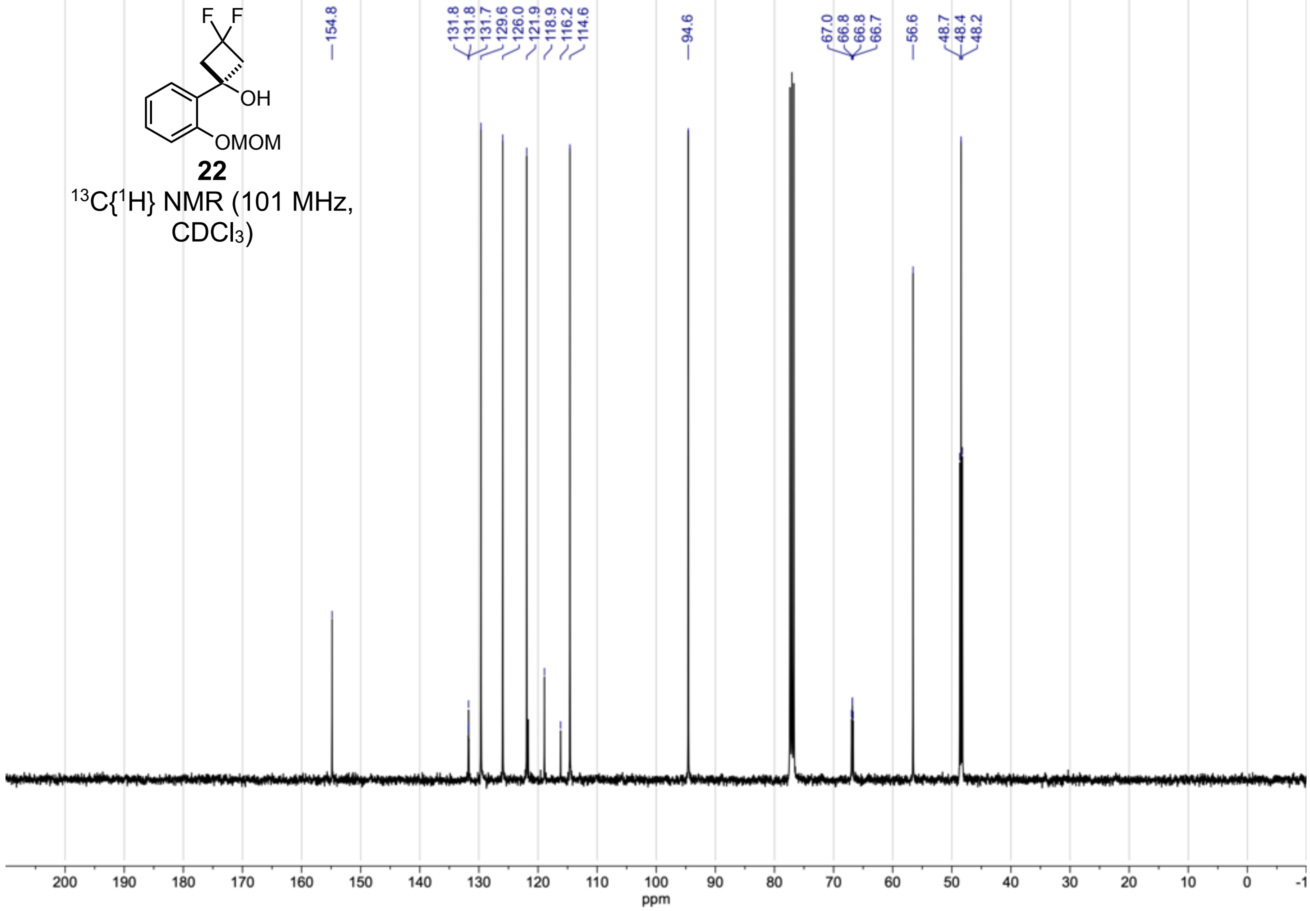

154.8
131.8 131.8 131.7 129.6 126.0 121.9 118.9 116.2 114.6
94.6
67.0 66.8 66.8 66.7
56.6
48.7 48.4 48.2
F
F
OH
OMOM
22
$^{13}C\{^{1}H\}$ NMR (101 MHz, $CDCl_3$)
ppm

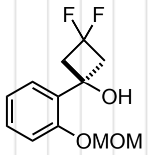

22

^{19}F NMR (377 MHz, $CDCl_3$)

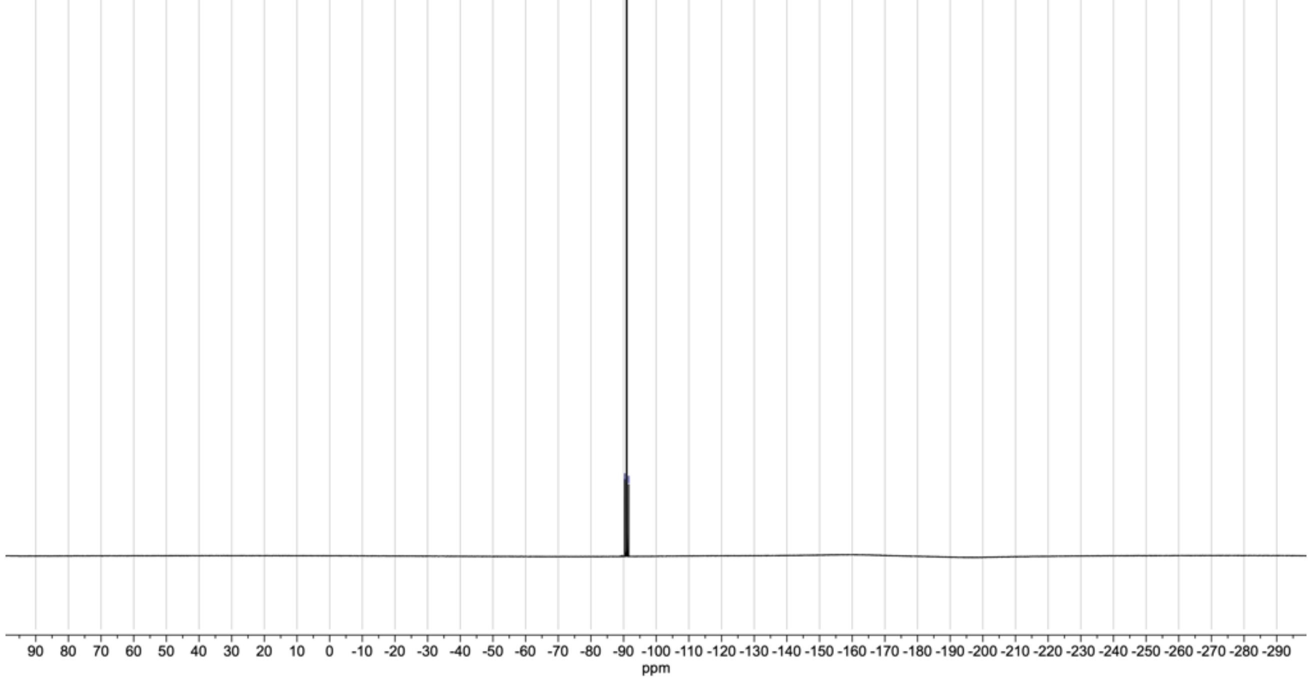

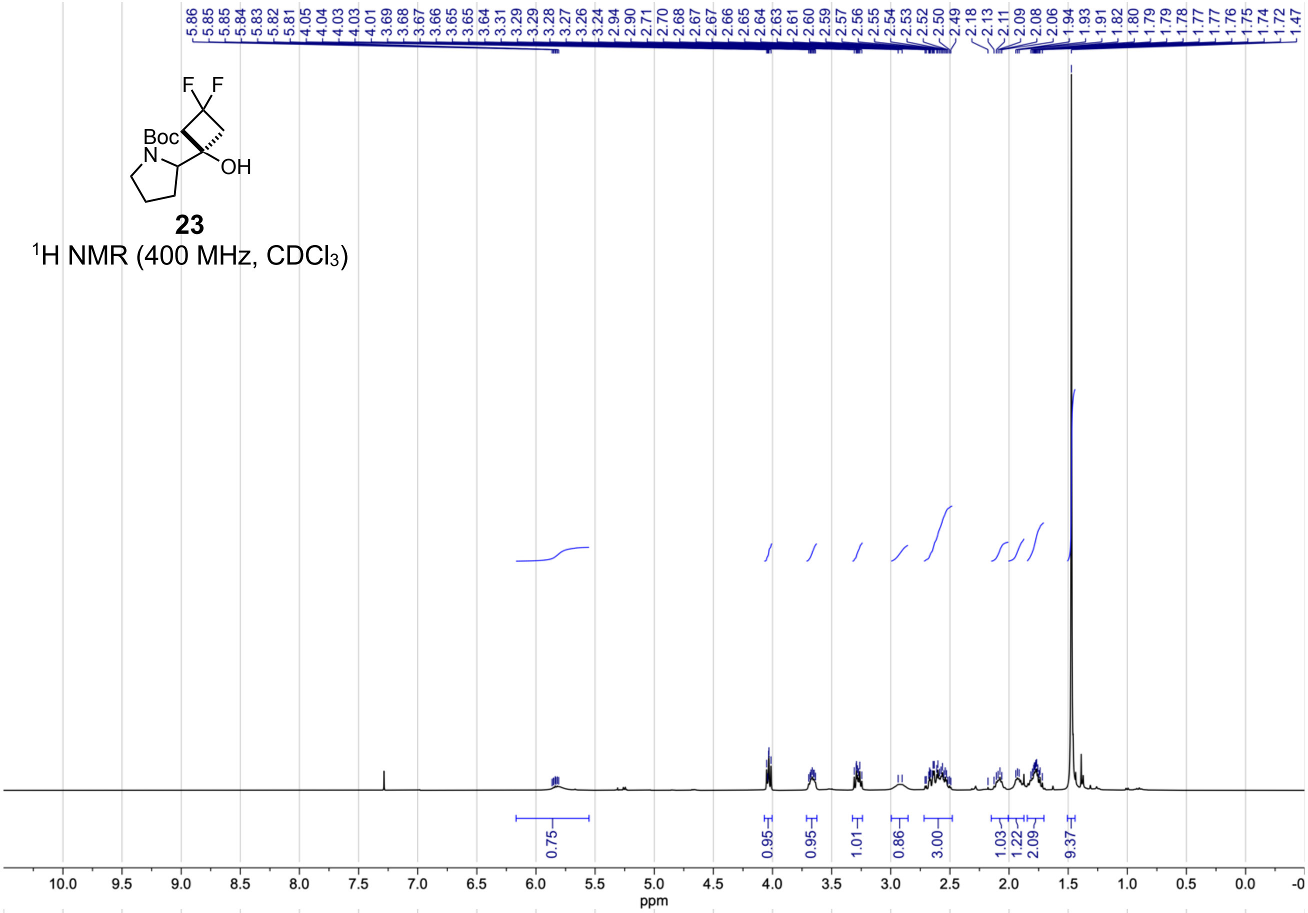
Boc
N
F
F
OH
23
^{1}H NMR (400 MHz, CDCl$_3$)
0.75
0.95
0.95
1.01
0.86
3.00
1.03
1.22
2.09
9.37
ppm

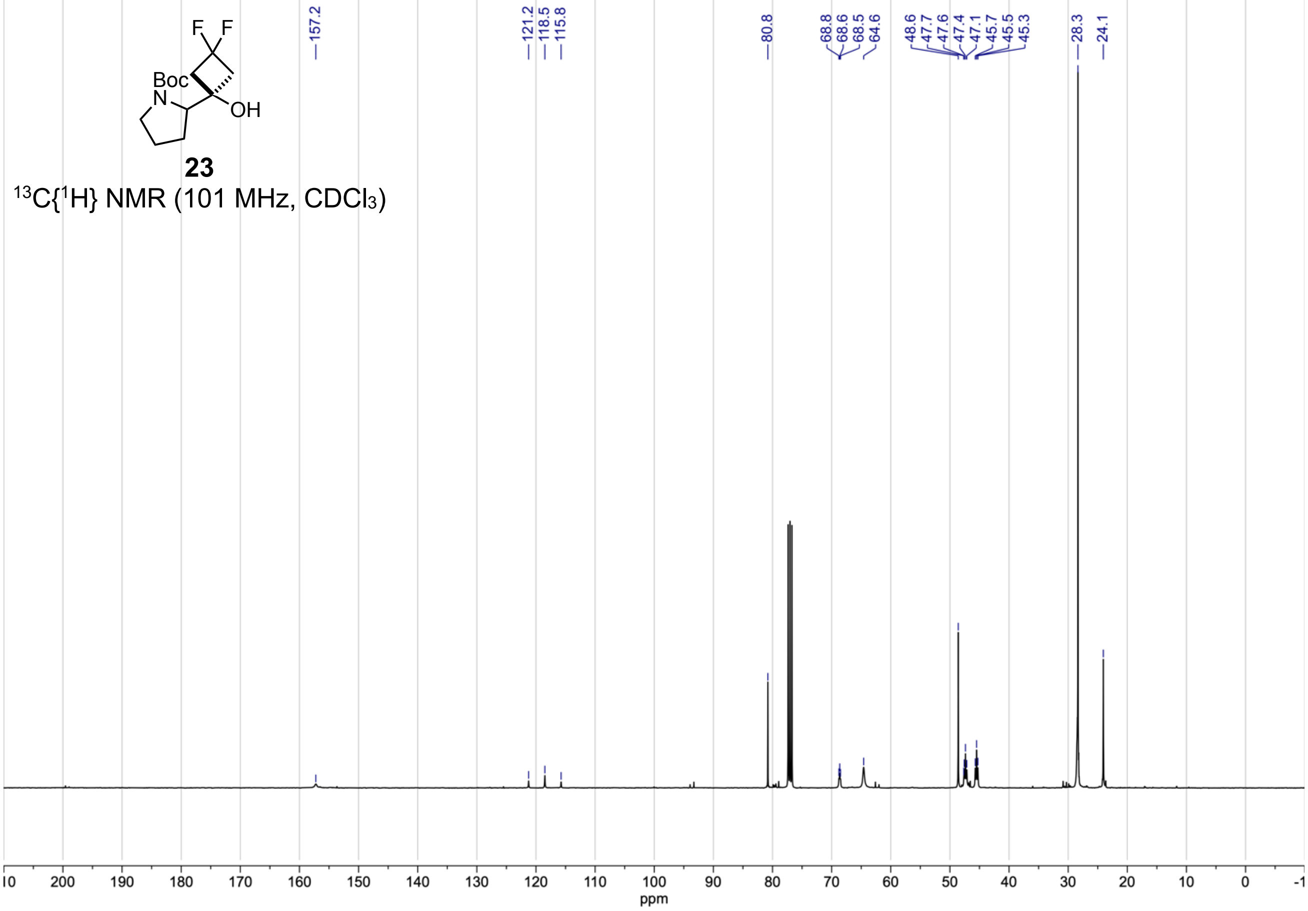
Boc
N
F
F
OH
23
^{13}C{^{1}H} NMR (101 MHz, CDCl$_3$)
157.2
121.2
118.5
115.8
80.8
68.8
68.6
68.5
64.6
48.6
47.7
47.6
47.4
47.1
45.7
45.5
45.3
28.3
24.1
ppm

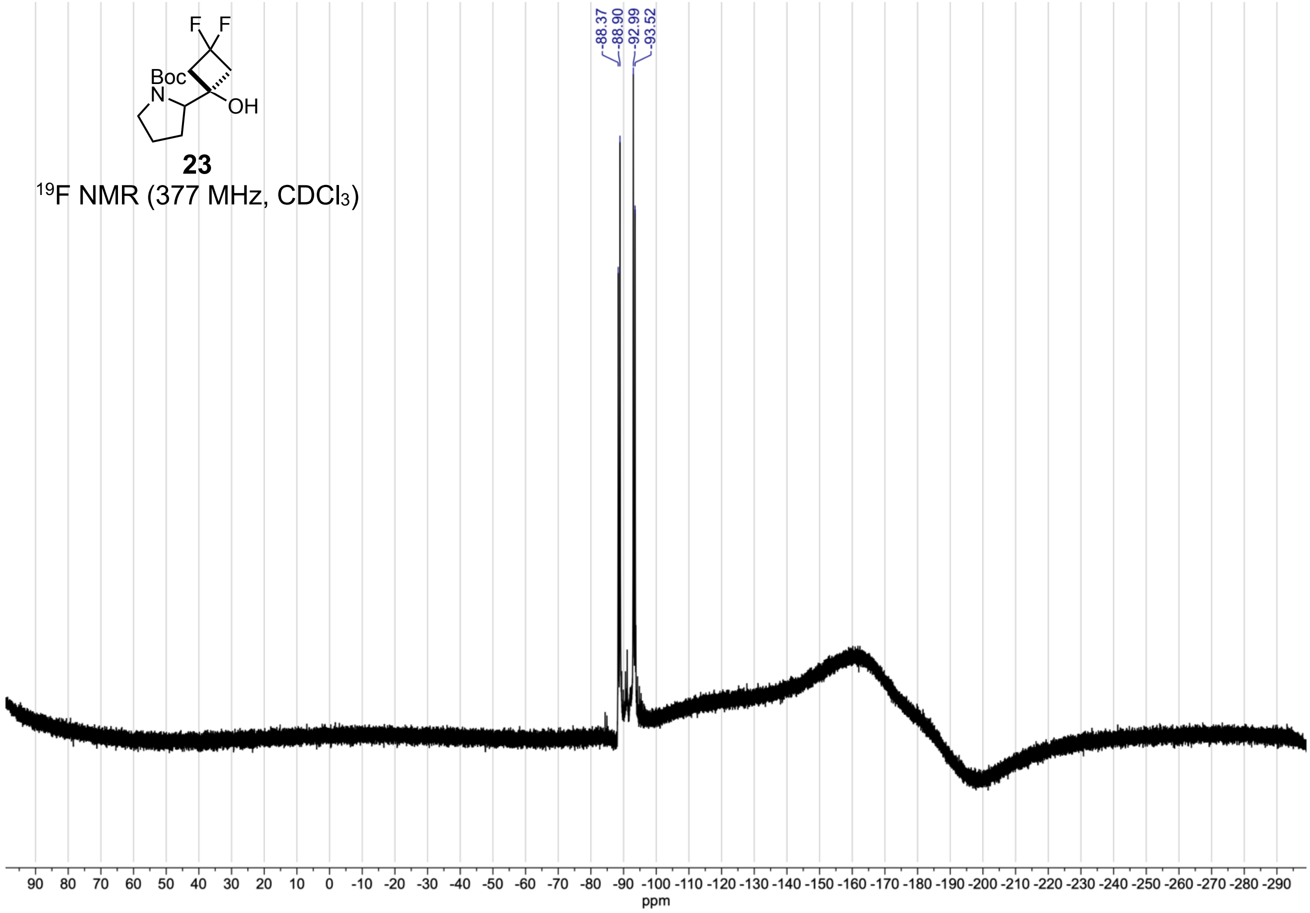
F
F
Boc
N
OH
23
19F NMR (377 MHz, CDCl3)
-88.37
-88.90
-92.99
-93.52
90 80 70 60 50 40 30 20 10 0 -10 -20 -30 -40 -50 -60 -70 -80 -90 -100 -110 -120 -130 -140 -150 -160 -170 -180 -190 -200 -210 -220 -230 -240 -250 -260 -270 -280 -290
ppm

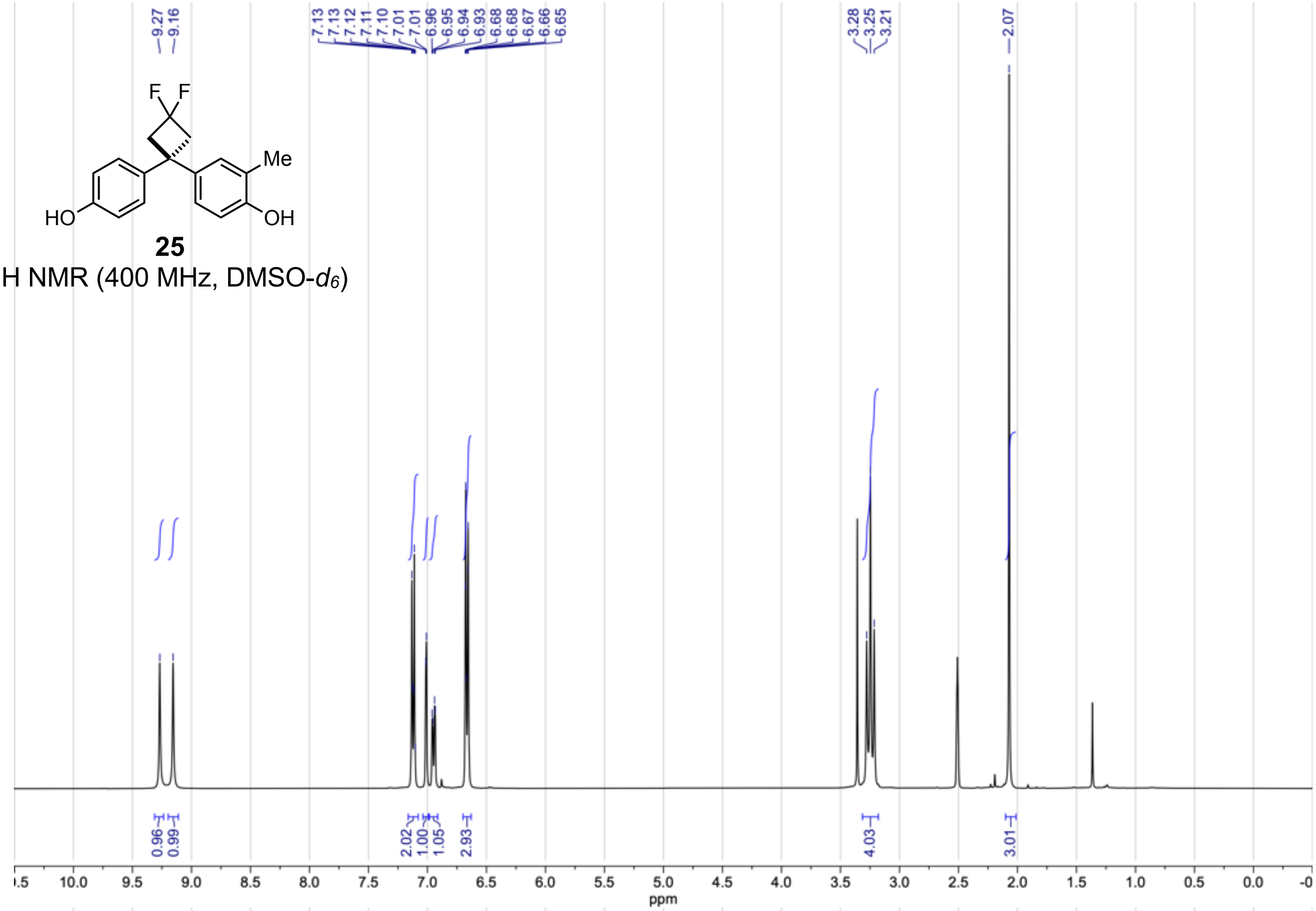

25

^{1}H NMR (400 MHz, DMSO-d_6)

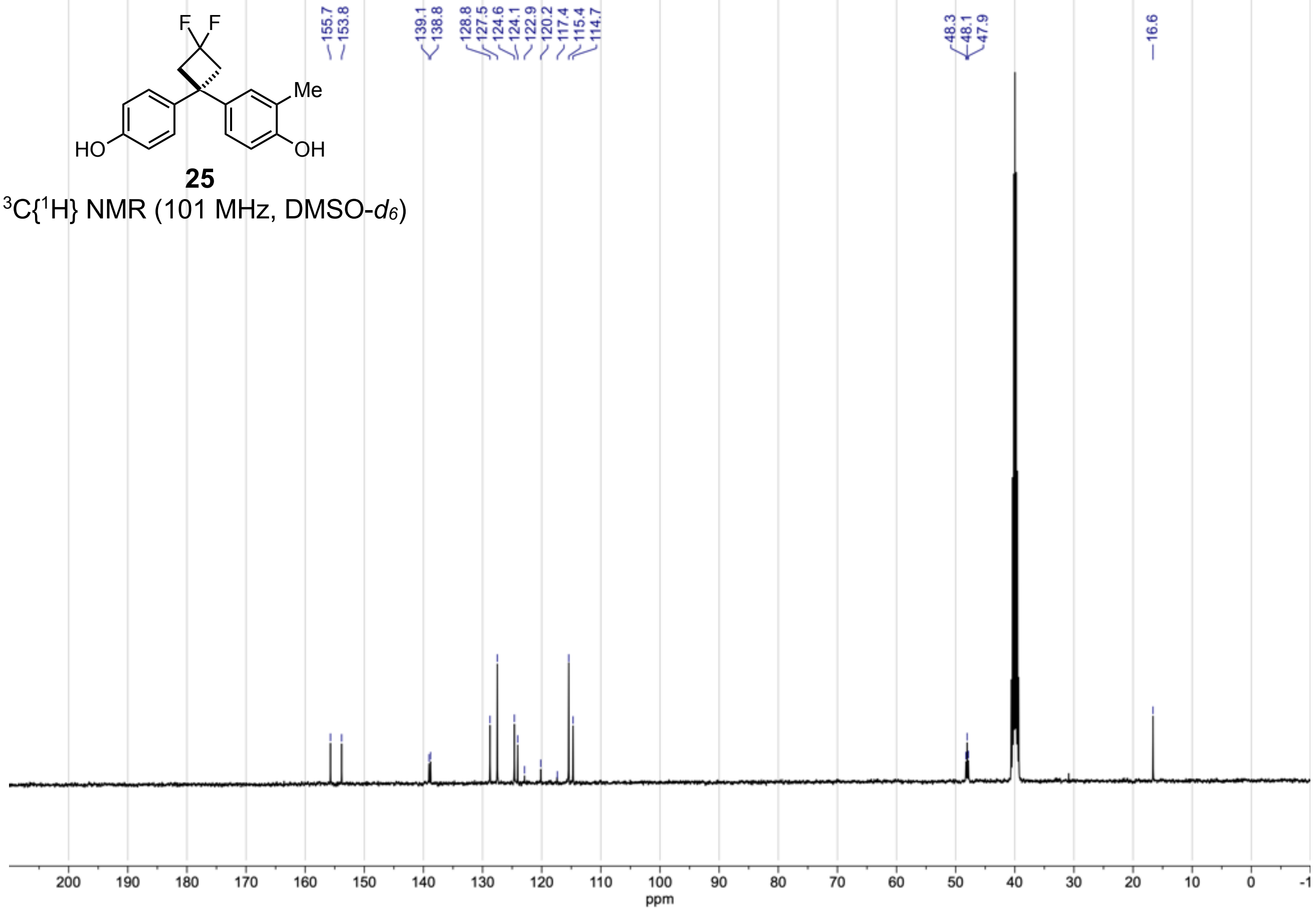

25

^{13}C{^{1}H} NMR (101 MHz, DMSO-d_6)

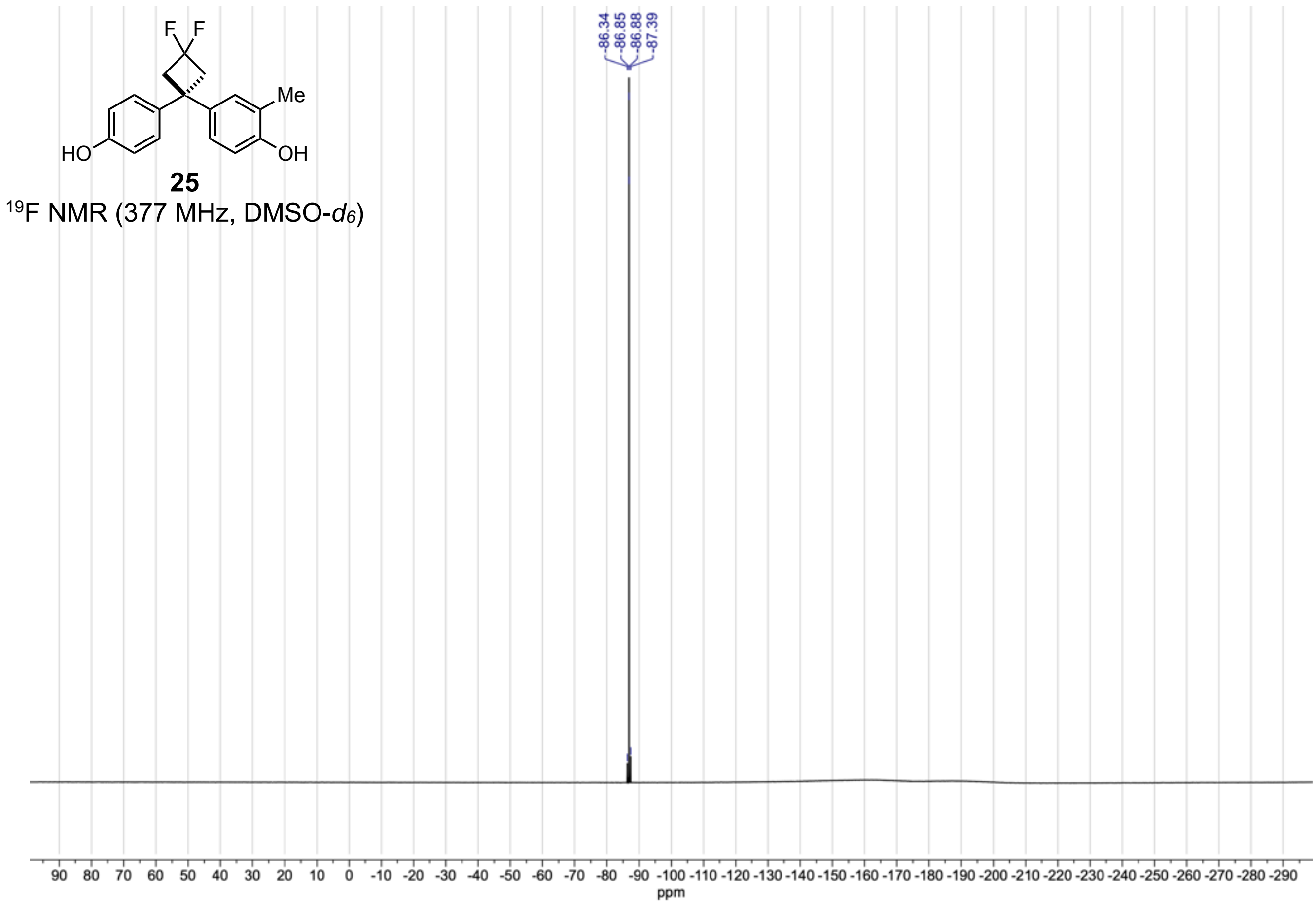
F F
Me
HO
OH
25
^{19}F NMR (377 MHz, DMSO-d_6)
-86.34
-86.85
-86.88
-87.39
ppm

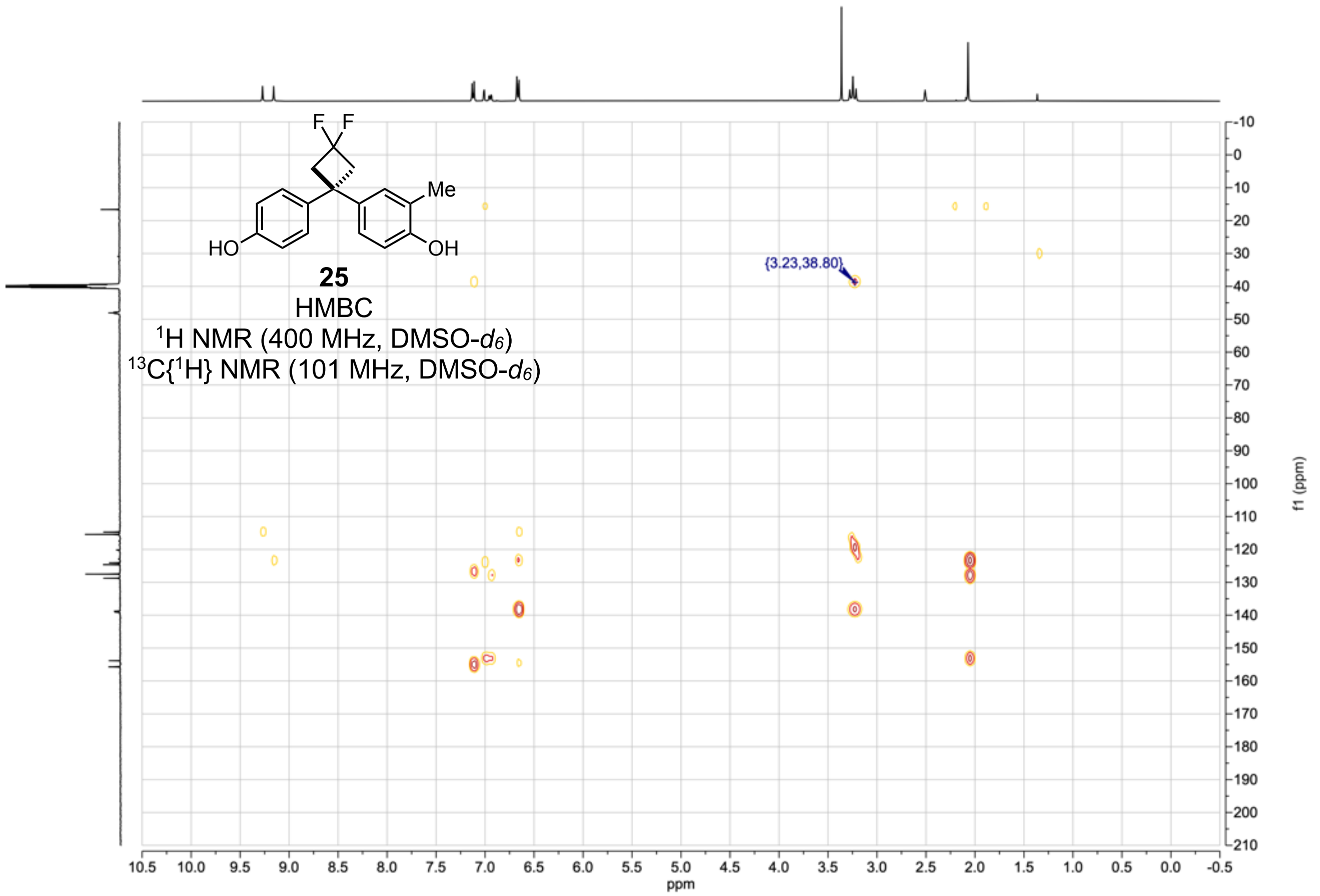
F F
Me
HO
OH
25
HMBC
^{1}H NMR (400 MHz, DMSO-d_6)
^{13}C{^{1}H} NMR (101 MHz, DMSO-d_6)
{3.23,38.80}
ppm
f1 (ppm)

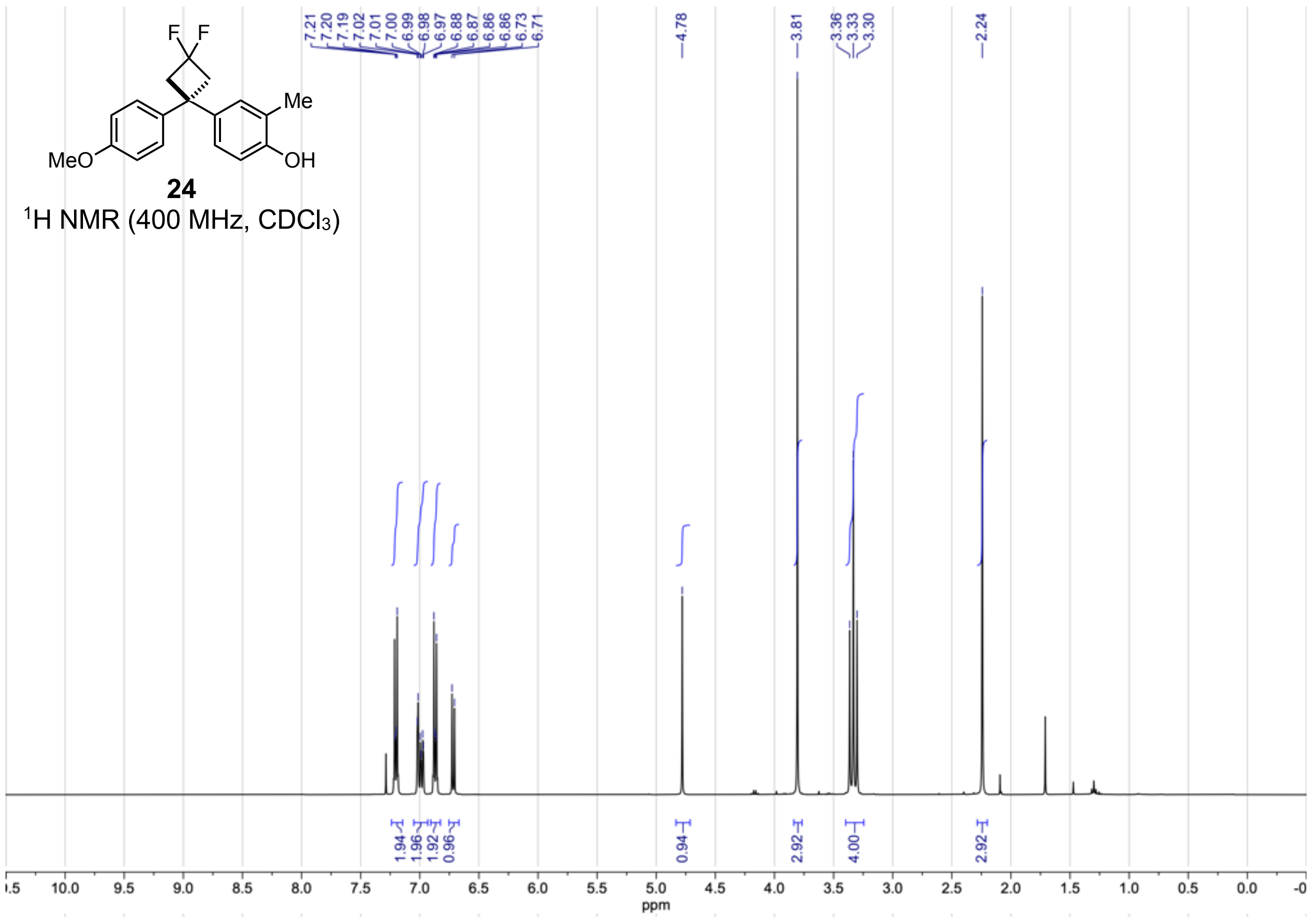
F F
Me
MeO
OH
24
^{1}H NMR (400 MHz, CDCl$_3$)
7.21 7.20 7.19 7.02 7.01 7.00 6.99 6.98 6.97 6.88 6.87 6.86 6.86 6.73 6.71
4.78
3.81
3.36 3.33 3.30
2.24
1.94 1.96 1.92 0.96
0.94
2.92
4.00
2.92
ppm

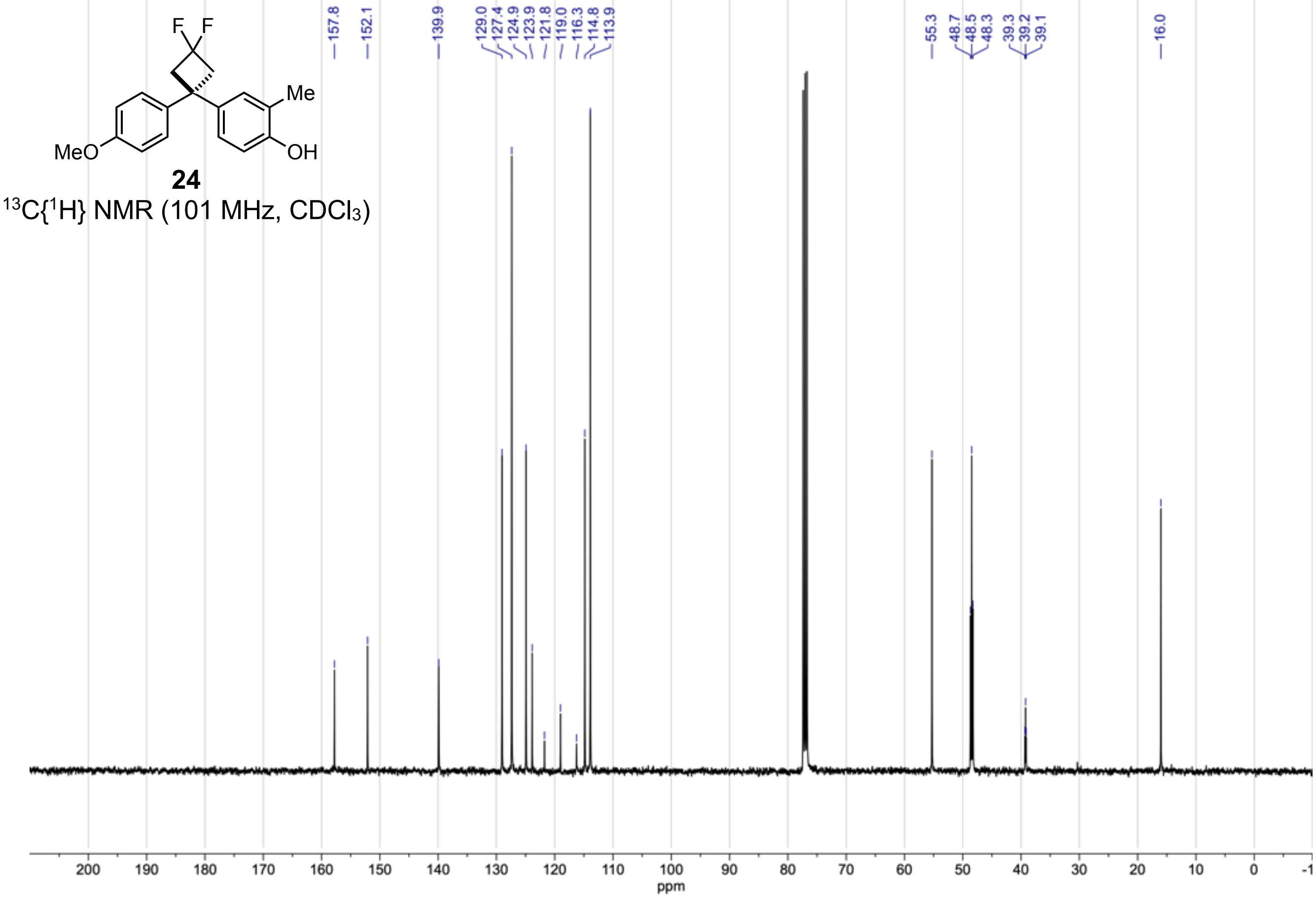
F F
Me
MeO
OH
24
^{13}C{^{1}H} NMR (101 MHz, CDCl$_3$)
157.8
152.1
139.9
129.0 127.4 124.9 123.9 121.8 119.0 116.3 114.8 113.9
55.3
48.7 48.5 48.3
39.3 39.2 39.1
16.0
ppm

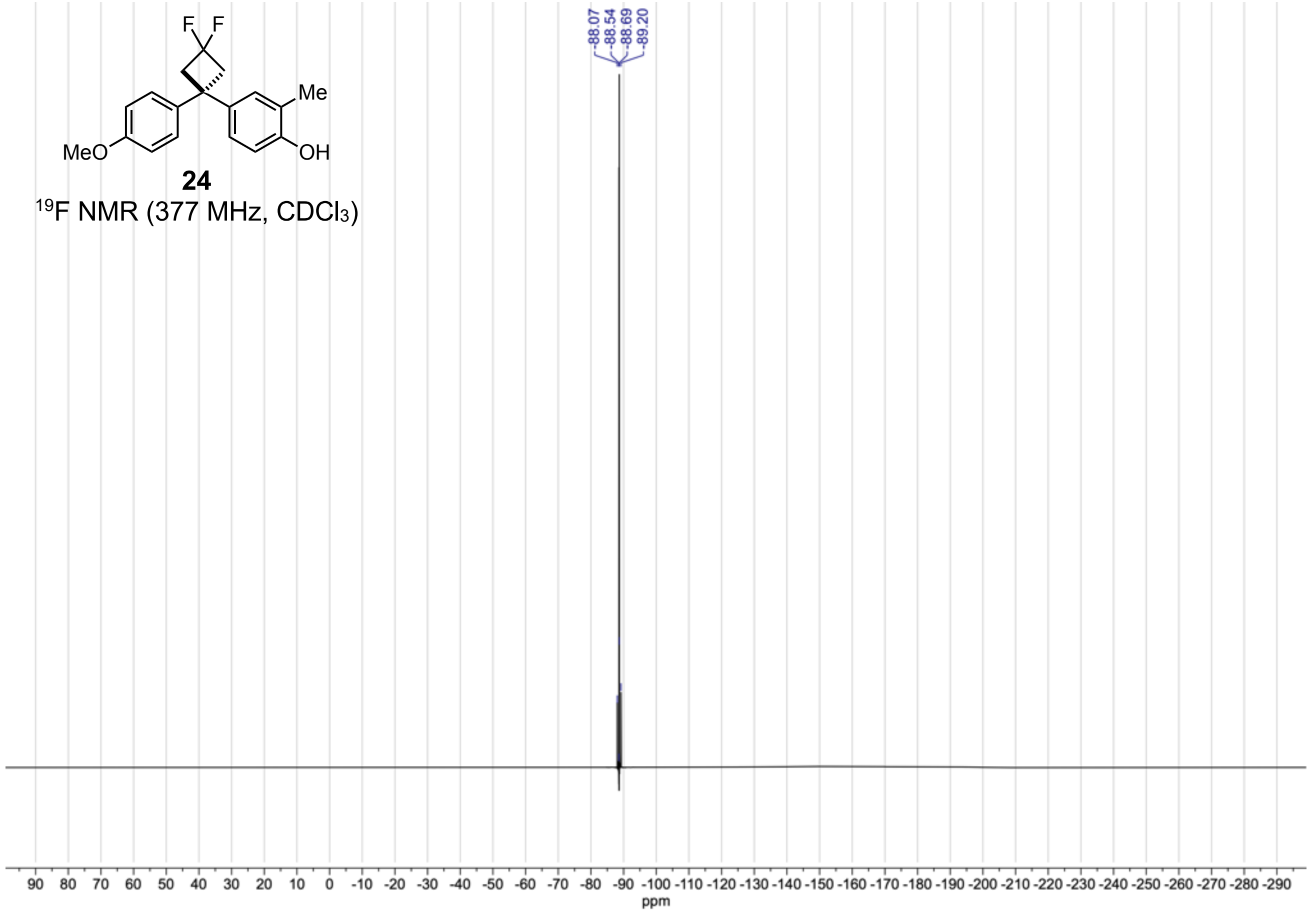
F F
Me
MeO
OH
24
19F NMR (377 MHz, CDCl3)
-88.07
-88.54
-88.69
-89.20
90 80 70 60 50 40 30 20 10 0 -10 -20 -30 -40 -50 -60 -70 -80 -90 -100 -110 -120 -130 -140 -150 -160 -170 -180 -190 -200 -210 -220 -230 -240 -250 -260 -270 -280 -290
ppm

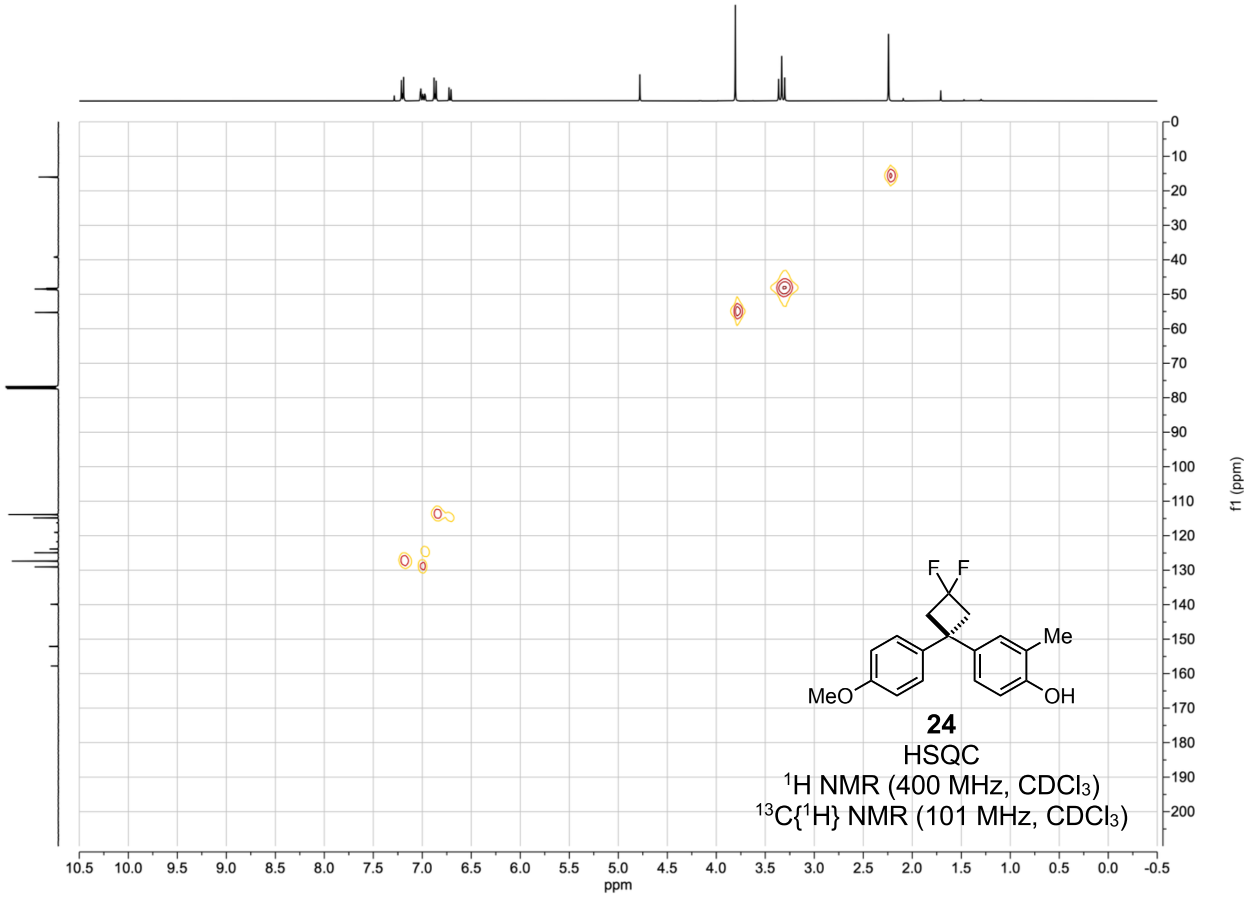
F F
Me
MeO
OH
24
HSQC
1H NMR (400 MHz, CDCl3)
13C{1H} NMR (101 MHz, CDCl3)
f1 (ppm)
0 10 20 30 40 50 60 70 80 90 100 110 120 130 140 150 160 170 180 190 200
10.5 10.0 9.5 9.0 8.5 8.0 7.5 7.0 6.5 6.0 5.5 5.0 4.5 4.0 3.5 3.0 2.5 2.0 1.5 1.0 0.5 0.0 -0.5
ppm

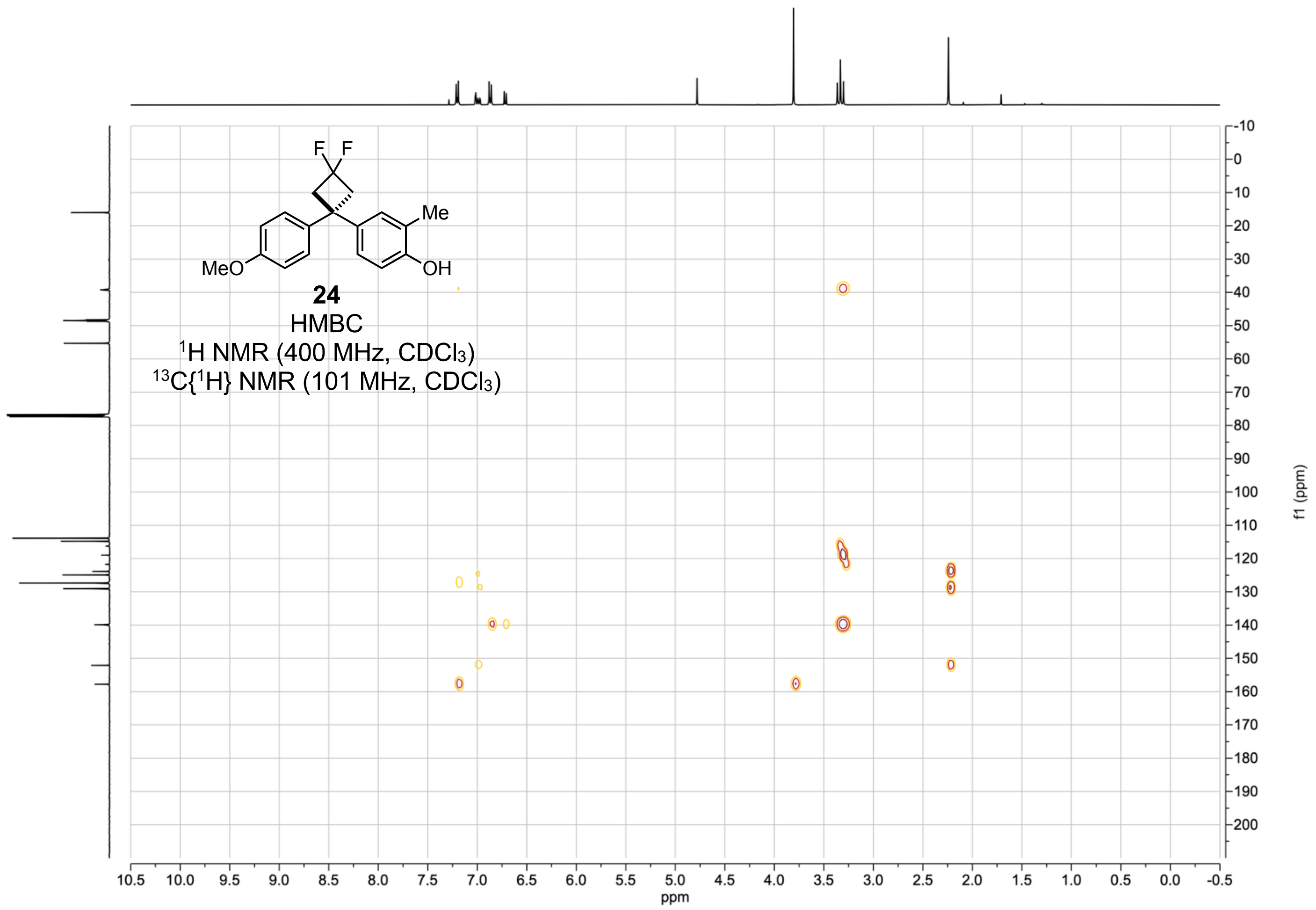
F F
Me
MeO
OH
24
HMBC
1H NMR (400 MHz, CDCl3)
13C{1H} NMR (101 MHz, CDCl3)
ppm
f1 (ppm)

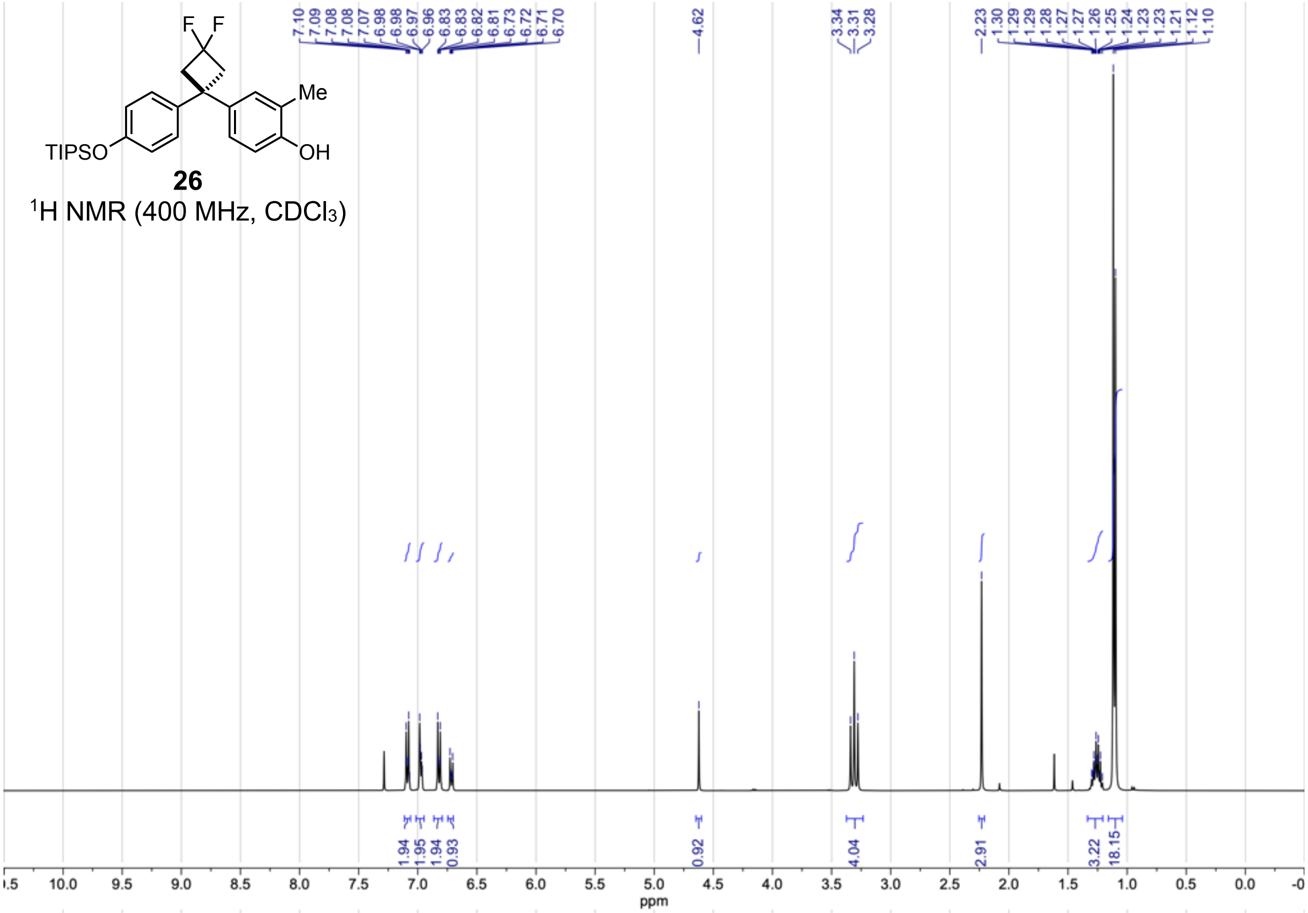
F F
Me
TIPSO
OH
26
1H NMR (400 MHz, CDCl3)
7.10 7.09 7.08 7.08 7.07 6.98 6.98 6.97 6.96 6.83 6.83 6.82 6.81 6.73 6.72 6.71 6.70
4.62
3.34 3.31 3.28
2.23 1.30 1.29 1.29 1.28 1.27 1.27 1.26 1.25 1.24 1.23 1.23 1.21 1.12 1.10
1.94 1.95 1.94 0.93
0.92
4.04
2.91
3.22 18.15
ppm

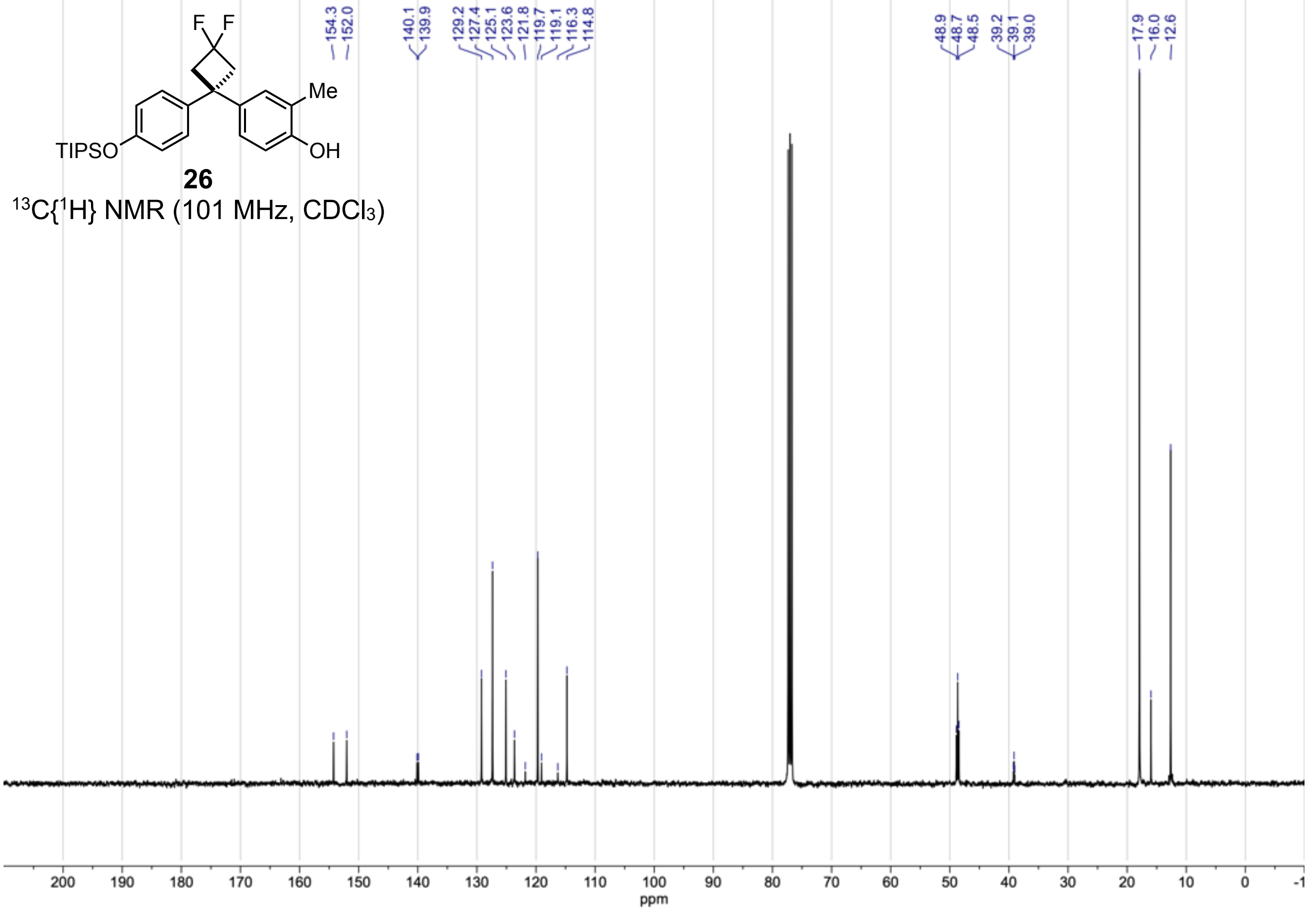
F F
Me
TIPSO
OH
26
13C{1H} NMR (101 MHz, CDCl3)
154.3 152.0
140.1 139.9
129.2 127.4 125.1 123.6 121.8 119.7 119.1 116.3 114.8
48.9 48.7 48.5
39.2 39.1 39.0
17.9 16.0 12.6
ppm

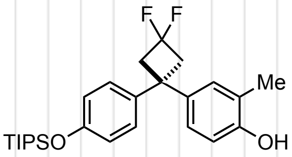

26

^{19}F NMR (377 MHz, $CDCl_3$)

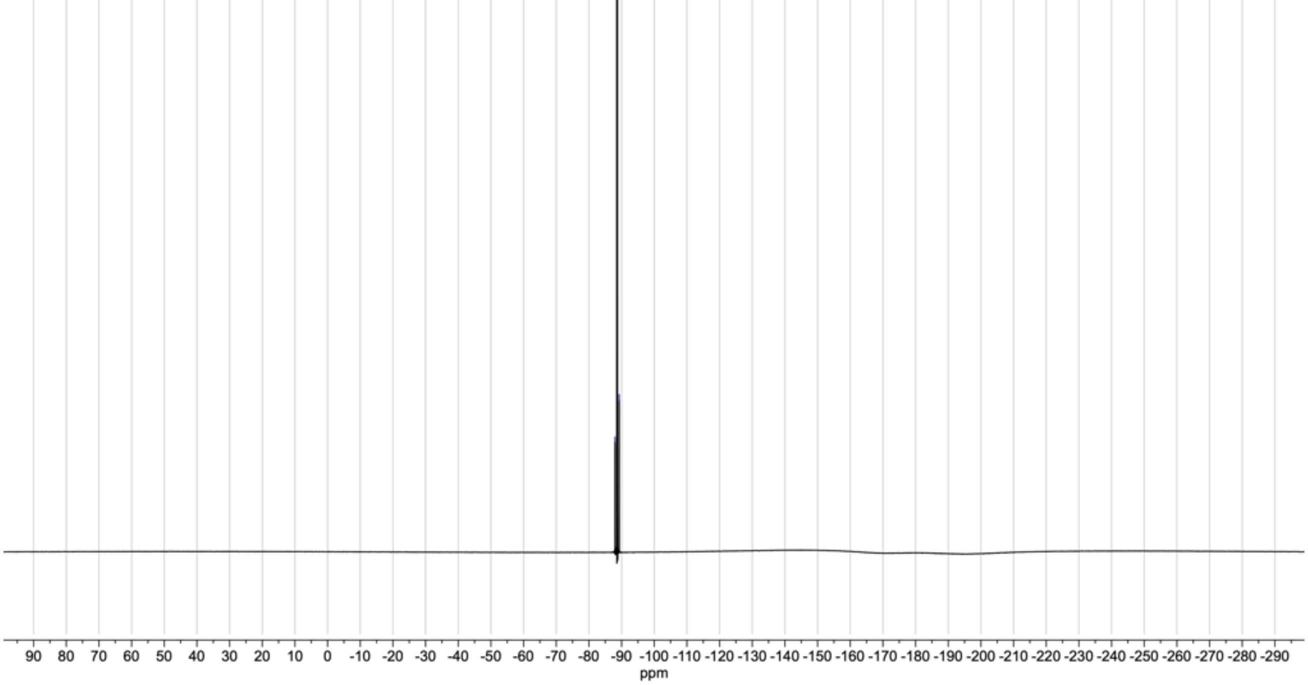

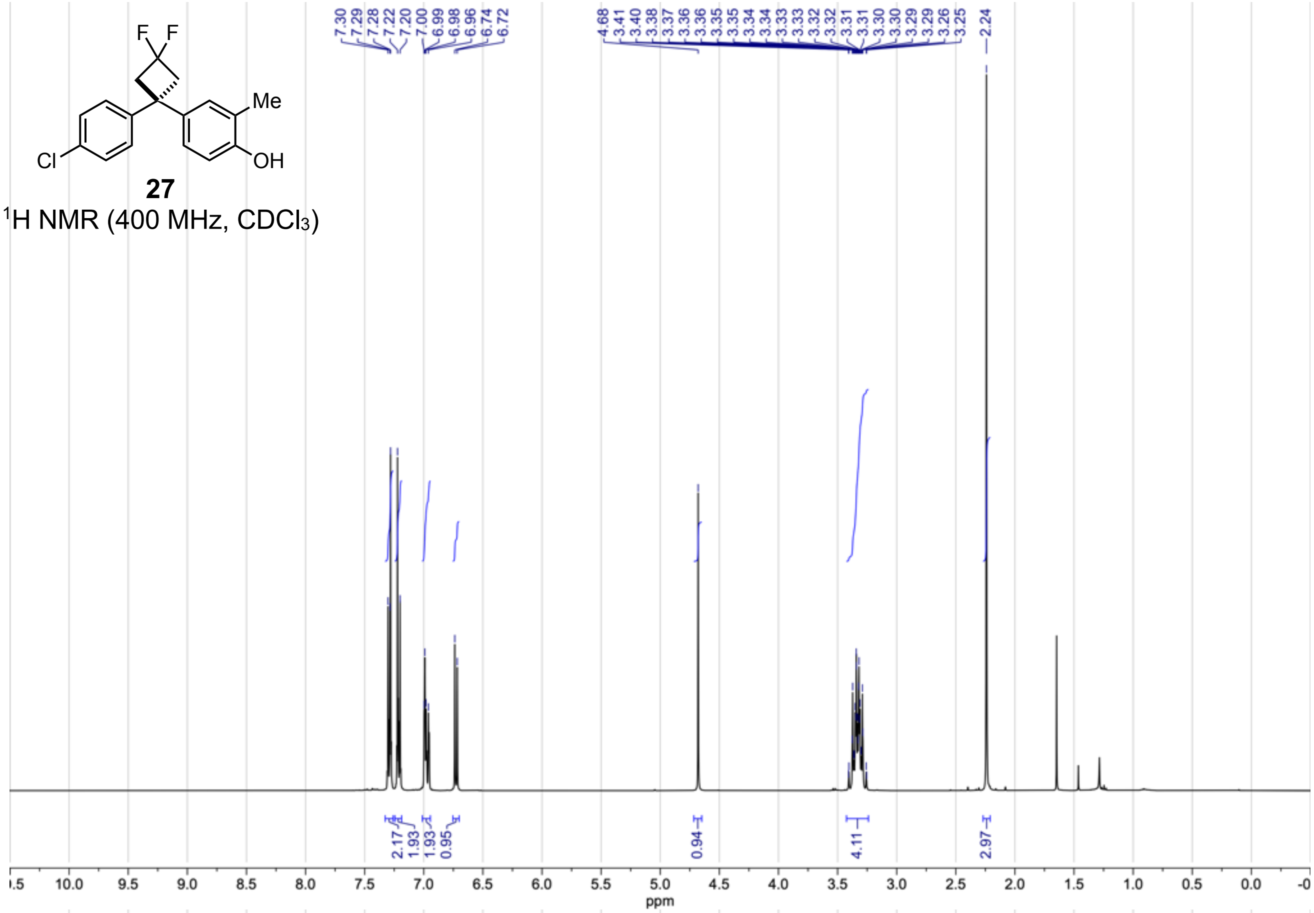
F F
Me
Cl
OH
27
^{1}H NMR (400 MHz, CDCl3)
7.30
7.29
7.28
7.22
7.20
7.00
6.99
6.98
6.96
6.74
6.72
4.68
3.41
3.40
3.38
3.37
3.36
3.36
3.35
3.35
3.34
3.34
3.33
3.33
3.32
3.32
3.31
3.31
3.30
3.30
3.29
3.29
3.26
3.25
2.24
2.17
1.93
1.93
0.95
0.94
4.11
2.97
ppm

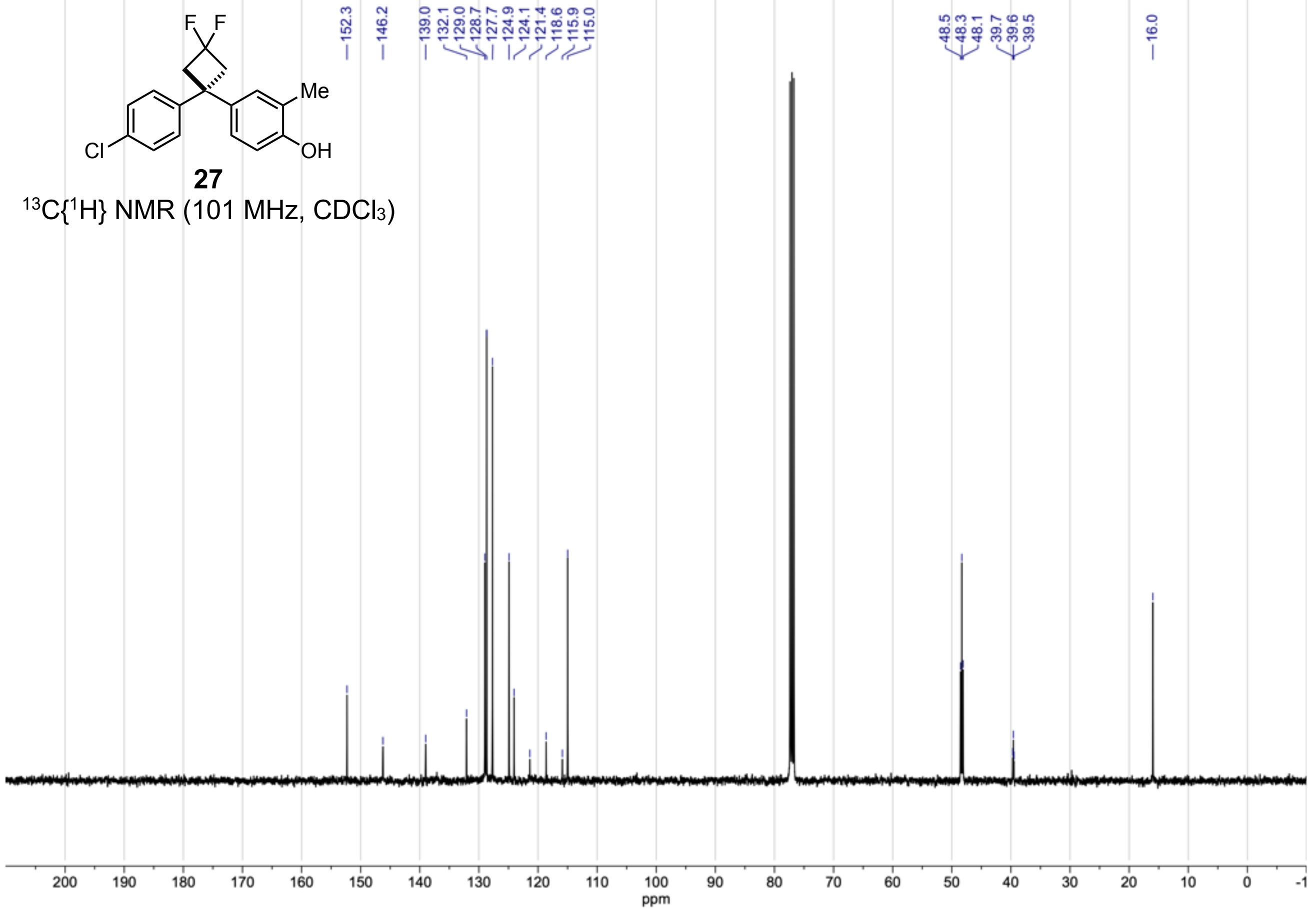
F F
Me
Cl
OH
27
^{13}C{^{1}H} NMR (101 MHz, CDCl3)
152.3
146.2
139.0
132.1
129.0
128.7
127.7
124.9
124.1
121.4
118.6
115.9
115.0
48.5
48.3
48.1
39.7
39.6
39.5
16.0
ppm

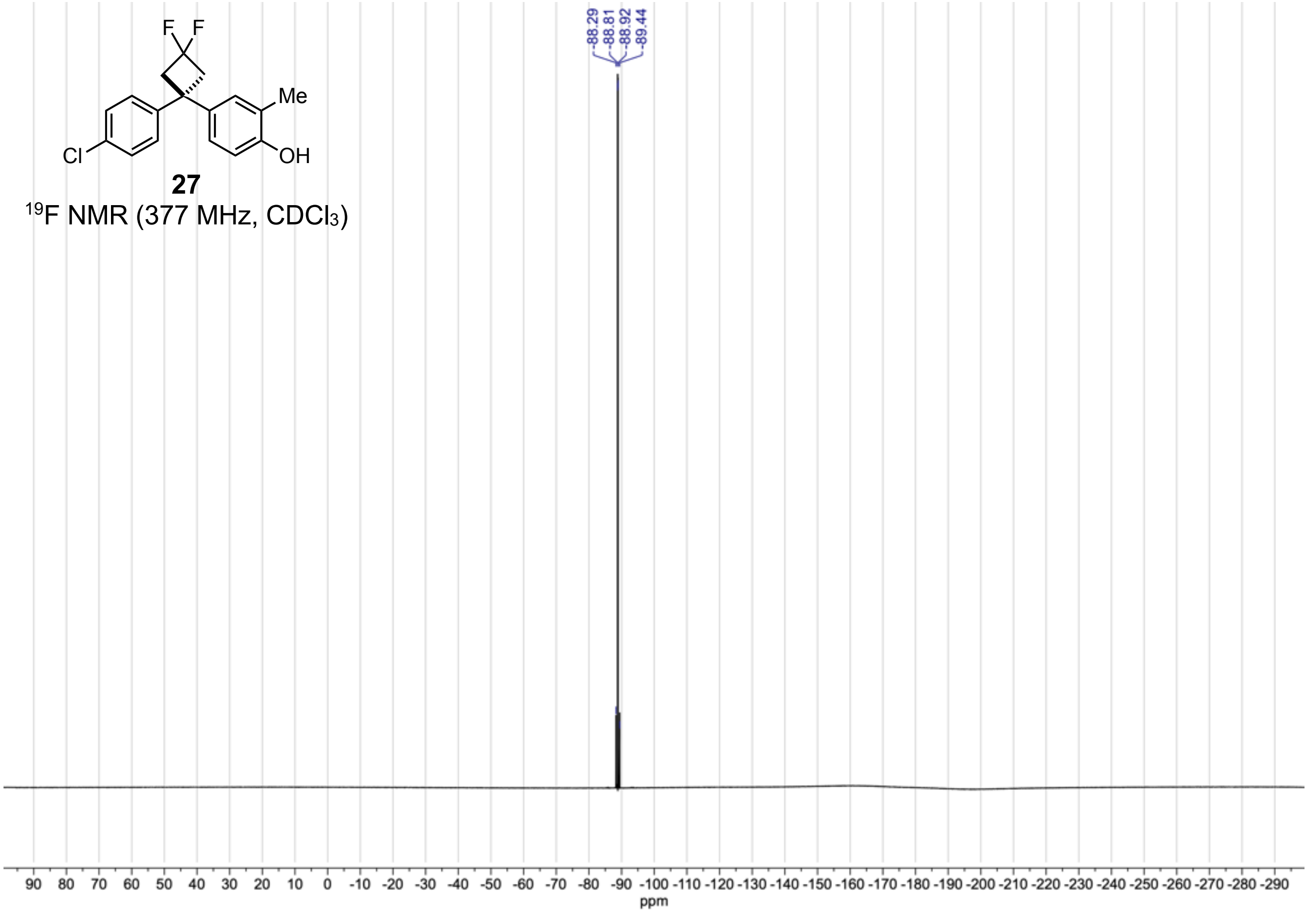

27

^{19}F NMR (377 MHz, $CDCl_3$)

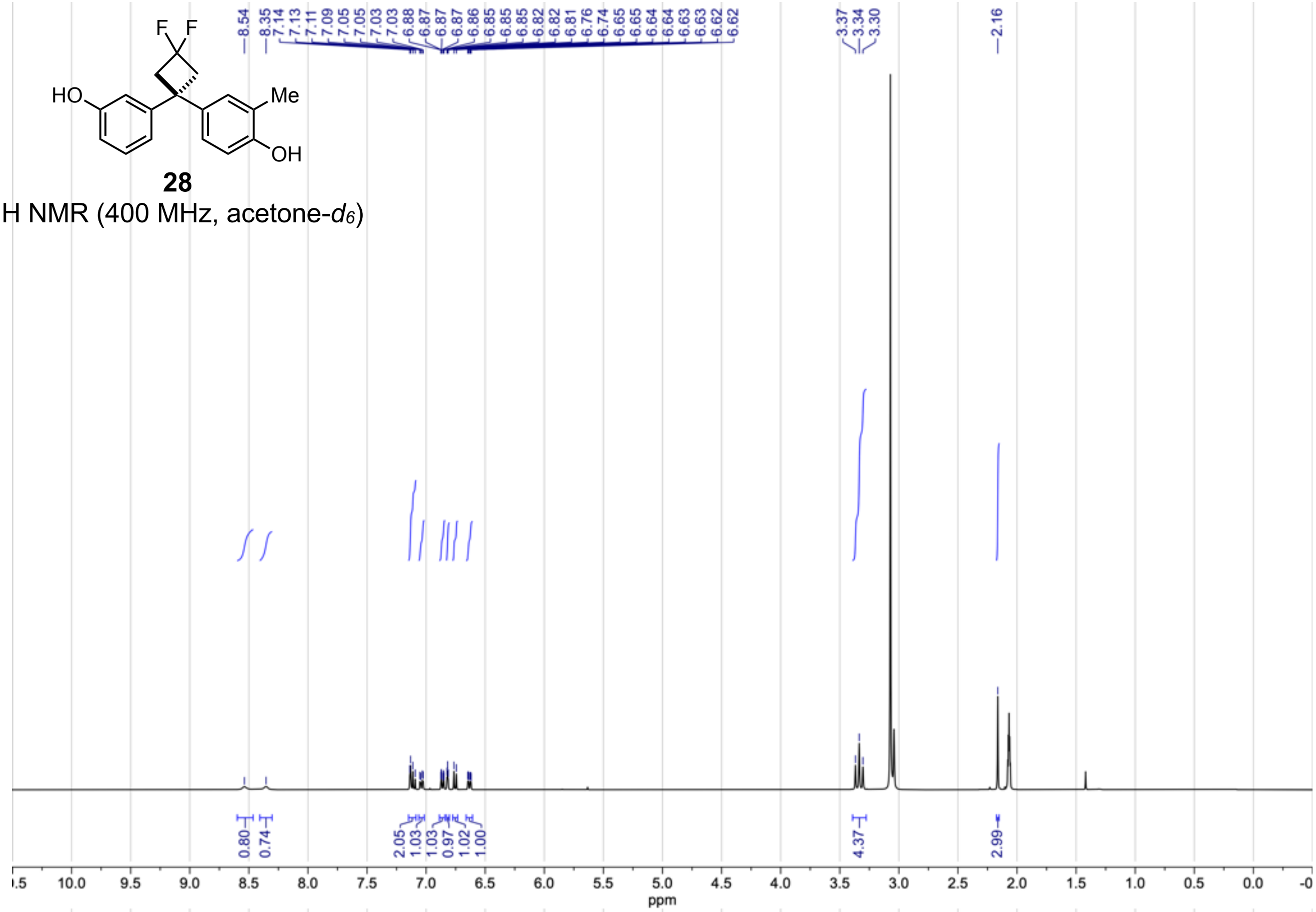

28
^{1}H NMR (400 MHz, acetone-d_6)

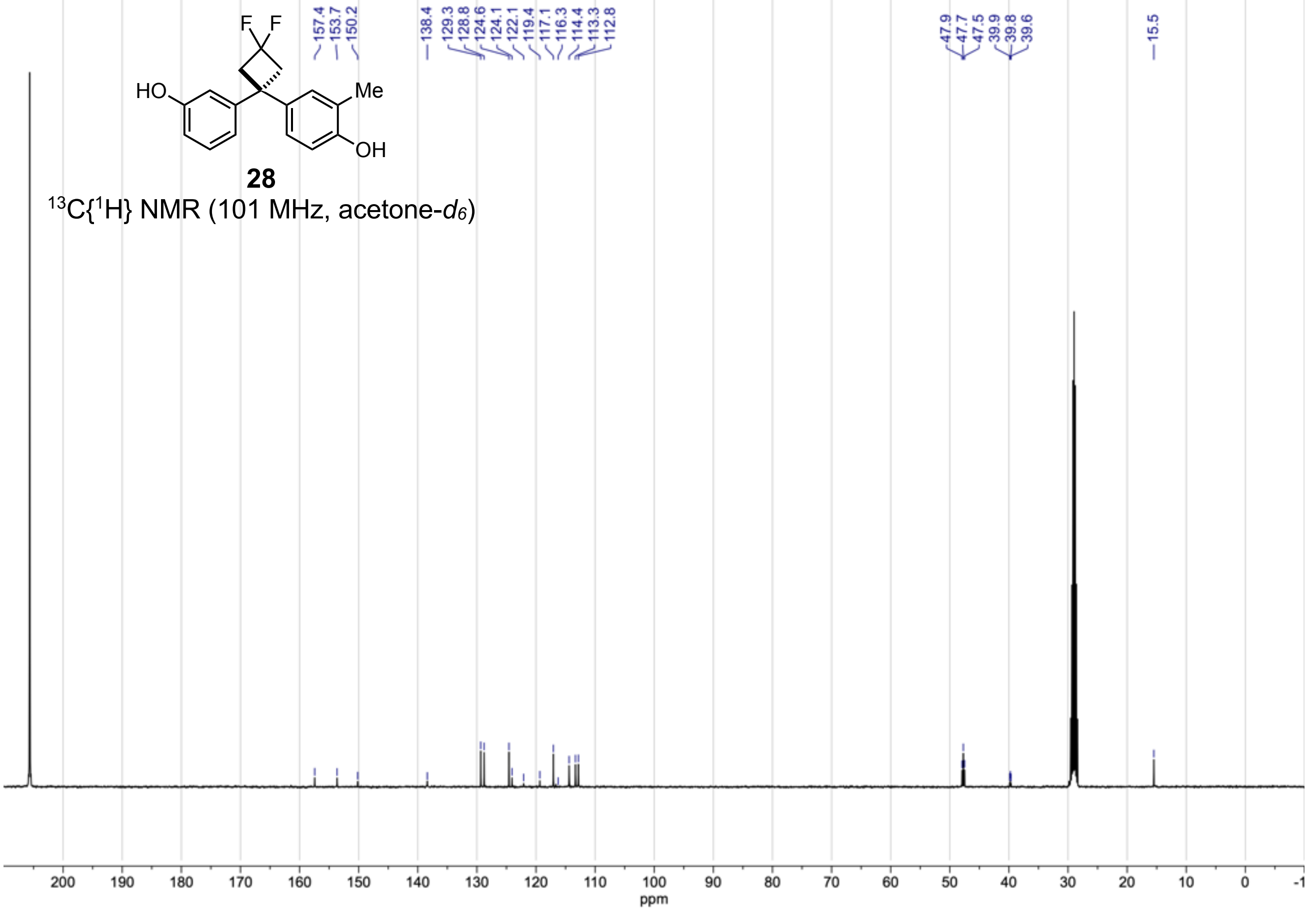

28
^{13}C{^{1}H} NMR (101 MHz, acetone-d_6)

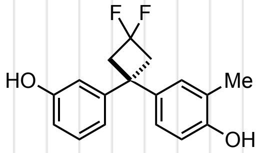

28

^{19}F NMR (377 MHz, acetone-d_6)

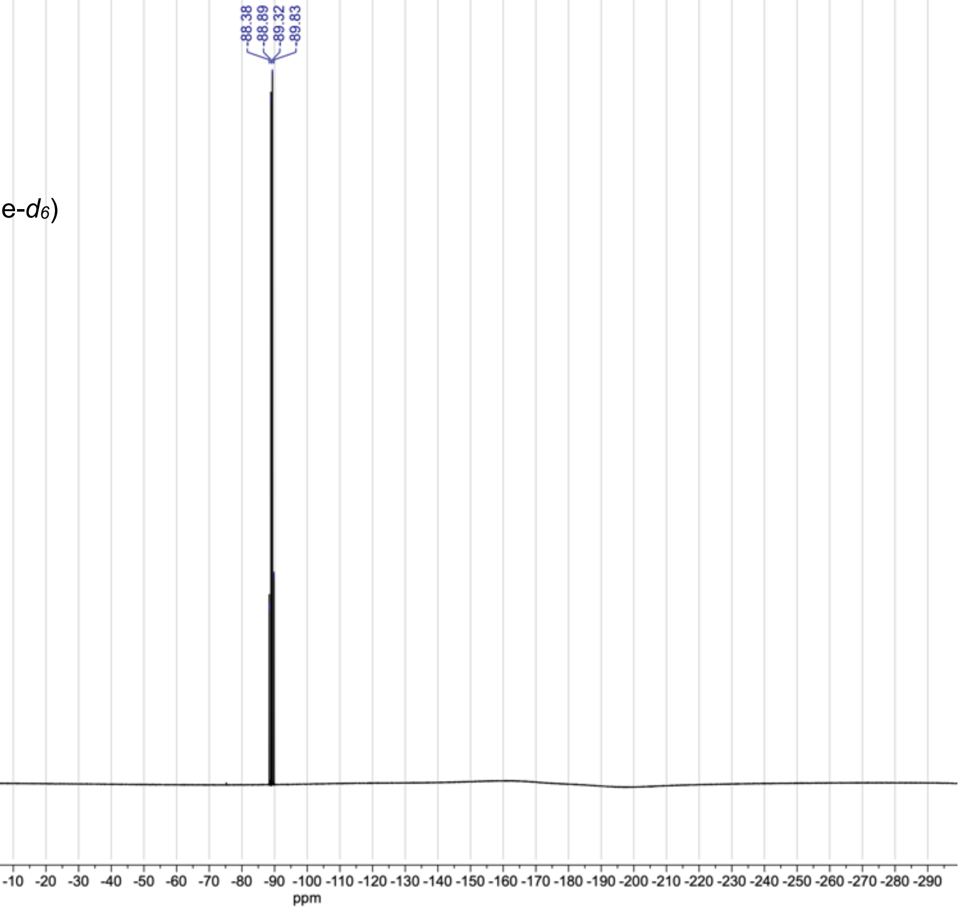

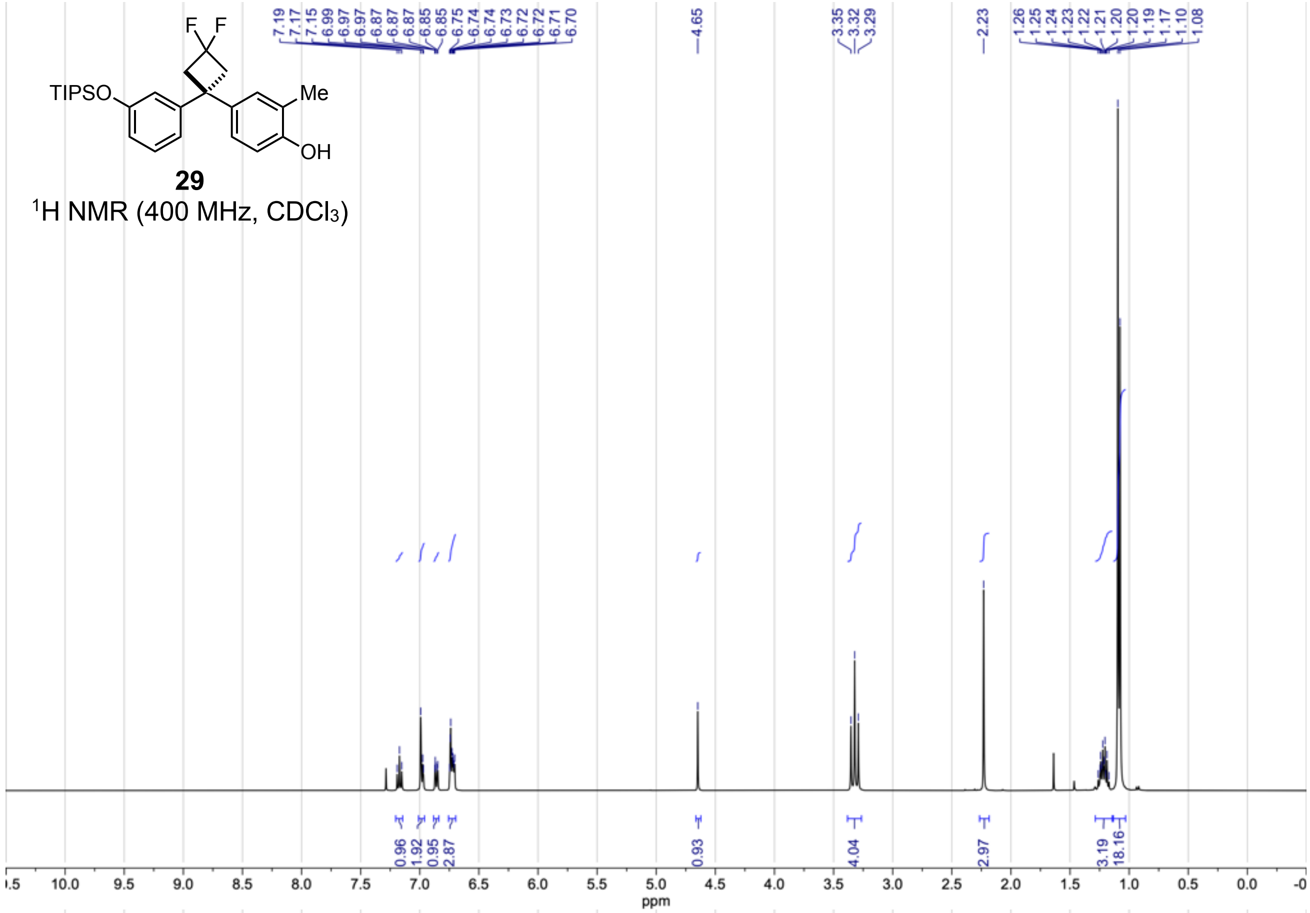
F F
TIPSO
Me
OH
29
^{1}H NMR (400 MHz, CDCl$_3$)
7.19 7.17 7.15 6.99 6.97 6.97 6.87 6.87 6.87 6.85 6.85 6.75 6.74 6.74 6.73 6.72 6.72 6.71 6.70
4.65
3.35 3.32 3.29
2.23
1.26 1.25 1.24 1.23 1.22 1.21 1.20 1.19 1.17 1.10 1.08
0.96 1.92 0.95 2.87 0.93 4.04 2.97 3.19 18.16
ppm

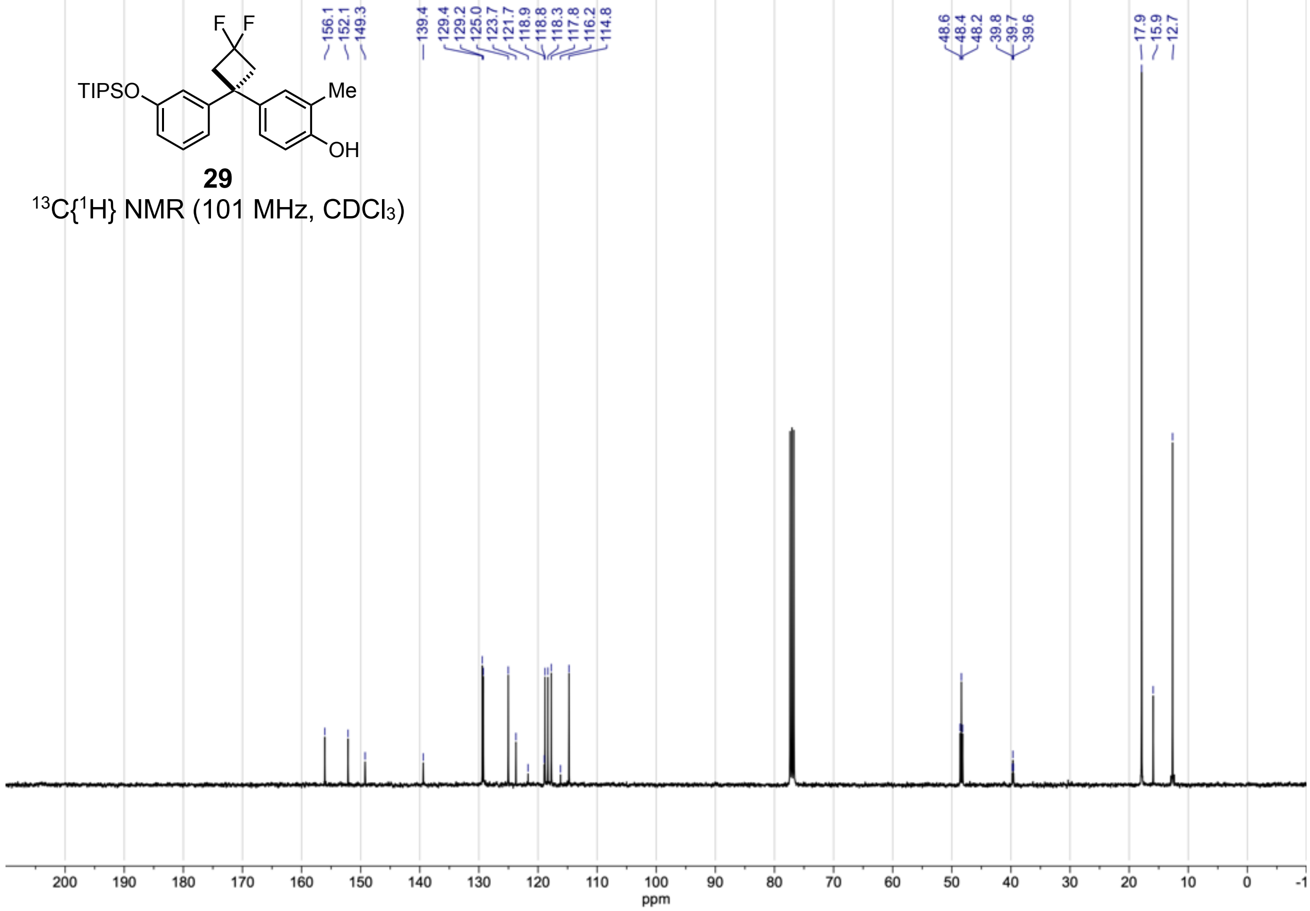
F F
TIPSO
Me
OH
29
^{13}C{^{1}H} NMR (101 MHz, CDCl$_3$)
156.1 152.1 149.3 139.4 129.4 129.2 125.0 123.7 121.7 118.9 118.8 118.3 117.8 116.2 114.8
48.6 48.4 48.2 39.8 39.7 39.6
17.9 15.9 12.7
ppm

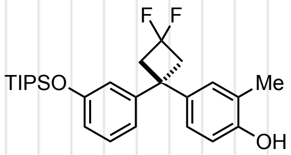

29

^{19}F NMR (377 MHz, $CDCl_3$)

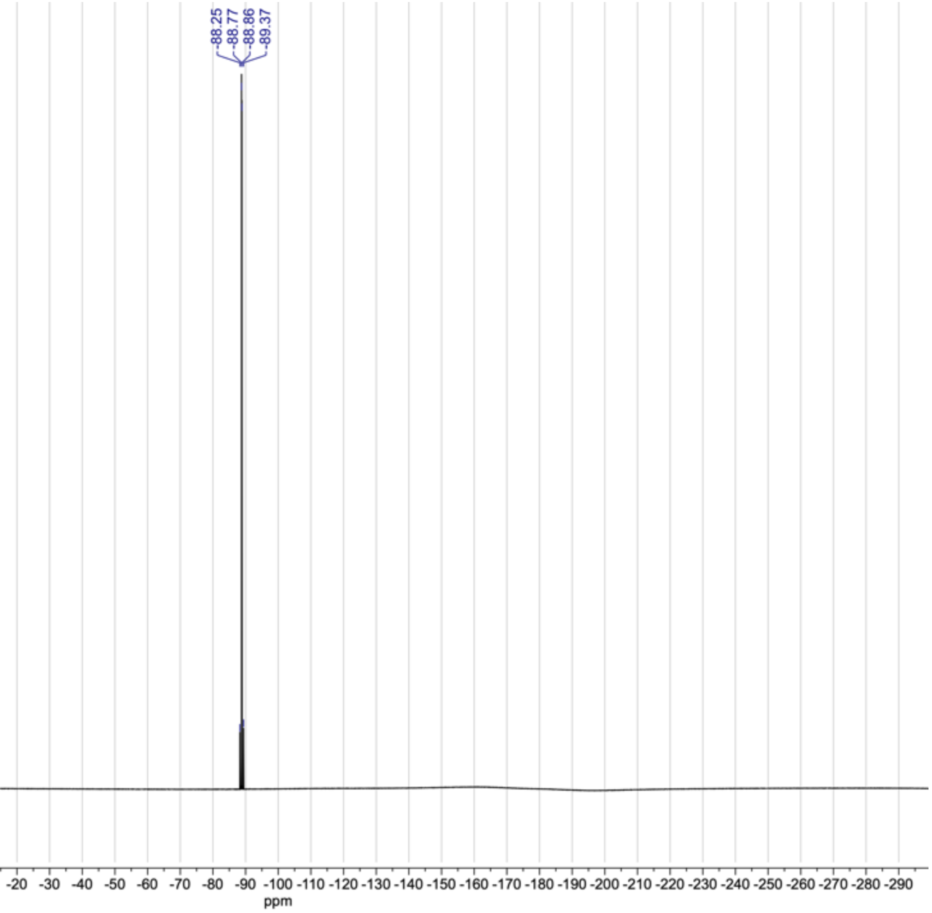

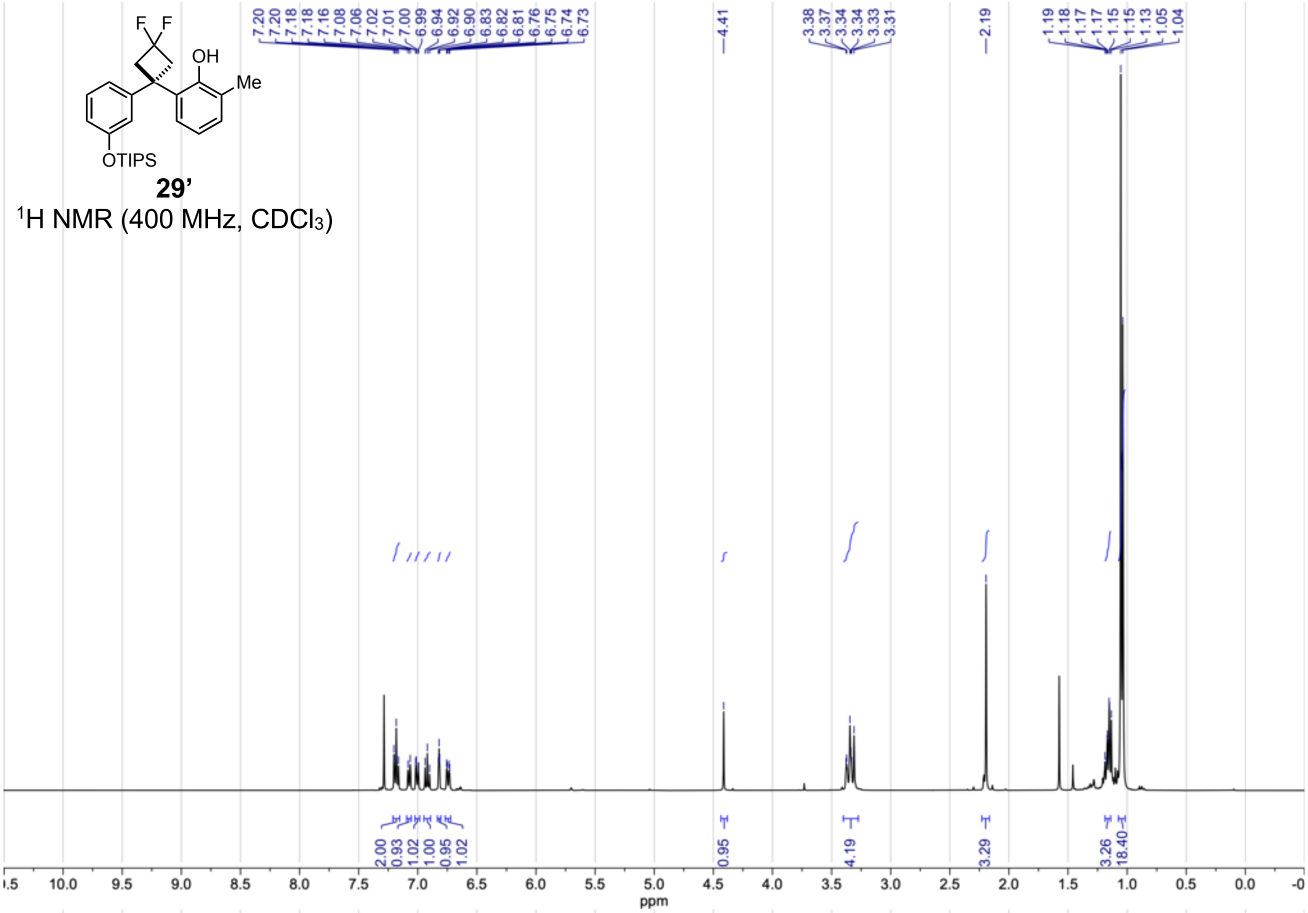

29'

^{1}H NMR (400 MHz, $CDCl_3$)

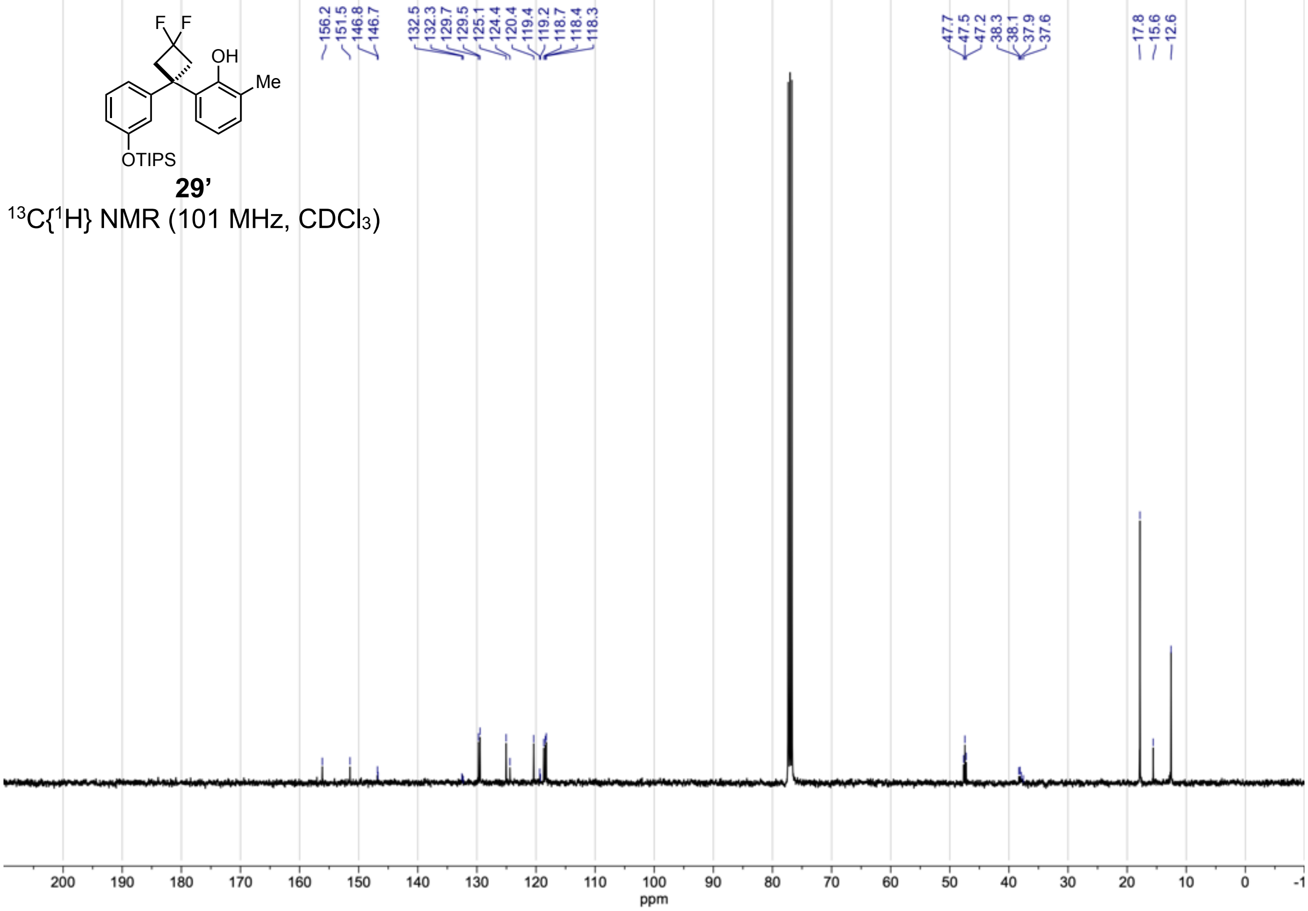

29'

^{13}C{^{1}H} NMR (101 MHz, $CDCl_3$)

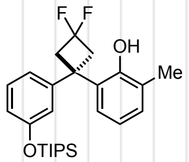

29’

^{19}F NMR (377 MHz, $CDCl_3$)

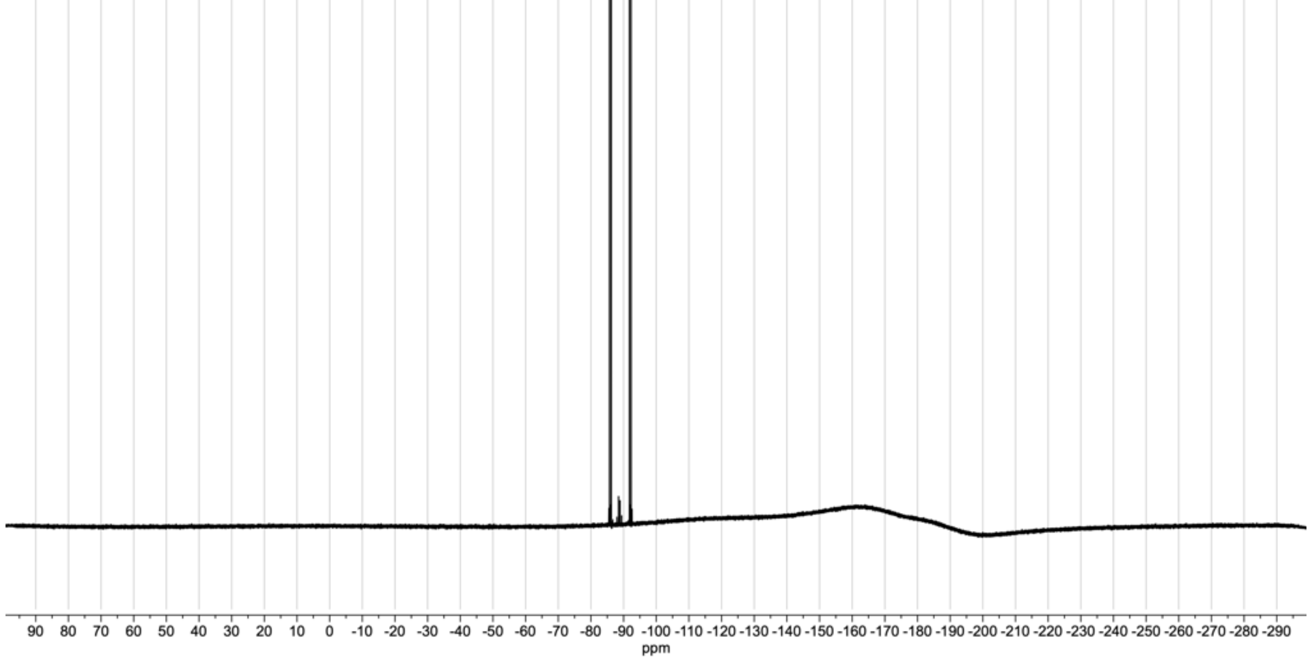

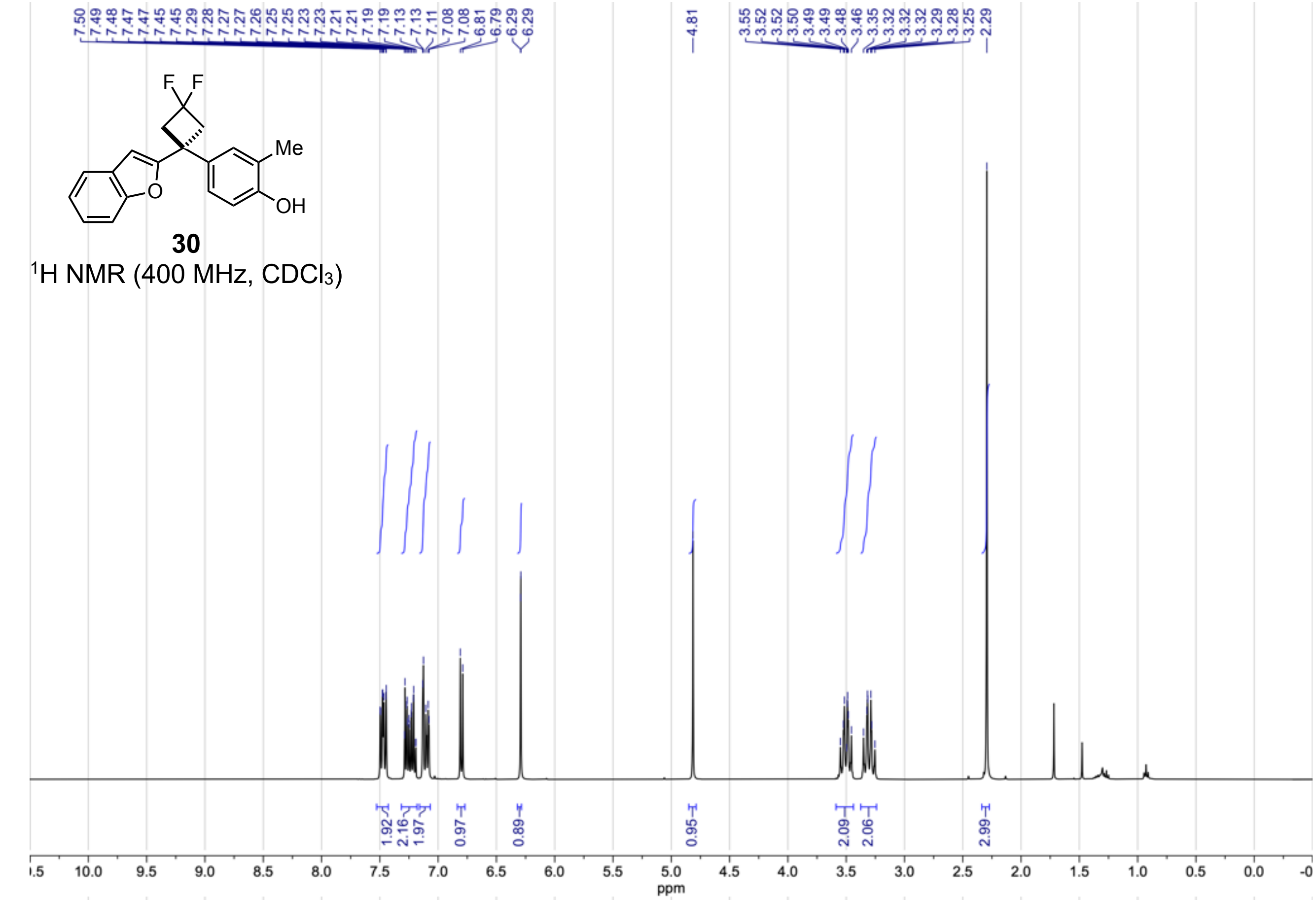
30
1H NMR (400 MHz, CDCl3)
ppm

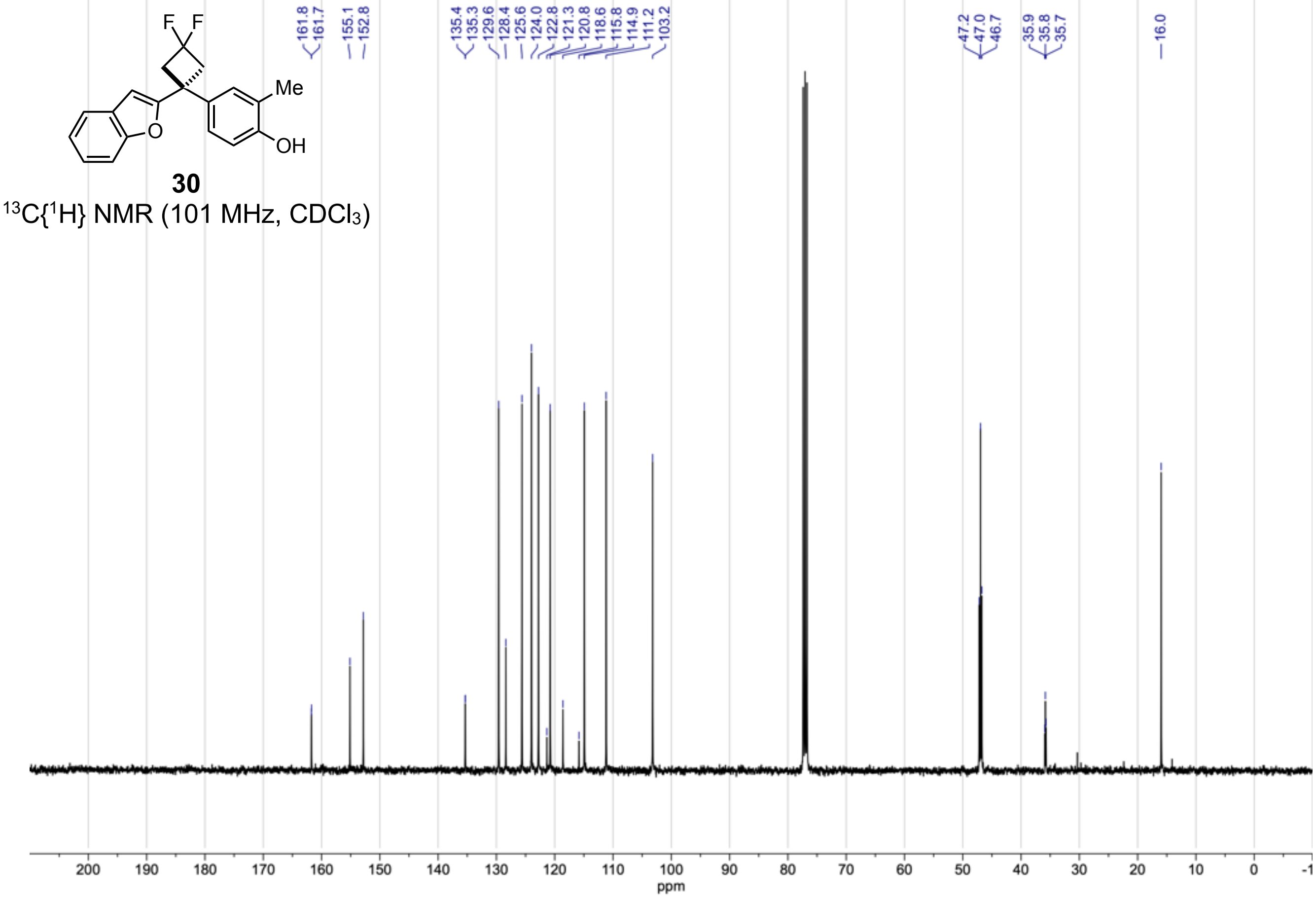
30
13C{1H} NMR (101 MHz, CDCl3)
ppm

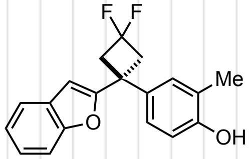

30

^{19}F NMR (377 MHz, $CDCl_3$)

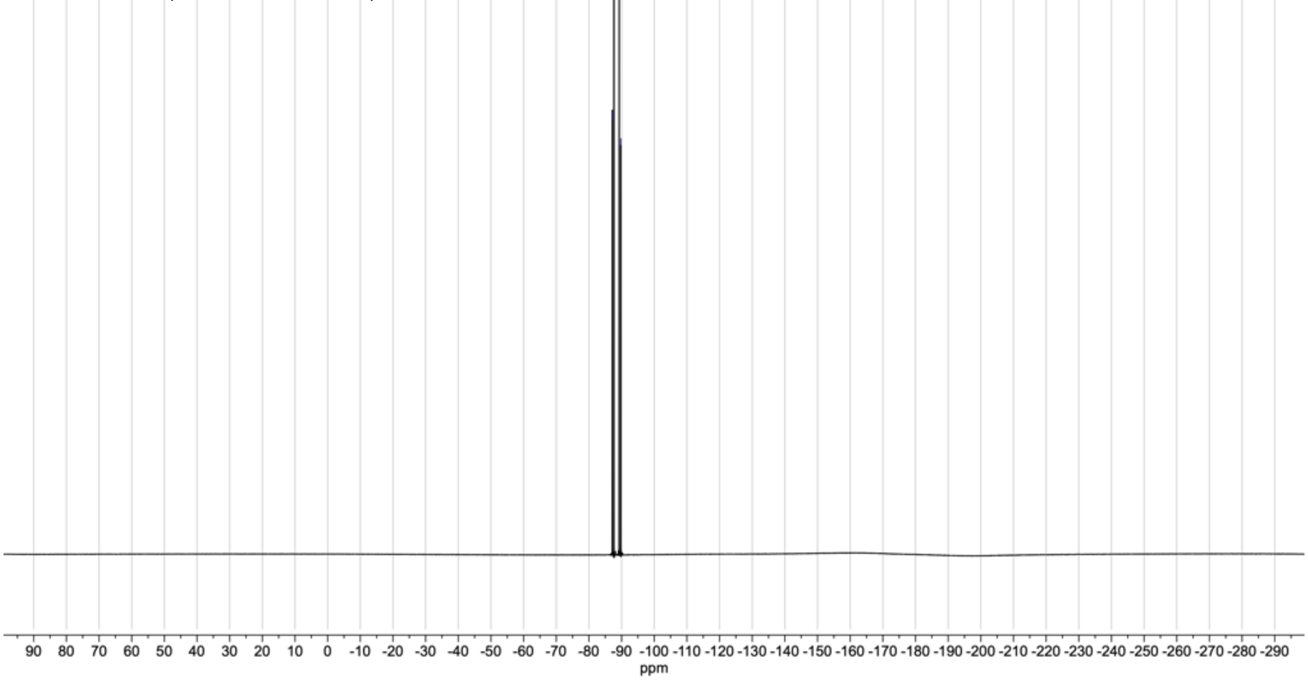

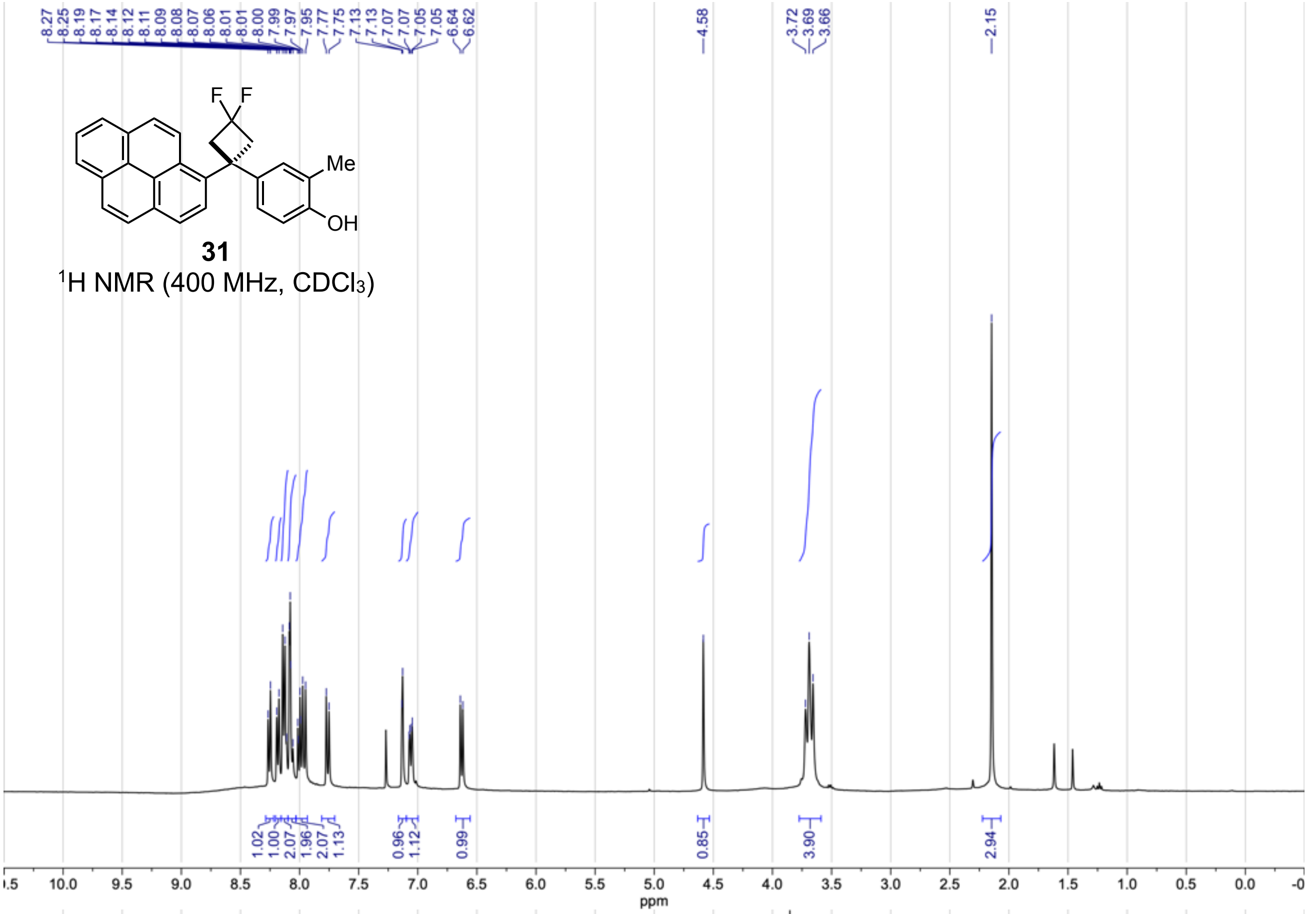
8.27
8.25
8.19
8.17
8.14
8.12
8.11
8.09
8.08
8.07
8.06
8.01
8.01
8.00
7.99
7.97
7.95
7.77
7.75
7.13
7.13
7.07
7.07
7.05
7.05
6.64
6.62
4.58
3.72
3.69
3.66
2.15
F F
Me
OH
31
^{1}H NMR (400 MHz, CDCl$_3$)
1.02
1.00
2.07
1.96
2.07
1.13
0.96
1.12
0.99
0.85
3.90
2.94
ppm

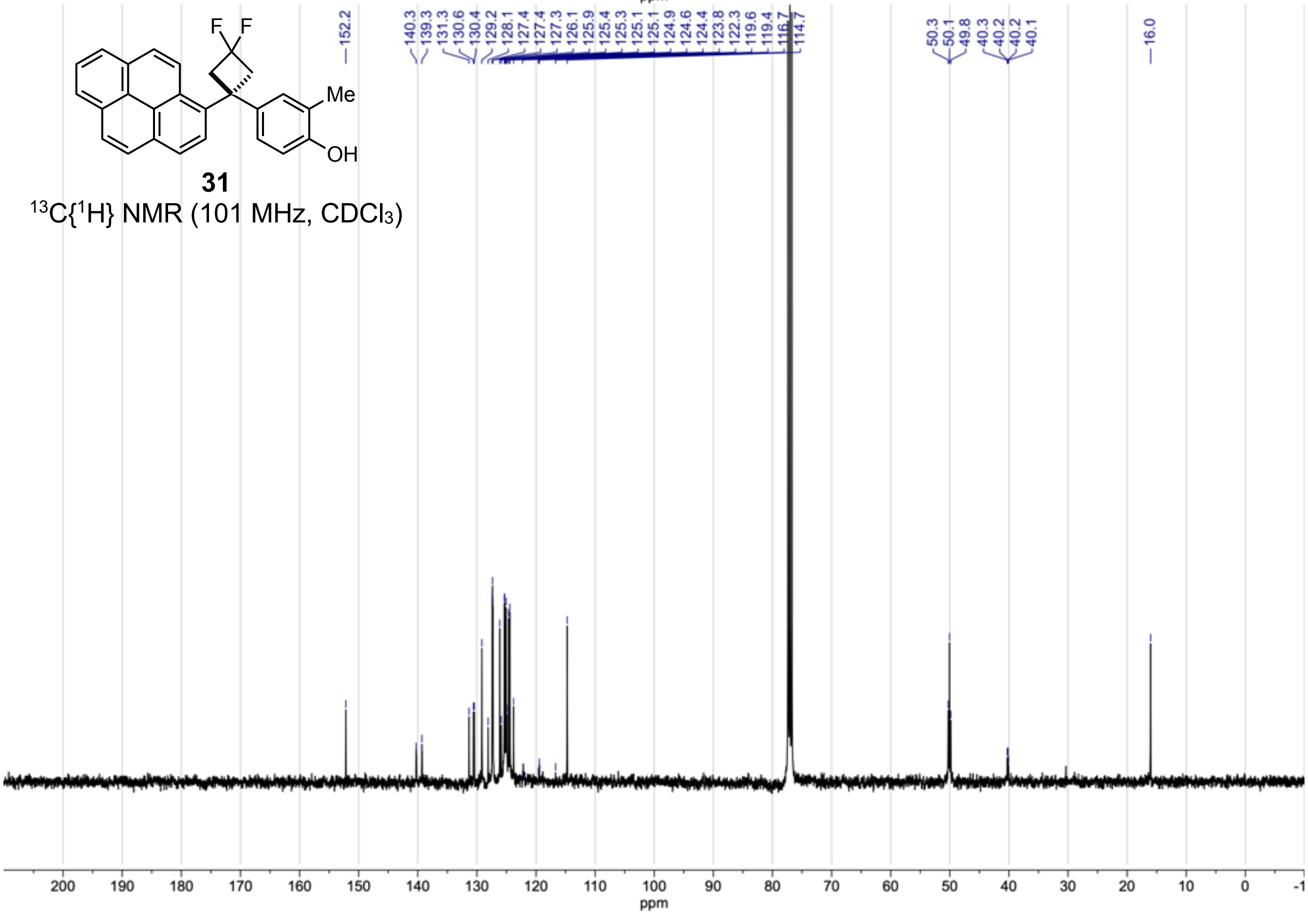
152.2
140.3
139.3
131.3
130.6
130.4
129.2
128.1
127.4
127.3
126.1
125.9
125.4
125.3
125.1
125.1
124.9
124.6
124.4
123.8
122.3
119.6
119.4
116.7
114.7
50.3
50.1
49.8
40.3
40.2
40.1
16.0
F F
Me
OH
31
^{13}C{^{1}H} NMR (101 MHz, CDCl$_3$)
ppm

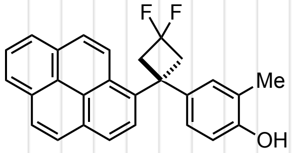

31

^{19}F NMR (377 MHz, $CDCl_3$)

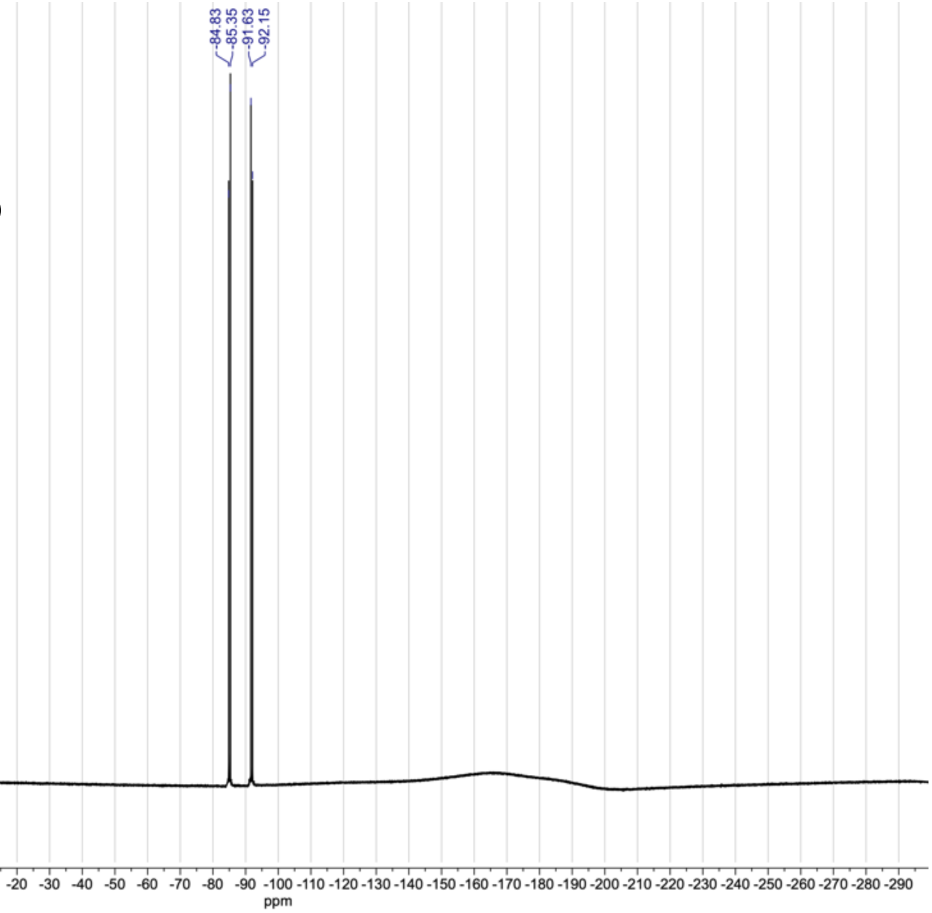

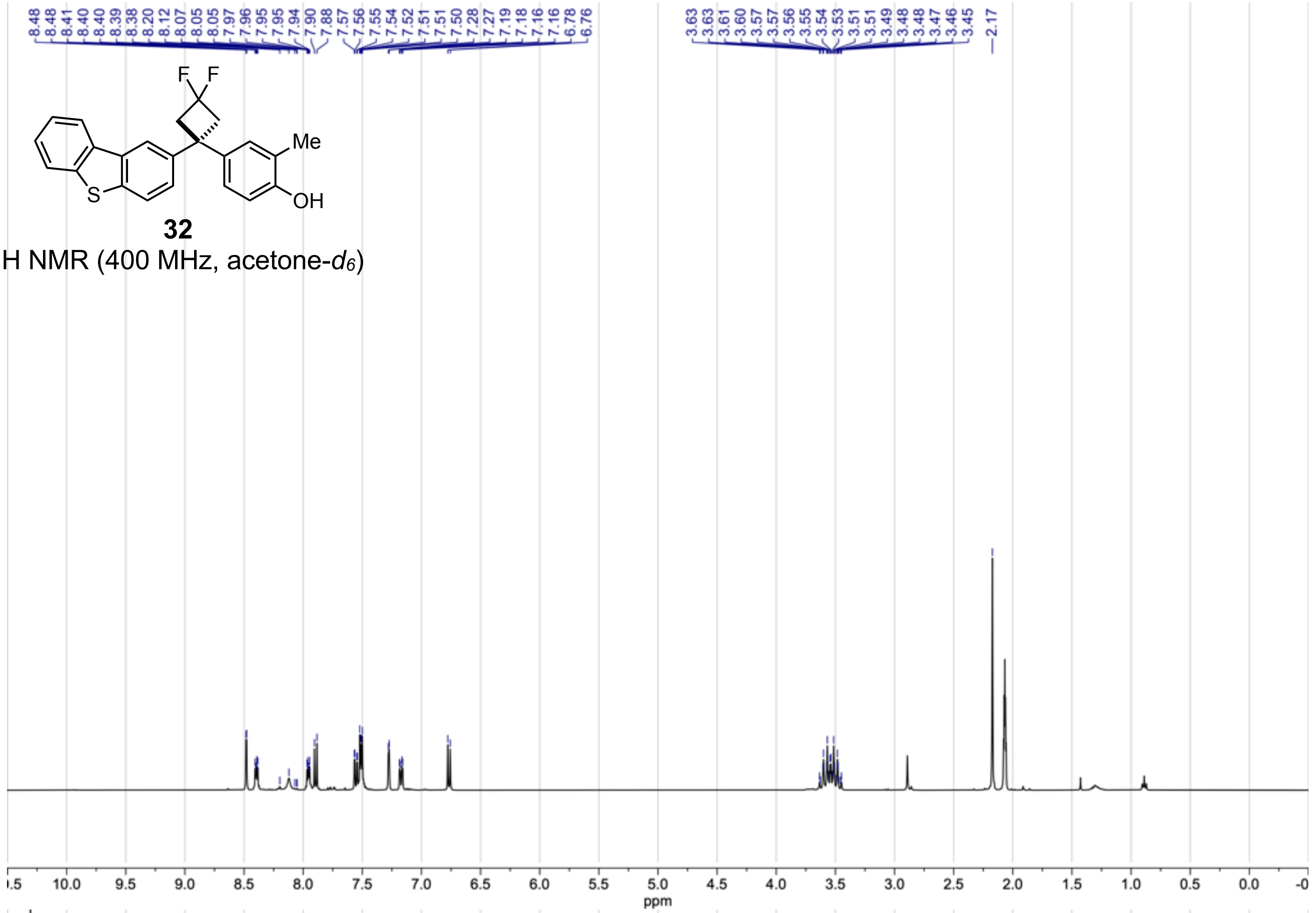
8.48
8.48
8.41
8.40
8.40
8.39
8.38
8.20
8.12
8.07
8.05
8.05
7.97
7.96
7.95
7.95
7.94
7.90
7.88
7.57
7.56
7.55
7.54
7.52
7.51
7.51
7.50
7.28
7.27
7.19
7.18
7.16
7.16
6.78
6.76
3.63
3.63
3.61
3.60
3.57
3.57
3.56
3.55
3.54
3.53
3.51
3.51
3.49
3.48
3.48
3.47
3.46
3.45
2.17
F F
Me
S
OH
32
^{1}H NMR (400 MHz, acetone-d_6)
ppm

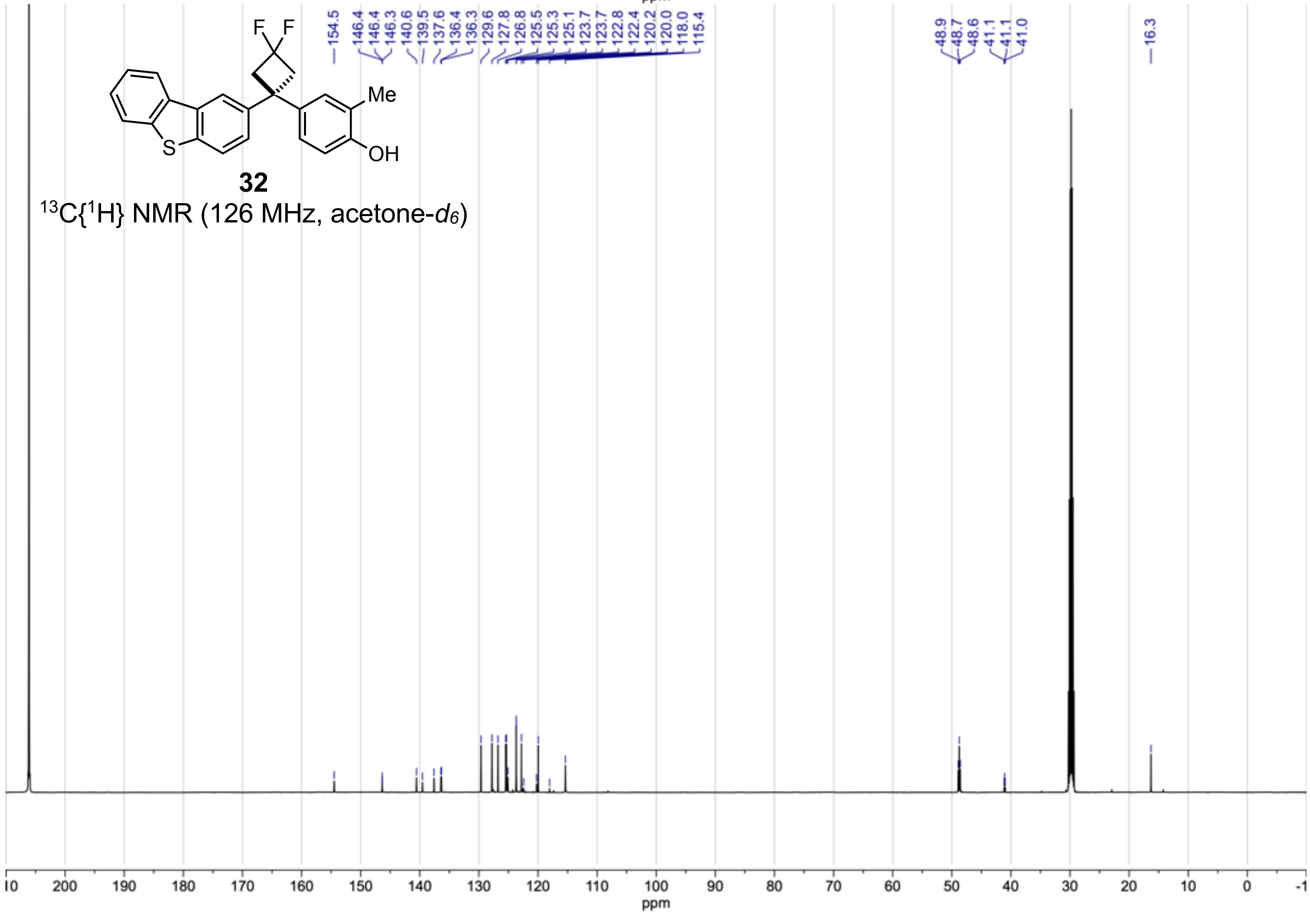
154.5
146.4
146.4
146.3
140.6
139.5
137.6
136.4
136.3
129.6
127.8
126.8
125.5
125.3
125.1
123.7
123.7
122.8
122.4
120.2
120.0
118.0
115.4
48.9
48.7
48.6
41.1
41.1
41.0
16.3
F F
Me
S
OH
32
^{13}C{^{1}H} NMR (126 MHz, acetone-d_6)
ppm

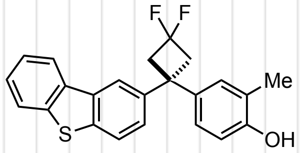

32

^{19}F NMR (377 MHz, acetone-*d*$_6$)

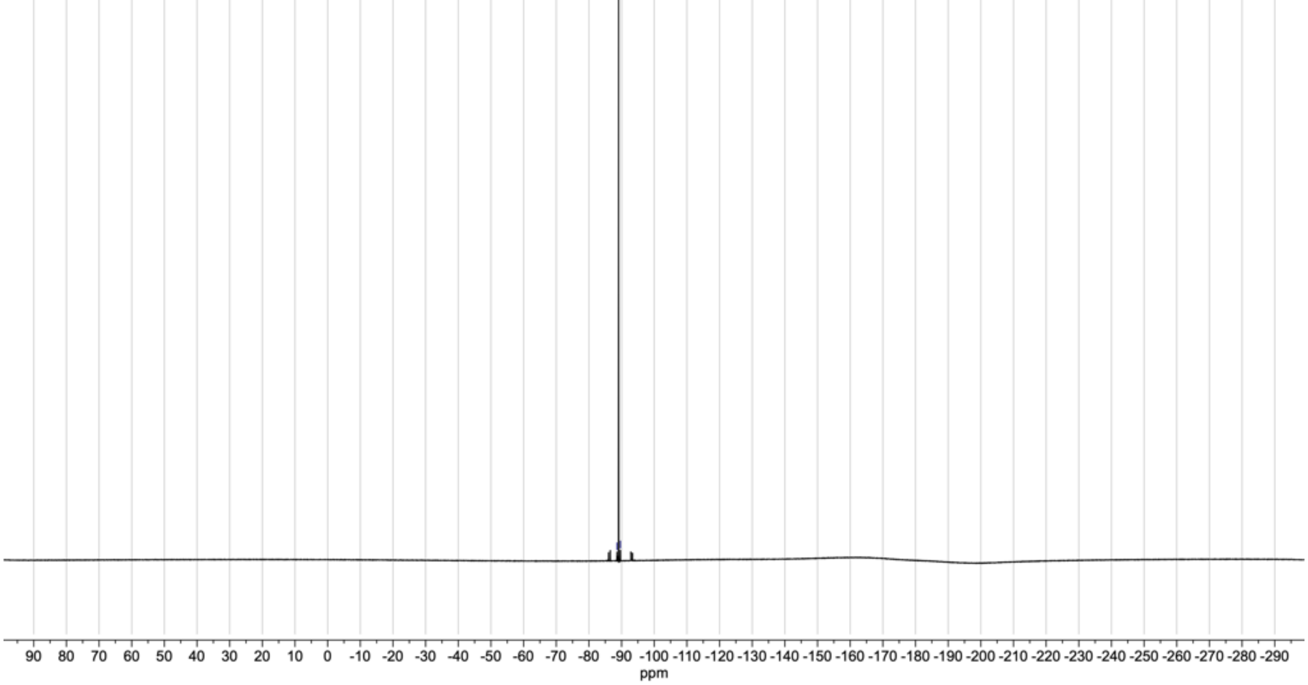

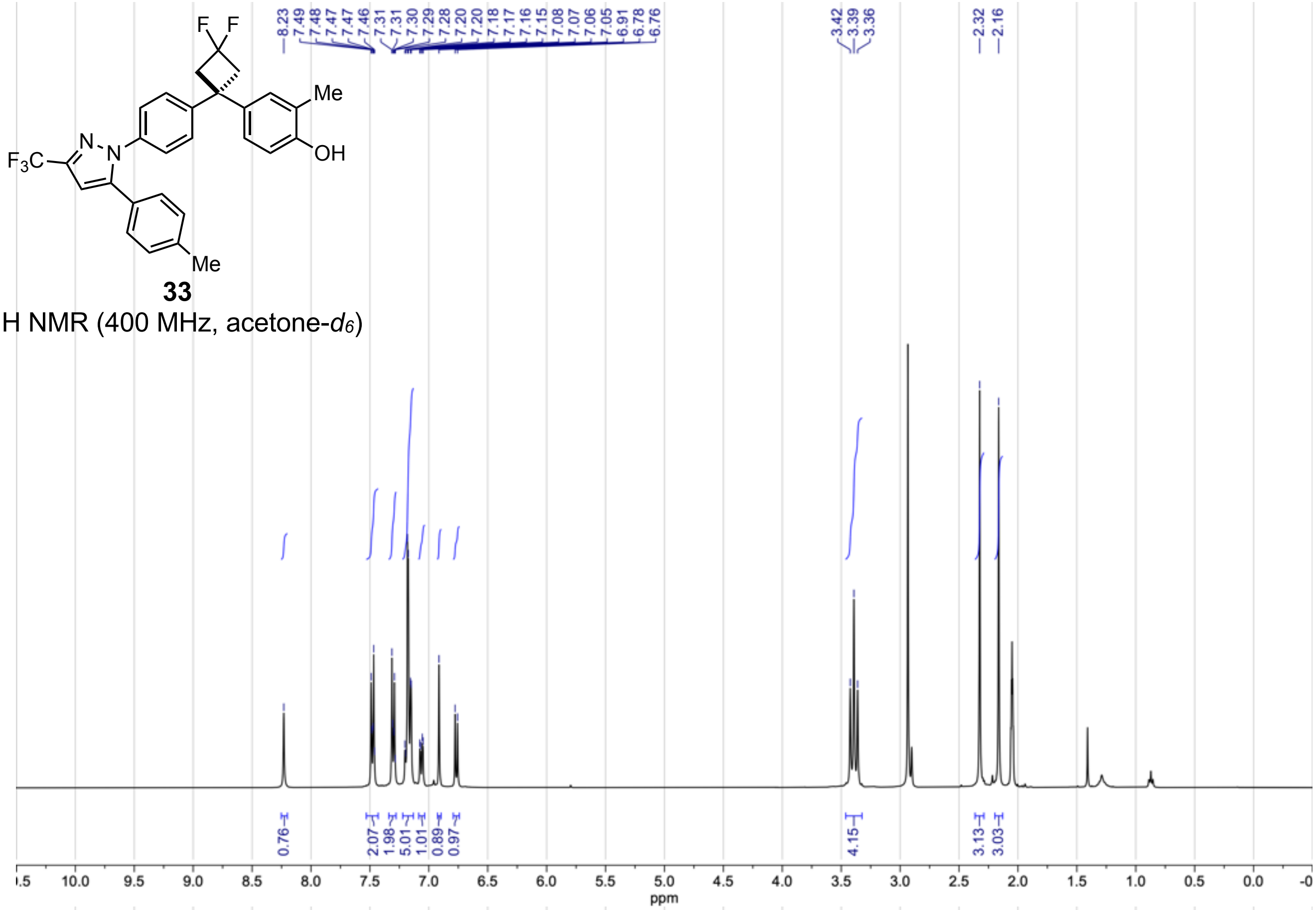

33

^{1}H NMR (400 MHz, acetone-d_6)

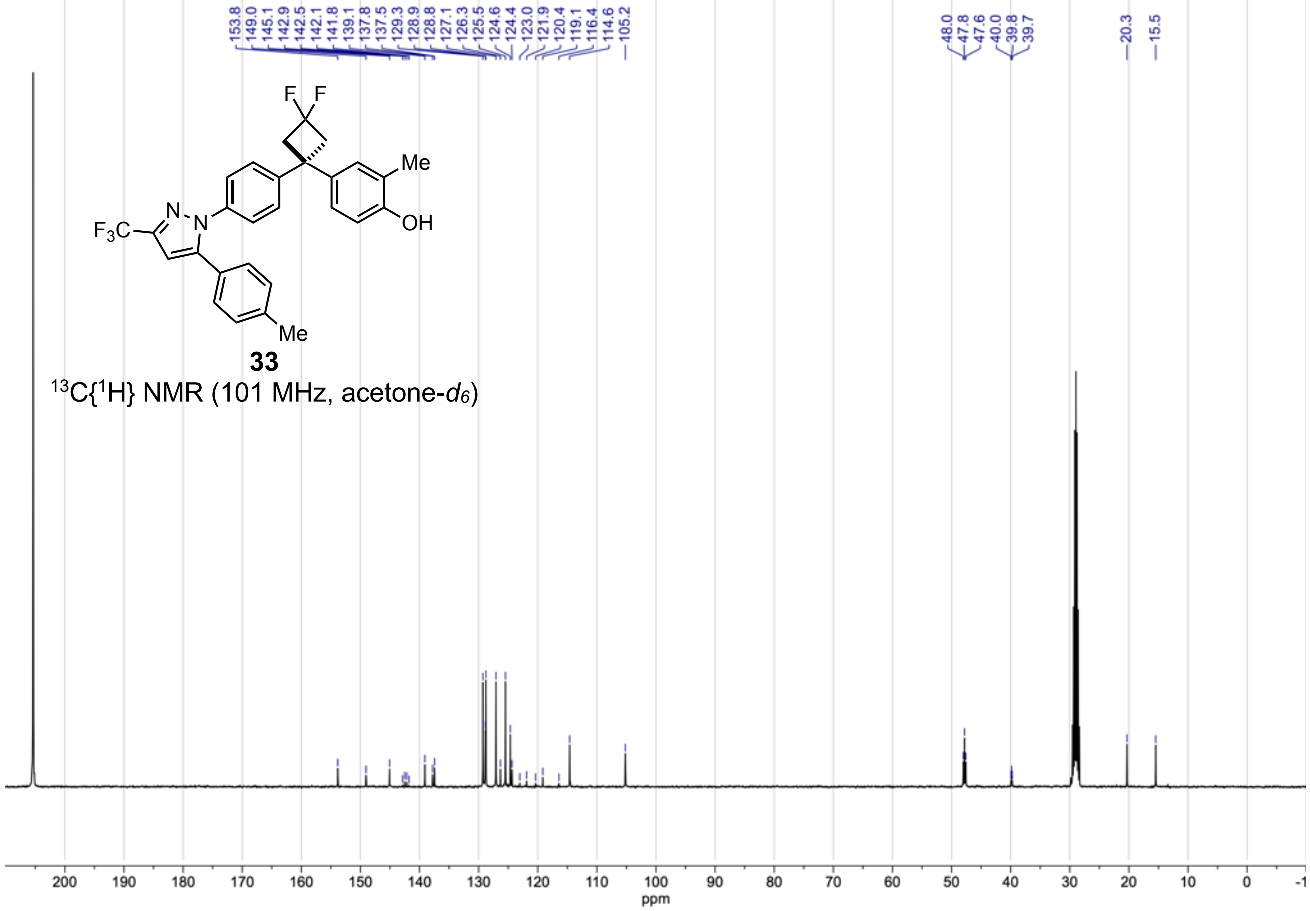

33

^{13}C{^{1}H} NMR (101 MHz, acetone-d_6)

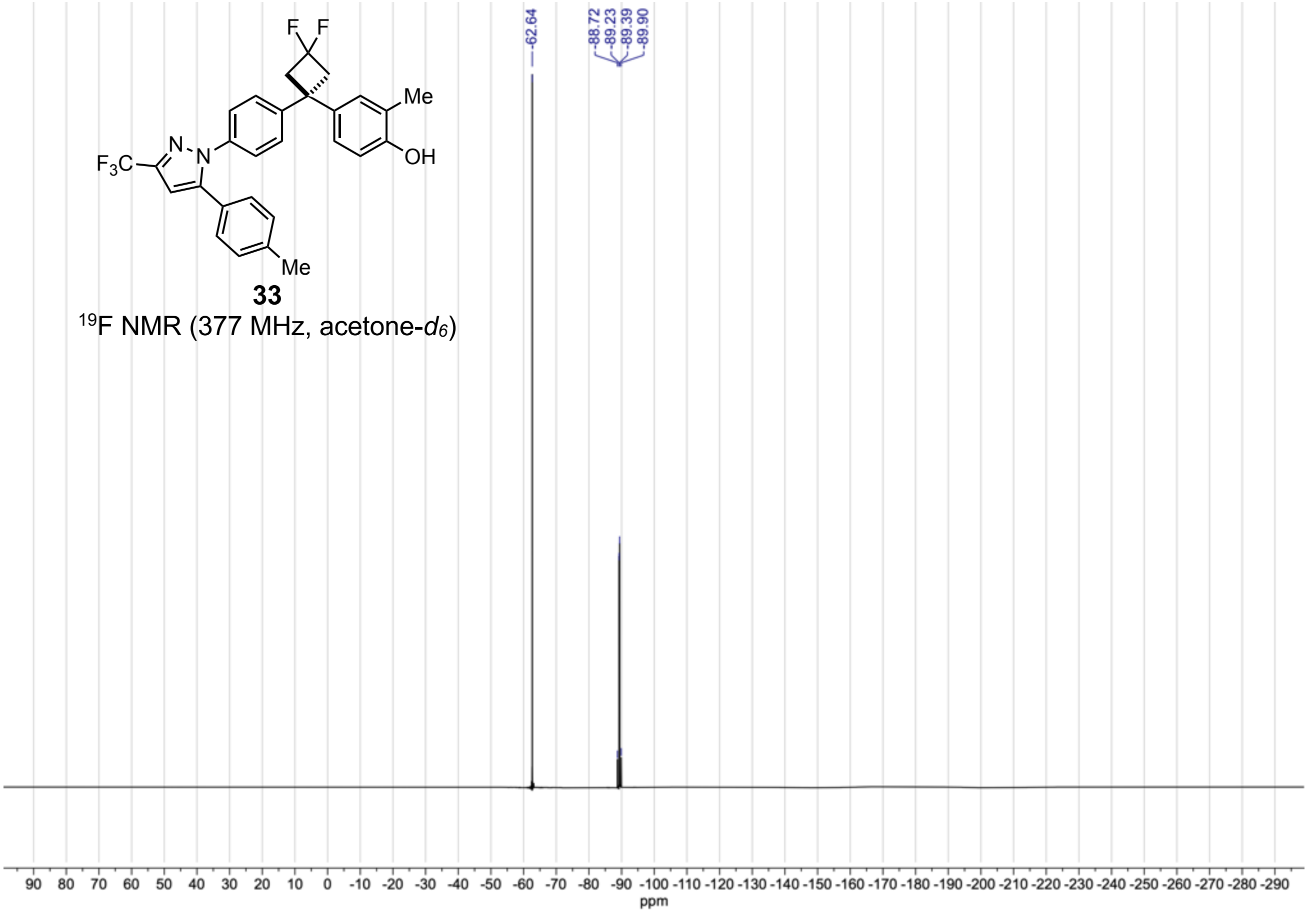

33

^{19}F NMR (377 MHz, acetone-d_6)

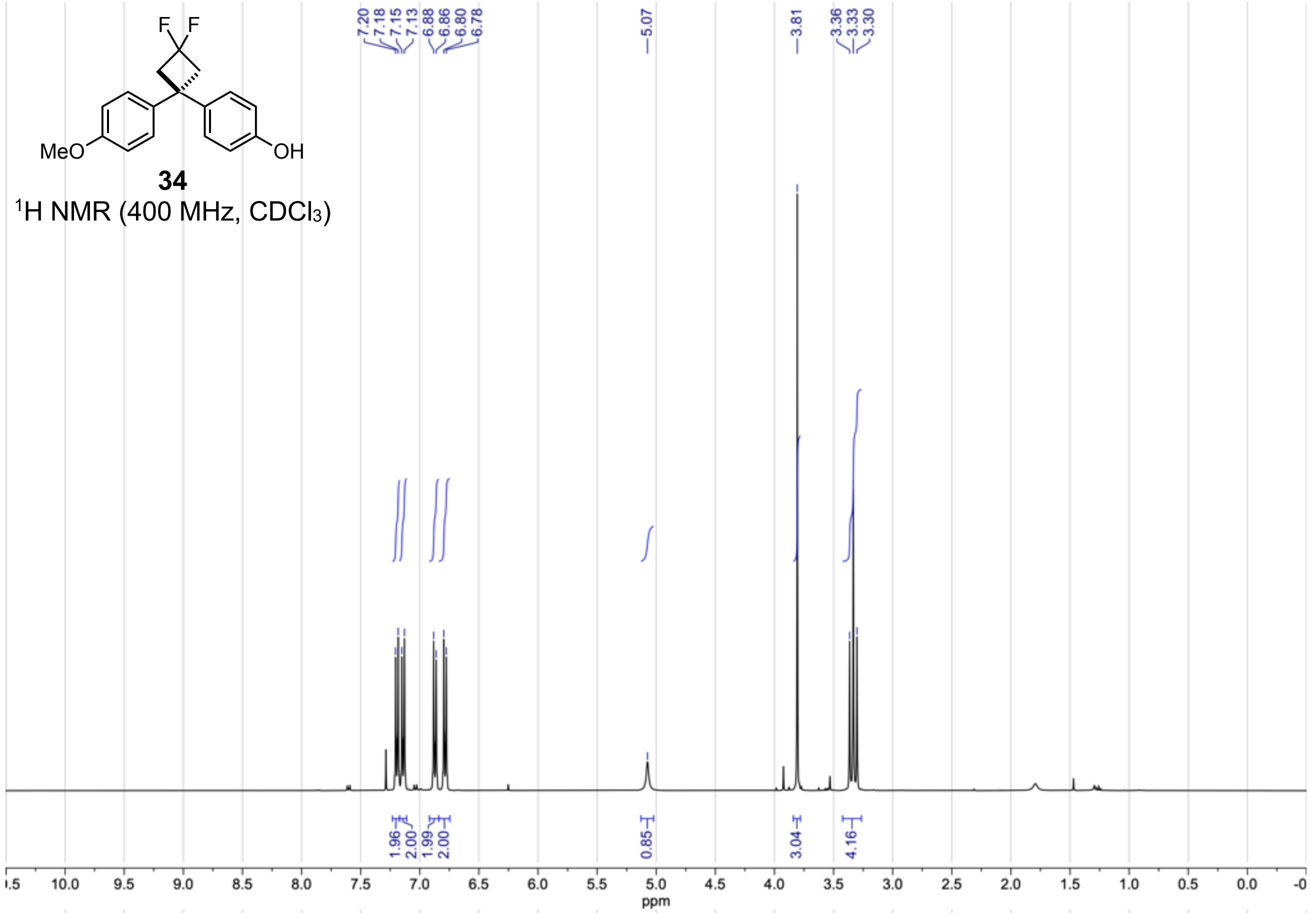

34

^{1}H NMR (400 MHz, $CDCl_3$)

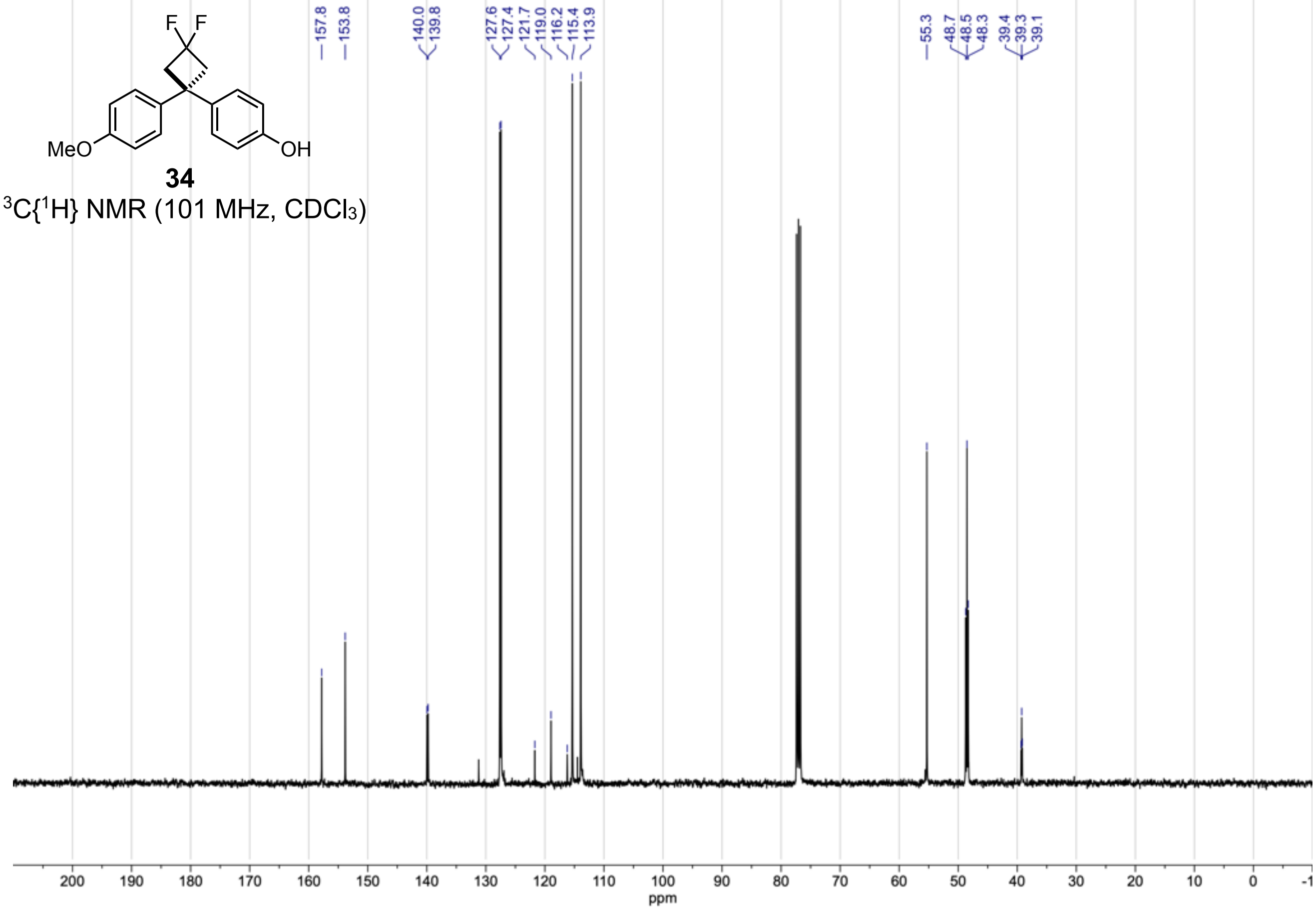

34

^{13}C{^{1}H} NMR (101 MHz, $CDCl_3$)

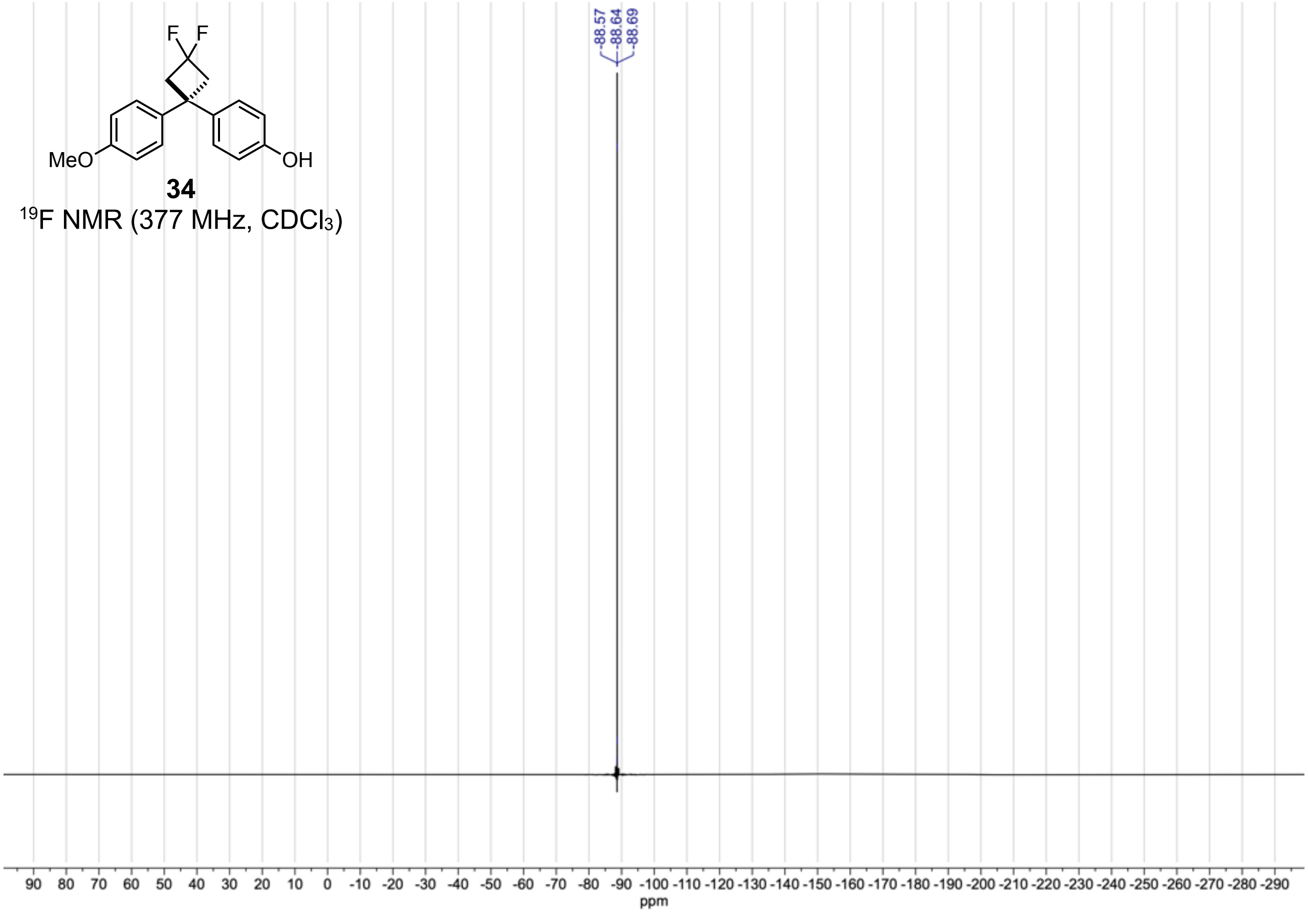

34

^{19}F NMR (377 MHz, $CDCl_3$)

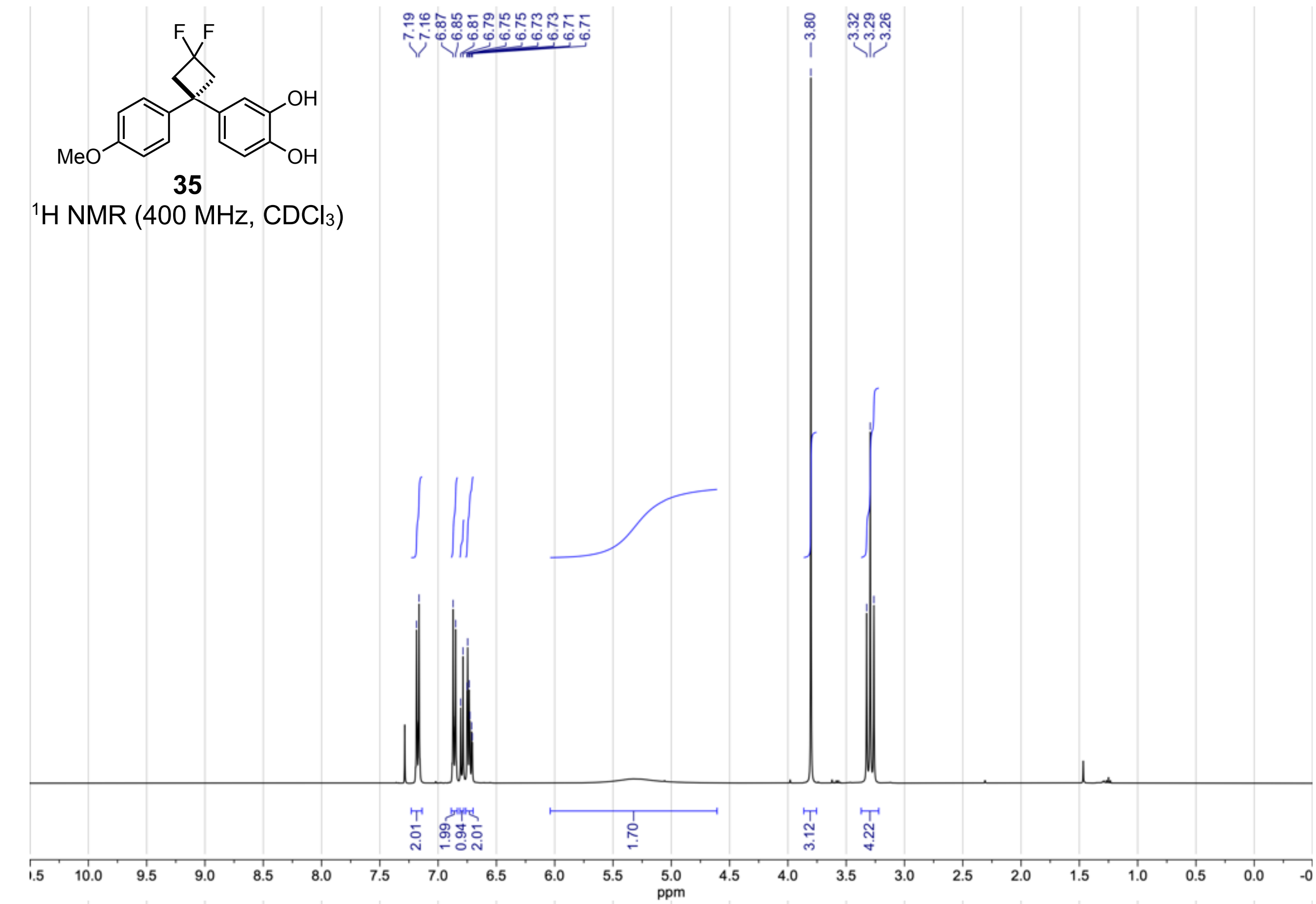
F F
OH
MeO
OH
35
1H NMR (400 MHz, CDCl3)
7.19 7.16 6.87 6.85 6.81 6.79 6.75 6.75 6.73 6.73 6.71 6.71
3.80
3.32 3.29 3.26
2.01 1.99 0.94 2.01 1.70 3.12 4.22
ppm

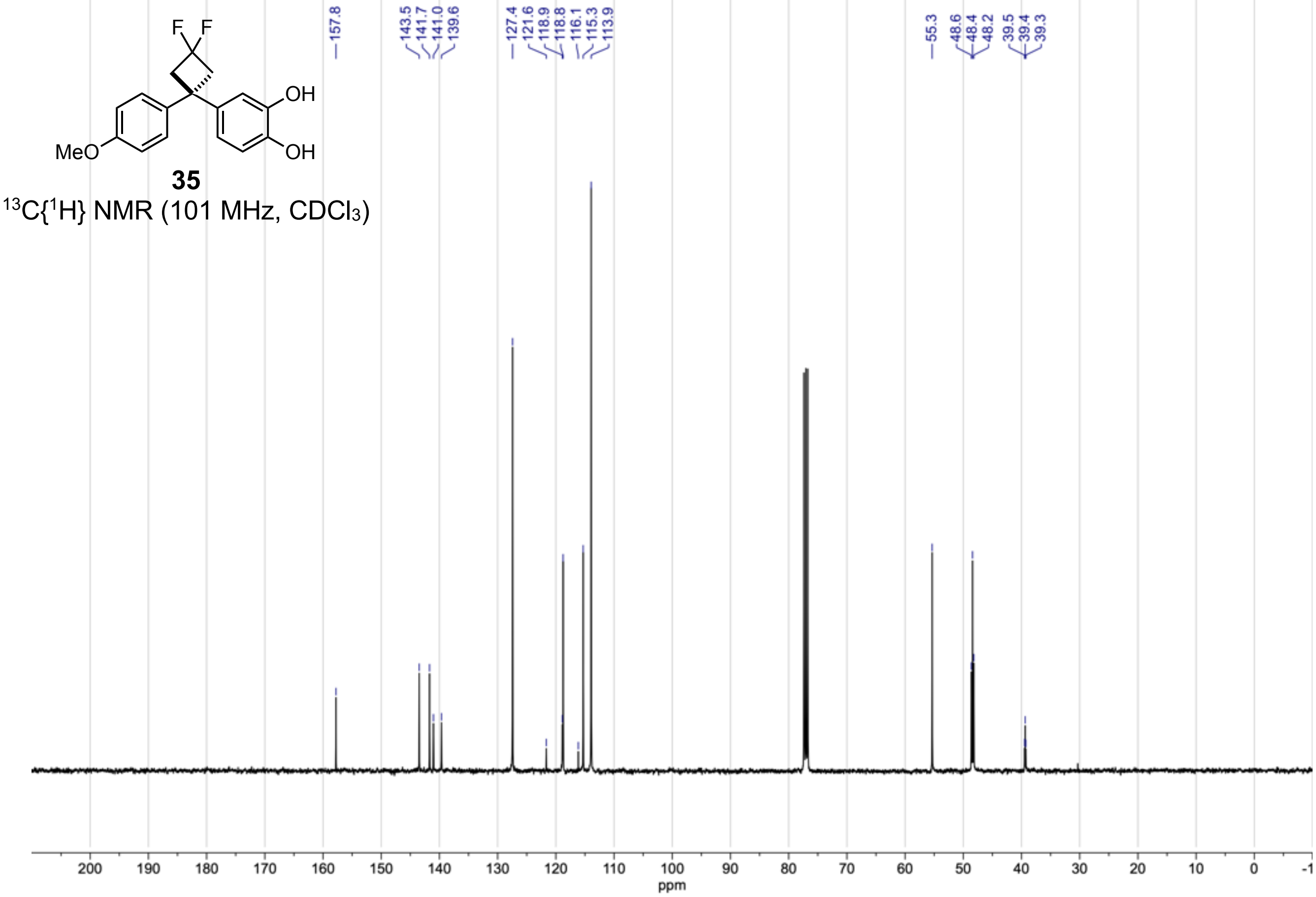
F F
OH
MeO
OH
35
13C{1H} NMR (101 MHz, CDCl3)
157.8
143.5 141.7 141.0 139.6
127.4 121.6 118.9 118.8 116.1 115.3 113.9
55.3 48.6 48.4 48.2 39.5 39.4 39.3
ppm

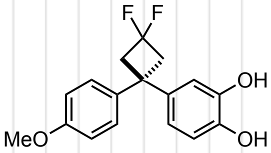

35

^{19}F NMR (377 MHz, $CDCl_3$)

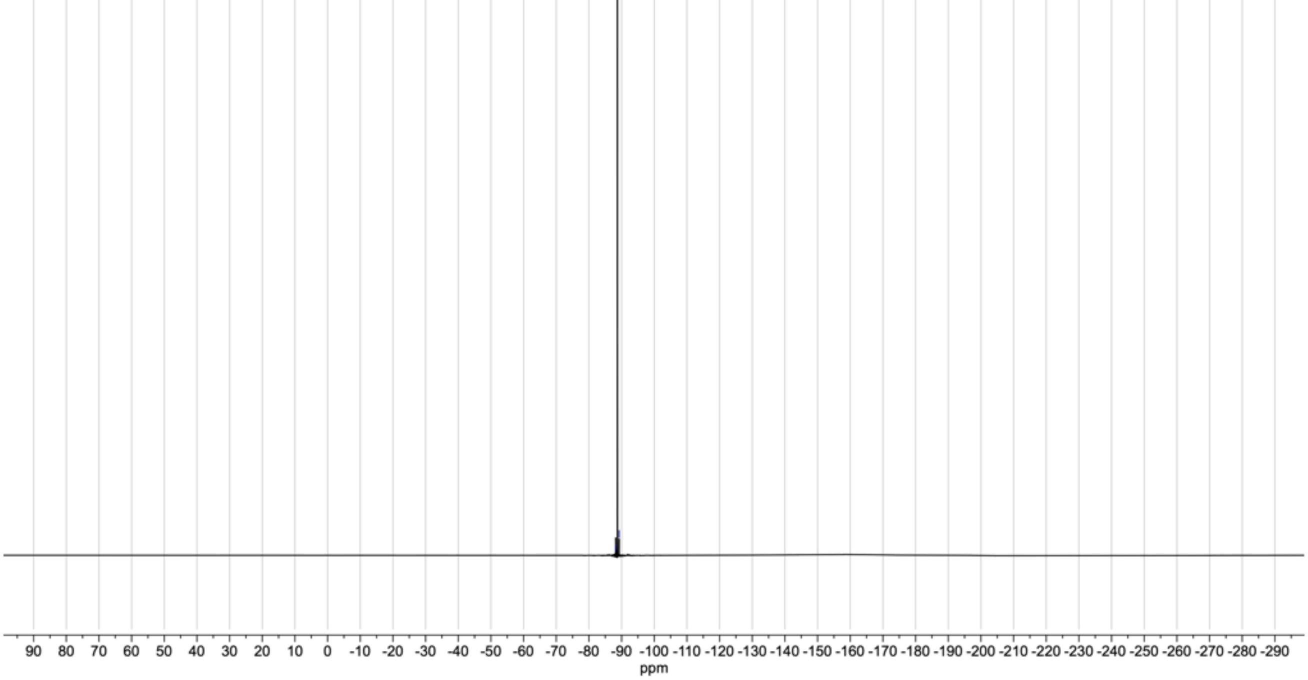

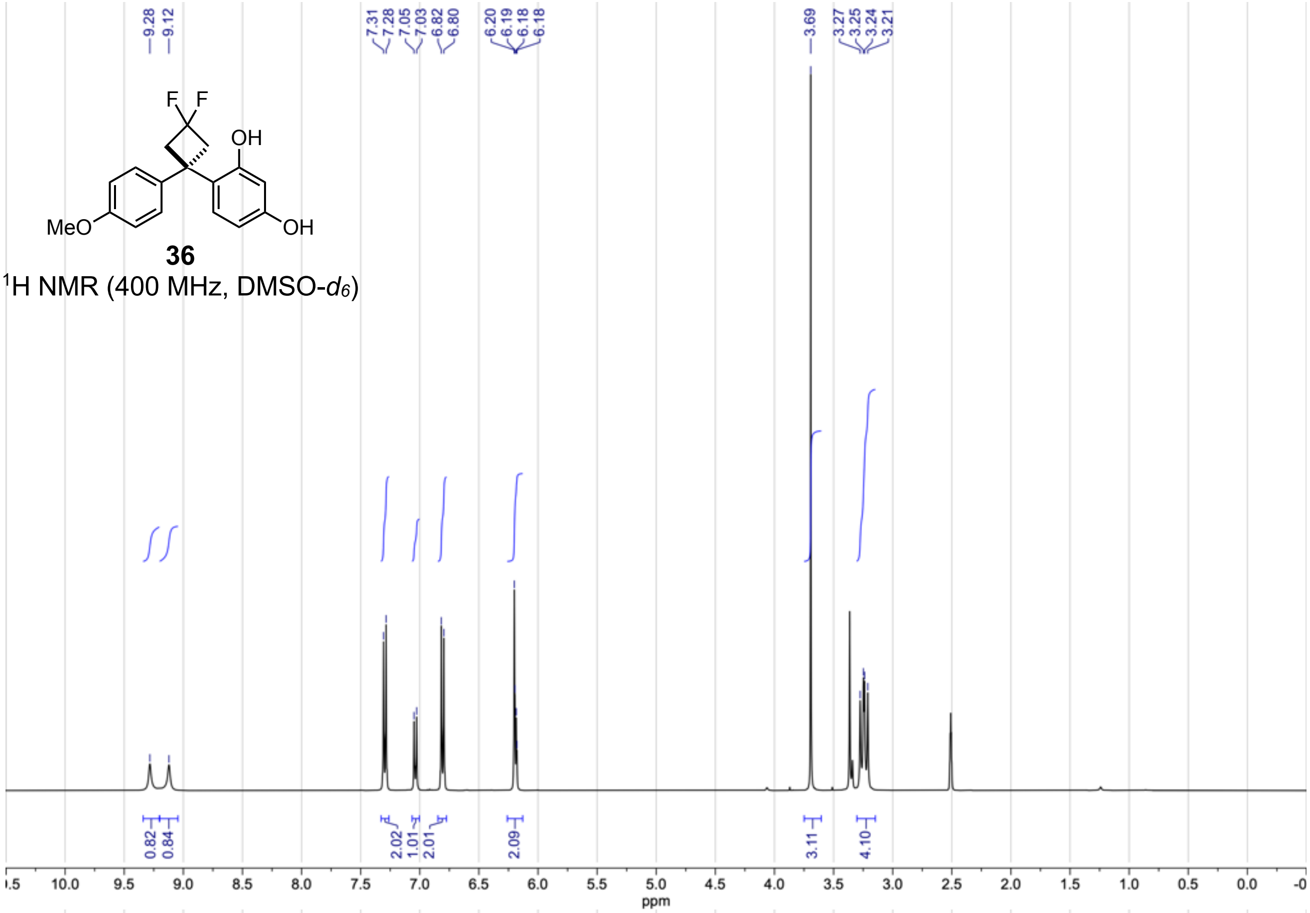

36

^{1}H NMR (400 MHz, DMSO-d_6)

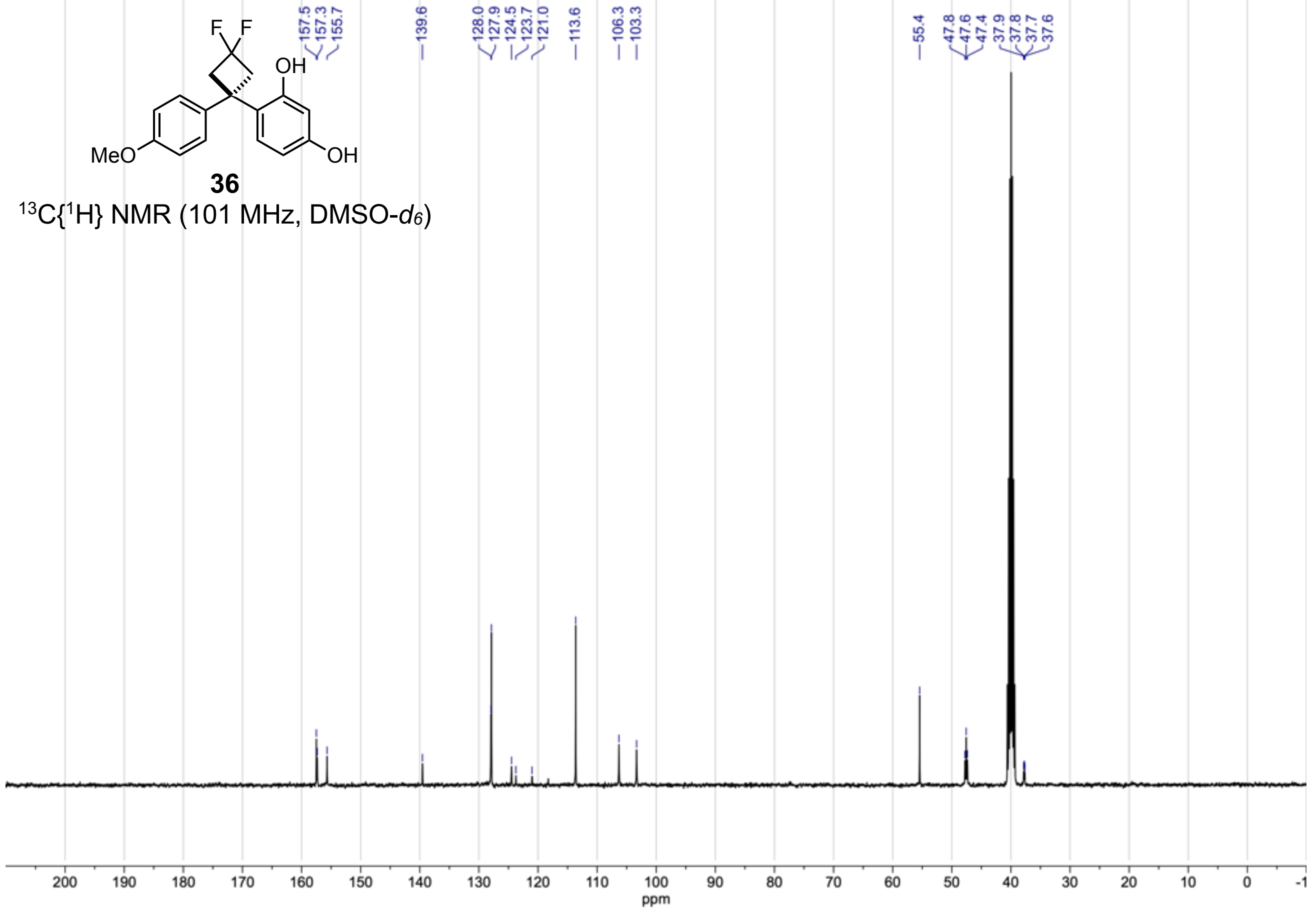

36

^{13}C{^{1}H} NMR (101 MHz, DMSO-d_6)

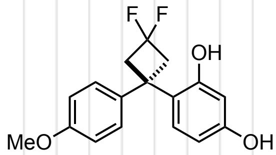

36

^{19}F NMR (377 MHz, DMSO-d_6)

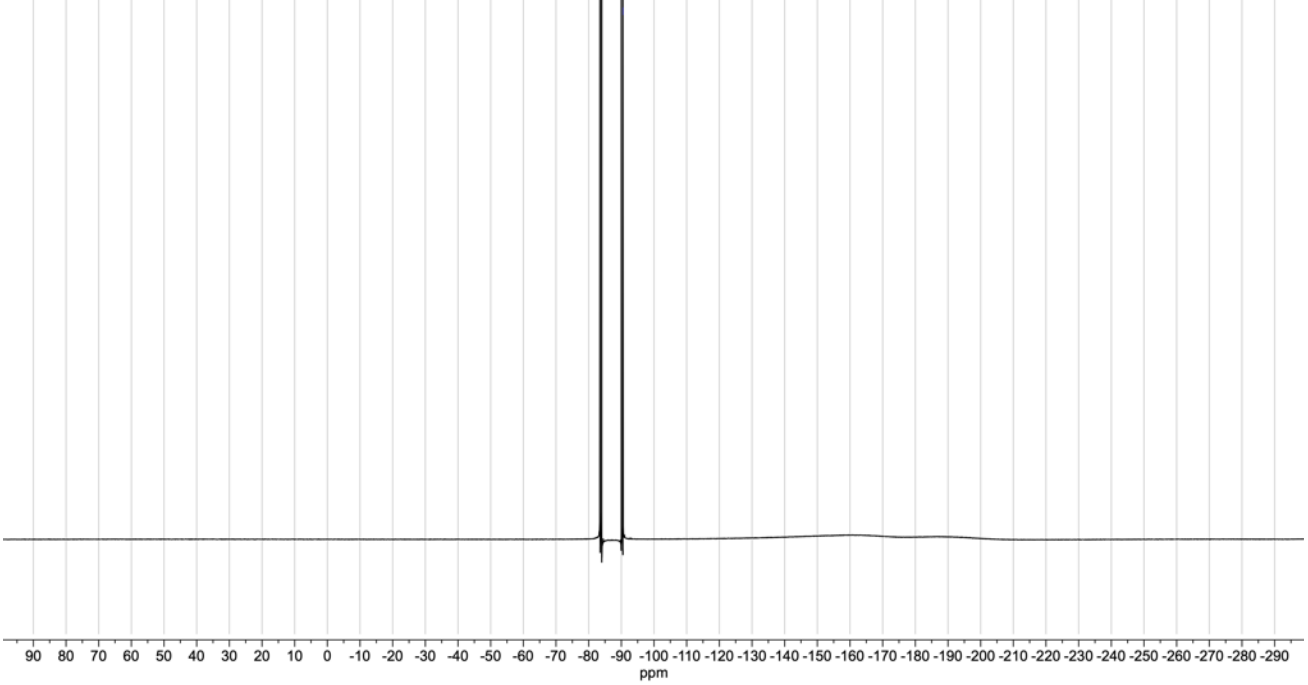

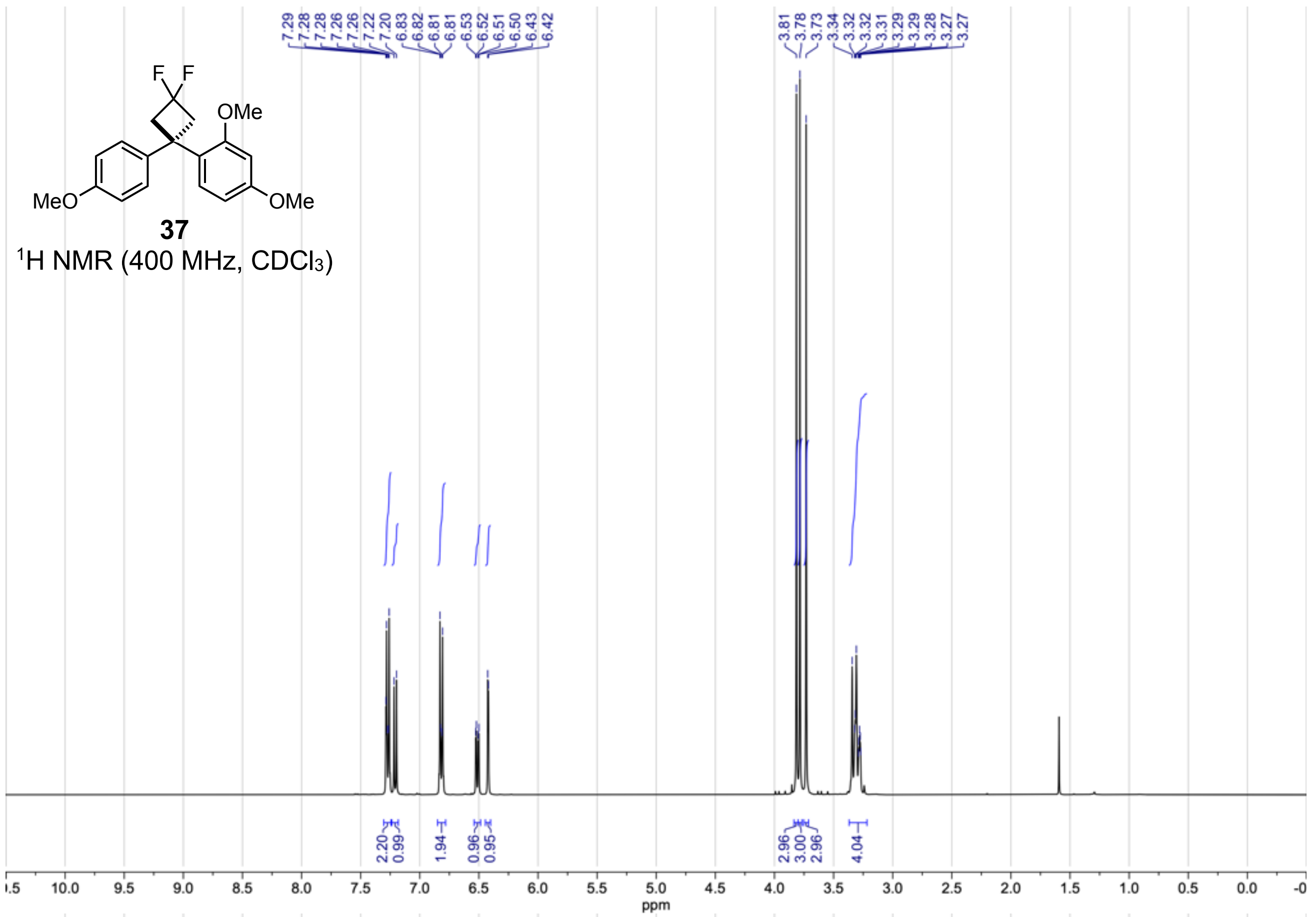
F F
OMe
MeO
OMe
37
1H NMR (400 MHz, CDCl3)
7.29 7.28 7.28 7.26 7.26 7.22 7.20 6.83 6.82 6.81 6.81 6.53 6.52 6.51 6.50 6.43 6.42
3.81 3.78 3.73 3.34 3.32 3.32 3.31 3.29 3.29 3.28 3.27 3.27
2.20 0.99 1.94 0.96 0.95 2.96 3.00 2.96 4.04
ppm

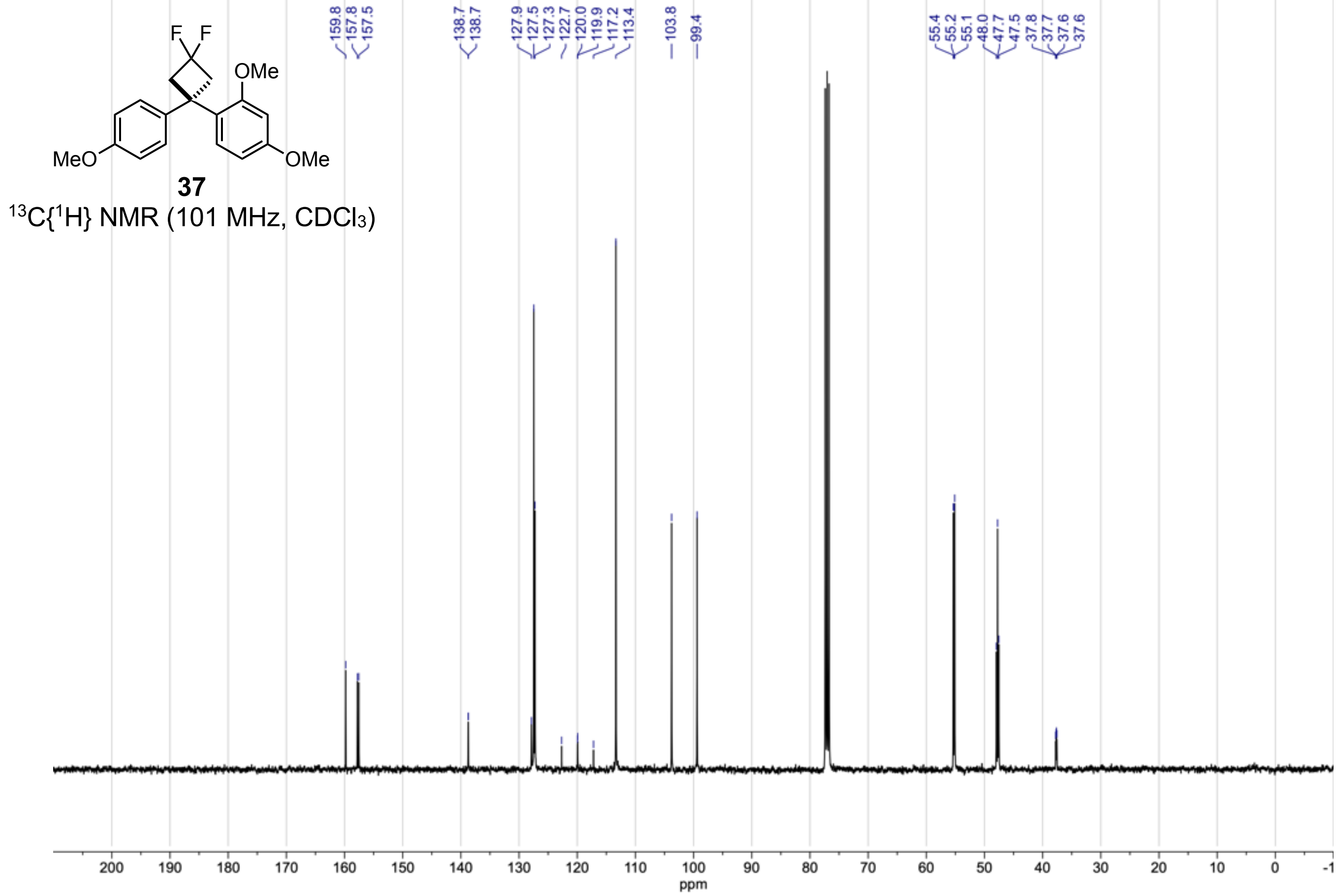
F F
OMe
MeO
OMe
37
13C{1H} NMR (101 MHz, CDCl3)
159.8 157.8 157.5 138.7 138.7 127.9 127.5 127.3 122.7 120.0 119.9 117.2 113.4 103.8 99.4
55.4 55.2 55.1 48.0 47.7 47.5 37.8 37.7 37.6 37.6
ppm

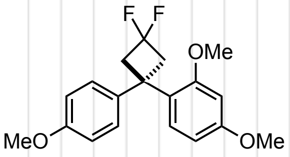

37

^{19}F NMR (377 MHz, $CDCl_3$)

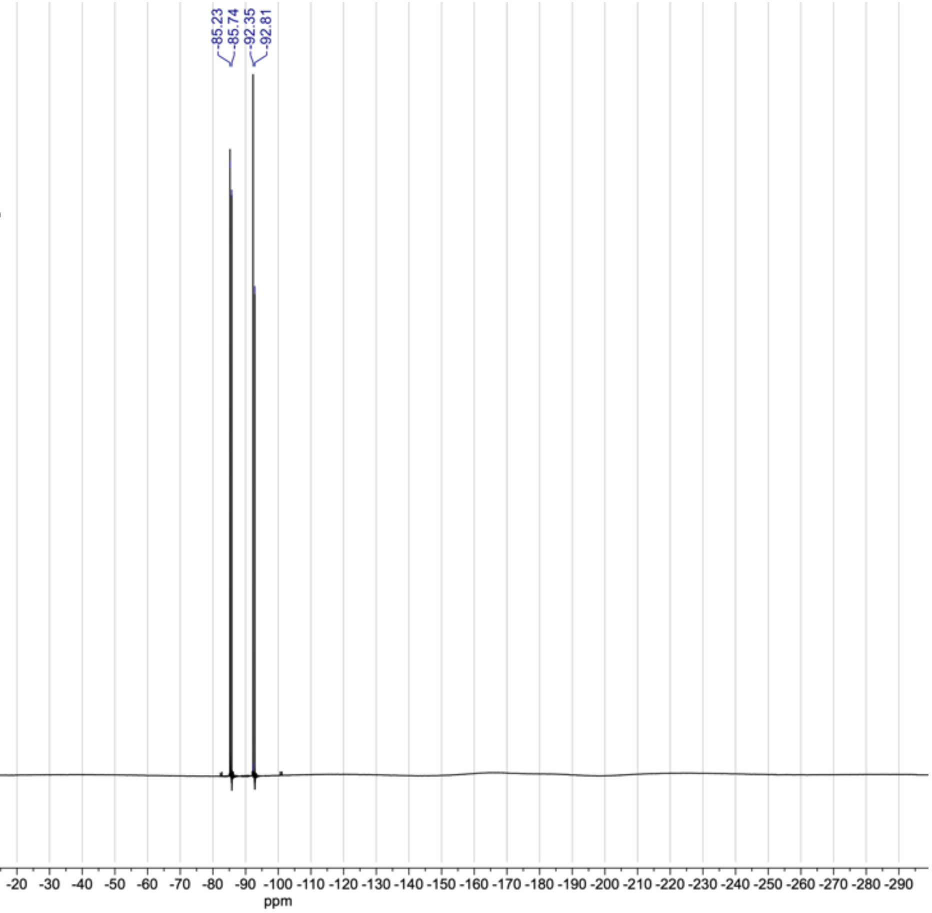

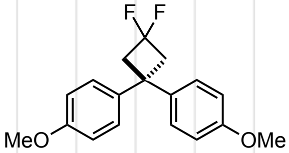

38

^{1}H NMR (400 MHz, $CDCl_3$)

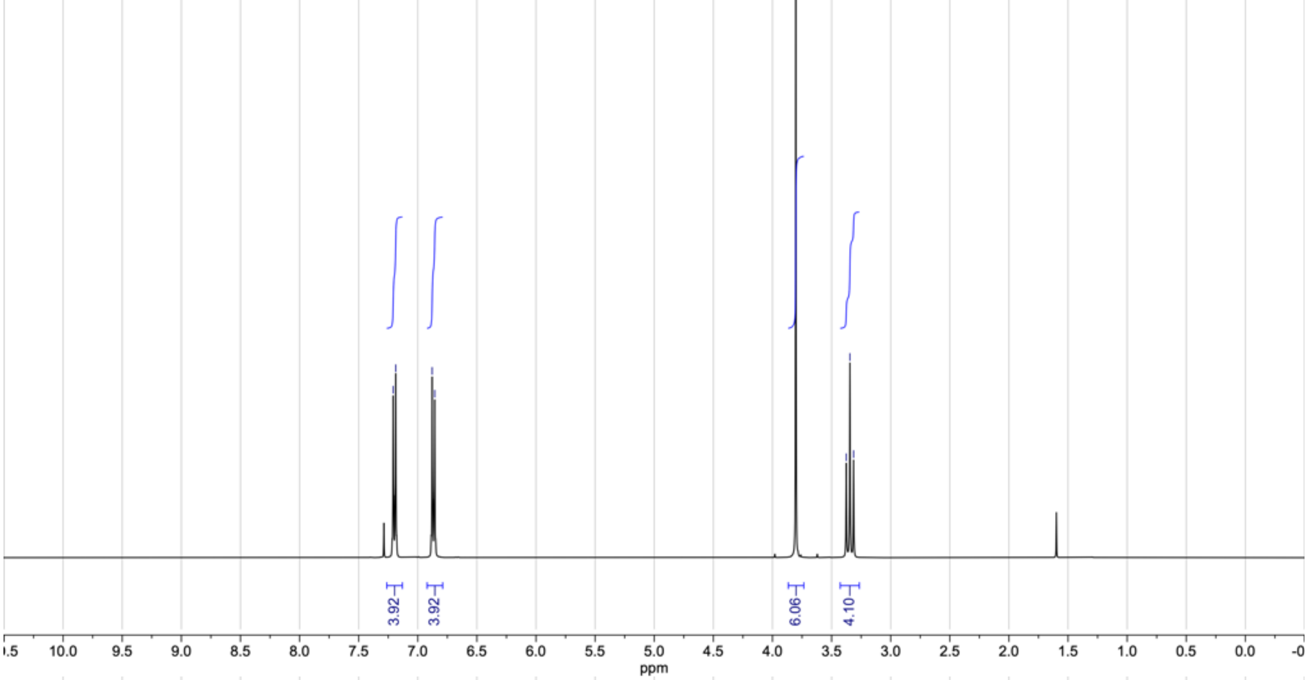

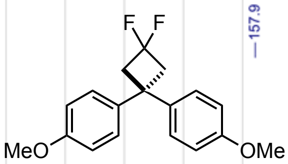

38

$^{13}C\{^1H\}$ NMR (101 MHz, $CDCl_3$)

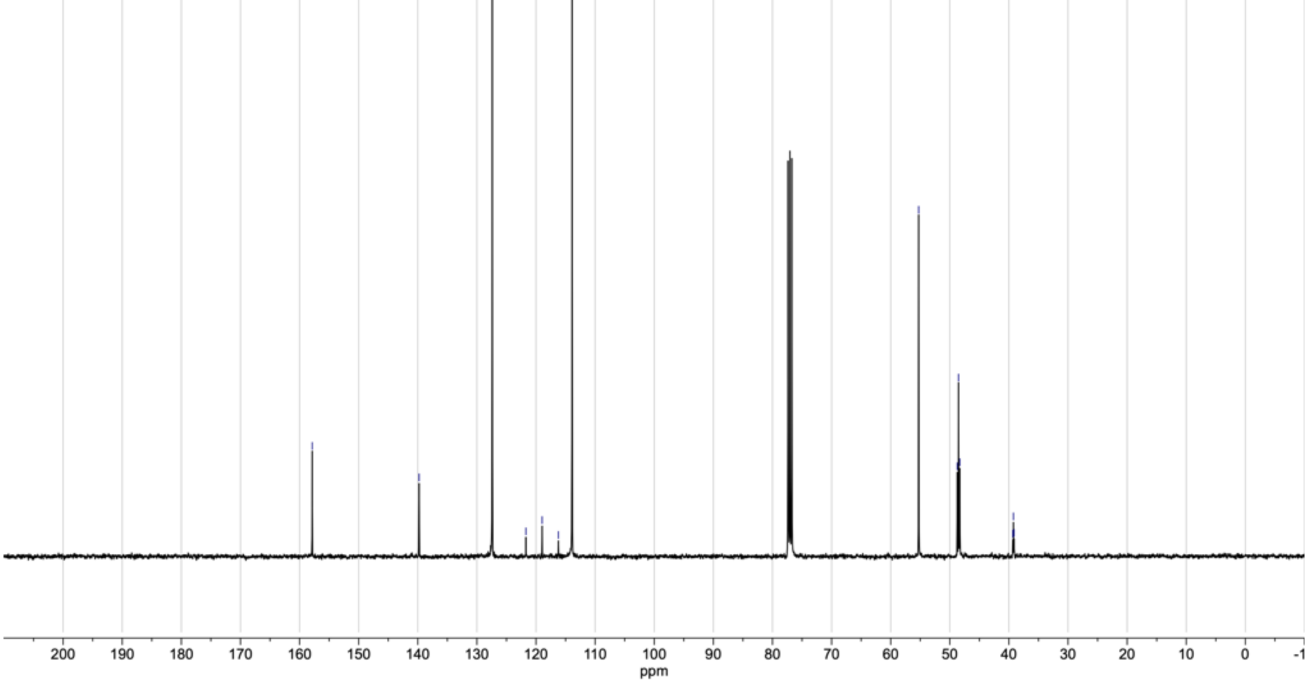

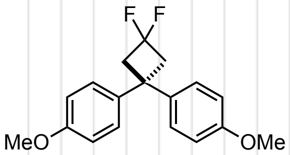

38

^{19}F NMR (377 MHz, $CDCl_3$)

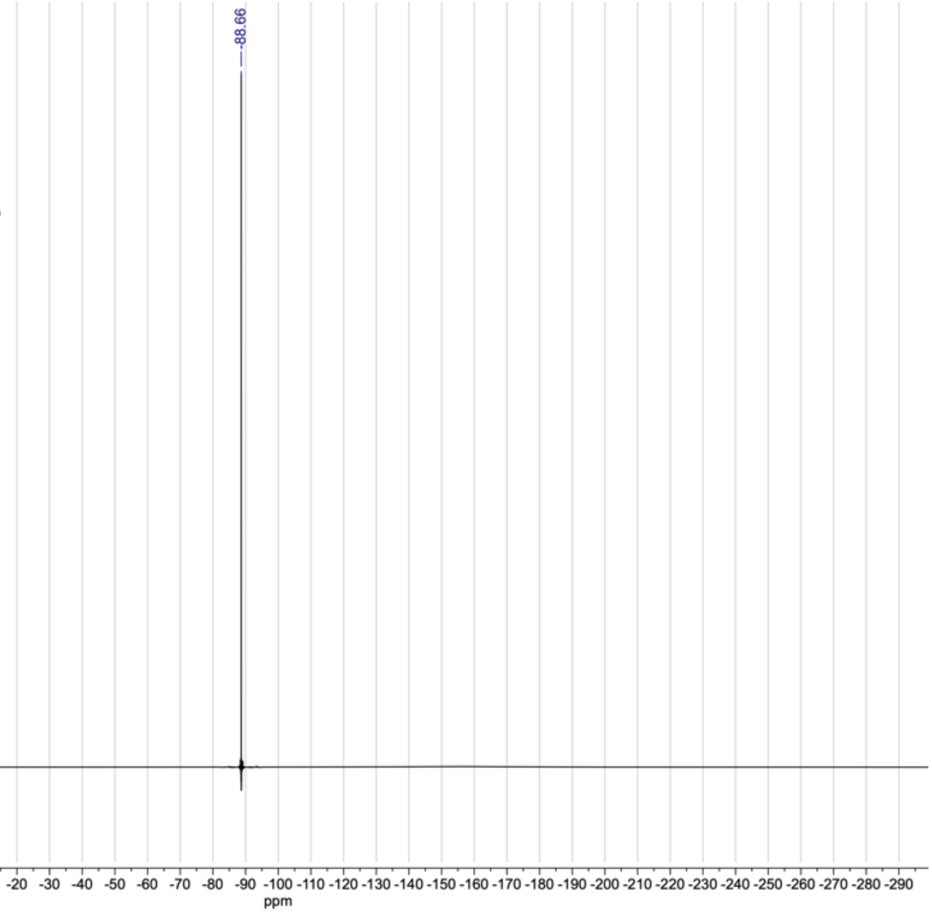

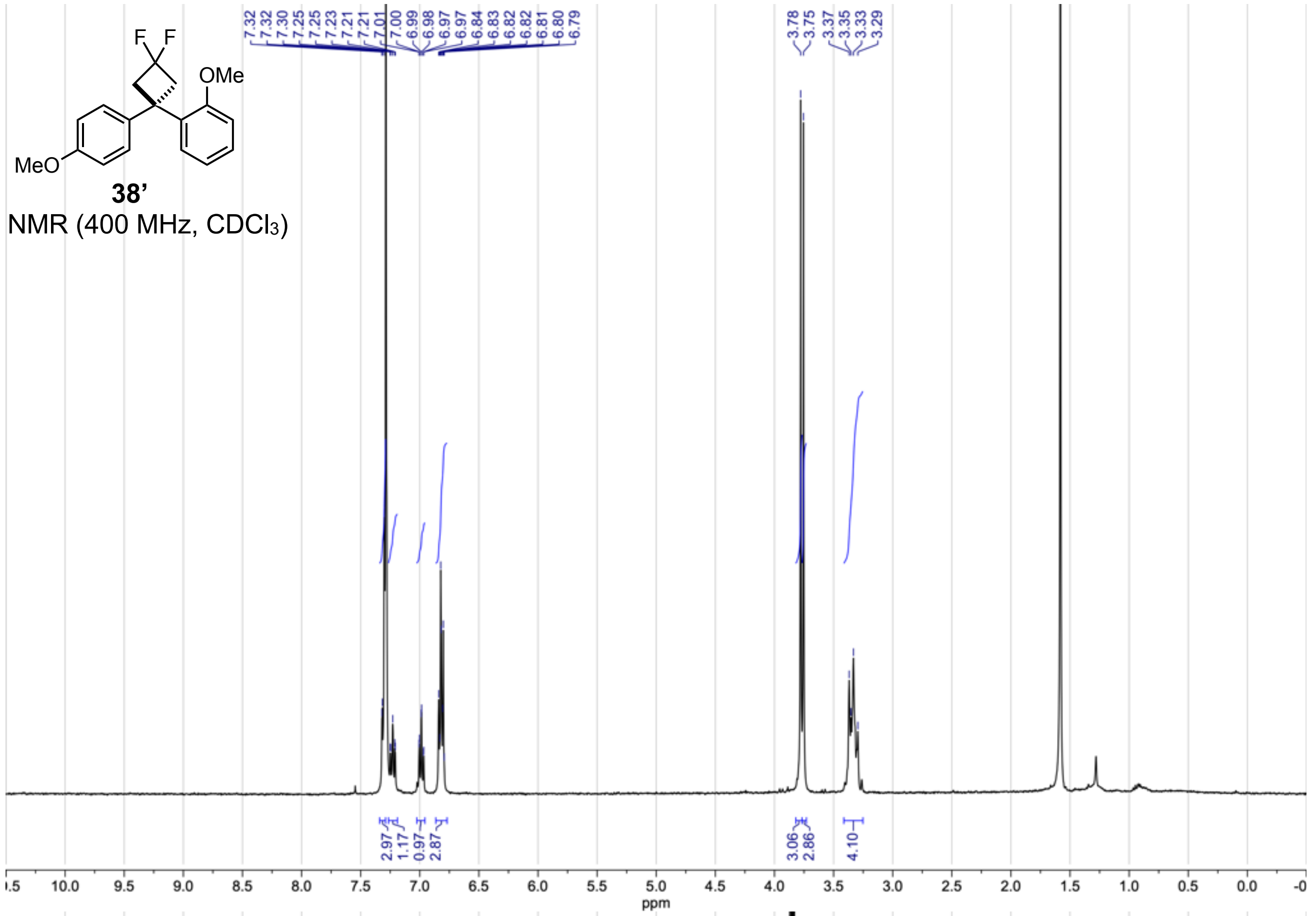

38'

^{1}H NMR (400 MHz, $CDCl_3$)

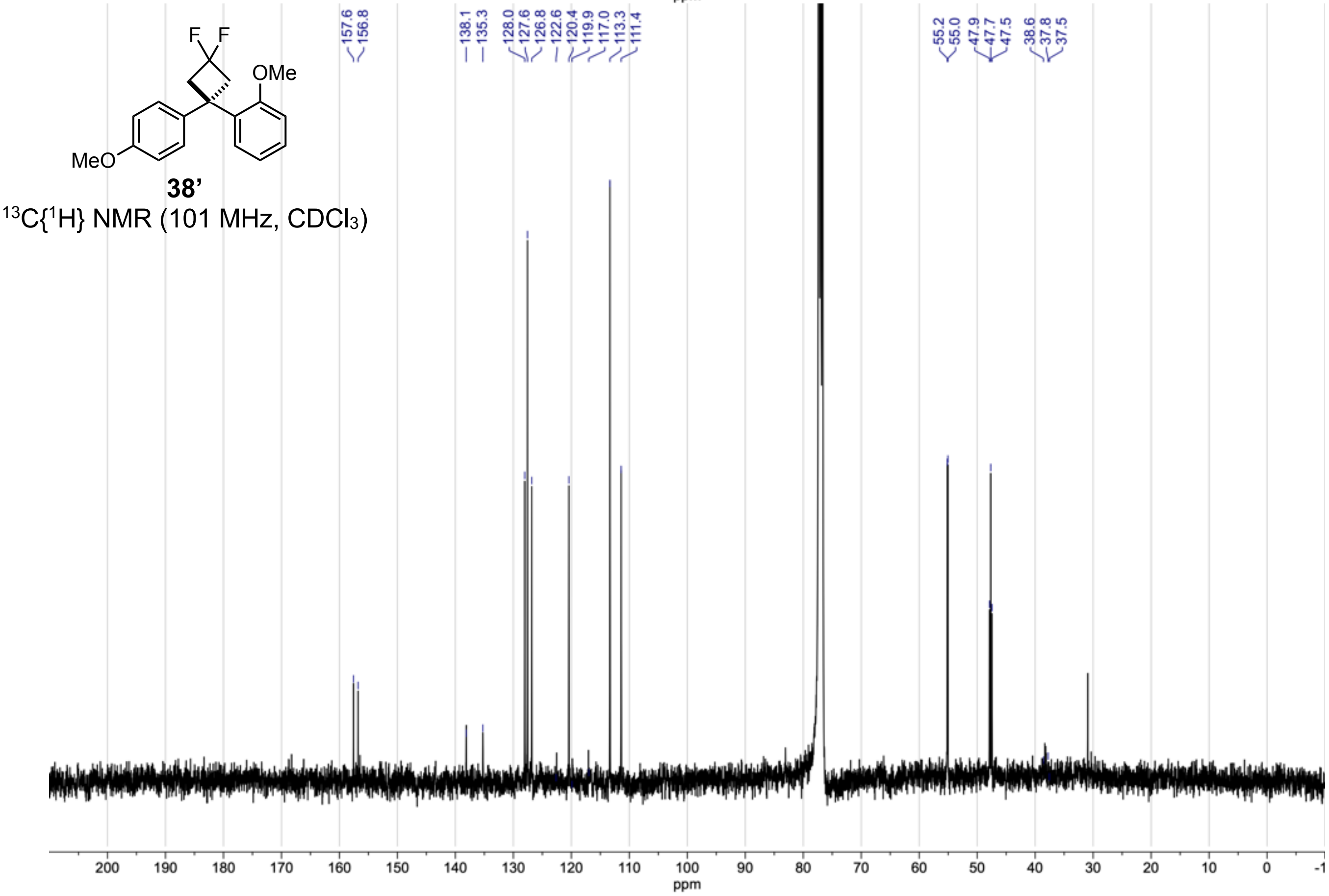

38'

$^{13}C\{^1H\}$ NMR (101 MHz, $CDCl_3$)

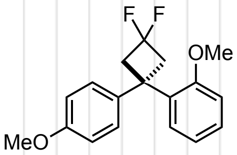

38'

^{19}F NMR (377 MHz, $CDCl_3$)

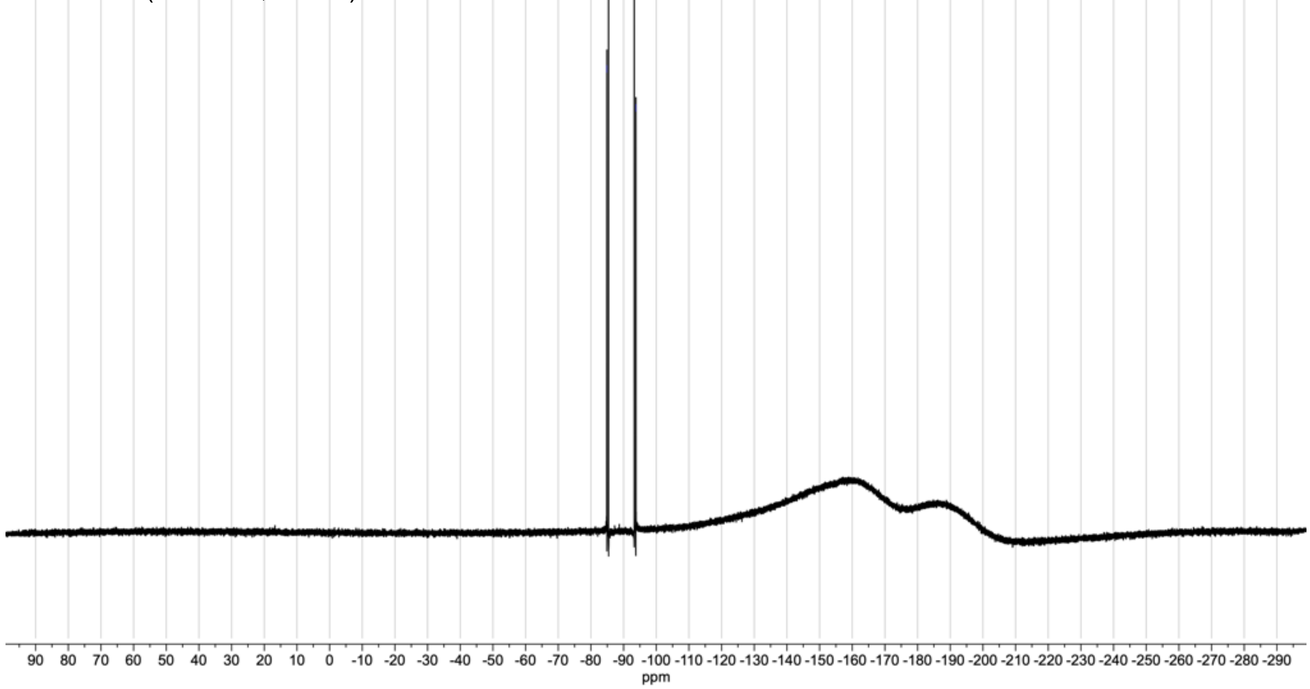

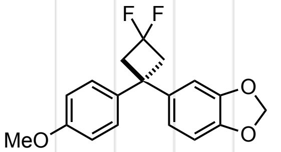

39

^{1}H NMR (400 MHz, $CDCl_3$)

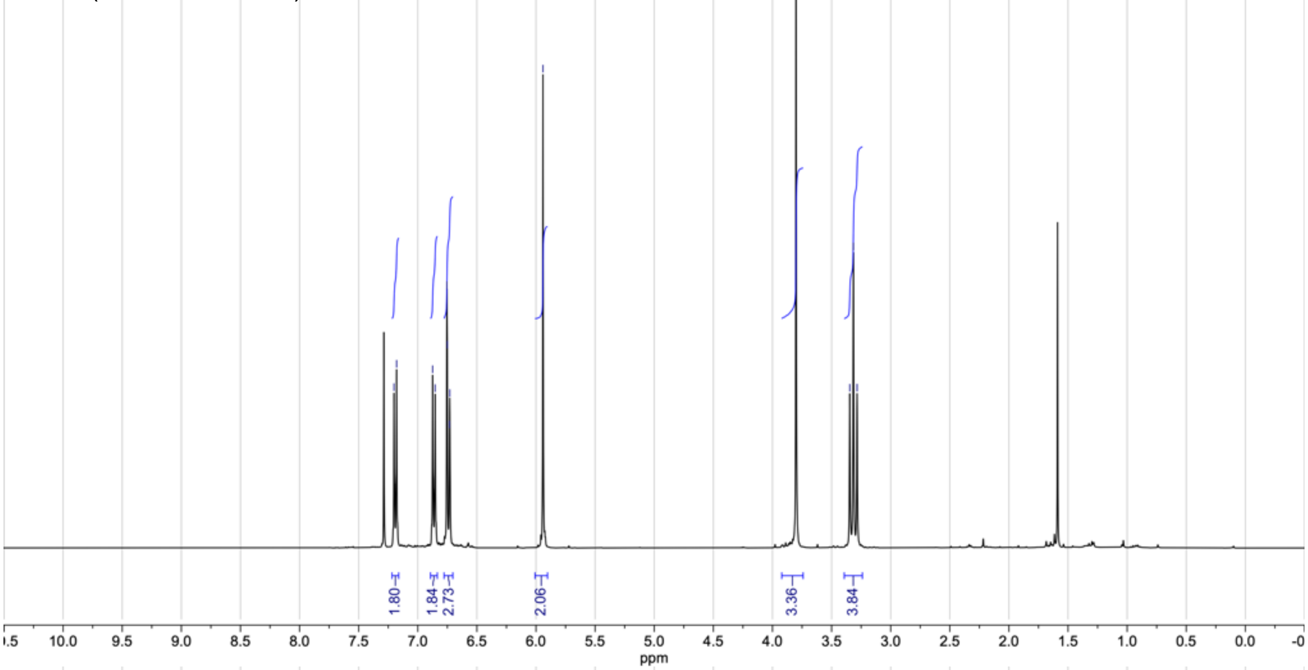

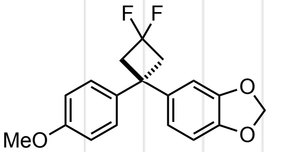

39

^{13}C{^{1}H} NMR (101 MHz, $CDCl_3$)

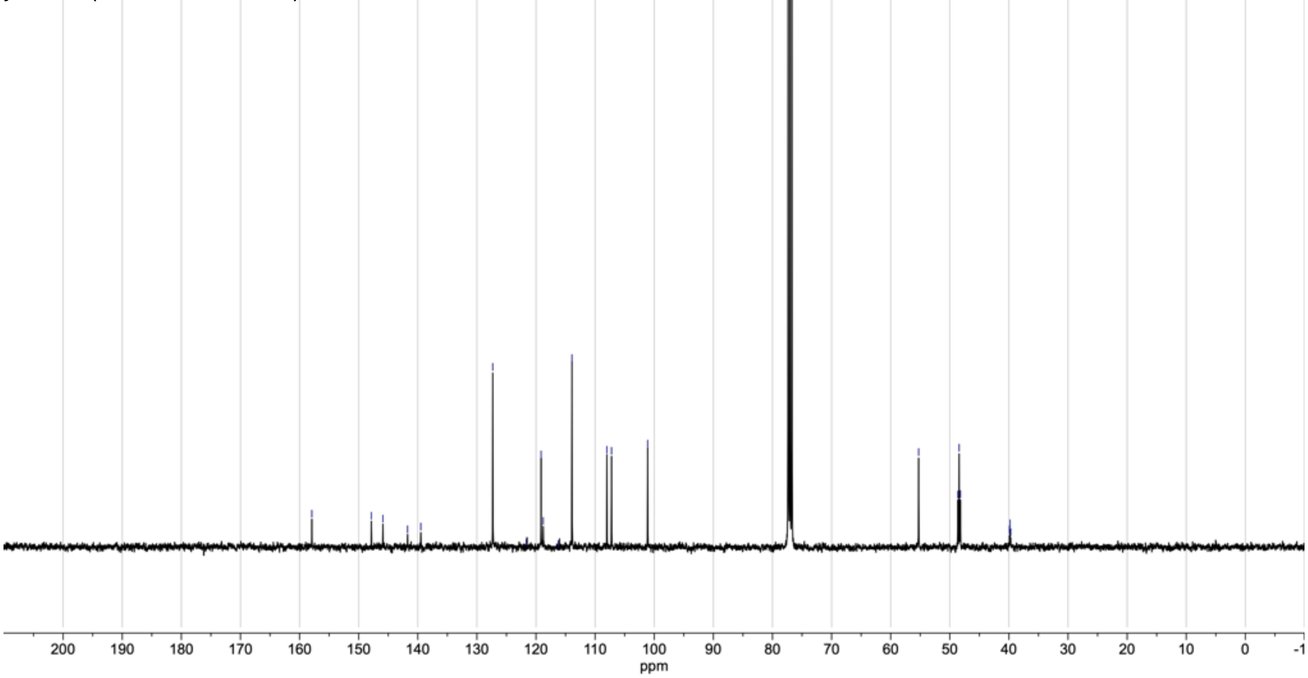

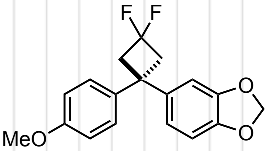

39

^{19}F NMR (377 MHz, $CDCl_3$)

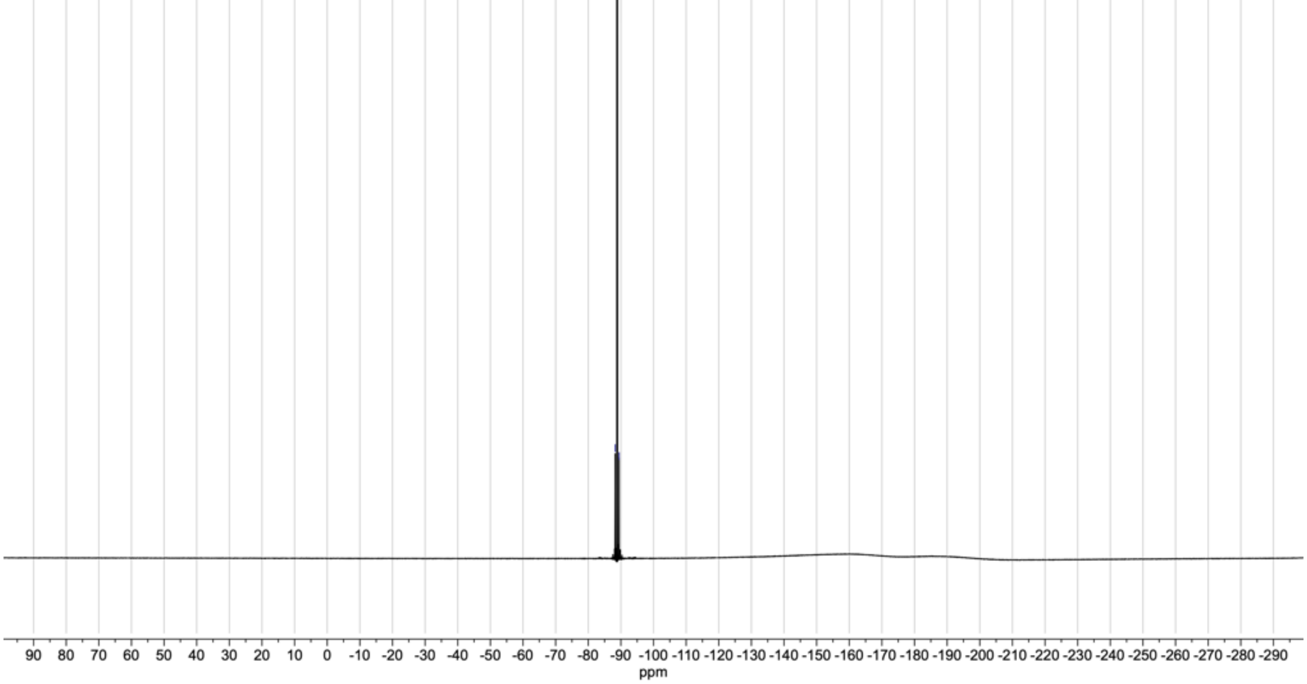

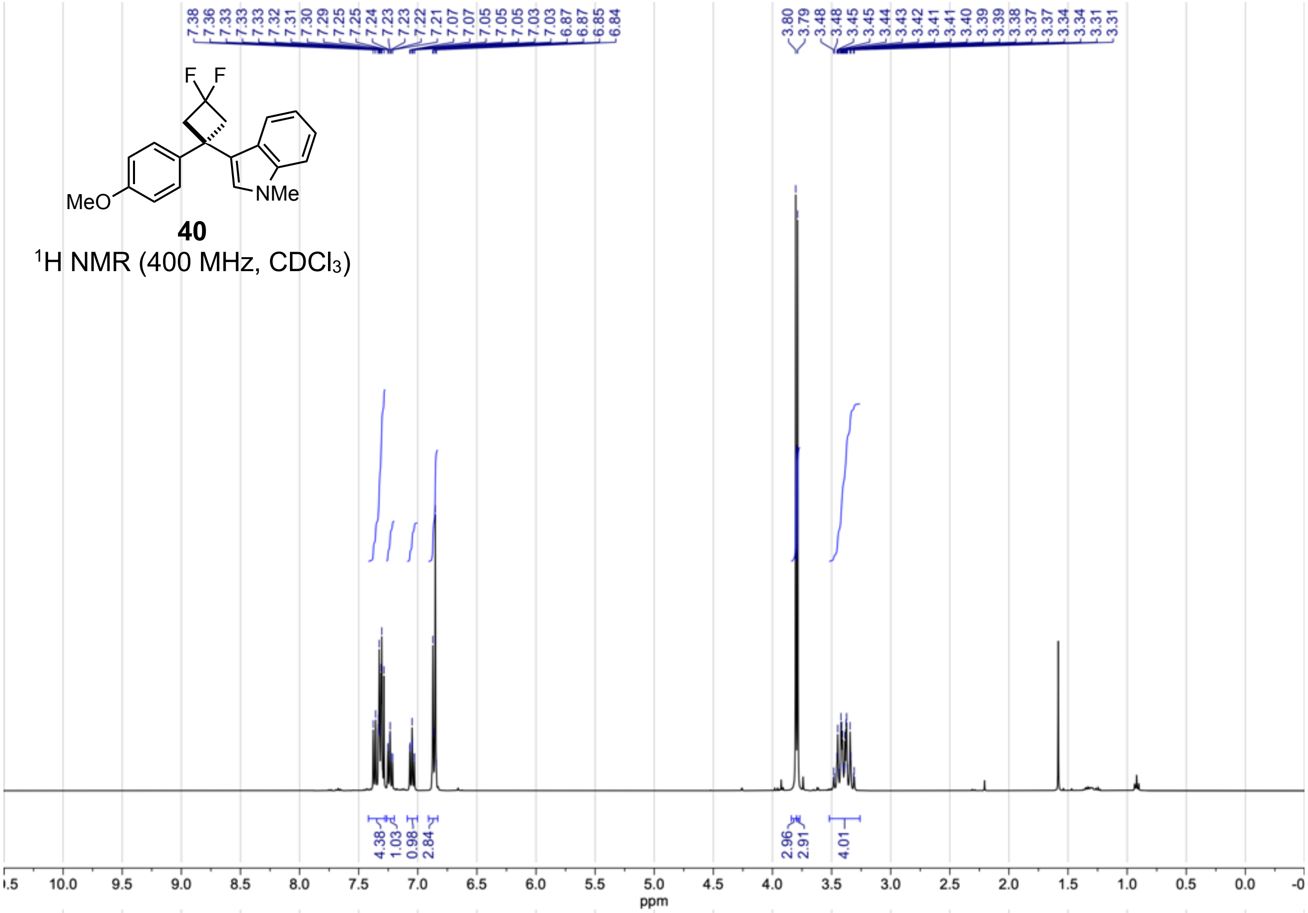
F F
MeO
NMe
40
¹H NMR (400 MHz, CDCl₃)
ppm

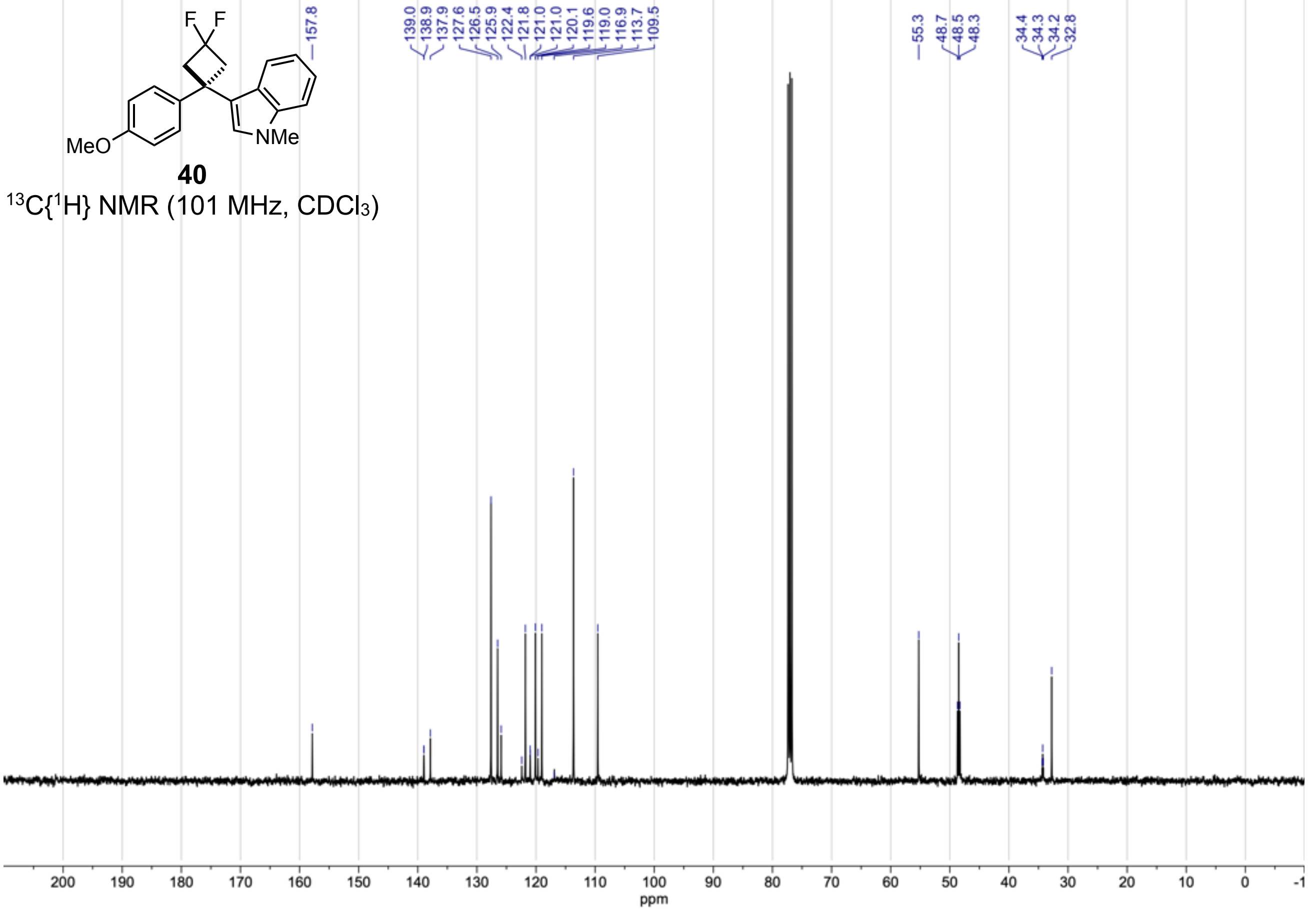
F F
MeO
NMe
40
¹³C{¹H} NMR (101 MHz, CDCl₃)
ppm

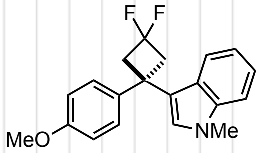

40

^{19}F NMR (377 MHz, $CDCl_3$)

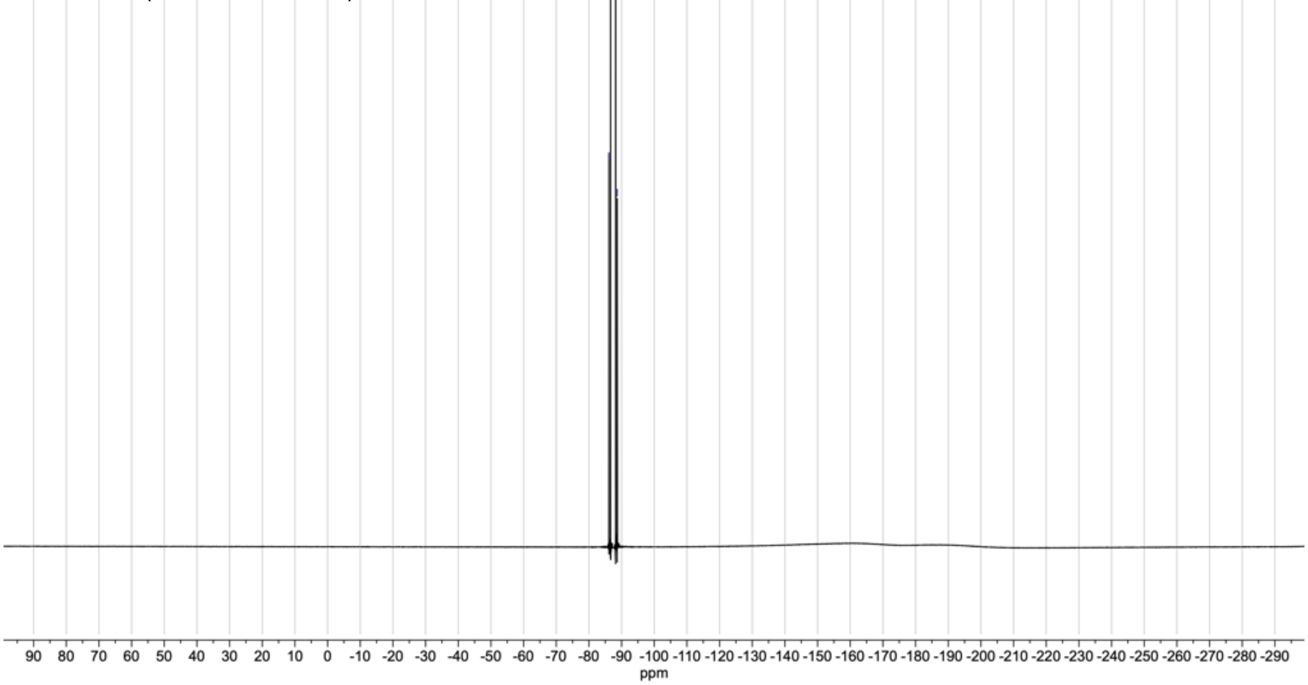

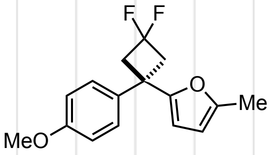

41

^{1}H NMR (400 MHz, $CDCl_3$)

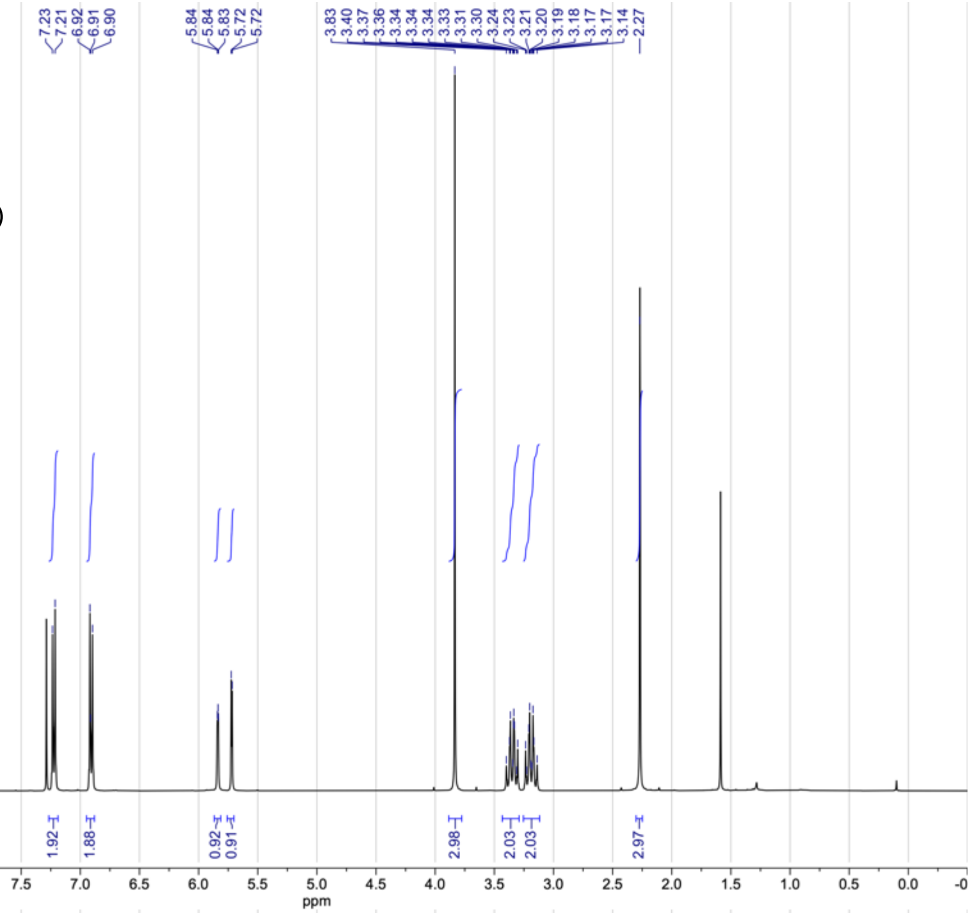

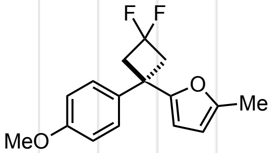

41

$^{13}C\{^{1}H\}$ NMR (101 MHz, $CDCl_3$)

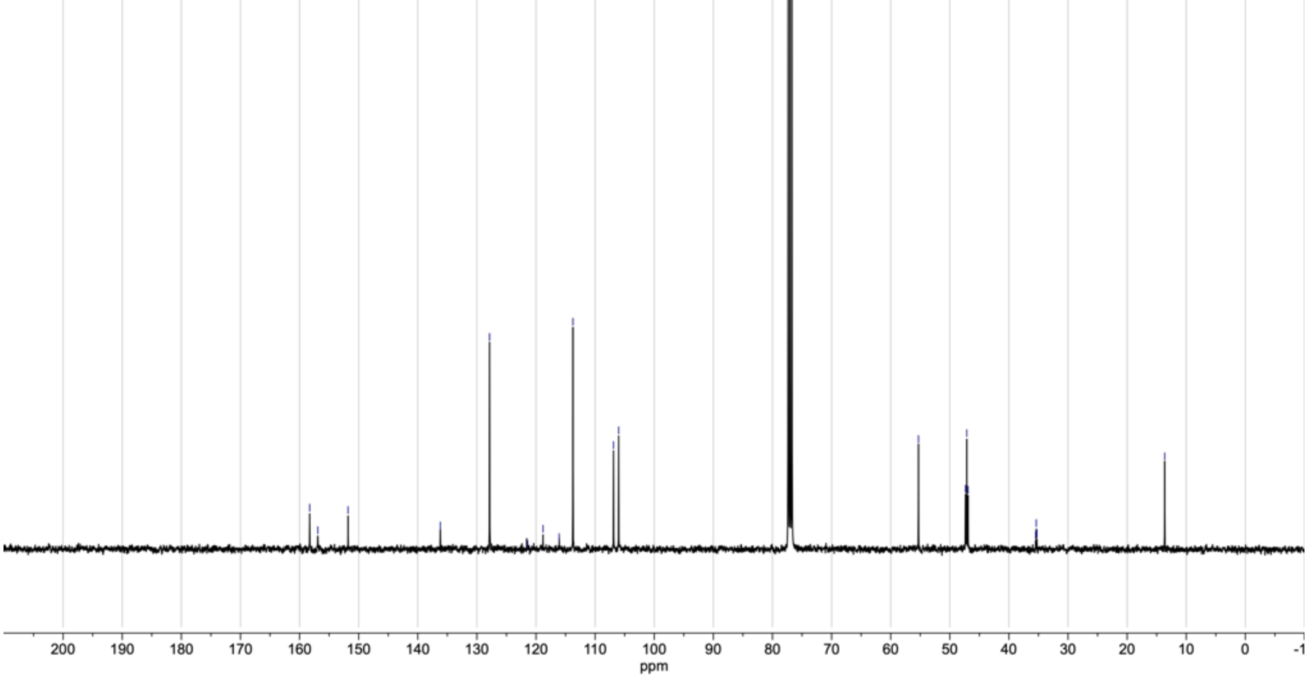

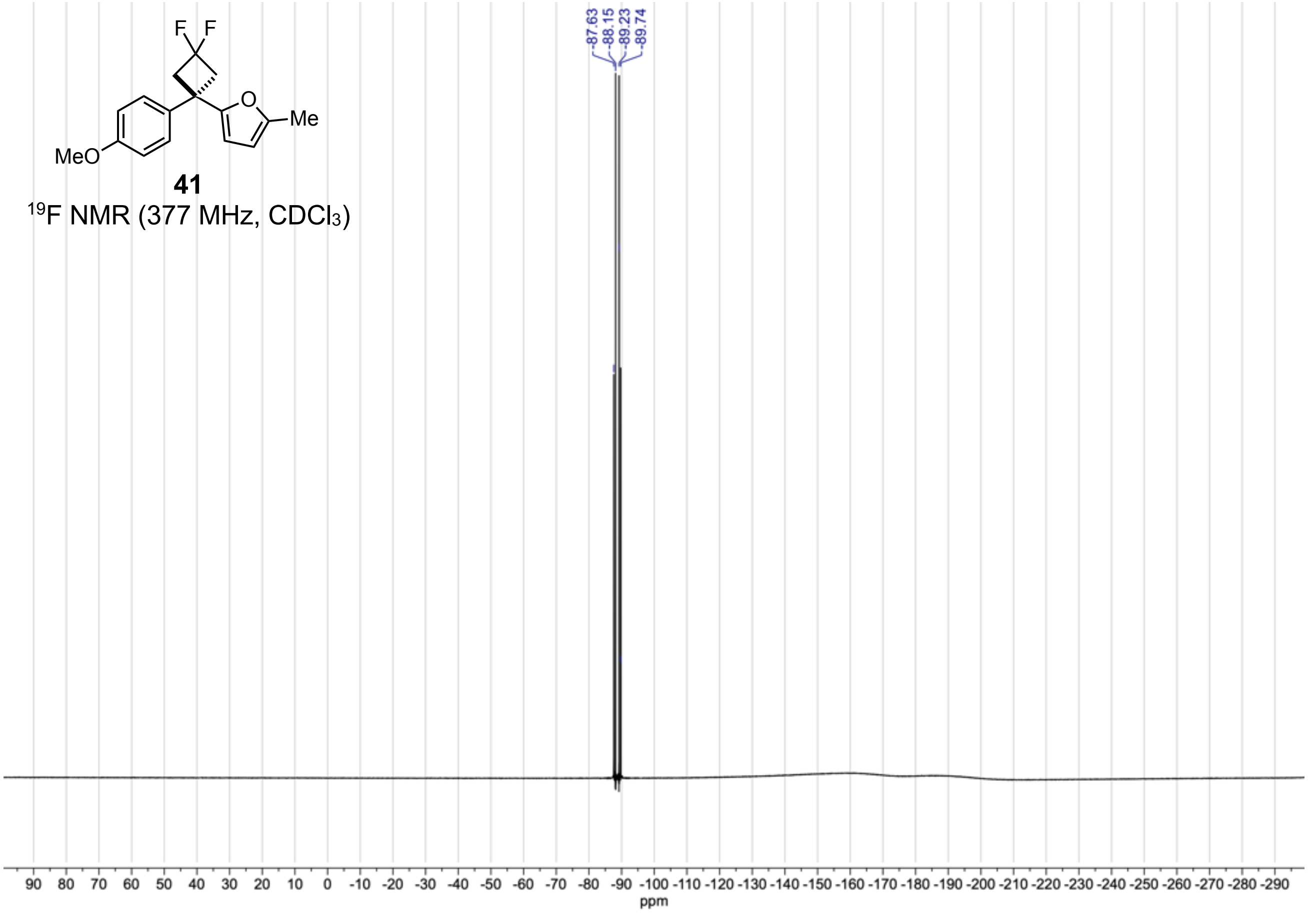

41

^{19}F NMR (377 MHz, $CDCl_3$)

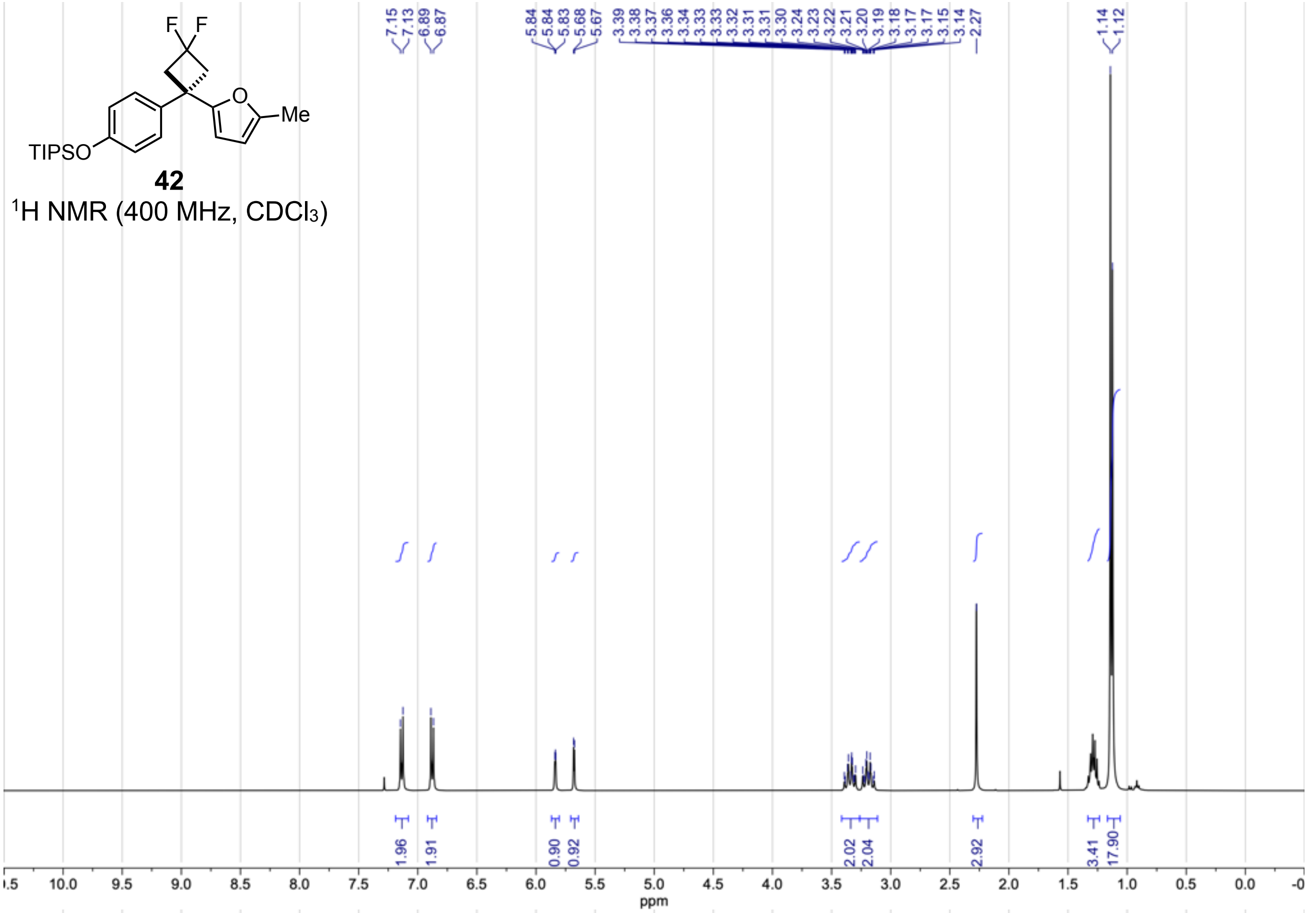
F F
O
Me
TIPSO
42
^{1}H NMR (400 MHz, $CDCl_3$)
7.15 7.13 6.89 6.87
5.84 5.84 5.83 5.68 5.67
3.39 3.38 3.37 3.36 3.34 3.33 3.33 3.32 3.31 3.31 3.30 3.24 3.23 3.22 3.22 3.21 3.20 3.19 3.18 3.17 3.17 3.15 3.14
2.27
1.14 1.12
1.96 1.91 0.90 0.92 2.02 2.04 2.92 3.41 17.90
ppm

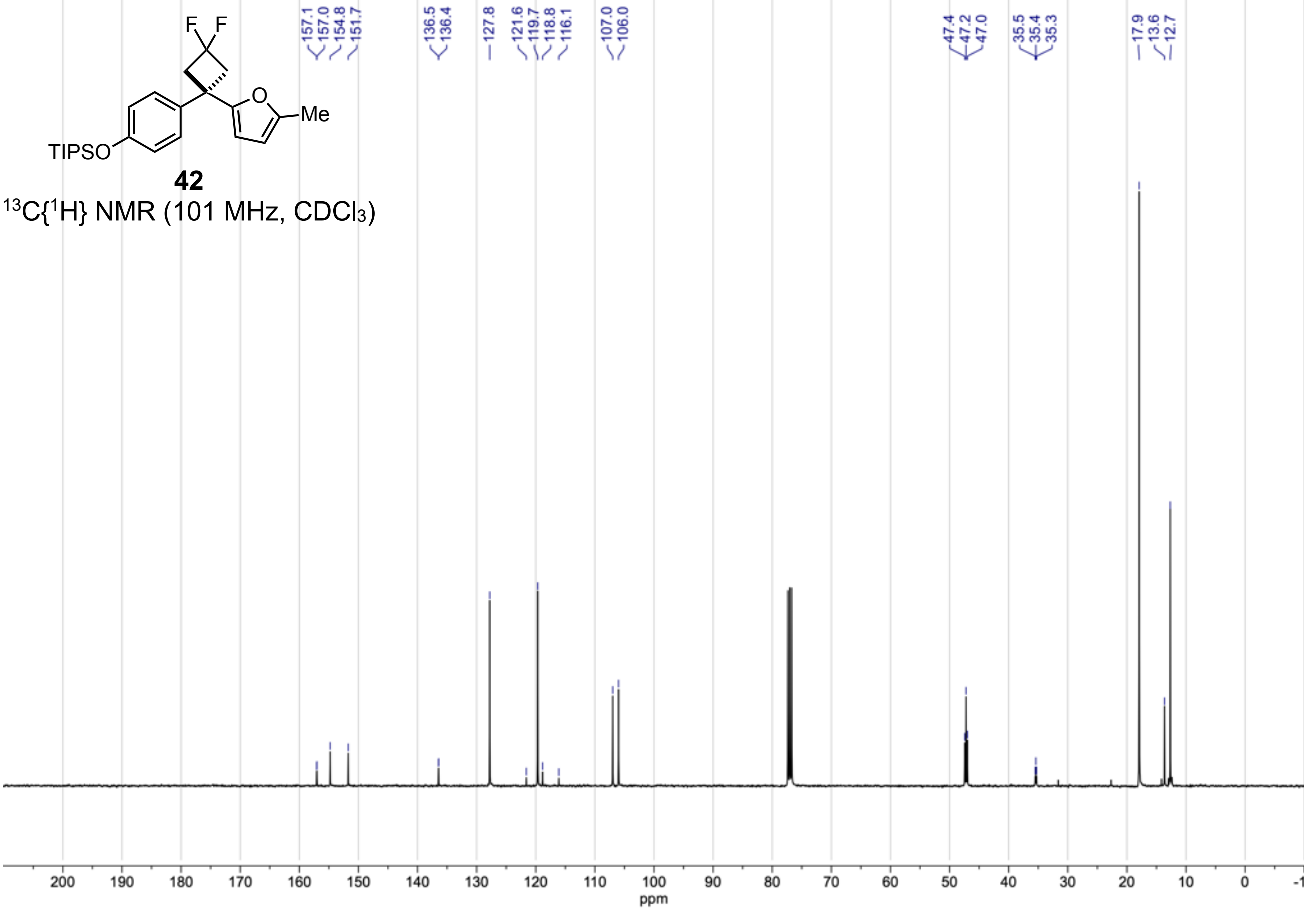
F F
O
Me
TIPSO
42
$^{13}C\{^{1}H\}$ NMR (101 MHz, $CDCl_3$)
157.1 157.0 154.8 151.7
136.5 136.4
127.8
121.6 119.7 118.8 116.1
107.0 106.0
47.4 47.2 47.0
35.5 35.4 35.3
17.9 13.6 12.7
ppm

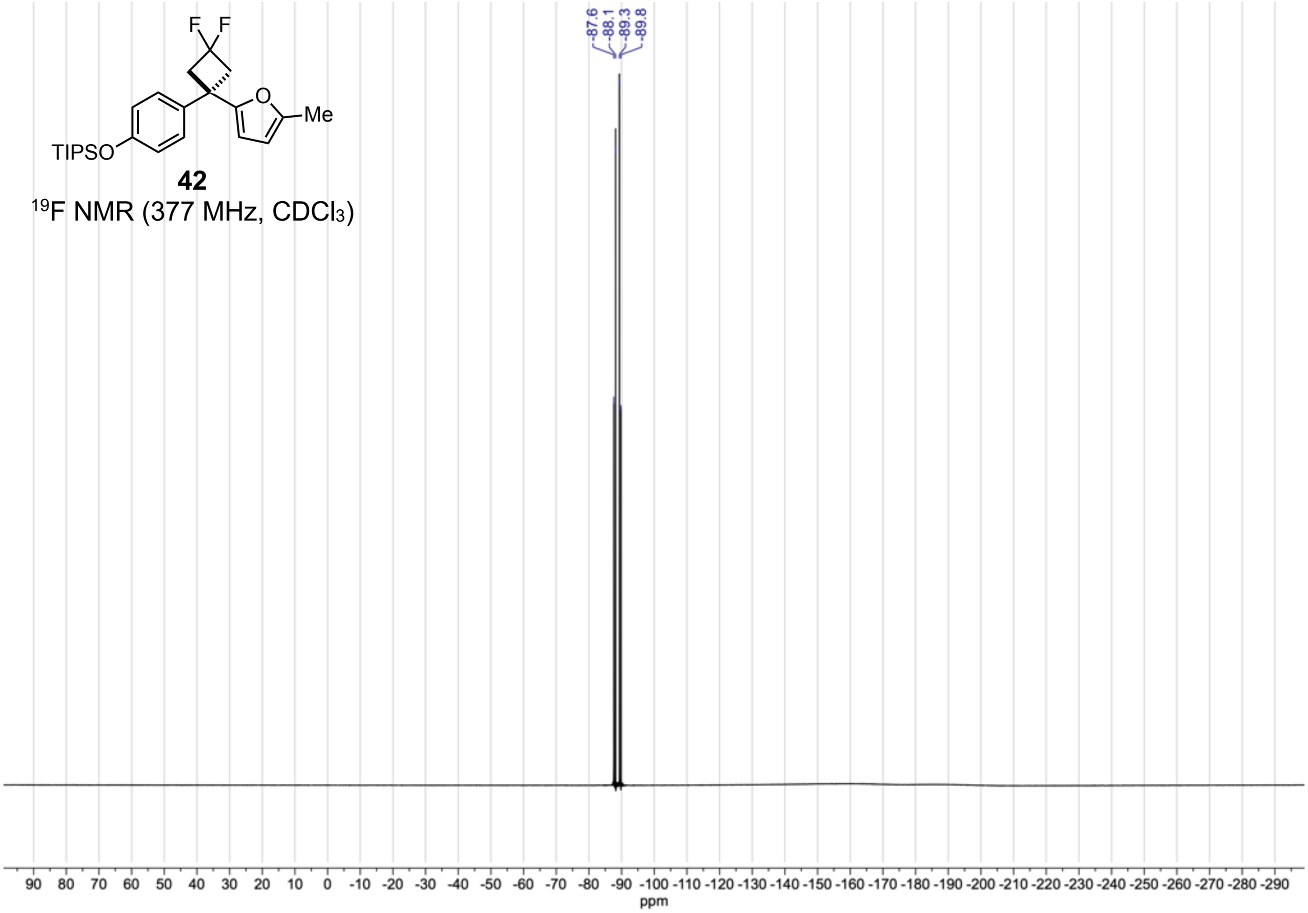
F
F
O
Me
TIPSO
42
19F NMR (377 MHz, CDCl3)
-87.6
-88.1
-89.3
-89.8
90 80 70 60 50 40 30 20 10 0 -10 -20 -30 -40 -50 -60 -70 -80 -90 -100 -110 -120 -130 -140 -150 -160 -170 -180 -190 -200 -210 -220 -230 -240 -250 -260 -270 -280 -290
ppm

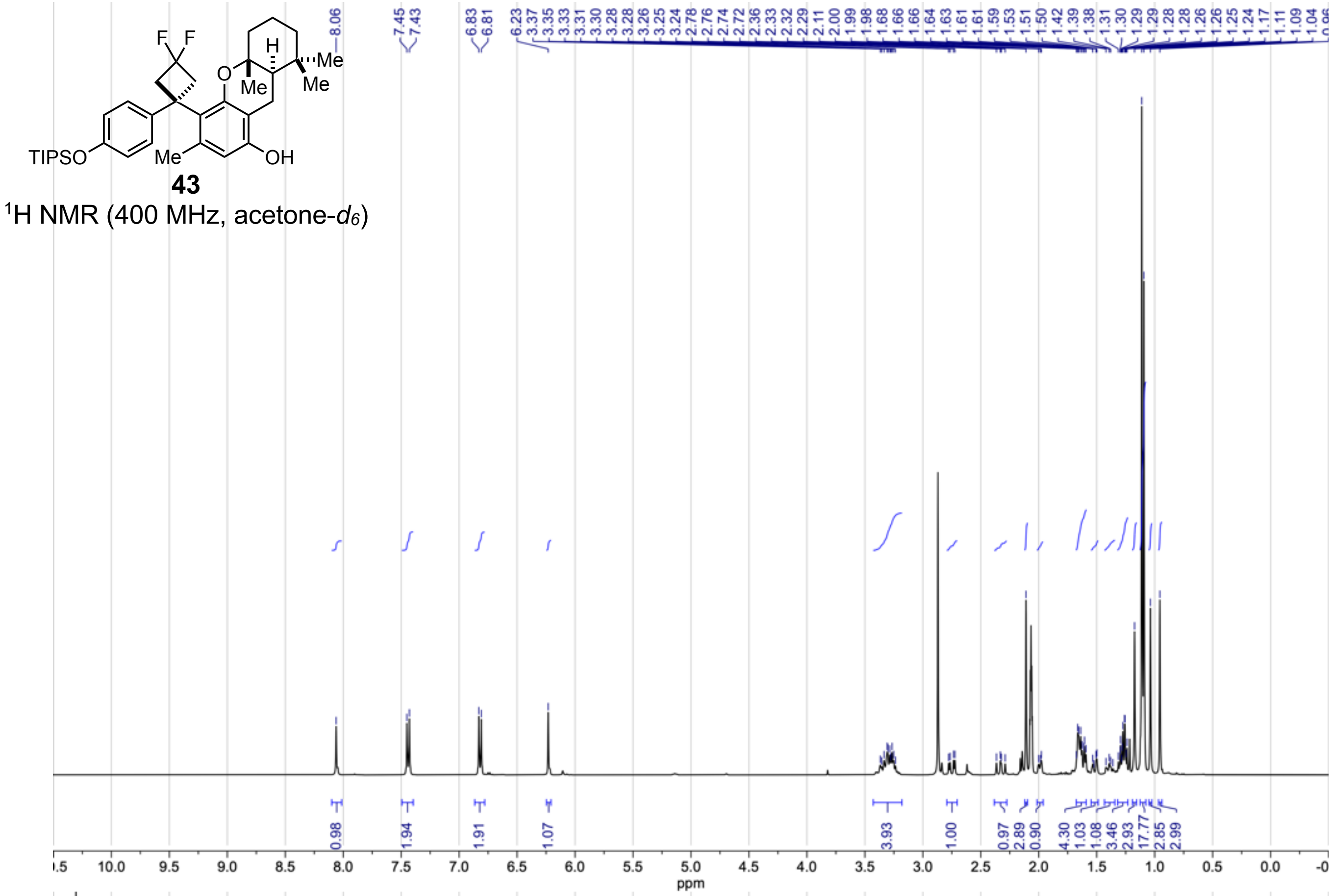

43

^{1}H NMR (400 MHz, acetone-d_6)

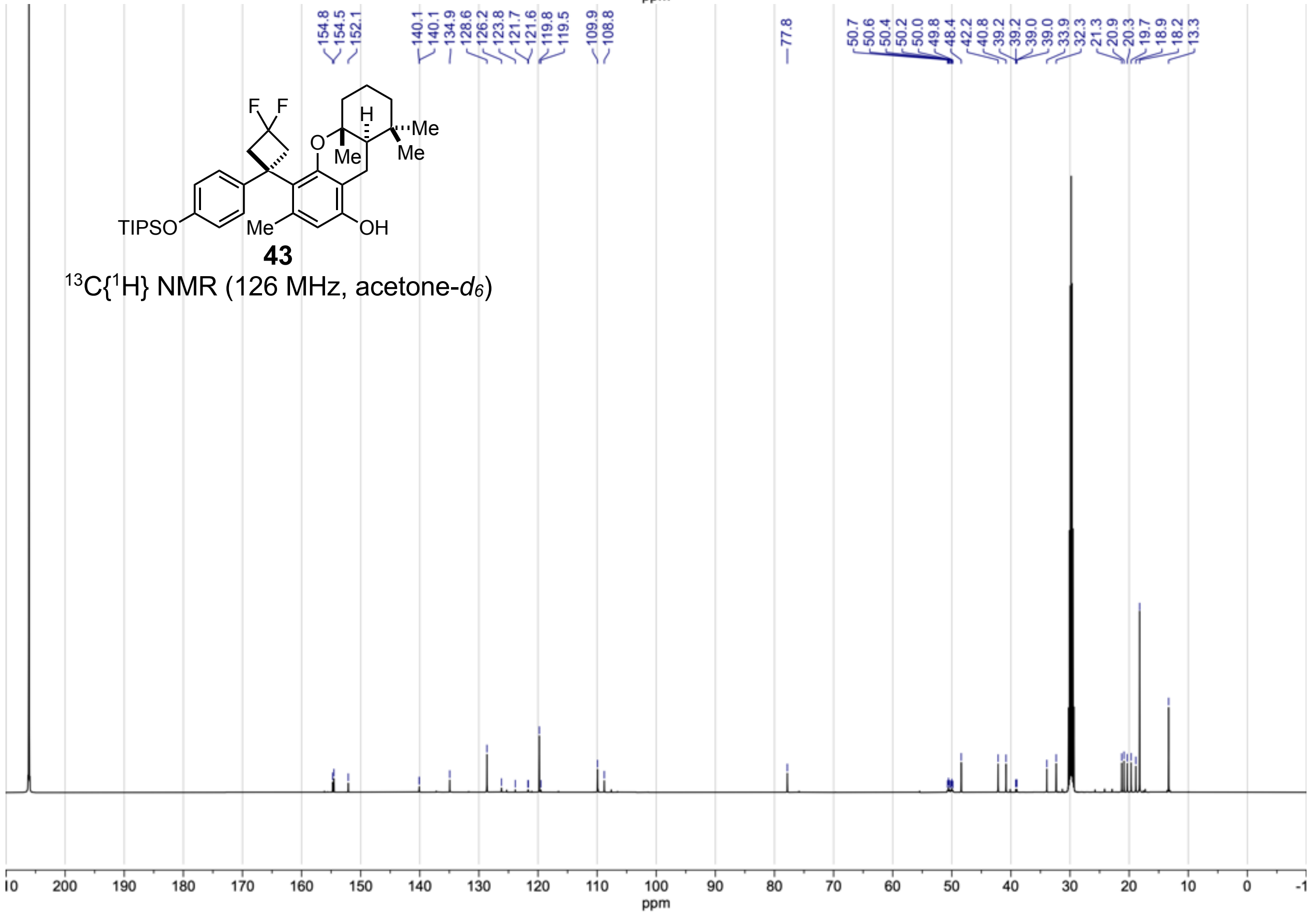

43

$^{13}C\{^{1}H\}$ NMR (126 MHz, acetone-d_6)

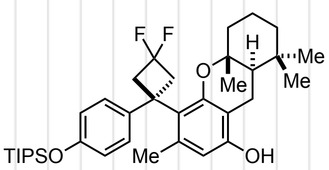

43

^{19}F NMR (377 MHz, acetone-d_6)

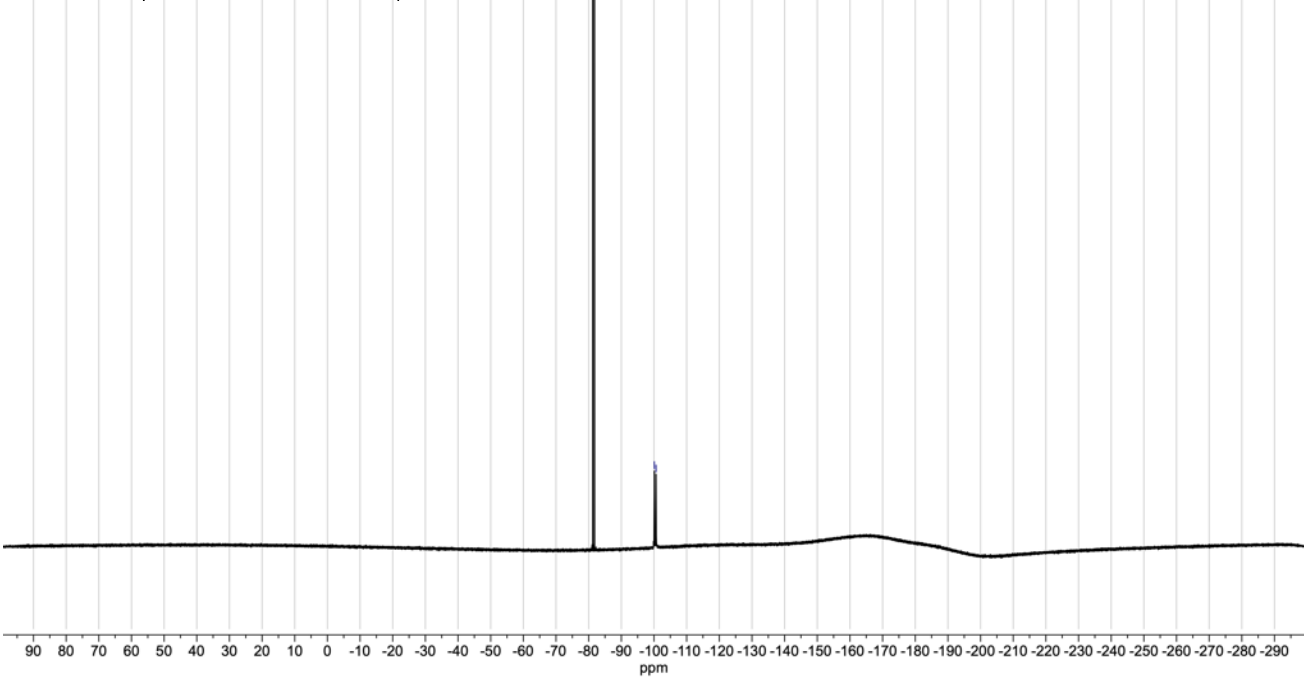

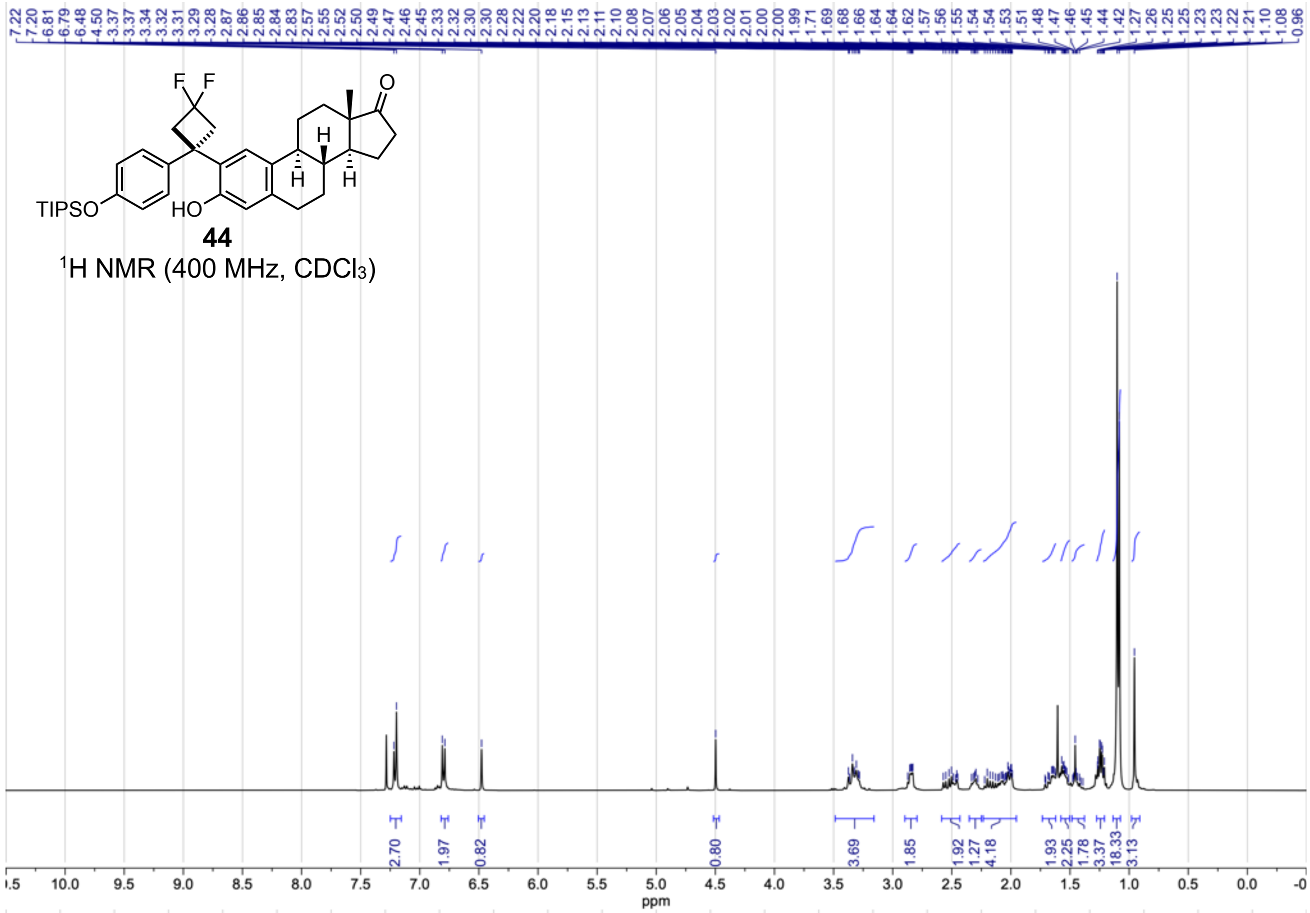

44

^{1}H NMR (400 MHz, $CDCl_3$)

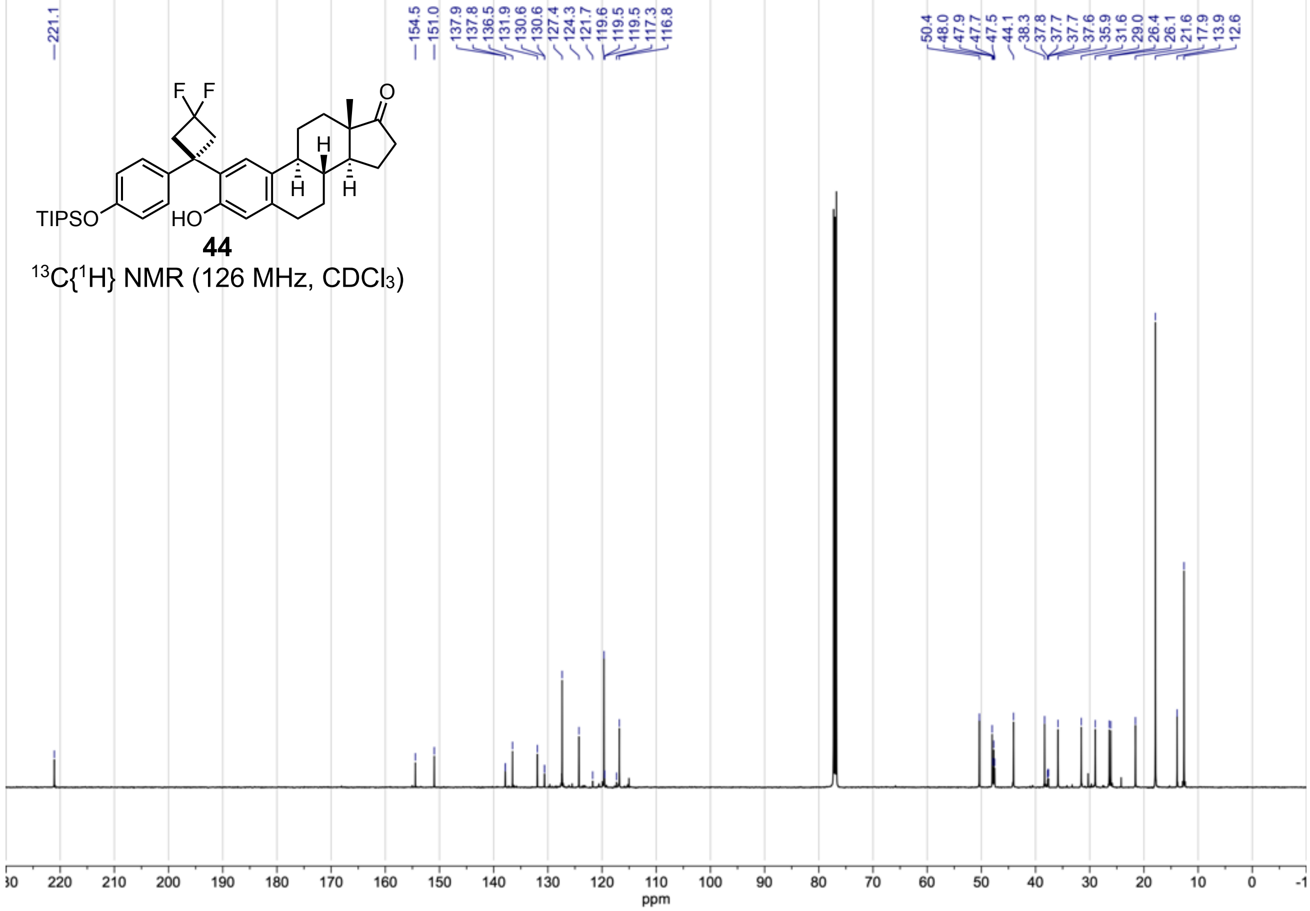

44

$^{13}C\{^{1}H\}$ NMR (126 MHz, $CDCl_3$)

44

^{19}F NMR (377 MHz, $CDCl_3$)

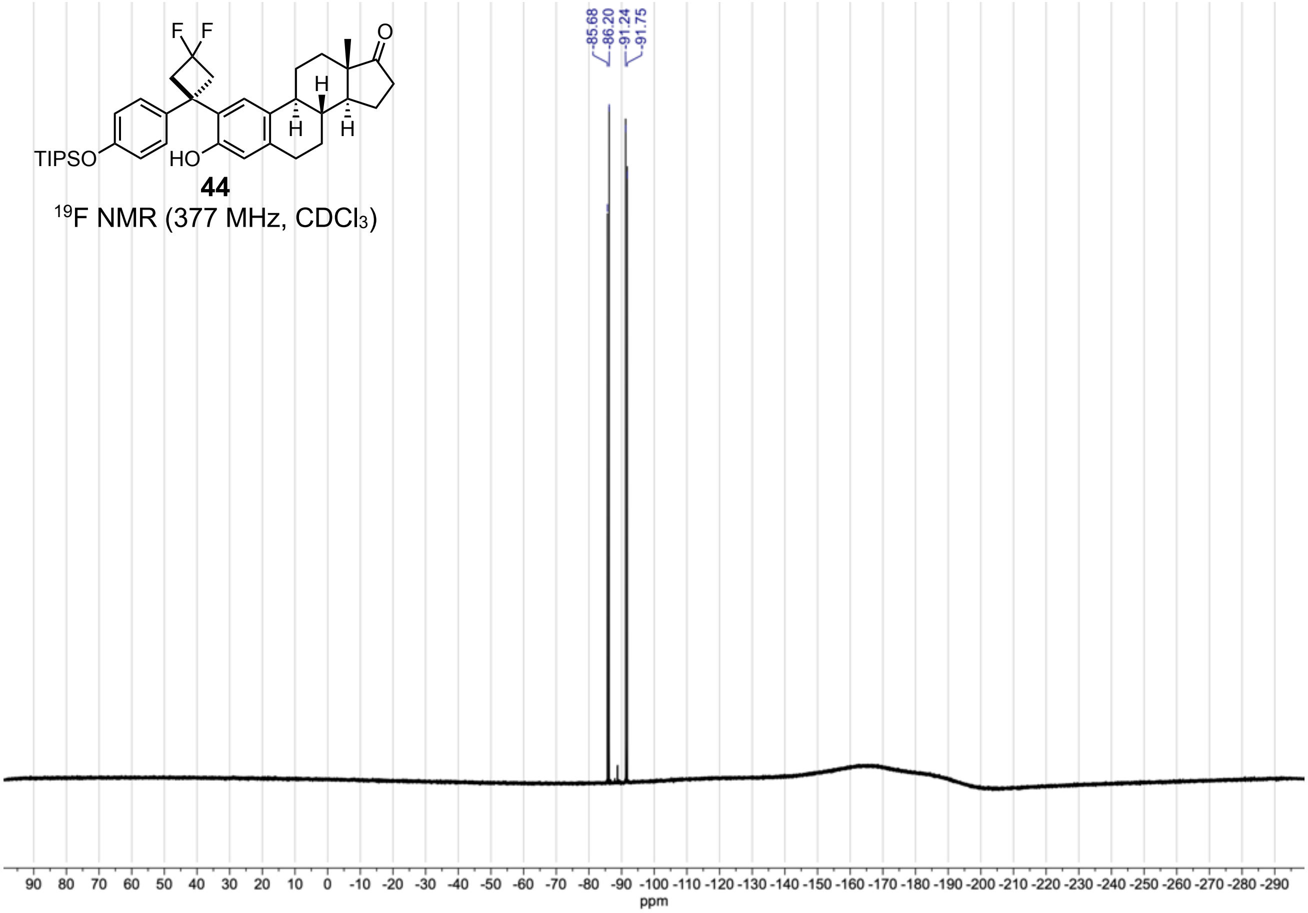

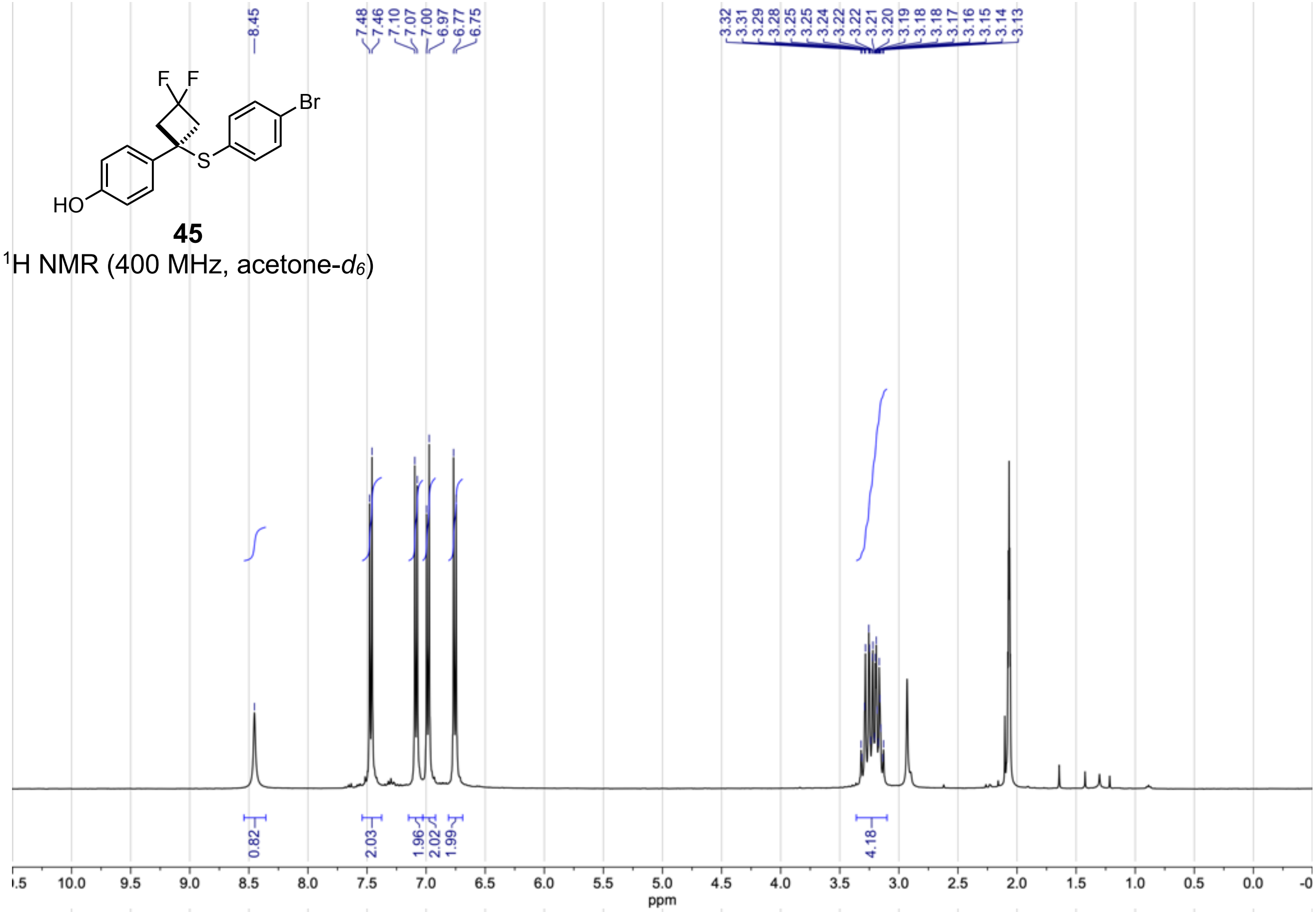

45

^{1}H NMR (400 MHz, acetone-d_6)

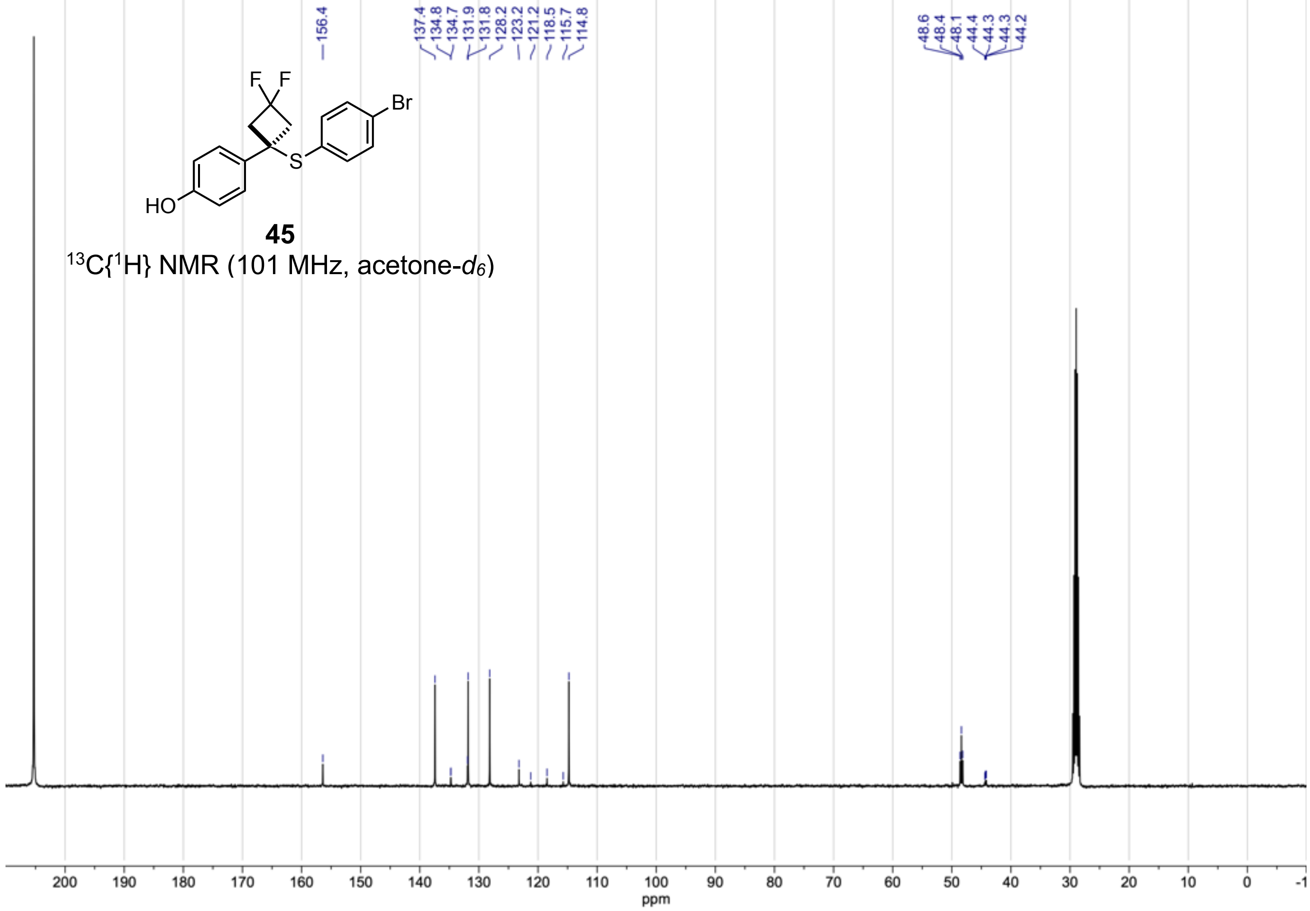

45

^{13}C{^{1}H} NMR (101 MHz, acetone-d_6)

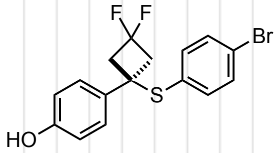

45

^{19}F NMR (377 MHz, acetone-d_6)

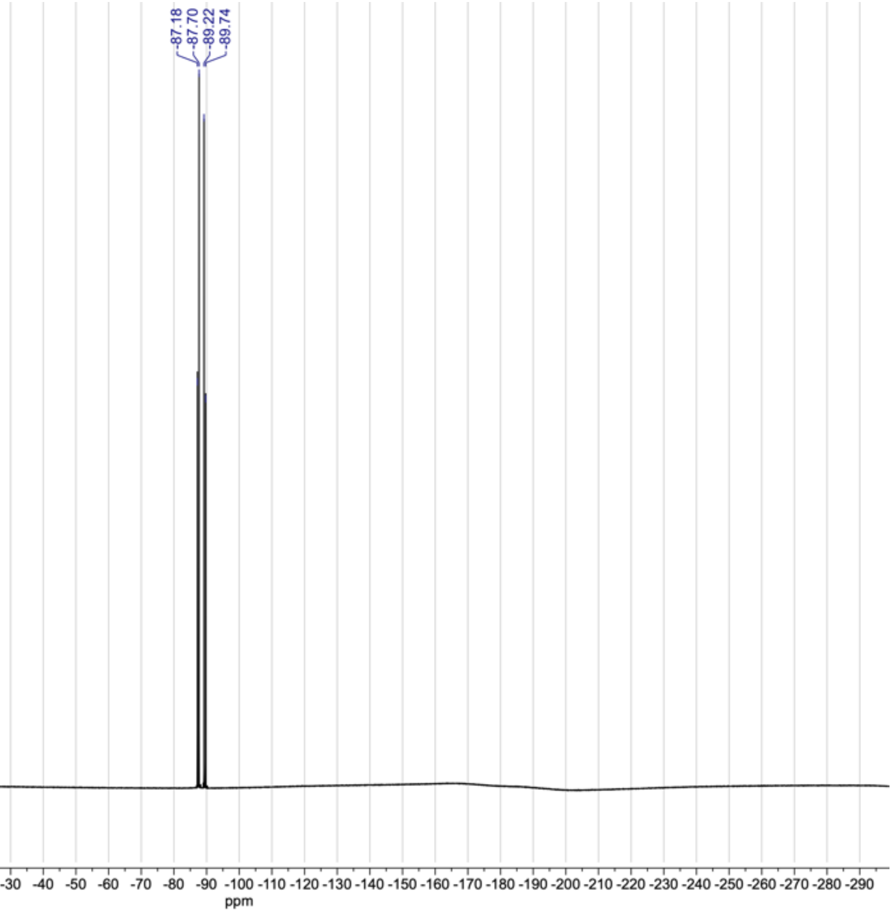

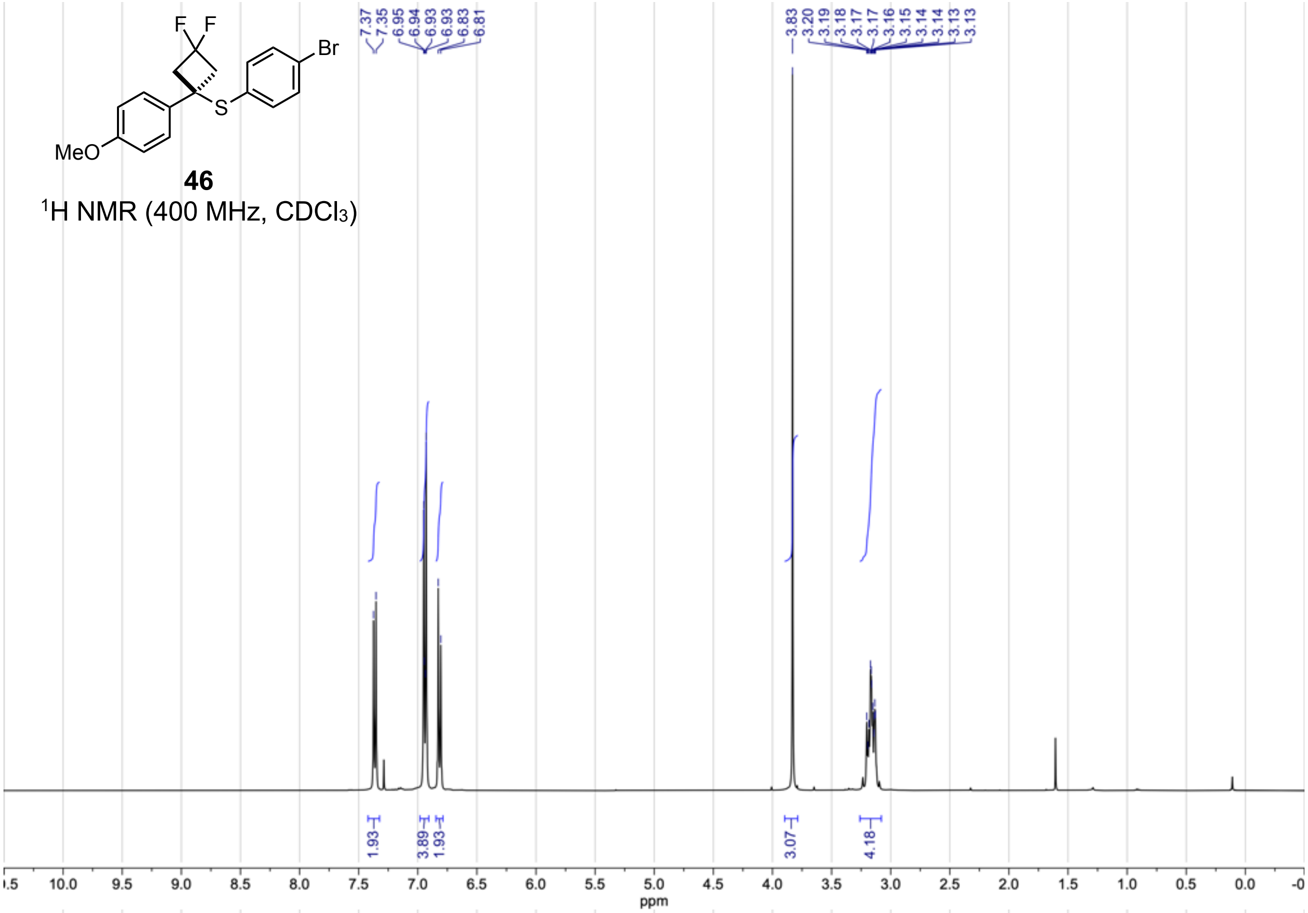
F
F
Br
S
MeO
46
1H NMR (400 MHz, CDCl3)
7.37
7.35
6.95
6.94
6.93
6.93
6.83
6.81
3.83
3.20
3.19
3.18
3.17
3.17
3.16
3.15
3.14
3.14
3.13
3.13
1.93
3.89
1.93
3.07
4.18
ppm

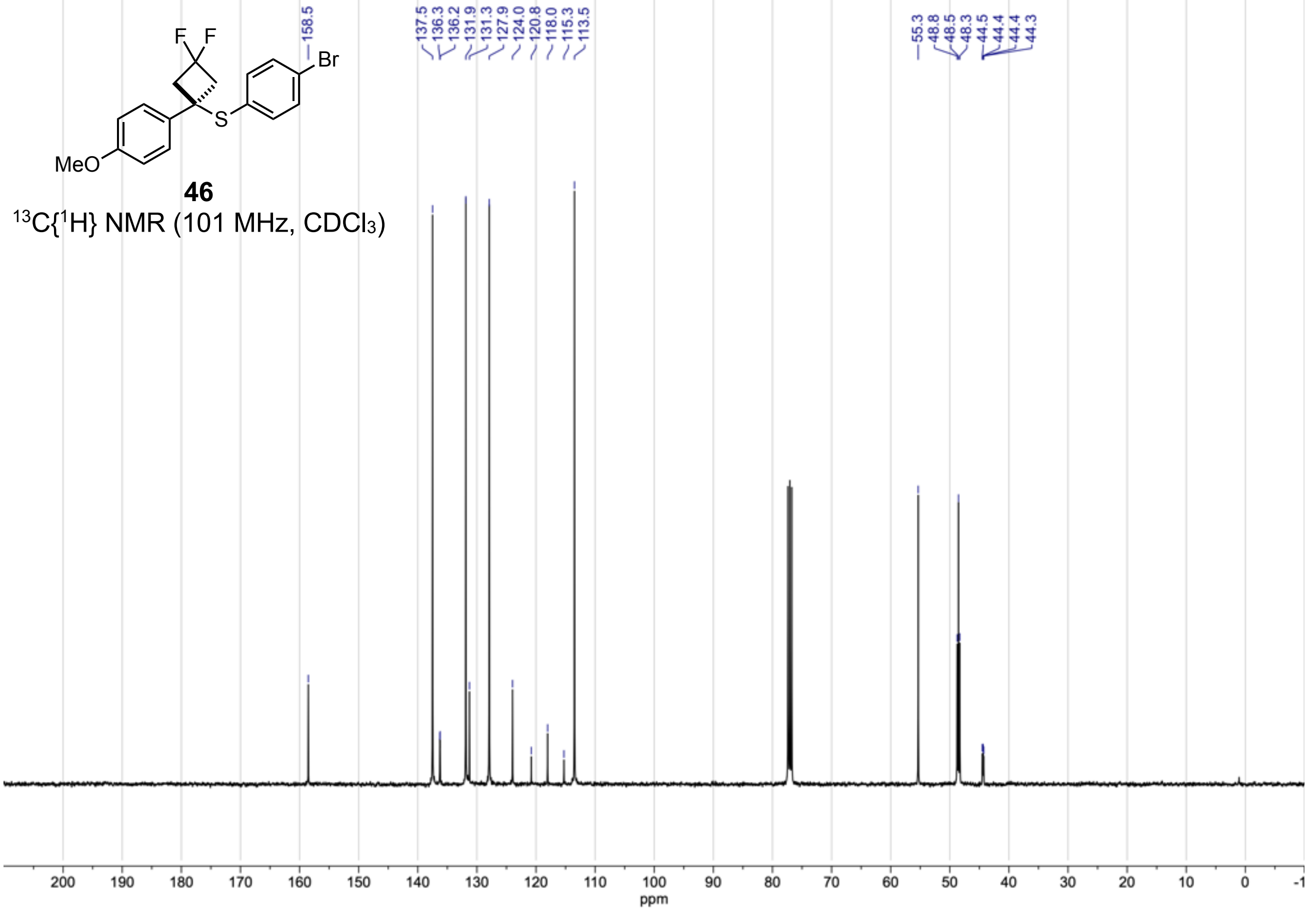
F
F
Br
S
MeO
46
13C{1H} NMR (101 MHz, CDCl3)
158.5
137.5
136.3
136.2
131.9
131.3
127.9
124.0
120.8
118.0
115.3
113.5
55.3
48.8
48.5
48.3
44.5
44.4
44.4
44.3
ppm

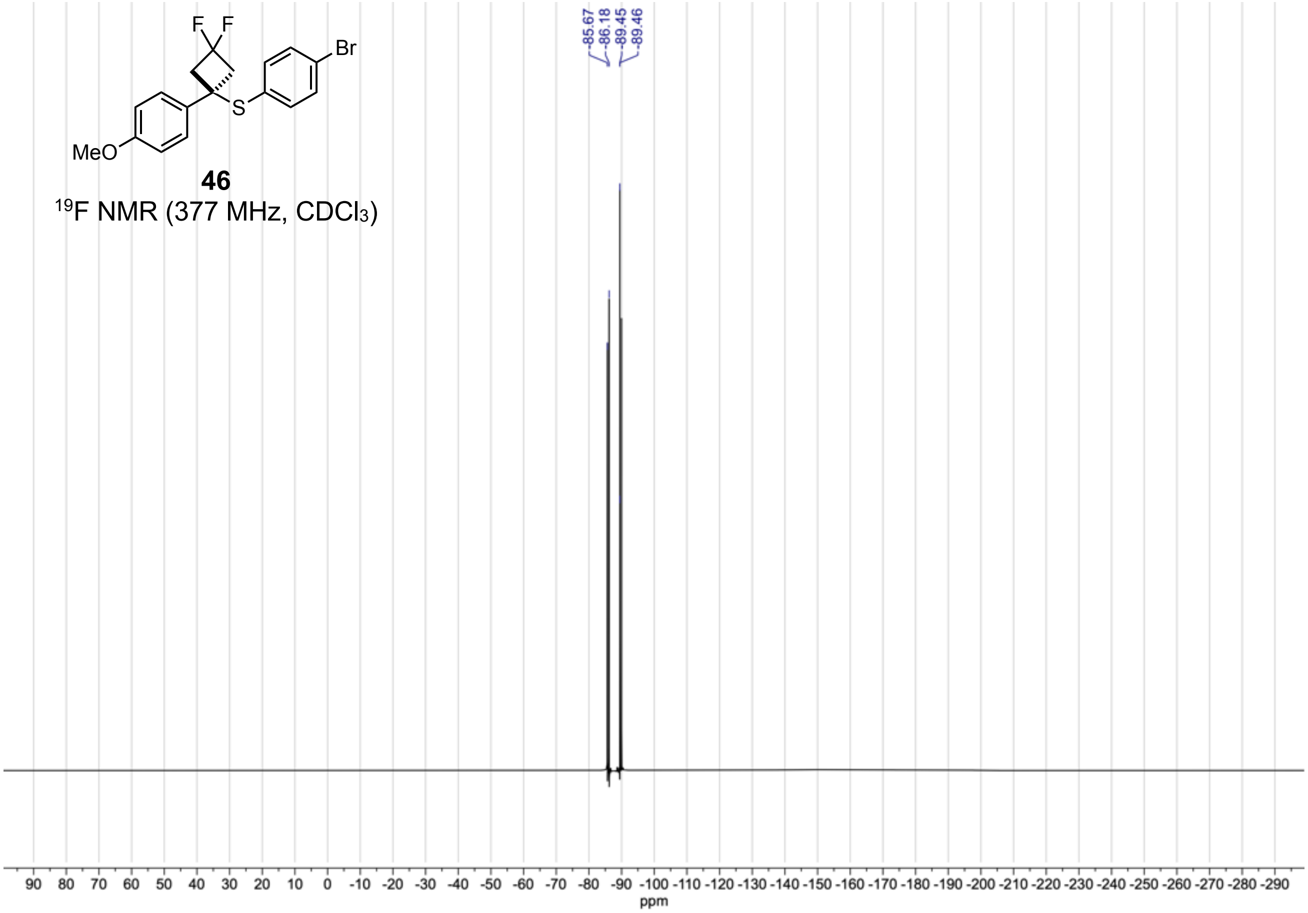

46

^{19}F NMR (377 MHz, $CDCl_3$)

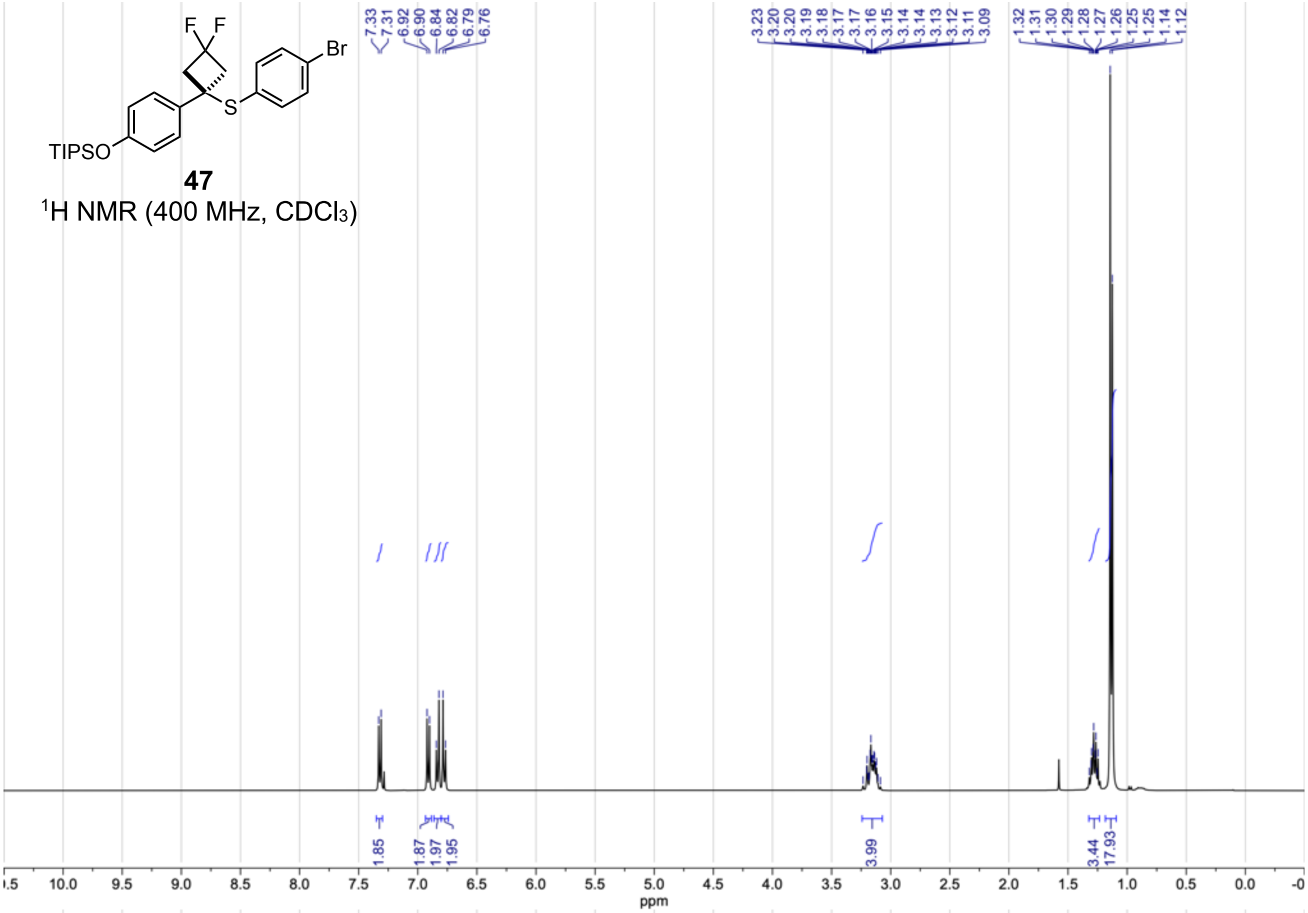
F F
Br
S
TIPSO
47
1H NMR (400 MHz, CDCl3)
7.33
7.31
6.92
6.90
6.84
6.82
6.79
6.76
3.23
3.20
3.20
3.19
3.18
3.17
3.17
3.16
3.15
3.14
3.14
3.13
3.12
3.11
3.09
1.32
1.31
1.30
1.29
1.28
1.27
1.26
1.25
1.25
1.14
1.12
1.85
1.87
1.97
1.95
3.99
3.44
17.93
10.0 9.5 9.0 8.5 8.0 7.5 7.0 6.5 6.0 5.5 5.0 4.5 4.0 3.5 3.0 2.5 2.0 1.5 1.0 0.5 0.0
ppm

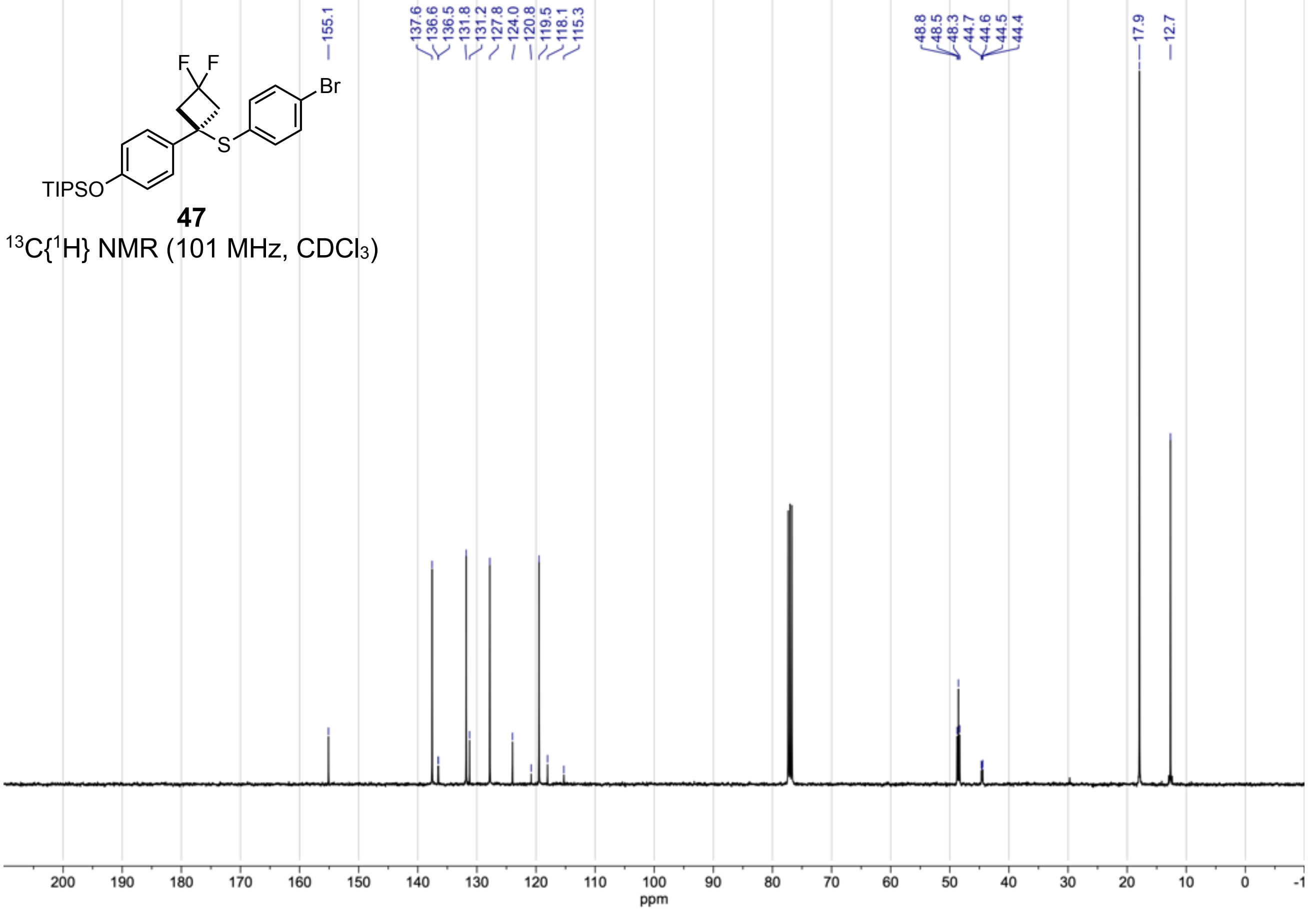
F F
Br
S
TIPSO
47
13C{1H} NMR (101 MHz, CDCl3)
155.1
137.6
136.6
136.5
131.8
131.2
127.8
124.0
120.8
119.5
118.1
115.3
48.8
48.5
48.3
44.7
44.6
44.5
44.4
17.9
12.7
200 190 180 170 160 150 140 130 120 110 100 90 80 70 60 50 40 30 20 10 0
ppm

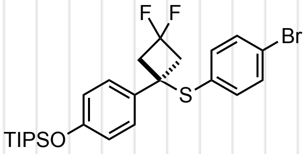

47

^{19}F NMR (377 MHz, $CDCl_3$)

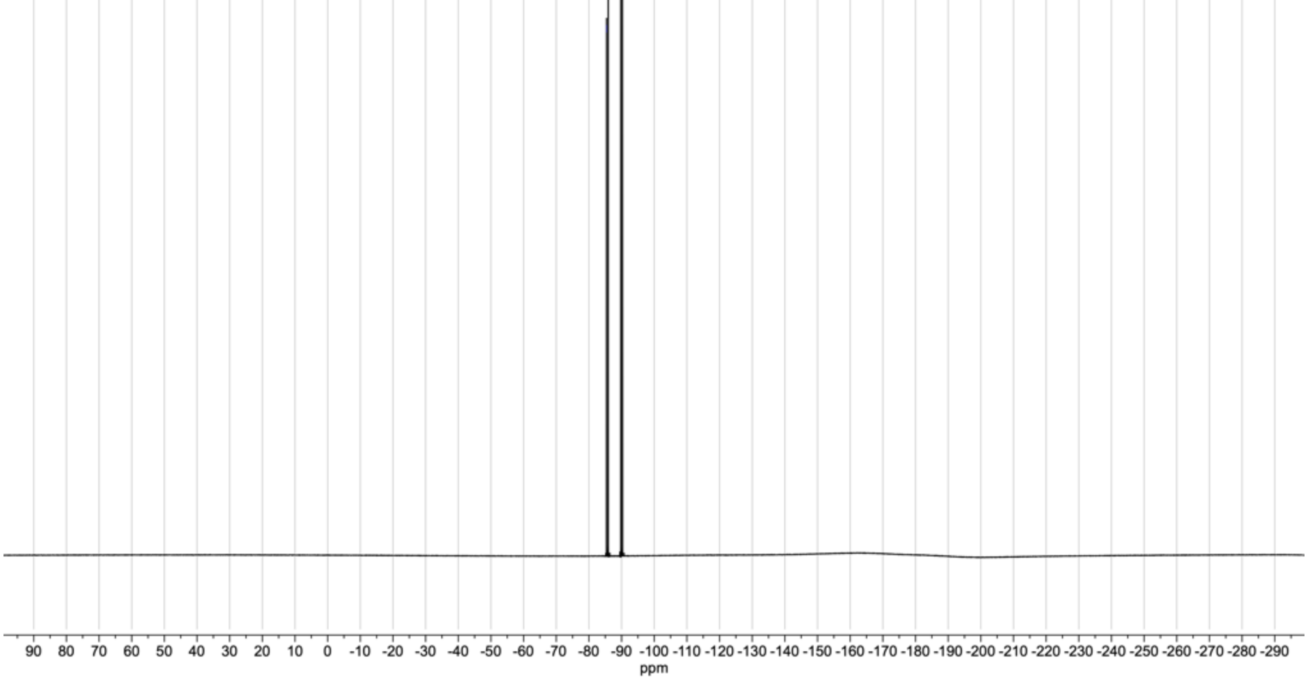

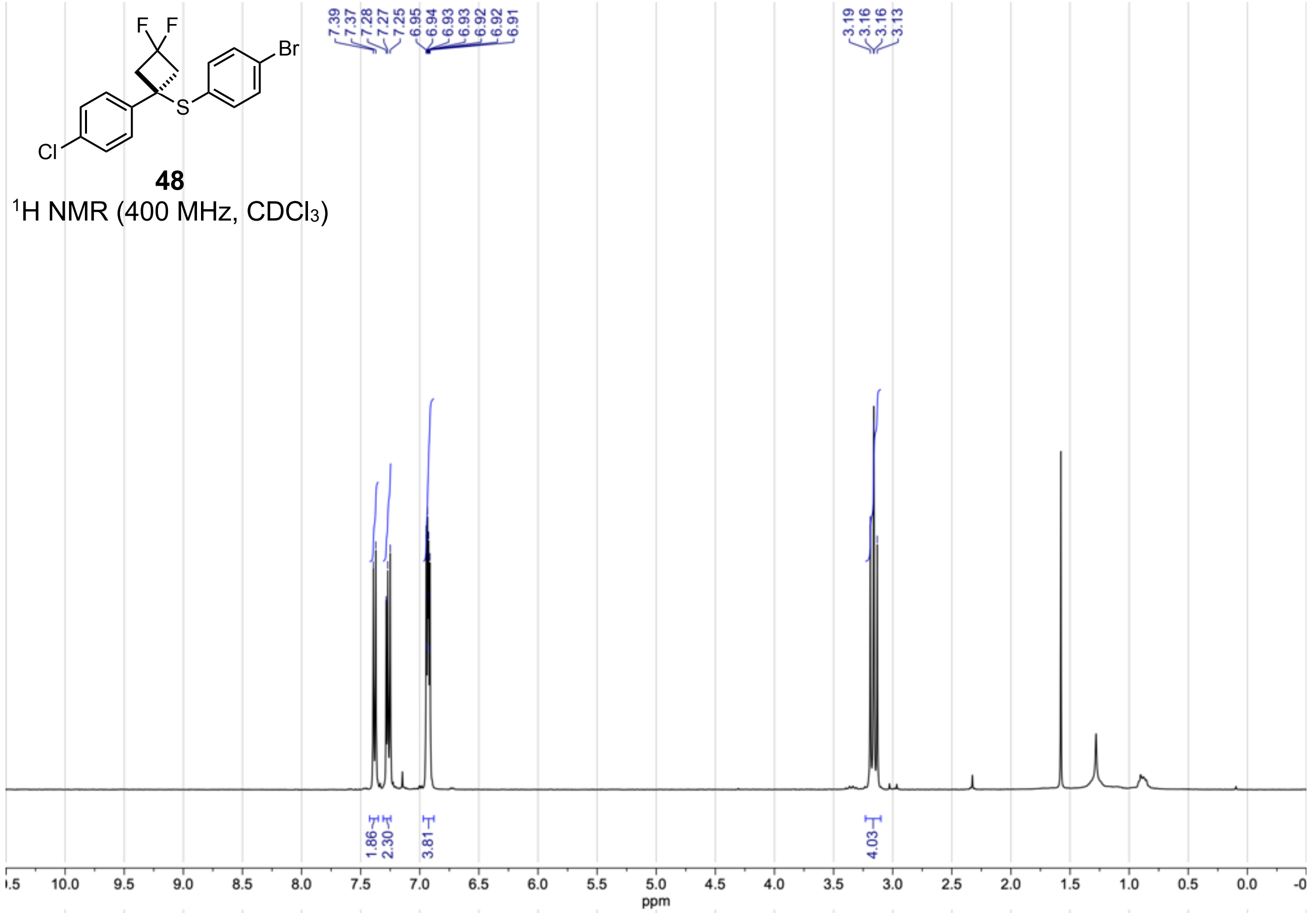

48
^{1}H NMR (400 MHz, $CDCl_3$)

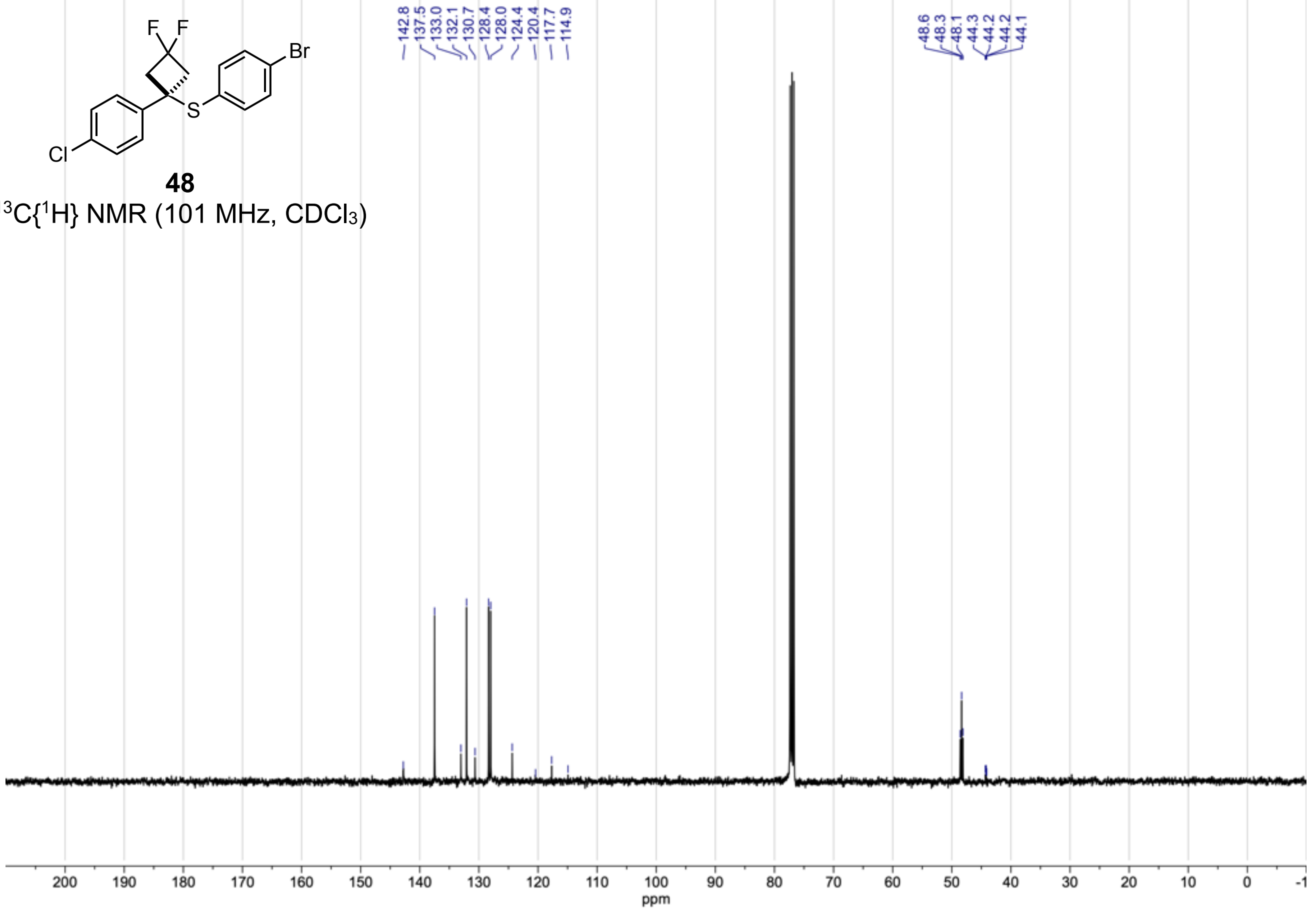

48
$^{13}C\{^1H\}$ NMR (101 MHz, $CDCl_3$)

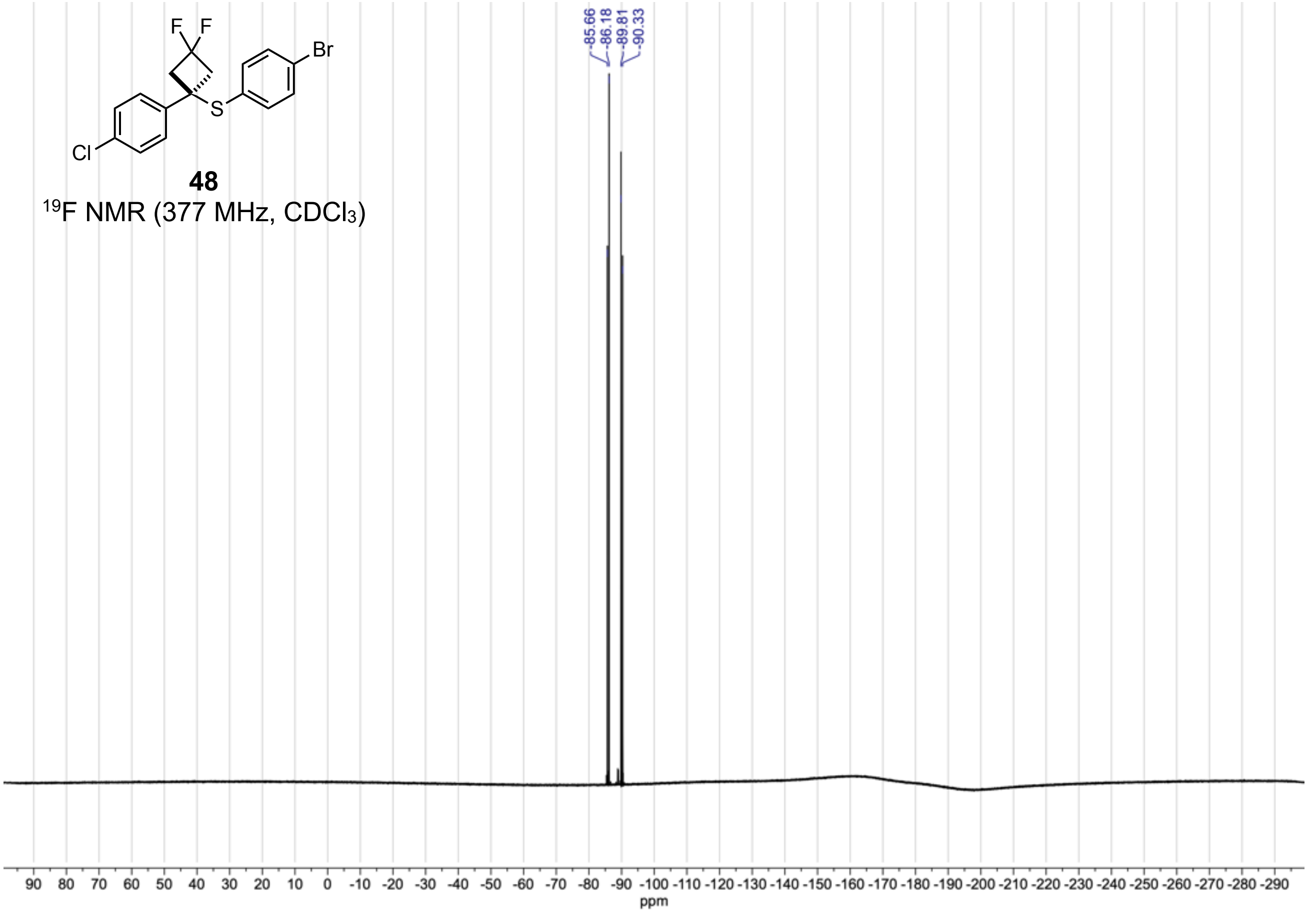

48

^{19}F NMR (377 MHz, $CDCl_3$)

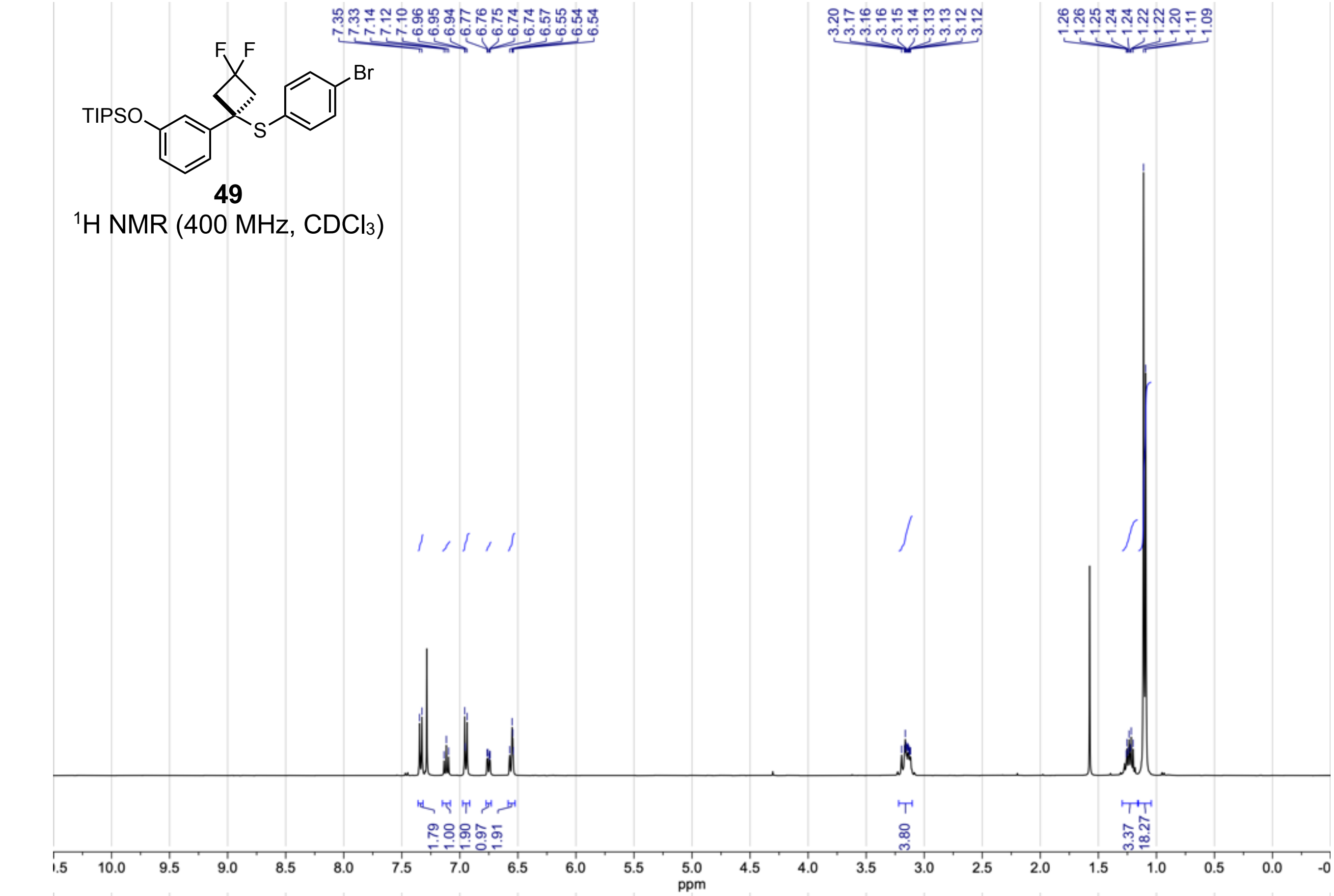
TIPSO
F
F
S
Br
49
^{1}H NMR (400 MHz, CDCl$_3$)
7.35 7.33 7.14 7.12 7.10 6.96 6.95 6.94 6.77 6.76 6.75 6.74 6.74 6.57 6.55 6.54 6.54
3.20 3.17 3.16 3.16 3.15 3.14 3.13 3.13 3.12 3.12
1.26 1.26 1.25 1.24 1.24 1.22 1.22 1.20 1.11 1.09
1.79 1.00 1.90 0.97 1.91 3.80 3.37 18.27
ppm

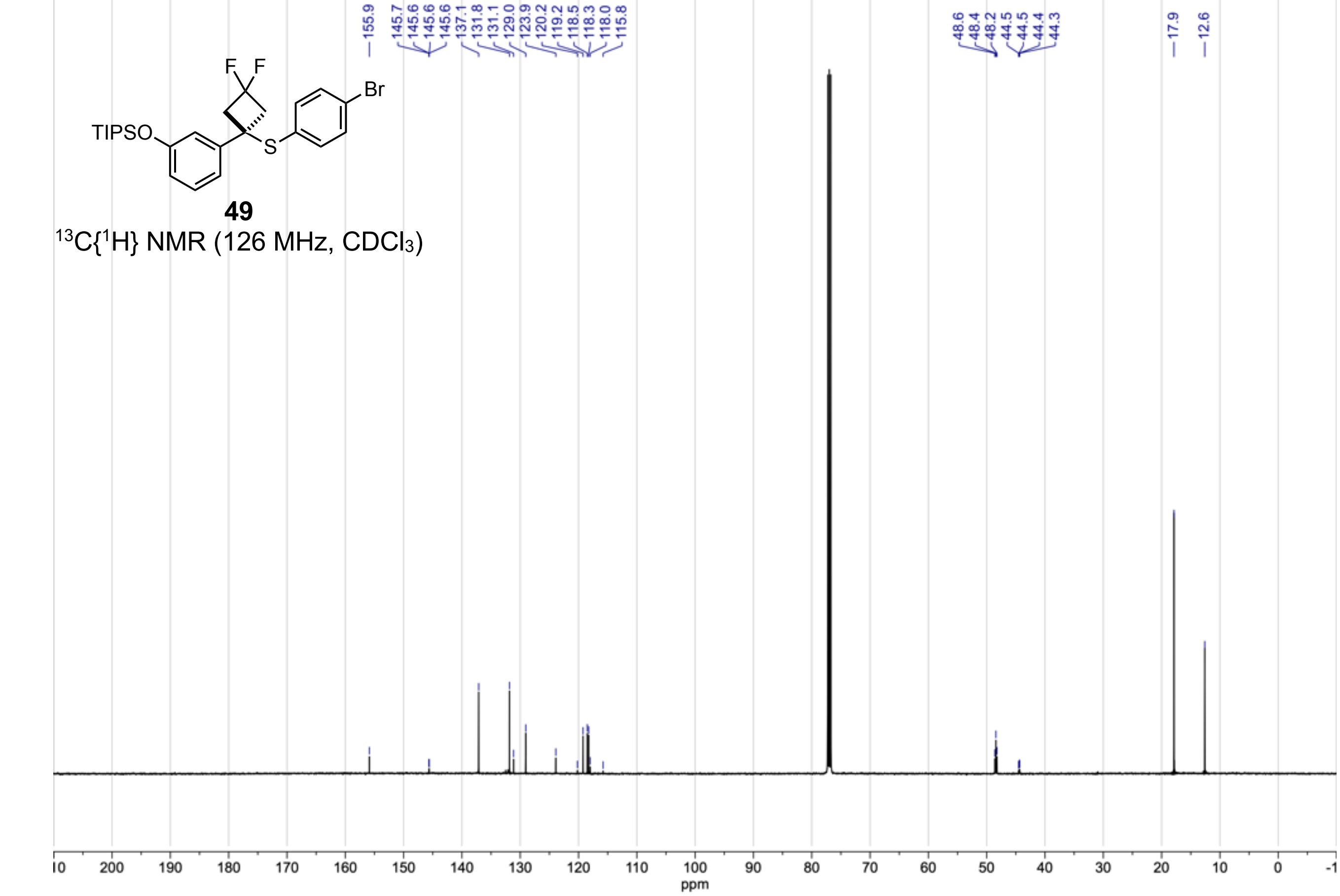
TIPSO
F
F
S
Br
49
^{13}C{^{1}H} NMR (126 MHz, CDCl$_3$)
155.9 145.7 145.6 145.6 145.6 137.1 131.8 131.1 129.0 123.9 120.2 119.2 118.5 118.3 118.0 115.8
48.6 48.4 48.2 44.5 44.4 44.3
17.9 12.6
ppm

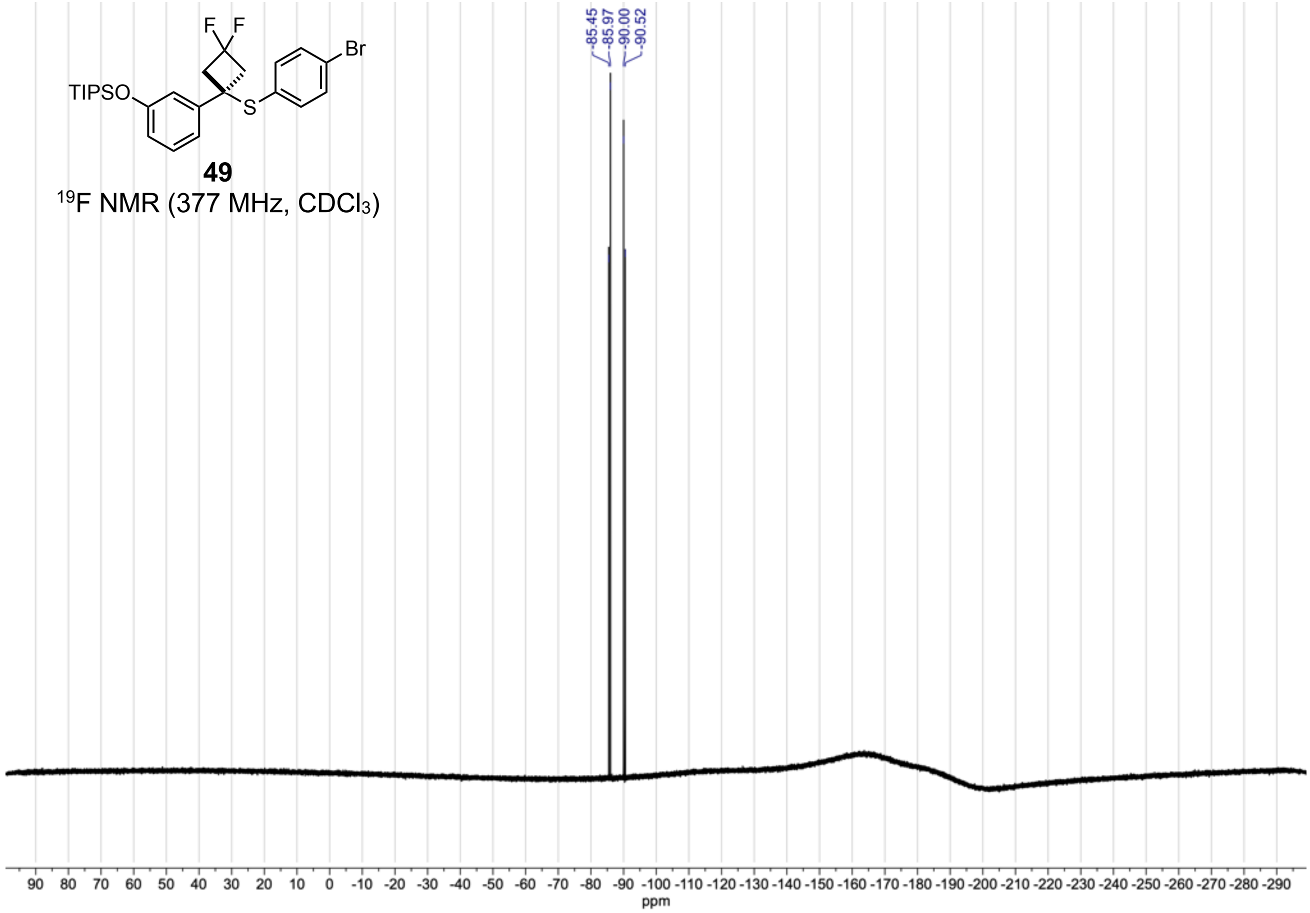
TIPSO
F
F
S
Br
49
19F NMR (377 MHz, CDCl3)
-85.45
-85.97
-90.00
-90.52
90 80 70 60 50 40 30 20 10 0 -10 -20 -30 -40 -50 -60 -70 -80 -90 -100 -110 -120 -130 -140 -150 -160 -170 -180 -190 -200 -210 -220 -230 -240 -250 -260 -270 -280 -290
ppm

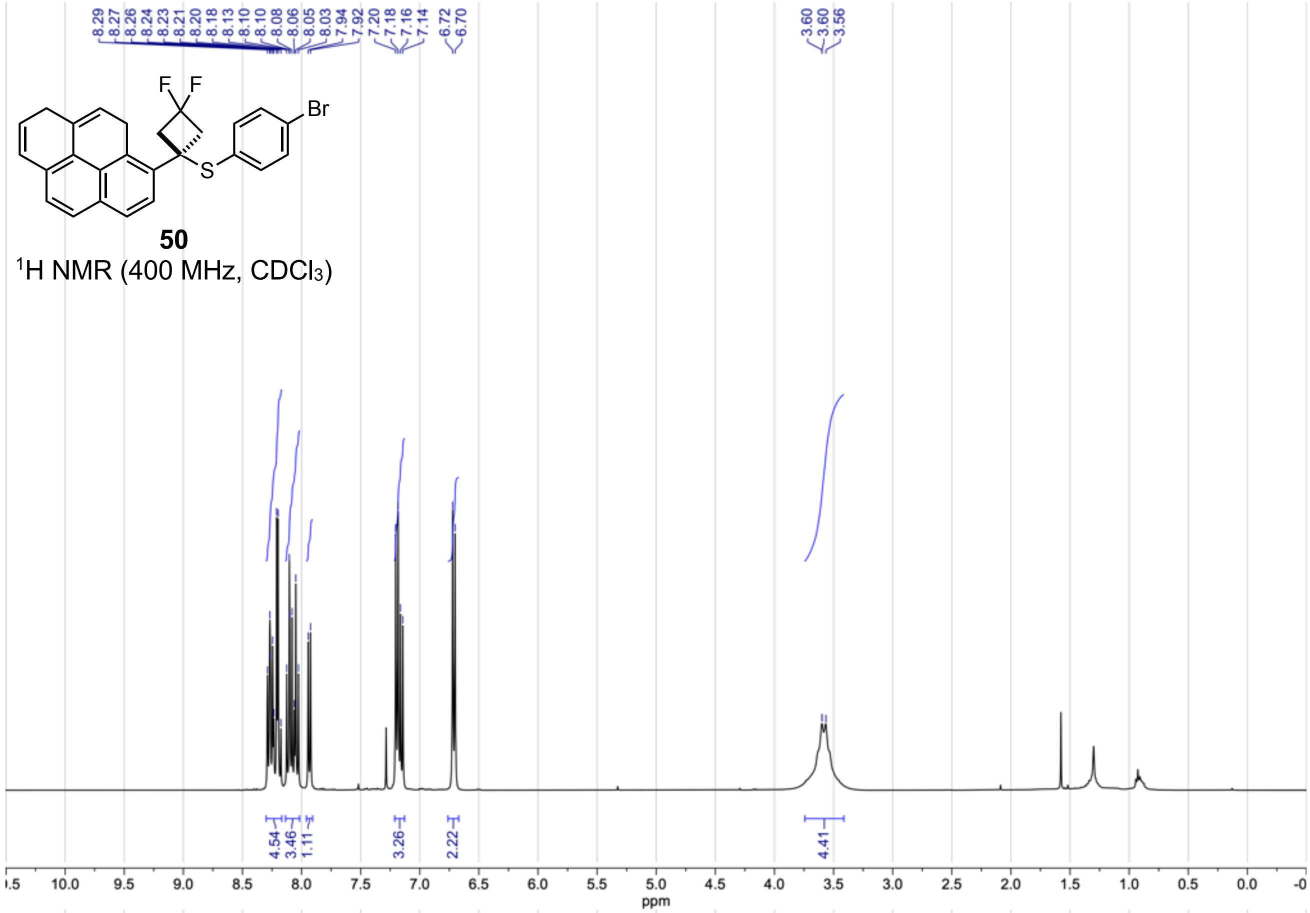

8.29 8.27 8.26 8.24 8.23 8.21 8.20 8.18 8.13 8.10 8.08 8.06 8.05 8.03 7.94 7.92 7.20 7.18 7.16 7.14 6.72 6.70
3.60 3.60 3.56
F F
Br
S
50
1H NMR (400 MHz, CDCl3)
4.54 3.46 1.11 3.26 2.22 4.41
10.0 9.5 9.0 8.5 8.0 7.5 7.0 6.5 6.0 5.5 5.0 4.5 4.0 3.5 3.0 2.5 2.0 1.5 1.0 0.5 0.0
ppm

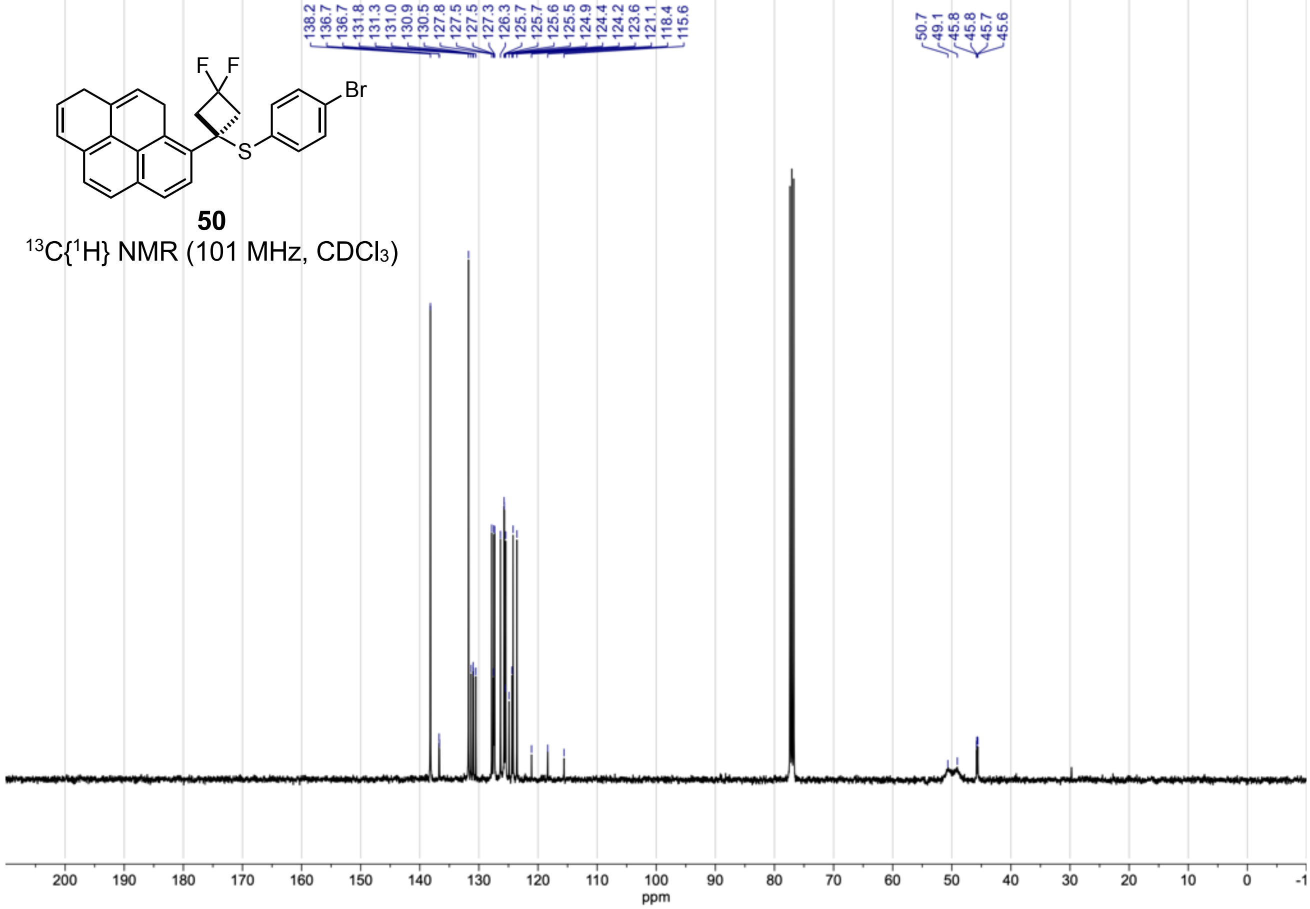

138.2 136.7 136.7 131.8 131.3 131.0 130.9 130.5 127.8 127.5 127.5 127.3 126.3 125.7 125.7 125.6 125.5 124.9 124.4 124.2 123.6 121.1 118.4 115.6
50.7 49.1 45.8 45.7 45.6
F F
Br
S
50
13C{1H} NMR (101 MHz, CDCl3)
200 190 180 170 160 150 140 130 120 110 100 90 80 70 60 50 40 30 20 10 0
ppm

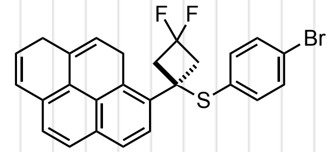

50

^{19}F NMR (377 MHz, $CDCl_3$)

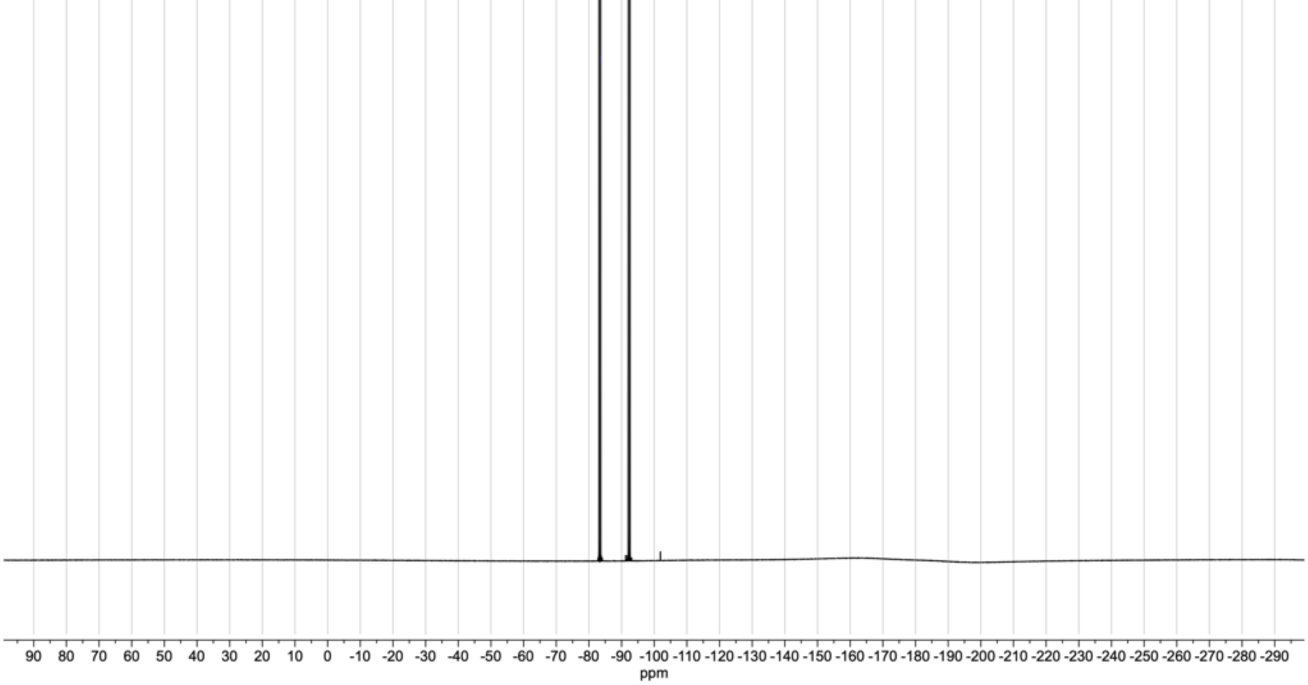

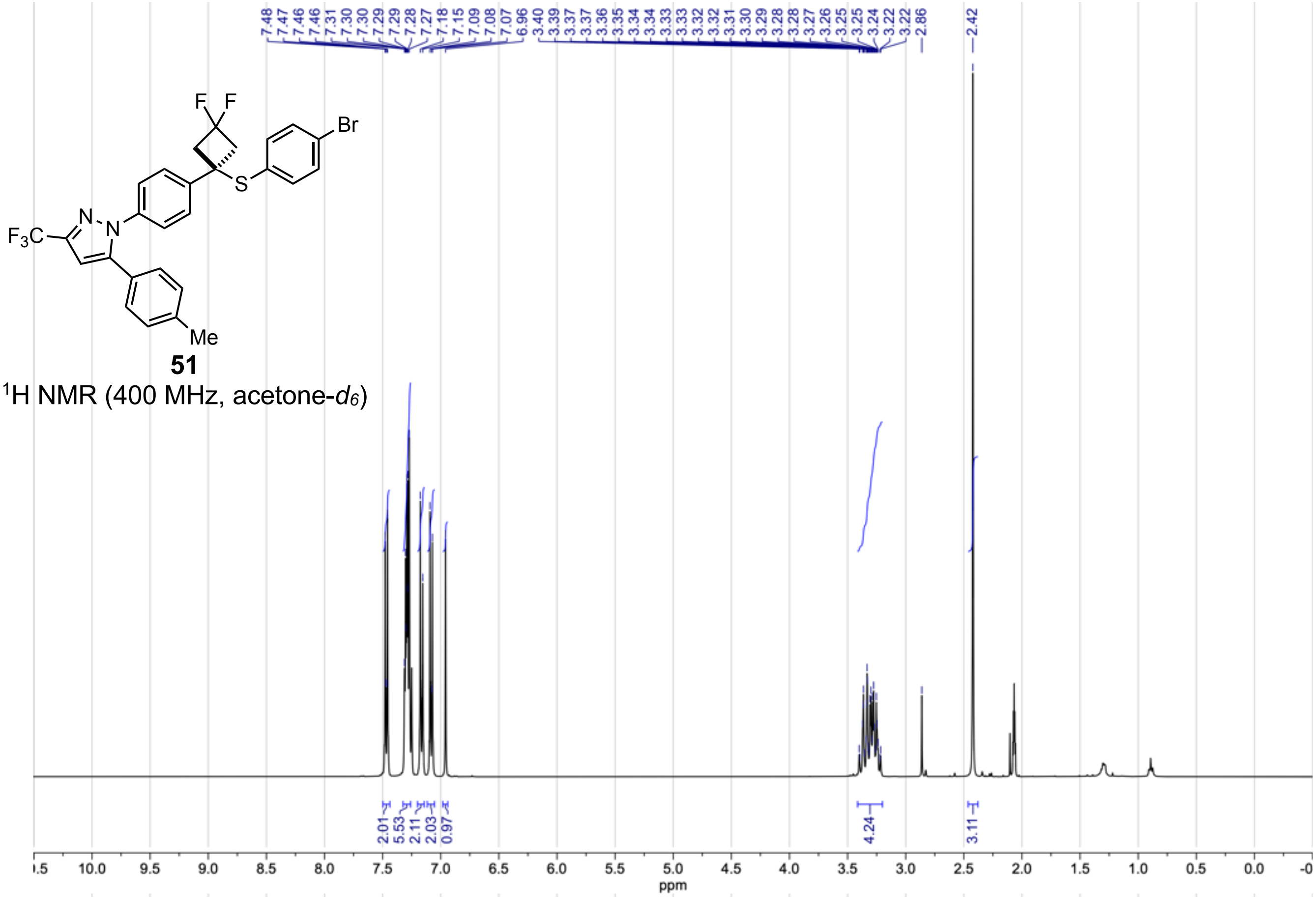

51

^{1}H NMR (400 MHz, acetone-d_6)

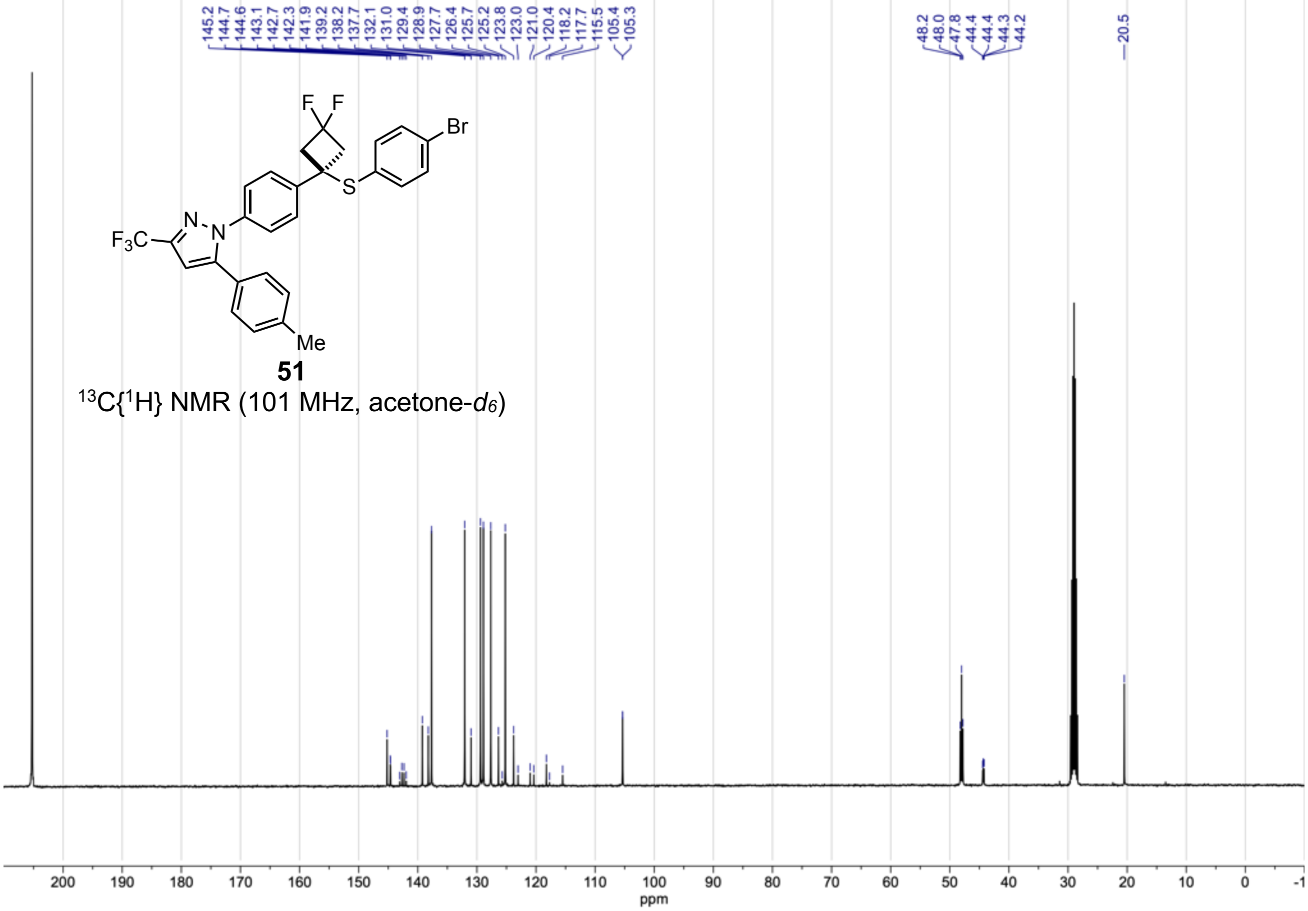

51

^{13}C{^{1}H} NMR (101 MHz, acetone-d_6)

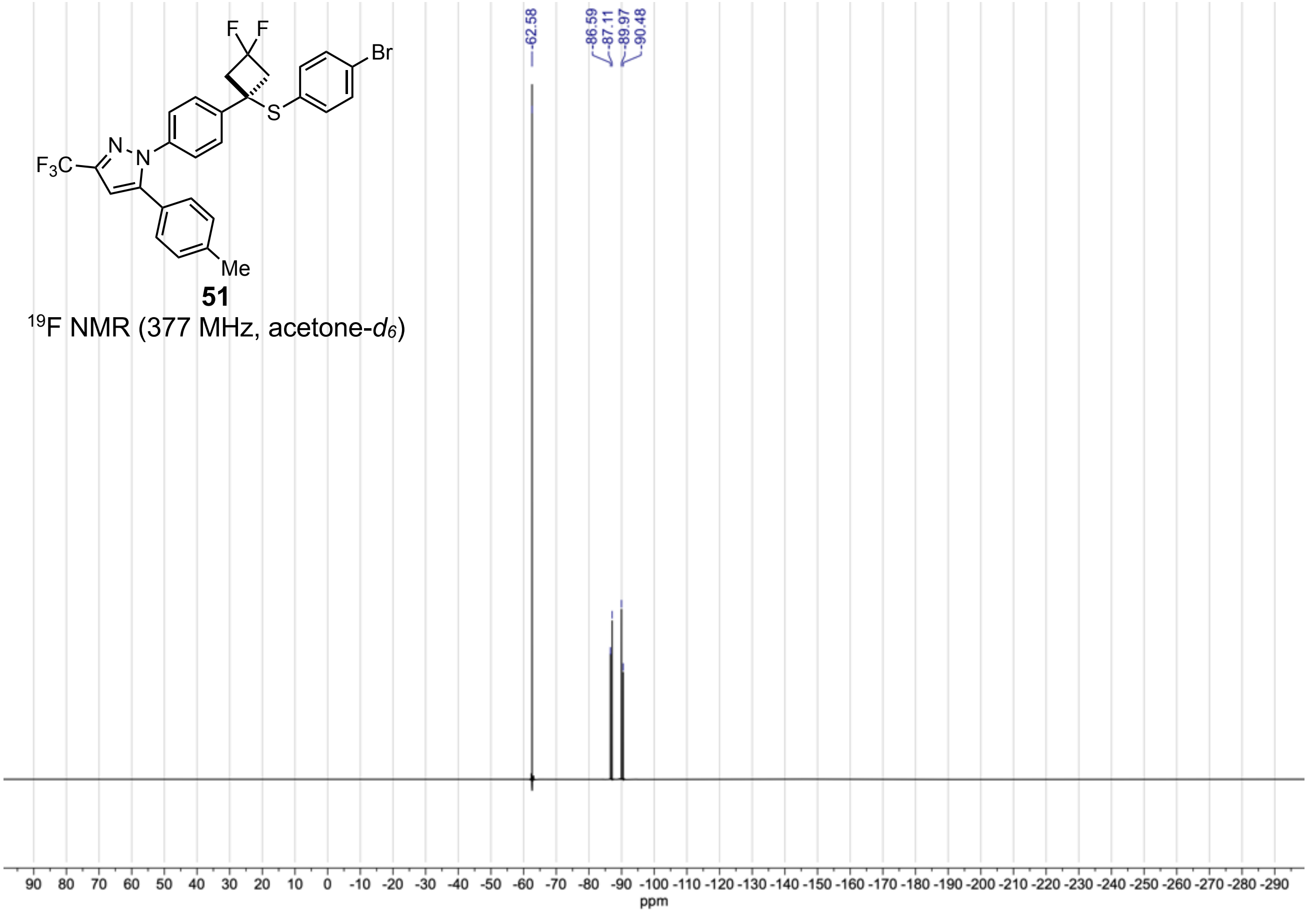

^{19}F NMR (377 MHz, acetone-d_6)

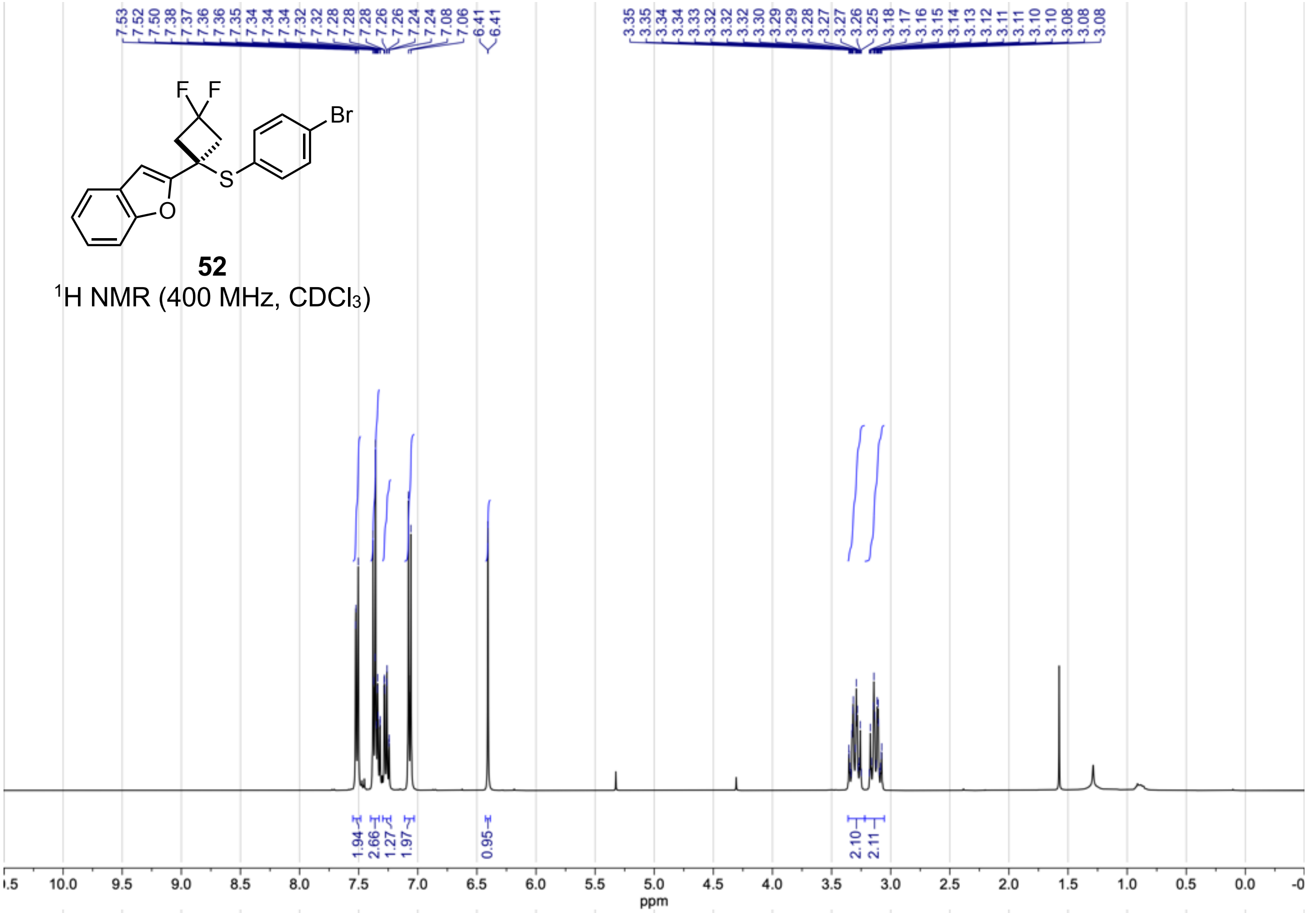

52
^{1}H NMR (400 MHz, CDCl$_3$)

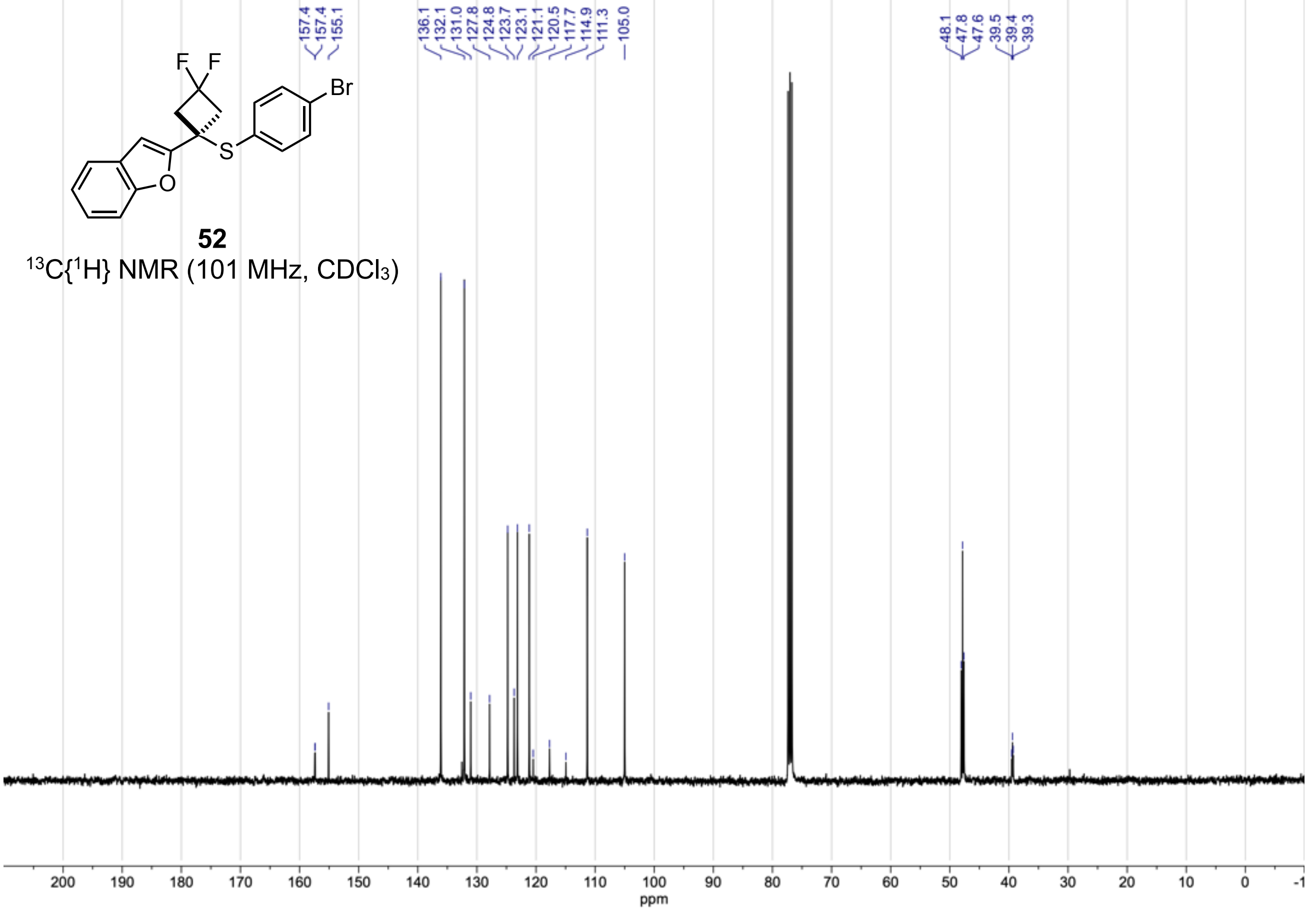

52
^{13}C{^{1}H} NMR (101 MHz, CDCl$_3$)

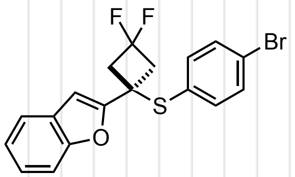

52

^{19}F NMR (377 MHz, $CDCl_3$)

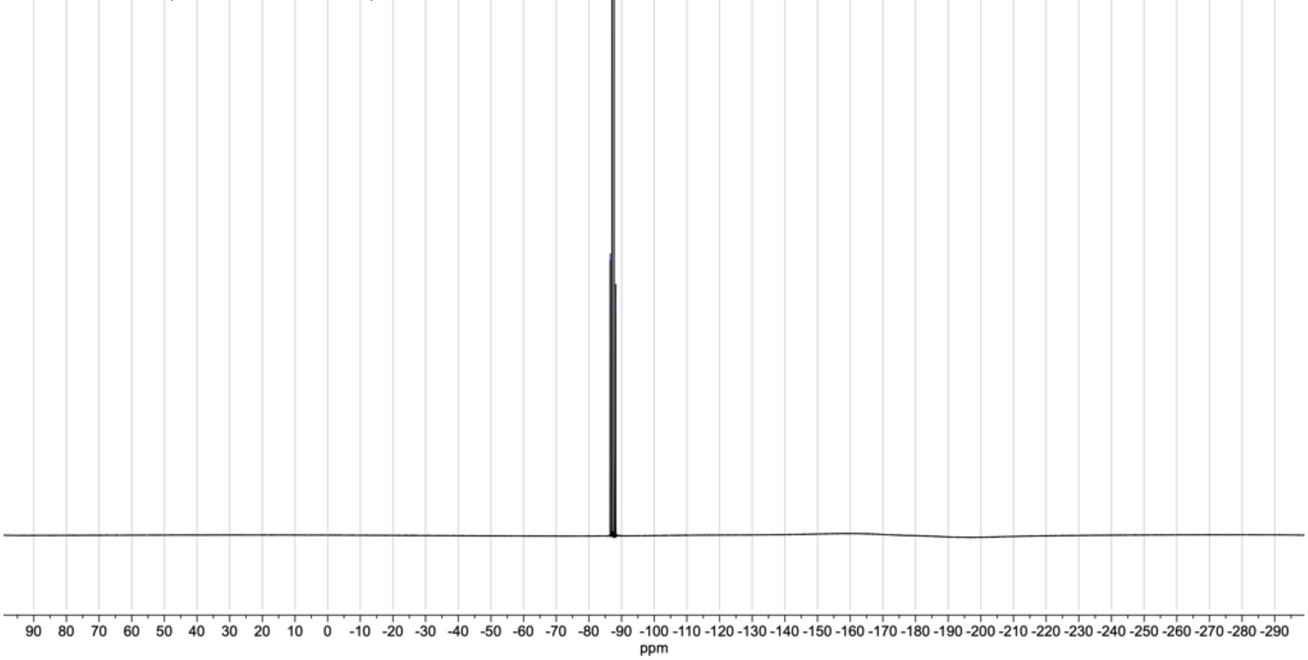

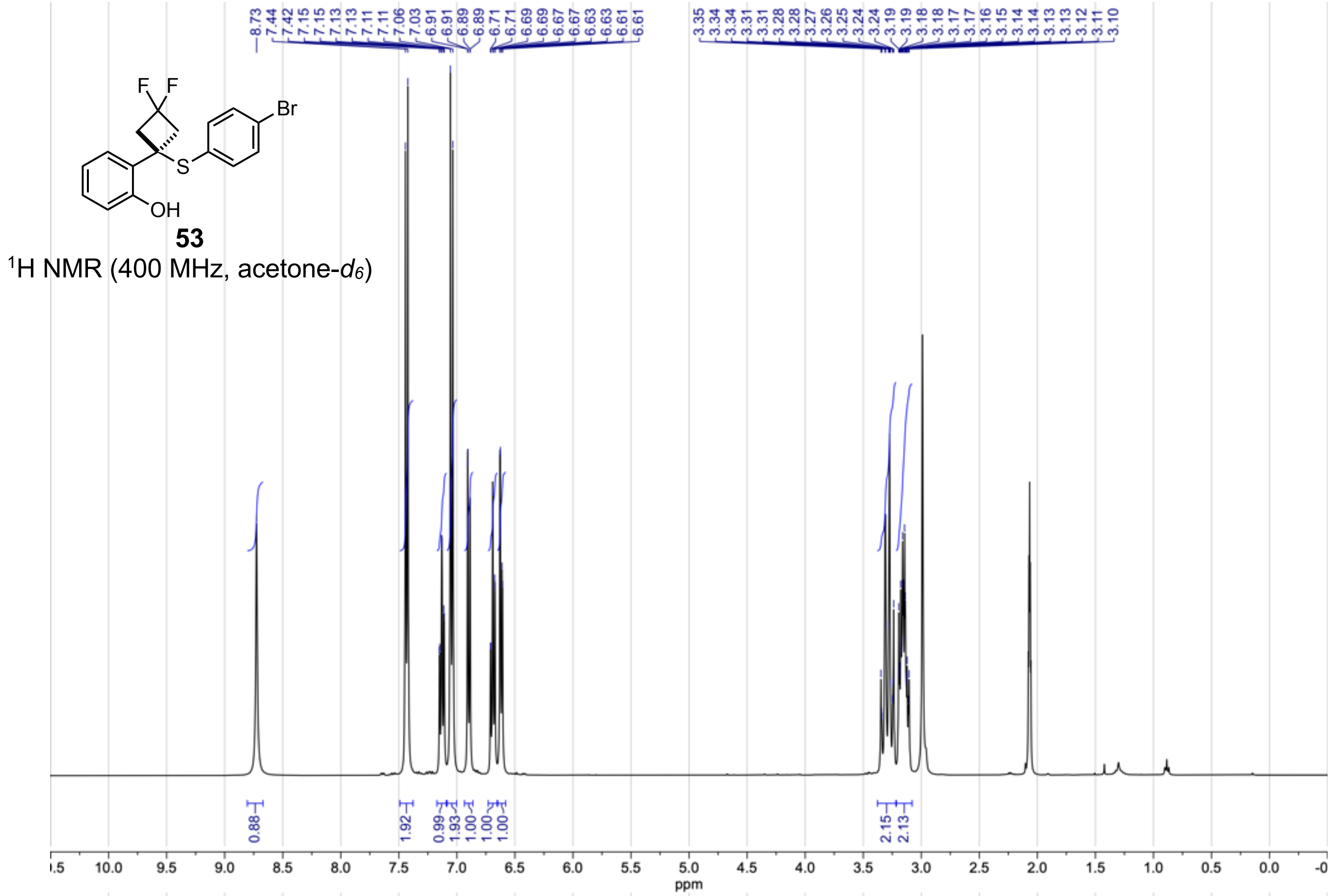

53
1H NMR (400 MHz, acetone-d6)
ppm

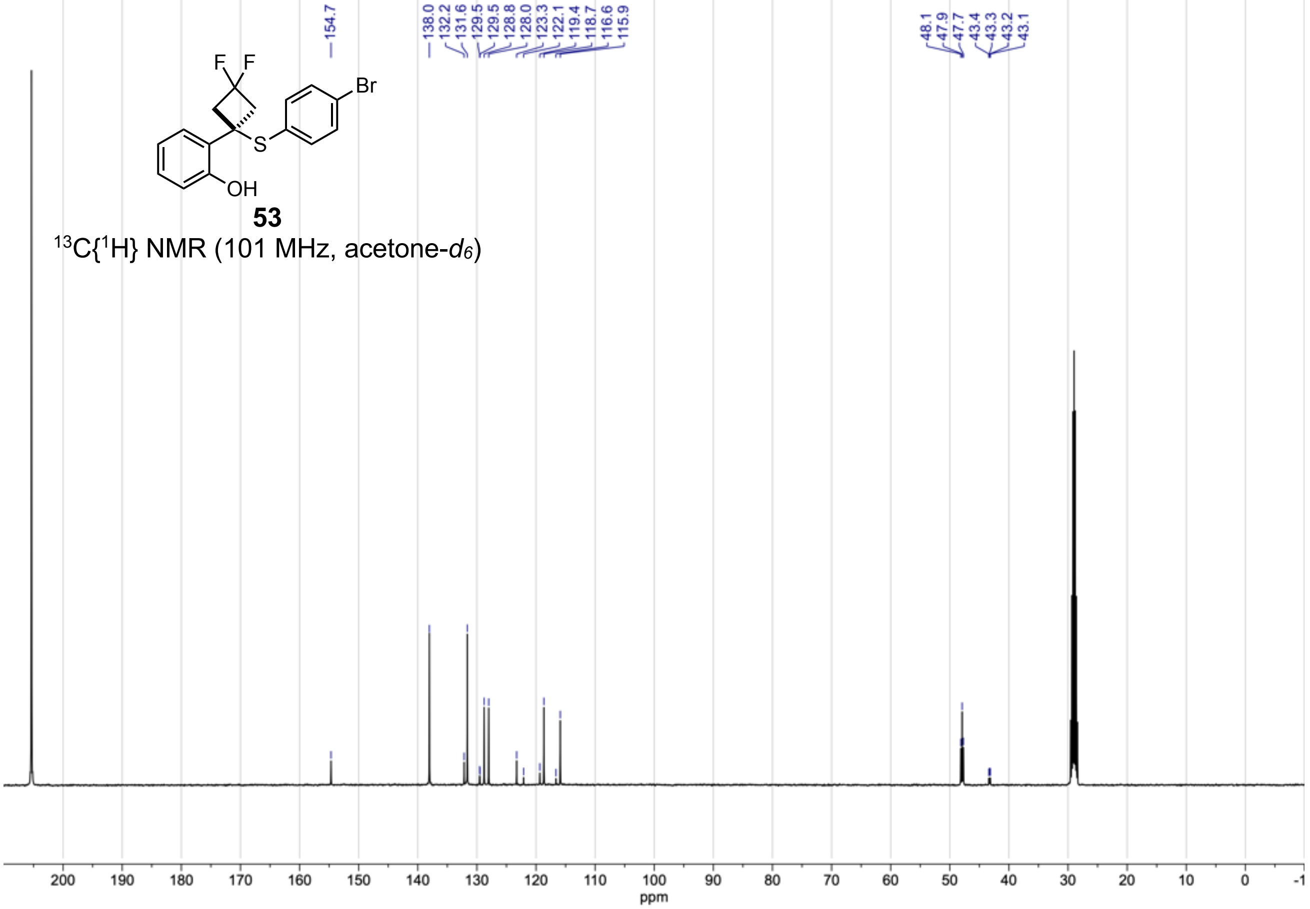

53
13C{1H} NMR (101 MHz, acetone-d6)
ppm

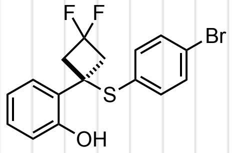

53

^{19}F NMR (377 MHz, acetone-d_6)

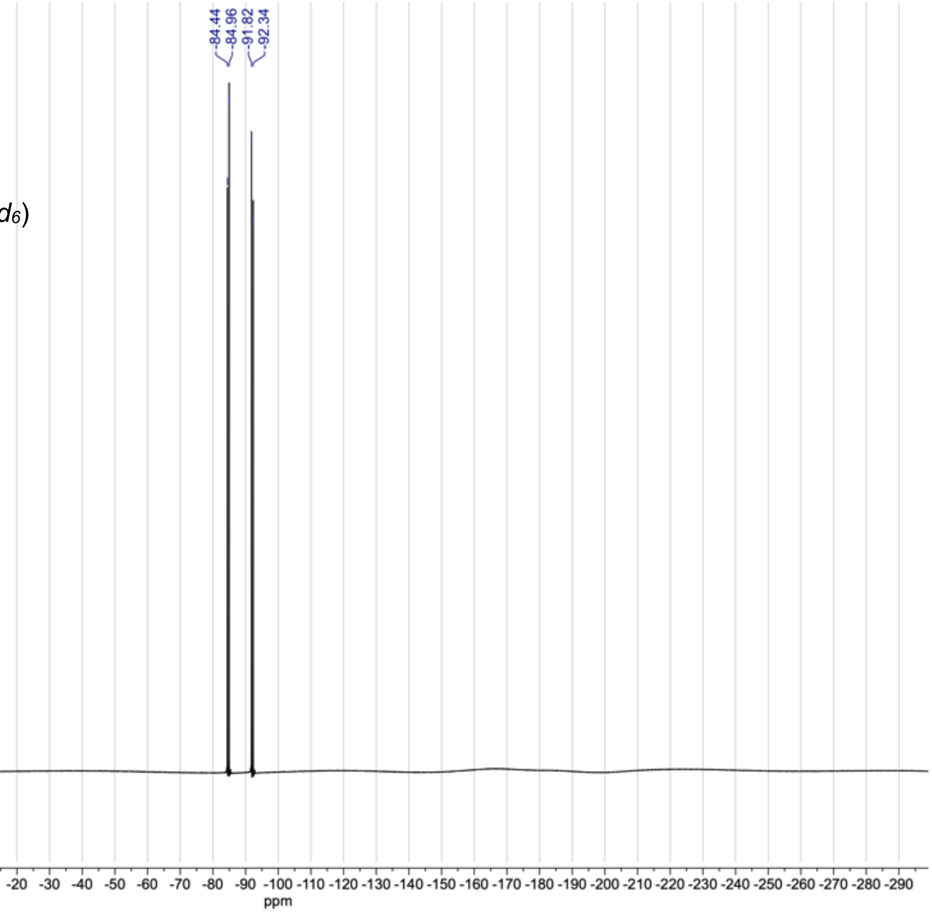

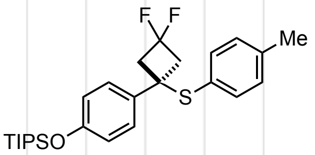

54

^{1}H NMR (400 MHz, $CDCl_3$)

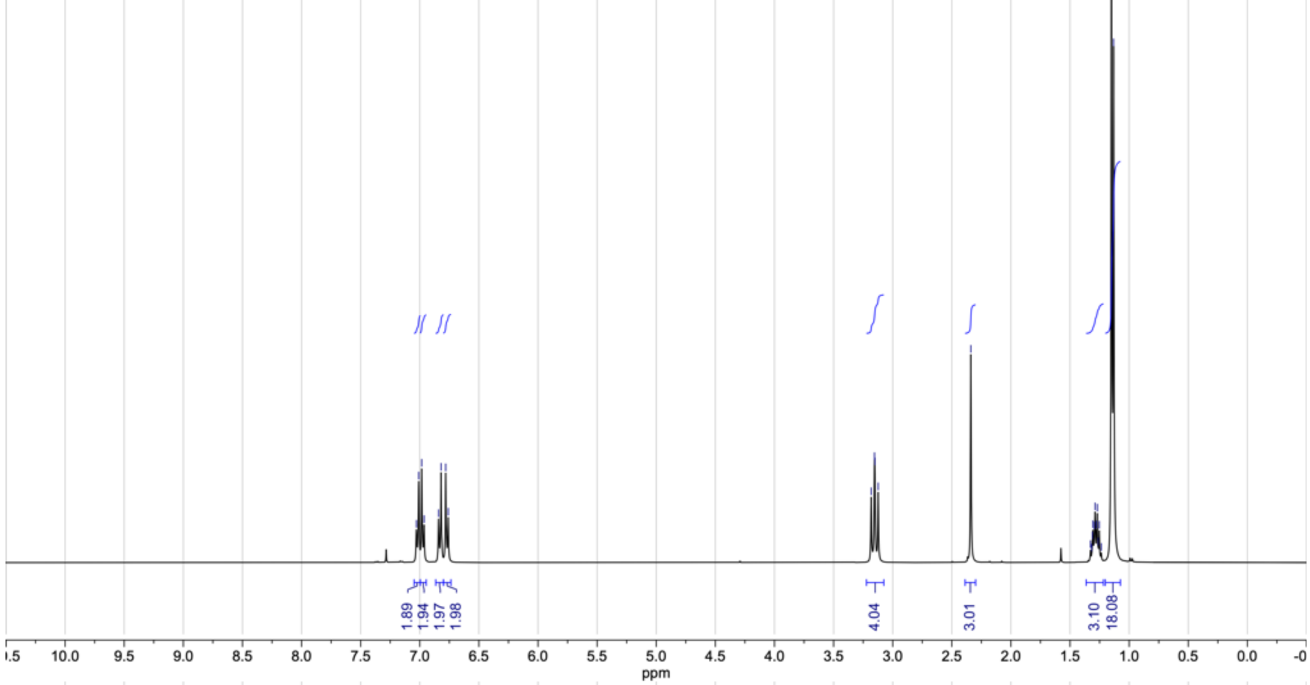

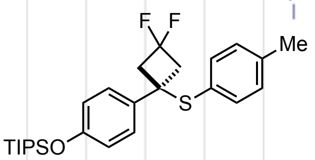

54

$^{13}C\{^{1}H\}$ NMR (101 MHz, $CDCl_3$)

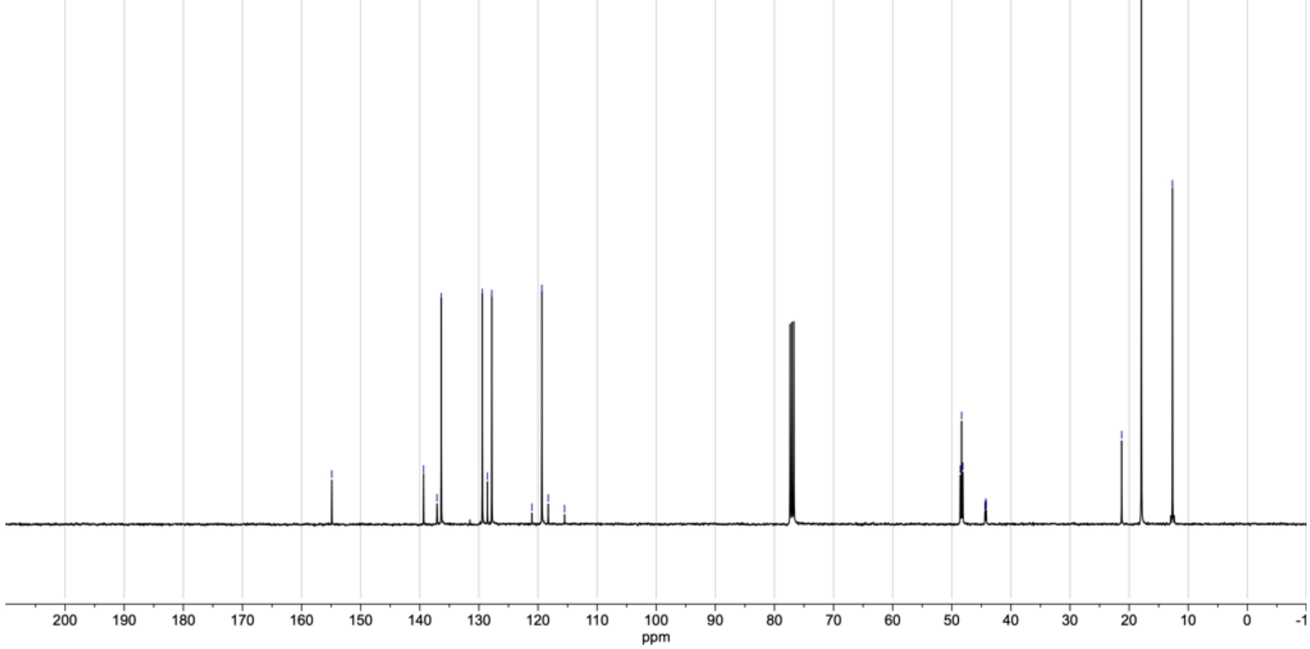

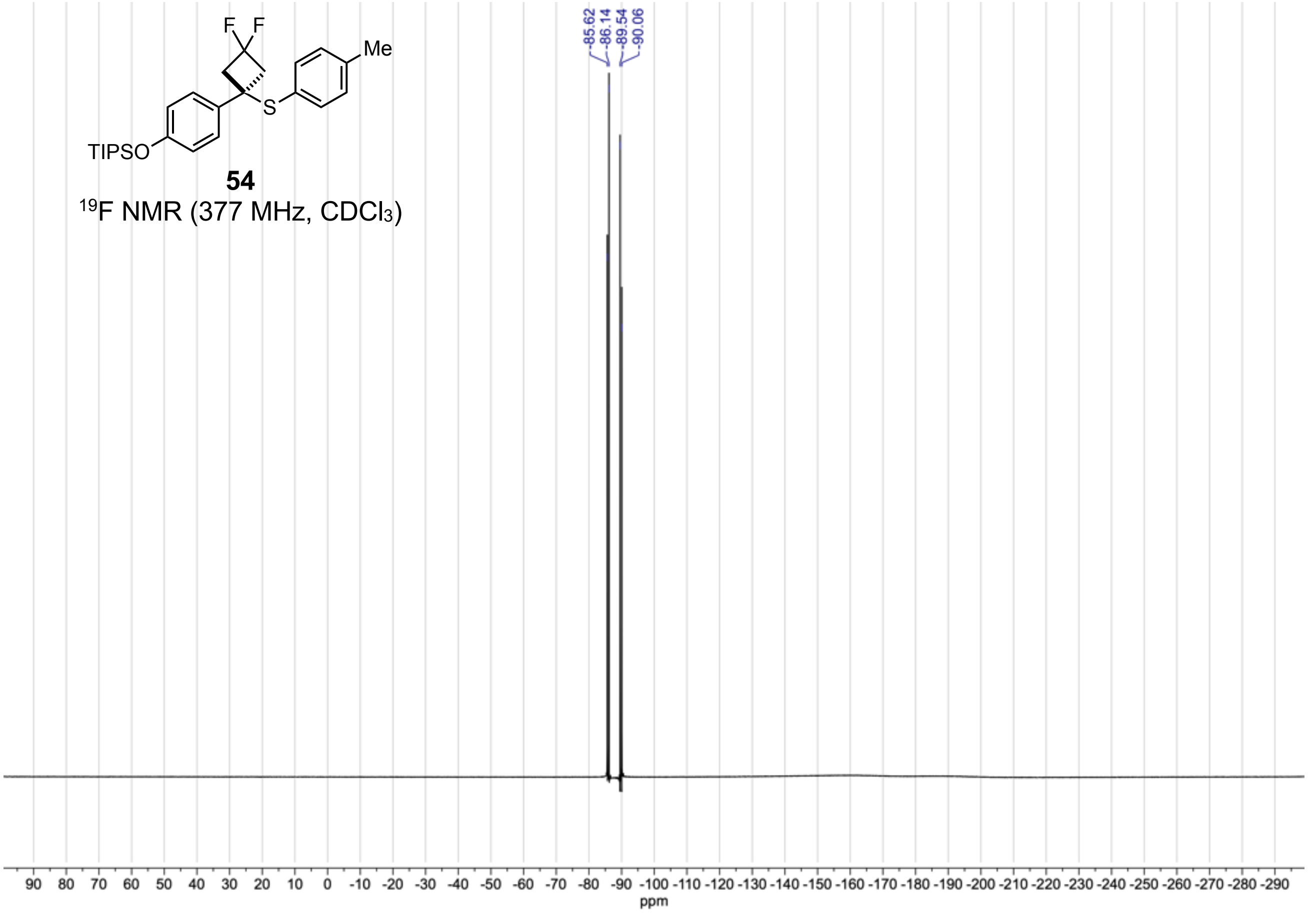

54

^{19}F NMR (377 MHz, $CDCl_3$)

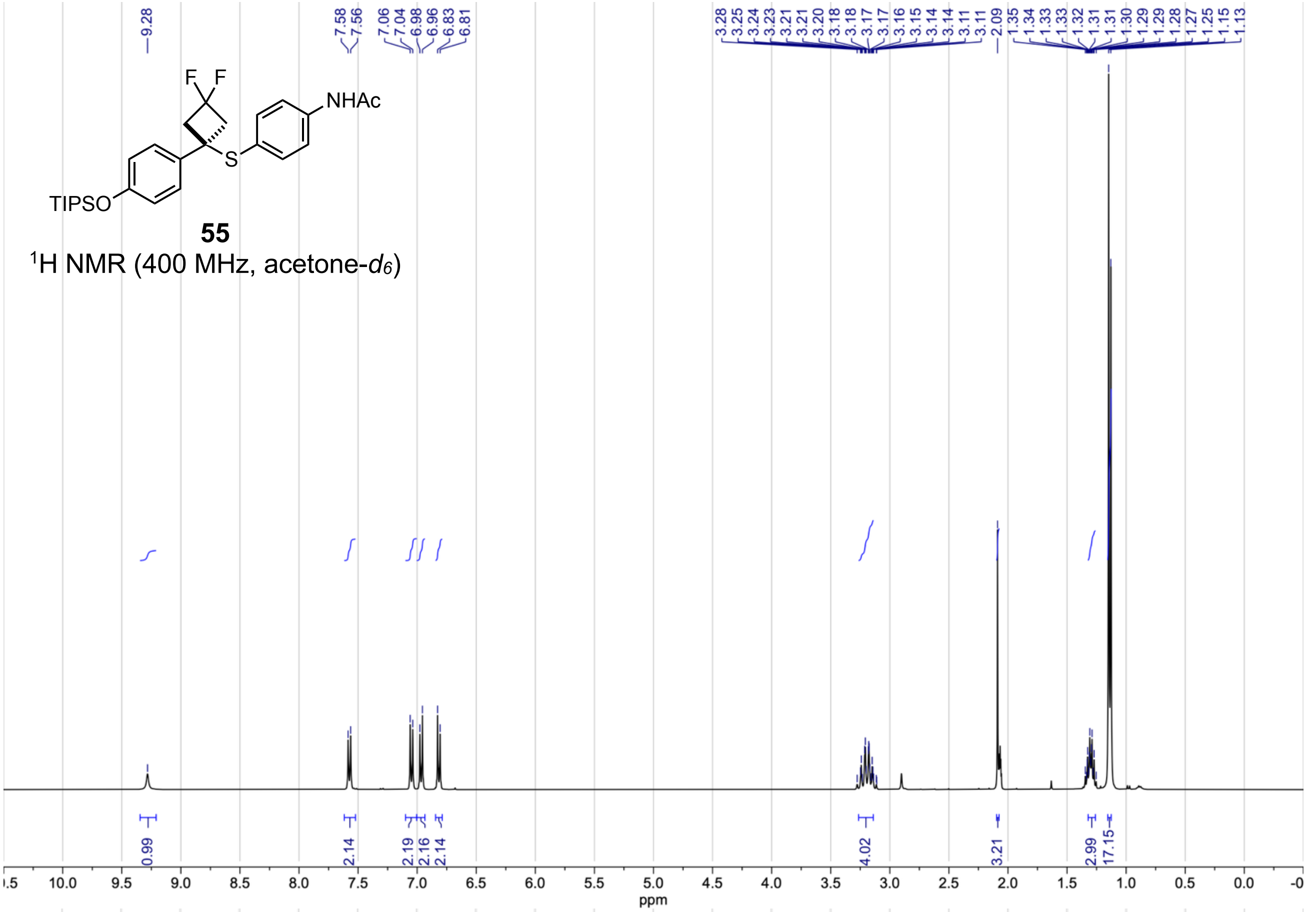
9.28
7.58
7.56
7.06
7.04
6.98
6.96
6.83
6.81
3.28
3.25
3.24
3.23
3.21
3.21
3.20
3.18
3.18
3.17
3.17
3.16
3.15
3.14
3.14
3.11
3.11
2.09
1.35
1.34
1.33
1.33
1.32
1.31
1.31
1.30
1.29
1.29
1.28
1.27
1.25
1.15
1.13
F
F
NHAc
S
TIPSO
55
1H NMR (400 MHz, acetone-d6)
0.99
2.14
2.19
2.16
2.14
4.02
3.21
2.99
17.15
ppm

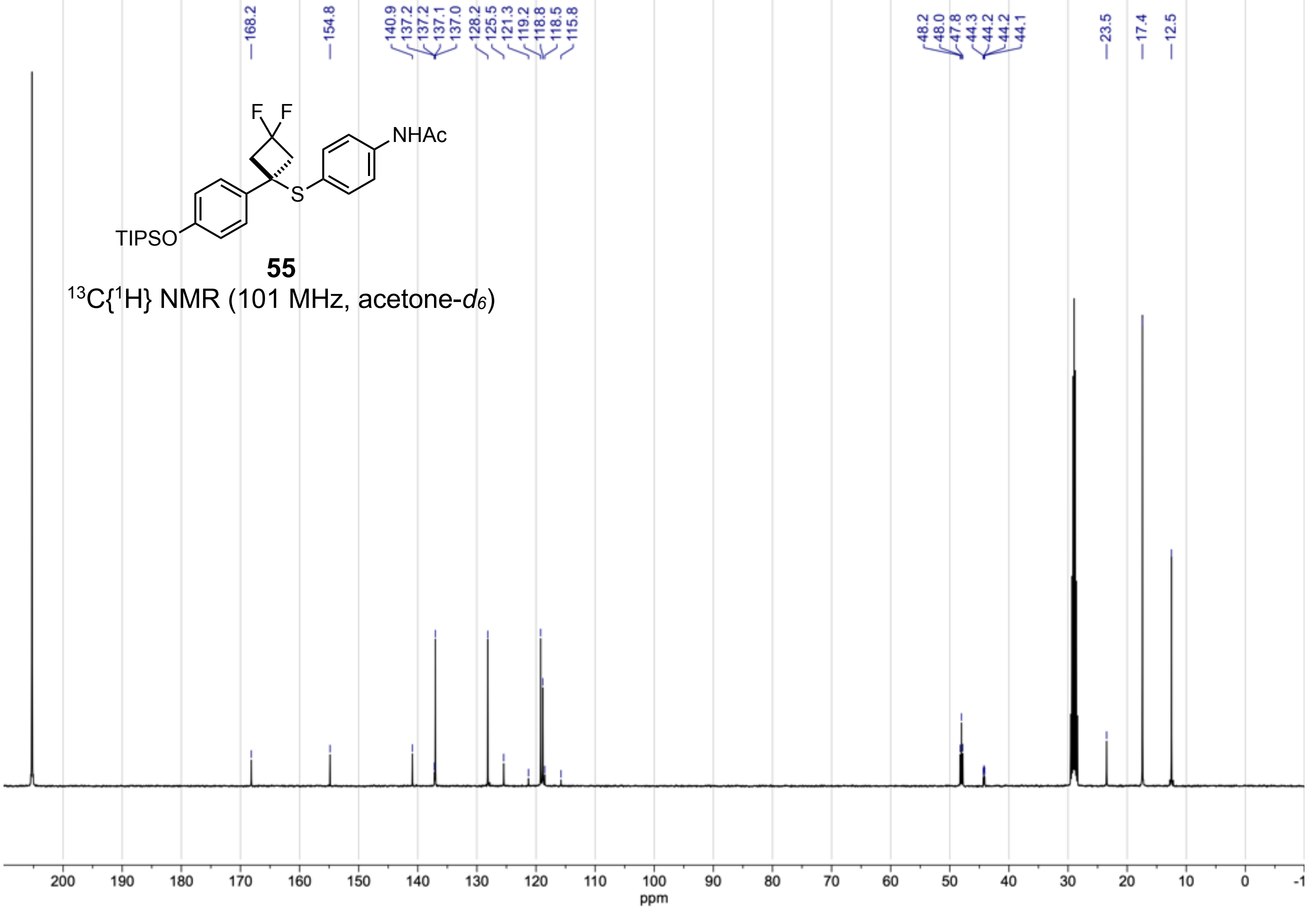
168.2
154.8
140.9
137.2
137.2
137.1
137.0
128.2
125.5
121.3
119.2
118.8
118.5
115.8
48.2
48.0
47.8
44.3
44.2
44.2
44.1
23.5
17.4
12.5
F
F
NHAc
S
TIPSO
55
13C{1H} NMR (101 MHz, acetone-d6)
ppm

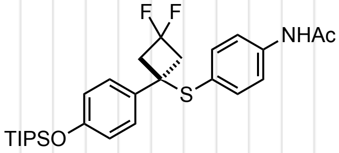

55

^{19}F NMR (377 MHz, acetone-d_6)

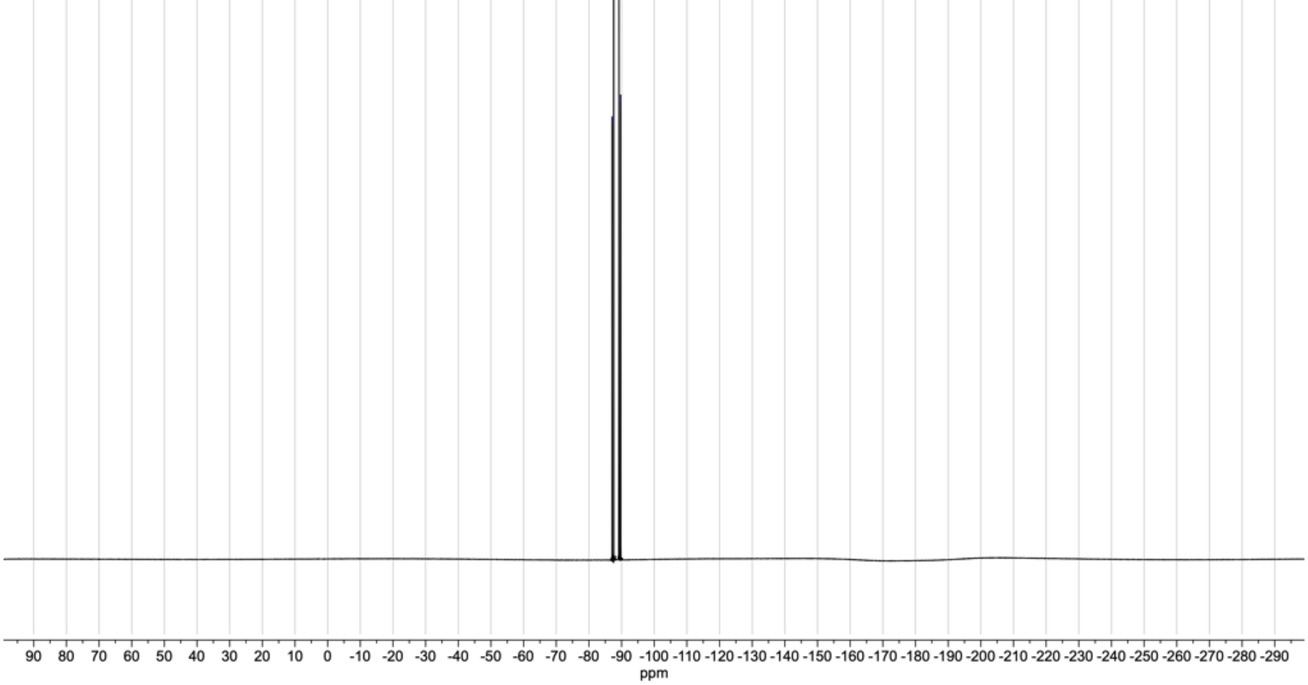

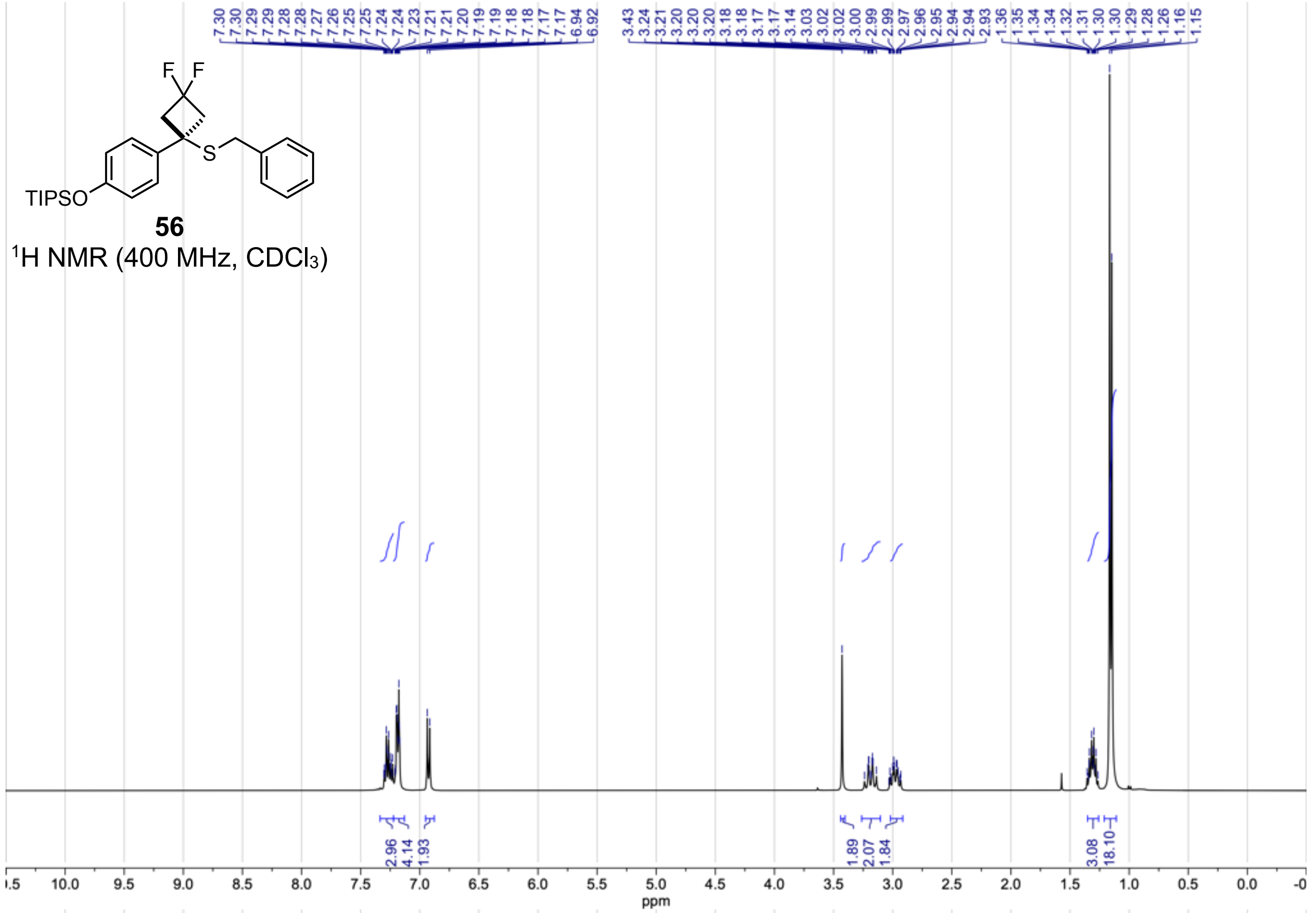
TIPSO
S
F F
56
^{1}H NMR (400 MHz, CDCl$_3$)
7.30 7.30 7.29 7.29 7.28 7.28 7.27 7.26 7.25 7.25 7.24 7.24 7.23 7.21 7.21 7.20 7.19 7.19 7.18 7.18 7.17 7.17 6.94 6.92
3.43 3.24 3.21 3.20 3.20 3.18 3.18 3.17 3.17 3.14 3.03 3.02 3.00 2.99 2.99 2.97 2.96 2.95 2.94 2.94 2.93
1.36 1.35 1.34 1.34 1.32 1.31 1.30 1.30 1.29 1.28 1.26 1.16 1.15
2.96 4.14 1.93 1.89 2.07 1.84 3.08 18.10
ppm

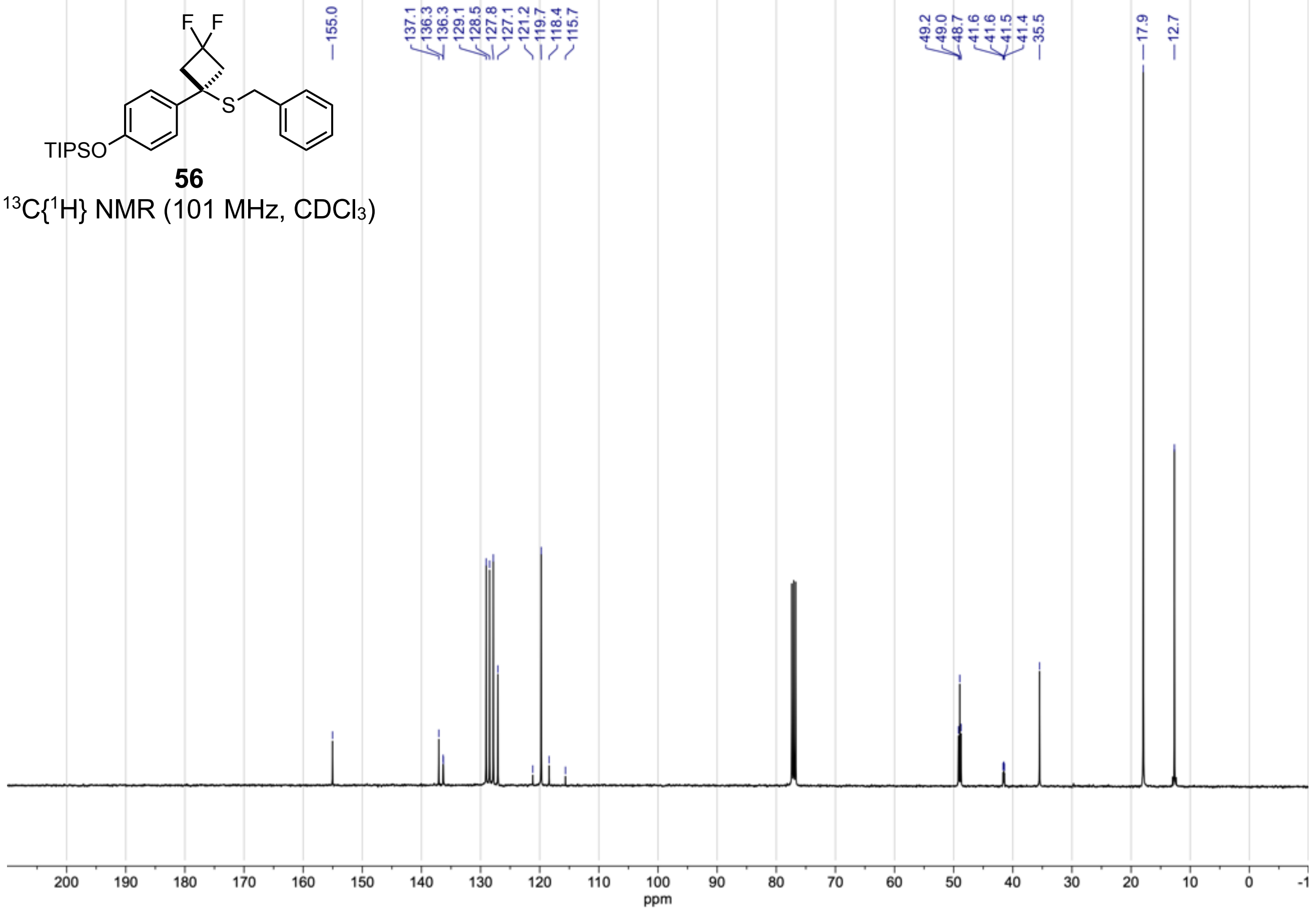
TIPSO
S
F F
56
^{13}C{^{1}H} NMR (101 MHz, CDCl$_3$)
155.0
137.1 136.3 136.3 129.1 128.5 127.8 127.1 121.2 119.7 118.4 115.7
49.2 49.0 48.7 41.6 41.5 41.4 35.5
17.9
12.7
ppm

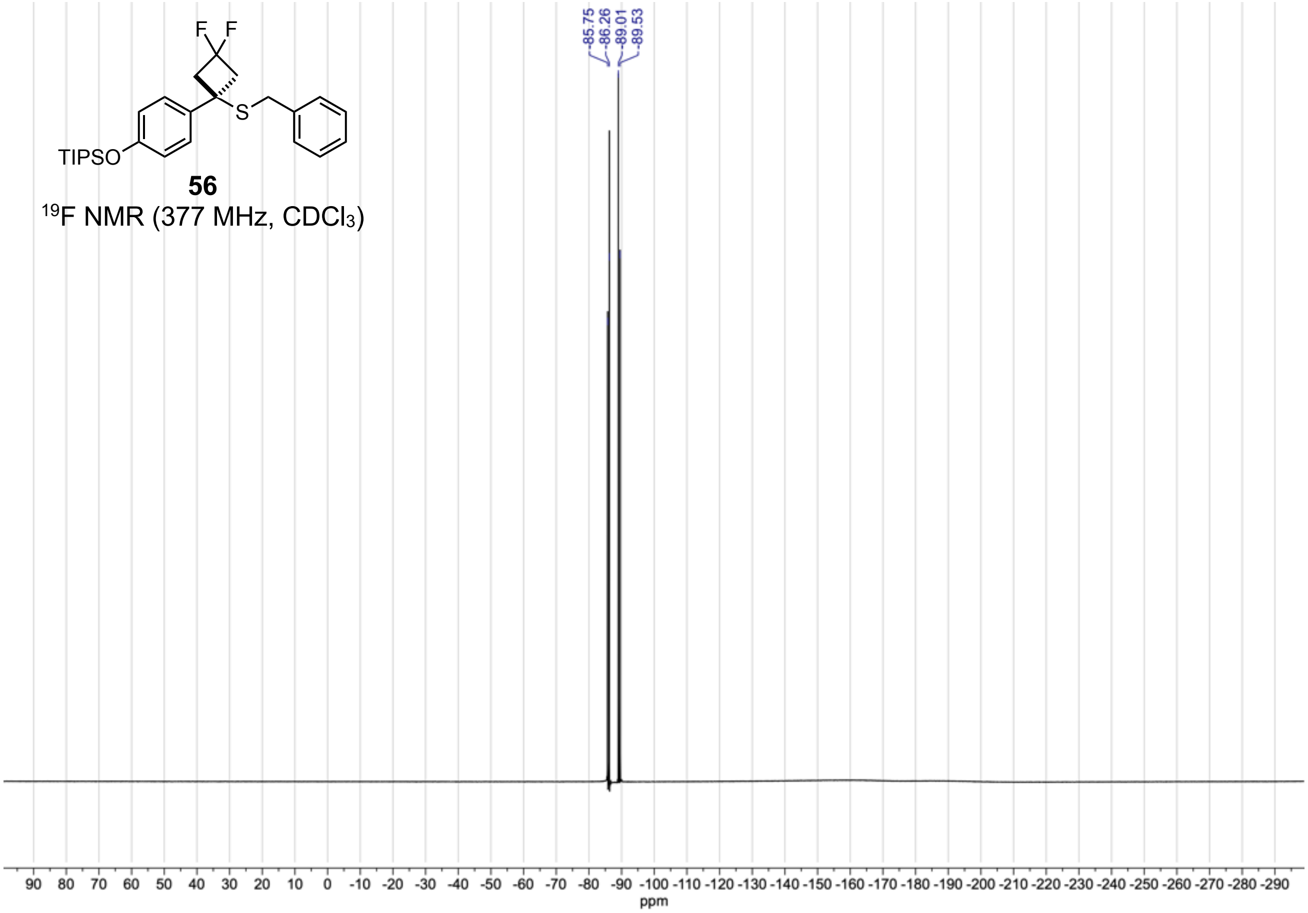
F F
S
TIPSO
56
^{19}F NMR (377 MHz, CDCl$_3$)
-85.75
-86.26
-89.01
-89.53
90 80 70 60 50 40 30 20 10 0 -10 -20 -30 -40 -50 -60 -70 -80 -90 -100 -110 -120 -130 -140 -150 -160 -170 -180 -190 -200 -210 -220 -230 -240 -250 -260 -270 -280 -290
ppm

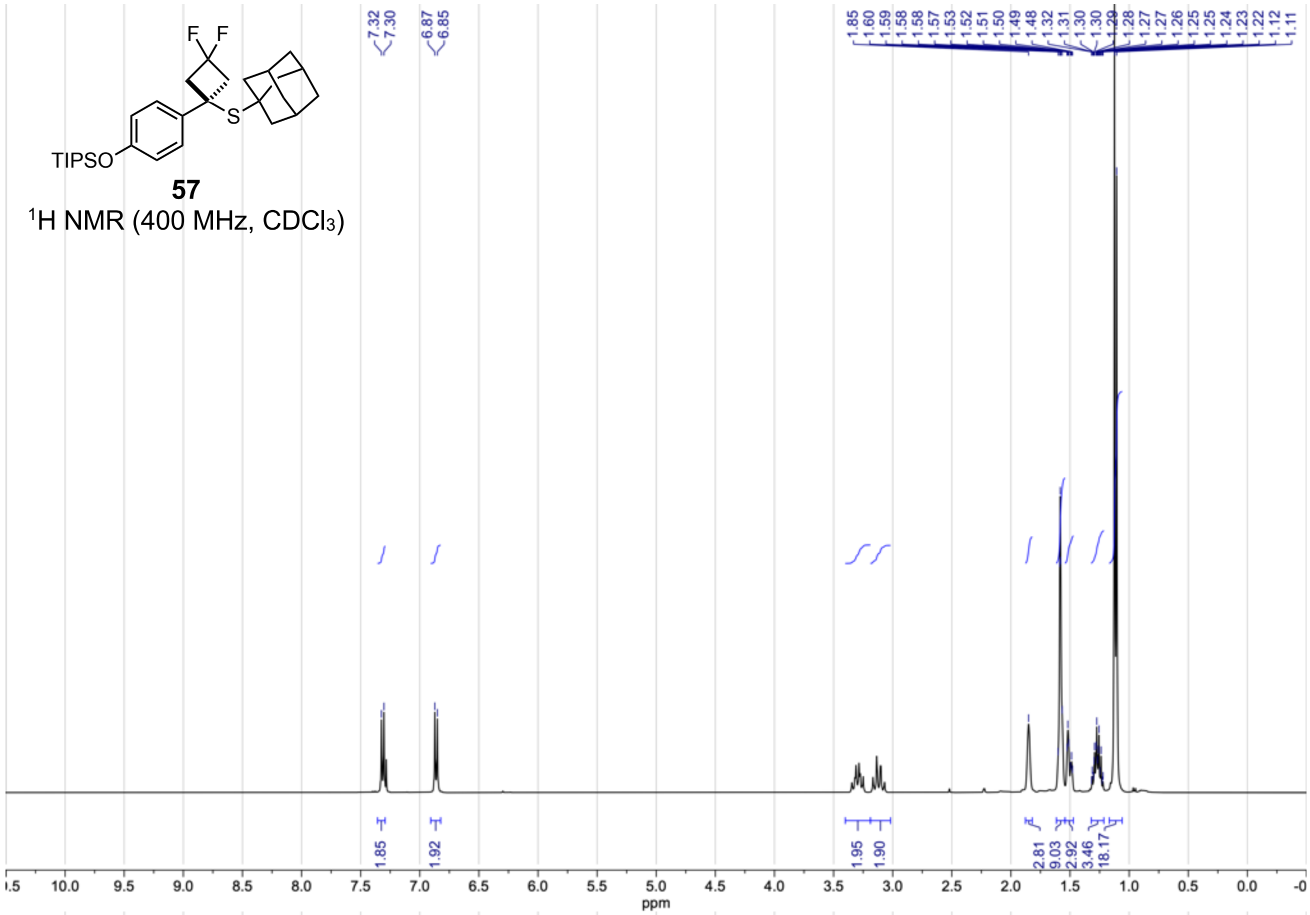

57
^{1}H NMR (400 MHz, $CDCl_3$)

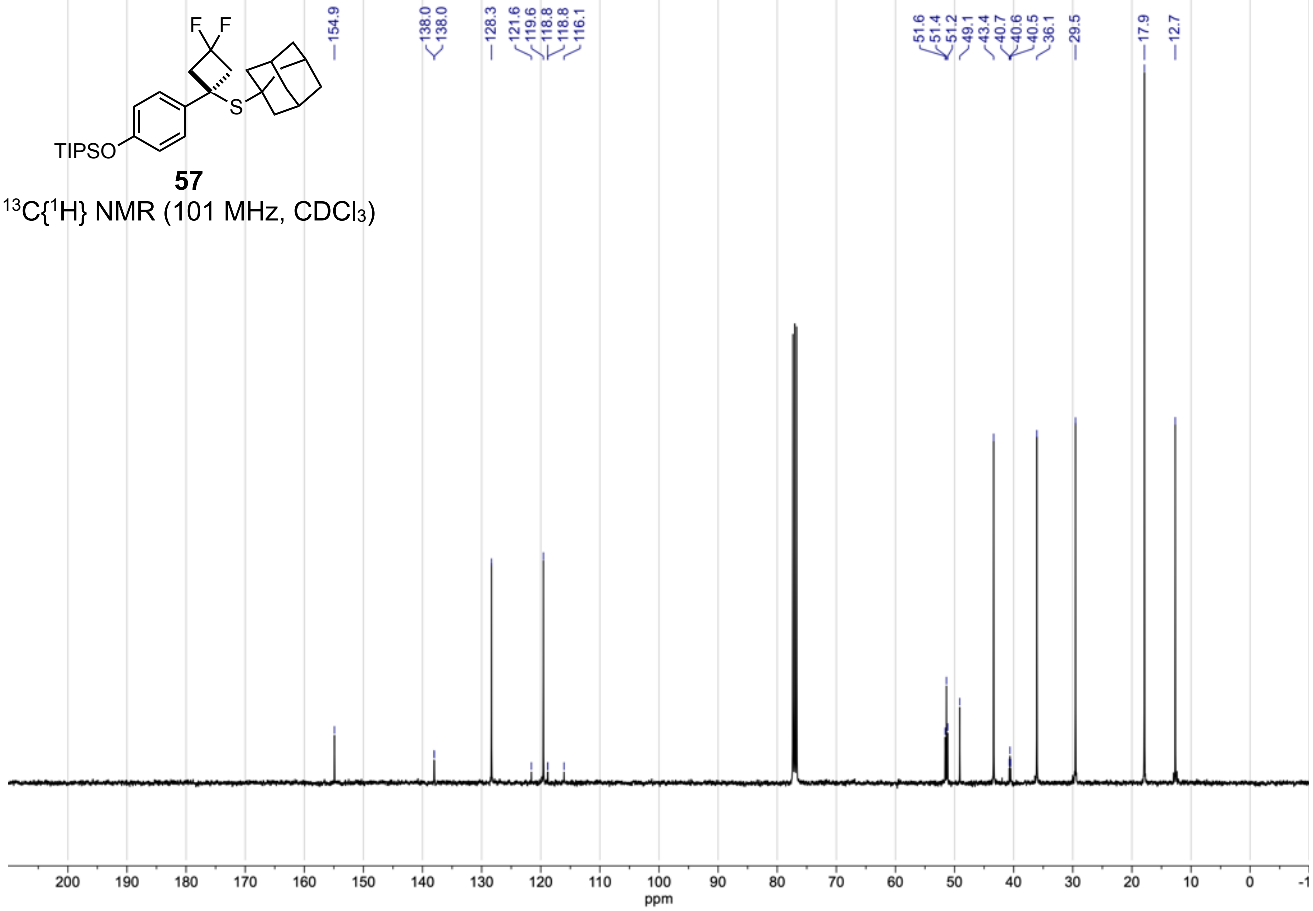

57
$^{13}C\{^1H\}$ NMR (101 MHz, $CDCl_3$)

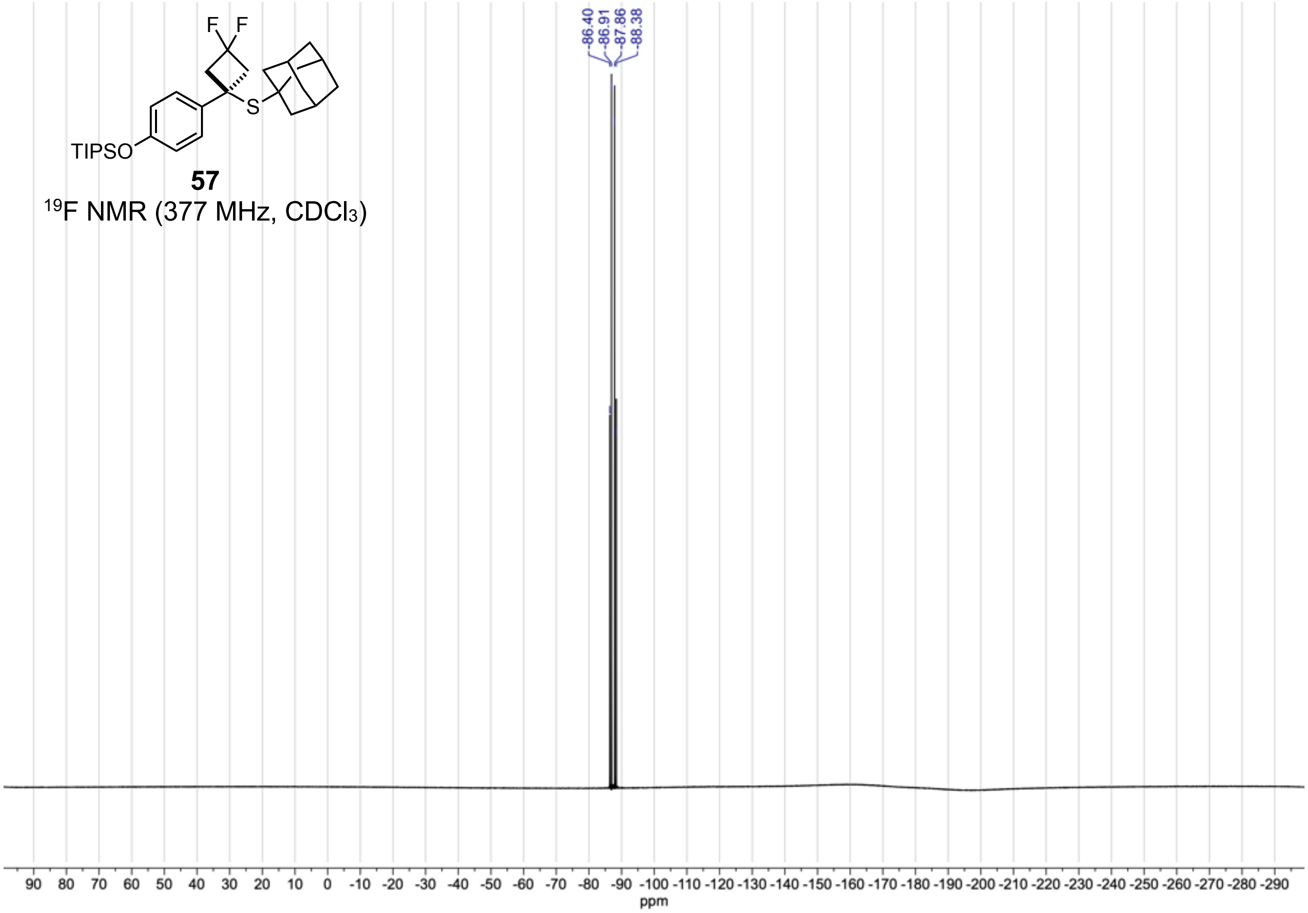
F F
TIPSO
S
57
^{19}F NMR (377 MHz, CDCl$_3$)
-86.40
-86.91
-87.86
-88.38
90 80 70 60 50 40 30 20 10 0 -10 -20 -30 -40 -50 -60 -70 -80 -90 -100 -110 -120 -130 -140 -150 -160 -170 -180 -190 -200 -210 -220 -230 -240 -250 -260 -270 -280 -290
ppm

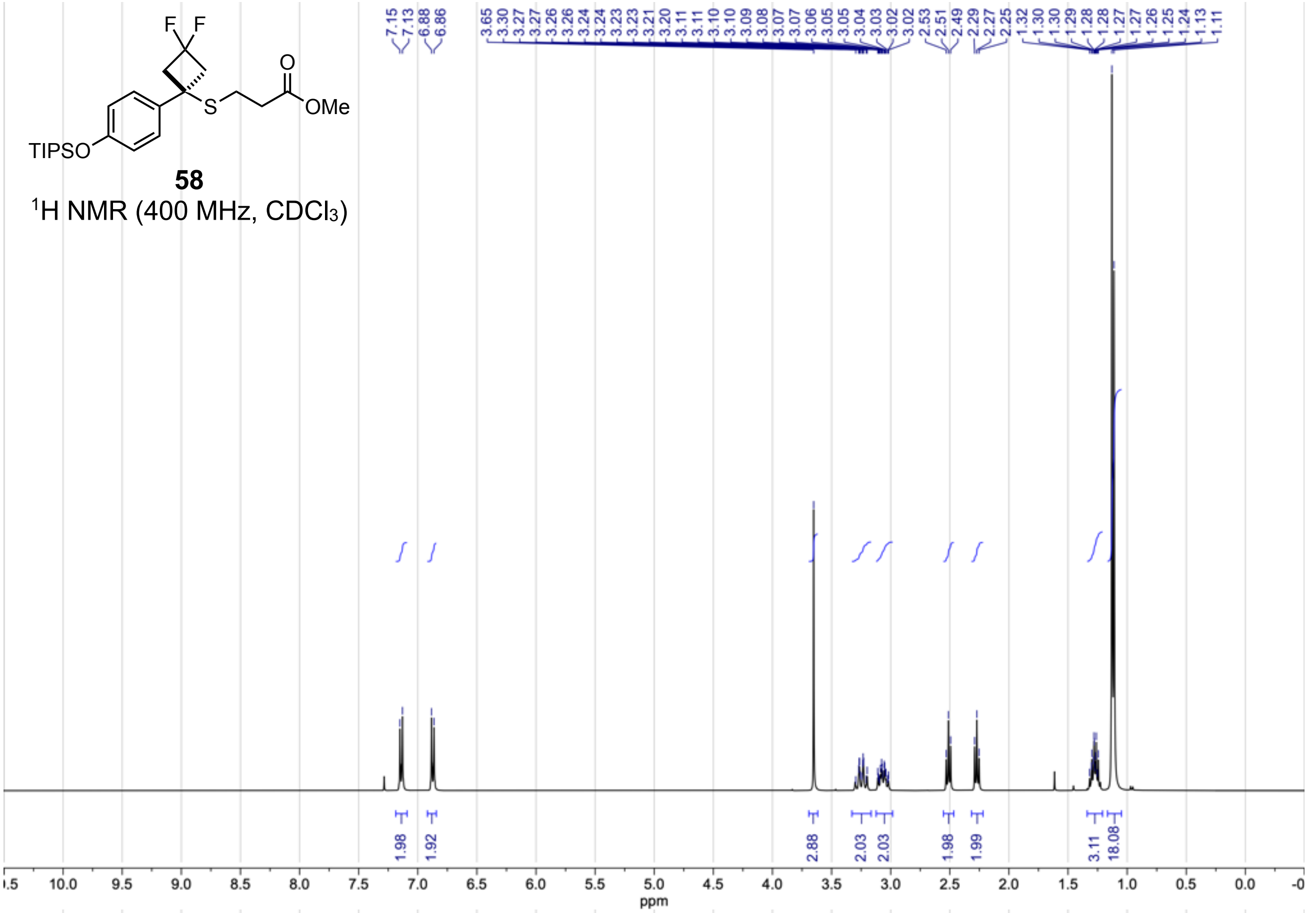

58

^{1}H NMR (400 MHz, $CDCl_3$)

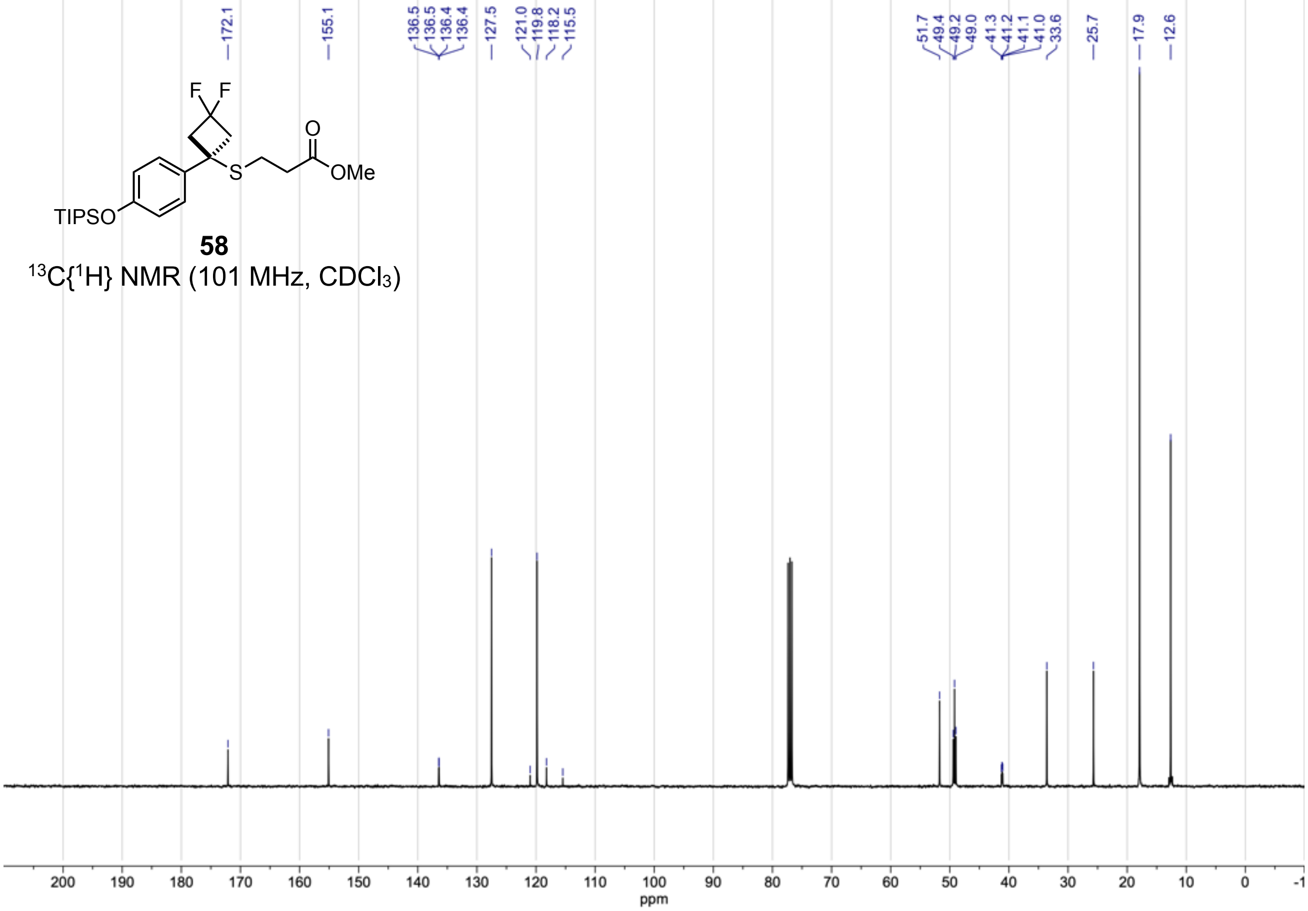

58

^{13}C{^{1}H} NMR (101 MHz, $CDCl_3$)

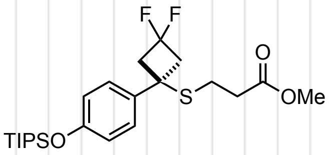

58

^{19}F NMR (377 MHz, $CDCl_3$)

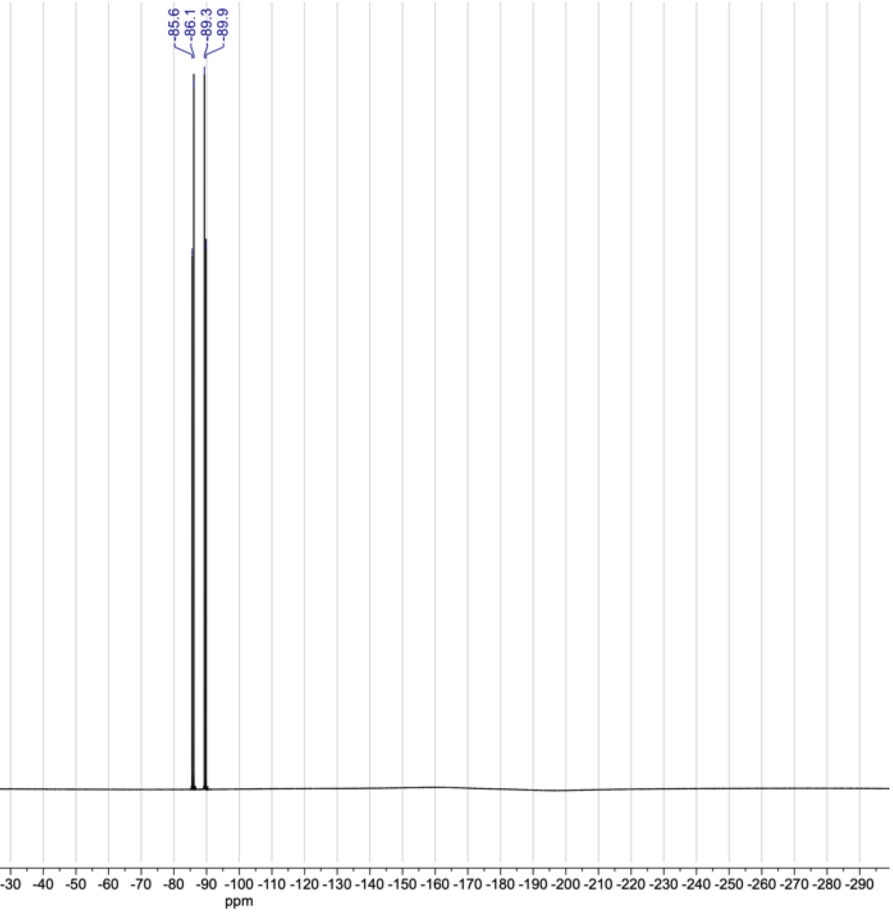

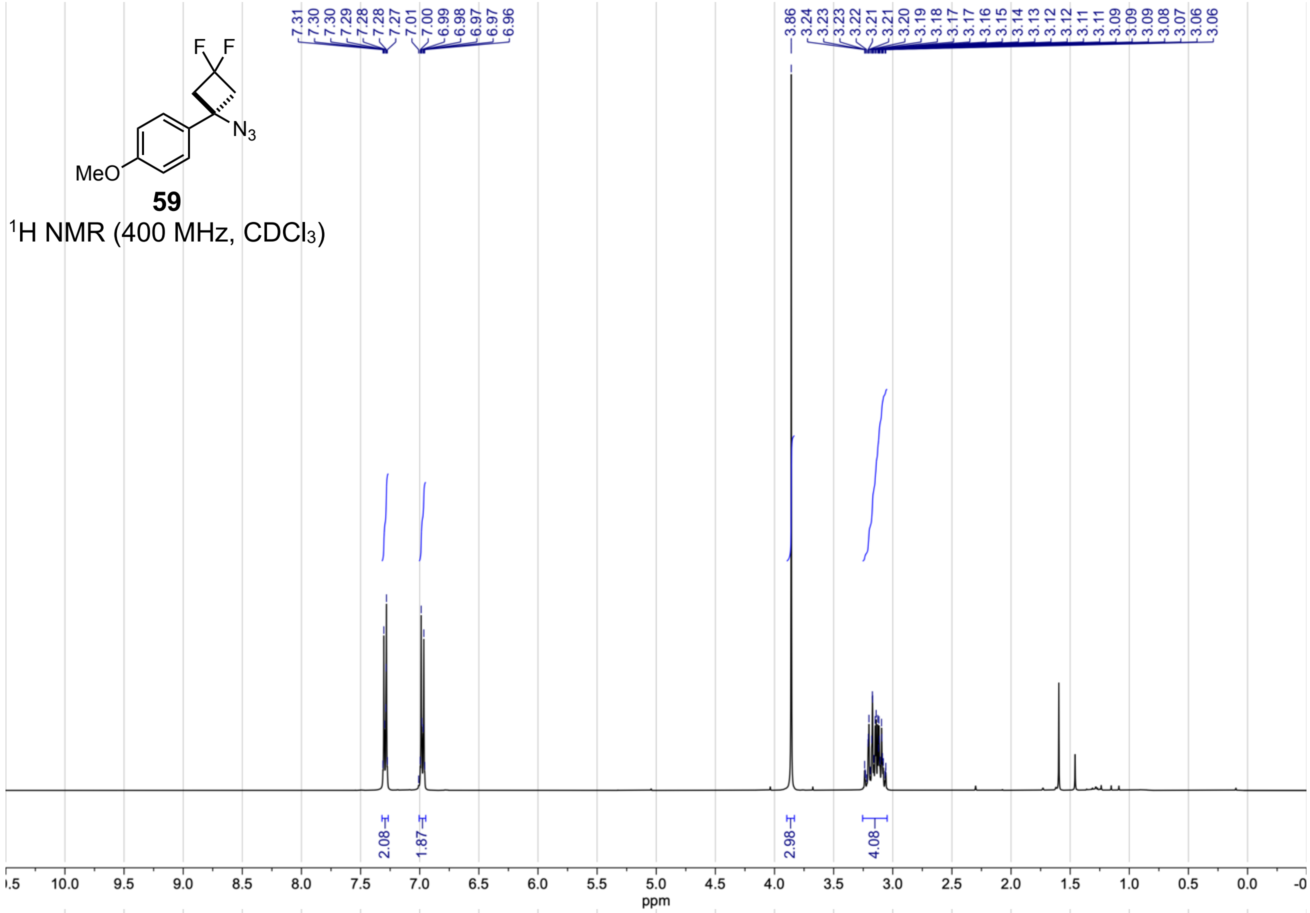

F F
N3
MeO
59
1H NMR (400 MHz, CDCl3)
7.31 7.30 7.29 7.28 7.28 7.27 7.01 7.00 6.99 6.98 6.97 6.97 6.96
3.86 3.24 3.23 3.23 3.22 3.21 3.21 3.20 3.19 3.18 3.17 3.17 3.16 3.15 3.14 3.13 3.12 3.12 3.11 3.11 3.09 3.09 3.08 3.07 3.06 3.06
2.08 1.87 2.98 4.08
ppm

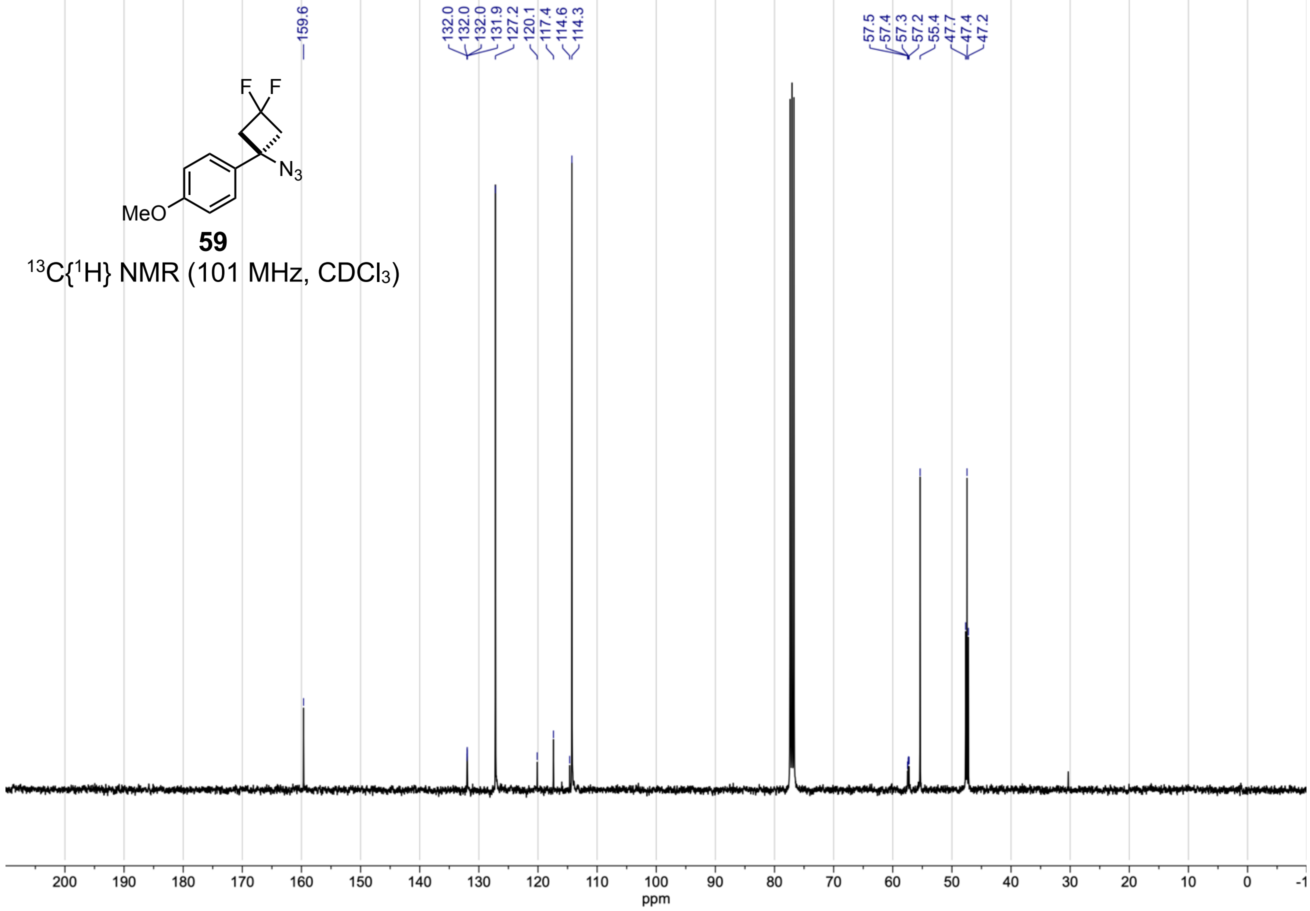

F F
N3
MeO
59
13C{1H} NMR (101 MHz, CDCl3)
159.6
132.0 132.0 132.0 131.9 127.2 120.1 117.4 114.6 114.3
57.5 57.4 57.3 57.2 55.4 47.7 47.4 47.2
ppm

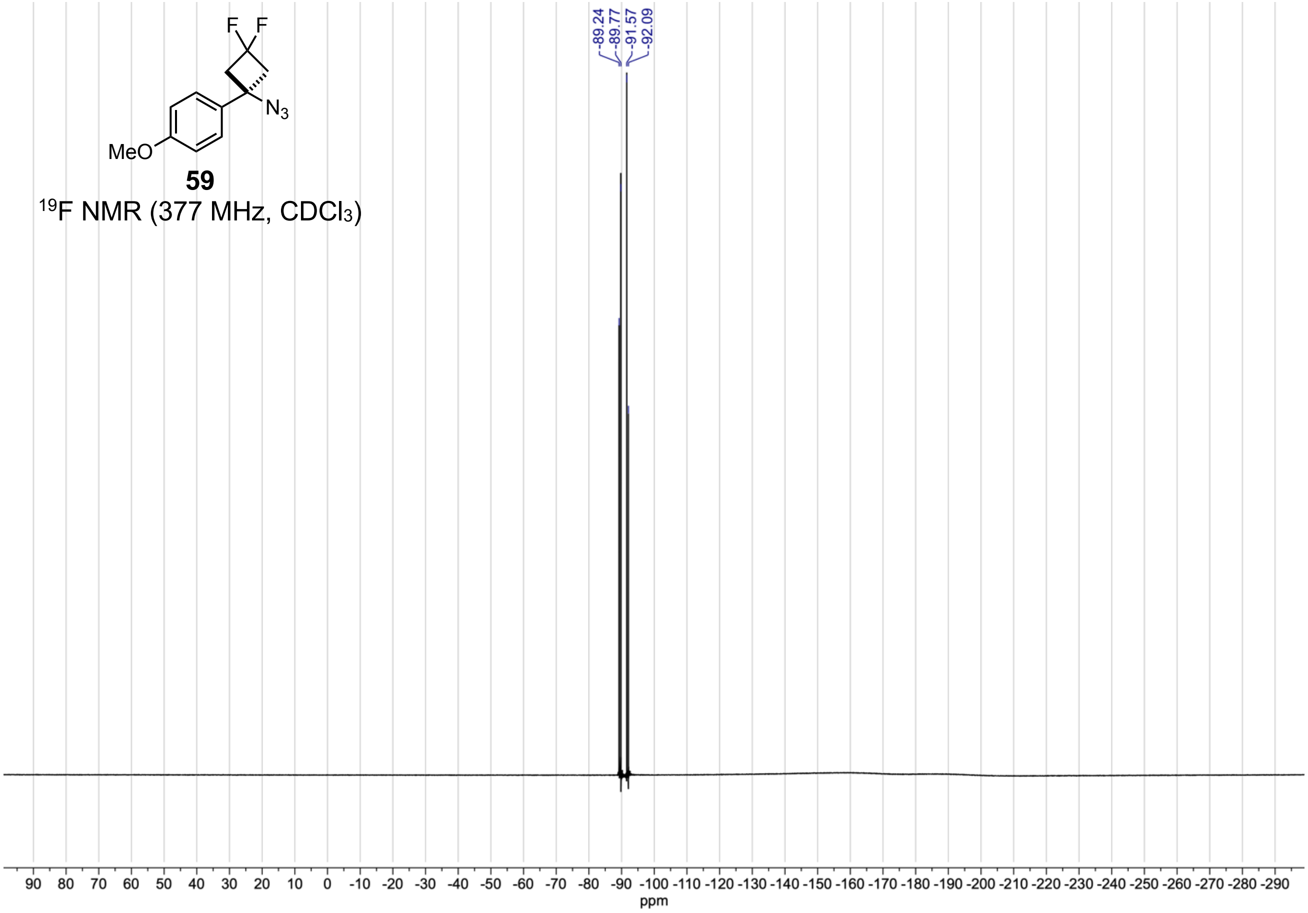
F
F
N3
MeO
59
19F NMR (377 MHz, CDCl3)
-89.24
-89.77
-91.57
-92.09
90 80 70 60 50 40 30 20 10 0 -10 -20 -30 -40 -50 -60 -70 -80 -90 -100 -110 -120 -130 -140 -150 -160 -170 -180 -190 -200 -210 -220 -230 -240 -250 -260 -270 -280 -290
ppm

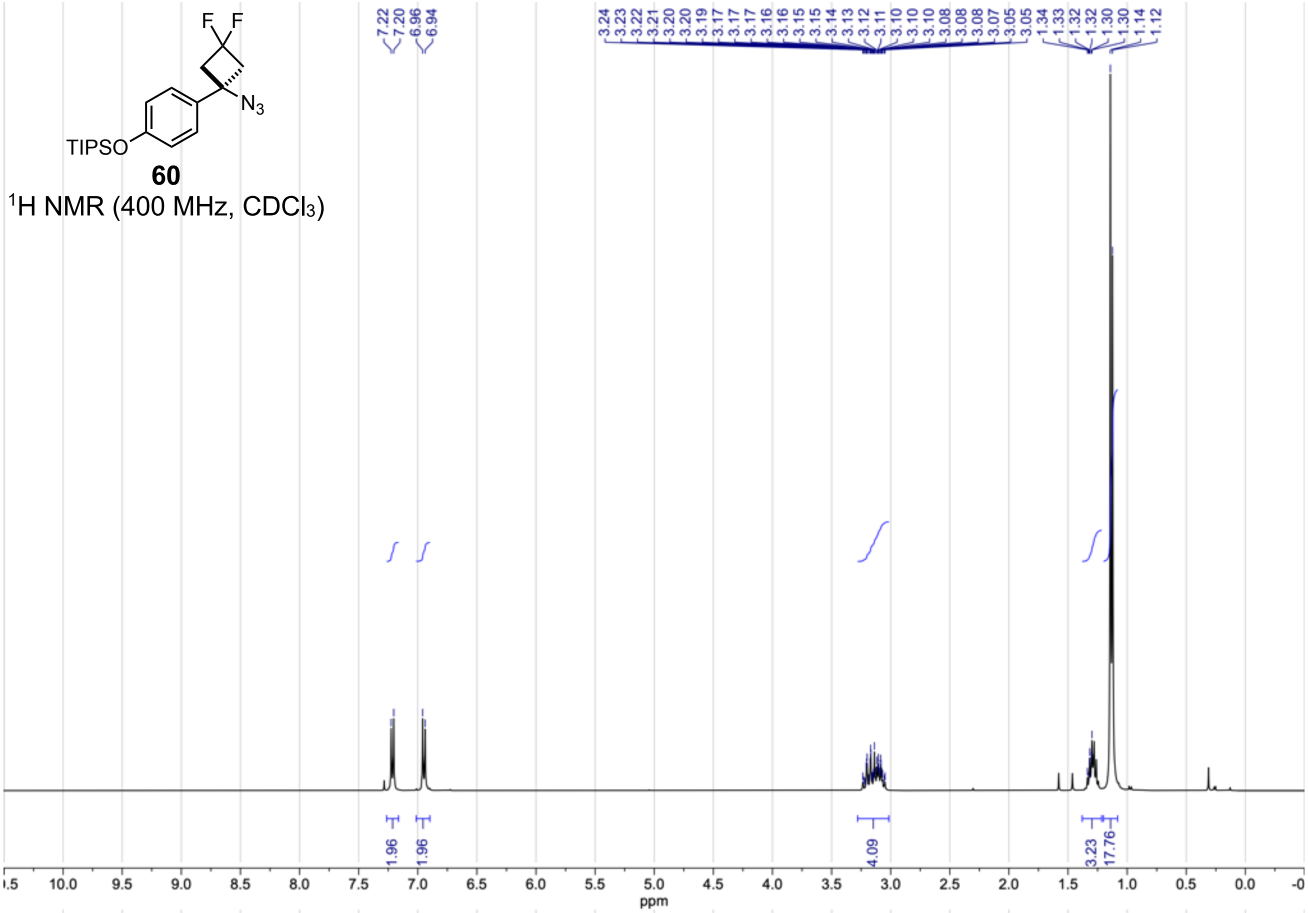

60

^{1}H NMR (400 MHz, $CDCl_3$)

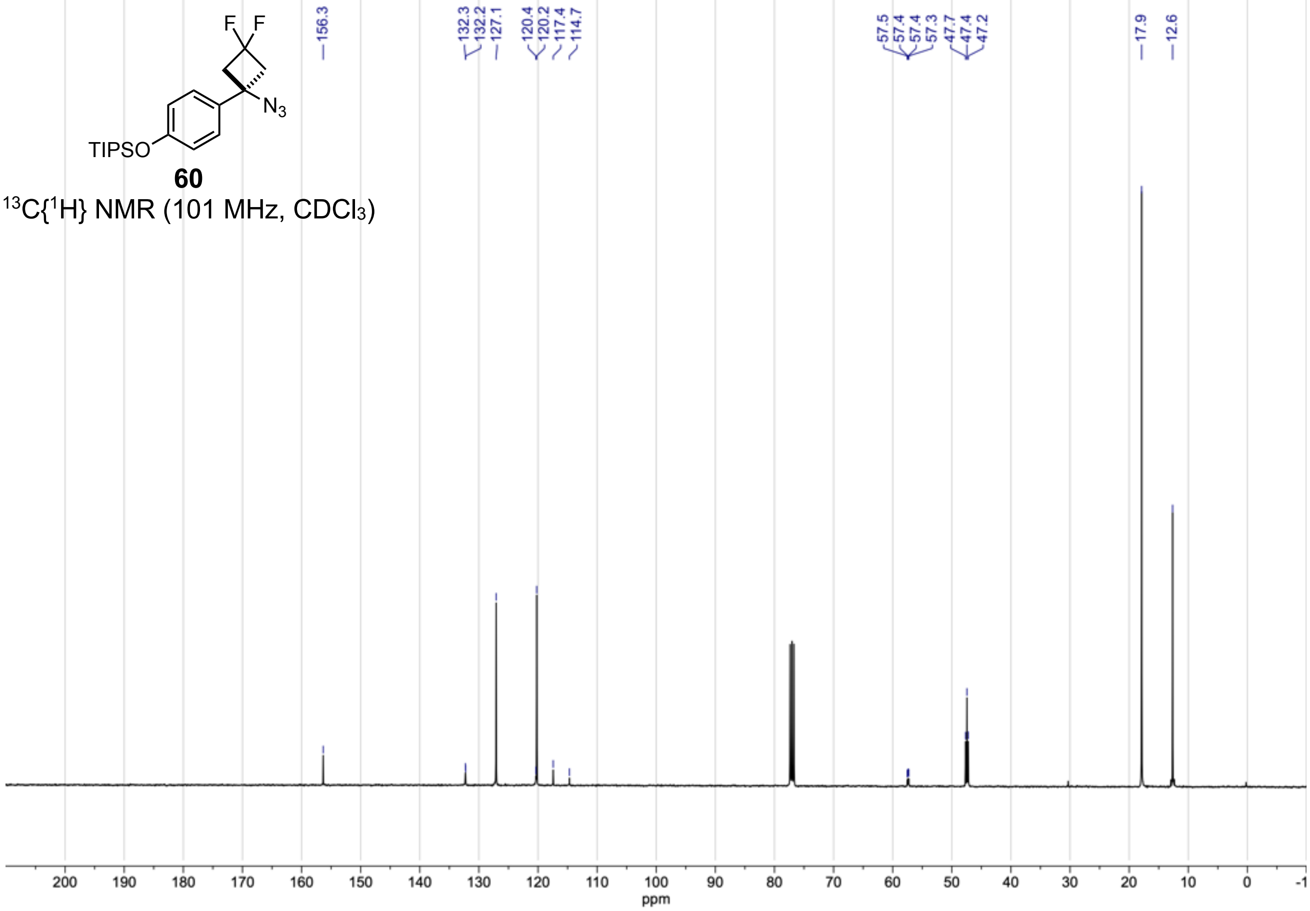

60

$^{13}C\{^1H\}$ NMR (101 MHz, $CDCl_3$)

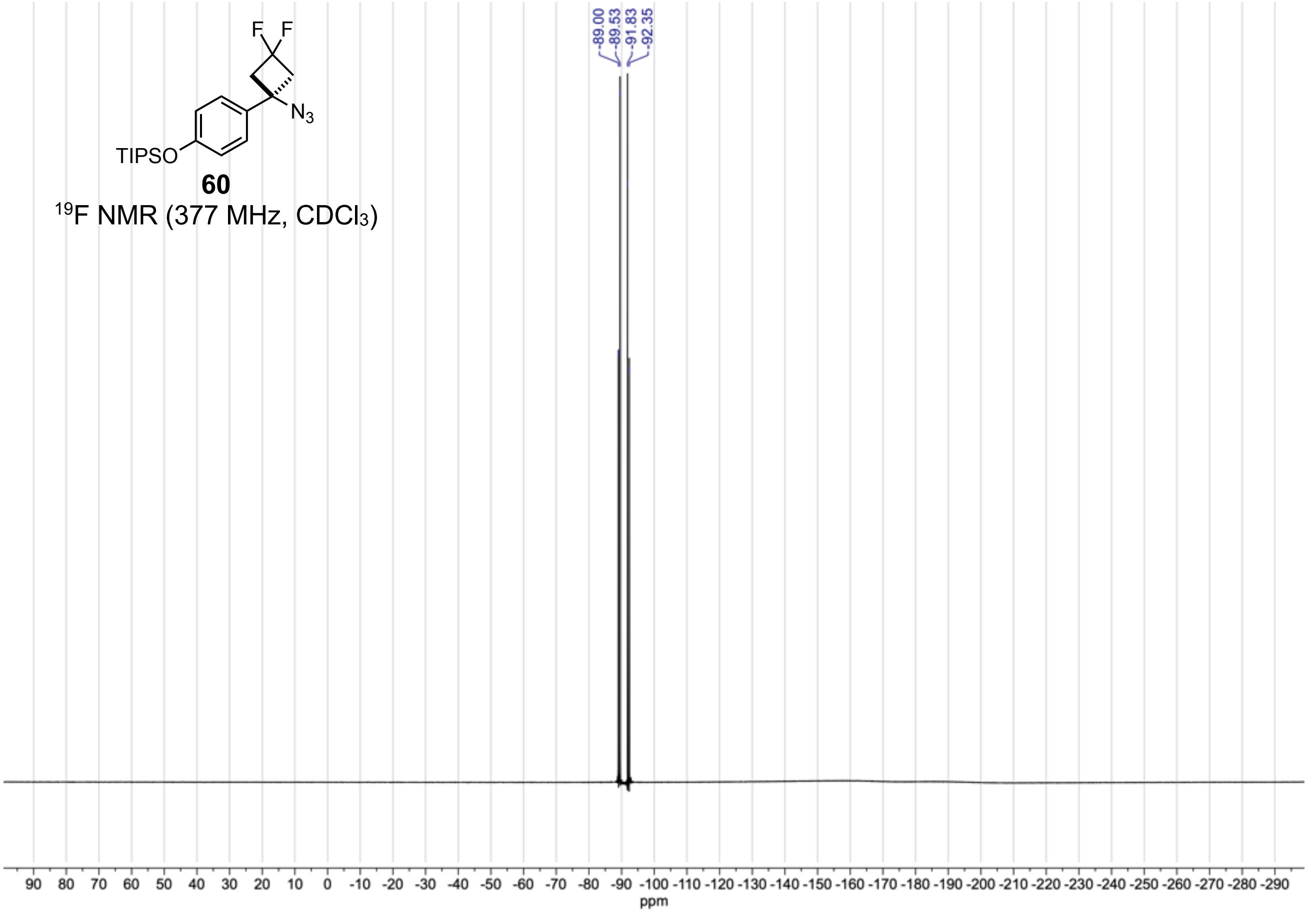
F F
N3
TIPSO
60
19F NMR (377 MHz, CDCl3)
-89.00
-89.53
-91.83
-92.35
90 80 70 60 50 40 30 20 10 0 -10 -20 -30 -40 -50 -60 -70 -80 -90 -100 -110 -120 -130 -140 -150 -160 -170 -180 -190 -200 -210 -220 -230 -240 -250 -260 -270 -280 -290
ppm

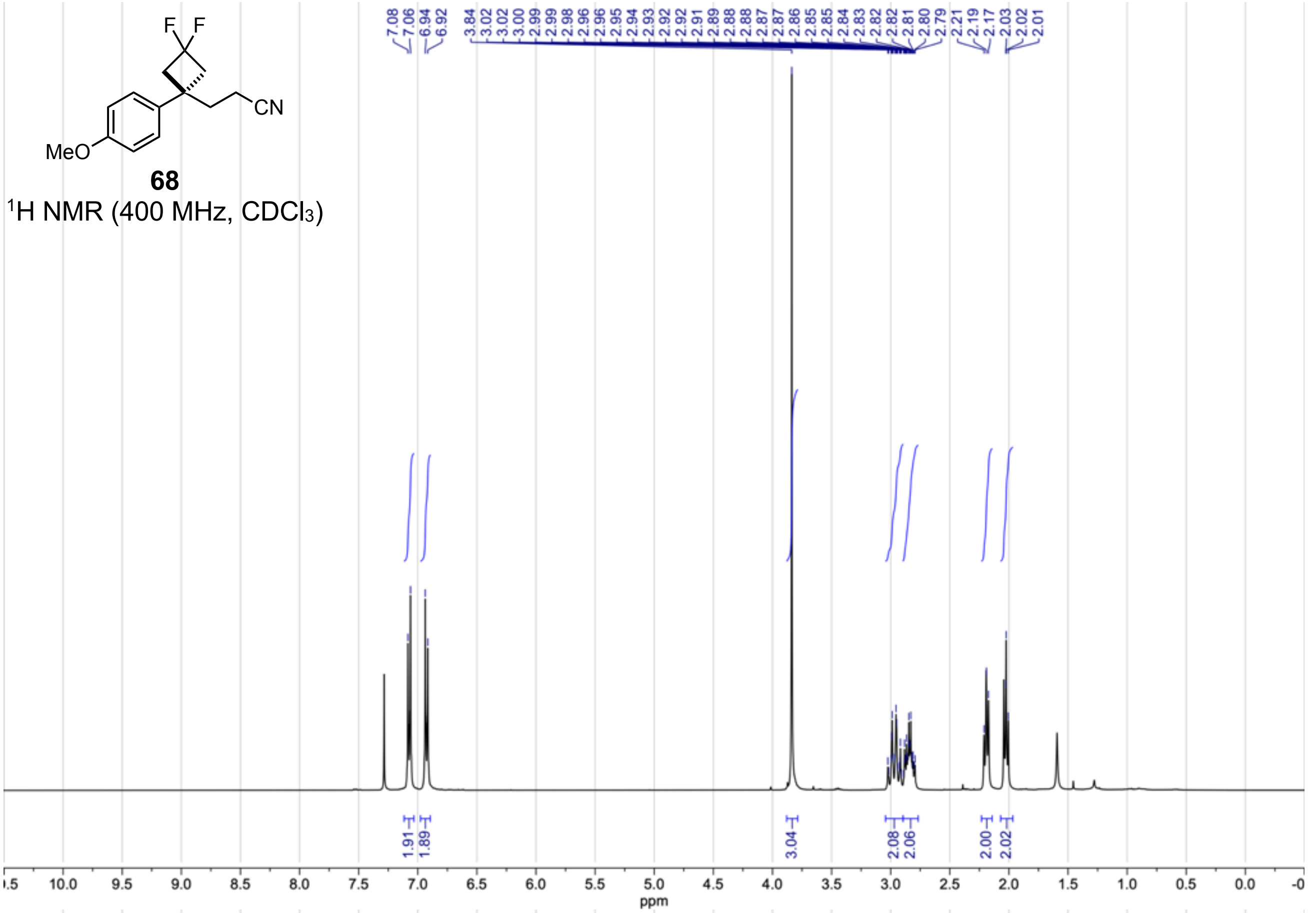

68

^{1}H NMR (400 MHz, $CDCl_3$)

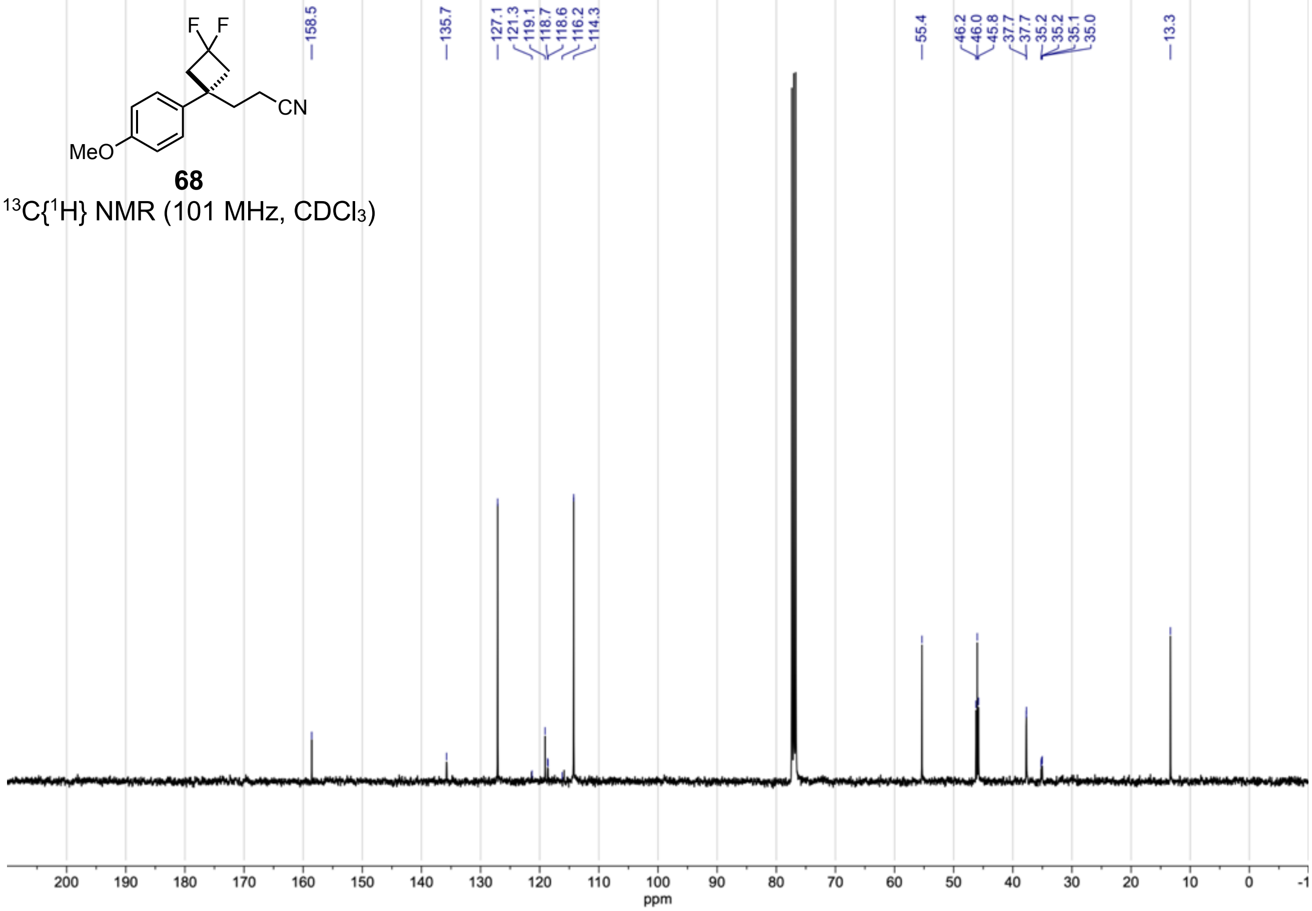

68

$^{13}C\{^1H\}$ NMR (101 MHz, $CDCl_3$)

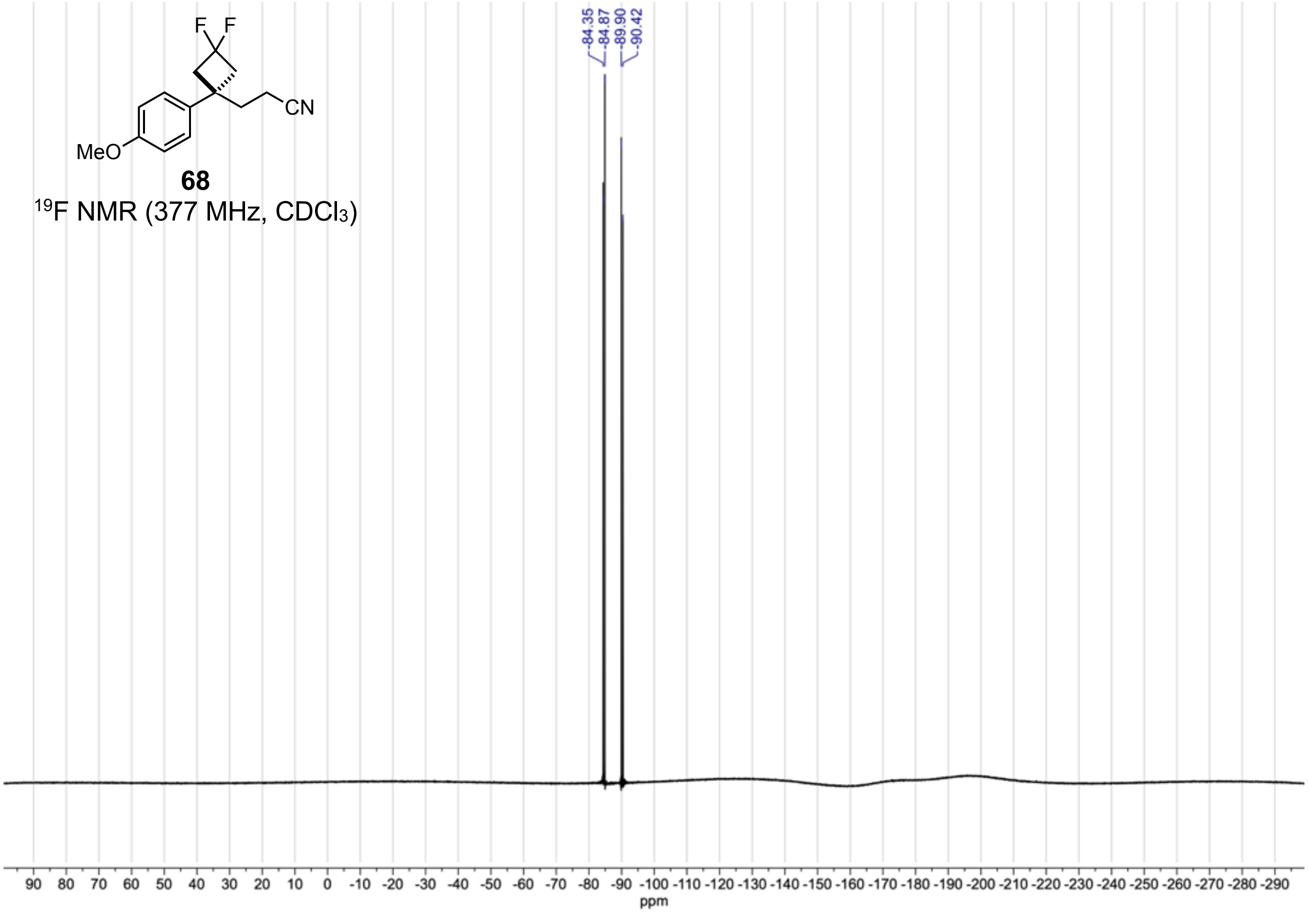
F F
CN
MeO
68
19F NMR (377 MHz, CDCl3)
-84.35
-84.87
-89.90
-90.42
90 80 70 60 50 40 30 20 10 0 -10 -20 -30 -40 -50 -60 -70 -80 -90 -100 -110 -120 -130 -140 -150 -160 -170 -180 -190 -200 -210 -220 -230 -240 -250 -260 -270 -280 -290
ppm

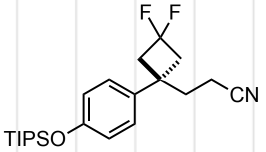

69

^{1}H NMR (400 MHz, $CDCl_3$)

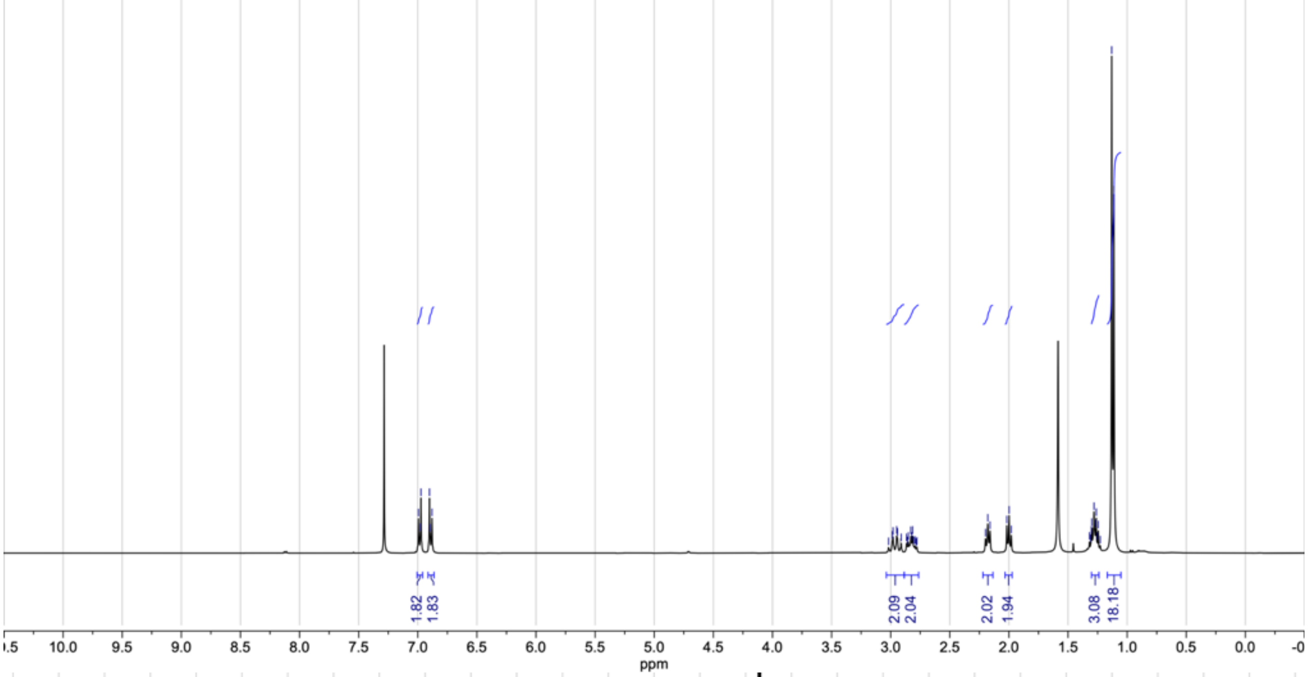

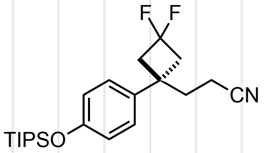

69

^{13}C{^{1}H} NMR (101 MHz, $CDCl_3$)

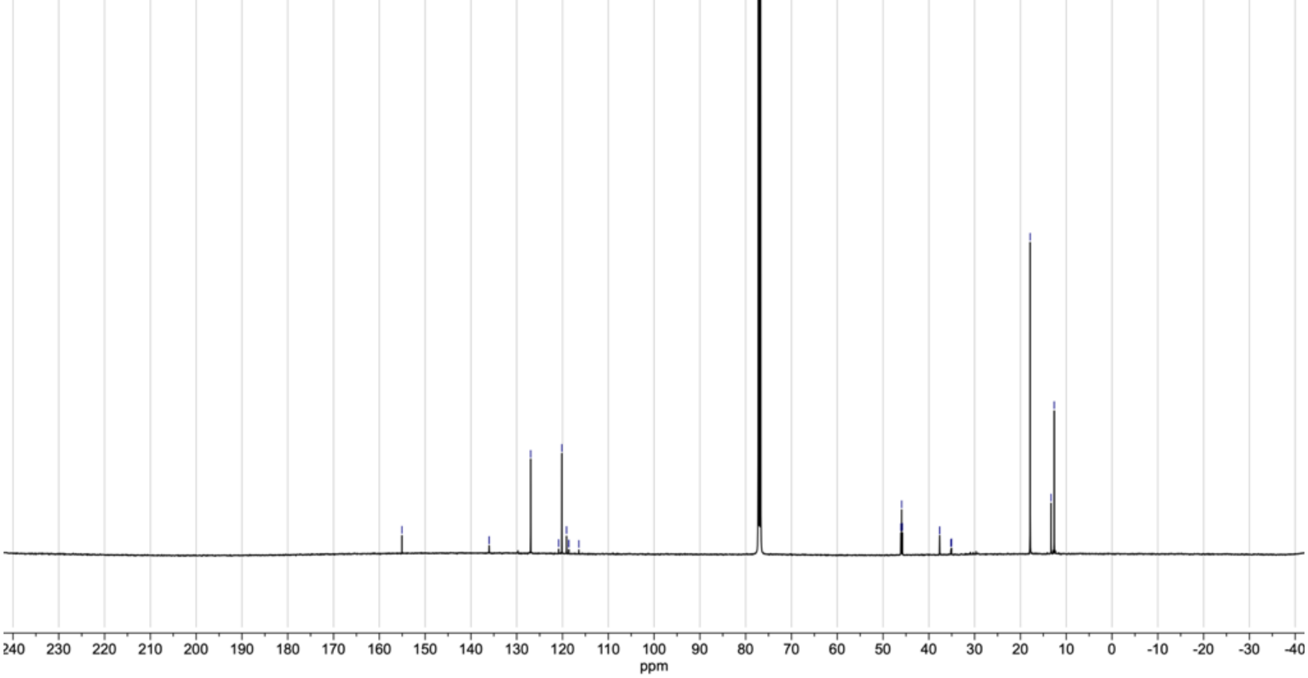

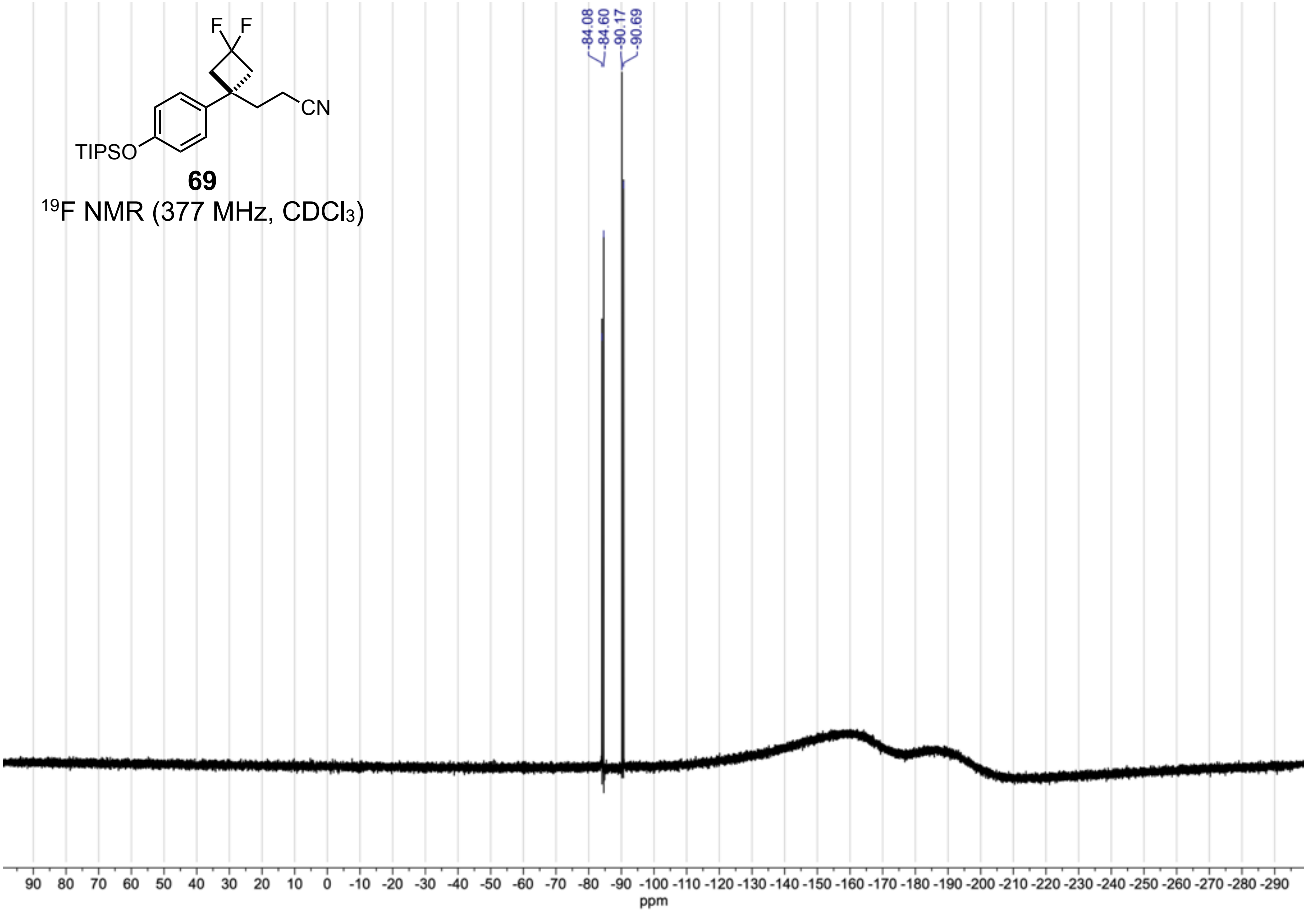

69

^{19}F NMR (377 MHz, $CDCl_3$)

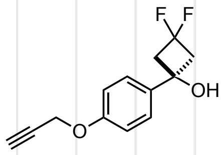

61

^{1}H NMR (400 MHz, $CDCl_3$)

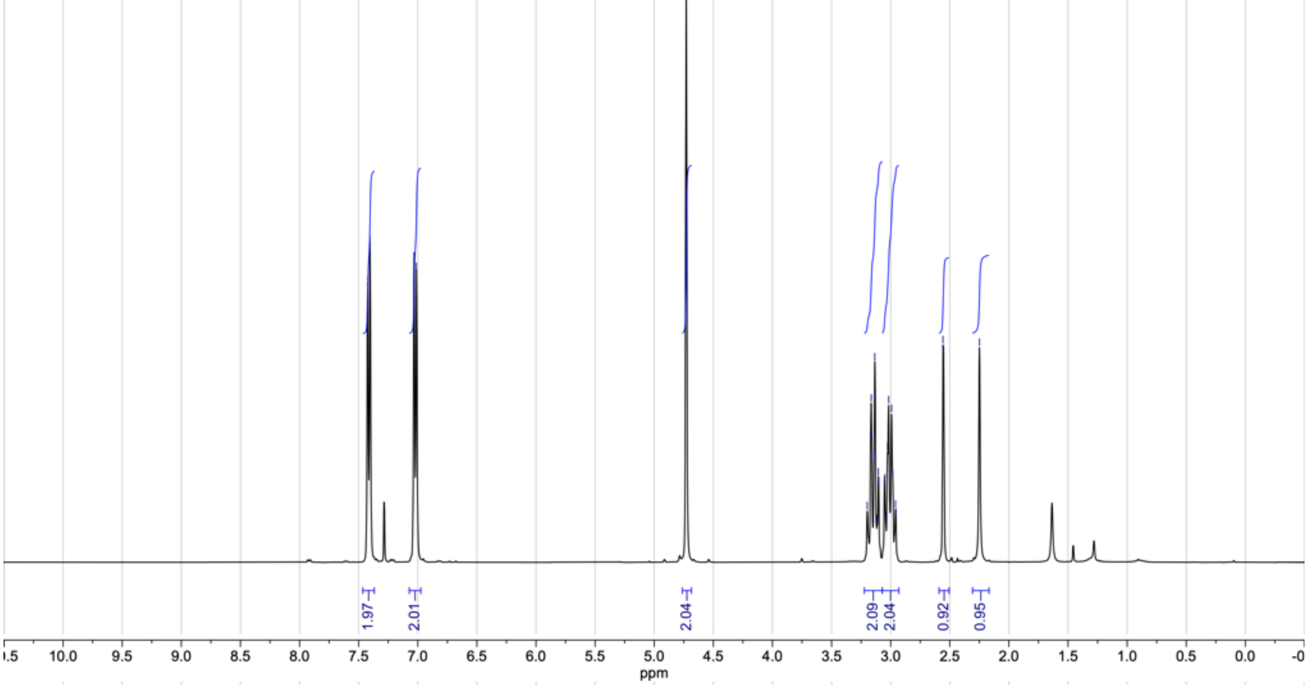

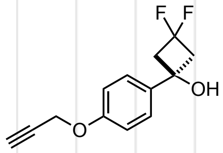

61

$^{13}C\{^{1}H\}$ NMR (101 MHz, $CDCl_3$)

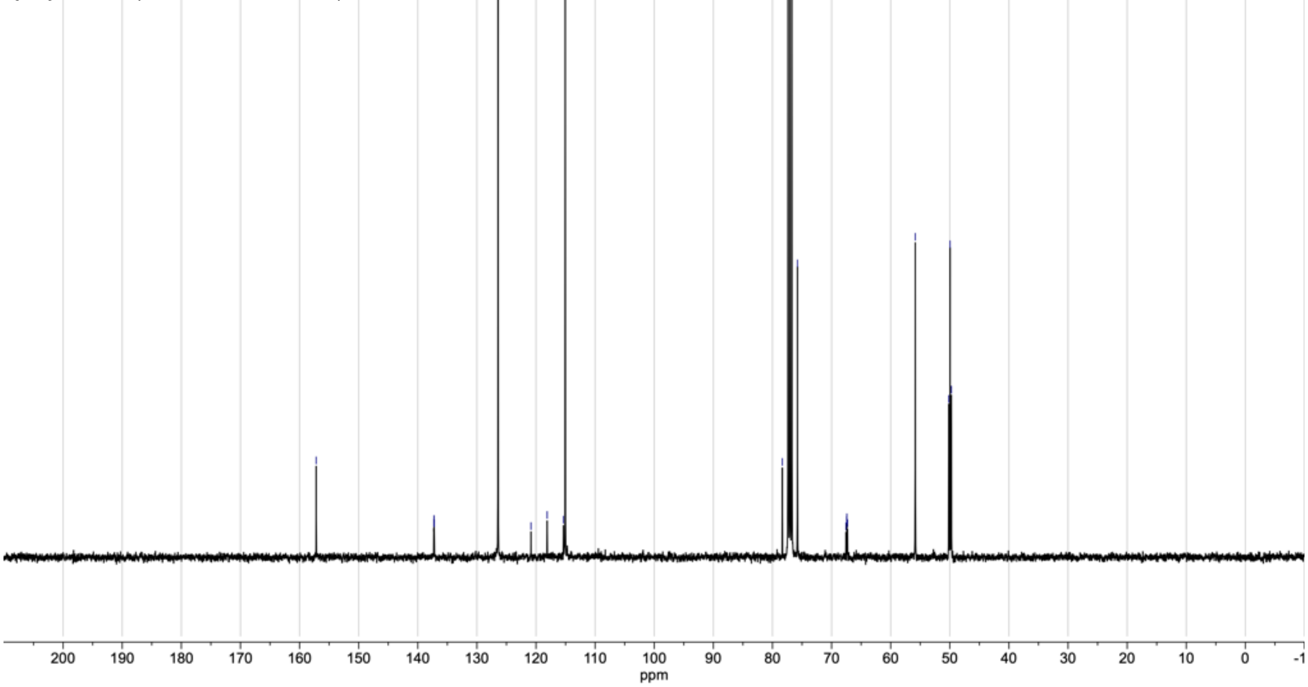

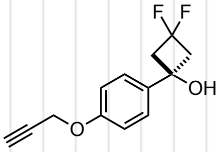

61

^{19}F NMR (377 MHz, $CDCl_3$)

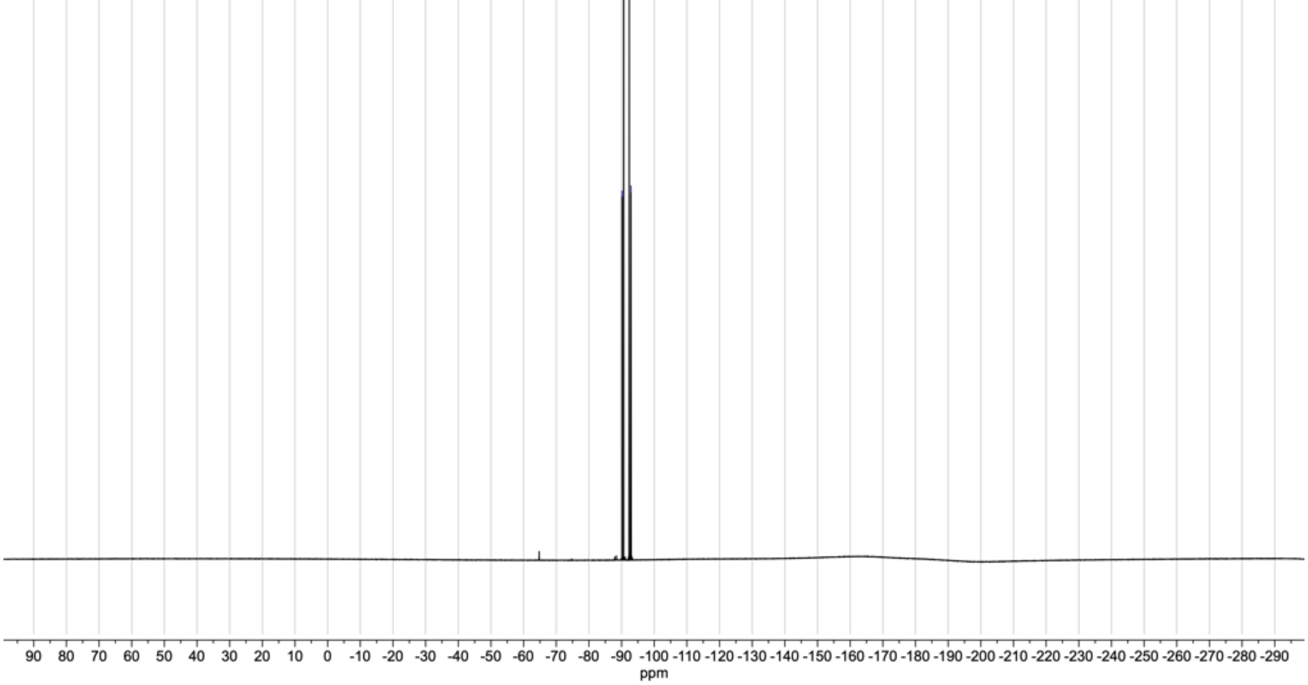

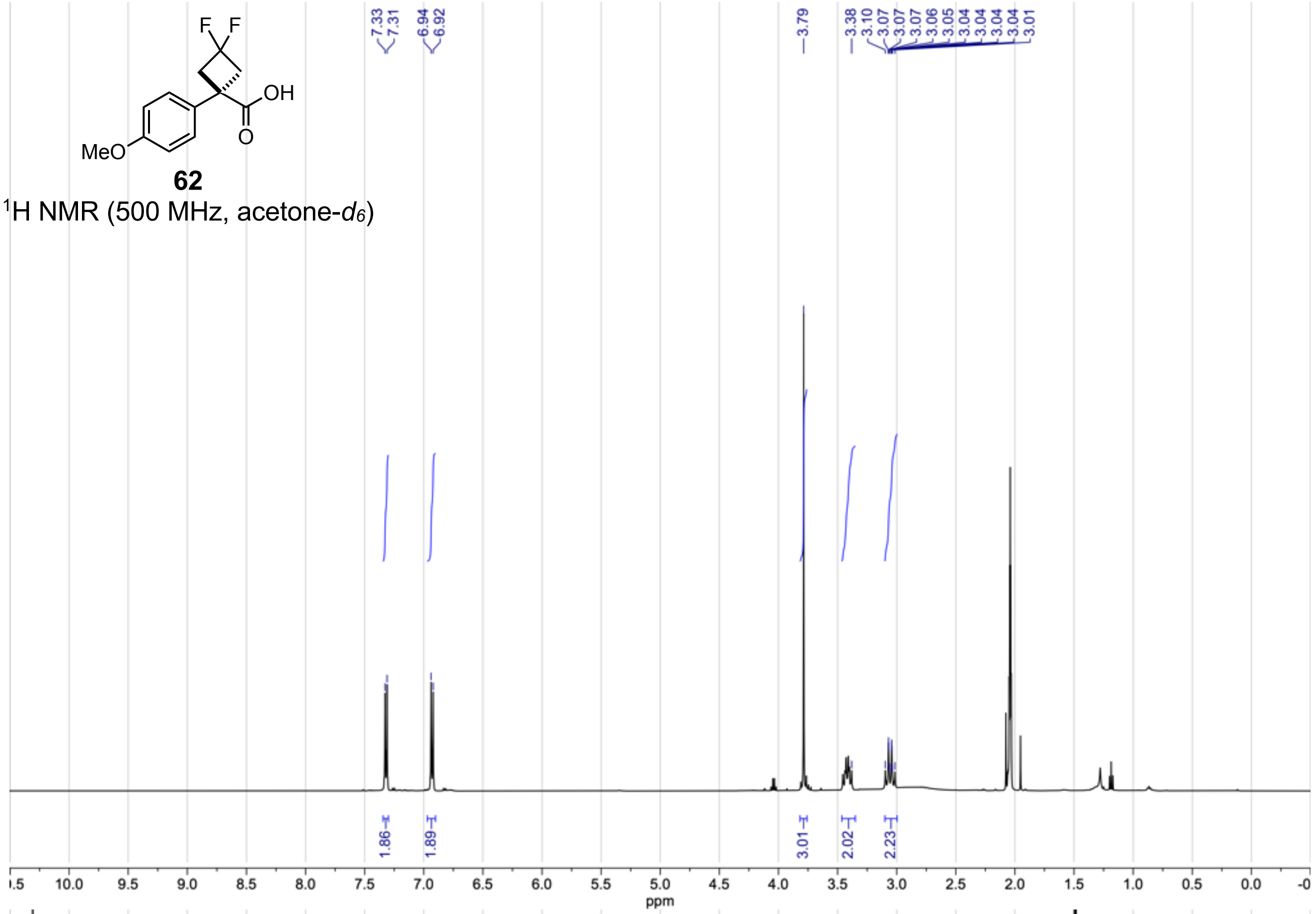
F F
OH
O
MeO
62
^{1}H NMR (500 MHz, acetone-d_6)
7.33
7.31
6.94
6.92
3.79
3.38
3.10
3.07
3.07
3.07
3.06
3.05
3.04
3.04
3.04
3.04
3.01
1.86
1.89
3.01
2.02
2.23
10.0 9.5 9.0 8.5 8.0 7.5 7.0 6.5 6.0 5.5 5.0 4.5 4.0 3.5 3.0 2.5 2.0 1.5 1.0 0.5 0.0
ppm

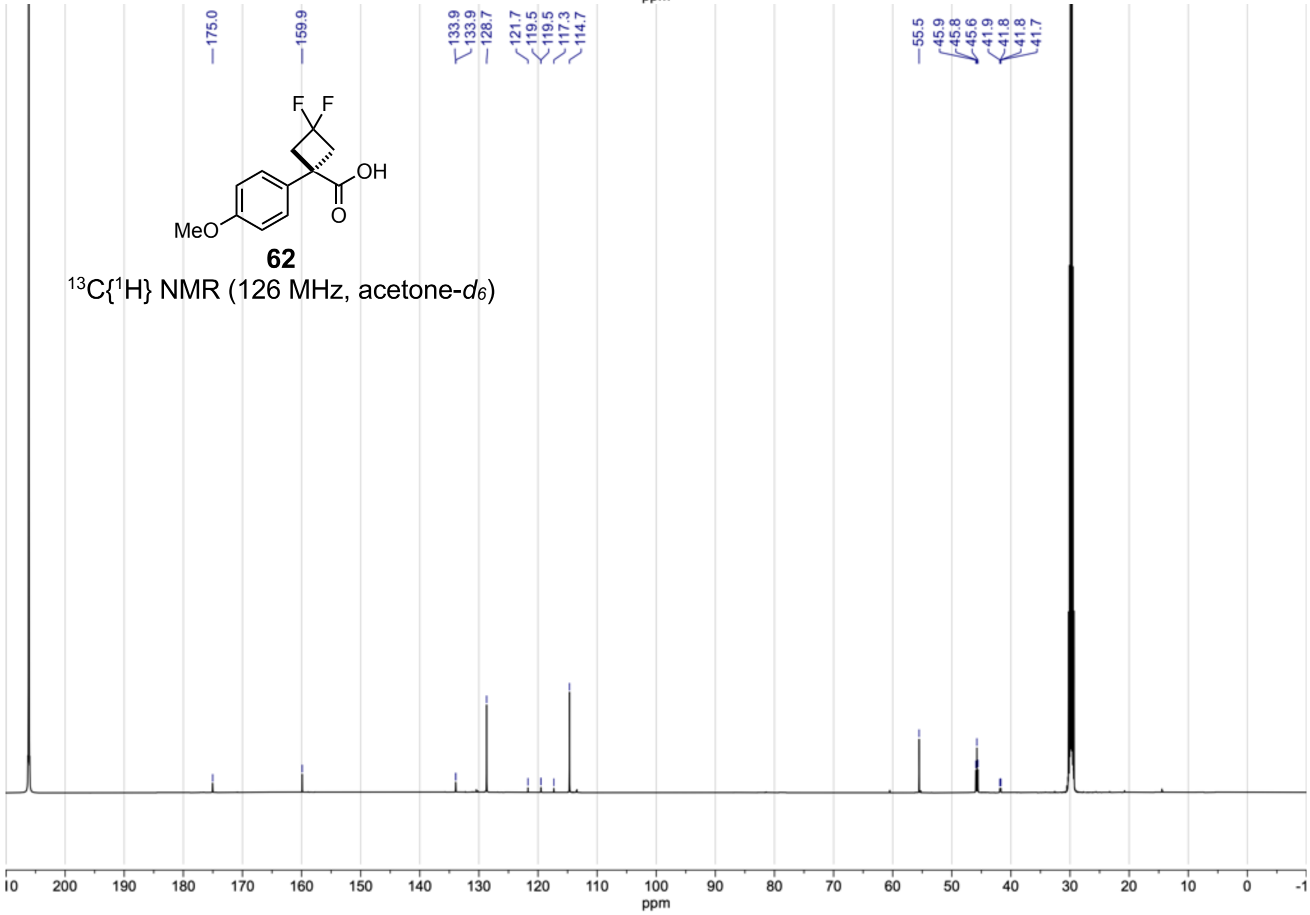
175.0
159.9
133.9
133.9
128.7
121.7
119.5
119.5
117.3
114.7
55.5
45.9
45.8
45.6
41.9
41.8
41.7
F F
OH
O
MeO
62
^{13}C{^{1}H} NMR (126 MHz, acetone-d_6)
200 190 180 170 160 150 140 130 120 110 100 90 80 70 60 50 40 30 20 10 0
ppm

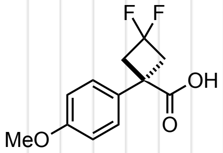

62

^{19}F NMR (377 MHz, acetone-d_6)

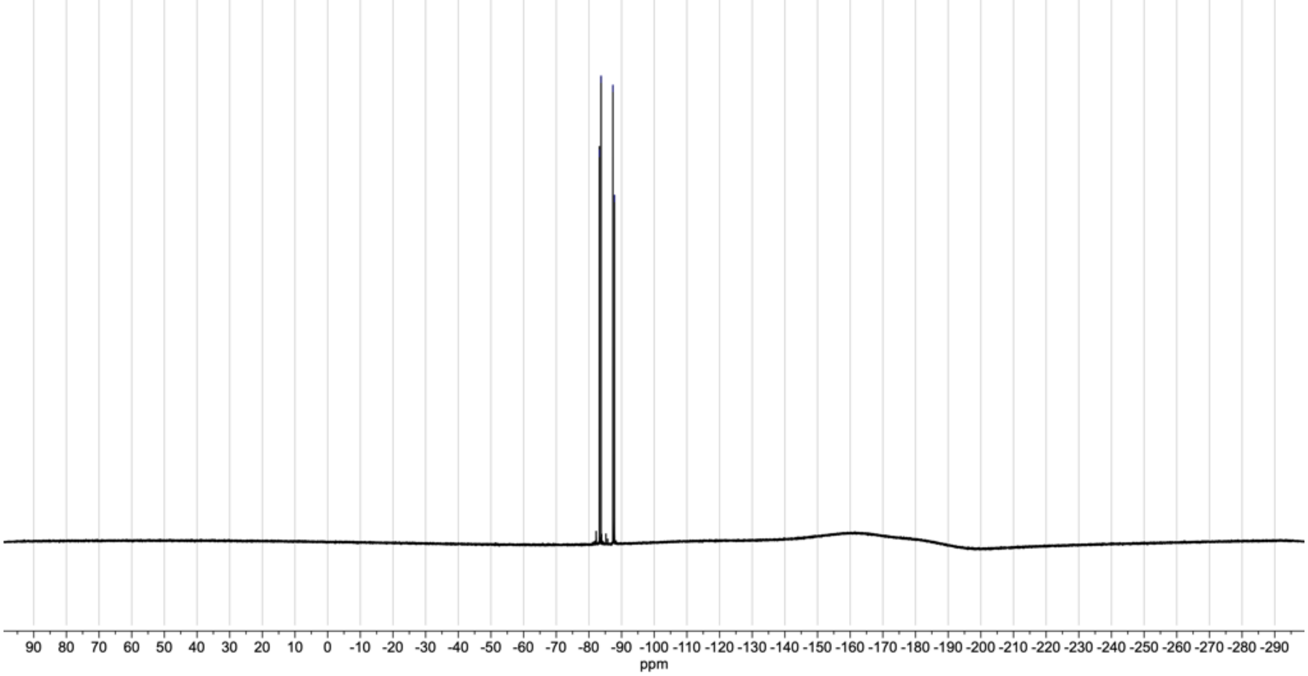

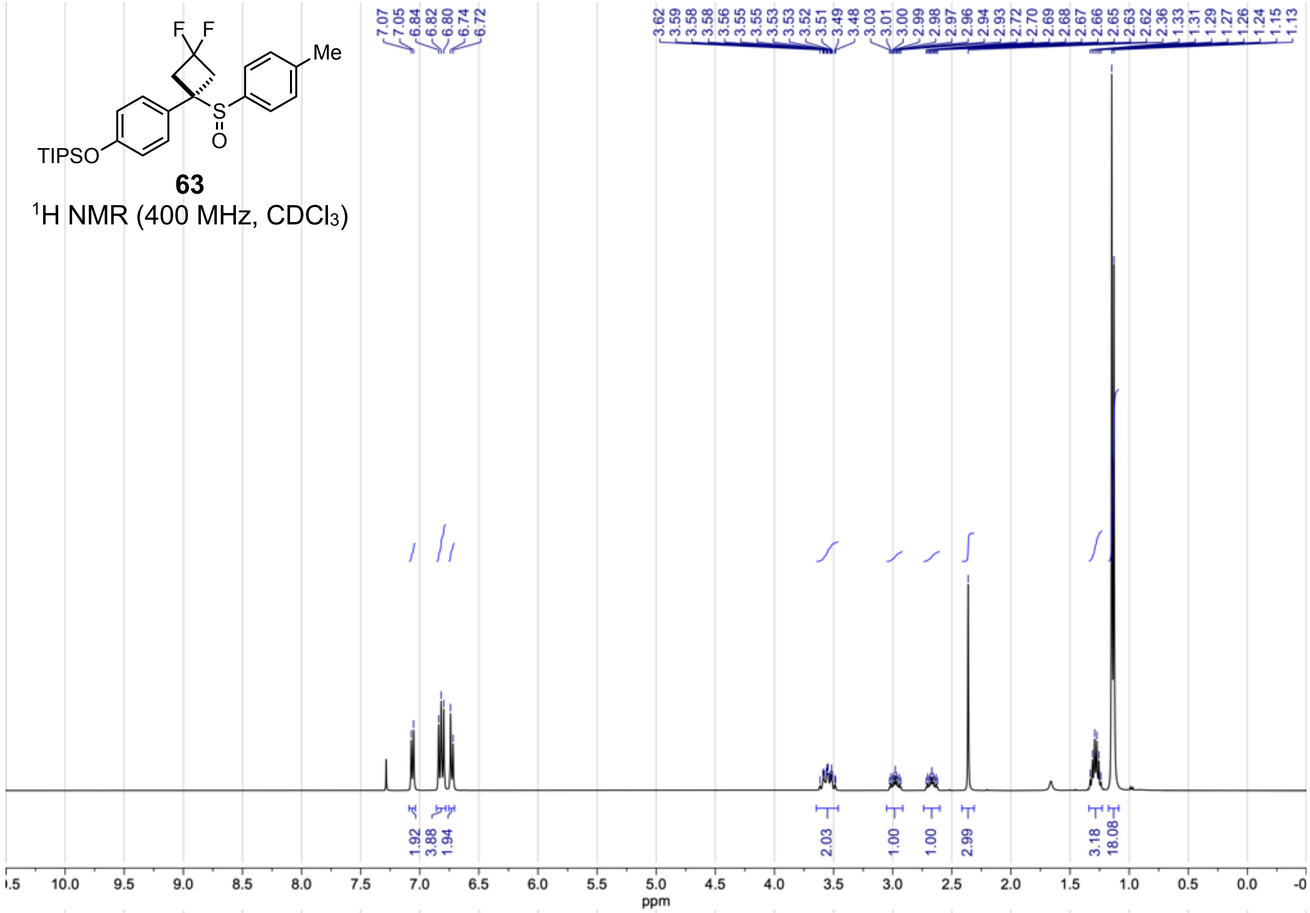

63

^{1}H NMR (400 MHz, $CDCl_3$)

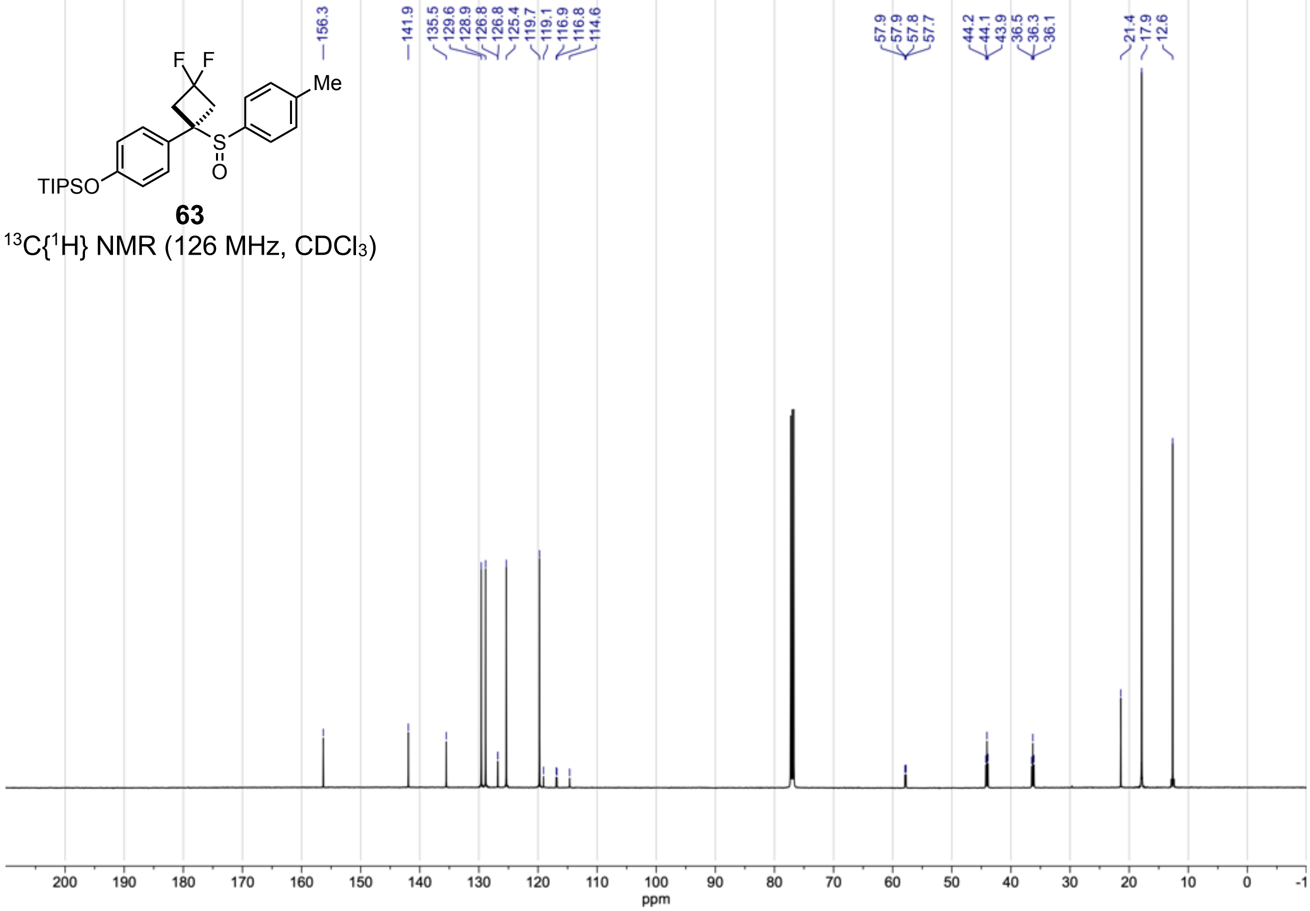

63

$^{13}C\{^{1}H\}$ NMR (126 MHz, $CDCl_3$)

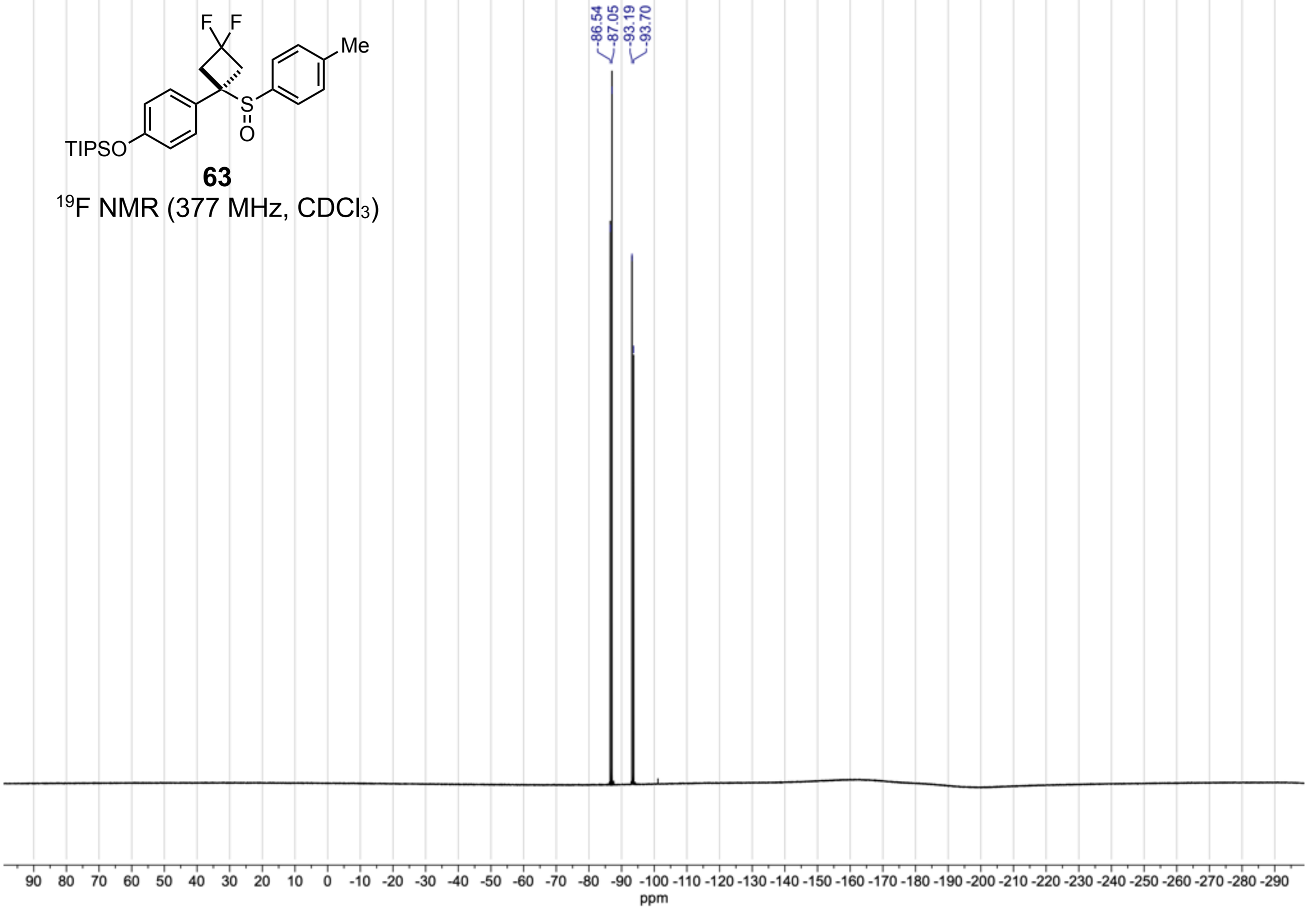
63
^{19}F NMR (377 MHz, $CDCl_3$)
-86.54
-87.05
-93.19
-93.70
90 80 70 60 50 40 30 20 10 0 -10 -20 -30 -40 -50 -60 -70 -80 -90 -100 -110 -120 -130 -140 -150 -160 -170 -180 -190 -200 -210 -220 -230 -240 -250 -260 -270 -280 -290
ppm

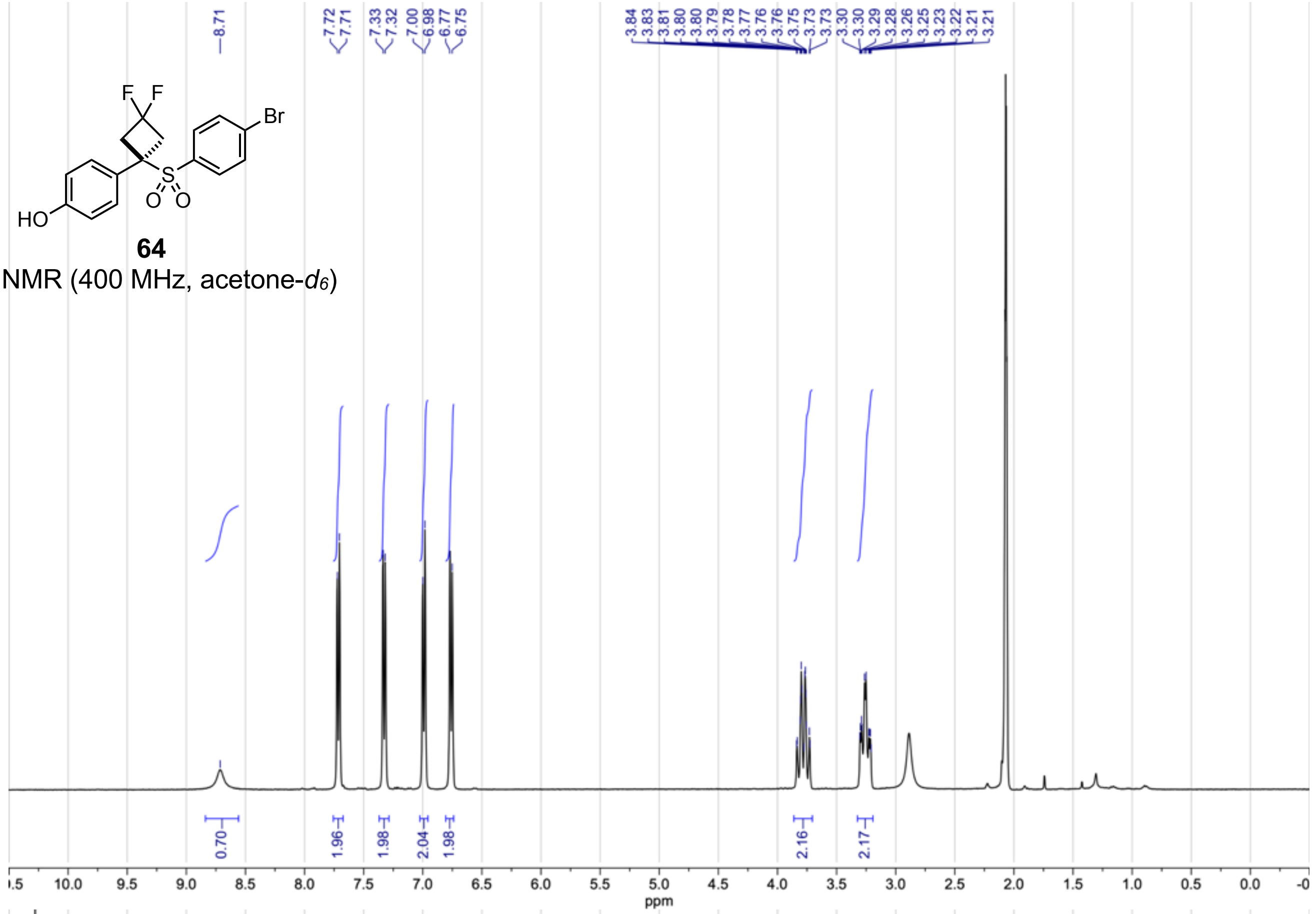

64
^{1}H NMR (400 MHz, acetone-d_6)

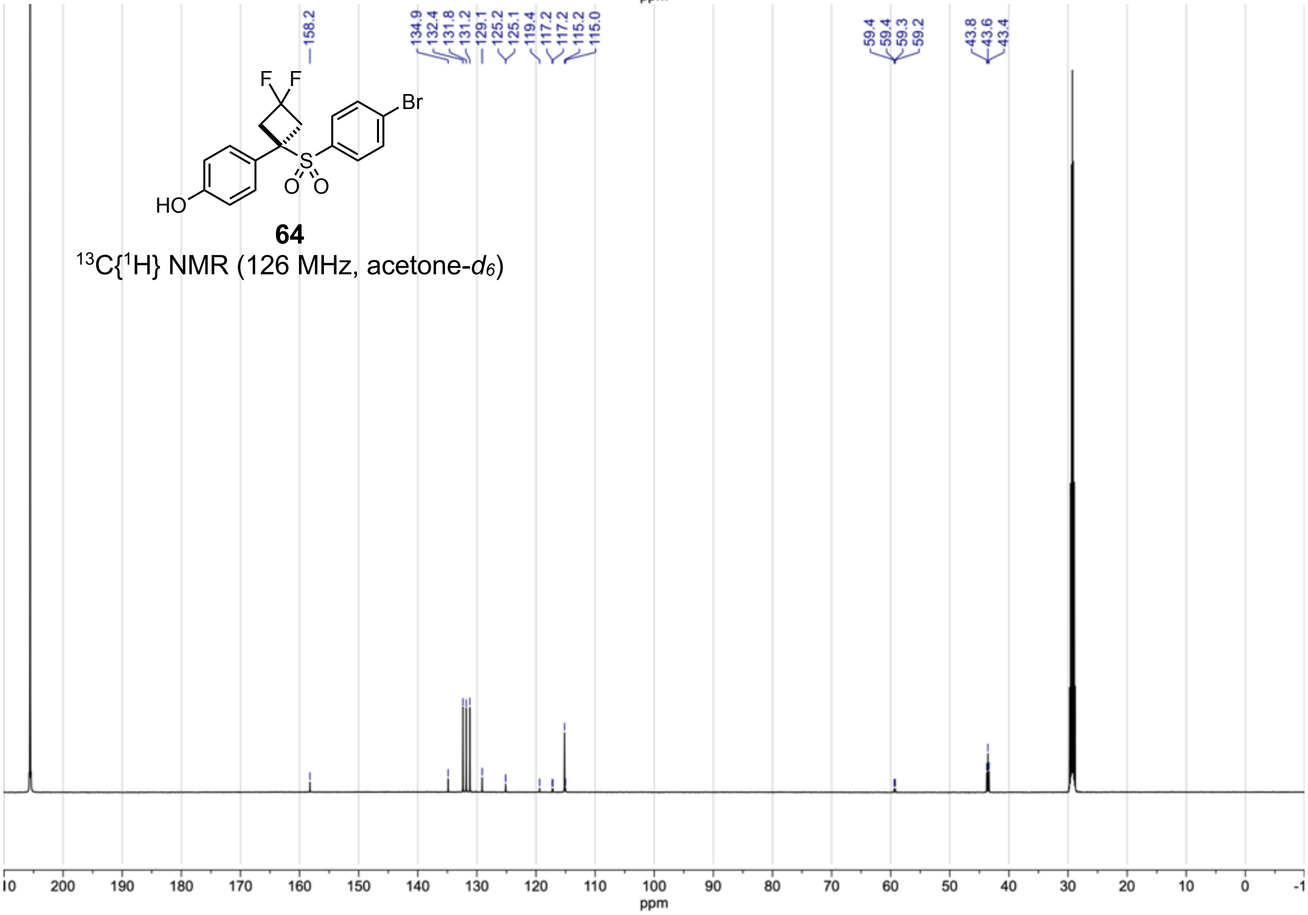

64
^{13}C{^{1}H} NMR (126 MHz, acetone-d_6)

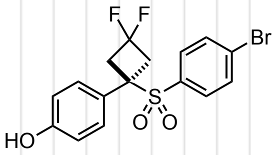

64

^{19}F NMR (377 MHz, acetone-d_6)

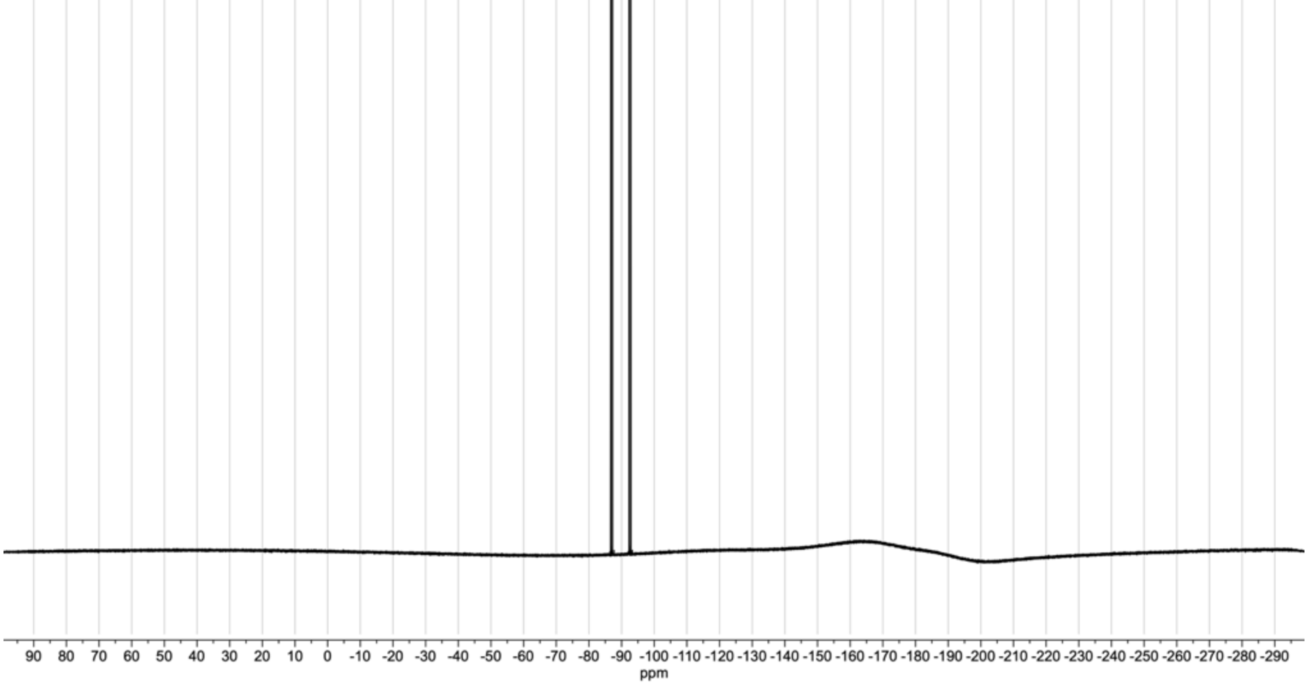

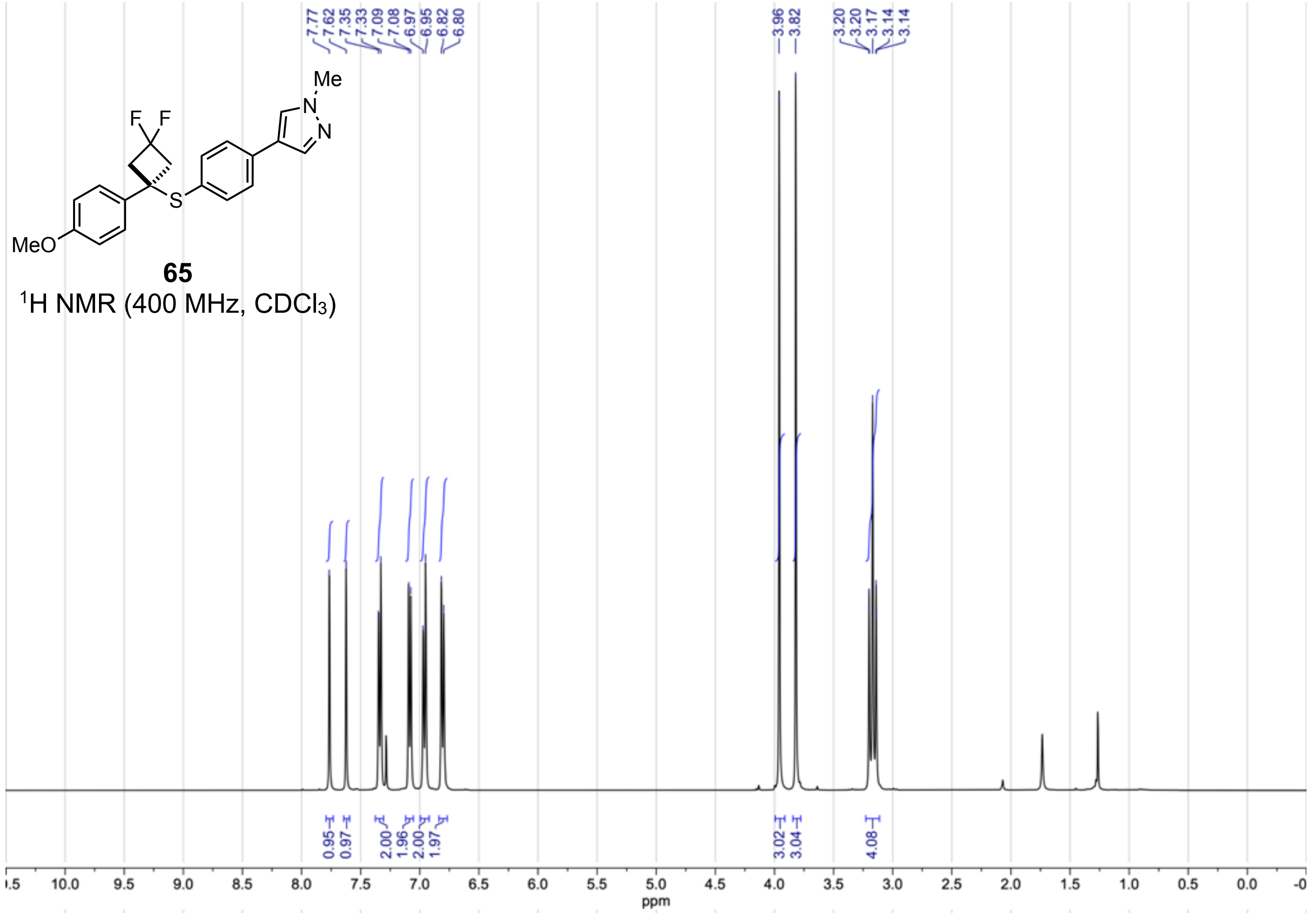

65

^{1}H NMR (400 MHz, $CDCl_3$)

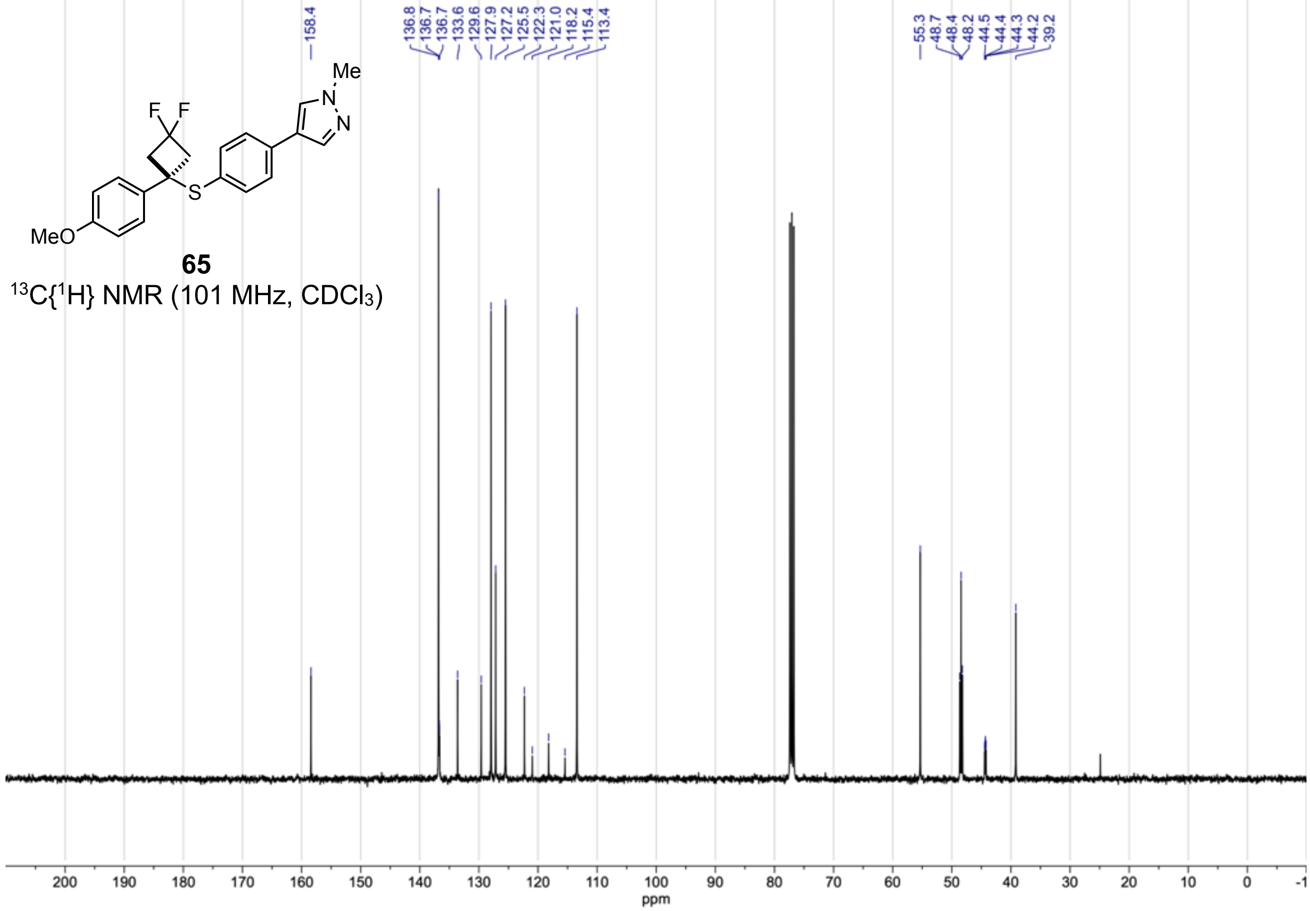

65

$^{13}C\{^{1}H\}$ NMR (101 MHz, $CDCl_3$)

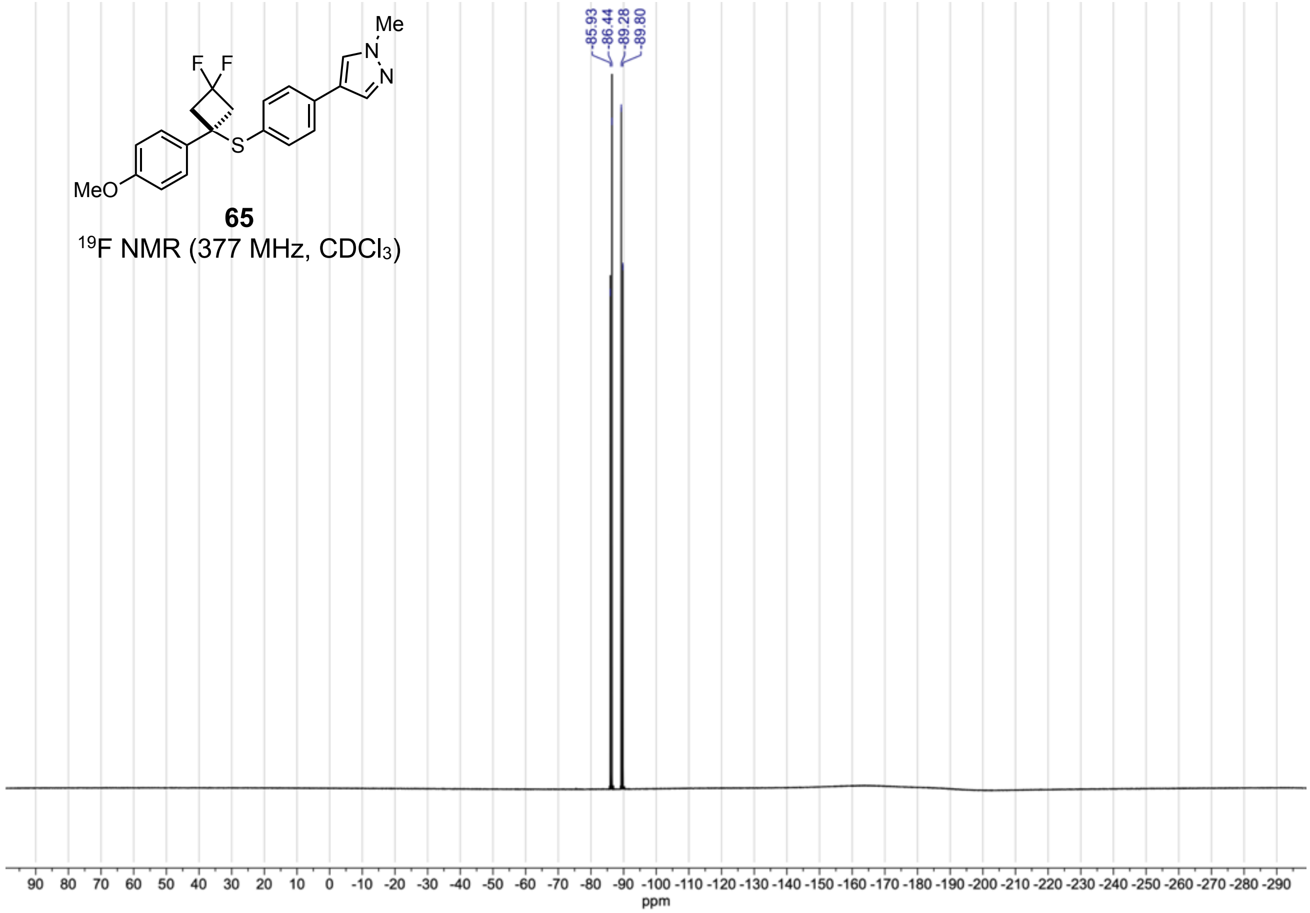

Me
N
N
F F
S
MeO
65
19F NMR (377 MHz, CDCl3)
-85.93
-86.44
-89.28
-89.80
90 80 70 60 50 40 30 20 10 0 -10 -20 -30 -40 -50 -60 -70 -80 -90 -100 -110 -120 -130 -140 -150 -160 -170 -180 -190 -200 -210 -220 -230 -240 -250 -260 -270 -280 -290
ppm

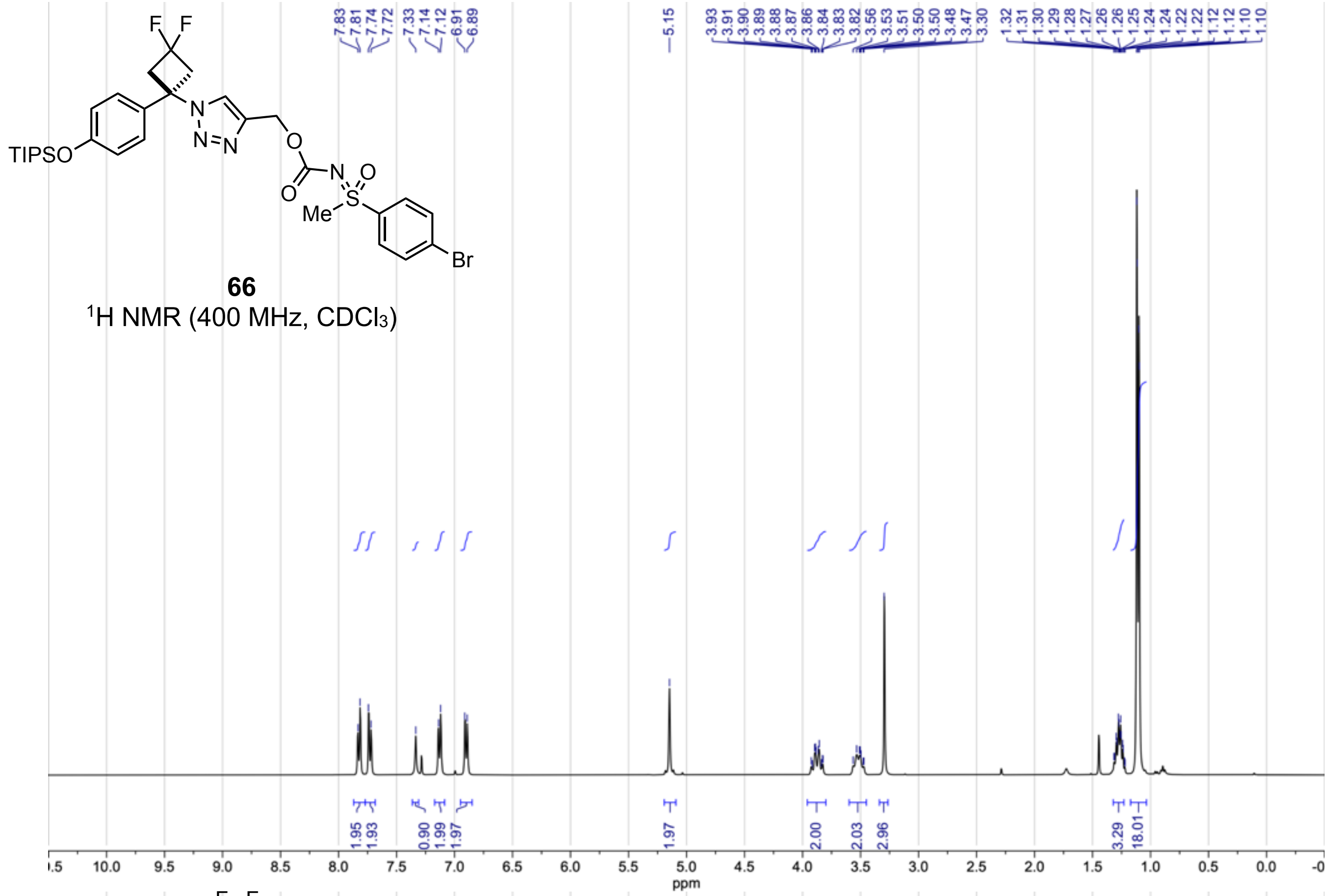

66

^{1}H NMR (400 MHz, $CDCl_3$)

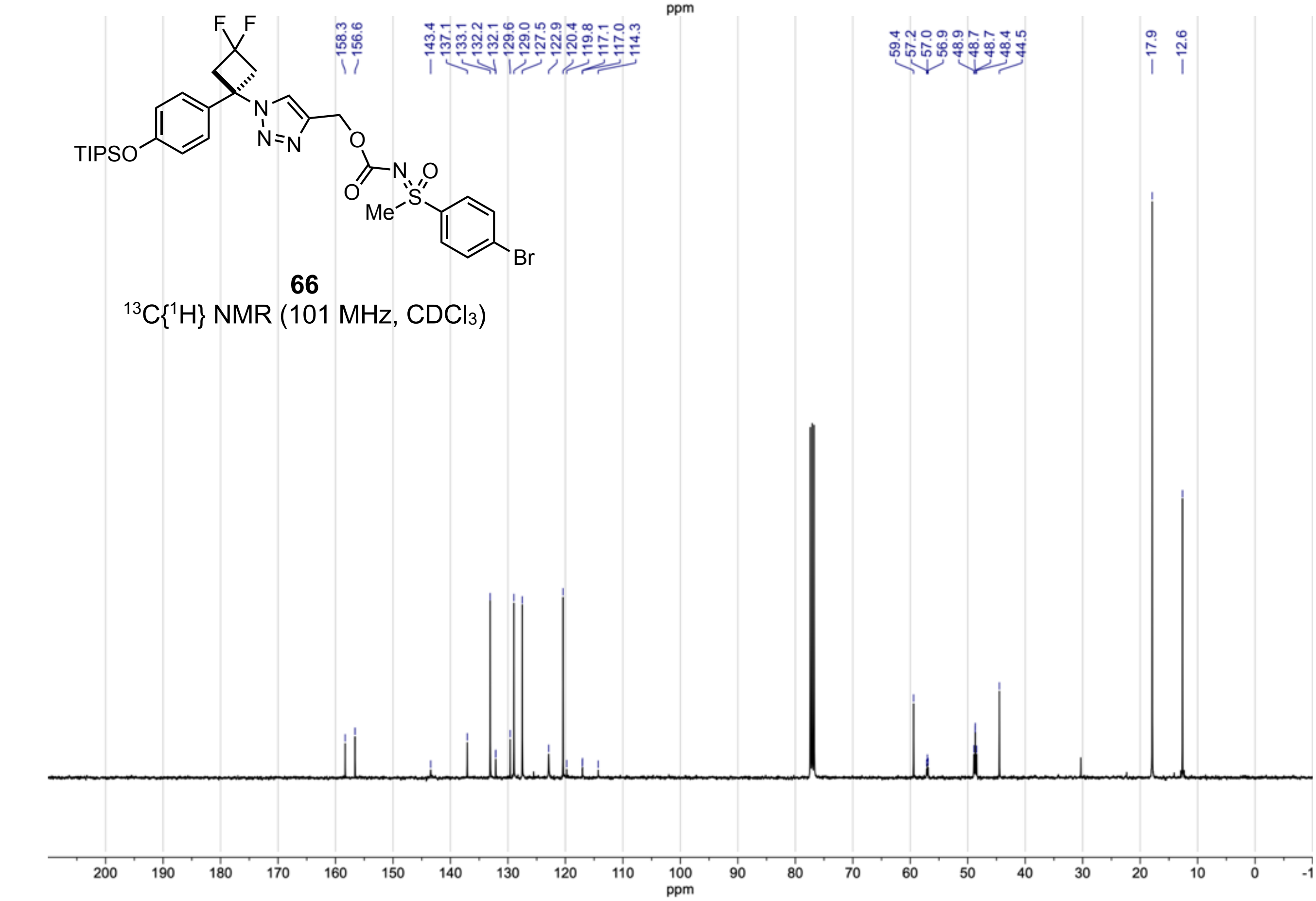

66

$^{13}C\{^{1}H\}$ NMR (101 MHz, $CDCl_3$)

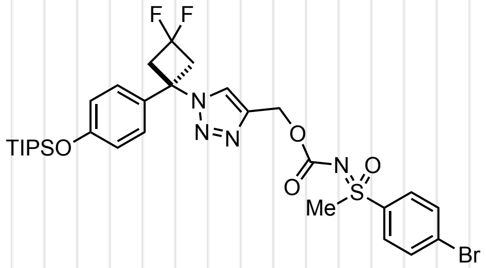

66

^{19}F NMR (377 MHz, $CDCl_3$)

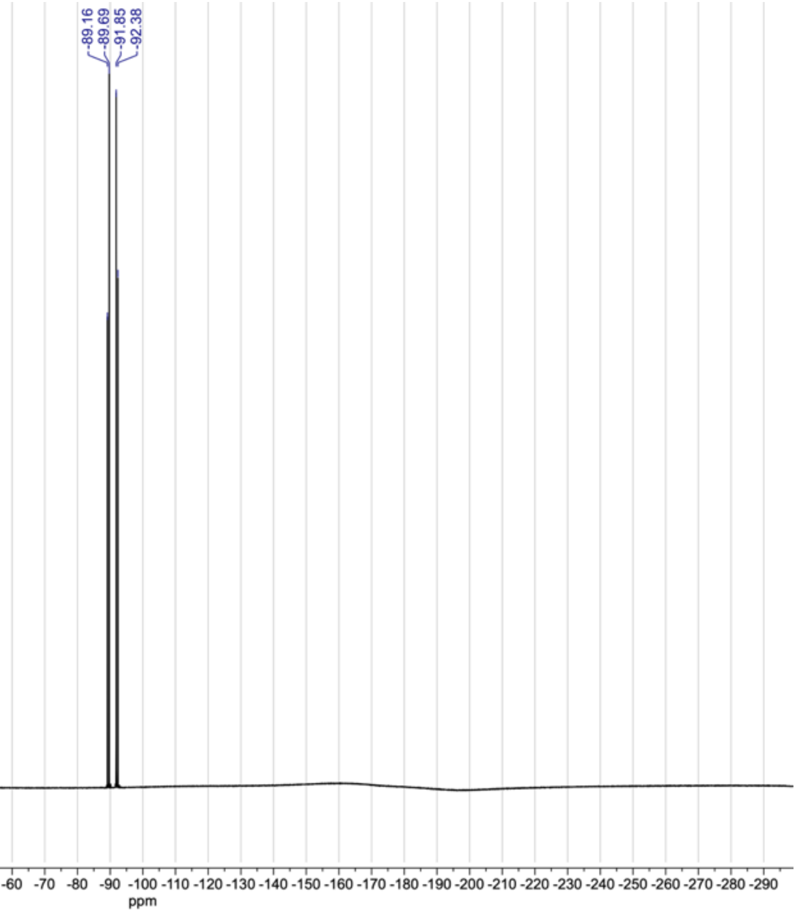

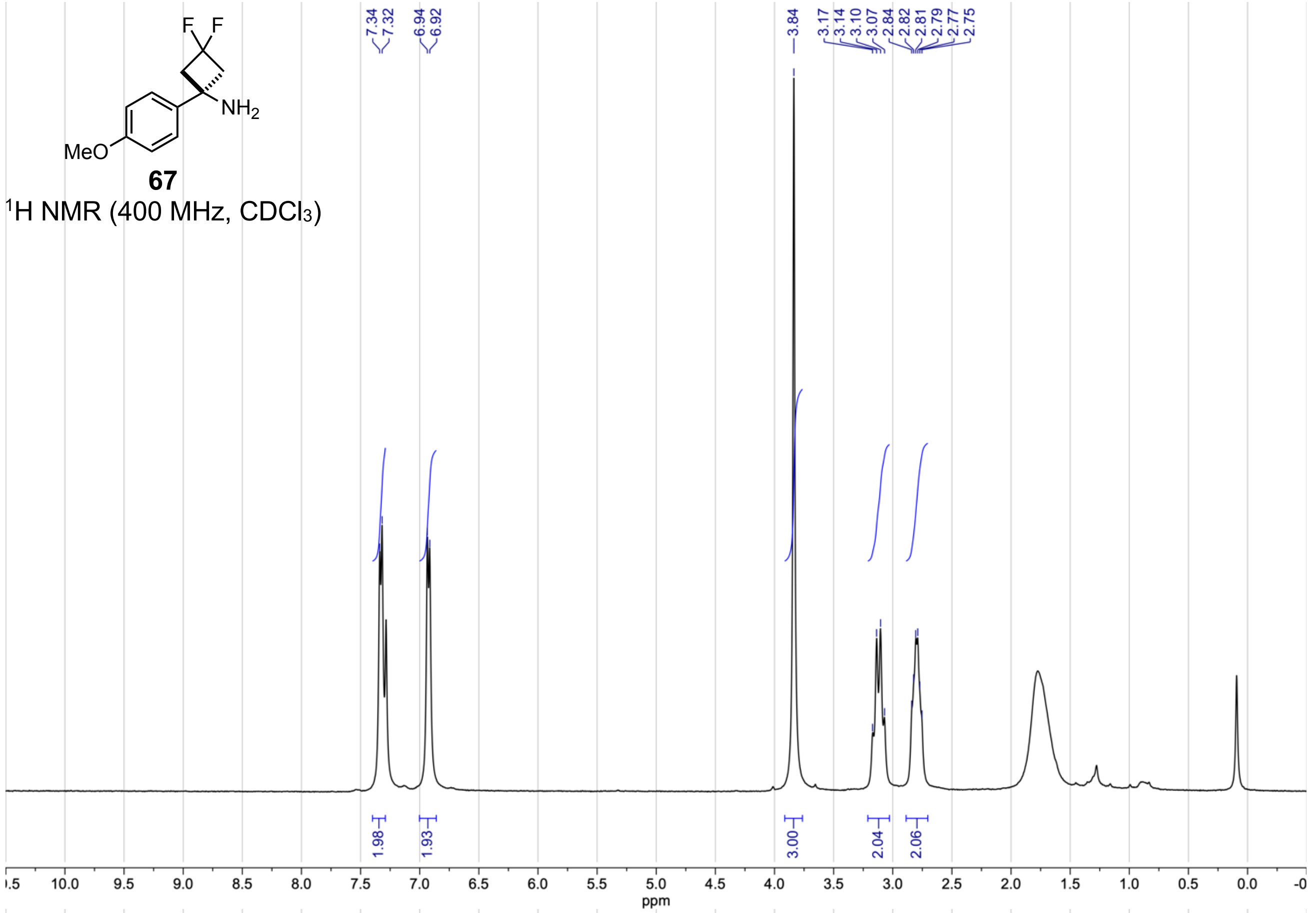

67

^{1}H NMR (400 MHz, $CDCl_3$)

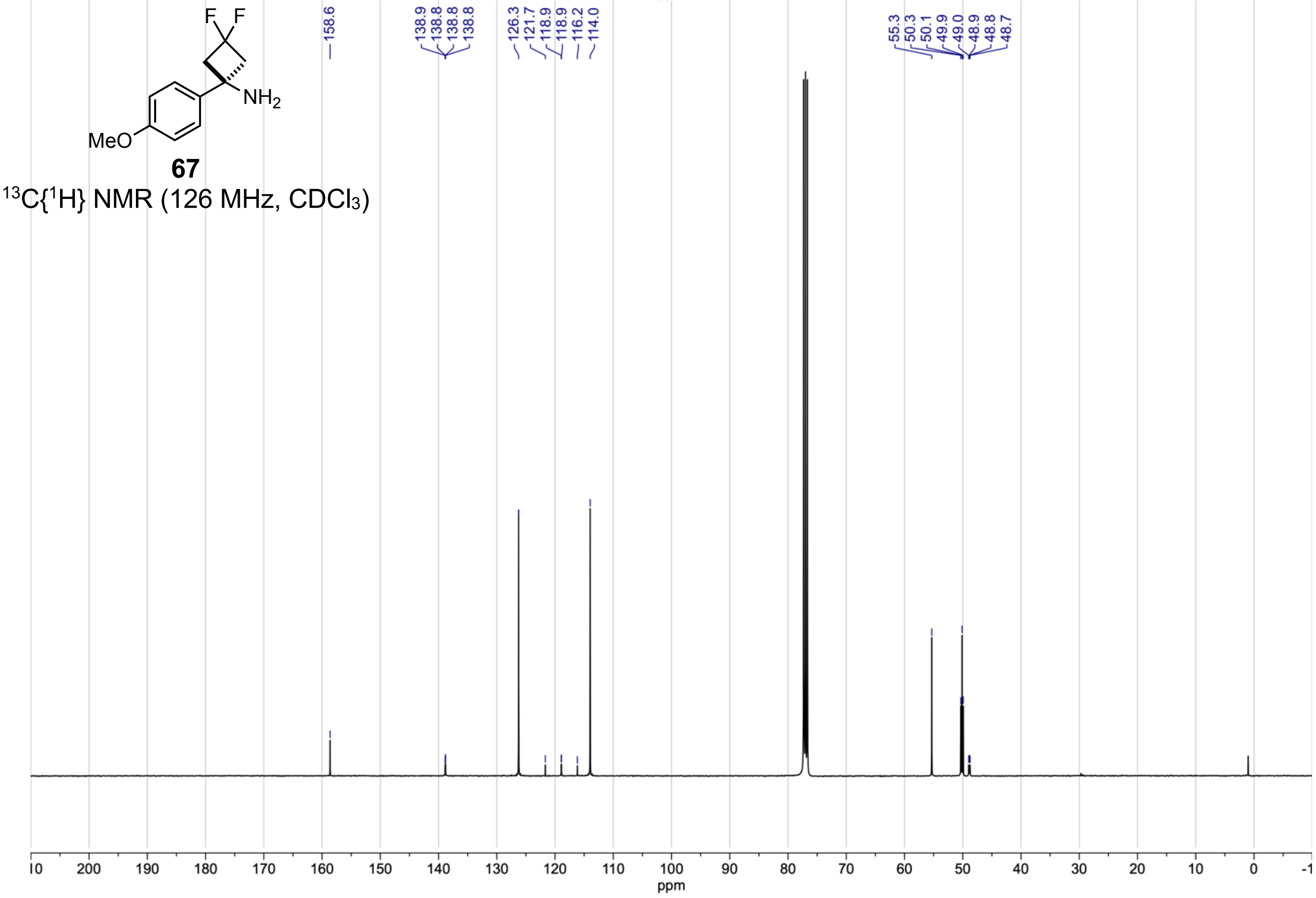

67

^{13}C{^{1}H} NMR (126 MHz, $CDCl_3$)

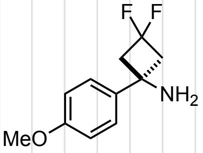

67

^{19}F NMR (377 MHz, $CDCl_3$)

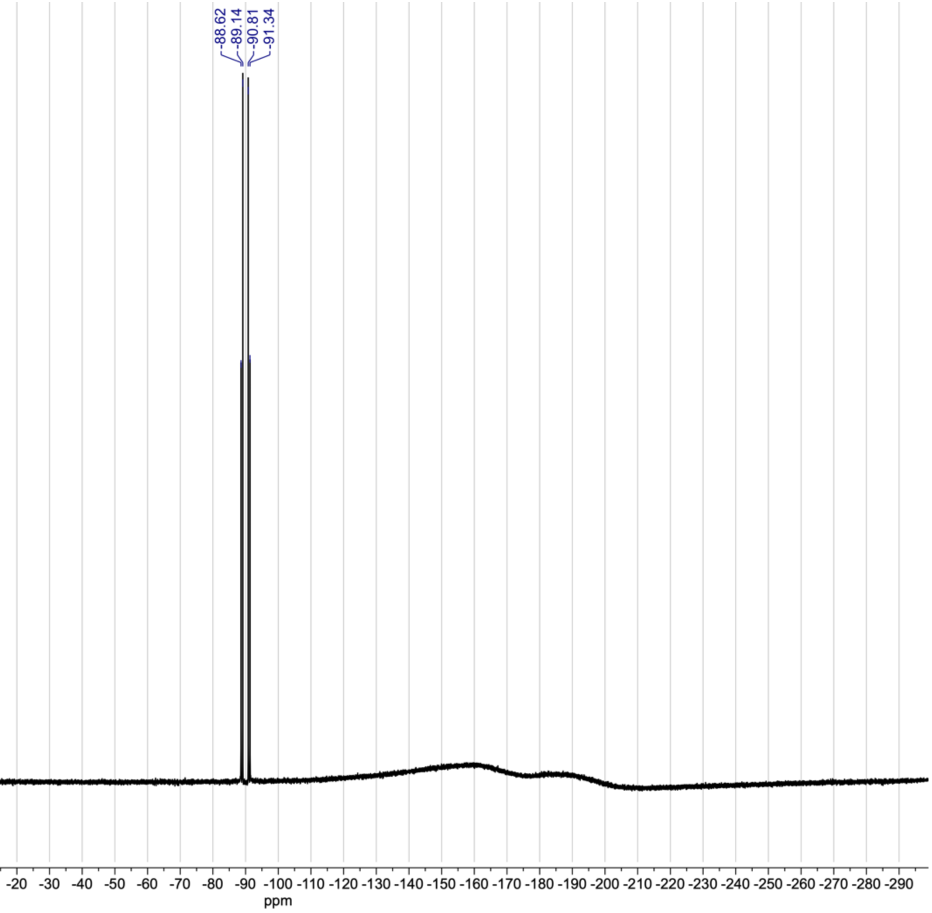

Supplement: Supplementary file 1 [file jo5c01175_si_001.pdf]
